# Proteomic and metabolomic analysis reveals new insights into quaternary amine metabolism in *Citrobacter amalonaticus* CJ25

**DOI:** 10.1128/msphere.00421-25

**Published:** 2025-08-25

**Authors:** Roshan Timsina, Ryan A. Gora, Donald J. Ferguson

**Affiliations:** 1 Department of Microbiology, Miami University 6403, Oxford, Ohio, USA; 2 Department of Biological Sciences, Miami University Regionals, Hamilton, Ohio, USA; Kansas State University, Manhattan, Kansas, USA

**Keywords:** quaternary amines, atherosclerosis, *Citrobacter amalonaticus *CJ25, non-atherogenic, dehydrogenases

## Abstract

Gut microbiota and human physiology are closely linked, and our microbiota can influence disease through immunological and metabolic activity. Quaternary amines, such as choline and carnitine, are abundant in the human gut and are found in red meat, beef, eggs, seafood, wheat, and beets. Canonically, choline and carnitine are broken down into the pro-atherogenic metabolite, trimethylamine (TMA), by various microbes in the human gut. A glycyl radical enzyme, CutC, is involved in the breakdown of choline to TMA. Carnitine is broken down to TMA via a gamma-butyrobetaine intermediate. TMA from the human gut increases levels of TMA *N*-oxide in blood and promotes atherosclerosis. *Citrobacter amalonaticus* CJ25, a gut strain isolated and characterized in our lab, has been shown to grow on choline or carnitine as the sole carbon-energy source without generating TMA. Because the genome lacks canonical enzymes involved in the degradation of choline and no TMA was produced in both choline and carnitine growth conditions, we analyzed the choline and carnitine metabolism using a combined metabolomic and proteomic approach. CJ25 metabolizes choline and carnitine into glycine betaine (GB) via pathways involving novel enzyme homologs, as indicated by proteomic analysis. The proteomics showed putative dehydrogenases that could be oxidizing choline and carnitine to GB. These non-atherogenic pathways involving novel enzyme homologs that we identify in CJ25 may also exist in other gut microbiota, which could amplify the effects of these pathways significantly, possibly reducing the risk of atherosclerotic cardiovascular disease in individuals harboring these microbiota.

**IMPORTANCE:**

The human gut microbiome has been shown to contribute to atherosclerotic cardiovascular disease with adverse health effects throughout the world. Gut microbes canonically metabolize quaternary amines into proatherogenic TMA. In this study, a gut bacterium, CJ25, metabolizes choline and carnitine to a non-atherogenic product, glycine betaine, potentially using novel dehydrogenase homologs for their oxidation. Notably, the ability of CJ25 to metabolize choline and carnitine in a non-atherogenic manner establishes its potential as a beneficial human gut bacterium. Additionally, enzymes identified in CJ25 for choline and carnitine breakdown may be present in other gut microbes, which could amplify the effects of these pathways and reduce the risk of atherosclerotic cardiovascular disease more universally.

## INTRODUCTION

The gut microbiome exhibits complex interactions with human physiology. These complex interactions can influence susceptibility to disease through immunological and metabolic activities. Food ingested can function as dietary antigens and growth substrates for various microbes in our gut (1). Quaternary amines (QAs) are one of the substrates that gut microbes use because of their relative abundance in the gut. They are abundant in red meat, eggs, seafood, wheat, and beets (2). QAs are ammonium salts having a positively charged nitrogen group surrounded by four side chains. Several QAs like choline (3), glycine betaine (GB) (4), carnitine, and gamma-butyrobetaine (GBB) (5) are utilized anaerobically by gut microbes, generating a byproduct trimethylamine (TMA). TMA is absorbed in the intestinal epithelium and transported to the liver via the bloodstream, which is further oxidized to trimethylamine *N*-oxide (TMAO) by the enzyme flavin monooxygenase. Increased TMAO levels in the bloodstream enhance the risks of atherosclerotic cardiovascular (CV) disease (6–9). Stubbs et al., 2015 (9) showed that serum levels of TMAO in chronic kidney disease increase the risk of atherosclerosis burden. Similarly, microbiota-dependent metabolites TMA and TMAO were associated with disease severity in patients with chronic heart failure (8). This clearly shows that the microbes degrade QAs in the diet to the proatherogenic metabolites TMA and TMAO. There is therefore great interest in exploring the routes of microbiota-dependent QA degradation in the human gut due to their relation to adverse CV events (10).

Choline and carnitine are QAs that play an important role in human physiology. Choline is obtained from a variety of food sources, such as eggs and meats (11). It is involved in the synthesis of the neurotransmitter acetylcholine and phosphatidylcholine, a major component of the cell membrane (12). *L*-carnitine is obtained from animal products such as meat, particularly red meat (13), and is involved in the metabolism of fatty acids by transporting them to the mitochondria for beta-oxidation. Enzymes involved in the microbial breakdown of QAs in our gut are still understudied and not fully characterized. Choline is canonically broken down anaerobically by a glycyl radical enzyme CutCD into TMA in the human gut. More specifically, CutC breaks the choline down to TMA and acetaldehyde, where CutD is an accessory factor for CutC (3). It can also be aerobically oxidized to GB by *Pseudomonas aeruginosa*, where GB could be used as an osmolyte or as a methyl donor (14). *Pseudomonas aeruginosa* converts choline to GB via a two-step reaction involving enzymes such as choline dehydrogenase (BetA) and betaine aldehyde dehydrogenase (BetB) (15). Studies have also suggested that choline is converted to glycine and ultimately to pyruvate via GB, with glycine and pyruvate contributing to the amino acid pool and central metabolism, respectively. Microbial carnitine metabolism can also occur in both aerobic and anaerobic environments. Carnitine can be converted to TMA using the monooxygenase enzyme in an aerobic environment. In an anaerobic environment, *Escherichia coli* converts carnitine to crotonobetaine and subsequently GBB via carnitine dehydratase and crotonobetaine reductase, respectively (16). In a gut bacterium, *Emergencia timonensis*, *L*-carnitine-derived GBB has been shown to produce TMA via a GBB utilization cluster (5). In *Pseudomonas aeruginosa*, carnitine is catabolized to GB via 3-dehydrocarnitine and betainyl CoA intermediate using a carnitine dehydrogenase, beta-ketoacid cleavage enzyme (BKACE), and the DhcAB enzyme system, respectively (17–19). Furthermore, in the gut bacterium *Eubacterium limosum*, quaternary amines such as carnitine, GBB, and proline betaine are demethylated, circumventing TMA production using unique pyrrolysine-lacking enzymes of the COG5598 superfamily (20–22).

*Citrobacter amalonaticus* CJ25, a gram-negative rod-shaped bacterium, is an organism of interest because of its unique ability to break down choline without producing a proatherogenic metabolite, TMA (23). It was originally isolated from a fecal sample from a healthy human volunteer, and its genome has been sequenced. This strain was shown to anaerobically use choline or carnitine as a sole source of carbon and energy (23), but the mechanism of this breakdown is still not understood. Though its genome does not encode a canonical CutC enzyme and no known corrinoid-dependent methyltransferase of the COG5598 superfamily, it can still break down choline. We are interested in determining the pathways for the breakdown of choline and carnitine in this organism. Because TMA was not detected in cultures grown on choline or carnitine, we hypothesized that CJ25 converts choline and carnitine into a non-atherogenic metabolite using novel quaternary amine degradation pathways.

**Fig 1:**
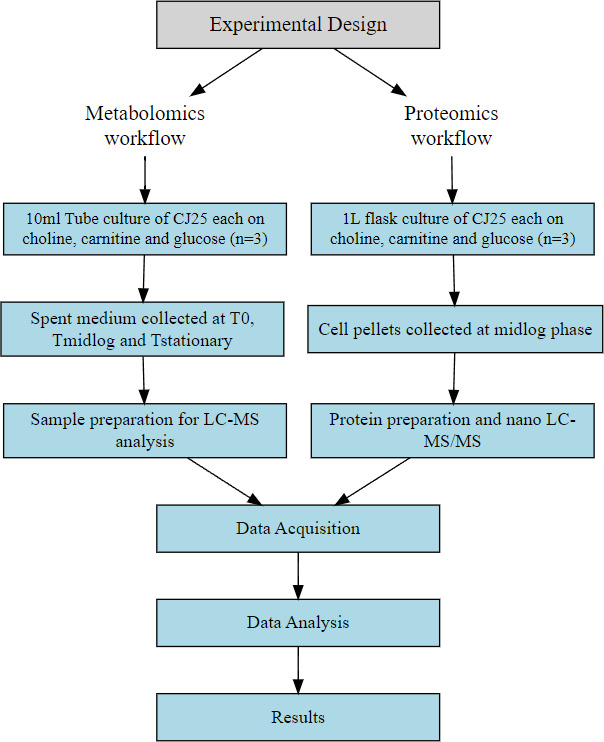
The experimental workflow of the sample preparation, metabolomic, and proteomic analysis.

In the present study, we used liquid chromatography-mass spectrometry (LC-MS/MS) analysis and nano liquid chromatography-tandem mass spectrometry (nano-LC-MS/MS; Fig. 1) to describe and integrate the data from metabolomics and proteomics to identify the downstream metabolites and enzymes that could be responsible for the breakdown of choline and carnitine. We also proposed hypothetical pathways of choline and carnitine catabolism.

## MATERIALS AND METHODS

### Anaerobic media components, culturing conditions, and sample collection

One liter of culture medium contained: 1.0 g NH_4_Cl_2_, 0.1 g NaCl, 0.1 g MgCl_2_·6H_2_O, 0.05 g CaCl_2_, 1× SL-10 trace elements solution (DSMZ medium 722), 1× selenite-tungstate solution (DSMZ medium 385), and resazurin (0.1% [wt/vol]). The medium was sparged with ultra-high purity 100% N_2_ gas for 30 min. Inside an anaerobic chamber, the following components were added to the base medium: 2 mM Dithiothreitol (DTT); 3 mM cysteine-HCl; 0.30 g Na_2_S·9H_2_O. For the preparation of 10 mL tube cultures, 10 mL of medium was aliquoted in 27 mL glass Balch-type tubes, sealed with butyl rubber stoppers, and capped with aluminum crimp caps. The headspaces of the tubes were exchanged with 100% N_2_ for three 1 min gas exchange cycles on a vacuum and gas manifold system. The tubes were autoclaved and stored at room temperature (23). For the preparation of flask cultures, the base medium was autoclaved first and sparged with ultra-high purity 100% N_2_ gas for 30 min. Inside an anaerobic chamber, 2 mM DTT, 3 mM cysteine-HCl, and 0.30 g Na_2_S·9H_2_O were added to the base medium using a sterile filter of 0.2 µm size. All chemical reagents were purchased from Sigma-Aldrich or Fisher Scientific with a purity of >98%.

The 10 mL culture tubes were inoculated, in triplicate, with 100 µL inoculum from the previous pure culture of CJ25. The media tubes were supplemented with 10 mM of choline (only for choline growth condition), 30 mM Na_2_SO_3_, 1× Vitamins (DSMZ medium 141), and 22 mM KH_2_PO_4_ buffer pH 7.2. Similar supplementation parameters were used for carnitine and glucose growth conditions, with the appropriate substrate added. The culture tubes were incubated at 37°C, and the absorbance was monitored at 600 nm by placing tubes directly into a Spectronic 20. For the metabolomic analysis, spent medium samples were collected at time points (0 hour), mid-log, and stationary phase. The collected samples were centrifuged, and the supernatant was filtered using a filter size of 0.2 µm. The filtered supernatants were stored at −80°C prior to sample preparation for LC-MS/MS.

A one-liter flask culture was inoculated with 10 mL of tube media at the mid-log phase for proteomic analysis. The absorbance of the flask culture was monitored by sampling 1 mL of culture in a cuvette at 600 nm by placing the cuvettes directly into a Spectronic 20. The culture was harvested at mid-log and centrifuged at 10,000 × *g* to collect the cell pellets. The cell pellets were stored at −80°C prior to sample preparation for nano-LC-MS/MS.

### Protein preparation and nano-LC-MS/MS analysis for proteomics

Cell pellets were resuspended in 100 µL of 5% SDS buffer in 50 mM TEAB (triethylammonium bicarbonate) solution. Samples were then vortexed briefly before proceeding to sonication by using the Bioruptor Pico (Diagenode, Denville, NJ), following the manufacturer’s suggested protocol. Briefly, the sonication temperature was set at 4°C; the sonication cycle was set at 30 s on and 30 s off. A total of 10 cycles were done for the cell pellet. After sonication, samples were centrifuged at 15,000 × *g* for 15 min at 4°C to remove any remaining insoluble material. The concentration of the proteins was measured using a Qubit fluorometer (ThermoFisher Scientific).

Fifty microgram of the samples was taken for trypsin digestion. Five microliter of 50 mM Ammpnium bicarbonate (ABC) containing 5 µg/µL DTT was added, and the sample was incubated at 65°C for 15 min, followed by the addition of 5 µL of 50 mM ABC containing 15 µg/µL iodoacetamide (incubated at Room temperature [RT] for 15 min in the dark). Samples were then acidified by adding 12% phosphoric acid (1:10 [vol/vol] acid to sample). For every 25 µL of samples, 165 µL of TEAB 1 M/MeOH (10:90 [vol/vol]) was added and then loaded to the S-trap for further washes. Samples were centrifuged at 4,000 × g for 3 min (4°C) to remove supernatant. A total of 150 µL of TEAB was added to the trap as a wash solution, and the trap was washed 3–6 times, depending on the initial loading volume. After the final wash, sequencing grade trypsin dissolved in 50 mM TEAB was added and digested overnight at 37°C. The following day, peptides were eluted from the trap by sequentially adding 40 µL of 50 mM TEAB, 0.1% Formic Acid (FA), and 0.1% FA in acetonitrile. The sample was pooled together, dried in a vacufuge, and resuspended in 20 µL of 50 mM acetic acid. The peptide concentration was determined by nanodrop (A280nm).

Nano-LC-MS/MS of protein identification was performed on a Thermo Scientific Orbitrap Fusion mass spectrometer equipped with a nanospray FAIMS Pro Sources operated in positive ion mode. Samples (4.0 µL) were separated on an easy spray nano column (Pepmap^T^ RSLC, C18 3µ 100A, 75 µm × 150 mm, Thermo Scientific) using a 2D RSLC HPLC system from Thermo Scientific. Each sample was injected into the µ-Precolumn Cartridge (Thermo Scientific) and desalted with 0.1% formic acid in water for 5 min. The injector port was then switched to inject, and the peptides were eluted off from the trap onto the column. Mobile phase A was 0.1% formic acid in water, and acetonitrile (with 0.1% formic acid) was used as mobile phase B. Flow rate was set at 300 nL/min. Mobile phase B was increased from 2% to 16% in 105 min and then increased from 16% to 25% in 10 min and again from 25% to 85% in 1 min and then kept at 95% for another 4 min before being brought back quickly to 2% in 1 min. The column was equilibrated at 2% of mobile phase B (or 98% A) for 15 min before the next sample injection. MS/MS data were acquired with a spray voltage of 1.95 kV, and a capillary temperature of 305°C was used. The scan sequence of the mass spectrometer was based on the preview mode data dependent TopSpeed method: the analysis was programed for a full scan recorded between *m/z* 375–1,500 and a MS/MS scan to generate product ion spectra to determine amino acid sequence in consecutive scans starting from the most abundant peaks in the spectrum in the next 3 s. To achieve high mass accuracy MS determination, the full scan was performed at Fourier transform (FT) mode, and the resolution was set at 120,000 with internal mass calibration. Three FAIMS compensation voltage (cv = −50, −65, and −80 V) were used for data acquisition. The Automatic Gain Control (AGC) target ion number for FT full scan was set at 4 × 10^5^ ions, maximum ion injection time was set at 50 ms, and micro scan number was set at 1. MSn was performed using High Energy Collisional Dissociation (HCD) in ion trap mode to ensure the highest signal intensity of MSn spectra. The HCD collision energy was set at 32%. The AGC Target ion number for ion trap MSn scan was set at 3.0E4 ions, maximum ion injection time was set at 35 ms, and micro scan number was set at 1. Dynamic exclusion is enabled with a repeat count of 1 within 60 s and a low mass width and high mass width of 10 ppm.

### Sample preparation and LC-MS/MS analysis for metabolomics

Spent medium samples were thawed on ice, and 20 µL of spent medium samples was added to 80 µL of MeOH and vortexed for 10 s and sonicated for 3 min. Then, the samples were spun at 20,000 rcf at 4°C for 10 min. The supernatant was collected, and 5 µL was injected into the LC/MS.

Instrumental analysis was performed on a Vanquish UHPLC coupled to an Orbitrap Exploris 480 mass spectrometer (ThermoFisher Scientific, Waltham, MA) equipped with high flow and low flow heat-electrospray ionization probes. Chromatographic separations were performed using an Agilent InfinityLab Poroshell 120 Hilic 2.7 µm × 2.1 × 100 mm column at a flow rate of 0.2 mL min^–1^. The mobile phase system consisted of water (A) and acetonitrile (B), both acidified with 0.1% of formic acid, using the following gradient elution: 0 min, 100% B; 15 min, 0% B. A column temperature of 40°C and an injection volume of 5.0 µL were used during the analysis.

The global settings for the MS were as follows: the instrument was operated in a positive mode with a positive ion spray voltage of 3.6 kV (3.0 kV for negative mode). Sheath gas, auxiliary gas, and sweep gas were set at 35, 10, and 1 arbitrary units, respectively, while both the ion transfer tube temperature and vaporization temperature were set at 350°C. AcquireX Deep Scan was used to acquire data. Full scan MS spectra in triplicate and three MS/MS spectra were recorded in the range of 50–750 *m*/*z*. The mass spectrometer calibration in the low mass and high mass range was performed with the Pierce FlexMix calibration (ThermoFisher Scientific, Waltham, MA).

### Data processing and statistical analysis

For proteomic data analysis, the data were searched using Mascot Daemon by Matric Science version 2.7.0 (Boston, MA) via Proteome Discoverer (version 2.4 Thermo Scientific), and the database was searched against the most recent Uniprot databases. The mass accuracy of the precursor ions was set to 10 ppm, and accidental pick of one ^13^C peak was also included in the search. The fragment mass tolerance was set to 0.5 Da. Carbamidomethylation is used as a fixed modification, and considered variable modifications were oxidation (Met) and deamidation (N and Q). Four missed cleavages for the enzymes were permitted. A decoy database was also searched to determine the false discovery rate (FDR), and peptides were filtered according to 1% FDR. Proteins identified with at least two unique peptides were considered for reliable identification. Any modified peptides were manually checked for validation.

For metabolomic analysis, Compound Discoverer (v3.2, ThermoFisher Scientific, Waltham, MA) was used to perform data processing, including retention time alignment, background removal, compound extraction and classification, compound grouping, chemical formula prediction, and compound annotation using a node-based methodology. The full MS data were used for peak picking, and the ddMS/MS data were used for identification only. The total number of features and the total number of annotations were considered for the characterization of the influence of each full MS parameter, while the number of MS/MS counts, the number of annotated compounds with MS/MS information, and the spectral quality were used to characterize the influence of MS/MS parameters. Following data processing, features were excluded using general filters such as background removal, mass accuracy (delta = ±0.5 ppm), and MS/MS for the preferred ion. Spectral quality was evaluated by matching experimental spectra with the MS/MS spectral library. The mzCloud best match score greater than or equal to 70% was used as the cutoff for spectral similarity. The optimum value for the full MS parameters was defined as the value that provided the highest total number of metabolite annotations within a mass accuracy of 5 ppm and 30% relative SD (RSD) of triplicate measurements. On the other hand, the value that gave the greatest number of annotated compounds with MS/MS information and improved MS/MS spectral quality within a mass accuracy of 5 ppm and 30% RSD of triplicate measurements was identified as the optimum value for the MS/MS parameters. The compounds’ annotation was performed after subtracting the background of uninoculated media from the experimental data to remove any background signals from the media components.

Metabolomic data were analyzed using auto-scaling and log transformation. Principal component analysis was performed by R 4.4.1 using the xcms package. All data visualization and statistical analysis, including heatmaps and scatterplots (using the Mummichog algorithm), were implemented using MetaboAnalyst v6.0, a web-based metabolomics pathway analysis platform (https://www.metaboanalyst.ca/). The significant metabolites were clustered using Euclidean distance and the Ward method to plot heatmaps. All statistical analyses required for the volcano plot for proteomics analysis were implemented in ExpressAnalyst (https://www.expressanalyst.ca/), and the visualization was performed with R 4.4.1. All other data visualization was performed with R 4.4.1. A *P*-value < 0.05 or FDR-corrected *P*(*q*) < 0.05 was considered statistically significant.

### BLAST analysis

BLAST alignments were performed using the NCBI blastp tool. Enzymes encoded in the CJ25 genome were used as target or subject sequences, while previously known enzymes with similar proposed activity were used as query sequences. BLOSUM45 was used as a scoring matrix. The scoring matrices were created using real sequences that shared no more than 45% identity. The BLAST results are expressed in three key components: *e*-value, percent identity, and percent similarity.

## RESULTS

### Untargeted metabolomics reveals unique metabolite production when CJ25 is grown on different substrates

**Fig 2:**
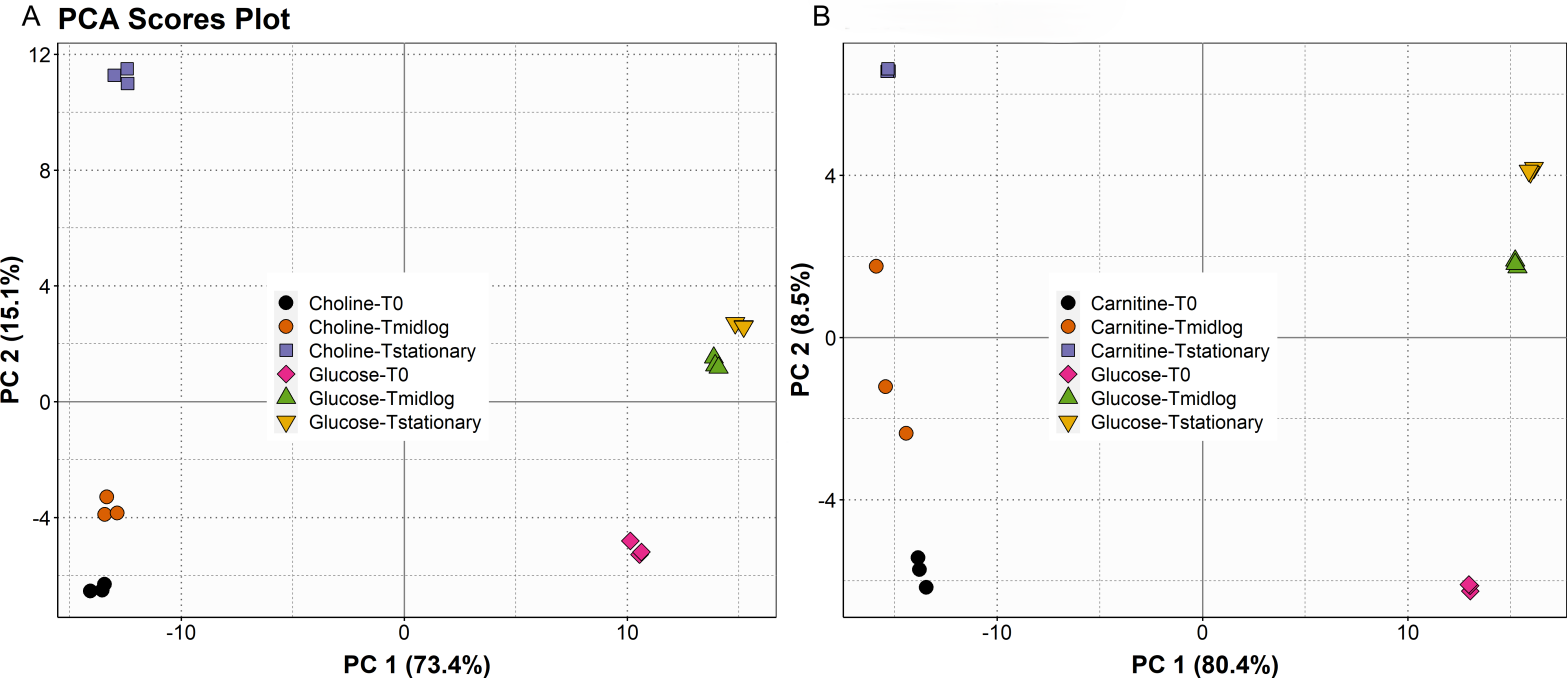
Principal component analysis plots in which extracellular metabolomes were compared between different growth conditions at three different time points (**A**) choline vs glucose and (**B**) carnitine vs glucose.

**Fig 3:**
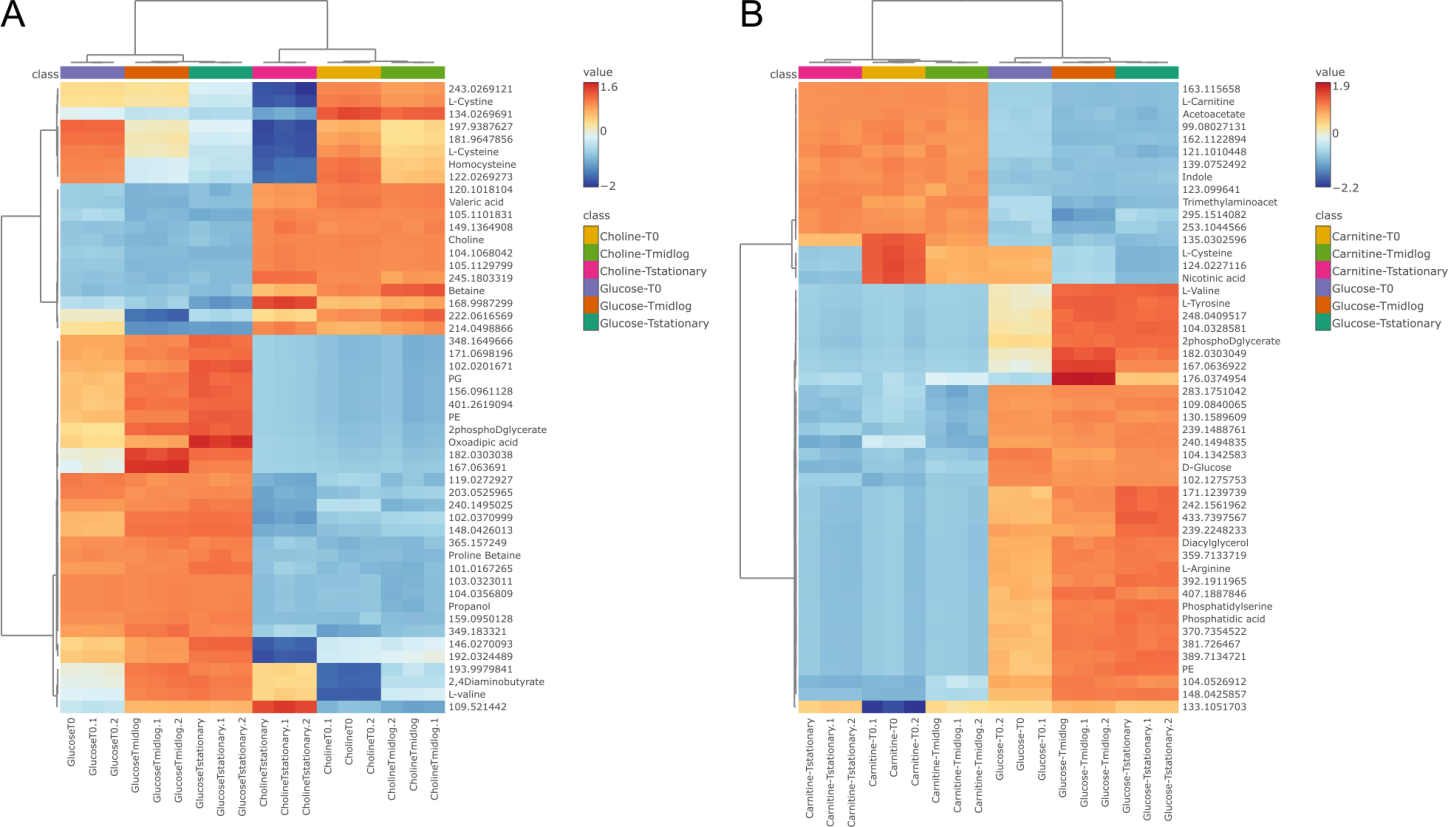
Heatmap of Ward’s hierarchical clustering obtained between extracellular metabolites at various growth conditions at different time points (**A**) choline vs glucose and (**B**) carnitine vs glucose.

We first hypothesized that CJ25 catabolizes choline and carnitine into unknown novel non-atherogenic products because TMA production was not detected in cultures supplemented with choline (23) or carnitine (Fig. S1). We therefore employed a metabolomic approach to identify the range of metabolites produced by CJ25 under different growth conditions. We analyzed the samples to examine the differences in metabolomes during the growth of CJ25 on choline, carnitine, or glucose at three different time points: *T*_0_, *T*_midlog_, and *T*_stationary_. Based on Compound Discoverer, there were 5,664 metabolic features exported from the positive mode, in which 1,403 (24.7%) features have tentative IDs (File S1). We chose to analyze the positive mode because it provided us with the most abundant and comparable information relating to the metabolic profile of different growth conditions at different time points. To comparatively visualize the metabolomes of three different growth conditions, the extracellular metabolites from the positive mode are shown in Supplementary Information (Fig. S2 and S3). A multivariate cluster analysis based on different time points for each growth condition implied that the metabolomes during choline and carnitine growth conditions are notably different from our control substrate glucose. A Principal Component analysis (PCA) plot indicated a distinct separation in metabolite composition at two different growth conditions. In the choline growth condition, PC1 and PC2 explained 73.4% and 15.1% of the total variation, respectively (Fig. 2A). Similarly, in the carnitine growth condition, PC1 and PC2 explained 80.4% and 8.5% of the total variation, respectively (Fig. 2B). The metabolomes in glucose and choline or carnitine growth conditions were significantly separated along PC1. To better show the difference between extracellular metabolites among time points for different growth conditions, 50 significant metabolites were selected for hierarchical Pearson’s clustering. The log transformation of the peak area was done for better visualization. Hierarchical Pearson’s clustering showed significant disparities between the experimental (choline and carnitine growth conditions) and control (glucose growth condition) groups at different time points (Fig. 3A and B). A total of 50 different extracellular metabolites, 14 of them annotated with a *P* < 0.05, fold change >1.5, and *t*-test at the 95% level, are presented in both the heatmaps. In choline vs glucose growth conditions, metabolites, including choline, glycine betaine, and amino acids such as l-cysteine, l-cystine, and homocysteine, were significantly higher in choline growth conditions. Similarly, in carnitine vs glucose growth conditions, metabolites, including l-carnitine, acetoacetate, indole, l-cysteine, nicotinic acid, and trimethyl-aminoacetone, were significantly higher in carnitine growth conditions.

### Untargeted metabolomics provides insights into the metabolic features of CJ25 under different growth substrates

The heatmap provided us with some significant metabolites; we therefore further conducted functional analysis using the mummichog algorithm (24) to detect characteristic metabolic features using Metaboanalyst 6.0 (https://www.metaboanalyst.ca/). Because functional analysis considers differences in particular pathways between two conditions, we chose to compare the mid-log of experimental (choline or carnitine) and control (glucose) growth conditions. Several pathways, such as arginine and proline metabolism, cysteine and methionine metabolism, and biosynthesis of various plant secondary metabolites, showed significant differences in the mid-log of choline and glucose growth conditions (Fig. S4A). Similarly, pathways like pantothenate and CoA biosynthesis, biosynthesis of various plant secondary metabolites, peptidoglycan biosynthesis, phenylalanine, tyrosine, and tryptophan biosynthesis pathways showed significant differences in mid-log of carnitine and glucose growth conditions (Fig. S4B). The functional analysis alone was not enough to discern the differences in specific metabolites among experimental vs control growth conditions, so we examined within-group differences and compared the starting time point (*T*_0_) and the mid-log phase (*T*_midlog_) of individual culture conditions for all the substrates used (Fig. S4C through E). Mummichog suggested that differentially abundant features between *T*_0_ vs *T*_midlog_ of choline, as well as carnitine (Table 1; Fig. S4D and E) growth conditions were associated with significant differences in glycine, serine, and threonine (GST) metabolism pathway. However, mummichog analysis showed completely different results for glucose growth conditions (Table 1). The top five enriched pathways for glucose growth conditions are folate biosynthesis, phenylalanine, tyrosine, and tryptophan biosynthesis pathway, pantothenate and coenzyme A biosynthesis, glutathione metabolism, and D-amino acid metabolism (Table 1). Other pathways that showed significant differences in choline growth conditions (*T*_0_ vs *T*_midlog_) included valine, leucine, and isoleucine biosynthesis; tyrosine metabolism; and cysteine and methionine metabolism (Table 1). Similarly, other pathways that showed significant differences in carnitine growth conditions (*T*_0_ vs *T*_midlog_) included monobactam biosynthesis, cyanamino acid metabolism, pantothenate and CoA biosynthesis, lysine biosynthesis, and valine, leucine, and isoleucine degradation (Table 1). More details of the top five enriched pathways for glucose, choline, and carnitine growth conditions, along with compound hits, are shown in Table 1. All the scatterplots for the pathways enrichment can be found in the supplementary information (Fig. S4).

**TABLE 1:** The top four enriched metabolic pathways between *T*_0_ and *T*_midlog_ of glucose, choline, and carnitine growth condition using the mummichog algorithm in MetaboAnalystR 6.0

Growth conditions	Pathway name	Compound hits^*a*^	*P*-value (Fisher)	Compound hits (KEGG ID)
	Folate biosynthesis	7/13	0.010191	C11355; C00568; C18239; C20248; C15996; C05924; C21065
Glucose (*T*_0_ vs *T*_midlog_)	D-amino acid metabolism	10/24	0.019348	C00041; C00133; C22611; C00097; C00793; C00077; C02265; C00993; C00624; C00515
	Phenylalanine, tyrosine, and tryptophan biosynthesis	8/17	0.01618	C00296; C01302; C04302; C00826; C00082; C02637; C00108; C00079
	Pantothenate and CoA biosynthesis	7/15	0.02588	C00097; C00429; C00099; C00183; C00049; C00106; C00966
	Glutathione metabolism	5/10	0.043877	C00097; C01879; C00077; C00134; C01672
	Glycine, serine, and threonine metabolism	14/21	0.013085	C00197; C00049; C00188; C00065; C00263; C00740; C00631; C05519; C00719; C03232; C00109; C03508; C00441; C00546
	Valine, leucine, and isoleucine biosynthesis	14/22	0.02303	C02612; C02226; C06032; C00188; C00671; C00123; C06006; C00109; C00407; C00233; C14463; C02631; C04236; C00183
Choline (*T*_0_ vs *T*_midlog_)	Tyrosine metabolism	9/12	0.016481	C05576; C00082; C00232; C03758; C04043; C05577; C00042; C00642; C01161
	Cysteine and methionine metabolism	16/27	0.036217	C02989; C00155; C00065; C00491; C00097; C00793; C00263; C01077; C00049; C11537; C21015; C00109; C03232; C02218; C00441; C00197
	Glycine, serine, and threonine metabolism	5/7	0.0016957	C00049; C00719; C03232; C0358; C00441
	Monobactam biosynthesis	4/5	0.0030087	C00049; C20258; C00441; C0392
Carnitine (*T*_0_ vs *T*_midlog_)	Cyanamino acid metabolism	5/11	0.021869	C00049; C02512; C00152; C08334; C18796
	Pantothenate and CoA biosynthesis	5/11	0.021869	C00429; C00099; C00183; C00049; C02642
	Lysine biosynthesis	4/7	0.016215	C00049; C00441; C20258; C03972
	Valine, leucine, and isoleucine degradation	3/5	0.034023	C00183; C00407; C00123

^*a*^ The mummichog compound hits represent the number of significant compounds divided by the total number of compound hits per pathway.

**Fig 4:**
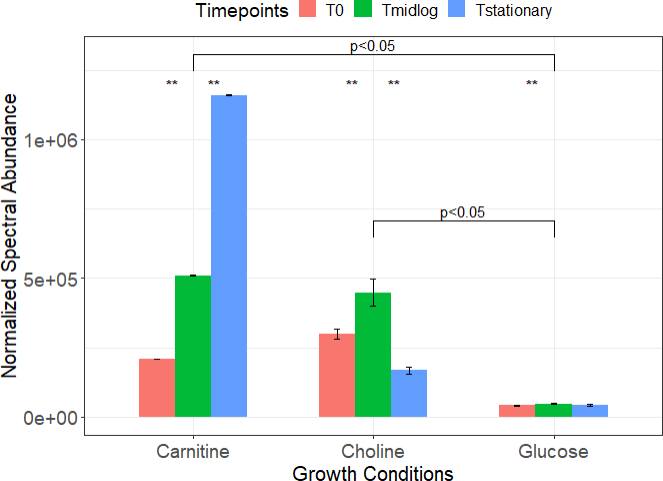
Spectral abundance of extracellular GB at three different time points among three growth conditions (carnitine vs glucose vs glucose). “**” Represents *P*-value < 0.05.

The functional analysis only considered differences within growth conditions; therefore, the metabolomics data needed to be further explored for relative concentrations of individual metabolites to see if there was an actual difference in those metabolic pathways between growth conditions. This was accomplished by comparing the relative spectral abundances of compounds/metabolites annotated from the heatmap and those belonging to the GST pathway in all three growth conditions. We found that GB exhibited significant differences between growth conditions. Normalized spectral abundance was used to compare the relative abundance of GB. The abundance of GB among choline vs glucose growth conditions showed a 9.5-fold increase in GB at the mid-log (Fig. 4). Similarly, the abundance of GB among carnitine vs glucose conditions at mid-log clearly showed differences where GB showed an exponential increase from *T*_0_ to *T*_stationary_ phase of growth in carnitine (Fig. 4). Furthermore, trimethylaminoacetone was also seen in higher abundance in carnitine vs glucose growth conditions. The metabolomics data suggest GB as a potential metabolite with significant change among the growth conditions; we therefore explored a proteomic approach to see if it supports the evidence from the metabolomics data. We further studied the enzymes or homologs of those enzymes known to be involved in the breakdown of choline and carnitine to GB.

### Global proteomic analysis of CJ25 grown on choline, carnitine, or glucose

**Fig 5:**
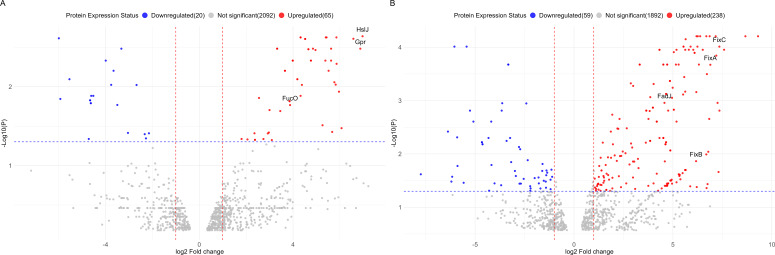
Volcano plots highlighting key upregulated proteins during growth on choline or carnitine. Differentially up- (red) and downregulated (blue) proteins in different growth conditions (**A**) choline vs glucose and (**B**) carnitine vs glucose.

**Fig 6:**
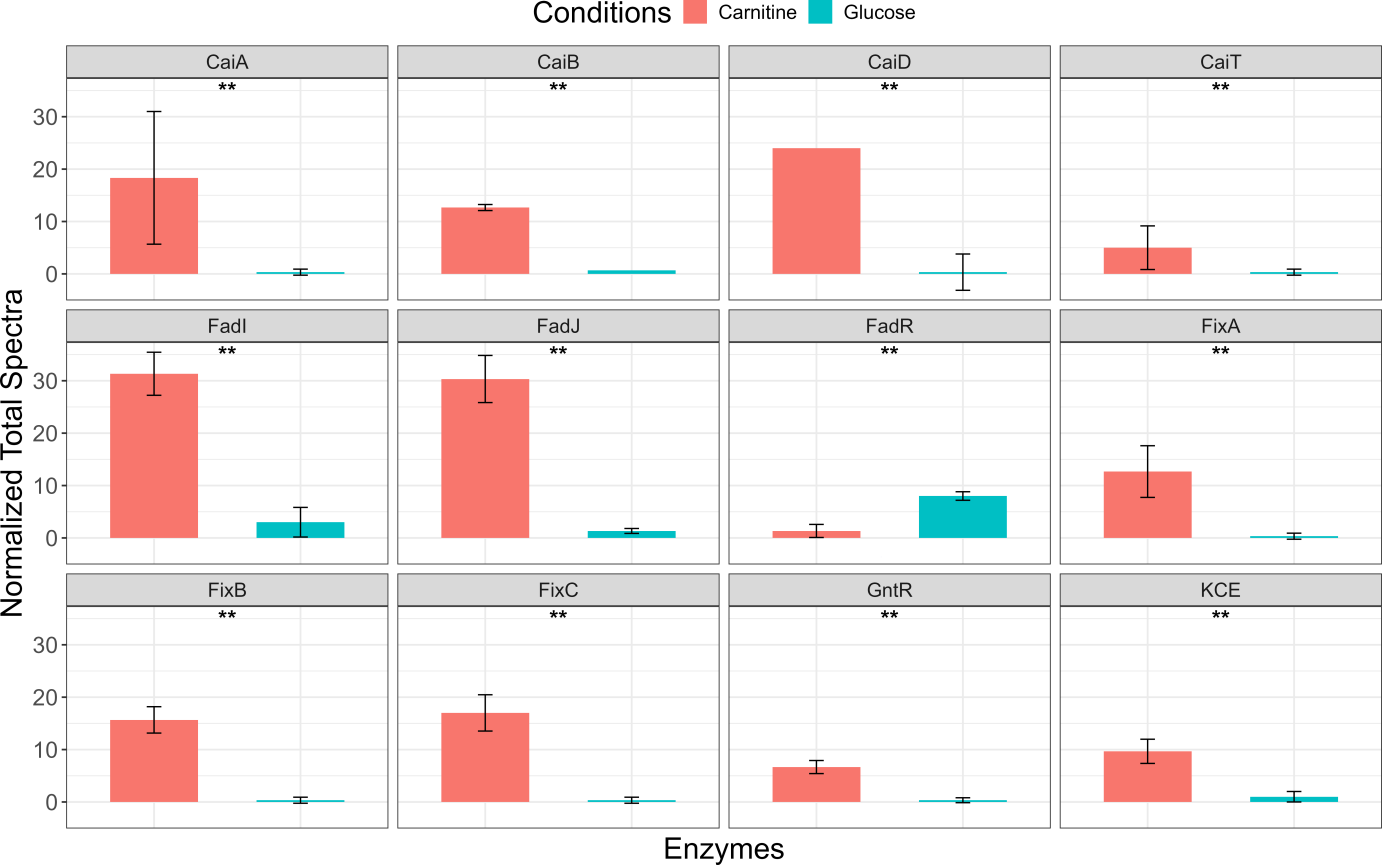
Spectral abundance of specific proteins of interest in carnitine vs glucose growth conditions, putative FadI (Acetyl CoA C acyltransferase), FadJ (3-hydroxyacyl CoA dehydrogenase), KCE (3-keto-5-aminohexanoate cleavage enzyme), CaiA (crotonobetainyl-CoA dehydrogenase), CaiB (L-carnitine CoA transferase), CaiD (crotonobetainyl-CoA hydratase), CaiT (L-carnitine/GBB antiporter), FixABC and putative FadR (GntR family negative regulator for fad regulon), and GntR (DNA-binding transcriptional regulator). “**” Represents *P*-value < 0.05.

Nano-LC-MS/MS proteomic analysis detected and identified a total of 2260 proteins. We found 238 upregulated and 59 downregulated proteins in carnitine vs glucose growth conditions (Fig. 5A). Similarly, 65 proteins were upregulated and 20 downregulated in choline vs glucose growth conditions (Fig. 5B). The list of differentially produced proteins for conditions: choline vs glucose and carnitine vs glucose is provided in the supplementary information (File S2 and 3). Putative annotation of differentially produced protein was performed using protein BLAST against the CJ25 genome in JGI IMG (https://img.jgi.doe.gov/cgi-bin/m/main.cgi). The specific protein identifiers in the supplementary file can be accessed through “UniParc” and “UniRef” databases that cross-reference the UniProtKB. The annotated protein list for carnitine vs glucose growth conditions showed significant upregulation of electron transfer flavoprotein beta subunit (FixA), electron transfer flavoprotein alpha subunit (FixB), electron transfer flavoprotein-quinone oxidoreductase (FixC), crotonobetainyl-CoA dehydrogenase (CaiA), crotonobetainyl CoA- hydratase (CaiB), carnitine CoA- ligase (CaiD), and carnitine-gBB antiporter (CaiT), which are crucial enzymes associated with carnitine metabolism (Fig. 6). However, since the heatmap identified trimethylaminoacetone as a top 10 differential metabolite between the carnitine and glucose growth conditions, suggesting it as a possible intermediate in carnitine degradation, we decided to explore the downstream breakdown of carnitine. The annotated protein list did not identify any enzymes that were previously known to be involved in downstream choline and carnitine breakdown to GB, so we hypothesized that there may be genes in the CJ25 genome that may be encoding enzymes involved in choline and carnitine breakdown in addition to their annotated function. This hypothesis led us to modify our approach to perform protein BLAST searches using previously known enzymes with similar activity against the genome to identify putative homologs in CJ25. A list of enzymes used to BLAST against the CJ25 genome and their putative homologs is listed in Table 2.

**TABLE 2:** The known enzymes used to BLAST against the CJ25 genome and their percentage identity and similarity to putative enzymes

Query	Putative homolog/ Target	E *E*-value	% Identity	% Similarity
Choline dehydrogenase (BetA)	Lactaldehyde reductase (FucO; Ga0439030_01_4762102_4763250)	4.00E-72	36	53
Betaine aldehyde dehydrogenase (BetB)	Succinate semialdehyde dehydrogenase (GabD; Ga0439030_01_700024_701475)	8.00E-116	37	55.2
Carnitine dehydrogenase (LcdH)-PA5386	3-hydroxyacyl CoA dehydrogenase (FadJ; Ga0439030_01_1061566_1063725)	1.00E-17	31	53
Carnitine dehydrogenase-related enzyme (CdhC)	Acetyl CoA-C-acyltransferase (FadI; Ga0439030_01_1060256_1061566)	No significant hits		
Carnitine dehydrogenase-related enzyme (CdhC)	3-keto-5-aminohexanoate cleavage enzyme (KCE; Ga0439030_01_2898547_2899356)	7.00E-24	24	47

**Fig 7:**
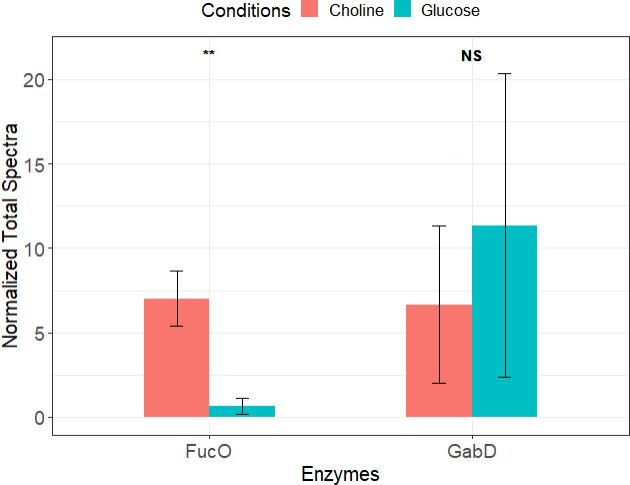
Spectral abundance of putative FucO and GabD in mid-log phase of growth in choline vs glucose conditions.“**” Represents *P*-value < 0.05. FucO-lactaldehyde reductase, GabD-succinate semi-aldehyde dehydrogenase.

We investigated the spectral abundance of identified putative homologs and compared them between growth conditions. Interestingly, homologs for previously known enzymes were significantly abundant under both choline and carnitine growth conditions, compared to glucose. We examined levels of lactaldehyde reductase (FucO) due to its sequence similarity to choline dehydrogenase (BetA), an enzyme known to oxidize choline to betaine aldehyde, and succinate semialdehyde dehydrogenase (GabD; Table 2), due to its sequence similarity to betaine aldehyde dehydrogenases (BetB). BetB is known to oxidize betaine aldehyde to glycine betaine. There was a significant increase in the abundance of lactaldehyde reductase (FucO); however, succinate semialdehyde dehydrogenase (GabD) was abundant but not significantly different between the choline and glucose growth conditions (Fig. 7). Although GabD was non-significant, we considered it based on the results from BLAST using the BLOSUM45 scoring matrix that showed 37% identity to a known betaine aldehyde dehydrogenase (BetB) (Table 2). Furthermore, we examined levels of 3-hydroxy acyl CoA dehydrogenase (FadJ) due to its sequence similarity with carnitine dehydrogenase (LcdH-PA5386), an enzyme known to oxidize carnitine to 3-dehydrocarnitine and acetyl CoA acyltransferase (FadI), but it did not show any significant hits to carnitine dehydrogenase-related enzyme (CdhC). FadI has been known to contribute to anaerobic oxidation of fatty acids in *E. coli* and convert a structurally similar compound, fatty acyl CoA (25), which is similar to 3-dehydrocarnitine. Also, we looked at levels of 3-keto-5-aminohexanoate cleavage enzyme (KCE) due to its sequence similarity with carnitine dehydrogenase-related enzyme (CdhC). KCE has been shown to be involved in the conversion of 3-dehydrocarnitine to betainyl-CoA (26). There was a significant increase in the abundance of 3-hydroxy acyl CoA dehydrogenase (FadJ), acetyl CoA acyltransferase (FadI), and KCE (Fig. 6) in the carnitine growth condition compared to glucose. Both FadI and FadJ were 10 times, and KCE was five times more abundant in carnitine growth conditions, suggesting their possible role in the breakdown of carnitine to GB. We were unable to identify a homolog for betainyl-CoA thiolase in the CJ25 genome, so we hypothesize that 3-hydroxy acyl CoA dehydrogenase (*fadJ*) may be performing a dual function because there is prior evidence of its dual functionality (26). In *Sinorhizobium meliloti*, 3-hydroxy acyl CoA dehydrogenase (*SM2011_c01638*) is shown to have dual functionality, where it was shown to degrade carnitine to 3-dehydrocarnitine and convert betainyl-CoA to GB (26). CJ25 could possibly be using 3-hydroxy acyl CoA dehydrogenase (FadJ) to convert carnitine to 3-dehydrocarnitine and betainyl-CoA to GB.

## DISCUSSION

**Fig 8:**
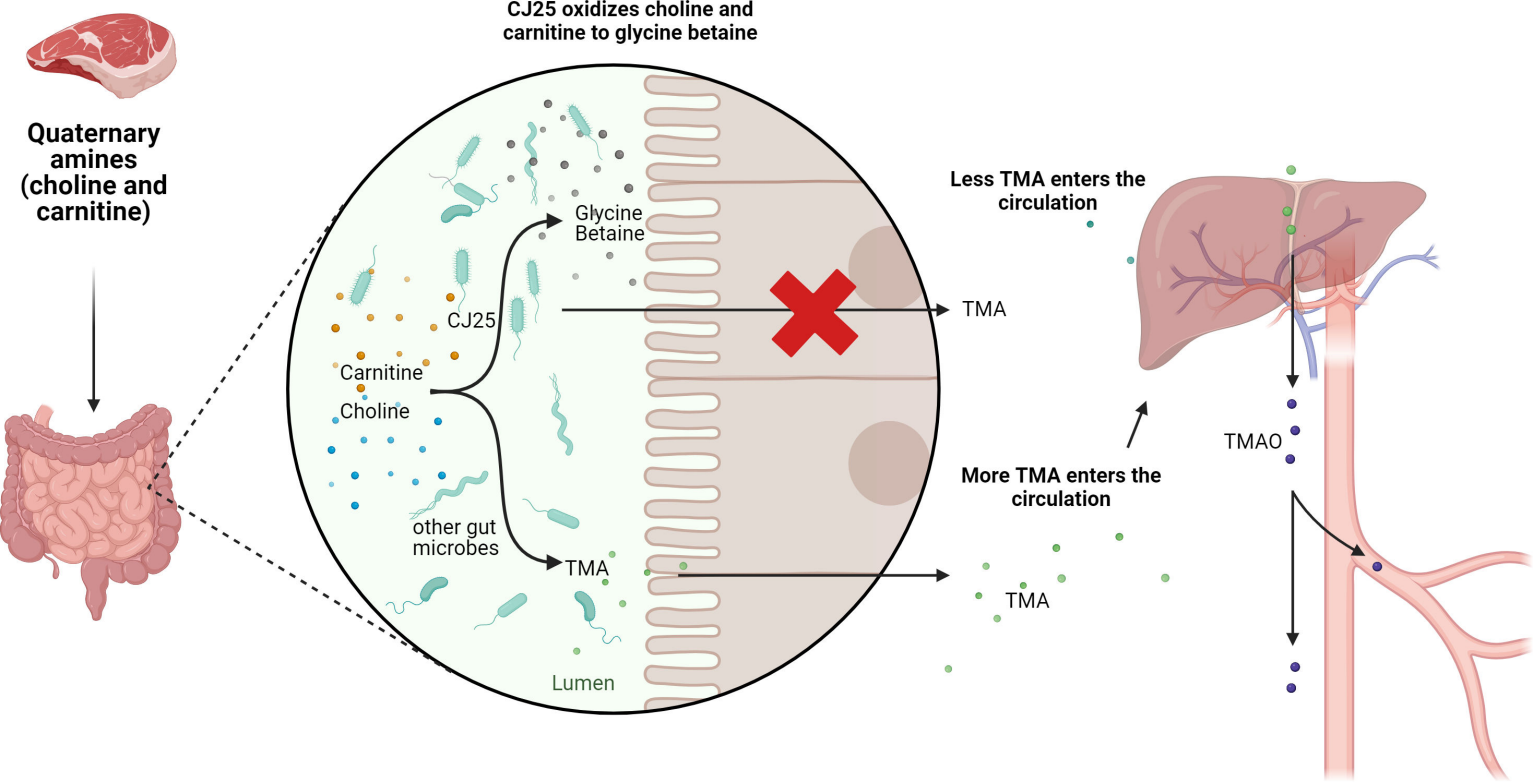
Schematic pathways of proposed hypotheses that reduce TMA production in the human gut, further reducing systemic TMAO in the human body.

We report here on the analysis of the likely non-atherogenic breakdown of the quaternary amines choline and carnitine to glycine betaine by CJ25. The lack of known choline-oxidizing and known downstream carnitine-oxidizing enzymes with trimethylaminoacetone as an intermediate in CJ25’s proteome suggests that these are non-atherogenic pathways involving novel enzymes. The degradation of carnitine to trimethylaminoacetone and further to GB in the cell-free extract of *Pseudomonas* species AK1 has been previously shown (27). Our data suggest that these pathways may be facilitated by several dehydrogenase homologs that are not previously known to degrade quaternary amines. This work reports the process required for the *in vitro* reconstruction of the initial steps of choline and carnitine breakdown by CJ25. Our work is consistent with published work on choline and carnitine breakdown to GB (14, 17), but expands it to include knowledge of novel enzymes involved in the initial breakdown of choline. Given our results from the combined proteomics and metabolomics, we hypothesize a model for the initial steps of choline and carnitine breakdown, which will require further investigation (Fig. 8).

The proteomics data further revealed significant differences in the abundance of enzymes involved in the gluconeogenesis pathway such as phosphoenolpyruvate synthetase (PpsA), fructose 1,6-bisphosphatase (GlpX), and glucose-1-phosphatase (Agp; Fig. S5) under the carnitine and choline grown conditions as compared to glucose. The differential abundance of enzymes in the gluconeogenesis pathway suggests that CJ25 could be breaking down choline and carnitine to pyruvate and generating glucose via gluconeogenesis. Because pyruvate is the gateway to central metabolism, we tracked the tricarboxylic acid cycle and observed a significant increase in the abundance of proteins like isocitrate lyase (AceA), malate synthase (GlcB), fumarate reductase (FrdB), and succinate dehydrogenase (FrdA; Fig. S6). The significant increase in abundance of AceA*,* GlcB*,* and FrdA can help us speculate that CJ25 could be using the glyoxylate cycle to satisfy its cellular carbon requirement when simple sugars are not available in the media. Nicotinamide Adenine Dinucleotide (NADH) is likely produced in the first step of the oxidation of choline and carnitine as well as the glyoxylate cycle; therefore, there should be a way that NADH is being consumed to generate energy, so we tried to further explore how NADH is being used to generate ATP. We saw a significant increase in the abundance of succinate dehydrogenase and fumarate reductase, which is responsible for the interconversion of succinate and fumarate. Therefore, fumarate could potentially be the ultimate electron acceptor in an anaerobic respiratory chain (Fig. S6).

Upon further exploration, proteomics data revealed previously known enzymes associated with carnitine metabolism, such as electron transfer flavoprotein beta subunit (FixA), electron transfer flavoprotein alpha subunit (FixB), electron transfer flavoprotein-quinone oxidoreductase (FixC), crotonobetainyl-CoA dehydrogenase (CaiA), crotonobetainyl CoA-hydratase (CaiB), carnitine CoA-ligase (CaiD), and carnitine-gBB antiporter (CaiT). The significant upregulation of the FixABC operon has been previously predicted to be involved in transferring electrons to carnitine (28–30), which raises an important and interesting question of whether carnitine is the electron donor or acceptor, or both. Also, the differential abundance of CaiABDT suggests the involvement of the gBB-crotonobetaine-carnitine cycle (18) for carnitine metabolism; however, this cycle does not involve the intermediate trimethylaminoacetone, which was seen in our metabolomics data. This led us to explore the downstream oxidation of L-carnitine to 3-dehydrocarnitine and their downstream products, which could be trimethylaminoacetone and subsequently GB. Furthermore, the putative 3-hydroxyacyl CoA dehydrogenase designated as FadJ and putative acetyl CoA acyltransferase designated as FadI, involved in the anaerobic beta-oxidation pathway (25), are abundant in carnitine-grown cells. By inspecting the molecular formulae of substrates involved in anaerobic beta-oxidation, we found some structural similarities to the proposed carnitine breakdown pathway. Also, the increased abundance of DNA-binding GntR family transcriptional regulator (GntR) and decreased abundance of GntR family negative regulator for fad regulon, a positive regulator of *fadA* (possibly FadR; Fig. 6) in carnitine-grown cells, suggests that the Fad regulon involving FadI and FadJ enzymes is involved in the breakdown of L-carnitine like in anaerobic fatty acid breakdown. This led us to a hypothesis that carnitine could be broken down to GB by Fad homologs typically involved in the anaerobic beta-oxidation pathway.

The data from our analysis of combined metabolomic and proteomic profiles produced by CJ25 during growth on the quaternary amines choline and carnitine gave us insights into likely non-atherogenic quaternary amine metabolism by a gut bacterium involving novel enzymes. Using untargeted LC-MS/MS metabolomics, we were able to differentiate the extracellular metabolome of CJ25 during growth on choline and carnitine compared to glucose. Due to the difficulty of identifying compounds using an untargeted approach, we were only able to identify a limited number of compounds from the data. Since the focus of our study was on extracellular metabolites, the major limitation of our study is the exclusion of some metabolic pathways or activities that could have been identified with intracellular metabolites; also, the insights into the intermediate steps of the metabolic pathway could be limited. These limitations may not represent the organism’s full metabolic potential under different growth conditions. However, this present study provides strong evidence for putative enzymes involved in the breakdown of choline and carnitine by CJ25. Our results demonstrated evidence of GB production in the mid-log phase during growth on choline and carnitine. Furthermore, results from our proteomic analysis showed increased abundance of enzymes such as lactaldehyde reductase (FucO) and succinate semialdehyde dehydrogenase (GabD) during growth on choline as well as 3-hydroxyacyl CoA dehydrogenase (FadJ) and acetyl CoA acyltransferase (FadI) during growth on carnitine, suggesting that these enzymes may be involved in catabolism of choline and carnitine to GB. The data also revealed that CJ25 may be using a gluconeogenesis pathway to satisfy cellular carbon needs by participating in the glyoxylate cycle to prevent the loss of carbon biomass. The data from the current study will possibly help us reconstitute the key steps of the pathways of choline and carnitine breakdown by CJ25 and identify novel enzymes for the non-atherogenic breakdown of choline and carnitine in the human gut. The identification of initial pathways and enzymes associated with choline and carnitine breakdown may also exist in other gut microbiota, which could amplify the effects of these pathways significantly, possibly reducing the risk of atherosclerotic cardiovascular disease. Further experimental investigations are required to elucidate the biological consequences of these enzymes and their relevance to choline and carnitine breakdown. Future work, including gene cloning, enzyme purification, and assays, will be crucial to confirm their activity in choline and carnitine breakdown.

## SUPPLEMENTAL MATERIAL

File S1 msphere.00421-25-s0001.xlsx Metabolomics compound annotation.

File S2 msphere.00421-25-s0002.xlsx Proteomics annotations for choline and glucose.

File S3 msphere.00421-25-s0003.xlsx Proteomics annotation for carnitine and glucose.

File S4 msphere.00421-25-s0004.rtf The proteins from CJ25.

Supplemental figures msphere.00421-25-s0005.docx Figures S1 through S6.

Legends msphere.00421-25-s0006.docx Legends for Files S1 through S4.

ASM does not own the copyrights to Supplemental Material that may be linked to, or accessed through, an article. The authors have granted ASM a non-exclusive, world-wide license to publish the Supplemental Material files. Please contact the corresponding author directly for reuse.
