# Supplementary material for: Proteomic and metabolomic analysis reveals new insights into quaternary amine metabolism in Citrobacter amalonaticus CJ25
Source: mSphere. 2025 Aug 25;10(9):e00421-25. doi: 10.1128/msphere.00421-25 (PMC12482172; doi:10.1128/msphere.00421-25)
Supplement: File S4 — The proteins from CJ25. [file msphere.00421-25-s0004.rtf]

>UPI00000000D1 6,7-dimethyl-8-ribityllumazine synthase OS=Citrobacter amalonaticus OX=35703 GN=ribE SS=EMBLWGS:MBE0396890 PC=UP000642697:Unassembled WGS sequenceMNIIEANVATPDARVAITIARFNNFINDSLLEGAIDALKRIGQVKDENITVVWVPGAYELPLAAGALAKTGKYDAVIALGTVIRGGTAHFEYVAGGASNGLAHVAQDSEIPVAFGVLTTESIEQAIERAGTKAGNKGAEAALTALEMINVLKAIKA>UPI0000000AA0 Universal stress protein UspA OS=Citrobacter amalonaticus OX=35703 GN=uspA SS=EMBLWGS:MBE0397850 PC=UP000642697:Unassembled WGS sequenceMAYKHILIAVDLSPESKVLVEKAVSMARPYNAKVSLIHVDVNYSDLYTGLIDVNLGDMQKRISEETHHALTELSTNAGYPITETLSGSGDLGQVLVDAIKKYDMDLVVCGHHQDFWSKLMSSARQLINTVHVDMLIVPLRDEEE>UPI0000000AFA Two-component system response regulator OmpR OS=Citrobacter amalonaticus OX=35703 GN=ompR SS=EMBLWGS:MBE0397753 PC=UP000642697:Unassembled WGS sequenceMQENYKILVVDDDMRLRALLERYLTEQGFQVRSVANAEQMDRLLTRESFHLMVLDLMLPGEDGLSICRRLRSQSNPMPIIMVTAKGEEVDRIVGLEIGADDYIPKPFNPRELLARIRAVLRRQANELPGAPSQEEAVIAFGKFKLNLGTREMFREDEPMPLTSGEFAVLKALVSHPREPLSRDKLMNLARGREYSAMERSIDVQISRLRRMVEEDPAHPRYIQTVWGLGYVFVPDGSKA>UPI0000006216 DNA-binding transcriptional regulator Fis OS=Citrobacter amalonaticus OX=35703 GN=fis SS=EMBLWGS:MBE0397625 PC=UP000642697:Unassembled WGS sequenceMFEQRVNSDVLTVSTVNSQDQVTQKPLRDSVKQALKNYFAQLNGQDVNDLYELVLAEVEQPLLDMVMQYTRGNQTRAALMMGINRGTLRKKLKKYGMN>UPI000003112A Thioredoxin TrxA OS=Citrobacter amalonaticus OX=35703 GN=trxA SS=EMBLWGS:MBE0398139 PC=UP000642697:Unassembled WGS sequenceMSDKIIHLTDDSFDTDVLKADGAILVDFWAEWCGPCKMIAPILDEIADEYQGKLTVAKLNIDQNPGTAPKYGIRGIPTLLLFKNGEVAATKVGALSKGQLKEFLDANLA>UPI000003EAC1 50S ribosomal protein L36 OS=Citrobacter amalonaticus OX=35703 GN=rpmJ SS=EMBLWGS:MBE0397676 PC=UP000642697:Unassembled WGS sequenceMKVRASVKKLCRNCKIVKRDGVIRVICSAEPKHKQRQG>UPI000003EAC5 30S ribosomal protein S5 OS=Citrobacter amalonaticus OX=35703 GN=rpsE SS=EMBLWGS:MBE0397680 PC=UP000642697:Unassembled WGS sequenceMAHIEKQAGELQEKLIAVNRVSKTVKGGRIFSFTALTVVGDGNGRVGFGYGKAREVPAAIQKAMEKARRNMINVALNNGTLQHPVKGVHTGSRVFMQPASEGTGIIAGGAMRAVLEVAGVHNVLAKAYGSTNPINVVRATIDGLENMNSPEMVAAKRGKSVEEILGK>UPI000003EAC8 30S ribosomal protein S8 OS=Citrobacter amalonaticus OX=35703 GN=rpsH SS=EMBLWGS:MBE0397683 PC=UP000642697:Unassembled WGS sequenceMSMQDPIADMLTRIRNGQAANKAAVTMPSSKLKVAIANVLKEEGFIEDFKVEGDTKPELELTLKYFQGKAVVESIQRVSRPGLRIYKRKDELPKVMAGLGIAVVSTSKGVMTDRAARQAGLGGEIICYVA>UPI000003EAE1 30S ribosomal protein S3 OS=Citrobacter amalonaticus OX=35703 GN=rpsC SS=EMBLWGS:MBE0397691 PC=UP000642697:Unassembled WGS sequenceMGQKVHPNGIRLGIVKPWNSTWFANTKEFADNLDSDFKVRQYLTKELAKASVSRIVIERPAKSIRVTIHTARPGIVIGKKGEDVEKLRKVVADIAGVPAQINIAEVRKPELDAKLVADSITSQLERRVMFRRAMKRAVQNAMRLGAKGIKVEVSGRLGGAEIARTEWYREGRVPLHTLRADIDYNTSEAHTTYGVIGVKVWIFKGEILGGMAAVEQPEKPAAQPKKQQRKGRK>UPI000003EAE2 50S ribosomal protein L22 OS=Citrobacter amalonaticus OX=35703 GN=rplV SS=EMBLWGS:MBE0397692 PC=UP000642697:Unassembled WGS sequenceMETIAKHRHARSSAQKVRLVADLIRGKKVSQALDILTYTNKKAAVLVKKVLESAIANAEHNDGADIDDLKVTKIFVDEGPSMKRIMPRAKGRADRILKRTSHITVVVSDR>UPI000003EAE3 30S ribosomal protein S19 OS=Citrobacter amalonaticus OX=35703 GN=rpsS SS=EMBLWGS:MBE0397693 PC=UP000642697:Unassembled WGS sequenceMPRSLKKGPFIDLHLLKKVEKAVESGDKKPLRTWSRRSTIFPNMIGLTIAVHNGRQHVPVFVTDEMVGHKLGEFAPTRTYRGHAADKKAKKK>UPI000003EAE4 50S ribosomal protein L2 OS=Citrobacter amalonaticus OX=35703 GN=rplB SS=EMBLWGS:MBE0397694 PC=UP000642697:Unassembled WGS sequenceMAVVKCKPTSPGRRHVVKVVNPELHKGKPFAPLLEKNSKSGGRNNNGRITTRHIGGGHKQAYRIVDFKRNKDGIPAVVERLEYDPNRSANIALVLYKDGERRYILAPKGLKAGDQIQSGVDAAIKPGNTLPMRNIPVGSTVHNVEMKPGKGGQLARSAGTYVQIVARDGAYVTLRLRSGEMRKVEADCRATLGEVGNAEHMLRVLGKAGAARWRGVRPTVRGTAMNPVDHPHGGGEGRNFGKHPVTPWGVQTKGKKTRSNKRTDKFIVRRRSK>UPI000003EAE6 50S ribosomal protein L4 OS=Citrobacter amalonaticus OX=35703 GN=rplD SS=EMBLWGS:MBE0397696 PC=UP000642697:Unassembled WGS sequenceMELVLKDAQSALTVSETTFGRDFNEALVHQVVVAYAAGARQGTRAQKTRAEVTGSGKKPWRQKGTGRARSGSIKSPIWRSGGVTFAARPQDHSQKVNKKMYRGALKSILSELVRQDRLIVVEKFSVEAPKTKLLAQKLKDMALEDVLIITGELDENLFLAARNLHKVDVRDATGIDPVSLIAFDKVVMTADAVKQVEEMLA>UPI000003EAF7 F0F1 ATP synthase subunit C OS=Citrobacter amalonaticus OX=35703 GN=atpE SS=EMBLWGS:MBE0398098 PC=UP000642697:Unassembled WGS sequenceMENLNMDLLYMAAAVMMGLAAIGAAIGIGILGGKFLEGAARQPDLIPLLRTQFFIVMGLVDAIPMIAVGLGLYVMFAVA>UPI000003EB27 DNA-directed RNA polymerase subunit omega OS=Citrobacter amalonaticus OX=35703 GN=rpoZ SS=EMBLWGS:MBE0397979 PC=UP000642697:Unassembled WGS sequenceMARVTVQDAVEKIGNRFDLVLVAARRARQMQVGGKDPLVPEENDKTTVIALREIEEGLINNQILDVRERQEQQEQEAAELQAVTAIAEGRR>UPI000003EB6C DNA-directed RNA polymerase subunit alpha OS=Citrobacter amalonaticus OX=35703 GN=rpoA SS=EMBLWGS:MBE0397672 PC=UP000642697:Unassembled WGS sequenceMQGSVTEFLKPRLVDIEQVSSTHAKVTLEPLERGFGHTLGNALRRILLSSMPGCAVTEVEIDGVLHEYSTKEGVQEDILEILLNLKGLAVRVQGKDEVILTLNKSGIGPVTAADITHDGDVEIVKPQHVICHLTDENASISMRIKVQRGRGYVPASTRIHSEEDERPIGRLLVDACYSPVERIAYNVEAARVEQRTDLDKLVIEMETNGTIDPEEAIRRAATILAEQLEAFVDLRDVRQPEVKEEKPEFDPILLRPVDDLELTVRSANCLKAEAIHYIGDLVQRTEVELLKTPNLGKKSLTEIKDVLASRGLSLGMRLENWPPASIADE>UPI000003EB6E 30S ribosomal protein S11 OS=Citrobacter amalonaticus OX=35703 GN=rpsK SS=EMBLWGS:MBE0397674 PC=UP000642697:Unassembled WGS sequenceMAKAPIRARKRVRKQVSDGVAHIHASFNNTIVTITDRQGNALGWATAGGSGFRGSRKSTPFAAQVAAERCADAVKEYGIKNLEVMVKGPGPGRESTIRALNAAGFRITNITDVTPIPHNGCRPPKKRRV>UPI000003FF29 RNA chaperone/antiterminator CspA OS=Citrobacter amalonaticus OX=35703 GN=cspA SS=EMBLWGS:MBE0397901 PC=UP000642697:Unassembled WGS sequenceMSGKMTGIVKWFNADKGFGFITPDDGSKDVFVHFSAIQNDGYKSLDEGQKVSFTIESGAKGPAAGNVTSL>UPI0000047C5B 30S ribosomal protein S12 OS=Citrobacter amalonaticus OX=35703 GN=rpsL SS=EMBLWGS:MBE0397705 PC=UP000642697:Unassembled WGS sequenceMATVNQLVRKPRARKVAKSNVPALEACPQKRGVCTRVYTTTPKKPNSALRKVCRVRLTNGFEVTSYIGGEGHNLQEHSVILIRGGRVKDLPGVRYHTVRGALDCSGVKDRKQARSKYGVKRPKA>UPI0000047CA9 Carbon storage regulator CsrA OS=Citrobacter amalonaticus OX=35703 GN=csrA SS=EMBLWGS:MBE0394514 PC=UP000642697:Unassembled WGS sequenceMLILTRRVGETLMIGDEVTVTVLGVKGNQVRIGVNAPKEVSVHREEIYQRIQAEKSQQSSY>UPI0000047CBB 50S ribosomal protein L20 OS=Citrobacter amalonaticus OX=35703 GN=rplT SS=EMBLWGS:MBE0396083 PC=UP000642697:Unassembled WGS sequenceMARVKRGVIARARHKKILKQAKGYYGARSRVYRVAFQAVIKAGQYAYRDRRQRKRQFRQLWIARINAAARQNGISYSKFINGLKKASVEIDRKILADIAVFDKVAFTALVEKAKAALA>UPI00000502F3 Protein YpfM OS=Citrobacter amalonaticus OX=35703 GN=ypfM SS=EMBLWGS:MBE0394700 PC=UP000642697:Unassembled WGS sequenceMIERELGNWKDFIEVMLRK>UPI00000502F7 Protein MgtS OS=Citrobacter amalonaticus OX=35703 GN=mgtS SS=EMBLWGS:MBE0395938 PC=UP000642697:Unassembled WGS sequenceMLGNMNVFMAVLGIILFSGFLAAYFSHKWDD>UPI0000059A65 RNA polymerase sigma factor RpoE OS=Citrobacter amalonaticus OX=35703 GN=rpoE SS=EMBLWGS:MBE0394603 PC=UP000642697:Unassembled WGS sequenceMSEQLTDQVLVERVQKGDQKAFNLLVVRYQHKVASLVSRYVPSGDVPDVVQESFIKAYRALDSFRGDSAFYTWLYRIAVNTAKNYLVAQGRRPPSSDVDAIEAENFESGGALKEISNPENLMLSEELRQIVFRTIESLPEDLRMAITLRELDGLSYEEIAAIMDCPVGTVRSRIFRAREAIDNKVQPLIRR>UPI0000059AF8 PTS glucose transporter subunit IIA OS=Citrobacter amalonaticus OX=35703 GN=crr SS=EMBLWGS:MBE0394744 PC=UP000642697:Unassembled WGS sequenceMGLFDKLKSLVSDDKKDTGTIEIVAPLSGEIVNIEDVPDVVFAEKIVGDGIAIKPTGNKMVAPVDGTIGKIFETNHAFSIESDSGIELFVHFGIDTVELKGEGFKRIAEEGQRVKVGDPVIEFDLPLLEEKAKSTLTPVVISNMDEIKELIKLSGSVTVGETPVIRIKK>UPI0000059AFA Phosphocarrier protein Hpr OS=Citrobacter amalonaticus OX=35703 GN=ptsH SS=EMBLWGS:MBE0394746 PC=UP000642697:Unassembled WGS sequenceMFQQEVTITAPNGLHTRPAAQFVKEAKGFTSEITVTSNGKSASAKSLFKLQTLGLTQGTVVTISAEGEDEQKAVEHLVKLMAELE>UPI0000059CB5 Sulfurtransferase-like selenium metabolism protein YedF OS=Citrobacter amalonaticus OX=35703 GN=yedF SS=EMBLWGS:MBE0395462 PC=UP000642697:Unassembled WGS sequenceMKNIVPDYRLDMVGEPCPYPAVATLEAMPQLKKGEILEVVSDCPQSINNIPLDARNHGYTVLDIQQDGPTIRYLIQK>UPI0000059CF2 Crossover junction endodeoxyribonuclease RuvC OS=Citrobacter amalonaticus OX=35703 GN=ruvC SS=EMBLWGS:MBE0395537 PC=UP000642697:Unassembled WGS sequenceMSIILGIDPGSRITGYGVIRQVGRQLTYLGSGCIRTKVDDLPSRLKLIYAGVTEIITQFQPDYFAIEQVFMAKNADSALKLGQARGVAIVAAVNQELPVFEYAARQVKQTVVGIGSAEKSQVQHMVRTLLKLPANPQADAADALAIAITHCHVSQNAMQMSESRLNLARGRLR>UPI0000059D29 Transcription antiterminator/RNA stability regulator CspE OS=Citrobacter amalonaticus OX=35703 GN=cspE SS=EMBLWGS:MBE0395575 PC=UP000642697:Unassembled WGS sequenceMAKIKGQVKWFNESKGFGFITPADGSKDVFVHFSAIQGNGFKTLAEGQNVEFEIQDGQKGPAAVNVTAI>UPI0000059D5D Ribose-phosphate diphosphokinase OS=Citrobacter amalonaticus OX=35703 GN=prs SS=EMBLWGS:MBE0395655 PC=UP000642697:Unassembled WGS sequenceMPDMKLFAGNATPELAQRIANRLYTSLGDAAVGRFSDGEVSVQINENVRGGDIFIIQSTCAPTNDNLMELVVMVDALRRASAGRITAVIPYFGYARQDRRVRSARVPITAKVVADFLSSVGVDRVLTVDLHAEQIQGFFDVPVDNVFGSPILLEDMLQLNLDNPIVVSPDIGGVVRARAIAKLLNDTDMAIIDKRRPRANVSQVMHIIGDVAGRDCVLVDDMIDTGGTLCKAAEALKERGAKRVFAYATHPIFSGNAANNLRNSVIDEVVVCDTIPLTDEIKALPNVRTLTLSGMLAEAIRRISNEESISAMFEH>UPI0000059DC9 PheST operon leader peptide PheM OS=Citrobacter amalonaticus OX=35703 GN=pheM SS=EMBLWGS:MBE0396082 PC=UP000642697:Unassembled WGS sequenceMNAAIFRFFFYFST>UPI0000059DE4 Murein lipoprotein Lpp OS=Citrobacter amalonaticus OX=35703 GN=lpp SS=EMBLWGS:MBE0396045 PC=UP000642697:Unassembled WGS sequenceMNRTKLVLGAVILGSTLLAGCSSNAKIDQLSSDVQTLNAKVDQLSNDVNAMRSDVQAAKDDAARANQRLDNQATKYRK>UPI0000059F9B Acyl carrier protein OS=Citrobacter amalonaticus OX=35703 GN=acpP SS=EMBLWGS:MBE0396206 PC=UP000642697:Unassembled WGS sequenceMSTIEERVKKIIGEQLGVKQEEVTNNASFVEDLGADSLDTVELVMALEEEFDTEIPDEEAEKITTVQAAIDYINGHQA>UPI0000059FA0 50S ribosomal protein L32 OS=Citrobacter amalonaticus OX=35703 GN=rpmF SS=EMBLWGS:MBE0396211 PC=UP000642697:Unassembled WGS sequenceMAVQQNKPTRSKRGMRRSHDALTAVTSLSVDKTSGEKHLRHHITADGYYRGRKVIAK>UPI000005A08B Translation initiation factor IF-1 OS=Citrobacter amalonaticus OX=35703 GN=infA SS=EMBLWGS:MBE0396409 PC=UP000642697:Unassembled WGS sequenceMAKEDNIEMQGTVLETLPNTMFRVELENGHVVTAHISGKMRKNYIRILTGDKVTVELTPYDLSKGRIVFRSR>UPI000005A176 Transcription antiterminator/RNA stability regulator CspE OS=Citrobacter amalonaticus OX=35703 GN=cspE SS=EMBLWGS:MBE0396702 PC=UP000642697:Unassembled WGS sequenceMSKIKGNVKWFNESKGFGFITPEDGSKDVFVHFSAIQTNGFKTLAEGQRVEFEITNGAKGPSAANVTAL>UPI000005A1FD Hemolysin expression modulator Hha OS=Citrobacter amalonaticus OX=35703 GN=hha SS=EMBLWGS:MBE0396837 PC=UP000642697:Unassembled WGS sequenceMSDKPLTKTDYLMRLRRCQTIDTLERVIEKNKYELSDNELAVFYSAADHRLAELTMNKLYDKIPSSVWKFIR>UPI000005A4AC 30S ribosomal protein S21 OS=Citrobacter amalonaticus OX=35703 GN=rpsU SS=EMBLWGS:MBE0397441 PC=UP000642697:Unassembled WGS sequenceMPVIKVRENEPFDVALRRFKRSCEKAGVLAEVRRREFYEKPTTERKRAKASAVKRHAKKLARENARRTRLY>UPI000005A4EF 30S ribosomal protein S15 OS=Citrobacter amalonaticus OX=35703 GN=rpsO SS=EMBLWGS:MBE0397535 PC=UP000642697:Unassembled WGS sequenceMSLSTEATAKIVSEFGRDANDTGSTDVQVALLTAQINHLQGHFAEHKKDHHSRRGLLRMVSQRRKLLDYLKRKDVARYTALIERLGLRR>UPI000005A541 Rod shape-determining protein MreB OS=Citrobacter amalonaticus OX=35703 GN=mreB SS=EMBLWGS:MBE0397611 PC=UP000642697:Unassembled WGS sequenceMLKKFRGMFSNDLSIDLGTANTLIYVKGQGIVLNEPSVVAIRQDRAGSPKSVAAVGHDAKQMLGRTPGNIAAIRPMKDGVIADFFVTEKMLQHFIKQVHSNSFMRPSPRVLVCVPVGATQVERRAIRESAQGAGAREVFLIEEPMAAAIGAGLPVSEATGSMVVDIGGGTTEVAVISLNGVVYSSSVRIGGDRFDEAIINYVRRNYGSLIGEATAERIKHEIGSAYPGDEVREIEVRGRNLAEGVPRGFTLNSNEILEALQEPLTGIVSAVMVALEQCPPELASDISERGMVLTGGGALLRNLDRLLMEETGIPVVVAEDPLTCVARGGGKALEMIDMHGGDLFSEE>UPI000005A5A1 Ilv operon leader peptide OS=Citrobacter amalonaticus OX=35703 GN=ilvL SS=EMBLWGS:MBE0398119 PC=UP000642697:Unassembled WGS sequenceMTALLRVISLVVISVVVIIIPPCGAALGRGKA>UPI000005A5D2 DNA-binding protein HU-alpha OS=Citrobacter amalonaticus OX=35703 GN=hupA SS=EMBLWGS:MBE0398401 PC=UP000642697:Unassembled WGS sequenceMNKTQLIDVIADKAELSKTQAKAALESTLAAITESLKEGDAVQLVGFGTFKVNHRAERTGRNPQTGKEIKIAAANVPAFVSGKALKDAVK>UPI000005A697 Ribonuclease P protein component OS=Citrobacter amalonaticus OX=35703 GN=rnpA SS=EMBLWGS:MBE0398074 PC=UP000642697:Unassembled WGS sequenceMVKLAFPRELRLLTPAHFTFVFQQPQRAGTPQITILGRLNSLGHPRIGLTVAKKNVRRAHERNRIKRLTRESFRLRQHELPAMDFVVVAKKGVADLDNRALSEALEKLWRRHCRLARGS>UPI000005A698 50S ribosomal protein L34 OS=Citrobacter amalonaticus OX=35703 GN=rpmH SS=EMBLWGS:MBE0398073 PC=UP000642697:Unassembled WGS sequenceMKRTFQPSVLKRNRSHGFRARMATKNGRQVLARRRAKGRARLTVSK>UPI000005A7ED 30S ribosomal protein S10 OS=Citrobacter amalonaticus OX=35703 GN=rpsJ SS=EMBLWGS:MBE0397698 PC=UP000642697:Unassembled WGS sequenceMQNQRIRIRLKAFDHRLIDQSTAEIVETAKRTGAQVRGPIPLPTRKERFTVLISPHVNKDARDQYEIRTHKRLVDIVEPTEKTVDALMRLDLAAGVDVQISLG>UPI000005A7EF 50S ribosomal protein L23 OS=Citrobacter amalonaticus OX=35703 GN=rplW SS=EMBLWGS:MBE0397695 PC=UP000642697:Unassembled WGS sequenceMIREERLLKVLRAPHVSEKASTAMEKTNTIVLKVAKDATKAEIKAAVQKLFEVEVEVVNTLVVKGKVKRHGQRIGRRSDWKKAYVTLKEGQNLDFVGGAE>UPI000005A7F2 50S ribosomal protein L16 OS=Citrobacter amalonaticus OX=35703 GN=rplP SS=EMBLWGS:MBE0397690 PC=UP000642697:Unassembled WGS sequenceMLQPKRTKFRKMHKGRNRGLAAGADVSFGSFGLKAVGRGRLTARQIEAARRAMTRAVKRQGKIWIRVFPDKPITEKPLAVRMGKGKGNVEYWVALIQPGKVLYEMDGVPEELAREAFKLAAAKLPIKTTFVTKTVM>UPI000005A7F3 50S ribosomal protein L29 OS=Citrobacter amalonaticus OX=35703 GN=rpmC SS=EMBLWGS:MBE0397689 PC=UP000642697:Unassembled WGS sequenceMKAKELREKSVEELNTELLNLLREQFNLRMQAASGQLQQSHLLKQVRRDVARVKTLLTEKAGA>UPI000005A7F5 50S ribosomal protein L14 OS=Citrobacter amalonaticus OX=35703 GN=rplN SS=EMBLWGS:MBE0397687 PC=UP000642697:Unassembled WGS sequenceMIQEQTMLNVADNSGARRVMCIKVLGGSHRRYAGVGDIIKITIKEAIPRGKVKKGDVLKAVVVRTKKGVRRPDGSVIRFDGNACVILNNNSEQPIGTRIFGPVTRELRNEKFMKIISLAPEVL>UPI000005A7F7 50S ribosomal protein L5 OS=Citrobacter amalonaticus OX=35703 GN=rplE SS=EMBLWGS:MBE0397685 PC=UP000642697:Unassembled WGS sequenceMAKLHDYYKDEVVNKLMTEFNYNSVMQVPRVEKITLNMGVGEAIADKKLLDNAAADLTAISGQKPLITKARKSVAGFKIRQGYPIGCKVTLRGERMWEFFERLITIAVPRIRDFRGLSAKSFDGRGNYSMGVREQIIFPEIDYDKVDRVRGLDITITTTAKSDEEGRALLAAFDFPFRK>UPI000005A7FA 50S ribosomal protein L18 OS=Citrobacter amalonaticus OX=35703 GN=rplR SS=EMBLWGS:MBE0397681 PC=UP000642697:Unassembled WGS sequenceMDKKSARIRRATRARRKLKELGATRLVVHRTPRHIYAQVIAPNGSEVLVAASTVEKAIAEQLKYTGNKDAAAAVGKAVAERALEKGIKDVSFDRSGFQYHGRVQALADAAREAGLQF>UPI000005A7FB 50S ribosomal protein L30 OS=Citrobacter amalonaticus OX=35703 GN=rpmD SS=EMBLWGS:MBE0397679 PC=UP000642697:Unassembled WGS sequenceMAKTIKITQTRSAIGRLPKHKATLLGLGLRRIGHTVEREDTPAVRGMVNAVSFMVKVEE>UPI000005A7FC 50S ribosomal protein L15 OS=Citrobacter amalonaticus OX=35703 GN=rplO SS=EMBLWGS:MBE0397678 PC=UP000642697:Unassembled WGS sequenceMRLNTLSPAEGSKKAGKRLGRGIGSGLGKTGGRGHKGQKSRSGGGVRRGFEGGQMPLYRRLPKFGFTSRKAAITAEVRLSDLAKVEGGVVDLNTLKAANIIGIQIEFAKVILAGEVTTPVTVRGLRVTKGARAAIEAAGGKIEE>UPI000005A910 Co-chaperone GroES OS=Citrobacter amalonaticus OX=35703 GN=ILP74_00620 SS=EMBLWGS:MBE0394028 PC=UP000642697:Unassembled WGS sequenceMSIRPLHDRVIVKRKEVESKSAGGIVLTGSAAGKSTRGEIIAVGKGRILDNGTVQPLDVKVGDIVIFNDGYGVKSEKIDNEEVLIMSESDILAIVEA>UPI000005A94A 30S ribosomal protein S18 OS=Citrobacter amalonaticus OX=35703 GN=rpsR SS=EMBLWGS:MBE0394073 PC=UP000642697:Unassembled WGS sequenceMARYFRRRKFCRFTAEGVQEIDYKDIATLKNYITESGKIVPSRITGTRAKYQRQLARAIKRARYLSLLPYTDRHQ>UPI000005A9F8 Two-component system response regulator ArcA OS=Citrobacter amalonaticus OX=35703 GN=arcA SS=EMBLWGS:MBE0397222 PC=UP000642697:Unassembled WGS sequenceMQTPHILIVEDELVTRNTLKSIFEAEGYDVFEATDGAEMHQILSEYDINLVIMDINLPGKNGLLLARELREQANVALMFLTGRDNEVDKILGLEIGADDYITKPFNPRELTIRARNLLSRTMNLGTVSEERRSVESYKFNGWELDINSRSLIGPDGEQYKLPRSEFRAMLHFCENPGKIQSRAELLKKMTGRELKPHDRTVDVTIRRIRKHFESTPDTPEIIATIHGEGYRFCGDLQD>UPI0000061470 IS6-like element IS26 family transposase OS=Citrobacter amalonaticus OX=35703 GN=ILP74_24585|ILP74_00060|ILP74_01335|ILP74_00980|ILP74_24400 SS=EMBLWGS:MBE0398484|EMBLWGS:MBE0393925|EMBLWGS:MBE0394165|EMBLWGS:MBE0394097|EMBLWGS:MBE0398465 PC=UP000642697:Unassembled WGS sequenceMNPFKGRHFQRDIILWAVRWYCKYGISYRELQEMLAERGVNVDHSTIYRWVQRYAPEMEKRLRWYWRNPSDLCPWHMDETYVKVNGRWAYLYRAVDSRGRTVDFYLSSRRNSKAAYRFLGKILNNVKKWQIPRFINTDKAPAYGRALALLKREGRCPSDVEHRQIKYRNNVIECDHGKLKRIINATLGFKSMKTAYATIKGIEVMRALRKGQASAFYYGDPLGEMRLVSRVFEM>UPI00000CD52B Envelope stress response regulator transcription factor CpxR OS=Citrobacter amalonaticus OX=35703 GN=cpxR SS=EMBLWGS:MBE0398268 PC=UP000642697:Unassembled WGS sequenceMNKILLVDDDRELTSLLKELLEMEGFNVLVAHDGEQALELLDDSIDLLLLDVMMPKKNGIDTLKALRQTHQTPVIMLTARGSELDRVLGLELGADDYLPKPFNDRELVARIRAILRRSHWSEQQQSSDNGSPTLEVDALSLNPGRQEASFDGQTLELTGTEFTLLYLLAQHLGQVVSREHLSQEVLGKRLTPFDRAIDMHISNLRRKLPERKDGHPWFKTLRGRGYLMVSAS>UPI00000D2AEC PheA operon leader peptide PheL OS=Citrobacter amalonaticus OX=35703 GN=pheL SS=EMBLWGS:MBE0394585 PC=UP000642697:Unassembled WGS sequenceMKHTPFFFAFFFTFP>UPI0000111D8F Integration host factor subunit alpha OS=Citrobacter amalonaticus OX=35703 GN=ihfA SS=EMBLWGS:MBE0396079 PC=UP000642697:Unassembled WGS sequenceMALTKAEMSEYLFDKLGLSKRDAKELVELFFEEIRRALENGEQVKLSGFGNFDLRDKNQRPGRNPKTGEDIPITARRVVTFRPGQKLKSRVENASPKDE>UPI000012847F CAMP-activated global transcriptional regulator CRP OS=Citrobacter amalonaticus OX=35703 GN=crp SS=EMBLWGS:MBE0397721 PC=UP000642697:Unassembled WGS sequenceMVLGKPQTDPTLEWFLSHCHIHKYPSKSTLIHQGEKAETLYYIVKGSVAVLIKDEEGKEMILSYLNQGDFIGELGLFEEGQERSAWVRAKTACEVAEISYKKFRQLIQVNPDILMRLSSQMARRLQVTSEKVGNLAFLDVTGRIAQTLLNLAKQPDAMTHPDGMQIKITRQEIGQIVGCSRETVGRILKMLEDQNLISAHGKTIVVYGTR>UPI000012E854 His operon leader peptide OS=Citrobacter amalonaticus OX=35703 GN=hisL SS=EMBLWGS:MBE0395255 PC=UP000642697:Unassembled WGS sequenceMTRVQFKHHHHHHHPD>UPI000012E897 Thr operon leader peptide OS=Citrobacter amalonaticus OX=35703 GN=thrL SS=EMBLWGS:MBE0397219 PC=UP000642697:Unassembled WGS sequenceMKRISTTITTTITITTGNGAG>UPI0000130A0D NADH-quinone oxidoreductase subunit NuoK OS=Citrobacter amalonaticus OX=35703 GN=nuoK SS=EMBLWGS:MBE0394941 PC=UP000642697:Unassembled WGS sequenceMIPLQHGLILAAILFVLGLTGLVIRRNLLFMLIGLEIMINASALAFVVAGSYWGQTDGQVMYILAISLAAAEASIGLALLLQLHRRRQNLNIDSVSEMRG>UPI000013142B Copper resistance system metallochaperone PcoC OS=Citrobacter amalonaticus OX=35703 GN=pcoC SS=EMBLWGS:MBE0397309 PC=UP000642697:Unassembled WGS sequenceMSILNKAILTGGLVMGVAFSAMAHPELKSSVPQADSAVAAPEKIQLNFSENLTVKFSGAKLTMTGMKGMSSHSPMPVAAKVAPGADPKSMVIIPREPLPAGTYRVDWRAVSSDTHPITGNYTFTVK>UPI000013142E Copper response regulator transcription factor PcoR OS=Citrobacter amalonaticus OX=35703 GN=pcoR SS=EMBLWGS:MBE0397307 PC=UP000642697:Unassembled WGS sequenceMQRILIVEDEQKTGRYLQQGLVEEGYQADLFNNGRDGLGAASKGQYDLIILDVMLPFLDGWQIISALRESGHEEPVLFLTAKDNVRDKVKGLELGADDYLIKPFDFTELVARVRTLLRRARSQAATVCTIADMTVDMVRRTVIRSGKKIHLTGKEYVLLELLLQRTGEVLPRSLISSLVWNMNFDSDTNVIDVAVRRLRSKIDDDFEPKLIHTVRGAGYVLEIREE>UPI000013347F Transcriptional regulator RcsB OS=Citrobacter amalonaticus OX=35703 GN=rcsB SS=EMBLWGS:MBE0394995 PC=UP000642697:Unassembled WGS sequenceMNNMNVIIADDHPIVLFGIRKSLEQIEWVNVVGEFEDSTALINNLPKLDAHVLITDLSMPGDKYGDGITLIKYIKRHFPSLSIIVLTMNNNPAILSAVLDLDIEGIVLKQGAPTDLPKALAALQKGKKFTPESVSRLLEKISAGGYGDKRLSPKESEVLRLFAEGFLVTEIAKKLNRSIKTISSQKKSAMMKLGVENDIALLNYLSSVTLSPADKD>UPI0000133664 Type II toxin-antitoxin system antitoxin RelB OS=Citrobacter amalonaticus OX=35703 GN=relB SS=EMBLWGS:MBE0394295 PC=UP000642697:Unassembled WGS sequenceMGSINLRIDDELKARSYAALEKMGVTPSEALRLMLEYIADNERLPFKQTLLSDEDAELVEIVKERLRNPKPVRVTLDEL>UPI0000133667 Type II toxin-antitoxin system mRNA interferase RelE OS=Citrobacter amalonaticus OX=35703 GN=relE SS=EMBLWGS:MBE0394296 PC=UP000642697:Unassembled WGS sequenceMAYFLDFDERALKEWRKLGSTVREQLKKKLVEVLESPRIEANKLRGMPDCYKIKLRSSGYRLVYQVIDEKVVVFVISVGKRERSEVYSEAVKRIL>UPI000013B05A Lipoprotein YgdR OS=Citrobacter amalonaticus OX=35703 GN=ygdR SS=EMBLWGS:MBE0394392 PC=UP000642697:Unassembled WGS sequenceMKKWAVIISAVGLAFAVSGCSSDYVMATKDGRMILTDGKPEIDDDTGLVSYHDQQGNAMQINRDDVSQIIER>UPI000013B1FE Uncharacterized protein OS=Citrobacter amalonaticus OX=35703 GN=ILP74_20045 SS=EMBLWGS:MBE0397652 PC=UP000642697:Unassembled WGS sequenceMKRLIPVALLTALLAGCAHDSPCVPVYDDQGRLVHTNTCMKGTTQDNWETAGAIAGGAAAVAGLTMGIIALSK>UPI000016225C Leucine-responsive transcriptional regulator Lrp OS=Citrobacter amalonaticus OX=35703 GN=lrp SS=EMBLWGS:MBE0396404 PC=UP000642697:Unassembled WGS sequenceMVDSKKRPGKDLDRIDRNILNELQKDGRISNVELSKRVGLSPTPCLERVRRLERQGFIQGYTALLNPHYLDASLLVFVEITLNRGAPDVFEQFNAAVQKLEEIQECHLVSGDFDYLLKTRVPDMSAYRKLLGETLLRLPGVNDTRTYVVMEEVKQSNRLVIKTR>UPI0000162295 30S ribosomal protein S4 OS=Citrobacter amalonaticus OX=35703 GN=rpsD SS=EMBLWGS:MBE0397673 PC=UP000642697:Unassembled WGS sequenceMARYLGPKLKLSRREGTDLFLKSGVRAIDTKCKIEQAPGQHGARKPRLSDYGVQLREKQKVRRIYGVLERQFRNYYKEAARLKGNTGENLLALLEGRLDNVVYRMGFGATRAEARQLVSHKAIMVNGRVVNIASYQVSPNDVVSIREKAKKQSRVKAALELAEQREKPTWLEVDAGKMEGTYKRKPERSDLSADINEHLIVELYSK>UPI00001654FE 30S ribosomal protein S9 OS=Citrobacter amalonaticus OX=35703 GN=rpsI SS=EMBLWGS:MBE0397591 PC=UP000642697:Unassembled WGS sequenceMAENQYYGTGRRKSSAARVFIKPGNGKIVINQRSLEQYFGRETARMVVRQPLELVDMVEKLDLYITVKGGGISGQAGAIRHGITRALMEYDESLRSELRKAGFVTRDARQVERKKVGLRKARRRPQFSKR>UPI0000165537 Peptidylprolyl isomerase PpiC OS=Citrobacter amalonaticus OX=35703 GN=ppiC SS=EMBLWGS:MBE0398133 PC=UP000642697:Unassembled WGS sequenceMAKTAAALHILVKEEKLALDLLEQIKNGADFGKLAKKHSICPSGKRGGDLGEFRQGQMVPAFDKVVFSCPVLEPTGPLHTQFGYHIIKVLYRN>UPI00001704D6 Tricarballylate/proton symporter TcuC OS=Citrobacter amalonaticus OX=35703 GN=tcuC SS=EMBLWGS:MBE0396663 PC=UP000642697:Unassembled WGS sequenceMTQQPSRAGTFGAILRVTSGNFLEQFDFFLFGFYATYIAKTFFPAESEFAALMLTFAVFGSGFLMRPIGAVVLGAYIDRIGRRKGLMVTLAIMGCGTLLIALVPGYQTIGLLAPVLVLVGRLLQGFSAGVELGGVSVYLSEIATPGNKGFYTSWQSASQQVAIVVAALIGYGLNVTLGHDEISEWGWRIPFFIGCMIIPLIFVLRRSLQETEAFLQRKHRPDTREIFTTIAKNWRIITAGTLLVAMTTTTFYFITVYTPTYGRTVLNLSARDSLVVTMLVGISNFIWLPIGGAISDRIGRRPVLMGITLLALVTTWPVMNWLTAAPDFTRMTLVLLWFSFFFGMYNGAMVAALTEVMPVYVRTVGFSLAFSLATAIFGGLTPAISTALVQLTGDKSSPGWWLMCAALCGLAATAMLFVRLSRGYQTAENKL>UPI00001D9947 Hcp family type VI secretion system effector OS=Citrobacter amalonaticus OX=35703 GN=ILP74_16625 SS=EMBLWGS:MBE0397010 PC=UP000642697:Unassembled WGS sequenceMPTPCYISITGQTQGNITAGAFTADSVGNIYVQGHEDEMLVQEFLHNVTVPTDPQSGQPSGQRAHKPFIFTVALNKAVPLLYNALASGEMLPKVELHWWRTSVEGKQEHYFTTRLTDATIVDMNLHMPHCQDPAQREFTQLLAVSLAYRKVEWEHIKSGTSGADDWRAPLEA>UPI0000212C14 Copper resistance protein OS=Citrobacter amalonaticus OX=35703 GN=ILP74_18250 SS=EMBLWGS:MBE0397311 PC=UP000642697:Unassembled WGS sequenceMNILITTTAFTALFCGAAFAQSSDIAHEAHRFVNNASAVSHVNSSTHENLPDRVNKNNTPSFSEMNEHERAIVAHSFMNNSASYAHQKMIEEHKKMLSGSDANSKTSSSSFNELNAGEKAALVHEQVNNAGAEAHQTQARKLRGLYSTR>UPI0000212C15 Peptidoglycan DD-metalloendopeptidase family protein OS=Citrobacter amalonaticus OX=35703 GN=ILP74_18255 SS=EMBLWGS:MBE0397312 PC=UP000642697:Unassembled WGS sequenceMYSTDVVKENAYLSATRSGLESNEIATLQRSLPSRFNLRHLKKNESLKLVLQKKAGKSRVVAYKFTSGSFNYTAYRISDKKFYNLSDTSGKGSLDYPLPATARLSSPFNPARLNPVSGKVSPHNGIDYSMPMNTKIVSVIDGKITRAEYNSTMGYFVEVTGKAGVKTRYLHLNKILVTKGARVTRGDAIALSGNSGRSSGPHLHYELVINNNPVNSLAFRAAAPADNKLEQHAFAHARDYERYLD>UPI0000212C16 DUF2933 domain-containing protein OS=Citrobacter amalonaticus OX=35703 GN=ILP74_18260 SS=EMBLWGS:MBE0397313 PC=UP000642697:Unassembled WGS sequenceMKSTTYALIAVAAIAAFALLREHWSHVAGYWPYLLLLVCPLMHLFHGHGGHGDHQHQGSENDKKN>UPI0000212C1F Copper/silver sensor histidine kinase SilS OS=Citrobacter amalonaticus OX=35703 GN=silS SS=EMBLWGS:MBE0397321 PC=UP000642697:Unassembled WGS sequenceMHSKPSRRPFSLALRLTFFISLSTILAFIAFTWFMLHSVENHFAEQDVSDLQQISTTLNRILQSPVDPDDKKISKIKESIASYRNVALLLLNPRGEVLFSSAQGAALRPAVNSADFSEHSRARDVFLWTVEDPAGPMDTGSEMKMETYRIIASSGQAIFQGKQQNYVMLTGLSINFHLHYLDALKKNLIAIAVVISLLIVLIIRIAVRQGHLPLRNVSNAIKNITSENLDARLEPTRVPIELEQLVISFNHMIGKIEDVFTRQANFSADIAHEIRTPITNLVTQTEIALSQDRTQRELEDVLYSSLEEYNRMTKMVSDMLFLAQADNNQLIPDRVMFDLRAEVMKVFEFFEAWAEERNITLKFNGMPCLVEGDPQMFRRAINNLLSNALRYTPEGQAITVSIREQESFFDLVIENPGKPIPEEHLSRLFDRFYRVDPSRQRKGEGSGIGLAIVKSIVEAHHGRVQVESDVRSTRFILSVPRLEKMIPETQC>UPI0000260692 DUF4223 family protein OS=Citrobacter amalonaticus OX=35703 GN=ILP74_20490 SS=EMBLWGS:MBE0397733 PC=UP000642697:Unassembled WGS sequenceMNTFIKMALLGAALATLTACTGHIENRNKTCSYDYLLHPAISISKMIGGCGPTAE>UPI0000274B6E Pyr operon leader peptide OS=Citrobacter amalonaticus OX=35703 GN=pyrL SS=EMBLWGS:MBE0394129 PC=UP000642697:Unassembled WGS sequenceMVQCVRHSVLPRLKTDAGLPFFFPLLTYSQPLK>UPI00002BA72A ABC transporter permease OS=Citrobacter amalonaticus OX=35703 GN=ILP74_18200 SS=EMBLWGS:MBE0397302 PC=UP000642697:Unassembled WGS sequenceMISLAGRDILHAWGKFVFTGIGLGLLIGVTLVMAGVYRGMVDDGKALLDNSGADLWVVQKDTLGPYAESSSLNDDVYRAILAMPGVSQAANATYLTMQVRKGESDVRTMVVGIAPGALGATPGWPPYLVAGRQITRGHYEAVADIATGFKLGDRLAIRRNHYTVVGLTRRMVSSSGDPMVFIPLKDAQEAQFLKDNDAIWQSRRRTEANPVFNRPGDPGLLDAVIASQSSNAFVNAVLVTLKPGHAPDEVAESIQRWKRLTVYTRAQMEDILVGKLIATSAKQIGMFLVILAIVSAAIVAFIIYSLTMDKIREIAVLKLIGTRNRTIAAMIMQQALALGVIGFVVGKITATFSAPAFPKYVLLTPMDSVAGFFAVLVICVLASLVAIRIALKVDPAEAIGG>UPI00003B8164 DUF1328 domain-containing protein OS=Citrobacter amalonaticus OX=35703 GN=ILP74_01550 SS=EMBLWGS:MBE0394204 PC=UP000642697:Unassembled WGS sequenceMFRWGIIFLVIALIAAALGFGGLAGTAAGAAKIVFVVGIILFLVSLFTGRKRP>UPI00005D0D6C TetR/AcrR family transcriptional regulator OS=Citrobacter amalonaticus OX=35703 GN=ILP74_18210 SS=EMBLWGS:MBE0397304 PC=UP000642697:Unassembled WGS sequenceMDTHPKHLPADERRAVTVESVVALAGSQNPSEITTAAIAKHMNLTQGALFRHFPNKEAIWQAVMEWVAERLLARIDRSAQGIESPLAAMEAMFMSHIEFVAEHPGVPRMMFGELQRAESTPAKRMVQTLIQRYGERLHRLIEKGKASGELSPSLDNEAAATLFIGTIQGLVMQSLLAGDVGRMHRDAPRVFAIYRRGIRSAQ>UPI00005D112D Cation efflux system protein CusF OS=Citrobacter amalonaticus OX=35703 GN=cusF SS=EMBLWGS:MBE0397318 PC=UP000642697:Unassembled WGS sequenceMRNSLKAVLFGAFSVMFSAGLHAETHQHGDMNAASDASVQQVIKGTGVVKDIDMNSKKITISHEAIPAVGWPAMTMRFTFVNADDAINALKTGNHVDFSFIQQGNISLLKSINVTQS>UPI00005EE28B AaeX family protein OS=Citrobacter amalonaticus OX=35703 GN=ILP74_19795 SS=EMBLWGS:MBE0397603 PC=UP000642697:Unassembled WGS sequenceMSLFPVIVVFGLSFPPIFFELLLSLAIFWLVRRVLVPTGIYDFVWHPALFNTALYCCLFYLISRLFV>UPI0000E1A2F2 50S ribosomal protein L27 OS=Citrobacter amalonaticus OX=35703 GN=rpmA SS=EMBLWGS:MBE0397553 PC=UP000642697:Unassembled WGS sequenceMAHKKAGGSTRNGRDSEAKRLGVKRFGGESVLAGSIIVRQRGTKFHAGTNVGCGRDHTLFAKADGKVKFEVKGPNNRKYISIVAE>UPI0000E9664A 50S ribosomal protein L36 OS=Citrobacter amalonaticus OX=35703 GN=rpmJ SS=EMBLWGS:MBE0396840 PC=UP000642697:Unassembled WGS sequenceMQVLNSLRSAKQRHPDCQIVKRKGRLYVICKSNPRFKAVQGRKKRR>UPI0000E97090 F0F1 ATP synthase subunit epsilon OS=Citrobacter amalonaticus OX=35703 GN=atpC SS=EMBLWGS:MBE0398092 PC=UP000642697:Unassembled WGS sequenceMAMTYHLDVVSAEQQMFSGLVEKIQVTGSEGELGIFPGHAPLLTAIKPGMIRIVKQFGHEEFIYLSGGILEVQPGSVTVLADTAIRGQDLDEARALEAKRKAEEHIKSSHGDVDYAQASAELAKAIAKLRVIELTKKAM>UPI0000E9A907 50S ribosomal protein L33 OS=Citrobacter amalonaticus OX=35703 GN=rpmG SS=EMBLWGS:MBE0397967 PC=UP000642697:Unassembled WGS sequenceMAKGIREKIKLVSSAGTGHFYTTTKNKRTKPEKLELKKFDPVVRQHVLYKEAKIK>UPI0000E9A97B 50S ribosomal protein L28 OS=Citrobacter amalonaticus OX=35703 GN=rpmB SS=EMBLWGS:MBE0397968 PC=UP000642697:Unassembled WGS sequenceMSRVCQVTGKRPVTGNNRSHALNATKRRFLPNLHSHRFWVESEKRFVTLRVSAKGMRVIDKKGIDTVLSELRARGEKY>UPI00013197EA Protein-export chaperone SecB OS=Citrobacter amalonaticus OX=35703 GN=secB SS=EMBLWGS:MBE0397941 PC=UP000642697:Unassembled WGS sequenceMSEQNNTEMAFQIQRIYTKDVSFEAPNAPHVFQKDWQPEVKLDLDTASTQLADDVYEVVLRVTVTASLGEETAFLCEVQQGGIFSISGIEGTQMAHCLGAYCPNILFPYARECITSLVSRGTFPQLNLAPVNFDALFMNYLQQQAGEGTEEHQDA>UPI00015761F2 P-II family nitrogen regulator OS=Citrobacter amalonaticus OX=35703 GN=glnK SS=EMBLWGS:MBE0396851 PC=UP000642697:Unassembled WGS sequenceMKLVTVVIKPFKLEDVREALSSIGIQGLTVTEVKGFGRQKGHAELYRGAEYSVNFLPKVKIDVAIADDQLDEVIDVISKAAYTGKIGDGKIFVAELQRVIRIRTGEADEAAL>UPI0001576908 Ethanolamine utilization microcompartment protein EutM OS=Citrobacter amalonaticus OX=35703 GN=eutM SS=EMBLWGS:MBE0394716 PC=UP000642697:Unassembled WGS sequenceMEALGMIETRGLVALIEASDAMVKAARVKLVGVKQIGGGLVTAMVRGDVAACKAATDAGAAAAQRIGELVSVHVIPRPHGDLEEVFPISFKGDSNI>UPI0001576C0C Fe-S cluster assembly scaffold IscU OS=Citrobacter amalonaticus OX=35703 GN=iscU SS=EMBLWGS:MBE0394647 PC=UP000642697:Unassembled WGS sequenceMAYSEKVIDHYENPRNVGSFDNSDENVGSGMVGAPACGDVMKLQIKVNNEGIIEDARFKTYGCGSAIASSSLVTEWVKGKSLDEAQAIKNTDIADELELPPVKIHCSILAEDAIKAAIADYKSKREAK>UPI000159C93D IS110 family transposase OS=Citrobacter amalonaticus OX=35703 GN=ILP74_01960 SS=EMBLWGS:MBE0394281 PC=UP000642697:Unassembled WGS sequenceMTVTNQFAAHVGLDWADKKHDVCVQFKNGERVFDVIEHTAEALDAWLTELHQKVKGRIAIALELKKGPVVYALQKYPFITVFPVHALSLARYRQAFSPSGAKDDPQDAELALELMLRYPQKIKAIEPDNADIRLLQQLVEQRRQLVEDKRRFVNRIINTLKQYYPQPLEWFSHRGSLLLCELIIRWPSLQQLKRARRDTIRNFLNAKGGRAMALTEQRVASIDNAIPLTTDPSVIEANALMAAALATQIKVVSEIIKTYDERIETLFDTLPDAGLFKSLPGMGPCMGPRMLAALGDNRDRFNSAEEIQNYAGIAPVTERSGQKSWVHWRWQCAKFVRQTFVEWAAKTVNSSYWAKLYYQGLREKGKSHQSAIRALAFKWIRIIYRCWKARTCYDEAKYLLALEARHSPLLKP>UPI00015C52A1 Elongation factor P-like protein YeiP OS=Citrobacter amalonaticus OX=35703 GN=yeiP SS=EMBLWGS:MBE0395095 PC=UP000642697:Unassembled WGS sequenceMPRANEIKKGMVLNYNGKLLIVKDIDIQSPTARGAATLYKMRFSDVRTGLKVEERFKGDDIVDTVTLSRRGVDFSYIDGNEYVFMDKEDYTPYTFTKDQIEEELLFIPEGGMPDMQVLTWDGQLLALELPQTVDLEIVETAPGIKGASASARNKPATLSTGLVIQVPEYLSAGEKIRIHIEERRYMGRAD>UPI00015C52AA F0F1 ATP synthase subunit B OS=Citrobacter amalonaticus OX=35703 GN=atpF SS=EMBLWGS:MBE0398097 PC=UP000642697:Unassembled WGS sequenceMNLNATILGQAIAFVLFVLFCMKYVWPPLMAAIEKRQKEIADGLASAERAHKDLDLAKASATDQLKKAKAEAQVIIEQANKRRAQILDEAKTEAEQERTKIVAQAQAEIDAERKRAREELRKQVAILAVAGAEKIIERSVDEAANSDIVDKLVAEL>UPI00015C5330 Transcription termination factor Rho OS=Citrobacter amalonaticus OX=35703 GN=rho SS=EMBLWGS:MBE0398141 PC=UP000642697:Unassembled WGS sequenceMNLTELKNTPVSELITLGENMGLENLARMRKQDIIFAILKQHAKSGEDIFGDGVLEILQDGFGFLRSADSSYLAGPDDIYVSPSQIRRFNLRTGDTISGKIRPPKEGERYFALLKVNEVNYDKPENARNKILFENLTPLHANSRLRMERGNGSTEDLTARVLDLASPIGRGQRGLIVAPPKAGKTMLLQNIAQSIAYNHPDCVLMVLLIDERPEEVTEMQRLVKGEVVASTFDEPASRHVQVAEMVIEKAKRLVEHKKDVIILLDSITRLARAYNTVVPASGKVLTGGVDANALHRPKRFFGAARNVEEGGSLTIIATALIDTGSKMDEVIYEEFKGTGNMELHLSRKIAEKRVFPAIDYNRSGTRKEELLTTQEELQKMWILRKIIHPMGEIDAMEFLINKLAMTKTNDDFFDMMKRS>UPI00015C55A6 DUF1869 domain-containing protein OS=Citrobacter amalonaticus OX=35703 GN=ILP74_09055 SS=EMBLWGS:MBE0395600 PC=UP000642697:Unassembled WGS sequenceMGKATYTVTVTNNSNGVSVDYETEAPMTLLVPDVAAEVVKDLVNTVRSYDTENEHDVCGW>UPI00015C5731 Stress-induced protein YchH OS=Citrobacter amalonaticus OX=35703 GN=ychH SS=EMBLWGS:MBE0395645 PC=UP000642697:Unassembled WGS sequenceMKRKNASLLGNVLMGLGLVVMVVGVGYSILNQLPQFNLPQFFAHGAVLSIFVGAILWLAGARVGGHEQVSDRYWWVRHYDKRCRRNDNRRHS>UPI00015C5A34 30S ribosomal protein S2 OS=Citrobacter amalonaticus OX=35703 GN=rpsB SS=EMBLWGS:MBE0397057 PC=UP000642697:Unassembled WGS sequenceMATVSMRDMLKAGVHFGHQTRYWNPKMKPFIFGARNKVHIINLEKTVPMFNEALAELNKIASRKGKILFVGTKRAASEAVKDAANSCDQFFVNHRWLGGMLTNWKTVRQSIKRLKDLETQSQDGTFEKLTKKEALMRTRELEKLENSLGGIKDMGGLPDALFVIDADHEHIAIKEANNLGIPVFAIVDTNSDPDGVDFVIPGNDDAIRAVSLYLGAVAATVREGRSQDLASQAEESFVEAE>UPI00015C5AB2 YbaB/EbfC family nucleoid-associated protein OS=Citrobacter amalonaticus OX=35703 GN=ILP74_15595 SS=EMBLWGS:MBE0396826 PC=UP000642697:Unassembled WGS sequenceMFGKGGLGNLMKQAQQMQEKMQQMQEEIAKLEVTGESGAGLVKVTINGAHNCRRVEIDPSLLEDDKEMLEDLVAAAFNDAARRIEETQKEKMASVSSGMQLPPGFKMPF>UPI00015C5AB3 50S ribosomal protein L7/L12 OS=Citrobacter amalonaticus OX=35703 GN=rplL SS=EMBLWGS:MBE0398384 PC=UP000642697:Unassembled WGS sequenceMSITKDQIIEAVSAMSVMDVVELISAMEEKFGVSAAAAVAVAAGPVEAAEEKTEFDVILKAAGANKVAVIKAVRGATGLGLKEAKDLVESAPAALKEGVSKDDAEALKKSLEEAGAEVEVK>UPI00015C5AB7 50S ribosomal protein L11 OS=Citrobacter amalonaticus OX=35703 GN=rplK SS=EMBLWGS:MBE0398381 PC=UP000642697:Unassembled WGS sequenceMAKKVQAYVKLQVAAGMANPSPPVGPALGQQGVNIMEFCKAFNAKTDSIEKGLPIPVVITVYADRSFTFITKTPPAAVLLKKAAGIKSGSGKPNKDKVGKISRAQLQEIAQTKAADMTGADIEAMTRSIEGTARSMGLVVED>UPI00015C5AB8 Transcription termination/antitermination protein NusG OS=Citrobacter amalonaticus OX=35703 GN=nusG SS=EMBLWGS:MBE0398380 PC=UP000642697:Unassembled WGS sequenceMSEAPKKRWYVVQAFSGFEGRVATSLREHIKLHNMEELFGEVMVPTEEVVEIRGGQRRKSERKFFPGYVLVQMVMNDASWHLVRSVPRVMGFIGGTSDRPAPISDKEVDAIMNRLQQVGDKPRPKTLFEPGEMVRVNDGPFADFNGVVEEVDYEKSRLKVSVSIFGRATPVELDFAQVEKA>UPI00015C5B7F Cell division protein FtsZ OS=Citrobacter amalonaticus OX=35703 GN=ftsZ SS=EMBLWGS:MBE0397126 PC=UP000642697:Unassembled WGS sequenceMFEPMELTNDAVIKVIGVGGGGGNAVEHMVRERIEGVEFFAVNTDAQALRKTAVGQTIQIGSGITKGLGAGANPEVGRNAADEDREALRAALDGADMVFIAAGMGGGTGTGAAPVVAEVAKDLGILTVAVVTKPFNFEGKKRMAFAEQGITELSKHVDSLITIPNDKLLKVLGRGISLLDAFGAANDVLKGAVQGIAELITRPGLMNVDFADVRTVMSEMGYAMMGSGVASGEDRAEEAAEMAISSPLLEDIDLSGARGVLVNITAGFDLRLDEFETVGNTIRAFASDNATVVIGTSLDPDMNDELRVTVVATGIGMDKRPEITLVTNKQVQQPVMDRYQQHGMAPLTQEQKPVAKVVNDNTPQTAKEPDYLDIPAFLRKQAD>UPI00015C5B80 Cell division protein FtsA OS=Citrobacter amalonaticus OX=35703 GN=ftsA SS=EMBLWGS:MBE0397127 PC=UP000642697:Unassembled WGS sequenceMIKATDRKLVVGLEIGTAKVAALVGEVLPDGMVNIIGVGSCPSRGMDKGGVNDLESVVKCVQRAIDQAELMADCQISSVYLALSGKHISCQNEIGMVPISEEEVTQEDVENVVHTAKSVRVRDEHRVLHVIPQEYAIDYQEGIKNPVGLSGVRMQAKVHLITCHNDMAKNIVKAVERCGLKVDQLIFAGLAASYSVLTEDERELGVCVVDIGGGTMDIAVYTGGALRHTKVIPYAGNVVTSDIAYAFGTPPSDAEAIKVRHGCALGSIVGKDESVEVPSVGGRPPRSLQRQTLAEVIEPRYTELLNLVNEEILQLQEQLRQQGVKHHLAAGIVLTGGAAQIEGLAACAQRVFHTQVRIGAPLNITGLTDYAQEPYYSTAVGLLHYGKESHLSGEAEVEKRVTASVGSWIKRLNSWLRKEF>UPI00015C5B90 Uncharacterized protein OS=Citrobacter amalonaticus OX=35703 GN=ILP74_01260 SS=EMBLWGS:MBE0394150 PC=UP000642697:Unassembled WGS sequenceMEQITVVIGDRLGKGQKVAAGVEKAGGRAVVVPGVAADMKLGDVMKAENATFGISFCGSGGAGAITAQNKYGYKAKYGMRSVDEGVTAINEGCNVLGFGFMDKEELGERLVQAWQKKYGA>UPI00015C5BFE TRNA guanosine(34) transglycosylase Tgt OS=Citrobacter amalonaticus OX=35703 GN=tgt SS=EMBLWGS:MBE0396900 PC=UP000642697:Unassembled WGS sequenceMKFELDTTDGRARRGRLVFDRGVVETPAFMPVGTYGTVKGMTPEEVEATGAQIILGNTFHLWLRPGQEIMKLHGDLHDFMQWKGPILTDSGGFQVFSLGDIRKITEQGVHFRNPINGDPIFLDPEKSMEIQYDLGSDIVMIFDECTPYPADWDYAKRSMEMSLRWAKRSRDRFDGLGNKNALFGIIQGSVYEDLRDISVKGLVEIGFDGYAVGGLAVGEPKEDMHRILEHVCPQIPADKPRYLMGVGKPEDLVEGVRRGIDMFDCVMPTRNARNGHLFVTDGVVKIRNAKHKSDTSPLDAECDCYTCRNYSRAYLHHLDRCNEILGARLNTIHNLRYYQRLMAGLRKAIEEGKLESFVTDFYQRQGRPVPPLNVD>UPI00015C5C88 DUF1107 domain-containing protein OS=Citrobacter amalonaticus OX=35703 GN=ILP74_00930 SS=EMBLWGS:MBE0394087 PC=UP000642697:Unassembled WGS sequenceMKIFQRYNPLQVAKYVKILFRGRLYIKDVGAFEFDKGKILIPKVKDKQHLSVMSEVNRQVLRLQTEMA>UPI00015C5D6C 23S rRNA (Pseudouridine(1915)-N(3))-methyltransferase RlmH OS=Citrobacter amalonaticus OX=35703 GN=rlmH SS=EMBLWGS:MBE0396691 PC=UP000642697:Unassembled WGS sequenceMKLQLVAVGTKMPDWVQTGFTEYLRRFPKDMPFELIEIPAGKRGKNADIKRILDKEGEQMLAAAGKNRIVTLDIPGKPWDTPQLATELERWKLDGRDVSLLIGGPEGLSPACKAAAEQSWSLSALTLPHPLVRVLVAESLYRAWSITTNHPYHRE>UPI00015C6074 50S ribosomal protein L19 OS=Citrobacter amalonaticus OX=35703 GN=rplS SS=EMBLWGS:MBE0394575 PC=UP000642697:Unassembled WGS sequenceMSNIIKQLEQEQMKQDVPSFRPGDTVEVKVWVVEGSKKRLQAFEGVVIAIRNRGLHSAFTVRKISNGEGVERVFQTHSPVVDSIAVKRRGAVRKAKLYYLRERTGKAARIKERLN>UPI00015C61F4 50S ribosomal protein L6 OS=Citrobacter amalonaticus OX=35703 GN=rplF SS=EMBLWGS:MBE0397682 PC=UP000642697:Unassembled WGS sequenceMSRVAKAPVVVPAGVDVKINGQVITIKGKNGELTRTLNDAVEVKHADNALTFGPRDGYVDGWAQAGTARALLNSMVIGVTEGFTKKLQLVGVGYRAAVKGNVVNLSLGFSHPVDHQLPAGITAECPTQTEIVLKGADKQVIGQVAADLRAYRRPEPYKGKGVRYADEVVRTKEAKKK>UPI00015C623D Bacterioferritin-associated ferredoxin OS=Citrobacter amalonaticus OX=35703 GN=bfd SS=EMBLWGS:MBE0397701 PC=UP000642697:Unassembled WGS sequenceMYVCLCNGVSDKKIRQAVRQFHPQSFQQLRKFIPVGNQCGKCVRAAREVMQDELMQMPEFKEIA>UPI00016030E5 GrxA family glutaredoxin OS=Citrobacter amalonaticus OX=35703 GN=ILP74_13560 SS=EMBLWGS:MBE0396446 PC=UP000642697:Unassembled WGS sequenceMFTVIFGRPGCPYCVRAKELAEKLSNERDDFNYRYIDIHAEGITKADLEKTVGKPVETVPQIFVDQKHIGGCTDFEAWAKENLNLFA>UPI0001603785 30S ribosomal protein S14 OS=Citrobacter amalonaticus OX=35703 GN=rpsN SS=EMBLWGS:MBE0397684 PC=UP000642697:Unassembled WGS sequenceMAKQSMKAREVKRVALADKYFAKRAELKAIISDVNASDEDRWNAVLKLQSLPRDSSPSRQRNRCRQTGRPHGYVGKFGLSRIKLREAAMRGEVPGLKKASW>UPI00016DBDB5 Silver-binding protein SilE OS=Citrobacter amalonaticus OX=35703 GN=silE SS=EMBLWGS:MBE0397322 PC=UP000642697:Unassembled WGS sequenceMKNIVLASLLGFGLISSAWATETVNIHDRVNNAQAPAHQMQSAAAPVGIQGTAPRMTGMDQHEQAIIAHETMTNGSADAHQKMVESHQKMMGNNTVSTTVPSTSYAAMNEHERAAVAHEFMNNGQSGPHQAMAEAHRRMINAG>UPI0001813D8D ATP-dependent Clp endopeptidase proteolytic subunit ClpP OS=Citrobacter amalonaticus OX=35703 GN=clpP SS=EMBLWGS:MBE0396864 PC=UP000642697:Unassembled WGS sequenceMSYSGERDNFAPHMALVPMVIEQTSRGERSFDIYSRLLKERVIFLTGQVEDHMANLIVAQMLFLEAENPEKDIYLYINSPGGVITAGMSIYDTMQFIKPDVSTICMGQAASMGAFLLTAGAKGKRFCLPNSRVMIHQPLGGYQGQATDIEIHAREILKVKGRMNELMAHHTGQSLEQIERDTERDRFLSAAEAVEYGLVDSILTHRN>UPI0001826F26 50S ribosomal protein L21 OS=Citrobacter amalonaticus OX=35703 GN=rplU SS=EMBLWGS:MBE0397554 PC=UP000642697:Unassembled WGS sequenceMYAVFQSGGKQHRVSEGQTVRLEKLDIATGESVEFAEVLMIANGEEVKIGVPFVDGGVIKAEVVAHGRGEKVKIVKFRRRKHYRKQQGHRQWFTDVKITGISA>UPI00018850FE UDP-3-O-acyl-N-acetylglucosamine deacetylase OS=Citrobacter amalonaticus OX=35703 GN=lpxC SS=EMBLWGS:MBE0397125 PC=UP000642697:Unassembled WGS sequenceMIKQRTLKRIVQATGVGLHTGKKVTLTLRPAPANTGVIYRRTDLNPPVDFPADAKSVRDTMLCTCLVNEHDVRISTVEHLNAALAGLGIDNIVIEVDAPEIPIMDGSAAPFVYLLLDAGIEELNSAKKFVRIKETVRVEDGDKWAEFRPYNGFTLDFTIDFNHPAIDSSTQRYAMNFSADAFMRQISRARTFGFMRDIEYLQSRGLCLGGSFDCAIVVDDYRVLNEDGLRFEDEFVRHKMLDAIGDLFMCGHNIIGAFTAYKSGHALNNKLLQAVLAKQEAWEYVTFQDDAELPLAFKAPSTVLA>UPI0001888804 Regulator OS=Citrobacter amalonaticus OX=35703 GN=ILP74_13905 SS=EMBLWGS:MBE0396513 PC=UP000642697:Unassembled WGS sequenceMRPNITIIIPEPYLPLDEYCRRTGTNKETARNLIEYGKLPIKPKGKQKKGLVEVNMAALTIQALSECDISLNA>UPI000199A549 Membrane protein YpdK OS=Citrobacter amalonaticus OX=35703 GN=ypdK SS=EMBLWGS:MBE0394781 PC=UP000642697:Unassembled WGS sequenceMKYFFMGISFMVIVWAGTFALMI>UPI000199A54A Protein YrbN OS=Citrobacter amalonaticus OX=35703 GN=yrbN SS=EMBLWGS:MBE0397532 PC=UP000642697:Unassembled WGS sequenceMKIADQFHDELCRLAAINFEAHVLHG>UPI00019B05E0 Arsenite efflux transporter metallochaperone ArsD OS=Citrobacter amalonaticus OX=35703 GN=arsD SS=EMBLWGS:MBE0394271|EMBLWGS:MBE0394313 PC=UP000642697:Unassembled WGS sequenceMKTLTVFDPAMCCSTGVCGSDVDQVLVDFSADVQWLKGRGVQVERYNLAQQPMSFVQNEKAKAFLEASGAEGLPLLLLDGETVMAGRYPKRAELARWFGIPLEKVGLAPTSCCGGNTSCC>UPI00019B05E7 Arsenical resistance protein ArsH OS=Citrobacter amalonaticus OX=35703 GN=arsH SS=EMBLWGS:MBE0394278 PC=UP000642697:Unassembled WGS sequenceMTGDLKNVDAGLFDTSITEARFGVPAVPHPPRILMLYGSVRERSYSRLATEEAARLLTAMGAEVRIFNPSGLPLPDDAPDSHPKVMELRELVRWSEGMVWCSPERHGAMTGIMKAQIDWIPLSEGAVRPSQGKTLAVMQVCGGSQSFNAVNQMRILGRWMRMITIPNQSSVAKAWQEFDEDGRMKPSSYYDRIVDVMEELVKFTLLTRGNSAYLVDRYSERKESAEELSRRVNQSKI>UPI00019B0BCA Electron transport complex subunit RsxA OS=Citrobacter amalonaticus OX=35703 GN=rsxA SS=EMBLWGS:MBE0395984 PC=UP000642697:Unassembled WGS sequenceMTDYLLLFVGTVLVNNFVLVKFLGLCPFMGVSKKLETAMGMGLATTFVMTLASICAWLIDTWILIPLNLIYLRTLAFILVIAVVVQFTEMVVRKTSPALYRLLGIFLPLITTNCAVLGVALLNINLGHNFLQSALYGFSAAVGFSLVMVLFAAIRERLAVADVPAPFRGNAIALITAGLMSLAFMGFSGLVKL>UPI00019B1144 NADH-quinone oxidoreductase subunit NuoB OS=Citrobacter amalonaticus OX=35703 GN=nuoB SS=EMBLWGS:MBE0394933 PC=UP000642697:Unassembled WGS sequenceMDYTLTRIDPNGENDRYPLQKQEIVTDPLEQEVNKNVFMGKLHDMVNWGRKNSIWPYNFGLSCCYVEMVTSFTAVHDVARFGAEVLRASPRQADLMVVAGTCFTKMAPVIQRLYDQMLEPKWVISMGACANSGGMYDIYSVVQGVDKFIPVDVYIPGCPPRPEAYMQALMLLQDSIGKERRPLSWVVGDQGVYRANMQSERERKRGERIAVTNLRTPDEI>UPI00019B1222 Ribosome hibernation promoting factor OS=Citrobacter amalonaticus OX=35703 GN=hpf SS=EMBLWGS:MBE0397570 PC=UP000642697:Unassembled WGS sequenceMQLNITGNNVEITEALRDFVNTKFAKLEQYFDRINQVYIVLKVEKVTHISDATLHVNGGEIHASAEGQDMYAAIDGLIDKLARQLTKHKDKLKQH>UPI00019B12FA 30S ribosomal protein S20 OS=Citrobacter amalonaticus OX=35703 GN=rpsT SS=EMBLWGS:MBE0397201 PC=UP000642697:Unassembled WGS sequenceMANIKSAKKRAVQSEKARKHNASRRSMMRTFIKKVYAAIEAGDKATALKAFNEMQPIVDRQAAKGLIHKNKAARHKANLTAQINKLA>UPI00019B1343 S-ribosylhomocysteine lyase OS=Citrobacter amalonaticus OX=35703 GN=luxS SS=EMBLWGS:MBE0394518 PC=UP000642697:Unassembled WGS sequenceMPLLDSFTVDHTRMEAPAVRVAKTMNTPHGDAITVFDLRFCIPNKEVMPEKGIHTLEHLFAGFMRNHLNGNGVEIIDISPMGCRTGFYMSLIGTPDEQRVADAWKAAMADVLKVQDQNQIPELNVYQCGTYQMHSLSEAQDIARHILERDVRVNSNEELALPKEKLQELHI>UPI00019B15D3 3-hydroxyacyl-ACP dehydratase FabZ OS=Citrobacter amalonaticus OX=35703 GN=fabZ SS=EMBLWGS:MBE0397046 PC=UP000642697:Unassembled WGS sequenceMTTNTHTLHIEEILELLPHRFPFLLVDRVLDFEEGRFLRAVKNVSVNEPFFQGHFPGKPIFPGVLILEAMAQATGILAFKSVGKLEPGELYYFAGIDEARFKRPVVPGDQMIMEVTFEKTRRGLTRFKGVALVDGKVVCEATMMCARSREA>UPI00019B177C PTS lactose/cellobiose transporter subunit IIA OS=Citrobacter amalonaticus OX=35703 GN=ILP74_22020 SS=EMBLWGS:MBE0398030 PC=UP000642697:Unassembled WGS sequenceMIALEEAVMEIIVNAGQSRSLCFEALHAARAGNIDEAKSLLREADGYARQAHHMQTKLIEQDAGEARQPMTLIMVHAQDHLMTSLLARELSEEIIHLYQR>UPI00019B184C F0F1 ATP synthase subunit beta OS=Citrobacter amalonaticus OX=35703 GN=atpD SS=EMBLWGS:MBE0398093 PC=UP000642697:Unassembled WGS sequenceMATGKIVQVIGAVVDVEFPQDAVPRVYDALEVMNGKESLVLEVQQQLGGGIVRTIAMGSSDGLRRGLEVKDLEHPIEVPVGKATLGRIMNVLGHPIDMKGDIGEEERWAIHRAAPSYEELSSSQELLETGIKVIDLMCPFAKGGKVGLFGGAGVGKTVNMMELIRNIAIEHSGYSVFAGVGERTREGNDFYHEMTDSNVLDKVSLVYGQMNEPPGNRLRVALTGLTMAEKFRDEGRDVLLFVDNIYRYTLAGTEVSALLGRMPSAVGYQPTLAEEMGVLQERITSTKTGSITSVQAVYVPADDLTDPSPATTFAHLDATVVLSRQIASLGIYPAVDPLDSTSRQLDPLVVGQEHYDTARGVQSLLQRYQELKDIIAILGMDELSEEDKLVVARARKIQRFLSQPFFVAEVFTGSPGKYVSLKDTIRGFKGIMEGEYDHLPEQAFYMVGSIDEAVEKAKKL>UPI00019F139D Iron-sulfur cluster assembly protein IscA OS=Citrobacter amalonaticus OX=35703 GN=iscA SS=EMBLWGS:MBE0394648 PC=UP000642697:Unassembled WGS sequenceMSITLSDSAAARVNTFLANRGKGFGLRLGVRTSGCSGMAYVLEFVDEPAAEDTVFEDKGVKVVVDGKSLQFLDGTQLDFVKEGLNEGFKFTNPNVKDECGCGESFHV>UPI00019F19DD Metal/formaldehyde-sensitive transcriptional repressor OS=Citrobacter amalonaticus OX=35703 GN=ILP74_10180 SS=EMBLWGS:MBE0395813 PC=UP000642697:Unassembled WGS sequenceMPHSPEDKKRILTRVRRIRGQVDALERALESGEPCLAILQQIAAVRGASNGLMGEMVEIHLKDELVSGETTPDQRAVRMAEIGHLLRAYLK>UPI00019F218C Peptidoglycan-associated lipoprotein Pal OS=Citrobacter amalonaticus OX=35703 GN=pal SS=EMBLWGS:MBE0396590 PC=UP000642697:Unassembled WGS sequenceMQLNKVLKGLMIALPVMAIAACSSNKNASNDGSEGMLGAGTGMDANGSGNMSSEEQARLQMQQLQQNNIVYFDLDKYDIRSDFAAMLDAHANFLRSNPSYKVTVEGHADERGTPEYNISLGERRANAVKMYLQGKGVSADQISIVSYGKEKPAVLGHDEAAYSKNRRAVLVY>UPI0001C092E7 RNase adapter RapZ OS=Citrobacter amalonaticus OX=35703 GN=rapZ SS=EMBLWGS:MBE0397572 PC=UP000642697:Unassembled WGS sequenceMVLMIVSGRSGSGKSVALRALEDMGFYCVDNLPVVLLPDLARTLAERQISAAVSIDVRNMPESPEIFEQAMNNLPEAFSPQLLFLDADRNTLIRRYSDTRRLHPLSSKNLSLESAIDQESDLLEPLRSRADLIVDTSEMSVHELAEMLRTRLLGKRERELTMVFESFGFKHGIPIDADYVFDVRFLPNPHWDPKLRPMTGLDKPVAAFLDRHTEVHNFIYQTRSYLELWLPMLETNNRSYLTVAIGCTGGKHRSVYIAEQLADYFRSRGKNVQSRHRTLEKRKT>UPI0001C09562 Glutaredoxin 3 OS=Citrobacter amalonaticus OX=35703 GN=grxC SS=EMBLWGS:MBE0397942 PC=UP000642697:Unassembled WGS sequenceMANIEIYTKATCPFCHRAKALLNSKGVSFQELPIDGDAVKREEMIKRSGRTTVPQIFIDAQHIGGCDDLYALDARGGLDPLLS>UPI0001C09646 YpfN family protein OS=Citrobacter amalonaticus OX=35703 GN=ILP74_04215 SS=EMBLWGS:MBE0394697 PC=UP000642697:Unassembled WGS sequenceMDWLAKYWWILVLVFLVGVLLNVIKDLKRVDHKKFLANKPELPPHRDFNDKWDDDDDWPKKDQPKK>UPI0001C096D2 Uracil phosphoribosyltransferase OS=Citrobacter amalonaticus OX=35703 GN=upp SS=EMBLWGS:MBE0394679 PC=UP000642697:Unassembled WGS sequenceMKIVEVKHPLVKHKLGLMRENDISTKRFRELASEVGSLLTYEATAGLETEKVTIEGWNGPVEVDQIKGKKITVVPILRAGLGMMEGVLENVPSARISVVGMYRNEETLEPVPYFQKLVSNIDERMALIVDPMLATGGSVIATIDLLKKAGCSSIKVLVLVAAPEGIAALEKAHPDVELYTASIDQGLNEHGYIIPGLGDAGDKIFGTK>UPI0001C09790 Nitrogen regulatory protein P-II OS=Citrobacter amalonaticus OX=35703 GN=glnB SS=EMBLWGS:MBE0394625 PC=UP000642697:Unassembled WGS sequenceMKKIDAIIKPFKLDDVREALAEVGITGMTVTEVKGFGRQKGHTELYRGAEYMVDFLPKVKIEIVVPDDIVETCVDTIIRTAQTGKIGDGKIFVFDVARVVRIRTGEEDDAAI>UPI0001C09878 Preprotein translocase subunit SecE OS=Citrobacter amalonaticus OX=35703 GN=secE SS=EMBLWGS:MBE0398379 PC=UP000642697:Unassembled WGS sequenceMSANTEAQGSGRGLEAIKWIVVVALLLVAIVGNYLYRDMMLPLRALAVVILIAAAGGVALLTTKGKATVAFAREARTEVRKVIWPTRQETLHTTLIVAAVTAVMSLILWGLDGILVRLVSFITGLRF>UPI0001C09A9B 30S ribosomal protein S17 OS=Citrobacter amalonaticus OX=35703 GN=rpsQ SS=EMBLWGS:MBE0397688 PC=UP000642697:Unassembled WGS sequenceMTDKIRTLQGRVVSDKMEKSIVVAIERFVKHPIYGKFIKRTTKLHVHDENNECGIGDKVEIRECRPLSKTKSWTLVRVVEKAVL>UPI0001C09AA0 30S ribosomal protein S13 OS=Citrobacter amalonaticus OX=35703 GN=rpsM SS=EMBLWGS:MBE0397675 PC=UP000642697:Unassembled WGS sequenceMARIAGINIPDQKHAVIALTSIYGIGKTRSKAILAAAGIAENVKISELSEGQIDTLRDEVAKFVVEGDLRREISMSIKRLMDLGCYRGLRHRRGLPVRGQRTKTNARTRKGPRKPIKK>UPI0001C09B2A 50S ribosomal protein L17 OS=Citrobacter amalonaticus OX=35703 GN=rplQ SS=EMBLWGS:MBE0397671 PC=UP000642697:Unassembled WGS sequenceMRHRKSGRQLNRNSSHRQAMFRNMAGSLVRHEIIKTTLPKAKELRRVVEPLITLAKTDSVANRRLAFARTRDNEIVAKLFNELGPRFASRAGGYTRILKCGFRAGDNAPMAYIELVDRSESKAEAAAE>UPI0001C09B34 DUF494 domain-containing protein OS=Citrobacter amalonaticus OX=35703 GN=ILP74_20130 SS=EMBLWGS:MBE0397661 PC=UP000642697:Unassembled WGS sequenceMFDVLMYLFETYIHNEAELRVDQDKLERDLTDAGFDREDIYNALLWLEKLADYQEGLAEPMQLASDPLSVRIYTPEECDRLDASCRGFLLFLEQIQVLNLETREMVIERVLALDTAEFDLEDLKWVILMVLFNIPGCENAYQQMEELLFEVNEGMLH>UPI0001C09E26 FtsH protease modulator YccA OS=Citrobacter amalonaticus OX=35703 GN=yccA SS=EMBLWGS:MBE0396335 PC=UP000642697:Unassembled WGS sequenceMDRIVSSSHDRTSLLSTHKVLRNTYFLLSLTLAFSAITATASTVLMLPSPGLILTLVGMYGLMFLTYKTANKPSGIISAFAFTGFLGYILGPILNAYLSAGMGDVIGMALGGTALVFFCCSAYVLTTRKDMSFLGGMLMAGIVVVLIGMVANIFLQLPALHLAISAVFILISSGAILFETSNIIRGGETNYIRATVSLYVSLYNIFVSLLSILGFASRD>UPI0001C0A0BD Ribosome silencing factor OS=Citrobacter amalonaticus OX=35703 GN=rsfS SS=EMBLWGS:MBE0396690 PC=UP000642697:Unassembled WGS sequenceMQGKALQDFVIDKIDDLKGQDIIALDVQGKSSITDCMIICTGTSSRHVMSIADHVVQESRAAGMLPLGVEGESVADWIVVDLGEVIVHVMQEESRRLYELEKLWS>UPI0001C0A120 Cell division protein FtsL OS=Citrobacter amalonaticus OX=35703 GN=ftsL SS=EMBLWGS:MBE0397137 PC=UP000642697:Unassembled WGS sequenceMISRVTEALSKVKGSIGSNERHALPGVIGDDLLRFGKLPLCLFICIIVTAVTVVTTAHHTRLLTAQREQLVLERDALDIEWRNLILEENALGDHSRVERIATEKLQMQHVDPSQENIVVQK>UPI0001C0A3DB Propanediol utilization microcompartment protein PduA OS=Citrobacter amalonaticus OX=35703 GN=pduA SS=EMBLWGS:MBE0395287 PC=UP000642697:Unassembled WGS sequenceMQQEALGMVETKGLTAAIEAADAMVKSANVMLVGYEKIGSGLVTVIVRGDVGAVKAATDAGAAAARNVGEVKAVHVIPRPHTDVEKILPKGINP>UPI0001C0A3E5 Propanediol utilization microcompartment protein PduN OS=Citrobacter amalonaticus OX=35703 GN=pduN SS=EMBLWGS:MBE0395276 PC=UP000642697:Unassembled WGS sequenceMHLARVTGAVVSTQKSPSLVGKKLLLVRRVSADGELPANPTAGDEVAVDSVGAGVGELVLLSSGSSARHVFSGPNEAIDLAVVGIVDTLSR>UPI0001C3404C Ribosome-associated translation inhibitor RaiA OS=Citrobacter amalonaticus OX=35703 GN=raiA SS=EMBLWGS:MBE0394586 PC=UP000642697:Unassembled WGS sequenceMTMNITSKQMEITPAIRQHVADRLAKLEKWQTHLINPHIILSKEPQGFIADATINTPNGHLVASAKHEDMYTAINDLINKLERQLNKVQHKGEARRATTSVKDANFVEAEEE>UPI0001C340E2 50S ribosomal protein L35 OS=Citrobacter amalonaticus OX=35703 GN=rpmI SS=EMBLWGS:MBE0396084 PC=UP000642697:Unassembled WGS sequenceMPKIKTVRGAAKRFKKTGGGGFKRKHANLRHILTKKSTKRKRHLRPKGLVSKGDLGLVIACLPYA>UPI0001C341CB Alkyl hydroperoxide reductase subunit C OS=Citrobacter amalonaticus OX=35703 GN=ahpC SS=EMBLWGS:MBE0396720 PC=UP000642697:Unassembled WGS sequenceMSLINTKIKPFKNQAFKNGEFIEVTEKDTEGRWSVFFFYPADFTFVCPTELGDVADHYEELQKLGVDVYSVSTDTHFTHKAWHSSSDTIAKIKYAMIGDPTGALTRNFDNMREDEGLADRATFVVDPQGIIQAIEVTAEGIGRDASDLLRKIKAAQYVASHPGEVCPAKWKEGEATLAPSLDLVGKI>UPI0001C34249 Fumarate/nitrate reduction transcriptional regulator Fnr OS=Citrobacter amalonaticus OX=35703 GN=fnr SS=EMBLWGS:MBE0395770 PC=UP000642697:Unassembled WGS sequenceMIPEKRIIRRIQSGGCAIHCQDCSISQLCIPFTLNEHELDQLDNIIERKKPIQKGQTLFKAGDELKSLYAIRSGTIKSYTITEQGDEQITGFHLAGDLVGFDAIGSGHHPSFAQALETSMVCEIPFETLDDLSGKMPNLRQQMMRLMSGEIKGDQDMILLLSKKNAEERLAAFIYNLSRRFAQRGFSPREFRLTMTRGDIGNYLGLTVETISRLLGRFQKSGMLAVKGKYITIENSDALAVLAGHTRNVA>UPI0001CB9476 IS5-like element ISKpn26 family transposase OS=Citrobacter amalonaticus OX=35703 GN=ILP74_18150 SS=EMBLWGS:MBE0397292 PC=UP000642697:Unassembled WGS sequenceMSHQLTFADSEFSTKRRQTRKEIFLSRMEQILPWQNMTAVIEPFYPKAGNGRRPYPLETMLRIHCMQHWYNLSDGAMEDALYEIASMRLFARLSLDSALPDRTTIMNFRHLLEQHQLARQLFKTINRWLAEAGVMMTQGTLVDATIIEAPSSTKNKEQQRDPEMHQTKKGNQWHFGMKAHIGVDAKSGLTHSLVTTAANEHDLNQLGNLLHGEEQFVSADAGYQGAPQREELAEVDVDWLIAERPGRVKTLKQHPRKNKTAINIEYMKASIRARVEHPFRIIKRQFGFVKARYKGLLKNDNQLAMLFTLANLFRVDQMIRQWERSQ>UPI0001CF70A1 Phage repressor protein OS=Citrobacter amalonaticus OX=35703 GN=ILP74_13910 SS=EMBLWGS:MBE0396514 PC=UP000642697:Unassembled WGS sequenceMSTFKIDLNVDSTPILDRVIEAYGFTQKLQLAEHLDMAASSLSSRYKRGVFPADIVVKCVAETGANLEWLATGQGRKFNDDELDILKIPRSKIVDGQLYDAGTLMLDKVIFLPGKPLPQQPICVLDGLVQYIVDQSYSEVYDDDWLVEVEGKTSVRTLTRIPVRKVRVSGVGMAFDCGIDDIKIIGRVVLTIK>UPI0001D14B62 Bifunctional 3-hydroxydecanoyl-ACP dehydratase/trans-2-decenoyl-ACP isomerase OS=Citrobacter amalonaticus OX=35703 GN=fabA SS=EMBLWGS:MBE0396350 PC=UP000642697:Unassembled WGS sequenceMVDKRESYTKEDLLASGRGELFGAKGPQLPAPNMLMMDRVVKMTETGGNFDKGYVEAELDINPDLWFFGCHFIGDPVMPGCLGLDAMWQLVGFYLGWLGGEGKGRALGVGEVKFTGQVLPSAKKVTYRIHFKRIVNRRLIMGLADGEVLVDGRLIYTANDLKVGLFQDTSAF>UPI0001D1571D Uncharacterized protein OS=Citrobacter amalonaticus OX=35703 GN=ILP74_18270 SS=EMBLWGS:MBE0397315 PC=UP000642697:Unassembled WGS sequenceMKKVVLMALALGLSLPAMASEKVIDMYKSENCGCCSLWGKAMEKDGFEVRTHVMNDQALSALKEKHAVPAGLRSCHTAVVGNLIIEGHVPAATIHKAMQSGSGIYGLATPGMPAGSPGMEMGARKEAYDVIAFSPEGSKKVFQRIE>UPI0001DC4835 Helix-turn-helix transcriptional regulator OS=Citrobacter amalonaticus OX=35703 GN=ILP74_02010 SS=EMBLWGS:MBE0394288 PC=UP000642697:Unassembled WGS sequenceMLMNEDTKRQTDMKNAALRAADVLRGLANSDRLLLMCQLSLGEANVAGLEAAVGITQPTLSQQLGVMRRLKLVETRRAGKMVYYRIEDNRIMTLLNTLYDLYCPQAGTGKGSHNNE>UPI0001DC4837 DNA starvation/stationary phase protection protein OS=Citrobacter amalonaticus OX=35703 GN=ILP74_02005 SS=EMBLWGS:MBE0394287 PC=UP000642697:Unassembled WGS sequenceMSKQIIDIGIAESSRVEIADGLSRLLADTYTLYIKTHYYHWNVTGPMFNSLHLMFENQYNELATAVDDIAERIRSLGCFAPGTYHEFSRLTAVNEDKDIPAAKNMIENLVQGQETVVKTARSLFPLVNNANDEATADLLTQRIQLHEKTAWMLRSLLE>UPI0001F919C2 Tyrosine-type recombinase/integrase OS=Citrobacter amalonaticus OX=35703 GN=ILP74_13915 SS=EMBLWGS:MBE0396515 PC=UP000642697:Unassembled WGS sequenceMSVRKLPTGEWIADFYTVNRSNGKNGKRIRKKFATKGEALAFENHTLQKVDAAPWLGEGKDKRTLIDLITMWYERHGVALSNGEKRKDAMTWAAECMGFPLATEFNAQLFTAYRAKRLDGHYARTNRVSKVSPKTMNLEHAYFLAMFNELKRIGEWSAPNPLENVRQYRTDETEMAFLTTEEIDRLLLECKRSKVKYLELVVKICLATGARWNEAATLKSSQIAGGKVTFVKTKGKRNRTIPLDDELLSELPETKGALFPKPCYNAFRSALERAGIELPSGQLTHVLRHTFASHFMMNGGNILVLQKILGHADITMTMRYAHFAPSHLEDAVRLNPLKCRKNVATA>UPI0001F91BD9 Terminase OS=Citrobacter amalonaticus OX=35703 GN=ILP74_13840 SS=EMBLWGS:MBE0396500 PC=UP000642697:Unassembled WGS sequenceMAKYSEELKGVVRALYLRRYTPKEIASELNLPNARIVYYWAEKYSWADLLSFESTEEAIERRYQLLASRDNKTDLDLKEMDMLIAHATKLRAQSNKHKEKMASGQSSGQAAARDNNDDEPRSKRKYKKNDITSLTQEDFDAWAEEHLFEYQKHLRNNIGQLVRNILKSRQIGATWYFAFEAFENAVMTGDPQIFLSASKAQAEVFRSYIVNIAEQYFGITLTGNPIRLSNGAELRFLSTNKNTAQSYSGHLYCDEYFWVPNFAKLNEVASAMATHDKWRTTYFSTPSAKTHQAYPFWTGDEWKQGSKKRAAIKFPSFNEMRDGGRLCPDGQWRYVITMEDAIAGGFNLANIEKLRNRYNDATFNMLYMCVFVDSKDSVFSFSDLEACGVEIDTWQDHNPDAARPFGDRPVWGGFDPARSGDLSCFVIIAPPMLAVEKFRVLKVIYWKGMNFRYQAKQIEQLFKKYNFTYLGVDVTGIGQGVFDNIQHFAMRVAVPIRYDLNTKNKLVLKAVDVVESQRIEWDKNLKEIAASFMSVRRTTTQSGNAMTFVADRSQDTGHAEAFWAITHGLHNEPLNYENKPKSRWGVRKEAA>UPI0001F91BE0 Uncharacterized protein OS=Citrobacter amalonaticus OX=35703 GN=ILP74_13805 SS=EMBLWGS:MBE0396493 PC=UP000642697:Unassembled WGS sequenceMIAGGELNKKQLAELRKALASMELPPRKRQRLIWRLAKYGVIAAAKRHVRNQESPDGQKWPGRKTKRKGKMLRNLPKLLHIREMPEIQAVRIYLQGGGYRNGETPVPAGTVGYAQQNGMRVKVSRSNQPRKAEAGKMATPAQAKKLRALGYRVKTGKRWKKPTLGEITKTMPYSQAGLLIRKLSGKAVKTSWTVDLPARVFLGMNDDEFDKALARQLQAIGFGWDVKAQDIKGKA>UPI0001F91BE2 DUF2597 family protein OS=Citrobacter amalonaticus OX=35703 GN=ILP74_13795 SS=EMBLWGS:MBE0396491 PC=UP000642697:Unassembled WGS sequenceMTKRISGMSFDTYMDGDLIHIEKITLDITDNSAAAQTRGVPDGYVDGDVAAEGEIEVSSKTLQVLTAKARAAGSWRGLPPLDFLFYAKASSEEMKVETFGNKLQVSNLLDIDPKGGGVTTHKIKYFVTSPKFVNINGVPYLEAEATENLIG>UPI0001F91BE3 Phage holin family protein OS=Citrobacter amalonaticus OX=35703 GN=ILP74_13790 SS=EMBLWGS:MBE0396490 PC=UP000642697:Unassembled WGS sequenceMQEHEKSLYSLLIIGALIAIGNVLTSNDPITPRLFAGRVILGSLVSVVAGAVLIQIPDASPLAIQGLGAALGIAGYQAVEVWLRRRAAGKQNGSKTNDPE>UPI0001F91BE5 Uncharacterized protein OS=Citrobacter amalonaticus OX=35703 GN=ILP74_13780 SS=EMBLWGS:MBE0396488 PC=UP000642697:Unassembled WGS sequenceMSSGQWLVVVALAFVWGWLTADWRRDSLELAINTAAQVAGNESRKVMQGIASDSARELEDKLEALKNVAPREIRTEILKPVFTNRCLSDEFVSMYNSAAAGTERALSGKPEN>UPI0001F91BE7 Phage tail assembly chaperone OS=Citrobacter amalonaticus OX=35703 GN=ILP74_13775 SS=EMBLWGS:MBE0396487 PC=UP000642697:Unassembled WGS sequenceMEQIKLCVCGVDIVFEPNQTAYNKFINEMAMDNKVAPAHNYLTRIVATESKEALAEVLKRPGAALQLVGKVNDIYAPELEIEVKN>UPI0001F91BE9 DUF2590 family protein OS=Citrobacter amalonaticus OX=35703 GN=ILP74_13765 SS=EMBLWGS:MBE0396485 PC=UP000642697:Unassembled WGS sequenceMSDVLYIDLLIQGGDFVLNTGNEPELCNNRKSIGQDIIHSIIESGLATELIAERSPTMRADIFTRMELLIEDDERIVPGTVEISEESQKRLWVTASTYDFGGISAQVDL>UPI0001F927D8 Sulfurtransferase TusA family protein OS=Citrobacter amalonaticus OX=35703 GN=ILP74_07210 SS=EMBLWGS:MBE0395261 PC=UP000642697:Unassembled WGS sequenceMAIKKLDVVTQVCPFPLIEAKAALAEMASGDELVIEFDCTQATEAIPQWAAEEGHAITDYQQVGDAAWSITVQKA>UPI0001FB5E88 YacC family pilotin-like protein OS=Citrobacter amalonaticus OX=35703 GN=ILP74_17090 SS=EMBLWGS:MBE0397094 PC=UP000642697:Unassembled WGS sequenceMKTFFRTVLFGSLMAVCANSYALSESEAEDMADLTAVFVFLKNDCGYQNLPNGQIRRALVFFAQQNQWDLSNYDTFDMKSLGEDSYRDLSGIGIPVAKKCKALARDSLSLLAYVK>UPI00020DA772 DUF2857 domain-containing protein OS=Citrobacter amalonaticus OX=35703 GN=ILP74_18550 SS=EMBLWGS:MBE0397368 PC=UP000642697:Unassembled WGS sequenceMMNSLSQAANGLLMQLVMDLRSGYLRRCESLGLNREEMQMLQGLSLEELHYLSGSEVSIISVGINHGNLVRMLQQARTEQKRLQRIDRALALGGSIELMANYFGLSSTDVAARRRIAGIDVRPGRGNALGDEENAALWRQWQKSGVEDAESADGLDVMMLAAEQMNVSLTSVWHAVRGWHKTRQPSPARTPVRKTA>UPI000217D2DC Copper/silver response regulator transcription factor SilR OS=Citrobacter amalonaticus OX=35703 GN=silR SS=EMBLWGS:MBE0397320 PC=UP000642697:Unassembled WGS sequenceMKILIVEDEIKTGEYLSKGLTEAGFVVDHADNGLTGYHLAMTAEYDLVILDIMLPDVNGWDIIRMLRTAGKGMPVLLLTALGTIEHRVKGLELGADDYLVKPFAFAELLARVRTLLRRGNTMITESQFKVADLSIDLVSRKVSRAGNRIVLTSKEFSLLEFFIRHQGEVLPRSLIASQVWDMNFDSDTNAIDVAVKRLRAKIDNDYGTKLIQTVRGVGYMLEVPDA>UPI000223C9B7 Excisionase family protein OS=Citrobacter amalonaticus OX=35703 GN=ILP74_07965 SS=EMBLWGS:MBE0395396 PC=UP000642697:Unassembled WGS sequenceMSEMTLIVPNDWVTEEKLVEITGLRPGTIERARKKCWMVGREYLHVSPDGVPKKNSECMYNRKAVDQWVESMSKKQPGARQ>UPI00023046B5 DUF4224 domain-containing protein OS=Citrobacter amalonaticus OX=35703 GN=ILP74_12960 SS=EMBLWGS:MBE0396333 PC=UP000642697:Unassembled WGS sequenceMTRENDLLTDAELIEFTGYQKPSKQREILDRGGVSYIPDREGRPMVTWTHINAVLNGQITVQTSTEEKPDFGAI>UPI00024125FD RNA polymerase-binding protein DksA OS=Citrobacter amalonaticus OX=35703 GN=dksA SS=EMBLWGS:MBE0397079 PC=UP000642697:Unassembled WGS sequenceMQEGQNRKTSSLSILAIAGVEPYQEKPGEEYMNEAQLSHFRRILEAWRNQLRDEVDRTVTHMQDEAANFPDPVDRAAQEEEFSLELRNRDRERKLIKKIEKTLKKVEDEDFGYCESCGVEIGIRRLEARPTADLCIDCKTLAEIREKQMAG>UPI0002412B6E Elongation factor Tu OS=Citrobacter amalonaticus OX=35703 GN=tuf SS=EMBLWGS:MBE0397702 PC=UP000642697:Unassembled WGS sequenceMSKEKFERTKPHVNVGTIGHVDHGKTTLTAAITTVLAKTYGGAARAFDQIDNAPEEKARGITINTSHVEYDTPTRHYAHVDCPGHADYVKNMITGAAQMDGAILVVAATDGPMPQTREHILLGRQVGVPYIIVFLNKCDMVDDEELLELVEMEVRELLSQYDFPGDDTPIVRGSALKALEGEAEWEAKIIELAGFLDSYIPEPERAIDKPFLLPIEDVFSISGRGTVVTGRVERGIIKVGEEVEIVGIKETAKSTCTGVEMFRKLLDEGRAGENVGVLLRGIKREEIERGQVLAKPGSIKPHTKFESEVYILSKDEGGRHTPFFKGYRPQFYFRTTDVTGTIELPEGVEMVMPGDNIKMVVTLIHPIAMDDGLRFAIREGGRTVGAGVVAKVMS>UPI00024F5F5F Integration host factor subunit beta OS=Citrobacter amalonaticus OX=35703 GN=ihfB SS=EMBLWGS:MBE0396384 PC=UP000642697:Unassembled WGS sequenceMTKSELIERLATQQSHIPAKAVEDAVKEMLEHMASTLAQGERIEIRGFGSFSLHYRAPRTGRNPKTGDKVDLEGKYVPHFKPGKELRDRANIYG>UPI00024FBE15 PTS sugar transporter subunit IIB OS=Citrobacter amalonaticus OX=35703 GN=ILP74_16000 SS=EMBLWGS:MBE0396905 PC=UP000642697:Unassembled WGS sequenceMLVIRTVCGNGIGSSLMAANNVKKICDELGIKADVASVDFANAVGEKADLYVTIKELANQFPAHCNVAIIRSYVHKAKIAEDITEALTKIAATHS>UPI00024FBF05 Acetyltransferase OS=Citrobacter amalonaticus OX=35703 GN=ILP74_18395 SS=EMBLWGS:MBE0397337 PC=UP000642697:Unassembled WGS sequenceMMTLKYPEPAIHEHSGGALFTLSPQGEPGVLPATHQHLVRLRAMLRQRLTGPVKMTCHPHRVGLSSSVAIYLEGKLKQAVNILITVTGQTSWPQEEEYAHPRWYITVPDSADLVYLMLWINGLDV>UPI00024FBF0A TIGR03746 family integrating conjugative element protein OS=Citrobacter amalonaticus OX=35703 GN=ILP74_18425 SS=EMBLWGS:MBE0397343 PC=UP000642697:Unassembled WGS sequenceMSRFRHAVKDRDQHIQTLRIACAVLAFFLLFTCAGWMLAPSKLTVHNPPDLRTGSTRPWWEVPPPTVYSFAFYIFQQLNAWPKNGEVDYSAKINALSPYLTPSCQDFLKADAKKRGDAGELTDRVRVVYEVPGRGYQSQSVTVQDRDHWIARLDVVADEYFHAEPVKRALVRYPLKVVRWEGDAERNPFGLALDCYAGVPQRLEAAPPAPKPEKSGVFQ>UPI00024FC4FD DUF2442 domain-containing protein OS=Citrobacter amalonaticus OX=35703 GN=ILP74_18500 SS=EMBLWGS:MBE0397358 PC=UP000642697:Unassembled WGS sequenceMTISAKRVSFDEATMWVELNDARTIGVPLAWFPRLLHASTEQLNSYELSPRGIHWDALDEDISIAGLLEGRGDVTHRPHKVA>UPI00024FC503 TIGR03761 family integrating conjugative element protein OS=Citrobacter amalonaticus OX=35703 GN=ILP74_18530 SS=EMBLWGS:MBE0397364 PC=UP000642697:Unassembled WGS sequenceMSDGKTKGGTTASRAGALQSSVNILLHTHYAIRLWEGRKRDAPDETGVKKKRPEIISMPQAIARAGNASRDSAADNPYADMALVRLEEALQRATLKINENVSSLDAILSAVPKGVTLSEVESADPLNVSVFSRSPLGYRCVWLLVGYDQLAMKAFQAFHYGLISRSQRDAILDNGGHAVRQVYGVIQPYRTLAVTRRDIAEKTTQGLVAIERNGEPDPDVLSGKKRSSFSPPLKNTIAEEE>UPI00024FC505 Uncharacterized protein OS=Citrobacter amalonaticus OX=35703 GN=ILP74_18545 SS=EMBLWGS:MBE0397367 PC=UP000642697:Unassembled WGS sequenceMKYRMVAPGLRAPHGTPPQCCQKALRQVRRFRQGARNYTRLDEKGCGYYKIDLGPFWRLLSRNEGRTWLLLSHERYNSAIRK>UPI00024FF875 Cu(+)/Ag(+) efflux RND transporter permease subunit SilA OS=Citrobacter amalonaticus OX=35703 GN=silA SS=EMBLWGS:MBE0397316 PC=UP000642697:Unassembled WGS sequenceMIEWIIRRSVANRFLVMMGALFLSIWGTWTIINTPVDALPDLSDVQVIIKTSYPGQAPQIVENQVTYPLTTTMLSVPGAKTVRGFSQFGDSYVYVIFEDGTDLYWARSRVLEYLNQVQGKLPAGVSSEIGPDATGVGWIFEYALVDRNGKHDLSELRSLQDWFLKFELKTIPNVAEVASVGGVVKQYQIQVNPVKLSQYGISLPEVKQALESSNQEAGGSSVEMAEAEYMVRASGYLQSIDDFNNIVLKTGENGVPVYLRDVARVQTGPEMRRGIAELNGQGEVAGGVVILRSGKNARDVITAVRDKLETLKASLPEGVEIVTTYDRSQLIDRAIDNLSSKLLEEFIVVAIVCALFLWHVRSALVAIISLPLGLCIAFIVMHFQGLNANIMSLGGIAIAVGAMVDAAIVMIENAHKRLEEWDHQHPGEQIDNATRWKVITDASVEVGPALFISLLIITLSFIPIFTLEGQEGRLFGPLAFTKTYSMAGAAALAIIVIPILMGFWIRGKIPAETSNPLNRVLIKAYHPLLLRVLHWPKTTLLVAALSIFTVIWPLSQVGGEFLPKINEGDLLYMPSTLPGVSPAEAAALLQTTDKLIKSVPEVASVFGKTGKAETATDSAPLEMVETTIQLKPEDQWRPGMTIDKIIDELDRTVRLPGLANLWVPPIRNRIDMLSTGIKSPIGIKVSGTVLSDIDATAQSIEAVAKTVPGVVSVLAERLEGGRYIDIDINREKASRYGMTVGDVQLFVSSAIGGAMVGETVEGVARYPINIRYPQDYRNSPQALKQMPILTPMKQQITLGDVADINVVSGPTMLKTENARPASWIYVDARGRDMVSVVNDIKTAISEKVKLRPGTSVAFSGQFELLEHANKKLKLMVPMTVMIIFILLYLAFRRVDEALLILMSLPFALVGGIWFLYWQGFHMSVATGTGFIALAGVAAEFGVVMLMYLRHAIEAHPELSRKETFTPEGLDEALYHGAVLRVRPKAMTVAVIIAGLLPILWGTGAGSEVMSRIAAPMIGGMITAPLLSLFIIPAAYKLIWLRRHKKSVS>UPI0002505AC5 DUF2724 domain-containing protein OS=Citrobacter amalonaticus OX=35703 GN=ILP74_23360 SS=EMBLWGS:MBE0398275 PC=UP000642697:Unassembled WGS sequenceMNDNAPSLASLLKHGCQVTHYRNTRGWIECPDGRFFKPEPNKVRFIKGMSKPFVYTKKINKGLFSALARLFKKLL>UPI0002505BC9 Phage major tail tube protein OS=Citrobacter amalonaticus OX=35703 GN=ILP74_23530 SS=EMBLWGS:MBE0398308 PC=UP000642697:Unassembled WGS sequenceMAVPHKLRLFTCFVNGSNCIGKVSSVTLPKLTRKTEDFQGGGMIGSAAVDLGLDSGALDTTMVVGGLVQSLLLNYCGDIDETRFRFAGEYYTDGESLLVEVELRGRITEMDGGESKQGEDTSVSYTMKNTYYKLTIDDKPLFEFDLLNFIYKKDGKNIYPDRITSALGMGN>UPI0002511058 Uncharacterized protein OS=Citrobacter amalonaticus OX=35703 GN=ILP74_19985 SS=EMBLWGS:MBE0397640 PC=UP000642697:Unassembled WGS sequenceMREPISLDQAEYKSALAASLFETILEKACAECSETLLNHISLACDLNQEIHRALIAELGMGDAK>UPI00025518C7 M15 family metallopeptidase OS=Citrobacter amalonaticus OX=35703 GN=ILP74_13785 SS=EMBLWGS:MBE0396489 PC=UP000642697:Unassembled WGS sequenceMTLSEKQQLFTVMVASLIHWAEEHGYRLTFGEAYRTPEQAALNAKKGSGISNSLHTQRLAVDFNLFVNGQYKTRTEDYLPLGEYWESLGGAWGGRFKSRPDGNHFSLEHNGVR>UPI00025CADA2 Phage portal protein OS=Citrobacter amalonaticus OX=35703 GN=ILP74_13845 SS=EMBLWGS:MBE0396501 PC=UP000642697:Unassembled WGS sequenceMSKKKRFVKRDQRGDKSKKMSIITFGKPEPVLTTGTDYRDIWYDNAADHFTQPIDRLALAQLINLNGQHGGIIHARKNMIVSDYQGGGLIHDQLEAAAFDYITFGDIAIAKIRNGWGDVIALEPLPGLYIRRRKVRDNALDKPGDYVVLQEGEPQVWPEEDIIFIKMYDPQQHIYGLPDYIGGVHSALLNSEAVIFRRRYYHNGAHTGGILYTRDPSMTDEMEEEIEQQLRDSKGIGNFSTILVNIPGGDGDAIKFIEMGDISAKDEFANIKNISAQDILNAHRFPAGLAGIVPQNTAGLGDVEKAERIYKKSEIAPIQRRFMLAVNNDPEIPERLHLNFDLSYTESTDKGAA>UPI000260ACFC DUF134 domain-containing protein OS=Citrobacter amalonaticus OX=35703 GN=ILP74_01990|ILP74_18185 SS=EMBLWGS:MBE0394284|EMBLWGS:MBE0397299 PC=UP000642697:Unassembled WGS sequenceMPRPRIPRNICGRPADTCFKPNARPMSQLEHVHLKEDEFEALRLVDLLGMQQQEAAVAMGVSRQTLANVLKAARFKVVDCLTQGKALIMHSEREGVTQDDHSHSSE>UPI000260ACFD Dinitrogenase iron-molybdenum cofactor OS=Citrobacter amalonaticus OX=35703 GN=ILP74_01995|ILP74_18180 SS=EMBLWGS:MBE0394285|EMBLWGS:MBE0397298 PC=UP000642697:Unassembled WGS sequenceMITAIPVNDDRVANHFTKASHLVLVDERGVELSRTENPALGADCSGKRKLVDLLVQQQVSRVVVRNIGERMLGKLLGHQIAVYQTDCGRRLFTELCDPDTRVLTELNKPEQGRQSFHHEAKGKKCCHSDGNTAENACQGGRQHHHGNGRCCHS>UPI00026735A2 NADH-quinone oxidoreductase subunit NuoI OS=Citrobacter amalonaticus OX=35703 GN=nuoI SS=EMBLWGS:MBE0394939 PC=UP000642697:Unassembled WGS sequenceMTLKELLVGFGTQVRSIWMIGLHAFAKRETQMYPEEPVYLPPRFRGRIVLTRDPDGEERCVACNLCAVACPVGCISLQKAETKDGRWYPEFFRINFSRCIFCGLCEEACPTTAIQLTPDFELGEYKRQDLVYEKEDLLISGPGKYPEYNFYRMAGMAIDGKDKGEAENEAKPIDVKSLLP>UPI0002674591 F0F1 ATP synthase subunit gamma OS=Citrobacter amalonaticus OX=35703 GN=atpG SS=EMBLWGS:MBE0398094 PC=UP000642697:Unassembled WGS sequenceMAGAKEIRSKIASVQNTQKITKAMEMVAASKMRKSQDRMAASRPYAETMRKVIGHLANGNLEYKHPYLEERDVKRVGYLVVSTDRGLCGGLNINLFKKLLADMKTWSDKGVQCDIAMIGSKGVSFFNSVGGNVVAQVTGMGDNPSLSELIGPVKVMLQAYDEGRLDRLYVVSNKFINTMSQVPTLTQLLPLPASEDEELKQKAWDYLYEPDPKPLLDTLLRRYVESQVYQGVVENLASEQAARMVAMKAATDNGGSLIKELQLVYNKARQASITQELTEIVGGASAV>UPI000272884C Chaperonin GroEL OS=Citrobacter amalonaticus OX=35703 GN=groL SS=EMBLWGS:MBE0394029 PC=UP000642697:Unassembled WGS sequenceMAAKDVKFGNDARVKMLRGVNVLADAVKVTLGPKGRNVVLDKSFGAPTITKDGVSVAREIELEDKFENMGAQMVKEVASKANDAAGDGTTTATVLAQSIITEGLKAVAAGMNPMDLKRGIDKAVAAAVEELKALSVPCSDSKAIAQVGTISANSDETVGKLIAEAMDKVGKEGVITVEDGTGLQDELDVVEGMQFDRGYLSPYFINKPETGAVELESPFILLADKKISNIREMLPVLEAVAKAGKPLLIIAEDVEGEALATLVVNTMRGIVKVAAVKAPGFGDRRKAMLQDIATLTGGTVISEEIGMELEKATLEDLGQAKRVVINKDTTTIIDGVGDEAAIQGRVTQIRQQIEEATSDYDREKLQERVAKLAGGVAVIKVGAATEVEMKEKKARVEDALHATRAAVEEGVVAGGGVALIRVASKIAGLKGQNEDQNVGIKVALRAMESPLRQIVLNCGEEPSVVANTVKAGDGNYGYNAATEEYGNMIDMGILDPTKVTRSALQYAASVAGLMITTECMVTDLPKGDAPDLGAAGGMGGMGGMGGMM>UPI000272897B Phage holin family protein OS=Citrobacter amalonaticus OX=35703 GN=ILP74_04930 SS=EMBLWGS:MBE0394825 PC=UP000642697:Unassembled WGS sequenceMSDPVSGTTVAAGGLMGASVFGLATGIDYGVVFGAFAGAVFYVATAANITRVRLIAYFMTSFIVGVLAAGLVGSKLSQATGYSDRPLDALGAVVVAAMTIKVLTFFNSQDLGSLFSILSRFRGGGASNGNK>UPI000272A6DD Cu(+)/Ag(+) efflux RND transporter outer membrane channel SilC OS=Citrobacter amalonaticus OX=35703 GN=silC SS=EMBLWGS:MBE0397319 PC=UP000642697:Unassembled WGS sequenceMFKLKLLSISTIFILAGCVSLAPEYQRPAAPVPQQFSLSHNSLTPAVNGYQDTGWRNFFVDPQVTRLIGEALTNNRDLRMAALKVEEARAQFNVTDADRYPQLNASSGITYSGGLKGDKPTTQEYDARLELSYELDFFGKLKNMSDADRQNYFASEEARRAVHILLVSNVSQSYFSQQLAYEQLRIARETLKNYEQSYAFVEQQLVTGSTNVLALEQARGQIESTRAEIAKREGDLAQANNALQLVLGTYRALPSEKGMKGGEIAPVKLPPNLSSQILLQRPDIMEAEYQLKAADANIGAARAAFFPSITLTSGLSASSTELSSLFTSGSGMWNFIPKIEIPIFNAGRNKANLKLAEIRQQQSVVNYEQKIQSAFKDVSDTLALRDSLSQQLESQQRYLDSLQITLQRARGLYASGAVSYIEVLDAERSLFATQQTILDLTYSRQVNEINLFTALGGGWVE>UPI00028307B8 AlpA family transcriptional regulator OS=Citrobacter amalonaticus OX=35703 GN=ILP74_21850 SS=EMBLWGS:MBE0397998 PC=UP000642697:Unassembled WGS sequenceMHTAFSSPSSAPAAPLMPVSDAVHERFIRLPEVMHLCGLSRSTIYDLISREAFPKQISLGGKNVAWAQSEITTWMADRIAERNRGYDA>UPI000299C11A Uncharacterized protein OS=Citrobacter amalonaticus OX=35703 GN=ILP74_06005 SS=EMBLWGS:MBE0395032 PC=UP000642697:Unassembled WGS sequenceMSNDRMTVVPDFLGELDAGVFMNKIAAALNTTALGVLNNGNKGKVVLTFDFERMGNSVEEKRVKIKHKLNYSTPTPRGKASEEDTTETPMWVNKGGKLTILQEDQGQLFGITGAVDGKLKAAQ>UPI0002A2E134 Toxin OS=Citrobacter amalonaticus OX=35703 GN=ILP74_02155 SS=EMBLWGS:MBE0394311 PC=UP000642697:Unassembled WGS sequenceMKFLPTTNMRAAKPCLSPVTIWQMLLSRLLEQHYGLTLNDTPFCDETVIQEHIDAGITLANAINFLVEKYELVRIDRRGFSWQEQTPYLTIIDIMRARRDLGLMNRN>UPI0002A2F086 Uncharacterized protein OS=Citrobacter amalonaticus OX=35703 GN=ILP74_02110 SS=EMBLWGS:MBE0394304 PC=UP000642697:Unassembled WGS sequenceMKLLTTAALVASLCIISAPPVLAENIVGSVRTWKSMQADGWKSADGYDDNHMHNALYQAVVLDNYPWTNHFFLRSRDGGSLYLADKKSLTVRYIDIKPRDEYPLHLRIIYQGNNEGQGCYFAVVDDRMANYWTAVEDKLLYQYPAVENVDEKAAELKKQFTPEVMMMVKDNCVNKRQQAALAARRTEKDRELQQWVAQQSLAELCRRTGNC>UPI0002A728B9 50S ribosomal protein L24 OS=Citrobacter amalonaticus OX=35703 GN=rplX SS=EMBLWGS:MBE0397686 PC=UP000642697:Unassembled WGS sequenceMAAKIRRDDEVIVLTGKDKGKRGKVKNVLSSGKVIVEGINLVKKHQKPVPALNQPGGIVEKEAAIQISNIAIFNAATGKADRVGFRFEDGKKVRFFKSNSETIK>UPI0002B611AB Thiol peroxidase OS=Citrobacter amalonaticus OX=35703 GN=tpx SS=EMBLWGS:MBE0395761 PC=UP000642697:Unassembled WGS sequenceMSQTVHFQGNPVAVANSIPQAGSKAQPFTLVAKDLSDVALSQFAGKRKVLNIFPSIDTGVCAASVRKFNQLATEIDNTVVLCVSADLPFAQSRFCGAEGLSNVITLSTLRNAEFLKNYGVEITEGPLKGLAARAVVVLDENDNVIFSQLVNEITNEPDYAAALEVLKA>UPI0002B6140F Flagellar motor switch protein FliN OS=Citrobacter amalonaticus OX=35703 GN=fliN SS=EMBLWGS:MBE0395385 PC=UP000642697:Unassembled WGS sequenceMSDMNNPSDENTGALDDLWADALNEQKTTTSKSAADAVFQQLGGGDVSGTLQDIDLIMDIPVKLTVELGRTRMTIKELLRLTQGSVVALDGLAGEPLDILINGYLIAQGEVVVVADKYGVRITDIITPSERMRRLSR>UPI0002B6147D Envelope stress response membrane protein PspB OS=Citrobacter amalonaticus OX=35703 GN=pspB SS=EMBLWGS:MBE0395743 PC=UP000642697:Unassembled WGS sequenceMSALFLAIPLTIFVLFVLPIWLWLHYSNRSSRGELSQSEQQRLVQLTDDAQRMRERIQALEDILDAEHPNWRDR>UPI0002B61652 Holliday junction branch migration protein RuvA OS=Citrobacter amalonaticus OX=35703 GN=ruvA SS=EMBLWGS:MBE0395538 PC=UP000642697:Unassembled WGS sequenceMIGRLRGIILEKQPPLVLLEVGGVGYEVHMPMTCFYELPESGKEAVVFTHFVVREDAQLLYGFNNKQERTLFKELIKTNGVGPKLALAILSGMSAQQFVNAVEREELGALVKLPGIGKKTAERLIVEMKDRFKGLHGDLFTPAADLVLTSPASPATDDAEQEAVAALVALGYKPQEASRMVSKIARPDASSETLIREALRAAL>UPI0002B61693 Propanediol dehydratase large subunit PduC OS=Citrobacter amalonaticus OX=35703 GN=pduC SS=EMBLWGS:MBE0395285 PC=UP000642697:Unassembled WGS sequenceMRSKRFEALAKRPVNQDGFVKEWIEEGFIAMESPNDPKPSIKIVNGAVTELDGKSVSQFDLIDHFIARYGINLARAEEVMAMDSVKLANMLCDPNVKRRDIVPLTTAMTPAKIVEVVSHMNVVEMMMAMQKMRARRTPSQQAHVTNVKDNPVQIAADAAEGAWRGFDEQETTVAVARYAPFNAIALLVGSQVGRPGVLTQCSLEEATELKLGMLGHTCYAETISVYGTEPVFTDGDDTPWSKGFLASSYASRGLKMRFTSGSGSEVQMGYAEGKSMLYLEARCIYITKAAGVQGLQNGSVSCIGVPSAVPSGIRAVLAENLICSSLDLECASSNDQTFTHSDMRRTARLLMQFLPGTDFISSGYSAVPNYDNMFAGSNEDAEDFDDYNVIQRDLKVDGGLRPVREEDVIAIRNKAARALQAVFAGMGLPPITDEEVEAATYAHGSKDMPERNIVEDIKFAQEIINKNRNGLEVVKALAQGGFTDVAQDMLNIQKAKLTGDYLHTSAIIVGDGQVLSAVNDVNDYAGPATGYRLQGERWEEIKNIPGALDPNELG>UPI0002B616D2 Flagellar hook-basal body complex protein FliE OS=Citrobacter amalonaticus OX=35703 GN=fliE SS=EMBLWGS:MBE0395394 PC=UP000642697:Unassembled WGS sequenceMSAIQGIEGVISQLQATAMSARAQETLPQPTVSFAGQLHAALDRISDTQTAARVQAEKFTLGEPGIALNDVMTDMQKASVSMQMGIQVRNKLVSAYQEVMSMQV>UPI0002B616ED YodD family peroxide/acid resistance protein OS=Citrobacter amalonaticus OX=35703 GN=yodD SS=EMBLWGS:MBE0395379 PC=UP000642697:Unassembled WGS sequenceMKTAKEYSDTAKREVSVDVDALLAAINEISESEIHRSGNDPDRVSVDGREYHTWHELAEAFELDIHDFSVTEINR>UPI0002B6173B Cystine ABC transporter permease OS=Citrobacter amalonaticus OX=35703 GN=tcyL SS=EMBLWGS:MBE0395474 PC=UP000642697:Unassembled WGS sequenceMQESIQLVIDSLPYLLKGAVFTLQLSIGGMFFGLLLGFILALMRLSPLLPLRWLARFYISIFRGTPLIAQLFMIYYGLPQFGIELDPVPSAMIGLSLNTAAYAAETLRAAISSIDKGQWEAAASIGMTPWQTMRRAILPQAARVALPPLSNSFISLVKDTSLAATIQVPELFRQAQLITSRTLEVFTMYLAASLIYWIMATVLSSLQNYFENQLNRQEREPK>UPI0002B6173C Cobalt ECF transporter S component CbiM OS=Citrobacter amalonaticus OX=35703 GN=cbiM SS=EMBLWGS:MBE0395302 PC=UP000642697:Unassembled WGS sequenceMKLEQQLKQLSFSGLAAALLLMIVPEQAFAMHIMEGFLPPMWALAWWLLFLPCLWYGLVRLRHIVMDDSHQKVLLALCGAFIFVLSALKIPSVTGSCSHPTGVGLAVILFGPGVVAVLGAIVLLFQALLLAHGGLTTLGANGMSMAVIGPVVGYMVWKMACRAGIRRDVGVFLCAMLADLVTYFVTSVQLGVAFPDPEAGATGSVVKFMGIFCLTQIPIAIAEGLLTVMIYDQLTKRQLITAQGH>UPI0002B617B4 Orphan toxin OrtT OS=Citrobacter amalonaticus OX=35703 GN=ortT SS=EMBLWGS:MBE0395851 PC=UP000642697:Unassembled WGS sequenceMSLYQKMLMFYAVMAAIAFLITWFLSHDKKRIRFLSAFLVGATWPMSFPVALLFSLF>UPI0002B6195F Propanediol utilization microcompartment protein PduU OS=Citrobacter amalonaticus OX=35703 GN=pduU SS=EMBLWGS:MBE0395270 PC=UP000642697:Unassembled WGS sequenceMERQPTTDRMIQEYVPGKQVTLAHLIANPGKDLFKKLGLQDSVSAIGILTITPSEASIIACDIATKSGAVEIGFLDRFTGAVVLTGDVSAVEYALKQVTRTLGEMMRFTTCSITRT>UPI0002B61BAB Propanediol dehydratase medium subunit PduD OS=Citrobacter amalonaticus OX=35703 GN=pduD SS=EMBLWGS:MBE0395284 PC=UP000642697:Unassembled WGS sequenceMEINEKLLRQIIEDVLSEMQTSDKPVSFRAPGTSTAPAAPAGDSFLTEIGEAKQGTQQDEVIIAVGPAFGLSQTVNIVGLPHKNILREVIAGIEEEGIKARVIRCFKSSDVAFVAVEGNRLSGSGISIGIQSKGTTVIHQQGLPPLSNLELFPQAPLLTLETYRQIGKNAARYAKRESPQPVPTLNDQMARPKYQAKSAILHIKETKYVVTGKNPQELRVAL>UPI0002B61C5E YciY family protein OS=Citrobacter amalonaticus OX=35703 GN=ILP74_09560 SS=EMBLWGS:MBE0395695 PC=UP000642697:Unassembled WGS sequenceMKRSRTEVGRWRMLRQASRRKARWLEGQSRRNMRIHSIRKCVLNQQRNSLLFAIHSL>UPI0002B61D3B Propanediol utilization microcompartment protein PduJ OS=Citrobacter amalonaticus OX=35703 GN=pduJ SS=EMBLWGS:MBE0395280 PC=UP000642697:Unassembled WGS sequenceMNNALGLVETKGLVGAIEAADAMVKSANVQLVGYEKIGSGLVTVMVRGDVGAVKAAVDAGCAAASVVGEVKSSHVIPRPHSDVEAILPKSA>UPI0002B61DDA DUF2594 family protein OS=Citrobacter amalonaticus OX=35703 GN=ILP74_08400 SS=EMBLWGS:MBE0395477 PC=UP000642697:Unassembled WGS sequenceMSTPDFSTAENNQELANEVTCLKALLTLMLQAMGQADAGRVILKMEKQIALVEDDAQAAVFSSTVKQIKQAYRQ>UPI0002B61E29 DUF333 domain-containing protein OS=Citrobacter amalonaticus OX=35703 GN=ILP74_10090 SS=EMBLWGS:MBE0395796 PC=UP000642697:Unassembled WGS sequenceMRAAFWVGCAALLLSACSSDNEPVQQATAAHVAPGLKASMTSSGEANCAMIGGSLSVARQLDGSAIGMCALPNGKRCSEQSLAAGSCGSY>UPI0002B61EC5 HigA family addiction module antidote protein OS=Citrobacter amalonaticus OX=35703 GN=ILP74_10605 SS=EMBLWGS:MBE0395895 PC=UP000642697:Unassembled WGS sequenceMKMANHPRPGDIIQEALDELNVSLREFARAMEIAPSTASRLLTGKAALTPEMAIKLSVVIGSSPEMWLNLQNTWSLAQAQKSVDVSRLRRLAVQ>UPI0002B61F26 DNA-binding transcriptional regulator H-NS OS=Citrobacter amalonaticus OX=35703 GN=hns SS=EMBLWGS:MBE0395683 PC=UP000642697:Unassembled WGS sequenceMSEALKILNNIRTLRAQARECTLETLEEMLEKLEVVVNERREEESAAAAEVEERTRKLQQYREMLIADGIDPNELLNSMAAVKSGTKAKRAARPAKYSYVDENGETKTWTGQGRTPAVIKKAMEEQGKQLDDFLIKE>UPI0002B621C0 Flagellar motor switch protein FliM OS=Citrobacter amalonaticus OX=35703 GN=fliM SS=EMBLWGS:MBE0395386 PC=UP000642697:Unassembled WGS sequenceMGDSILSQAEIDALLNGDSDTKDEPTPGIAGESEIRPYDPNTQRRVVRERLQALEIINERFARQFRMGLFNLLRRSPDITVGAIRIQPYHEFARNLPVPTNLNLIHLKPLRGTGLVVFSPSLVFIAVDNLFGGDGRFPTKVEGREFTHTEQRVINRMLKLALEGYSDAWKAINPLEVEYVRSEMQVKFTNITTSPNDIVVNTPFHVEIGNLTGEFNICLPFSMIEPLRELLVNPPLENSRNEDQNWRDNLVRQVQHSELELVANFADIPLRLSQILKLQPGDVLPIEKPDRIIAHVDGVPVLTSQYGTVNGQYALRVEHLINPILNSLNEEQPK>UPI0002B621C9 CDP-diacylglycerol--glycerol-3-phosphate 3-phosphatidyltransferase OS=Citrobacter amalonaticus OX=35703 GN=pgsA SS=EMBLWGS:MBE0395480 PC=UP000642697:Unassembled WGS sequenceMQFNIPTLLTLFRVILIPFFVVVFYLPFTWAPFVAALIFCIAAVTDWFDGFLARRWNQSTRFGAFLDPVADKVLVAIAMVLVTEHYHSWWVTLPAATMIAREIIISALREWMAELGKRSSVAVSWIGKVKTTSQMAALAWLLWRPNIWVEYAGIALFFVAAVLTLWSMFQYLNAARGDLLDQ>UPI0002B6242F Osmotically-inducible lipoprotein OsmB OS=Citrobacter amalonaticus OX=35703 GN=osmB SS=EMBLWGS:MBE0395729 PC=UP000642697:Unassembled WGS sequenceMFVTSKKMTAAVLAIALAMSLSACSNWSKRDRNTAIGAGAGALGGAVLTDGSTLGTLGGAAVGGIIGHQVGK>UPI0002E6D3A4 TIGR03758 family integrating conjugative element protein OS=Citrobacter amalonaticus OX=35703 GN=ILP74_18440 SS=EMBLWGS:MBE0397346 PC=UP000642697:Unassembled WGS sequenceMPMTSEQTNAFKAGSGSLDVNILHLLCIGALLAFLFLWAAWALSDVWTGWSNTKVRDAALGRFAVRTVLLLLVCIWMFAS>UPI0002F1C917 YbdD/YjiX family protein OS=Citrobacter amalonaticus OX=35703 GN=ILP74_01380 SS=EMBLWGS:MBE0394174 PC=UP000642697:Unassembled WGS sequenceMFGNLGQAKKYLGQAAKMLIGIPDYDNYVEHMQTNHPDKPYMSYEEFFRERQQARYGGDGKGGMRCC>UPI0002FB2594 DUF2786 domain-containing protein OS=Citrobacter amalonaticus OX=35703 GN=ILP74_18555 SS=EMBLWGS:MBE0397369 PC=UP000642697:Unassembled WGS sequenceMNNSQRQLRLLNLVRKLLKLGRSNSNAHEAGLALQRAQKLMARYGISELDAGLTSVREASSRTAPSDAEKVPEWMVTLVRGVCHAFGCQAYYSWRQTSAGYRRSVTFYGFSEKPEIAAYAFDVLTRQLKDATNSYLKTQSKRLKLATRRARAEQFRDGWVCGVREVISATDISSEEQQVMSHWLESRSMKTVTTRELKACRGADTARYQGYEAGQNARLHQGVSGRGPAAIGYRQD>UPI0003060187 Uncharacterized protein OS=Citrobacter amalonaticus OX=35703 GN=ILP74_17760 SS=EMBLWGS:MBE0397221 PC=UP000642697:Unassembled WGS sequenceMTKVRNCVLDALSINVNNIISLVVGSFPLDPTVSKTAVILTILTAT>UPI00031598A7 ParA family protein OS=Citrobacter amalonaticus OX=35703 GN=ILP74_18570 SS=EMBLWGS:MBE0397370 PC=UP000642697:Unassembled WGS sequenceMKILPVISPKGGEGKSTFAAYLAGFLADAGLNTLLVDADYSQPTASSIFALEHESPFGLYELLMQMVSDHTQCISQTAIKNLDVIYSNDPDELLPTAMLHAADGRLRLRNILQHPFFNRYDVIIVDSKGATGVMTELSLLSSTGNVMGVVKPILPDVREFIRGSLHMLTRLKTYENYGIRLPDISILVNCIENTLLDREAMDGLAAIINEKHYDASALGNRDVYRLLDTRIEALDIFKLGHVKQQPVHRLEYKTRRKGPAAAVTMHDLASELFPEWQSHFSDVLTREVRHV>UPI00031DE9EA Uncharacterized protein OS=Citrobacter amalonaticus OX=35703 GN=ILP74_18410 SS=EMBLWGS:MBE0397340 PC=UP000642697:Unassembled WGS sequenceMAGQITFFPLSSVCWTFSDGAGWHIDVIPASSAVLLLRPVALLPAGAPLLRWRDGQLLLDIGRFSLPLSARECAVSWRSPKIPRRLYCPRMTLRELGHLLNVSAFVFICGGYDPAASQRRYR>UPI000326D812 TIGR03751 family conjugal transfer lipoprotein OS=Citrobacter amalonaticus OX=35703 GN=ILP74_18405 SS=EMBLWGS:MBE0397339 PC=UP000642697:Unassembled WGS sequenceMYRTVFILALSCVMLSGCSTSKEEMLPAGDNTMLELWNGADGGGSTSRQSAAARDTLRRPLTGSETQADAQADRSYSRTQESEITQQFPRLPNPDMVMYLYPHLADGNTPVPGYSTVFPFYSQTQYAMPGERTEAL>UPI000326E963 Transglycosylase SLT domain-containing protein OS=Citrobacter amalonaticus OX=35703 GN=ILP74_18480 SS=EMBLWGS:MBE0397354 PC=UP000642697:Unassembled WGS sequenceMGNRSILTGALALGLLMAAVPDGHADQTVPEGYVRVAMAHGVPPEALYSVSLSESSRKLPRGIRPWPWTINVAGKGYRYETRLQAWQALQVFMKRHPLKRIDVGIAQVNLGWNGHHFASTWDAFDPYTNLNAAATILRECWARKPGSWLDAAGCYHHPAGGQPAARYRAIVRGHLAKISPAPRISAPAAEAPRSVAALTPDPGFVWTEPGR>UPI000326F92C Integrase domain-containing protein OS=Citrobacter amalonaticus OX=35703 GN=ILP74_18335 SS=EMBLWGS:MBE0397325 PC=UP000642697:Unassembled WGS sequenceMSRFSKQLCKQLVTLARQGRGSYKTVADRSRIAERFSERLSELNIQIRDVKHIKTSHIEKYIESRKADSLSLRTLQNEMSAIRSVLLSAGRNKLADPNHTSLSNQALGISGANRDGTKLPITDEKLNAVVNFALKKDEGVALAVQLSRYLGLRTEETVQSAKSLKTWKQSLINNNERVRVVFGTKGGRPRETTVFNREKVLSILDKAIHYVSEHNGKLIDKPSLHTAIDRYRNIVREAGMNGKNAPHSLRYAYSRDAVNHHIKNGMSRDEAEALVSMDLGHGDGRGRYIKQVYFRGETE>UPI0003270826 TIGR03745 family integrating conjugative element membrane protein OS=Citrobacter amalonaticus OX=35703 GN=ILP74_18435 SS=EMBLWGS:MBE0397345 PC=UP000642697:Unassembled WGS sequenceMHHSESVVARLRRLASRTLTRAGTLALLGWLTCKPAFADLPSVEAPESGGGSGLSGQIKGYLQDGIVIGGLVVAAVAFINVAIAALHTFTEVRNEKATWTKFGAIVVVGVVLLVAVIWLLGKSADILL>UPI0003270962 Uncharacterized protein OS=Citrobacter amalonaticus OX=35703 GN=ILP74_18130 SS=EMBLWGS:MBE0397288 PC=UP000642697:Unassembled WGS sequenceMHTDVYTLKTPLDTLSWLCLLESELLSIRAFQRLDLHTDRDEPNELTFLEDSIIGTGTAYGWFVFLLGEGDIPPLPDTSKNLLFTLDELGKEINRPFWEKAVDEGIQDARCDRAIAALERM>UPI00032713E4 Copper resistance membrane spanning protein PcoS OS=Citrobacter amalonaticus OX=35703 GN=pcoS SS=EMBLWGS:MBE0397306 PC=UP000642697:Unassembled WGS sequenceMRFKISLTTRLSLIFSAVMLTVWWLSSFILISTLNGYFDNQDRDFLTGKLQLTEEFLKTETFRNKTDIKSLSEKINDAMVGHNGLFISIKNMENEKIVELYAKNSVVPAVLLNKSGDILDYMIQTEENNTVYRSISRRVAVTPEQGKSKHVIITVATDTGYHTLFMDKLSTWLFWFNIGLVFISVFLGWLTTRIGLKPLREMTSLASSMTVHSLDQRLNPDLAPPEISETMQEFNNMFDRLEGAFRKLSDFSSDIAHELRTPVSNLMMQTQFALAKERDVSHYREILFANLEELKRLSRMTSDMLFLARSEHGLLRLDKHDVDLAAELNELRELFEPLADETGKTITVEGEGVVAGDSDMLRRAFSNLLSNAIKYSPDNTCTAIHLERDSDCVNVMITNTISGQVPANLERLFDRFYRADSSRFYNTEGAGLGLSITRSIIHAHGGELSAEQQGREIVFSVRLLMD>UPI0003355E03 PaaI family thioesterase OS=Citrobacter amalonaticus OX=35703 GN=ILP74_18175|ILP74_02000 SS=EMBLWGS:MBE0397297|EMBLWGS:MBE0394286 PC=UP000642697:Unassembled WGS sequenceMNCYLDARKAHTCCMVCSSRDNNPDTVNLMFSEHPDGSVCADYTANHRHQGYTGLLHGGMTSTLLDAAMTHCLFMQGVQALTAELTVRFISPVCTGDKLMVCARLLGQRRGIYLLEAWLTKGQQTVARATAKFIVPSQDIALAHR>UPI0003590EE1 Uncharacterized protein OS=Citrobacter amalonaticus OX=35703 GN=ILP74_18450 SS=EMBLWGS:MBE0397348 PC=UP000642697:Unassembled WGS sequenceMMKTVLKATLITALITGPVLAADGTGKVQLSDLHFATAANGLQQIEGTGTNVSGGPVKTVIVKFNLLQNGAVIGNTAAMAENLEPGQQWKLQAPYDSITNKPDSFKVTELTVFNN>UPI0003B5BFAF ABC transporter ATP-binding protein OS=Citrobacter amalonaticus OX=35703 GN=ILP74_18195 SS=EMBLWGS:MBE0397301 PC=UP000642697:Unassembled WGS sequenceMSSKGIHIESLSKRYGDGDTAVFALRDVNMHVAPGEVVGLIGPSGSGKSTLLKCLGAVIEPTAGRMTLGDEVIYADGWKVRDLRALRRDKIGFVFQAPYLIPFLDVTDNVALLPMLAGVANGESRAKALELLTALDVQHRARAMPSQLSGGEQQRVAIARGLVNRPPVILADEPTAPLDSERAMAVIRILNDMARKFETAIIVVTHDEKIIPTFKRIYHIRDGVTHEEAGEGREFE>UPI0003B674F1 Efflux RND transporter periplasmic adaptor subunit OS=Citrobacter amalonaticus OX=35703 GN=ILP74_18205 SS=EMBLWGS:MBE0397303 PC=UP000642697:Unassembled WGS sequenceMKKLPLQGRTLALLAVIIPLLVLFIYVGLRSGPLAPVAVTVASVESQAITPALFGIGTVEARYTYKIGPTFAGRVKRLEVHVGDQVKAGQVLGEMEPVDLDDRVRSQESVFKRAEAALREAEARQAYAQTQARRYEQLFAVRSTSEEIVTTKRQELQIADAALSAAREDIARARSDREALVAQRSNLRLIAPVDGVVAVRDADPGTTIVAGQAVVEVIDPKSLWINVRFDQISASGLAGGLPAHIVLRSRGGQTLKGRVLRVEPKADAVTEETLAKVTFDNKPEPLPPVGELAEVTVDLPALPAAPLIPNAAVQREGDKVGVWQIVDGDLHFSPVKLGTSDLNGYVQVREGLKNGDQVVTYSEKALTARSRIHVVEHIPGVSR>UPI0003BD0D65 DnaA initiator-associating protein DiaA OS=Citrobacter amalonaticus OX=35703 GN=diaA SS=EMBLWGS:MBE0397519 PC=UP000642697:Unassembled WGS sequenceMLDRIKVCFTESIQTQIAAAEALPDAISRAAMTLVHSLLNGNKILCCGNGTSAANAQHFAASMINRFETERPSLPAIALNTDNVVLTAIANDRLHDEVYAKQVRALGHAGDVLLAISTRGNSRDIVKAVEAAVTRDMTIVALTGYDGGELAGLLGPQDVEIRIPSHHSARIQEMHMLTVNCLCDLIDNTLFPHQDD>UPI0003BE0EBC Uncharacterized protein OS=Citrobacter amalonaticus OX=35703 GN=ILP74_07990 SS=EMBLWGS:MBE0395401 PC=UP000642697:Unassembled WGS sequenceMTPETDNAIRAACRRCTEEIQQAMRKKPKPNWNETVPPIINKHHKKIEALGVSLLEFVVKTGRLNGRFGAEQ>UPI0003BE5867 Phage terminase small subunit P27 family OS=Citrobacter amalonaticus OX=35703 GN=ILP74_19925 SS=EMBLWGS:MBE0397629 PC=UP000642697:Unassembled WGS sequenceMARPPKPPAYLDEIAAQQWKAKAKQLAERGDLTPADWNNLELYCVNYSMYRKAVEDLATRGFSIVNSQGGESRNPALSAKADAEKILIKMSSLLGFDPVSRRRNPVETEEEDELDRLE>UPI0003BE7BEF Phage head closure protein OS=Citrobacter amalonaticus OX=35703 GN=ILP74_19940 SS=EMBLWGS:MBE0397632 PC=UP000642697:Unassembled WGS sequenceMRAGKMKRRVTFQKSESHRDPTGQVIYEWADLATVWAEIRAISGRERMSSGALYSEATVRIWTRYRDDITTANRILYRSPNVRGQVYGIVAVIPDVDHTRLELLCKGGIFNE>UPI0003BED045 50S ribosomal protein L13 OS=Citrobacter amalonaticus OX=35703 GN=ILP74_08250 SS=EMBLWGS:MBE0395447 PC=UP000642697:Unassembled WGS sequenceMTYKSVKHGLPRSFTRVWVMTDTGRETTGYVKSDGEWHINCERIRATGAKVLRWKEG>UPI0003BF340F Phage gp6-like head-tail connector protein OS=Citrobacter amalonaticus OX=35703 GN=ILP74_19935 SS=EMBLWGS:MBE0397631 PC=UP000642697:Unassembled WGS sequenceMSELIGLEEAKLHCRIDDDYEDTLIQAYIDAALEVCQKHIGKRFDNGLEFTPAIKIGCLMYVSQLYEYRTMIGDTDAKEIPMAVSALWSVYRDVGVY>UPI0003EDA0ED Site-specific integrase OS=Citrobacter amalonaticus OX=35703 GN=ILP74_18070 SS=EMBLWGS:MBE0397277 PC=UP000642697:Unassembled WGS sequenceMSDKNHYTTWEELLEEYFFARNLRAATEWSYAKVVKGFLKFTGADITPSMVTHHEVLRWRRYVLREKQQSAQTWNNKIAHLRALYNYAMESGLLPAGKNPFNNCTVQRDRKKKRTLNRSQLTRLYLIMQQAEIESNRKIFARGGRSALYPAWFWITVLDTLRYTGMRQNQLLHIQLRDVNLTEGYIDLRLEGSKTHREWRVPVVKQLRLRLQLLLTRATEAGAGPKDNLFDVSFYIAGKKAKFDRNDVTVMHQKIRSFFRRLSKECGFAVSPHRFRHTLATELMKAPERNLQLVKDLLGHRSVSTTMEYVELKMDIVGKTLEEELSLHTDLCVERELQLLTQN>UPI0004450AF3 DUF945 domain-containing protein OS=Citrobacter amalonaticus OX=35703 GN=ILP74_02130 SS=EMBLWGS:MBE0394307 PC=UP000642697:Unassembled WGS sequenceMRLASRFGRINQIRRDRPLTHEELMSHVPSVFGSDKHESRSDRYTYIPTITILESLQREGFEPFFACQTKVRDQSKREHTKHMLRLRRAGQLTGHQVPEIILLNSHDGSSSYQMLPGLFRGVCTNGLVCGQSFGEVRVPHKGNVVEKVIEGAYEVLGVFDRVEEKRDAMQSLALPAPARHALANAALKYRFGEEHQPVTVSQLLTPRRREDYSDDLWTVYQRVQENLMKGGLSGRTAQGKSSRTRAVTGIDGDVKLNRALWVMAENMLDFFGR>UPI00044AAB77 Uncharacterized protein OS=Citrobacter amalonaticus OX=35703 GN=ILP74_18445 SS=EMBLWGS:MBE0397347 PC=UP000642697:Unassembled WGS sequenceMNSHRPAMRRNRRVPGITGLVLLTFLASAAAQASEKDELASVQRQLDQVQASLERARVAAAQADPADRGRFFFDYRQATSDLNTIRSGIDRYLEPSRAQPRDPSYVAGNYRRERP>UPI00044CF6E0 Uncharacterized protein OS=Citrobacter amalonaticus OX=35703 GN=ILP74_18365 SS=EMBLWGS:MBE0397331 PC=UP000642697:Unassembled WGS sequenceMRNKTTVRKVLTRAGIFLACMALFMVVSLMMADTAMKHPTEAAAFRSWMQSTRYGWLMWRLALYALVAWGLWKIRHAPGFREAYRRPLLRISVVSVLFIALCEYAMFTGPGA>UPI0004537B05 Uncharacterized protein OS=Citrobacter amalonaticus OX=35703 GN=ILP74_18115 SS=EMBLWGS:MBE0397286 PC=UP000642697:Unassembled WGS sequenceMGLDIFLYSQHRQIVAVPAPYHPVCELSRDFSLITWVDKHVGEVENATCLELSREHIQQLKERLDCAIATFTEGNEDNPLLQRRLQYVFTLRDRLESLLSDFDFEHEYLWFYAAW>UPI0004565C69 Copper resistance system metallochaperone PcoE OS=Citrobacter amalonaticus OX=35703 GN=pcoE SS=EMBLWGS:MBE0397305 PC=UP000642697:Unassembled WGS sequenceMKKILVSFIAMMAVASSAWAAETMNMHDQVNNAQAPAHQMQSSAEKSAVQGESMKMMDMSGHDQAAMSHEMMQNGNSAAHQDMAEMHKKMMKSKPAASNETAKSFSEMNEHEKAAVMHEKANNGQSSVIHQQQAEKHRSQITQN>UPI00049EA231 Anaerobic C4-dicarboxylate transporter DcuB OS=Citrobacter amalonaticus OX=35703 GN=dcuB SS=EMBLWGS:MBE0393981 PC=UP000642697:Unassembled WGS sequenceMLFSIQLLIILICLFYGARKGGIALGLLGGIGLVILVFVFHLQPGKPPVDVMLVIIAVVAASATLQASGGLDVMLQIAEKLLRRNPKYVSIVAPFVTCTLTILCGTGHVVYTILPIIYDVAIKNNIRPERPMAASSIGAQMGIIASPVSVAVVSLVAMLGNVTFEGKHLEFLDLLAITIPSTLIGILAIGIFSWFRGKDLDKDEDFQKFISVPENHHYVYGDTATLLDKKLPKSNWLAMWIFLAAIAVVAILGAFSELRPSFGGKALSMVLVIQMFMLLTGALIIILTKTNPASISKNEVFRSGMIAIVAVYGIAWMAETMFGAHMTEIEGVLGNMVKEYPWAYAIVLLLVSKFVNSQAAALAAIVPVALAIGVDPAYIVASAPACYGYYILPTYPSDLAAIQFDRSGTTRIGRFVINHSFILPGLIGVSVSCVFGWIFAAMYGFL>UPI00049EA244 2-iminobutanoate/2-iminopropanoate deaminase OS=Citrobacter amalonaticus OX=35703 GN=ridA SS=EMBLWGS:MBE0394132 PC=UP000642697:Unassembled WGS sequenceMSKTIATENAPAAIGPYVQGVDLGSMIITSGQIPVDPKTGSVSDDVSAQARQSLENVKAIVEAAGLKVGDIVKTTVFVKDLNDFATVNATYEAFFTEHNATFPARSCVEVARLPKDVKIEIEAIAVRR>UPI00049EA4B6 Uncharacterized protein OS=Citrobacter amalonaticus OX=35703 GN=ILP74_04770 SS=EMBLWGS:MBE0394795 PC=UP000642697:Unassembled WGS sequenceMSQQEQFCHACGMPLSAPDAKGASDKYCAYCSDAEGNLKPWDDAVSGLAGFLDSWQKVGPEESRKRAIRYLTSMPAWAHKADD>UPI00049EA523 Uncharacterized protein OS=Citrobacter amalonaticus OX=35703 GN=ILP74_18315 SS=EMBLWGS:MBE0397324 PC=UP000642697:Unassembled WGS sequenceMKKKLLFVTDLYYQAKGRIYYEEDMFLSEKLGQFFDLCLCHPKSATSFIENSNVILFRNTGPVMNFASQYNEFRSKALDTGSRVFNQLSGKADMKGKNYLIELTKAGYPVIPSVDRHKDLKSLPAVDNYIVKLKQGADSLGMNFVSADELSRTELTGRLVQPVIDFVYEVSFYFINHQFQYALYAPNPDERWRLEPYIATPSDLKFSRRFIDWNGIDYGIQRVDACRTKDGDLLLMELEDLNPYLSLELLDDETRDSFVQNLSSALIAYAAT>UPI00049EA5A9 DUF805 domain-containing protein OS=Citrobacter amalonaticus OX=35703 GN=ILP74_23610 SS=EMBLWGS:MBE0398323 PC=UP000642697:Unassembled WGS sequenceMTLQQWLFSFKGRIGRRDFWIWIALWIVSMVVLFSLESQKLLPDQIAPFALVCLLWPTAAVTVKRLHDRGRSGLWALLIILAWMLLAGNWAILPGMWQWVVGRFAPTLILIMTLIDLGAFVGTQGENKYGKDTQDVAFKSAP>UPI00049EA60A PTS glucitol/sorbitol transporter subunit IIC OS=Citrobacter amalonaticus OX=35703 GN=ILP74_05750 SS=EMBLWGS:MBE0394983 PC=UP000642697:Unassembled WGS sequenceMIVEIAEGFIHLFQSAGKIFSGMIVSTIPMLITLILTVNFIMKLIGQQRMEKVATLMGKSKILTYGILPSFAWFFLSSPGALTMGKFLPERCKPAYQDALGSTVHPLTSLFPHIVPSELFIWLGVAAGLTRLDLPVADLALRYIAAGILLGFIRGFLTEYIFTRLEKRELNQE>UPI00049EA60C SirB family protein OS=Citrobacter amalonaticus OX=35703 GN=ILP74_09370 SS=EMBLWGS:MBE0395661 PC=UP000642697:Unassembled WGS sequenceMNSVTLLLGVHLASIALSVSLFVLRYGWSECHSARAHARWTRVVPPIVDTVLLLTGVGLIAKTHILPFTEQGTWLTEKLFGVIIYIVLGFIALDYRRARSRQARLIAFPLALVVLYIIIKLATTKIPLLG>UPI00049EA61F Membrane-bound lytic murein transglycosylase EmtA OS=Citrobacter amalonaticus OX=35703 GN=emtA SS=EMBLWGS:MBE0395626 PC=UP000642697:Unassembled WGS sequenceMKLRWFAFLIVLLAGCSSKQDYKNPPWNAEVPVKRAMQWMPISEKAGAAWGVSPQLITAIIAIESGGNPNAVSKSNAIGLMQLKASTSGRDVYRRMGWSGEPTTSELKNPERNISMGAAYLSILETGPLAGIEDPQVMQYALVVSYANGAGALLRTFSSDRKKAINKINDLSADEFVEHVADNHPAPQAPRYIYKLQRALDAM>UPI00049EA676 Type I toxin-antitoxin system SymE family toxin OS=Citrobacter amalonaticus OX=35703 GN=ILP74_03110 SS=EMBLWGS:MBE0394493 PC=UP000642697:Unassembled WGS sequenceMTIEAGSPEIAIIRPFRRLKVSYVRKRHEDPKTGNTRRYSRHASLTINGDWLEQAGFPTGTLVNVAVMQGKLVIEQVREEQAG>UPI00049EA67D 23S rRNA (Adenine(2030)-N(6))-methyltransferase RlmJ OS=Citrobacter amalonaticus OX=35703 GN=ILP74_21105 SS=EMBLWGS:MBE0397854 PC=UP000642697:Unassembled WGS sequenceMLSYRHSFHAGNHADVLKHTVQSLIIESLKEKEKPFLYLDTHAGAGRYQLSGEHAERTGEYLEGIARIWQQDDLPAELEPYIGVVKHFNRSGQLRYYPGSPLIARQLLREQDSLHMTELHPSDFPLLRGEFQKDERARVARADGYQQLKAKLPPVSRRGLILIDPPYEIKSDYQAVVAGISEGYKRFATGTYALWYPVVLRQQIKRMVHDLEATGIRKILQIELAVRPDSDQRGMTASGMIVVNPPWKLEAQMNTVLPWLHSKLVPAGTGHTLVNWIVPE>UPI00049EA6F7 HAAAP family serine/threonine permease OS=Citrobacter amalonaticus OX=35703 GN=ILP74_19170 SS=EMBLWGS:MBE0397484 PC=UP000642697:Unassembled WGS sequenceMETATNNSVVLDASAPARRAGMTESEWREAIKFDSIDTGWVIMSIGMAIGAGIVFLPVQVGLMGLWVFLLSSVIGYPAMYLFQRLFINTLAESPECKDYPSVISGYLGKNWGILLGALYFVMLVIWMFVYSTAITNDSASYLHTFGVTEGLLSDNPFYGLVLICILVAISSRGEKLLFKISTGMVLTKLLVVAALGVSMVGMWHLYNIGALPPMALLVKNAIITLPFTLTSILFIQTLSPMVISYRSREKSVEVARHKALRAMNIAFGILFVTVFFYAVSFTLAMGHDEAVKAYEQNISALAIAAQFISGAGAGWVKVVSVILNIFAVMTAFFGVYLGFREATQGIAMNILRRKLPAEKINETLVQRGIMVFAILLAWSAIVLNAPVLSFTSICSPIFGMVGCLIPAWLVYKVPALHKYKGVSLYLIIVTGLLLCVSPFLAFS>UPI00049EA72E Two-component system response regulator BaeR OS=Citrobacter amalonaticus OX=35703 GN=baeR SS=EMBLWGS:MBE0395199 PC=UP000642697:Unassembled WGS sequenceMTELPIDENTPRILIVEDEPKLGQLLIDYLRAASYAPTLISHGDQVLSYVRQTPPDLILLDLMLPGTDGLTLCREIRRFSEVPIVMVTAKIEEIDRLLGLEIGADDYICKPYSPREVVARVKTILRRCKPQRELQQLDAESPLIVDESRFQASWRNKMLDLTPAEFRLLKTLSHEPGKVFSREQLLNHLYDDYRVVTDRTIDSHIKNLRRKLEALDEEQSFIRAVYGVGYRWEADACRIV>UPI00049EA805 Peptide ABC transporter permease SapC OS=Citrobacter amalonaticus OX=35703 GN=sapC SS=EMBLWGS:MBE0395738 PC=UP000642697:Unassembled WGS sequenceMPYDSVYSEKRPPGTLRTAWRKFYGDTTAMVGLYGCAGLVVLCLFGNWFAPYGIDQQFLGYQLLPPSWSRYGEVSFFLGTDDLGRDVLSRLLSGAAPTVGGAFVVTLAATLCGLFLGVVAGATHGLRSAVLNHILDTLLSIPSLLLAIIVVAFAGPHLTHAMFAVWLALLPRMVRSVYSMVHDELEKEYVIAARLDGASTLNILWFAILPNITAGLITEITRALSMAILDIAALGFLDLGAQLPSPEWGAMLGDALELIYVAPWTVMLPGAAIMISVLLVNLLGDGVRRAIIAGVE>UPI00049EA83B Flagellar hook-associated protein FlgL OS=Citrobacter amalonaticus OX=35703 GN=flgL SS=EMBLWGS:MBE0396218 PC=UP000642697:Unassembled WGS sequenceMRISTQMMYQQNMRGITNSQAEWMKYGEQMSTGKRVINPSDDPIAASQAVVLSQAQSENSQFALARTFATQKVSLEENVLNQVTTAIQSAQEKIVSAGNGTLSDDDRASLATDLQGIRDQLMNLANSTDGNGRYIFAGYKTETAPFAQADGTYNGGTENITQQVDASRSMTIGHTGNKIFDKITSNAVPEPNNGTSETNLFKMLDSAIASLNSPVGDDATLKEQAKAVIDKTNRGLKNSLNNVLSVRAELGTQLKELESLDSLGTDRALGQTQQMSNLVDVDWNAAISSYVMQQAALQASYKAFTDMQGMSLFQLNR>UPI00049EA868 Acid phosphatase AphA OS=Citrobacter amalonaticus OX=35703 GN=aphA SS=EMBLWGS:MBE0393921 PC=UP000642697:Unassembled WGS sequenceMRKITLALSAICLLFTLNHPASALASSPSPLNPGTNVAKLAEQAPVHWVSVAQIENTLTGRPPMAVGFDIDDTVLFSSPGFWRGKKTWSPDSEAYLKNPAFWEKMNNGWDEFSIPKEVARQLIDMHVRRGDSIFFITGRSPTKTETVSKTLADNFHIPAANMNPVIFAGDKPGQNTKTQWLQDKNIRMFYGDSDNDITAARDAGIRGIRILRASNSTYRPLPQAGAYGEEVIVNSEY>UPI00049EA870 Alanine/ornithine racemase family PLP-dependent enzyme OS=Citrobacter amalonaticus OX=35703 GN=ILP74_01140 SS=EMBLWGS:MBE0394126 PC=UP000642697:Unassembled WGS sequenceMRNQDFPCLFVNLSVIRDNTRTLIALCRQHGVEPVGVNKLSCEAANVARAMIDAGISTIADSRIQNLKKIADLPVDKLLLRLPQISLAHDIVAYADISLNSEEHTLQALSAAAIAQNKTHRVILMHDLGDLREGCVDPLETRRLARLVHKELPGLTLEGLGANLACYGGVEPTTENQQALVDLAHQIEDELGIALRTISGASSAALFLLINGGLPTGVNQLRLGASLIMGFGLNDEPIPDTRQDAIKLGVEIIELKDKPSVPQHSTALDAMGRKPVFEDLGVHHRALAALGEQDVSFSQLRPFDPGVKVLGASSDHLILDVTHAASGYQVGDIVYFSLGYSGVLQCMTSEYVGKQYSYF>UPI00049EAA2D Bifunctional acetaldehyde-CoA/alcohol dehydrogenase OS=Citrobacter amalonaticus OX=35703 GN=adhE SS=EMBLWGS:MBE0395685 PC=UP000642697:Unassembled WGS sequenceMAVTNVAELNALVERVKKAQREYASFTQEQVDKIFRAAALAAADARIPLAKMAVAESGMGIVEDKVIKNHFASEYIYNAYKDEKTCGVLSEDDTFGTITIAEPIGIICGIVPTTNPTSTAIFKSLISLKTRNAIIFSPHPRAKDATNKAADIVLQAAIAAGAPKDLIGWIDQPSVELSNALMHHPDINLILATGGPGMVKAAYSSGKPAIGVGAGNTPVVIDETADIKRAVASVLMSKTFDNGVICASEQSVVVVDSVYDAVRERFASHGGYMLQGKELKAVQDVILKNGALNAAIVGQPAYKIAELAGFTVPETTKILIGEVKVVDESEPFAHEKLSPTLAMYRAKDFEDAVIKAEKLVAMGGIGHTSCLYTDQDNQPERVAYFGQMMKTARILINTPASQGGIGDLYNFKLAPSLTLGCGSWGGNSISENVGPKHLINKKTVAKRAENMLWHKLPKSIYFRRGSLPIALDEVITDGHKRALIVTDRFLFNNGYADQITSVLKAAGVETEVFFEVEADPTLSVVRKGAELANSFKPDVIIALGGGSPMDAAKIMWVMYEHPETHFEELALRFMDIRKRIYKFPKMGVKAKMIAVTTTSGTGSEVTPFAVVTDDATGQKYPLADYALTPDMAIVDANLVMDMPKSLCAFGGLDAVTHALEAYVSVLASEFSDGQALQALKLLKENLPASYNEGSKNPVARERVHSAATIAGIAFANAFLGVCHSMAHKLGSQFHIPHGLANALLISNVIRYNANDNPTKQTAFSQYDRPQARRRYAEIADHLGLSAPGDRTAAKIEKLLAWLESLKAELGIPKSIREAGVQEADFLAHVDKLSEDAFDDQCTGANPRYPLISELKQILLDTFYGREFSEGTPAAKEVAAAPKAEKKAKKSA>UPI00049EAADF Barstar family protein OS=Citrobacter amalonaticus OX=35703 GN=ILP74_19775 SS=EMBLWGS:MBE0397600 PC=UP000642697:Unassembled WGS sequenceMNIYTFDFDEIEDQDDFYREFARMFGLAKEKVSDLDSLWDIIMSEALPLPLEIEFVHLPEKSRRRYGALILLFDEAEEELEGQLRFNVRH>UPI00049EAB02 Succinate dehydrogenase flavoprotein subunit OS=Citrobacter amalonaticus OX=35703 GN=sdhA SS=EMBLWGS:MBE0396629 PC=UP000642697:Unassembled WGS sequenceMKLPVREFDAVVIGAGGAGMRAALQISQSGQTCALLSKVFPTRSHTVSAQGGITVALGNTHEDNWEWHMYDTVKGSDYIGDQDAIEYMCKTGPEAILELEHMGLPFSRLDDGRIYQRPFGGQSKNFGGEQAARTAAAADRTGHALLHTLYQQNLKNHTTIFSEWYALDLVKNADGAVVGCTALCIETGEVVYFKARATVLATGGAGRIYQSTTNAHINTGDGVGMAIRAGVPVQDMEMWQFHPTGIAGAGVLVTEGCRGEGGYLLNKHGERFMERYAPNAKDLAGRDVVARSIMIEIREGRGCDGPWGPHAKLKLDHLGKDVLESRLPGILELSRTFAHVDPVKEPIPVIPTCHYMMGGIPTKVTGQALTVNEQGEDVVVPGLFAVGEIACVSVHGANRLGGNSLLDLVVFGRAAGLHLQESIAEQGELRDATEDEIDASLARLNRWNNNRDGEDPVVIRKALQECMQHNFSVFREGDAMAKGLEELKAIRERLKNARLDDTSSEFNTQRVECLELDNLMETAYATAVSANFRTESRGAHSRFDFPERDDENWLCHSLYLPETESMTRREVNMEPKLRPAFPPKIRTY>UPI00049EAC37 Sulfurtransferase TusA OS=Citrobacter amalonaticus OX=35703 GN=tusA SS=EMBLWGS:MBE0397814 PC=UP000642697:Unassembled WGS sequenceMSDLFSSPDHTLDAQGLRCPEPVMMVRKTVRNMRTGETLLIVADDPATTRDIPGFCTFMEHELVAKETDSLPYRYLVRKGQ>UPI00049EAC58 Biotin transporter OS=Citrobacter amalonaticus OX=35703 GN=bioP SS=EMBLWGS:MBE0398175 PC=UP000642697:Unassembled WGS sequenceMALLIITTILWAFSFSLFGEYLAGHVDSYFAVLVRVGLAALVFLPFLRTRGHSLKTIGLYMLVGAMQLGIMYMLSFHAYLYLTVSELLLFTVLTPLYITLIYDLMSKRRLRWGYAFSALLAVIGAGIIRYDQVTDHFWTGLLLVQLSNISFAIGMVGYKRLMETRPMPQHNAFAWFYIGAFLVAVVAWFMLGNAQKMPETTLQWGILVFLGVAASGIGYFMWNYGATQVDAGTLGIMNNMHVPAGLLVNLAIWHQQPHWPSFITGAMVILASLWVHRKWVAPRSEQTADDRRRDCAPIE>UPI00049EAC83 MHS family MFS transporter OS=Citrobacter amalonaticus OX=35703 GN=ILP74_10140 SS=EMBLWGS:MBE0395806 PC=UP000642697:Unassembled WGS sequenceMTTHESSTVILKKNKKVLVASLTGSAIEWFDYFLYGTAAALVFNKIFFPMVDPVIGLILSYLSFSLTFFIRPIGGVIFAHIGDRIGRKKTLVLTLSLMGGATVMIGLLPTYEMIGIWAPILLILMRIIQGMGIGGEWGGALLLAYEYAPEKRKGFFGSIPQAGVTIGMLMATFIVSLMTLFSEEDFLSWGWRIPFLLSSVLVLLGLWIRRDIDETPDFKKVKATGQVAKAPLRDTLTHHWREVLIAAGLKVVETAPFYIFSTFVVSYATSTLSYQKSQVLEAVTVGALIATVMIPLMGLLSDKIGRKRMYAISVALLGLFIVPWFLLLNTGTTWGIMLATIVMFGVLWAPVTAVLGTLCSEIFSANVRYTGITLGYQIGAALAGGTAPLIATGLLAKYDGDWVPVAWYLLTTVAISLVAIFFASRVKRSPAINAQPNEL>UPI00049EACFF Phosphoribulokinase OS=Citrobacter amalonaticus OX=35703 GN=ILP74_20420 SS=EMBLWGS:MBE0397719 PC=UP000642697:Unassembled WGS sequenceMSAKHPVIAVTGSSGAGTTTTSLAFRKIFAQLNLRAAEVEGDSFHRYTRPEMDMAIRKARDAGRHISYFGPDANDFGLLEQTFIEYGQTGKGQSRKYLHTYDEAVPWNQVPGTFTPWQPLPEPTDVLFYEGLHGGVVTPQHEVARHVDLLVGVVPIVNLEWIQKLIRDTSERGHSREAVMDSVVRSMDDYINYITPQFSRTHINFQRVPTVDTSNPFAAKGIPSLDESFVVIHFRNLEGIDFPWLLAMLQGSFISHINTLVVPGGKMGLAMELIMLPLVQRLMEGKKIE>UPI00049EAEA3 Colanic acid biosynthesis glycosyltransferase WcaE OS=Citrobacter amalonaticus OX=35703 GN=wcaE SS=EMBLWGS:MBE0395220 PC=UP000642697:Unassembled WGS sequenceMLLSIITVAFRNLDGIVKTHASLAHLAKATDIRFEWIVVDGGSNDGTQEFLENLCGNYHLRFVSEPDNGIYDAMNKGIEMARGKFALFLNSGDIFHPDVAHVVRQLQTQKDDVMITGDALLDFGDGHKTKRSAKPGWYIYHSLPASHQAIFFPLAGLKVWHYDLQYKVSSDYALAARLYKEGYDFKKLDGLVSEFSMGGVSTTNNLELCEDAKKVQRQILRVPGFWAELSKLLRLRTTGKTKALYNKA>UPI00049EAFAF Nitrite reductase small subunit NirD OS=Citrobacter amalonaticus OX=35703 GN=nirD SS=EMBLWGS:MBE0397730 PC=UP000642697:Unassembled WGS sequenceMSQWTNICHIDDILPATGVCALLGEEQVAIFRPYHNDQVFAISNIDPFFESSVLSRGLIAEHQGELWVASPLKKQRFRLSDGLCMEDERHSVAHYDARVKDGIVQLRS>UPI00049EAFC7 Phospholipid-binding protein MlaC OS=Citrobacter amalonaticus OX=35703 GN=mlaC SS=EMBLWGS:MBE0397559 PC=UP000642697:Unassembled WGS sequenceMLKRLMMVALLVIAPLSTAIAADQTNPYKLMNEAAQKTFDRLKNEQPKIRSNPDYLRDVVDQELLPYVQVKYAGALVLGRYYKEATPAQREAYFAAFREYLKQAYGQALAMYHGQTYQIAPEQPLGDASIVPIRVTILDPNGRPPVRLDFQWRKNTQTGHWQAYDMIAEGVSMITTKQNEWSDLLRTKGIDGLTAQLKSIAQQKITLEEKK>UPI00049EB0AE Colicin V production protein OS=Citrobacter amalonaticus OX=35703 GN=cvpA SS=EMBLWGS:MBE0394906 PC=UP000642697:Unassembled WGS sequenceMVWIDYAIIAVIGFSCLVSLIRGFVREALSLVTWGCAFFVASHYYTYLSVWFSGFEDELVRNGIAIAVLFIATLIVGAIVNYVVGQLVEKTGLSGTDRVLGVCFGALRGVLIVAAILFFLDTFTGLSKGEDWSKSRLIPEFSFIIRWFFDYLQSSSSFLPRA>UPI00049EB1E3 Cytochrome c nitrite reductase pentaheme subunit OS=Citrobacter amalonaticus OX=35703 GN=nrfB SS=EMBLWGS:MBE0393944 PC=UP000642697:Unassembled WGS sequenceMSVLRSLLTAGVLASGLLWSLSGITATPTPQESERWEVTPQRSPDAACLDCHKPDTEGMHGKHASVTNPNNKLPVTCTNCHGKPSPQHREGAKDVMRFNEPMYNVEQQNSVCLSCHLPEQLQKAFWPHDVHVTKVACASCHSLHPKQDTMQTLSDKGRVKICVDCHSDQRNNPNFNPASVPLLKEHP>UPI00049EB223 TIGR03757 family integrating conjugative element protein OS=Citrobacter amalonaticus OX=35703 GN=ILP74_18380 SS=EMBLWGS:MBE0397334 PC=UP000642697:Unassembled WGS sequenceMKLTSLVFLPALLPASVLAGTVIFTDSQHLPANLPPDVPVVLLDGPDQLQAEMFGELPADPQQAEAQVRQVMASSAWQQKQLQLNDSYRQVVRAWELGIKKVPAVVFDDRDVVYGTTDVDVATSLRNRGGGQ>UPI00049EB2BC LacI family DNA-binding transcriptional regulator OS=Citrobacter amalonaticus OX=35703 GN=ILP74_13690 SS=EMBLWGS:MBE0396472 PC=UP000642697:Unassembled WGS sequenceMDKRLKIREIAARTQLSISTVSRVLAGKSNTSDNARRLVLECARELGVMEGMAAGRLLLNSLMIFAPQRAFDERSDIFYYRVIQSVNKGLASHEVRLRYCALEEHDSDAQLFLARMNEPDTHAAILLGIDDPHIHDLAVDVGKPCLLINCRDRLMRLPVVAPDHRAIGERAAGYLFEMGHREVMNVLCLRRYTMELRLAGIRDAWRSHNMKFTDDRDLLVATNFSARETEQLVSEWISERKGQDLPTAFLVGGDFMAAGVISALQKRGLRVPQDISVMSIDGFNLASIQDVPLTAVHVPRDELGTEAVYMLQQRLVRPEAPVSSLLLNGTLAVRESVRRIRQGKRRTAVEQEGLYDR>UPI00049EB2D8 Replication-associated recombination protein RarA OS=Citrobacter amalonaticus OX=35703 GN=rarA SS=EMBLWGS:MBE0396401 PC=UP000642697:Unassembled WGS sequenceMSNLSLDFSDNTFQPLAARMRPENLAQYIGQQHLLAAGKPLPRAIEAGHLHSMILWGPPGTGKTTLAEVIARYADADVERISAVTSGVKEIREAIERARQNRHAGRRTILFVDEVHRFNKSQQDAFLPHIEDGTITFIGATTENPSFELNSALLSRARVYLLKSLTTEDIEQVLRQAMEDKARGYGGQDIVLPDDTRKAIAELVNGDARRALNTLEMMADMAEVDDSGKRVLLPALLTEIAGERSARFDNKGDRFYDLISALHKSVRGSAPDAALYWYARIITAGGDPLYVARRCLAIASEDVGNADPRAMQVAISAWDCFTRVGPAEGERAIAQAIVYLACAPKSNAVYTAFKAALSDARERPDYDVPVHLRNAPTKLMKEMGYGQEYRYAHDEPNAYAAGEEYFPPEMAQTRYYHPTNRGLEGKIGEKLAWLAGQDQNSPIKRYR>UPI00049EB313 3-deoxy-D-manno-oct-2-ulosonate III transferase WaaZ OS=Citrobacter amalonaticus OX=35703 GN=waaZ SS=EMBLWGS:MBE0397955 PC=UP000642697:Unassembled WGS sequenceMSNVKFITRAGVQQLIANRTSDNVIIFLSGPTSRGTPLSLLQNNDVITVNGAAEYLLSNSITPFIYVLTDARFLLQRRHDFYKFSRASKFTVVNMDVYEAATEEDKKYMRTHCLILRAFYKREKGGFFKKLKLEMLSKWNKNLLIRVPISKRKRLVGFSKDISLGYCSCHTVAYTAMQIAYSLGYDNTICSGLDITGSCARFYDESRNPMPSELSKDLIKILPFFQFMCESVDDFNIYNLSDDTAISYDIIPYIKAADVAALPSRQHIRNNAHDLHDRADSYVS>UPI00049EB36C DUF2057 family protein OS=Citrobacter amalonaticus OX=35703 GN=ILP74_13005 SS=EMBLWGS:MBE0396341 PC=UP000642697:Unassembled WGS sequenceMKTGTVTLMIALCLPVTVFATTLRLSTDIDLLVLDGKKVSSSLLRGADSIELDNGPHQLVFRVEKTIRLPSHAEQLYISPPLVISFDTQRVEQVNFHLPRLESDRDASHFDVAPRLELLDGNAMPIPVQLDILAITSKTKAIDFEAETERYNKSAKRASLPQFATMMADDSTLLSGVSELDTIPPQSQTLTEQRLKYWFRQADPQTRNSFLQWAEKQPSS>UPI00049EB41D Formate hydrogenlyase subunit HycB OS=Citrobacter amalonaticus OX=35703 GN=hycB SS=EMBLWGS:MBE0394485 PC=UP000642697:Unassembled WGS sequenceMNRFVIADSTLCIGCHTCEAACSETHRQHGLQSMPRLKVMLNEKESAPQLCHHCEDAPCATVCPVNAINRVDGAVQLNESLCVSCKLCGIACPFGAIEFSGSRPLHIPANANTPKAPPAPPAPARVSTLLDWVPGVRAIAVKCDLCSFDEQGPACVRMCPTKALHLVNNMDIARASKRKRELTFNTDFGDLSLFQQAQSGEAK>UPI00049EB49C Glutathione-regulated potassium-efflux system ancillary protein KefG OS=Citrobacter amalonaticus OX=35703 GN=kefG SS=EMBLWGS:MBE0397715 PC=UP000642697:Unassembled WGS sequenceMSQPAKVLLLYAHPESQDSVANRVLLKPATQLSNVTVHDLYAHYPDFFIDIPHEQALLREHDVIVFQHPLYTYSCPALLKEWLDRVLSRGFASGPGGNQLAGKYWRSVITTGEPESAYRYDALNRYPMSDVLRPFELTAGMCRMHWLSPIIIYWARRQSAQELESHAKAYGDWLAHPVPVGGR>UPI00049EB510 MFS transporter OS=Citrobacter amalonaticus OX=35703 GN=ILP74_10950 SS=EMBLWGS:MBE0395957 PC=UP000642697:Unassembled WGS sequenceMSRTTTVNDATASDIDDQRISQPVQFIQRGTSAFMRVTLALFSAGLATFALLYCVQPILPVLSHEFGVSPASSSISLSISTGMLAVGLLFTGPLSDAIGRKPVMVTALLLASCCTLLSTMMTSWHGILVMRALIGLSLSGVAAVGMTYLSEEIHPSFVAFSMGLYISGNSIGGMSGRLISGVFTDFFSWRIALAAIGCFALASALMFWKILPESRHFRPTSLRPKTLFINFRIHWRDQGLPLLFAEGFLLMGSFVTLFNYIGYRLMLSPWELSQAVVGLLSVAYLTGTWSSPKAGSMTVRYGRGPVMLFSTVVMLFGLLMTLFSSLWLIFAGMLLFSAGFFGAHSVASSWIGPRARRAKGQASSLYLFSYYLGSSIAGTLGGVFWHNYGWNGVGGFIAIMLVLAILVGTRLHHRLHV>UPI00049EB553 Chitoporin OS=Citrobacter amalonaticus OX=35703 GN=chiP SS=EMBLWGS:MBE0396665 PC=UP000642697:Unassembled WGS sequenceMRTFSGKRSALALAIAGITALSGLVVVPQAQAEGFIDDSTLTGGIYYWQRERDRKDVTDGDKYKTNLSHATWNANLDFQSGYAADMFGLDVAAFTAIEMAENGDSGHPNEIAFSKKNKGYDEDYSGDKSGISLYKAAAKFKYGPAWARAGYIQPTGQTLLAPHWSFMPGTYQGAEAGANFDYGDAGALSFSYMWANEYKAPWHTEVDKFYQGDKKTKVDYLHSVGAKYDFKNDLVLEAAFGQSEGYVDQYFAKASYKFDLGGNPFSTSYQFYGARDKVDDRSASDIYDGTAWLQALTFGYKVAEVVDLRLEGTWVKAEGQQGFFLQRMTPTYASSNGRLDIWWDNRSDFNANGEKAVFFGAMYDLKNWNLPGWAVGASYVYAWDAKPSTWQSNPDAYYDKNRTIEESSYSLDAVYTLQDGRAKGTMFKLHFTEYDNHSNIPSWGGGYGNIFQDERDVKFIVIAPFTIF>UPI00049EB5F3 Flagella biosynthesis chaperone FliJ OS=Citrobacter amalonaticus OX=35703 GN=fliJ SS=EMBLWGS:MBE0395389 PC=UP000642697:Unassembled WGS sequenceMAEHGALATLKDLAEKEVDDAALLLGEMRRGFQQAEEQLTMLLDYQNEYRSNLNNDMSQGIASNRWINYQQFIQTLEKAVEQHRQQLTQWTQKVDMALNSWREKKQRLQAWQTLQDRQNAAALLAENRLDQKKMDEFAQRAAMRKPE>UPI00049EB65F Riboflavin synthase OS=Citrobacter amalonaticus OX=35703 GN=ILP74_11275 SS=EMBLWGS:MBE0396019 PC=UP000642697:Unassembled WGS sequenceMFTGIVQGTAKLVSIDEKPNFRTHVVELPEYMLDALETGASVAHNGCCLTVTEINGNRISFDLMKETLRITNLGDLKVGDLLNVERAAKFSDEIGGHLMSGHIMTTAEVSKILTSENNRQIWFKVQDPTLMKYILYKGFIGIDGISLTVGEVTATRFCVHLIPETLERTTLGKKKLGARVNIEIDPQTQAVVDTVERVLAAREAAVTKTASEA>UPI00049EB703 GMP/IMP nucleotidase OS=Citrobacter amalonaticus OX=35703 GN=yrfG SS=EMBLWGS:MBE0397749 PC=UP000642697:Unassembled WGS sequenceMHIDIAWQNVDTVLLDMDGTLLDLAFDNYFWQKLVPETYGAQNGISPQEAQERIRQEYHAVQHTLNWYCLDYWSDRLGLDICAMTTAMGPRAVLRDDTIPFLDALKACGKRRILLTNAHPHNLAVKLEHTGLASHLDLLLSTHTFGYPKEDQRLWRAVAEETGMSAEKTLFIDDSEPILDAAAKFGIRYCLGVTNPDSGVAEKQYARHPSLNDYRRLIPSLM>UPI00049EB74B Aldose-1-epimerase OS=Citrobacter amalonaticus OX=35703 GN=ILP74_23045 SS=EMBLWGS:MBE0398215 PC=UP000642697:Unassembled WGS sequenceMHSGGKTLHLQAGHYCAKIVTVGAGLAELTHRGRHVVIPHKPEEIPLAHLGKVLIPWPNRVAKGCYQHNGKRFQLAINDPVTHSAIHGLLAWRDWQINQQSKTEASLTIFLPPSYGYPFALVSEVIYRLEETHGLHVFIRTQNLSDEIAPYGAGAHPYLTCNLHPIDGCELTLPASEETGSERDFTTPRRIAGTSIDHTFRVLTPEVEWEVRMTCASQNITTFLRSHQPWLQVYTGEKLARRGLAVEPMTCPPDAFNSGTGLIYLAPGETHQMHFLIGSE>UPI00049EB78C TRNA (N6-threonylcarbamoyladenosine(37)-N6)-methyltransferase TrmO OS=Citrobacter amalonaticus OX=35703 GN=tsaA SS=EMBLWGS:MBE0397031 PC=UP000642697:Unassembled WGS sequenceMSHFQFEQIGVIRSPYKEKFAVPRQPGLVKSVNGELHLLSPYNQADAVRGLEAFSHLWVIFVFHQTMEGGWRPTVRPPRLGGNARMGVFATRSTFRPNPVGMSLVELKGVVCQKDQVILQLGSLDLVDGTPVIDIKPYLPFAESLPNATASYAQQAPQAEMSVSFTDEIEQQFPALEKRYPLLKTFIRDVLAQDPRPAYRKGEETGKNYAVWLHDFNVRWRVTETGFEVFALEPR>UPI00049EB856 DeoR family transcriptional regulator OS=Citrobacter amalonaticus OX=35703 GN=ILP74_22085 SS=EMBLWGS:MBE0398043 PC=UP000642697:Unassembled WGS sequenceMETKQKERIRRLMELLKKTDRIHLKDAARMLEVSVMTIRRDLSQDDEPLPLTLLGGYIVMVNKPAASSHAPLPAVKTHHRDDLPVAILAAGLVSENDLIFFDNGPEMPLVISMIPDEITFTGICYSHQVFIALNEKPNATAILCGGTYRAKSDAFYDANNPSPLDSLNPRKVFISASGVHEHFGVSWFNPDDLATKRKAMERGLRKILLTRYALFDEVAPASIGPLSTFDVLICDRPLPADYATHCRNGSVNVITPDSDGD>UPI00049EB88A Amino acid ABC transporter substrate-binding protein OS=Citrobacter amalonaticus OX=35703 GN=ILP74_20050 SS=EMBLWGS:MBE0397653 PC=UP000642697:Unassembled WGS sequenceMKKMMIATLAAAGVLLAVANQAHAGTTLDAVKKKGFVQCGISDGLPGFSYADADGKFSGIDVDVCRGVAAAVLGDDTKVKYTPLTAKERFTALQSGEVDLLSRNTTWTSSRDAGMGMSFTGVTYYDGIGFLTHNKAGLKSAKELDGATVCIQAGTDTELNVADYFKANNMKYTPVTFDRSDESAKALESGRCDTLASDQSQLYALRIKLSNPAEWLVLPEVISKEPLGPVVRRGDDEWFSIVRWTLFAMLNAEEMGVTSKNVDEKAANPATPDMAHLLGKEGDYGKDLKLDNKWAYNIIKQVGNYAEIFERNVGSESPLKIKRGQNNLWNNGGIQYAPPVR>UPI00049EB93A Sn-glycerol-3-phosphate ABC transporter permease UgpE OS=Citrobacter amalonaticus OX=35703 GN=ugpE SS=EMBLWGS:MBE0397794 PC=UP000642697:Unassembled WGS sequenceMIENRRGLTIFSHTMLILGIVVILFPLYVAFVAATLDNNAVFETPMTLIPGTHLLENMKNIWANGVGVNSAPFWLMMLNSFIMAFGITVGKIAVSMLSAFAIVWFRFPLRNLFFWMIFITLMLPVEVRIFPTVEVIANLNMLDSYAGLTLPLMASATATFLFRQFFMTLPDELIEAARIDGASPMRFFCDIVLPLSKTNLAALFVITFIYGWNQYLWPLLIITDVNLGTAVAGIKGMIATGEGTTLWNQVMAAMLLTLIPPVVIVLAMQRAFVRGLVDSEK>UPI00049EB97B Ferric iron uptake transcriptional regulator OS=Citrobacter amalonaticus OX=35703 GN=fur SS=EMBLWGS:MBE0396659 PC=UP000642697:Unassembled WGS sequenceMTDNNTALKKAGLKVTLPRLKILEVLQGPDNHHVSAEDLYKRLIDMGEEIGLATVYRVLNQFDDAGIVTRHNFEGGKSVFELTQQHHHDHLICLDCGKVIEFSDESIEARQREIASKHGIRLTNHSLYLYGHCAEGDCRENEHAHDAK>UPI00049EB983 YncE family protein OS=Citrobacter amalonaticus OX=35703 GN=ILP74_10435 SS=EMBLWGS:MBE0395862 PC=UP000642697:Unassembled WGS sequenceMHFRHLFSPRLRGSLLLGSLLVASSFSAQAAEDLLRKGVGKGAYEMAFSQQENALWLATSQSRKLDKGGIVYRLDPVTLDVTQVIHNDLKPFGATINNATQTLWFGNTVNSAVTAIDAKTSEVKGRLVLDARKRSEEVRPLQPRELVVDEATNTVYISGLGKESVIWVVDGEKIALKETIQNTGKMGAGLAIDSQAKRLYTTNADGEFITIDTATNKILSRKKLLEDGKEHFFINLSLDTAGHRAFVTDSKAAEVVVVDTRNGNVLAKVAAPESLAVLFNPTRNEVYVTHRQAGKVSVIDAKSYNVVKTFDTPTFPNSLALSADGKTLYVSVKQKSTREKEATEPDDVIRIAL>UPI00049EB9A7 Pyroglutamyl-peptidase I OS=Citrobacter amalonaticus OX=35703 GN=pcp SS=EMBLWGS:MBE0396635 PC=UP000642697:Unassembled WGS sequenceMKTVLITGFEPFGGEQINPSWEVVSQLDNAIIAGCRVVARQLPCVFGESLSVLNSAIDTLSPSLVLAIGQAGGRTDITVERVAINVDDARIPDNKGQQPVDEPIVAGGPAAWFSTLPVKAMVAAMREAGVPASVSQTAGTFVCNHVMYGLLHKLSGIAEVKGGFIHIPYLPQQAAAHPGAPSMAAETVRLALEVAIATALQVDDDIAVTGGATH>UPI00049EB9AD YqaE/Pmp3 family membrane protein OS=Citrobacter amalonaticus OX=35703 GN=ILP74_03380 SS=EMBLWGS:MBE0394542 PC=UP000642697:Unassembled WGS sequenceMGFWRIVFTIILPPLGVLLGKGFGWAFILNIVLTLLGYIPGLIHAFWVQTRPSSLS>UPI00049EB9F2 DUF412 domain-containing protein OS=Citrobacter amalonaticus OX=35703 GN=ILP74_05460 SS=EMBLWGS:MBE0394925 PC=UP000642697:Unassembled WGS sequenceMSTPDHRSVNFFSLFRRGQHYSKTWPMEKRLAPVFVENRVIRMTRYAIRFMPPIAIFTLCWQIALGGQLGPAVATALFALSLPMQGLWWLGKRSVTPLPPSILNWFYEVRGKLQEAGQALSPVEGKPDYQALADTLKRAFKQLDKTFLDDL>UPI00049EBA01 Uridine phosphorylase OS=Citrobacter amalonaticus OX=35703 GN=udp SS=EMBLWGS:MBE0398179 PC=UP000642697:Unassembled WGS sequenceMSRSDVFHLGLTKNDLQGATLAIVPGDPERVEKIAALMEKPVKLASHREFTTWRAELDGKAVIVCSTGIGGPSTSIAVEELAQLGIRTFLRIGTTGAIQPHINVGDVLVTTASVRLDGASLHFAPMEYPAVADFECTTALVEAAKSIGATTHVGVTASSDTFYPGQERYDTFSGRVVRRFKGSMEEWQSMGVMNYEMESATLLTMCASQGLRAGMVAGVIVNRTQQEIPNAETMKQTESHAVKIVVEAARRLI>UPI00049EBA04 Pyruvate dehydrogenase (Acetyl-transferring), homodimeric type OS=Citrobacter amalonaticus OX=35703 GN=aceE SS=EMBLWGS:MBE0397107 PC=UP000642697:Unassembled WGS sequenceMSERFPNDVDPIETRDWLQAIESVIREEGVERAQYLIDQLLSEARKGGVKVAAGTGASNYINTIAVEDEPEYPGNLELERRIRSAIRWNAIMTVLRASKKDLELGGHMASFQSSATVYDVCFNHFFRARNEQDGGDLVYFQGHISPGIYARAFLEGRLTEEQMDNFRQEVHGNGLSSYPHPKLMPEFWQFPTVSMGLGPIGAIYQAKFLKYLEHRGLKDTSKQTVYAFLGDGEMDEPESKGAITIATREKLDNLVFVINCNLQRLDGPVTGNGKIVNELEGIFEGAGWNVIKVMWGGRWDELLRKDTSGKLIQLMNETVDGDYQTFKSKDGAYVREHFFGKYPETAALVADWTDEQIWALNRGGHDPKKVYAALKKAQETKGKATVILAHTIKGYGMGDTAEGKNIAHQVKKMNMDGVRYIRDRFNVPVTDEQVEKLSYITFPEGSEEHKYLHERRQALHGYLPSRQPNFTEKLELPTLEDFGALLEEQNKEISTTIAFVRALNVMLKNKSIKDRLVPIIADEARTFGMEGLFRQIGIYSPNGQQYTPQDREQVAYYKEDEKGQILQEGINELGAGSSWLAAATSYSTNDLPMIPFYIYYSMFGFQRIGDLCWAAGDQQARGFLIGGTSGRTTLNGEGLQHEDGHSHIQSLTIPNCISYDPSYAYEVAVIMHDGLERMYGEKQENVYYYITTLNENYHMPAMPEGAEEGIRKGIYKLETLEGSKGKVQLLGSGSILRHVREAAEILAKDYGVGSDVYSVTSFTELARDGQDCERWNMLHPLETPRVPYIAQVMNDAPAVASTDYMKLFAEQVRTYVPADDYRVLGTDGFGRSDSRENLRHHFEVDASYVVVAALGELAKRGEIDKKVVADAITKFNIDAEKVNPRLA>UPI00049EBA2D TIGR03752 family integrating conjugative element protein OS=Citrobacter amalonaticus OX=35703 GN=ILP74_18415 SS=EMBLWGS:MBE0397341 PC=UP000642697:Unassembled WGS sequenceMQIKSNALVKFIVPAVVIAGLAVGLKSCKNEPAEQSQAKQSGGPLHNLSPDELKALGVEGDTPEDTLRTLIGRLNDVRDRQKTLDKQNADLVKENERLRRRNQDVSGQVNEAVNGLREQYDKRQAQLQNEQLSLTAKIQELTDKLKKPDSEKKTADSDIPLGLGLDGMGSSTGGSASQGSDGMMWVAPTDQKETDPRDAASGKASPQFPTSFLGENELTRQKAAYEEKVKGRTNEKGAEESAEPVYTLPENSTLVGSRAMTALLGRVPINGTVTDPYPFKVLIGKDNLTANGIELPDVEGAIVSGTASGDWTLACVRGQVNSVTFVFSDGTVRTLPRPDTSNNAGGQNNNAQAKQDTGTSGGIGWISDENGIPCIGGERKSNASTYLPTIFGLSAAGAAGEAMSQGQYTTQNNVNGISATMTGDAGQAALGKAISGGMSETTDWIKQRYGMAFDAIYVPPGARLAVHITRQLAIDYEDKGRKVRYDFTLPGGVSDNGGLD>UPI00049EBA48 Arabinose-5-phosphate isomerase KdsD OS=Citrobacter amalonaticus OX=35703 GN=kdsD SS=EMBLWGS:MBE0397564 PC=UP000642697:Unassembled WGS sequenceMSHLALQPGFDFQQAGKEVLAIEREGLAELDQYINQDFTLACEKMFNCTGKVVVMGMGKSGHIGRKMAATFASTGTSSFFVHPGEAAHGDLGMVTSQDVVIAISNSGESSEIAALIPVLKRLQVQLICITGRPESSMARAADVHLCVKVPQEACPLGLAPTSSTTATLVMGDALAVALLKARGFTAEDFALSHPGGALGRKLLLRVNDIMHTGDEIPHVTKEASLRDALLEITRKNLGMTVICDDAMKIDGIFTDGDLRRVFDMGVDVRQLGIADVMTPGGIRVRPGILAVDALNLMQSRHITSVMVADGDQLLGVLHMHDLLRAGVV>UPI00049EBB08 Protein phosphatase CheZ OS=Citrobacter amalonaticus OX=35703 GN=cheZ SS=EMBLWGS:MBE0395516 PC=UP000642697:Unassembled WGS sequenceMMQPQIKPVDEHSAGDIIARIGSLTRMLRDSLRELGLDQAIAEAAEAIPDARDRLDYVVQMTAQAAERALNSVEASQPHQDEMEKGAKALSKRWDEWFENPIELSDARELVTDTRKYLGDVPGHTSFTNAQLLDIMMAQDFQDLTGQVIKRMMDVIQEIERQLLMVLLENIPEQSSRPKRESESLLNGPQVDATKAGVVASQDQVDDLLDSLGF>UPI00049EBBC9 Trk system potassium transporter TrkH OS=Citrobacter amalonaticus OX=35703 GN=trkH SS=EMBLWGS:MBE0398194 PC=UP000642697:Unassembled WGS sequenceMHFRAITRIVGLLVILFSGTMILPGLVALIYRDGAGRAFTQTFFVALVIGSMLWWPNRREKGELKSREGFLIVVLFWTVLGSVGALPFIFSESPNLTITDAFFESFSGLTTTGATTLVGLDSLPHAILFYRQMLQWFGGMGIIVLAVAILPILGVGGMQLYRAEMPGPLKDNKMRPRIAETAKTLWLIYVLLTVACALALWFAGMPAFDAIGHSFATIAIGGFSTHDASVGYFDSPTINTIIAIFLLISGCNYGLHFSLLSGRSLKVYWRDPEFRMFIGVQLTLVVICTLVLWFHNIYNSALTTLNQAFFQVVSMATTAGFTTDSIARWPLFLPVLLLCSAFIGGCAGSTGGGLKVIRILLLFKQGNRELKRLVHPNAVYSIKLGNRALPERILEAVWGFFSAYALVFIVSMLAIIATGVDDFSAFASVVATLNNLGPGLGVVADNFASMNPVAKWILIANMLFGRLEVFTLLVLFTPTFWRE>UPI00049EBC24 YecH family protein OS=Citrobacter amalonaticus OX=35703 GN=ILP74_08490 SS=EMBLWGS:MBE0395491 PC=UP000642697:Unassembled WGS sequenceMDSVHGHEVLNMMIESGENYSTASLEAAIKARFGEAARFHTCSASGMTAGELVAFLAAKGKFIAQDDGFSTHESKICRH>UPI00049EBC43 C40 family peptidase OS=Citrobacter amalonaticus OX=35703 GN=ILP74_11590 SS=EMBLWGS:MBE0396076 PC=UP000642697:Unassembled WGS sequenceMRFWFLFCTALLLAGCSSHRAPPPNARLSDSITVIAGLNDQLQTWRGTPYRYGGMSRGGVDCSGFVLMTMRDQFSLQLPRETRQQAKLGTEIDKDDLLPGDLVFFKTGSGESGLHVGIYDTNGQFIHASTSRGVMRSSLDNVYWRKNFWQARRI>UPI00049EBC9A Prolipoprotein diacylglyceryl transferase OS=Citrobacter amalonaticus OX=35703 GN=lgt SS=EMBLWGS:MBE0394396 PC=UP000642697:Unassembled WGS sequenceMTSSYLHFPEFDPVIFSIGPVALHWYGLMYLVGFIFAMWLATRRANRPGSGWTKNEVENLLYAGFLGVFLGGRIGYVLFYNFPLFLDNPLYLFRVWDGGMSFHGGLIGVILVMIIFAKRTKRSFFQVSDFIAPLIPFGLGAGRLGNFINGELWGRVDPNFSYAMLFPGSRTEDILLLQTNPQWQSIFDTYGSLPRHASQLYEMALEGVVLFIILNLFIRKPRPMGSVSGLFLIGYGAFRIIVEFFRQPDQQFTGEWVQYISMGQILSIPMIVAGVIMMVWAYRRRPQQHVS>UPI00049EBCCB Urease subunit gamma OS=Citrobacter amalonaticus OX=35703 GN=ILP74_18905 SS=EMBLWGS:MBE0397434 PC=UP000642697:Unassembled WGS sequenceMELTPREKDKLLLFTAALVAERRLARGLKLNYPESVALISAFIMEGARDGKSVASLMEDGRHVLNREQVMEGVPEMIPDIQVEATFPDGSKLVTVHSPIV>UPI00049EBD8D Acetolactate synthase small subunit OS=Citrobacter amalonaticus OX=35703 GN=ilvN SS=EMBLWGS:MBE0398044 PC=UP000642697:Unassembled WGS sequenceMQKQSHENVILELTVRNHPGVMTHVCGLFARRAFNVEGILCLPIQSSDQSRIWLLVNDDQRLEQMISQIDKLEDVVKVVRNQSDPTMFNKIAVFFE>UPI00049EBDF3 D-alanyl-D-alanine carboxypeptidase DacA OS=Citrobacter amalonaticus OX=35703 GN=dacA SS=EMBLWGS:MBE0396694 PC=UP000642697:Unassembled WGS sequenceMKTTFSARFLQRMALTTALCTAAISGAHADDLNIKTMIPGVPQIDAESYILIDYNSGKVLAEQNADTRRDPASLTKMMTSYVIGQAMKAGKFKETDLVTIGNDAWATGNPVFKGSSLMFLKPGMQVPVSQLIRGINLQSGNDACVAMADFAAGSQDAFVGLMNSYVNALGLKNTHFQTVHGLDADGQYSSARDMALIGQALIRDVPNEYSVYKEKEFTFNGIRQLNRNGLLWDNSLKVDGIKTGHTDKAGYNLVASATEGQMRLISAVMGGRTYKGRETESKKLLTWGFRFFETVNPLKAGKEFASEPSWFGDTDRASLGVDKDVYLTIPRGRMKDLKASYVLNSTELHAPLQKNQVVGTINFQLDGKTIEQRPLVVLQEIPEGNFFSKIIDYIKLMFHHWFG>UPI00049EBE75 Class 1b ribonucleoside-diphosphate reductase subunit beta OS=Citrobacter amalonaticus OX=35703 GN=nrdF SS=EMBLWGS:MBE0394530 PC=UP000642697:Unassembled WGS sequenceMTLSRVSAINWNKIQDDKDLEVWNRLTSNFWLPEKVPLSNDIPAWQNLSAAEQQLTIRVFTGLTLLDTIQNIAGAPSLMADSLTPHEEAVLSNISFMEAVHARSYSSIFSTLCQTKDVDAAYAWSEENAPLQQKAQIILAHYASDDPLKKKIASVFLESFLFYSGFWLPMYFSSRGKLTNTADLIRLIIRDEAVHGYYIGYKYQKGLEKRSQSERDELKDFALELLMELYDNEVRYTEELYADTAWAEDVNAFLCYNANKALMNLGYEALFPAEMAEVNPAILAALSPNADENHDFFSGSGSSYVMGKAVETEDEDWNF>UPI00049EBF06 Stress response protein ElaB OS=Citrobacter amalonaticus OX=35703 GN=elaB SS=EMBLWGS:MBE0394948 PC=UP000642697:Unassembled WGS sequenceMSYQFGDSRIDDDLTLLSETLEEVLRSSGDPADQKYIELKARAEKALDEVKNRVSQASDSYYYRARQAVYRADDYVHEKPWQGIGVGAAVGLVLGLLLARR>UPI00049EBF38 Cold shock-like protein CspD OS=Citrobacter amalonaticus OX=35703 GN=cspD SS=EMBLWGS:MBE0396414 PC=UP000642697:Unassembled WGS sequenceMETGTVKWFNNAKGFGFICPEGGGEDIFAHYSTIQMDGYRTLKAGQSVQFDVHQGPKGNHASVIVPIEAEAVA>UPI00049EBFEA Isochorismatase family protein OS=Citrobacter amalonaticus OX=35703 GN=ILP74_16180 SS=EMBLWGS:MBE0396936 PC=UP000642697:Unassembled WGS sequenceMSEKRVVMVVDMQIGVFATPRINRDACVERINQLTRAADLTIFIQHTEAGGLEEGSAGFALLPELETPVNAKYVTKTACDAFYKTALEQLLREQAISHFVICGCATDYCVDTTIRNGASRSYRITVAEDAHTTAHRAAAPAQTLIAHHNDVWRNLIVPGNPVRVKPVETILQDWKTN>UPI00049EC0C3 NAD-dependent DNA ligase LigA OS=Citrobacter amalonaticus OX=35703 GN=ligA SS=EMBLWGS:MBE0394750 PC=UP000642697:Unassembled WGS sequenceMESIEQQLTELRTTLRHHEYLYHVMDAPEIPDAEYDRLMRELRELEAQHPELITPDSPTQRVGAAPLASFSQIRHEVPMLSLDNVFDEESFLAFNKRVQDRLKSSDKLTWCCELKLDGLAVSILYENGVLVSAATRGDGTTGEDITSNVRTIRAIPLKLHGDNIPTRLEVRGEVFLPQAGFEKINEDARRTGGKLFANPRNAAAGSLRQLDPRITAKRPLTFFCYGVGVLEGGELPDTHLGRLLQFKAWGLPVSDRVTLCDSPEAVLAFYHKVEEDRPTLGFDIDGVVIKVNSMALQEQLGFVARAPRWAVAFKFPAQEQMTFVRDVEFQVGRTGAITPVARLEPVQVAGVLVSNATLHNADEIERLGLRIGDKVVIRRAGDVIPQVVNVVLSERPDDTRPVAFPTHCPVCGSDVERVEGEAVARCTGGLICGAQRKESLKHFVSRRAMDVDGMGDKIIDQLVEKEYVHTPADLFRLTAGKLTGLDRMGPKSAQNVVEALEKSKSTTFARFLYALGIREVGEATAAGLAAYFGTLEALEAASIDELQKVPDVGIVVATHVFNFFAEESNREVIGQLLAEGIHWPAPVVINAEEIDSPFAGKTVVLTGSLSQMSRDDAKARLVELGAKVAGSVSKKTDLVIAGEAAGSKLAKAQELGIAVIDEAEMLRLLGV>UPI00049EC0F4 YbfA family protein OS=Citrobacter amalonaticus OX=35703 GN=ILP74_14610 SS=EMBLWGS:MBE0396644 PC=UP000642697:Unassembled WGS sequenceMELYKEYPAHVVLLRRTFAVVAGVLALPVMLFWKDRARFYSYLHRVWSKTSDKPVWMDQAEKATCDFY>UPI00049EC0F5 Uncharacterized protein OS=Citrobacter amalonaticus OX=35703 GN=ILP74_05660 SS=EMBLWGS:MBE0394965 PC=UP000642697:Unassembled WGS sequenceMKKSVLFGIAGMLFVSASASAMSISGQAGKEYTNVGVGFGTESTGLALSGNWMHSDDDGDVAGLGLGLNLPLGPLMATVGGKGVYTNPNDSDEGYAAAVGGGLQWPIGDSFRLFGEYYYSPDSLSSGIQSYEEANAGARLTIMRPLSIEAGYRYLNLAGKDGNRDNAIADGPYVGVNASF>UPI00049EC0FA Two-component response regulator DpiA OS=Citrobacter amalonaticus OX=35703 GN=dpiA SS=EMBLWGS:MBE0396705 PC=UP000642697:Unassembled WGS sequenceMTEPLTLLIVEDEMLLAEMHAEYIRHIPGFNQIWLAGNLAQARMMIERFKPGLILLDNYLPDGKGITLLHELTASHYPGGVVFTTAASDMETVADAVRSGAFDYLIKPIAYERLGQTLTRYQQRRRMLAENDSASQRQIDEMFNAYARGEPKDELPTGIDALTLNAVKKLFADPQVQHTAETVAQALTISRTTARRYLEYCASRHLVIAEIVHGKVGRPQRIYHG>UPI00049EC12B Phosphate starvation-inducible protein PsiF OS=Citrobacter amalonaticus OX=35703 GN=psiF SS=EMBLWGS:MBE0396926 PC=UP000642697:Unassembled WGS sequenceMKITLLVTLLFGLIFLTTVGAAEKTLTPQQQRMTTCNQQATAKALKGDARKTYMSDCLKNSKSAPGEKSLTPQQQKMRECNVQATEQSLKGDDRSKFMSACLKKAA>UPI00049EC182 DUF1456 family protein OS=Citrobacter amalonaticus OX=35703 GN=ILP74_06590 SS=EMBLWGS:MBE0395143 PC=UP000642697:Unassembled WGS sequenceMLSNDILRSLRYALKANNNDMVRILALADMESTSAGFDTWMTREDEEGFVRCPDIILSGFLNGLIYEKRGKDESAPELALERRVNNNTVLKKLRIAFSLKTDDIQAIMTQQNVRVSMPEITAMMRSPDHKNYRECGDQFMRYFLRGLTARLHGNKG>UPI00049EC2A7 Anaerobic glycerol-3-phosphate dehydrogenase subunit A OS=Citrobacter amalonaticus OX=35703 GN=glpA SS=EMBLWGS:MBE0394974 PC=UP000642697:Unassembled WGS sequenceMKTRDSQTSDVIIIGGGATGAGIARDCALRGLRVILVERHDIATGATGRNHGLLHSGARYAVTDAESARECISENQILKRIARHCVEPTDGLFITLPEDELSFQATFIRACEEAGIRAEAIDPQQARIIEPSVNPQLIGAVKVPDGTVDPFRLTAANMLDAKEHGAIILTAHEVTGLIREGATVSGVHVRNHLNGETQTLHAPVVVNAAGIWGQRIAEYADLSIRMFPAKGSLLIMDHRINQHVINRCRKPSDADILVPGDTISLIGTTSTHIDYNDIDSNRVTADEVDILLREGEKLAPIMAKTRILRAYSGVRPLVASDDDPSGRNVSRGIVLFDHAKRDGLDGFITITGGKLMTYRLMAEWATDAVCRKLGNTRPCITADTPLPGSQEPTESTLKRVISLPAPLRGSAVYRHGDRTPAWLSHGRQHRSLVCECEAVTAGEVQYAVENLNVNSLLDLRRRTRVGMGTCQGELCACRAAGLLQRFNITTAAQSITQLSDFLNERWKGVQPIAWGDALRESEFTRWVYQGLCGLEKEPRDEI>UPI00049EC32B DNA-directed RNA polymerase subunit beta OS=Citrobacter amalonaticus OX=35703 GN=rpoC SS=EMBLWGS:MBE0398386 PC=UP000642697:Unassembled WGS sequenceMKDLLKFLKAQTKTEEFDAIKIALASPDMIRSWSFGEVKKPETINYRTFKPERDGLFCARIFGPVKDYECLCGKYKRLKHRGVICEKCGVEVTQTKVRRERMGHIELASPTAHIWFLKSLPSRIGLLLDMPLRDIERVLYFESYVVIEGGMTNLERQQILTEEQYLDALEEFGDEFDAKMGAEAIQALLKSMDLEQECETLREELNETNSETKRKKLTKRIKLLEAFVQSGNKPEWMILTVLPVLPPDLRPLVPLDGGRFATSDLNDLYRRVINRNNRLKRLLDLAAPDIIVRNEKRMLQEAVDALLDNGRRGRAITGSNKRPLKSLADMIKGKQGRFRQNLLGKRVDYSGRSVITVGPYLRLHQCGLPKKMALELFKPFIYGKLELRGLATTIKAAKKMVEREEAVVWDILDEVIREHPVLLNRAPTLHRLGIQAFEPVLIEGKAIQLHPLVCAAYNADFDGDQMAVHVPLTLEAQLEARALMMSTNNILSPANGEPIIVPSQDVVLGLYYMTRDCVNAKGEGMVLTGPKEAERIYRAGLASLHARVKVRITEYEKDANGEFVAKTSLIDTTVGRAILWMIVPKGLPFSIVNQALGKKAISKMLNTCYRILGLKPTVIFADQTMYTGFAYAARSGASVGIDDMVIPEKKHEIISEAEAEVAEIQEQFQSGLVTAGERYNKVIDIWAAANDRVSKAMMDNLQTETVINRDGQEEQQVSFNSIYMMADSGARGSAAQIRQLAGMRGLMAKPDGSIIETPITANFREGLNVLQYFISTHGARKGLADTALKTANSGYLTRRLVDVAQDLVVTEDDCGTLEGITMTPVIEGGDVKEPLRDRVLGRVTAEDILKPGTADILVPRNTLLHEQWCDLLEANSVDSVKVRSVVSCDTDFGVCAHCYGRDLARGHIINKGEAIGVIAAQSIGEPGTQLTMRTFHIGGAASRAAAESSIQVKNKGSIRLSNAKSVVNSSGKLVITSRNTELKLIDEFGRTKESYKVPYGSVMAKGDGEQVAGGETVANWDPHTMPVITEVSGFVRFTDMIDGQTITRQTDELTGLSSLVVLDSAERTTGGKDLRPALKIVDAQGNDVLIPGTDMPAQYFLPGKAIVQLEDGVQISSGDTLARVPQESGGTKDITGGLPRVADLFEARRPKEPAILAEISGIISFGKETKGKRRLVITPVDGSDPYEEMIPKWRQLNVFEGERVERGDVVSDGPEAPHDILRLRGVHAVTRYIVNEVQDVYRLQGVKINDKHIEVIVRQMLRKATIESAGSSDFLEGEQVEYSRVKIANRELEANGKVGATFSRDLLGITKASLATESFISAASFQETTRVLTEAAVAGKRDELRGLKENVIVGRLIPAGTGYAYHQDRMRRRAAGELPAAPQVTAEDASASLAELLNAGLGGSDNE>UPI00049EC385 Helix-turn-helix transcriptional regulator OS=Citrobacter amalonaticus OX=35703 GN=ILP74_23770 SS=EMBLWGS:MBE0398355 PC=UP000642697:Unassembled WGS sequenceMYQDVSHLLSRLIHGPAPLRQIFFASTLTPIPALAYQVDFPRLEIVLEGELTDASLTDTLTPYDVLYVPAGGWNLPQWQVPVITLSILFGKQQLGFSVVQWDGETYQNLVKQHVARRGPRIGSFLLQTLNEMQMQPQEQQTARLIVASLLSHCRDLLGSQIQTASRSQALFEAIRDYIDERYASPLTRESVAQAFYISPNYLSHLFQKTGAVGFNEYLNHTRLEHAKTLLKGYDLKVKEVAHTCGFVDSNYFCRLFRKNTERSPSEYRRQYHSQLTEKQVSPE>UPI00049EC401 5-dehydro-4-deoxy-D-glucuronate isomerase OS=Citrobacter amalonaticus OX=35703 GN=kduI SS=EMBLWGS:MBE0394381 PC=UP000642697:Unassembled WGS sequenceMDVRQSIHSAHAKTLDTQGLRKEFLVEKVFVADEYTMVYSHIDRIIVGGIMPVAKTVSVGGEVGKQLGVTYFLERRELGVINIGGAGTITVDGQCYEIGHRDALYVGKGAKEVVFASVDSATPAKFYYNCAPAHTTYPTKKVTPADVAPVTLGDNLTSNRRTINKYFVPDVLETCQLSMGLTELAPGNLWNTMPCHTHERRMEVYFYFNMEEDTCVFHMMGQPQETRHIVMHNEQAVISPSWSIHSGVGTKAYTFIWGMVGENQVFDDMDHVAVKDLR>UPI00049EC40C Phosphohistidine phosphatase SixA OS=Citrobacter amalonaticus OX=35703 GN=sixA SS=EMBLWGS:MBE0394862 PC=UP000642697:Unassembled WGS sequenceMQVFIMRHGDAALDAASDSVRPLTPCGCDESRLMANWLKGQKVDIERVLVSPFLRAEQTLDVVGGCMNLPRDVDVLPELTPCGDVGLVSAYLQALANEGVATALVISHLPLVGYLVSELCPGETPPMFTTSAIASVTLDESGKGVFNWQMSPCNLKMAKAI>UPI00049EC44B Adenylosuccinate synthase OS=Citrobacter amalonaticus OX=35703 GN=purA SS=EMBLWGS:MBE0394061 PC=UP000642697:Unassembled WGS sequenceMGNNVVVLGTQWGDEGKGKIVDLLTERAKYVVRYQGGHNAGHTLVINGEKTVLHLIPSGILRENVISIIGNGVVLSPAALMKEMKGLEDRGVPVRERLLLSEACPLILDYHVALDNAREKARGAKAIGTTGRGIGPAYEDKVARRGLRVGDLFDKATFAEKLKEVMEYHNFQLVNFYKVDAVDYQKVLDDVMAIADILTSMVVDVSDLLDQARKRGDFVMFEGAQGTLLDIDHGTYPYVTSSNTTAGGVATGSGLGPRYVDYVLGIIKAYSTRVGAGPFPTELFDDIGEFLCKQGNEYGATTGRRRRTGWLDSVAVRRAVQINSLSGFCLTKLDVLDGLKEVKICVAYRMPDGREVTTTPLAADDWEGIEPIYETMPGWSESTFGVKERSGLPQAALNYIKRIEELTGVPIDIISTGPDRTETMILRDPFDA>UPI00049EC45A General stress protein OS=Citrobacter amalonaticus OX=35703 GN=ILP74_10050 SS=EMBLWGS:MBE0395789 PC=UP000642697:Unassembled WGS sequenceMAEHRGGSGNFAEDRDKASEAGKKGGQHSGGNFKNDPQRASEAGKKGGQNSHSGGRKSDNS>UPI00049EC49A DJ-1/PfpI family protein OS=Citrobacter amalonaticus OX=35703 GN=ILP74_08530 SS=EMBLWGS:MBE0395499 PC=UP000642697:Unassembled WGS sequenceMKRVAVLLASGFEEAEAIVTIDILRRLNIDVDTLACAESRAVVSYHNVPMVTDYTLAGQQERLYDAVVLPGGPQGSVNLAANPQVIEFVSRHDAAGKLICPICSAAARVLGGNGLLKGRRYVCSGDLYKTVTDGVYVDAPVVEDGHLLSGKGLGHIFDFALTLSARLLGDDQPVREHAEHIYYPW>UPI00049EC4A3 Type I DNA topoisomerase OS=Citrobacter amalonaticus OX=35703 GN=topA SS=EMBLWGS:MBE0395719 PC=UP000642697:Unassembled WGS sequenceMGKALVIVESPAKAKTINKYLGNDYVVKSSVGHIRDLPTSGSAAKKSADSTSTKTAKKPKKDERGALVNRMGVDPWHNWDAHYEVLPGKEKVVSELKQLAEKADHIYLATDLDREGEAIAWHLREVIGGDDTRYSRVVFNEITKNAIRQAFEKPGELNIDRVNAQQARRFMDRVVGYMVSPLLWKKIARGLSAGRVQSVAVRLVVEREREIKAFVPEEFWEIDANTTTPSGDALPLQVTHQNDKPFRPENREQTLAAVSLLEKARYSVLEREDKPTSSKPGAPFITSTLQQAASTRLGFGVKKTMMMAQRLYEAGYITYMRTDSTNLSQDAVNMVRGYIGDNFGKKYLPESPNQYASKENSQEAHEAIRPSDVSVLAETLKDMEADAQKLYQLIWRQFVACQMTPAQYDSTTLTVGAGDFRLKARGRILRFDGWTKVMPALRKGDEDRTLPAVNKGDALSLVELIPAQHFTKPPARFSEASLVKELEKRGIGRPSTYASIISTIQDRGYVRVENRRFYAEKMGEIVTDRLEENFRELMNYDFTAQMENSLDQVANHEIEWKGVLDNFFSDFTQQLDKAEKDPEEGGMRPNQMVLTSIDCPTCGRKMGIRTASTGVFLGCSGYALSPKERCKTTINLVPENEVLNVLEGDDAETNALRAKRRCQKCGTAMDSYLIDPKRKLHVCGNNPTCDGYEIEEGEFRIKGYDGPIVECEKCGSEMHLKMGRFGKYMACTNDECKNTRKILRNGEVAPPKEDPVPLPELPCEKSDAYFVLRDGAAGVFLAANTFPKSRETRAPLVEELYRFRDRLPEKLRYLADAPQQDPEGNKTLVRFSRKTKQQYVAAEKDGKATGWSAFFVDGKWVEGKK>UPI00049EC513 Dipeptide ABC transporter permease DppB OS=Citrobacter amalonaticus OX=35703 GN=dppB SS=EMBLWGS:MBE0397885 PC=UP000642697:Unassembled WGS sequenceMLQFILRRLGLVIPTFIGITLLTFAFVHMIPGDPVMIMAGERGISPERHAQLLAELGLDKPMWQQYLHYIWGVMHGDLGISLKSRLPVWDEFVPRFKATLELGVCAMIFATAVGIPVGVLAAVKRGSIFDHTAVGLALTGYSMPIFWWGMMLIMLVSVQLNLTPVSGRVSDMVFLDDSNPLTGFMLIDTAIWGEEGNFIDAVVHMILPAMVLGTIPLAVIVRMTRSSMLEVLGEDYIRTARAKGLTRMRVIIIHALRNAMLPVVTVIGLQVGTLLAGAILTETIFSWPGLGRWLIDALQRRDYPVVQGGVLLVATMIILVNLLVDLLYGVVNPRIRHKK>UPI00049EC5FC Alpha,alpha-trehalase OS=Citrobacter amalonaticus OX=35703 GN=ILP74_21120 SS=EMBLWGS:MBE0397857 PC=UP000642697:Unassembled WGS sequenceMINQKLQHAPSEEITIDVDLFYETDPCELKLDEMIEAEPEPEMIEGLPASDALTPADRYLELFEHVQSSKIFADSKTFPDCAPKMDPLDILIRYRKVRRHRDFDLRRFVENHFWLPEVFDSAYVSNPENSLKEHIDQLWPVLTREPQDHIPWSSLLALPQSYIVPGGRFSETYYWDSYFTMLGLAESGREDLLKCMADNFAWMIENYGHIPNGNRTYYLSRSQPPVFALMVELFEEDGVRGARRYLDHLKMEYAFWMDGAESLVLNQAYRHAVRMPDGSLLNRYWDDRDTPRDESWLEDVETAKHSGRPPNEVYRDLRAGAASGWDYSSRWLRDPTRLASIRTTQFIPIDLNAFLFKLESTIANISALKGERDVEALFRQKASNRRAAVNRYLWDEENGCFRDYDWRREQMALFSAASIVPLYVGMATHEQADRLGDTVRARLLTPGGIMASEYETGEQWDKPNGWAPLQWMAIQGFKMYGDDALGDEIAHSWLQTVNQFYQQHHKLIEKYHIASGTPREGGGGEYPLQDGFGWTNGVARRLIGLYGEP>UPI00049EC611 YaiY family protein OS=Citrobacter amalonaticus OX=35703 GN=ILP74_16165 SS=EMBLWGS:MBE0396933 PC=UP000642697:Unassembled WGS sequenceMADFTLSKSLFNGKHRETASTPGNIAYAVFVLFCFWAGAQILNLLVHAPGVYEHLMQVQETGRPRVEIGLGVGTIFGLVPFLVGSLIFGVIAAILRWRYRRQ>UPI00049EC750 RamA family antibiotic efflux transcriptional regulator OS=Citrobacter amalonaticus OX=35703 GN=ramA SS=EMBLWGS:MBE0396758 PC=UP000642697:Unassembled WGS sequenceMTISAQVIDTIVEWIDDNLNQPLRIDDIARHAGYSKWHLQRLFLQYKGESLGRYIRERKLLLAARDLRESDEKVYDICLRYGFDSQQTFTRIFTRTFNQPPGAYRKENHSRAH>UPI00049EC8E9 Rsd/AlgQ family anti-sigma factor OS=Citrobacter amalonaticus OX=35703 GN=ILP74_24030 SS=EMBLWGS:MBE0398396 PC=UP000642697:Unassembled WGS sequenceMLNQLEDLTERVRGSNKLVDRWLHVRKHLLVAYYNLVGIKPGKESYMQLNEKALDDFCQSLVDYLSTGHFSIYERILHKLEGNGQLLKATKIWPLLEANTQRIMDYYDSSLETAIDHDNCLEFQQVLSDLGEALEARFALEDKLIMLVFDAMHDNAVIKRPA>UPI00049EC96A Cytochrome b561 OS=Citrobacter amalonaticus OX=35703 GN=cybB SS=EMBLWGS:MBE0395810 PC=UP000642697:Unassembled WGS sequenceMGNKYSSLQIGIHWLVFLLVIVAYCAMEFRGFFPRSDRPLINMIHVSSGIAILVLMVARLLIRLKYPAPPIVPKPKPMMTGLAHLGHLVIYLLFIALPLIGMVMMYNRGNPWMAFGLVMPHAAEGNFDLVDTLKSWHITLANLGYFVIALHAAAALLHHYFWKDNTLLRMMPRKR>UPI00049ECB19 AI-2E family transporter YdiK OS=Citrobacter amalonaticus OX=35703 GN=ydiK SS=EMBLWGS:MBE0396057 PC=UP000642697:Unassembled WGS sequenceMVNIRQPRDIAQVLLSVLFLAIMIVACLWIVQPFILGFAWAGTIVIATWPVLLRLQKLLWGRRSLAVLVMTLLLVLLFVIPIALLVNSIVDGSGPLIHAVTSGDMTLPDLAWLNSIPLVGAKLYAGWHNLLDMGGTAIMAKVRPYIGTTTTWFVGQAAHIGRFMMHCSLMLLFSALLYWRGETVALGIRHFAYRLAAKRGDAAMLLAAQAIRAVALGVVVTALVQAVLGGIGLAVSGVPYATLLSVVMILSCLVQLGPLPVLIPAIIWLYWSGDTTWGSVLLAWSCVVGTLDNVIRPMLIRMGADLPLILILSGVIGGLIAFGMIGLFIGPVLLAVSWRLFSAWVNEAPVPTSEPEEILEELGEIENLKK>UPI00049ECB5F 6-N-hydroxylaminopurine resistance protein OS=Citrobacter amalonaticus OX=35703 GN=yiiM SS=EMBLWGS:MBE0398266 PC=UP000642697:Unassembled WGS sequenceMRYPVDVFSGKIQDYAGSRPSAIGKIQVDGELMLTELGLEGDEQAETKIHGGPDRALCHYPREHYLYWAREFPDQADQFVAPAFGENLSTDGLTEHNVYIGDIFRWGEALIQVTQPRSPCFKLNFHFGISDMASLMQNCGKTGWLCSVIAPGMVSADAPLELVSRVSDVSVQEAIAIAWHMPFDDEQYHRLLSAAGLSKSWTRTMQKRRLSGKIEDQSRRLWGK>UPI00049ECC5D 4Fe-4S dicluster domain-containing protein OS=Citrobacter amalonaticus OX=35703 GN=ILP74_15870 SS=EMBLWGS:MBE0396879 PC=UP000642697:Unassembled WGS sequenceMKQYGFLIDMQNCYGCKTCSMACKSENMTPMGVLWRRVRERHTDEPNTQAFISMSCNHCDDPQCMKVCPAGTYSKRADGIVVQDHDRCIGCRMCIMACPWSAPVYDPAEGKTSKCNLCAERLDEGLQPRCVESCPAGVLRFGEIAELRKEHTTPWAVLEKRYNLPDHTISQPNIVIIPPRD>UPI00049ECD1F TRNA 4-thiouridine(8) synthase ThiI OS=Citrobacter amalonaticus OX=35703 GN=thiI SS=EMBLWGS:MBE0396882 PC=UP000642697:Unassembled WGS sequenceMKFIIKLFPEITIKSQSVRLRFIKMLTGNIRNVLKHYDETLAVVRHWDNIEVRAKDENQRLAIRDALTRIPGIHHILEVEDVPFTDMHDIFEKALVQYRDQLEGKTFCVRVKRRGKHEFSSIDVERYVGGGLNQHIESARVKLTNPDVTVHLEVEDDRLLLIKGRYEGIGGFPIGTQEDVLSLISGGFDSGVSSYMLMRRGCRVHYCFFNLGGAAHEIGVRQVAHYLWNRFGSSHRVRFVAINFEPVVGEILEKVDDGQMGVVLKRMMVRAASKVAERYGVQALVTGEALGQVSSQTLTNLRLIDNVSDTLILRPLISYDKEHIINLARQIGTEDFARTMPEYCGVISKSPTVKAIKAKIEAEEQNFDFSILDKVVEEASNIDIRDIAQQTQQDVVEVETVSGFGPNDVILDIRSIDEQDDKPLKVEGVDVVSLPFYKLSTQFGDLDQNKTWLLWCERGVMSRLQALYLREQGFENVKVYRP>UPI00049ECD71 Maltose/maltodextrin ABC transporter substrate-binding protein MalE OS=Citrobacter amalonaticus OX=35703 GN=malE SS=EMBLWGS:MBE0398450 PC=UP000642697:Unassembled WGS sequenceMKIKTGARVFALSALAAMMISAPALAKIEEGKLVIWINGDKGYNGLAEVGKKFEKDTGIKVTIEHPDKLEEKFPQVAATGDGPDIIFWAHDRFGGYAQSGLLAEITPDKAFQDKLYPFTWDAVRYNGKLIAYPIAVEALSLIYNKDLVANPPKTWEEIPALDKELKAKGKSALMFNLQEPYFTWPLIAADGGYAFKFENGTYDVKDVGVDSAGAKAGLGFLVDLIKNKHMNADTDYSIAEAAFNKGDTAMTINGPWAWTNIDKSKVNYGVTLLPTFKGKPSKPFVGVLSAGINAASPNKELAKEFLENYLLTDQGLEEVNKDKPLGAVALKSYQDQLAKDPRIAATMDNAQKGEIMPNIPQMSAFWYAVRTAVINAASGRQTVDAALKDAQGRITK>UPI00049ECE1A Phosphoserine phosphatase OS=Citrobacter amalonaticus OX=35703 GN=serB SS=EMBLWGS:MBE0394215 PC=UP000642697:Unassembled WGS sequenceMPNITWCDLPEDVSLWPGLPLSLSGDEVMPLDYHAGRSGWLLYGRGLDKQRLTQYQSKLGAAMVIVAAWCVEDYQVIRLAGSLTPRATKLAHDAQLDVAPLGKIPHLRTPGLLVMDMDSTAIQIECIDEIAKLAGTGERVAEVTERAMRGELDFTASLRSRVATLKGADANILHQVRESLPLMPGLTQLVLKLETLGWKVAIASGGFTFFAEYLREKLRLTAAVANELEIMDGKFTGNVIGDIVDAQYKAKTLTRLAQEYEIPIAQTVAIGDGANDLPMIKTAGLGIAYHAKPKVNEKTEITIRHADLMGVFCILSGSMNQK>UPI00049ECF03 D-ribose ABC transporter substrate-binding protein OS=Citrobacter amalonaticus OX=35703 GN=ILP74_15165 SS=EMBLWGS:MBE0396745 PC=UP000642697:Unassembled WGS sequenceMKLRLTLLTAATLTALSFSAHAAEKGTIMIMVNSLDNPYYASEAKGASEKAQELGYKTTILSHSEDVKKQNELIDTAIGKKVQGIILDNADSTASVAAIEKAKKAGIPVILINREIPVDDVALVQITHNNFQAGSEVANVFVEKMGEKGKYAELTCNLADNNCVTRSKSFHQVIDQFPDMVSVARQDAKGTLIDGKRIMDSILQAHPDVKGVICGNGPVALGAIAALKAANRNDVVVVGIDGSNDERDAVKAGTLQATVMLQAQAIAAQGVTDLDNYLQKGVKPEKQRVMFRGILITKDNADKVQDFNIKS>UPI00049ECFA2 VOC family metalloprotein YjdN OS=Citrobacter amalonaticus OX=35703 GN=yjdN SS=EMBLWGS:MBE0393966 PC=UP000642697:Unassembled WGS sequenceMSLSPYISFAGNCADAIAYYQKTLGAELLYKISFGEMPKPAQDSEDGCPSGMTFPDTAIAHANVRIADSDIMMSDSIPTGNAHYSGFTLVLDTQNVNEGKQWFDNLAAEGQIEMDWQETFWAHGFGKVSDRYGVPWMINVVKQPQPTE>UPI00049ECFD8 Environmental stress-induced protein Ves OS=Citrobacter amalonaticus OX=35703 GN=ves SS=EMBLWGS:MBE0396108 PC=UP000642697:Unassembled WGS sequenceMEYFDIRKMPVSLWRNGAGETREICCFPPATRDFHWRASIASIAGNGEFSLFPGIERVITLLEGSEVTLESQGAFSHTLKQHQPFTFAGERVVKARLTEGQMSMDFNIMTRRDSCQAKVRVADRTFTTFGSRGGVVFVISGAWQLGDKLLTADQGACWHDGKHTLRLLKAEGKLLFSEITWLQGYSPDSVQ>UPI00049ECFE7 Microcin C ABC transporter permease YejB OS=Citrobacter amalonaticus OX=35703 GN=ILP74_06300 SS=EMBLWGS:MBE0395087 PC=UP000642697:Unassembled WGS sequenceMGAYLIRRLLLVIPTLWAIITINFFIVQIAPGGPVDQAIAAIEFGQTSALPGAGSEGVRASHARTGVGNISDSNYRGGRGLDPEVIAEITQRYGFDKPLHERYFTMLWNYIRFDFGDSLFRSASVLTLIKESLPVSITLGLWSTLIIYLVSIPLGIRKAVHNGSRFDVWSSAFIIIGYAIPAFLFAILLIVFFAGGSYYDLFPLRGLVSANFDTLPWYQKVTDYLWHITLPVLATVIGGFAALTMLTKNSFLDEVRKQYVVTARAKGVSEGNILWKHVFRNAMLLVIAGFPATFISMFFTGSLLIEVMFSLNGLGLLGYEATVSRDYPVMFGTLYIFTLIGLLLNILSDISYTLVDPRIDFEGR>UPI00049ED070 DUF1471 domain-containing protein OS=Citrobacter amalonaticus OX=35703 GN=ILP74_11000 SS=EMBLWGS:MBE0395966 PC=UP000642697:Unassembled WGS sequenceMKLKNTLLASALLSATAFSVNAATELTPEQAAALKPYDRVTVTGRFNAIGDAVKAVNRRADKEGAASFYVVDTSDFGNSGNWRVVADFYKADAEKAEASKNRVINGVVELPKSEAVMLEPYDTVTVQGFYRSQPEVNDAITKAAKEKDAYAFYIVRQIDANQGGNQRITAFIYKKDAKKRVVQSPDAIPADSDAGRAALAAGGEAAKNVEIPGVATTASPSSEVGRFFETQSTKGGRYTVTLPDGTKVEELNKATAAMMVPFDSIKFTGNYSSMTEVSYQVAKRAAKQGAKYYHITRQWQERGNNMTISADLYK>UPI00049ED07C DNA repair protein RadC OS=Citrobacter amalonaticus OX=35703 GN=radC SS=EMBLWGS:MBE0397969 PC=UP000642697:Unassembled WGS sequenceMETAEYVLPREKLLALGVDTLTDDELLALFLRTGTPGKDVFALAKEILEHFGSLYSLLTADYSQFSAVTGIGVAKYAQLKGIAELARRFYEVQIKGTNPLLSPVLTRDFLQSQLAGEAREVFMVVFLDSQHHVIKHSRLFSGTLTHVEIHPREIIREAIKINASAVILAHNHPSGCAEPSKADKLITERVVKCCQFMDIRVLDHLVIGRGEYVSFAERGWI>UPI00049ED086 Sulfurtransferase complex subunit TusB OS=Citrobacter amalonaticus OX=35703 GN=tusB SS=EMBLWGS:MBE0397706 PC=UP000642697:Unassembled WGS sequenceMLHTLHRSAWHSDFSAILRLLAEGDELLLLQDGVTAAVEGCRFLESLQNTPITVYALKEDIDARGLGGQISDSVVRVDYTDFVRLTVKHASQMAW>UPI00049ED0E6 YtjB family periplasmic protein OS=Citrobacter amalonaticus OX=35703 GN=ILP74_01600 SS=EMBLWGS:MBE0394214 PC=UP000642697:Unassembled WGS sequenceMARAKLKFRLHRAVIVLFCLALLVALMQGASWFSQNHQRQRNPQLEELARTLAHQVTLNIAPLMRTETPDEKRIKTILQQLTKESRILDAGVYDEQGDMIARAGESVNVRDRLALDGKKAGGYFNQQIVEPIQGKNGPLGYLRLTLDTHTLATEARQVDNTTNILRLMLLLSLAIGVVLTRTLLQGKRTRWQQSPFLLTANKSVPEEEESEKKESFVTGKEN>UPI00049ED292 DNA-binding protein HU-beta OS=Citrobacter amalonaticus OX=35703 GN=hupB SS=EMBLWGS:MBE0396861 PC=UP000642697:Unassembled WGS sequenceMNKSQLIDKIAAGADISKAAAGRALDAIIASVTESLKEGDDVALVGFGTFAVKERAARTGRNPQTGKEIAIAAAKVPGFRAGKALKDAVN>UPI00049ED33A Oxygen-insensitive NAD(P)H nitroreductase OS=Citrobacter amalonaticus OX=35703 GN=nfsB SS=EMBLWGS:MBE0396762 PC=UP000642697:Unassembled WGS sequenceMDIVSVALKRHSTKAFDPAKKLTAEEAEKIKTLLQFSPSSTNSQPWHFIVASTEEGKARVAKSAAGNFVFNERKMLDASHVVVFCAKTAMDDAWLERVVDQEEADGRFASAEAKAANHKGRTFFADMHRKELKDDAHWMAKQVYLNVGNFLLGVAALGLDAVPIEGFDAAILDAEFGLKEKGFTSVVVVPVGHHSVEDFNASLPKSRLPLSTIITEC>UPI00049ED3A1 Cytochrome c-type protein NapC OS=Citrobacter amalonaticus OX=35703 GN=napC SS=EMBLWGS:MBE0395011 PC=UP000642697:Unassembled WGS sequenceMENANRKPGRIKRLWQWWRRPSRLALGTLLLIGFVGGIIFWGGFNTGMEKANTEEFCISCHEMRNTVYQEYMETVHYNNRSGVRATCPDCHVPHEFVPKMIRKIKASKELYAKALGLIDTPQKFEAHRLTMAQNEWRRMKDNNSQECRNCHNFDFMDLTAQKSVAAKMHDQAVKDGQTCIDCHKGIAHKLPDMRDVKPGF>UPI00049ED4AF 50S ribosomal protein L25 OS=Citrobacter amalonaticus OX=35703 GN=rplY SS=EMBLWGS:MBE0395081 PC=UP000642697:Unassembled WGS sequenceMFTINAEVRKEQGKGASRRLRAANKFPAIIYGGKEAPIAIELDHDSIMNMQAKEGFYSDVLTIVVDGKEVKVKAQAVQRHAFKPKLTHIDFVRA>UPI00049ED503 Sigma-54-dependent transcriptional regulator OS=Citrobacter amalonaticus OX=35703 GN=ILP74_01845 SS=EMBLWGS:MBE0394260 PC=UP000642697:Unassembled WGS sequenceMVLATTQSILMQIQPTIQRFARMLASVLQLEVEIVDDNLCRVAGTGAYGKYLGRPLSGNSRLLRYVLESKKEKVVTHSRFDPLCEGCDSKDNCREKAFLGTPVILQDRCVGVISLIAVTHEQQEHINDNLREFSDYVRHISTIFVSKLLEDQSGSDNISKIFSTMIENMDQGVLVIDEDNRVQFANQTALKILGAVQNNMVGKSVRFRPLTFESNFTHGHMQHIVSWDDKSELIIGQLHHVQGRQLFLMAFHQSHTSSSVSIATDEPHIEQLVGECRVMRQLKRLIGRIAPSPSSVMIVGESGTGKEVVARAIHKLSDRRNKPFIAINCAAIPEQLLESELFGYVKGAFTGASANGKTGLIQAANSGTLFLDEIGDMPLMLQAKLLRAIEAREVLPLGASSPVQVDIRIISATNQNLGQFIAEGKFREDLFYRLNVIPLTLPPLRERQDDIELLVHYFLHLHTRRLGLVYPGIAPDVVTLLRHHQWPGNLRELSNLMEYLVNVVPSGEVIDSTLLPPNLINNGKAPESVGAVAHAAQLLSEDTGGTALEEMEKQMIREALSRHSNKKQVADELGIGIATLYRKIKKYELLNA>UPI00049ED5E8 Glycine C-acetyltransferase OS=Citrobacter amalonaticus OX=35703 GN=kbl SS=EMBLWGS:MBE0397949 PC=UP000642697:Unassembled WGS sequenceMRGDFYKQLTNDLQTARAEGLFKEERIITSAQQADITVADGSHVINFCANNYLGLANHPELIAAAKAGMDSHGFGMASVRFICGTQDSHKQLEQKLAEFLGMEDAILYSSCFDANGGLFETLLGAEDAIISDALNHASIIDGVRLCKAKRFRYANNDMQELEARLQEARDAGARHVLIATDGVFSMDGVIANLKGVCDLADKYNALVMVDDSHAVGFVGENGRGSHEYCDVMGRVDIITGTLGKALGGASGGYTAARKEVVEWLRQRSRPYLFSNSLAPAIVAASIKVLEMVESGGELRDRLWANARQFREQMSAAGFTLAGADHAIIPVMLGDAVIAQDFARELQKEGIYVTGFFYPVVPKGQARIRTQMSAAHTPEQITRAVEAFTRIGKQLGVIA>UPI00049ED65B PTS sorbose transporter subunit IIA OS=Citrobacter amalonaticus OX=35703 GN=ILP74_24195 SS=EMBLWGS:MBE0398425 PC=UP000642697:Unassembled WGS sequenceMVNAIFCAHGKLACAMLESVHMVYGDANVEAVAFVPGENASDIVTKLEKLVSAHTDSEWLIAVDLQCGSPWNAAATLAMRNPAIRVISGLSLPLALELVDNQSSMNVDELCEHLTTIAQQTCVVWQHLETTEEDF>UPI00049ED7B0 Quaternary ammonium compound efflux SMR transporter SugE OS=Citrobacter amalonaticus OX=35703 GN=sugE SS=EMBLWGS:MBE0394037 PC=UP000642697:Unassembled WGS sequenceMSWIILLIAGLLEVVWAVGLKYTHGFSRLTPSIITIAAMIVSMALLAWAMKTLPVGTAYAVWTGIGAVGAAITGILLLGESANPMRLASLALIVVGIIGLKLSTH>UPI00049ED88D Non-heme ferritin OS=Citrobacter amalonaticus OX=35703 GN=ftnA SS=EMBLWGS:MBE0395492 PC=UP000642697:Unassembled WGS sequenceMLKTEMIDKLNEQMNLELYSSLLYQQMSAWCSYHSFEGAAAFLRRHAQEEMTHMQRLFDYLTDTGSLPRINTVSSPFAEYASLDELFRVTYEHEQLITQKINELAHAAMTSQDYPTFNFLQWYVAEQHEEEKLFKSVIDKLTLAGKSGEGLYFIDKELSTLDTAN>UPI00049ED93C Superoxide response transcriptional regulator SoxS OS=Citrobacter amalonaticus OX=35703 GN=soxS SS=EMBLWGS:MBE0393930 PC=UP000642697:Unassembled WGS sequenceMSHQQIIQTLIEWIDEHIDQPLNIDVVAKKSGYSKWYLQRMFRTVMRQTLGDYIRQRRLLLAAVELRNTERPIFDIAMDLGYVSQQTFSRVFRREFDRTPSDYRHRL>UPI00049ED940 IclR family transcriptional regulator OS=Citrobacter amalonaticus OX=35703 GN=ILP74_05670 SS=EMBLWGS:MBE0394967 PC=UP000642697:Unassembled WGS sequenceMLESSKVPALTRAIDILNLIARIGPCSAATIIETLGIPKSTAYLLLSELKRQRFISVDHQENFCLWTKLVELSGHALSKMDLRELARPRLTQLMDETGLLCHLGIIDHESAYYILKVESSSTISVRSHEGKSLSLYRSGIGKCLLAWQPAAVRNAIIEQLVWEQATPTTITQPQQLSDELERIRQRGWSFDNGEDYPDVRCVAAPVFNANNEPAAAISVVGTRLEINEENRDYLAGKAIACAKDISRLLGWKSPFDSLAS>UPI00049ED9C1 Excinuclease ABC subunit B OS=Citrobacter amalonaticus OX=35703 GN=uvrB SS=EMBLWGS:MBE0396553 PC=UP000642697:Unassembled WGS sequenceMSKPFKLNSAFKPSGDQPEAIRRLEEGLEDGLAHQTLLGVTGSGKTFTIANVIADLQRPTMVLAPNKTLAAQLYGEMKEFFPDNAVEYFVSYYDYYQPEAYVPSSDTFIEKDASVNEHIEQMRLSATKALLERRDVIVVASVSAIYGLGDPDLYLKMMLHLTVGMIIDQRAILRRLAELQYTRNDQAFQRGTFRVRGEVIDIFPAESDDIALRVELFDEEVERLSLFDPLTGQVESTISRYTIYPKTHYVTPRERIVQAMEEIKVELAERRKILLANDKLLEEQRLSQRTQFDLEMMNELGYCSGIENYSRFLSGRGPGEPPPTLFDYLPADGLLVVDESHVTIPQIGGMYRGDRARKETLVEYGFRLPSALDNRPLKFEEFEALAPQTIYVSATPGNYELEKSGGEVVDQVVRPTGLLDPVIEVRPVATQVDDLLSEIRKRAEINERVLVTTLTKRMAEDLTEYLEEHGERVRYLHSDIDTVERMEIIRDLRLGEFDVLVGINLLREGLDMPEVSLVAILDADKEGFLRSERSLIQTIGRAARNINGKAILYGDKITASMAKAIGETERRREKQQQYNEEHGITPQGLNKKVVDILALGQNIAKTKAKGRGKSRSAVQSDVVELDMTPKALQQKIHELEGQMMQHAQNLEFEEAAQIRDQLHQLRELFIAAS>UPI00049ED9D7 Uncharacterized protein OS=Citrobacter amalonaticus OX=35703 GN=ILP74_15680 SS=EMBLWGS:MBE0396843 PC=UP000642697:Unassembled WGS sequenceMGRRLVQLYFGLALYGVSTAMFVRADLGADPWNVFHLGLANLLSMKIGVVMIIVGALVLLLWIPLRQRPGLGTLSNVIVIGLAADAALAMIPTFSSLAVRSLMLVAAVVVNALATGMYIGAGFGAGPRDGLMTGIHARTGWSVRTIRTAIEVSVLLSGWLLGGTLGVGTVLYALAIGPLIQICLPWFRYKPRARIQTA>UPI00049EDA8A 30S ribosomal protein S12 methylthiotransferase RimO OS=Citrobacter amalonaticus OX=35703 GN=rimO SS=EMBLWGS:MBE0396459 PC=UP000642697:Unassembled WGS sequenceMSKVTHQPKIGFVSLGCPKNLVDSERILTELRTEGYDVVPSYDDADMVIVNTCGFIDSAVQESLEAIGEALNENGKVIVTGCLGAKEDQIREVHPKVLEITGPHSYEQVLEHVHHYVPKPKHNPFLSLVPEQGVKLTPRHYAYLKISEGCNHRCTFCIIPSMRGDLVSRPIGDVLSEAKRLVDAGVKEILVISQDTSAYGVDVKHRTGFYNGEPVKTSMVSLCEQLSKLGIWTRLHYVYPYPHVDDVIPLMAEGKILPYLDIPLQHASPRILKLMKRPGSVDRQLARIKQWREICPELTLRSTFIVGFPGETEDDFQMLLDFLKEARLDRVGCFKYSPVEGAGANALPDQVPEEVKEERWNRFMQLQQQISAERLQEKVGREILVIVDEVDEEGAIGRSMADAPEIDGAVYLNGETNVKPGDVIRVKVENADEYDLWGTRA>UPI00049EDABA 1-phosphofructokinase OS=Citrobacter amalonaticus OX=35703 GN=fruK SS=EMBLWGS:MBE0395099 PC=UP000642697:Unassembled WGS sequenceMSRRVATITLNPAYDLVGFCPEIERGEVNLVKTTGLHAAGKGINVAKVLKDLGIDVTVGGFLGKDNQDGFQQLFSELGIANRFQVVQGRTRINVKLTEKDGEVTDFNFSGFEVTPADWERFVNDSLSWLGQFDMVCVSGSLPSGVSPEAFTDWMTRLRSQCPCIIFDSSREALVAGLKAAPWLVKPNRRELEIWAGRKLPEMKDVIEAAHALREQGIAHVVISLGAEGALWVNASGEWLAKPPSVDVVSTVGAGDSMVGGLIYGLLMRESSEHTLRLATAVAALAVSQSNVGITDRPQLAAMMSRVDLQPFN>UPI00049EDB31 MFS transporter OS=Citrobacter amalonaticus OX=35703 GN=ILP74_23035 SS=EMBLWGS:MBE0398213 PC=UP000642697:Unassembled WGS sequenceMSQNITNPATLRLPFKEKLAYGMGDLGSNILLDIGTLYLLKFYTDVLGLPGTYGGIIFLIAKFFTAFTDMGTGIMLDSRRKIGPKGKFRPFVMYAAFPVTLLAIANFVGTPFEITGKTVMATVLFMLYGLFFSMMNCSYGAMVPAITKNPDERASLAAWRQGGATLGLLLCTVGFVPVMNLIEGNSQLGYIFAATLFSLFGLFFMWCCYAGVKERYVEVKPVDAAQKPGLLQSFRAIAGNRPLFILCIANLCTLGAFNVKLAIQVYYTQYVLNDPILLSWMGFFSMGCIFIGVFLMPGMVRRFGKKKVYIGGLLIWVAGDLLNYLFGGGSVSFVAFSCLAFFGSAFVNSLNWALVSDTVEYGEWRTGVRSEGTVYTGFTFFRKVSQALAGFFPGWMLTQIGYVPNVVQSAGTVEGLRQLIFIYPCALAVVTIIAMGCFYNLNEKMYIRIVGEIEARKQTI>UPI00049EDB7B Stationary-phase-induced ribosome-associated protein OS=Citrobacter amalonaticus OX=35703 GN=sra SS=EMBLWGS:MBE0395899 PC=UP000642697:Unassembled WGS sequenceMKSNRQARHILGLDHKISNQRKVVTEGDKSSVVNNPTGRKRHADSKK>UPI00049EDBB1 YaeP family protein OS=Citrobacter amalonaticus OX=35703 GN=ILP74_16790 SS=EMBLWGS:MBE0397037 PC=UP000642697:Unassembled WGS sequenceMEKYCELIRKRYAEIASGDLGYIPDALGCVLKVLNEIAADSALSESVREKAAYAAANLLVSDYVNE>UPI00049EDC74 UvrD-helicase domain-containing protein OS=Citrobacter amalonaticus OX=35703 GN=ILP74_18080 SS=EMBLWGS:MBE0397279 PC=UP000642697:Unassembled WGS sequenceMTWKTTAEQNAIIEWKGNHLVVNAFAGTGKTSTLVNYAEANPESKMLYLAYNRAVRDEAERKFPFNVECKTSHQLAWARFGRHFRDRLTASLRITDVARKLNTRHWPLARLALSGLNMFLCSADPEPGMIHLPSEDDRHGLDAGKILGAIQILWYEMSRTDSVFPVTHDTYLKLFQLSHPDLSKRWDTILFDEAQDANPVTSVFVLNQPCRVILVGDRYQQIYRFRGADNALNAPQLAQADRLWLTASFRFGPEVARMANILLERAGEEKRVTGNGGQDAVVSSIPDEAEHVAVLSRTVSGVIGSALTASLMEKKVFWVGGIEGYKTEELEDLYWFSADMPVKMQSPRLSRDYRDFDEYCSIAKATQDVEMNQAIRLLDDFFPLPQKLAIMRRQVVTHEKDAQVTVSTAHRSKGLEWPVVMLSEDFTDITDPLLSEDERQDETNLLYVAVTRARRTLVLNELMRWLSDEVGKNRETTYETVPSGNGESADRHEETGKTSESE>UPI00049EDCAC PTS sugar transporter subunit IIA OS=Citrobacter amalonaticus OX=35703 GN=ILP74_21980 SS=EMBLWGS:MBE0398023 PC=UP000642697:Unassembled WGS sequenceMTTTQPLPDILLLTHGGWGQQLCNSLRMVMGEIKGVTEIALMPVDTLGEFYQRVEDVVKTMPEGSLILTDFVGGTTSNVAARLSADYPVAVVSGLNASLLLEALDRREHGSLTTCVSELVDAGRSSCLDVVAHVRQLQQSQ>UPI00049EDD06 Bifunctional dihydroneopterin aldolase/7,8-dihydroneopterin epimerase OS=Citrobacter amalonaticus OX=35703 GN=folB SS=EMBLWGS:MBE0397427 PC=UP000642697:Unassembled WGS sequenceMDIVFIEQLSVITTIGVYDWEQTIEQKLVFDIEMAWDNRKSAKSDDVADCLSYADIAETIVNHVEGGRFALVERVAEEVAELLLTRFNSPWVRIKLSKPGAVARAANVGVIIERSNNLKEK>UPI00049EDD09 Serine hydroxymethyltransferase OS=Citrobacter amalonaticus OX=35703 GN=glyA SS=EMBLWGS:MBE0394627 PC=UP000642697:Unassembled WGS sequenceMLKREMNIADYDAELWQAMEQEKVRQEEHIELIASENYTSPRVMQAQGSQLTNKYAEGYPGKRYYGGCEYVDIVEQLAIDRAKELFGADYANVQPHSGSQANFAVYTALLQPGDTVLGMNLAQGGHLTHGSPVNFSGKLYNIVPYGIDESGKIDYEEMAQLAQTHKPKMIIGGFSAYSGVVDWAKMREIADSIGAYLFVDMAHVAGLIAAGVYPNPVPHAHVVTTTTHKTLAGPRGGLILAKGGDEELYKKLNSAVFPSAQGGPLMHVIAGKAVALKEAMEPEFKVYQQQVAKNAKAMVEVFLNRGYKVVSGGTENHLFLLDLVDKNLTGKEADAALGRANITVNKNSVPNDPKSPFVTSGIRIGSPAITRRGFKEAEAKELAGWMCDVLDNINDEAVIERIKGKVLDICARFPVYA>UPI00049EDD29 YehR family lipoprotein OS=Citrobacter amalonaticus OX=35703 GN=ILP74_06595 SS=EMBLWGS:MBE0395144 PC=UP000642697:Unassembled WGS sequenceMKALNTFFSVVFASVLVFSLAGCGDKEESKTFKADVNGTEIVMTYTYKGDKVIKQTSENKINYASIGVKTKEDAAKILDPISEKYQNIPGVEEKLTYKDGYAEETVSVDMEKVDFKKLQGVMGTQFSGDPSNGISMKQSQKMLETAGFKEVK>UPI00049EDD53 Beta-phosphoglucomutase OS=Citrobacter amalonaticus OX=35703 GN=pgmB SS=EMBLWGS:MBE0395754 PC=UP000642697:Unassembled WGS sequenceMKPQAIVFDLDGVITDTAHLHFLAWRQVAAELGIVIDEVFNDRLKGISRGESLLRILRHGGKEGVFTQAEREQIAARKNALYVDSLRSLTPEAVLPGIKELLTTLRTEQIKTGLASVSLNAPAILQALQMTSLFDFCANAALIRRSKPDPEIFLAACNGLHVNPRACIGIEDAQAGIEAINASGMRSVGIGSTLTDADLLLPTTEFLTWPRLSAFWQLDK>UPI00049EDF83 LacI family DNA-binding transcriptional regulator OS=Citrobacter amalonaticus OX=35703 GN=ILP74_01040 SS=EMBLWGS:MBE0394107 PC=UP000642697:Unassembled WGS sequenceMRNHRISLQDIATLAGVTKMTVSRYIRSPKKVAKETGERIAQIMEEINYIPNRAPAMLLNAQSYTLGVLIPSFQNQLFADILAGIESVTSDHNYQTLIANYNYDRESEEESVINLLSYNIDGIILSEKYHTLRTVKFLRSAAIPIIELMDIQGDRLDMEVGFDNRQAAFDMVSTMLDKRQRRKILYLGSKDDIRDEQRFRGYCDAMTRRGLTPLRVNPKAISSIRLGMQLMRDALIAHPDLDGVFCTNDDIAMGALLFCRERDLSVPEQVSIAGFHGLEMGRQMIPSLASVITPRFDIGRMAAQMLLSKIKNNDHNHNTIDLGYQIYHGNTL>UPI00049EDFA5 Xanthine/proton symporter XanP OS=Citrobacter amalonaticus OX=35703 GN=xanP SS=EMBLWGS:MBE0397984 PC=UP000642697:Unassembled WGS sequenceMSVNTLESENAQPVAQTQNSELIYRLEDRPPLPQTLFAACQHLLAMFVAVITPALLICQALGLPAQDTQHIISMSLFASGVASIIQIKAWGPVGSGLLSIQGTSFNFVAPLIMGGTALKTGGADVPTMMAALFGTLMLASCTEMVLSRILHLARRIITPLVSGVVVMIIGLSLIQVGLTSIGGGYAAMSDNTFGAPKNLMLAGVVLLLIILLNRQRNPYLRVASLVIAMAAGYALAWFMGMLPENNAPASQDLIMVPTPLYYGLGIDWNLLLPLMLVFMITSLETIGDITATSDVSEQPVSGPLYMKRLKGGVLANGLNSFVSAVFNTFPNSCFGQNNGVIQLTGVASRYVGFVVALMLIVLGLFPAVSGFVQHIPEPVLGGATLVMFGTIAASGVRIVSREPLNRRAILIIALSLAVGLGVSQQPLILQFAPDWVKNLLSSGIAAGGITAIVLNLIFPPEKQ>UPI00049EDFC0 Carbohydrate porin OS=Citrobacter amalonaticus OX=35703 GN=ILP74_22025 SS=EMBLWGS:MBE0398031 PC=UP000642697:Unassembled WGS sequenceMNMIKKLPLAMAVVAALCPISVLAQEFTQEQIDAIVAKAVDKALAERQAKMDAAVAKKTDVIVEPQSAAQSPDMAIPFGVKFSGYARYGAHFQSGDQKYVGVDGSYNGASAIGRLGNEGNGGEFQLSKAFKGDNGAIWDINVMIDHWGDEVNLKKAYAGVTNVLESNPNAYIWAGRDFHQRPQQGINDYFWMNHDGQGAGVKNFDIGGVQFDVATVAAVESCSPEVMEDEANPSRITCTGGSGTGDKGNYAVTSKIHGMKLGPLDLEIYANYGFDSKAVDSDDRLKAWQGAFVVSHTNDSGVNKVIARYSDNSDNSVYNKTDDLTAIYASFEGSHKFTQQAQVEYLLAFHDYDNSADQSENRKNYGAIVRPMYFWNDVHSTWLEAGYQRVDYDNGGDNKGWKLTLSQNMSIAMGPEFRPMLRFYVTGGKVDNDRTARVNGTDDETLDDFNVGAMWEAWF>UPI00049EE02A Peptidoglycan glycosyltransferase MrdB OS=Citrobacter amalonaticus OX=35703 GN=mrdB SS=EMBLWGS:MBE0396692 PC=UP000642697:Unassembled WGS sequenceMTDNPNKKTFWDKIHIDPTMLLILLALLVYSSLVIWSASGQDIGMMERKVGQITMGLVIMVVMAQIPPRVYEGWAPYLYIFCIILLVAVDAFGAISKGAQRWLDLGIVRFQPSEIAKIAVPLMVARFINRDVCPPSLKNTAIALVLIFMPTLLVAAQPDLGTSILVALSGLFVLFLSGLSWRLIGVAVLLLAAFIPILWFFLMHDYQRQRVMMLLDPETDPLGAGYHIIQSKIAIGSGGLSGKGWLHGTQSQLEFLPERHTDFIFAVLAEELGLIGILILLALYILLIMRGLWIAAHAQTTFGRVMAGGLMLILFVYVFVNIGMVSGILPVVGVPLPLVSYGGSALIVLMAGFGIVMSIHTHRKMLSKSV>UPI00049EE061 Glutaredoxin-dependent arsenate reductase OS=Citrobacter amalonaticus OX=35703 GN=arsC SS=EMBLWGS:MBE0394316 PC=UP000642697:Unassembled WGS sequenceMSNITIYHNPACGTSRNTLEMIRNSGNEPTVIHYLETPPSRDELVKLIADMGISVRALLRKNVEPYEELGLAEDKFTDNQIIDFMLQHPILINRPIVVTPLGTKLCRPSEVVLDILPDAQKAAFAKEDGEKVVDETGKRLK>UPI00049EE088 Murein tripeptide/oligopeptide ABC transporter ATP binding protein OppD OS=Citrobacter amalonaticus OX=35703 GN=oppD SS=EMBLWGS:MBE0395690 PC=UP000642697:Unassembled WGS sequenceMSVIETAGVPFAQQRANALLDVKDLRVTFSTPDGDVTAVNDLNFTLRAGETLGIVGESGSGKSQTAFALMGLLAANGRIGGSATFSGREILNLPEHELNKLRAEQISMIFQDPMTSLNPYMRVGEQLMEVLMLHKSMSKAEAFEESVRMLDAVKMPEARKRMKMFPHEFSGGMRQRVMIAMALLCRPKLLIADEPTTALDVTVQAQIMTLLNELKREFNTAIIMITHDLGVVAGICDKVLVMYAGRTMEYGNARDVFYQPVHPYSIGLLNAVPRLDGEGDEMLTIPGNPPNLLRLPKGCPFQPRCPHAMEICSTTPPLEEFSPGRLRACFKPVEDLA>UPI00049EE0D4 NupC/NupG family nucleoside CNT transporter OS=Citrobacter amalonaticus OX=35703 GN=ILP74_06380 SS=EMBLWGS:MBE0395103 PC=UP000642697:Unassembled WGS sequenceMDIMRSVVGMAVLLVIAYLLSVNKKHISLRTVGAALVLQIAIGGIMLYFPPGKWLVEQAALGVHKVMSYSDAGSAFIFGSLVGPKMDVLFDGAGFIFAFRVLPAIIFVTALISLLYYIGVMGLLIRILGGIFQKALNISKIESFVAVTTIFLGQNEIPAIVKPFIDRLNRNELFTAICSGMASIAGSMMIGYAGMGVPIDYLLAASLMAIPGGILFARILSPATEESKVTFENLSFTETPPKSIIEAAASGAMTGLKIAAGVATVVMAFVAIIALLNGIIGGIGGWFGYGHATLEGIFGWVLAPLAWIMGVDWSDATLAGSLIGQKLAINEFVAYLNLSPYLQDGGTLDVKTIAIISFALCGFANFGSIGVVVGAFSAISPQRAPEIAQLGMRALAAATLSNLMSATIAGFFIGLA>UPI00049EE10A Threonine--tRNA ligase OS=Citrobacter amalonaticus OX=35703 GN=thrS SS=EMBLWGS:MBE0396086 PC=UP000642697:Unassembled WGS sequenceMPVITLPDGSQRHYDHAVSPMDVALDIGPGLAKATLAGRVNGELVDASDLIENDATLSIITAKDEEGLEIIRHSCAHLLGHAIKQLWPNTKMAIGPVVDNGFYYDVDLDRTLTQEDIDALEKRMHELAEKNYDVIKKKVSWHEARETFVKRGEIYKVSILDENIAHDDKPGLYHHEEYVDMCRGPHVPNMRFCHHFKLMKTAGAYWRGDSDNKMLQRIYGTAWADKKALSAYLQRLEEAAKRDHRKIGKQLDLYHMQEEAPGMVFWHNDGWTIFRELEVFVRSKLKEYQYQEVKGPFMMDRVLWEKTGHWDNYKDAMFTTSSENREYCIKPMNCPGHVQIFNQGLKSYRDLPLRMAEFGSCHRNEPSGALHGLMRVRGFTQDDAHIFCTEEQIRDEVNACIRMVYDMYSTFGFEKIVVKLSTRPEKRIGSDEMWDRAEADLAVALEENNIPFEYQLGEGAFYGPKIEFTLYDCLDRAWQCGTVQLDFSLPSRLSASYVGENNERQVPVMIHRAILGSMERFIGILTEEFAGFFPTWLAPVQVVVMNITDSQSEYVNELTQKLQNAGIRVKADLRNEKIGFKIREHTLRRVPYMLVCGDKEVEAGKVAVRTRRGKDLGSLDVSEVIEKLQQEIRSRSLQQLEE>UPI00049EE1C4 TRAP transporter small permease OS=Citrobacter amalonaticus OX=35703 GN=ILP74_01180 SS=EMBLWGS:MBE0394134 PC=UP000642697:Unassembled WGS sequenceMGEGYSSVMDVLYRISMWIAGLALLVMVAVIPVGIFARYVMNSALSWPEPIAILCMVTFTFIGAAVSYRAGSHIAVSMVTDRLGEMGRRICFIGADLMLLAISIFILWYGSTLCYELWQQPVAEFPILTAGENYLPLPIGSAIMLLFIIEKICRGAQYQRPVVMLGSTS>UPI00049EE1C7 Nucleoside diphosphate kinase regulator OS=Citrobacter amalonaticus OX=35703 GN=rnk SS=EMBLWGS:MBE0396717 PC=UP000642697:Unassembled WGS sequenceMSRPTIIINDLDAERIDRLLEQPAYADLPIADALNAELDRAQMCSPETMPHDVVTMNSRVKFRNLSDGEIRVRTLVYPAAMTDSSTQLSVMAPVGAALLGVRVGDTIHWELPGGVSTHLEVLELEYQPEAAGDFLR>UPI00049EE1D2 Maltose/maltodextrin ABC transporter ATP-binding protein MalK OS=Citrobacter amalonaticus OX=35703 GN=malK SS=EMBLWGS:MBE0398451 PC=UP000642697:Unassembled WGS sequenceMASVQLRNVTKAWGDVVVSKDINLDIHEGEFVVFVGPSGCGKSTLLRMIAGLETITSGDLYIGETRMNDIPPAERGVGMVFQSYALYPHLSVAENMSFGLKLAGAKKEVMNQRVNQVAEVLQLAHLLERKPKALSGGQRQRVAIGRTLVAEPRVFLLDEPLSNLDAALRVQMRIEISRLHKRLGRTMIYVTHDQVEAMTLADKIVVLDAGRVAQIGKPLELYHYPADRFVAGFIGSPKMNFLPVKVTATAIDQVQVELPNRQHVWLPVDSRDVQVGVNMSLGIRPEHLLPSDIADVTLEGEVQVVEQLGHETQIHIQIPAIRQNLVYRQNDVVLVEEGATFAIGLPPERCHLFREDGTACRRLHKEPGV>UPI00049EE29E Bis(5'-nucleosyl)-tetraphosphatase (Symmetrical) ApaH OS=Citrobacter amalonaticus OX=35703 GN=apaH SS=EMBLWGS:MBE0397167 PC=UP000642697:Unassembled WGS sequenceMATYLIGDVHGCYDELIALLQQVNFTPESDTLWLTGDLVARGPGSLDVLRYVKSLGDCVRLVLGNHDLHLLAVFAGISRNKPKDRLSPLLEAPDADELLNWLRRQPLLQIDEEKKLVMAHAGITPQWDLQTAKDCARDVEAVLSSDSYPFFLDAMYGDMPNNWTPELTGLARLRFITNAFTRMRYCFPNGQLDMYSKESPEDAPAPLKPWFAIPGPVSEAYSIAFGHWASLEGKGTPEGIYALDTGCCWGGNLTCLRWEDKQYFVQPSNRQMDLNEGEAVNA>UPI00049EE2D3 Sulfolactaldehyde 3-reductase OS=Citrobacter amalonaticus OX=35703 GN=yihU SS=EMBLWGS:MBE0398218 PC=UP000642697:Unassembled WGS sequenceMAVIAFIGLGQMGAPMASNLLKQGHQLSVFDVNPDAVQRLVEKGAQPARSPAQAAEGAEFVITMLPNGDLVRSVLLGEKGVCERLSPQALVIDMSTIHPLQTDKLIADLRVKGFAMMDVPVGRTSDHAVAGTLLLLAGGTPDQVERATPVLMAMGNELINAGGPGMGIRVKLINNYMSIALNALSAEAAVLCEALGLSFDVALQVMSGTPAGKGHFTTSWPNKVLKGDLSPAFMIDLAHKDLGIALDVANQLHVPMPLGAASREVYNQARAAGRGREDWTAILEQVRTSAGLKNHH>UPI00049EE33F GDP-L-fucose synthase OS=Citrobacter amalonaticus OX=35703 GN=ILP74_07005 SS=EMBLWGS:MBE0395223 PC=UP000642697:Unassembled WGS sequenceMSKQRIFIAGHRGMVGSAIARQLAQRGDVELVLRTRDELNLLDSKAVLDFFATERIDQVYLAAAKVGGIVANNTYPADFIFENMMMESNIIHAAHLHNVNKLLFLGSSCIYPKLAKQPMAESELLQGTLEPTNEPYAIAKIAGIKLCESYNRQYGRDYRSVMPTNLYGPHDNFHPSNSHVIPALLRRFHEATMQNAADVVVWGSGTPMREFLHVDDMAAASIHVMELDSEVWQENTQPMLSHINVGTGVDCTIRELAQTIAQVVGYKGRVVFDATKPDGTPRKLLDVTRLHQLGWYHEISLEAGLASTYQWFLENQHRFRG>UPI00049EE40C 6-phosphogluconate phosphatase OS=Citrobacter amalonaticus OX=35703 GN=yieH SS=EMBLWGS:MBE0398084 PC=UP000642697:Unassembled WGS sequenceMSGIEAVFFDCDGTLVDSEVICSRAYVAMFQEFGITLDLEETFKRFKGVKLYEIIDIINEEHGVTLAKADLEPVYRAEVARLFDSELEVIPGANTLLDSMTVPMCVVSNGPVSKMQHSLGKLGMLHHFPDKLYSGYDIQRWKPDPALMFHAAKAMNVNAQNCILVDDSSAGAQSGIDAGMEVFYFCADPHNKPIEHPKVTTFTDLAQLPALWKARGWNITR>UPI00049EE431 LysR family transcriptional regulator OS=Citrobacter amalonaticus OX=35703 GN=ILP74_11260 SS=EMBLWGS:MBE0396016 PC=UP000642697:Unassembled WGS sequenceMWSEYALEVVDAVARNGSFSSAAQELHRVPSAVSYTVRQLEEWLAVPLFERRHRDVVLTPAGAWFLKEGRSVIKKMQITRQQCQQIANGWRGQLPIAVDNIVRPERTRQLIVDFYRHFDDVELLVFQEVFNGVWDALSDGRVELAIGATQAIPVGGRYTFRDMGKLSWCCVVASTHPLAAMSGPLSDDTLRNWPSLVLEDTSRTLPKRVTWLLDNQKRVVVPDWDSSATCISAGLCVGMVPTHFAKPYLNKGQWVALNLENPFPDAACCLTWQQNDMSPALIWLLDYLGDSETLNKEWLREPDETPAEEA>UPI00049EE5A3 DUF485 domain-containing protein OS=Citrobacter amalonaticus OX=35703 GN=ILP74_00150 SS=EMBLWGS:MBE0393942 PC=UP000642697:Unassembled WGS sequenceMNDTIYQRIEDSARFRELVEKRQRFATILSIIMLVVYISFILLIAFAPGWLGTPLHEGTSVTRGIPIGVGVILISFVLTGIYIWRANGEFDRLNKAVLHEVNAL>UPI00049EE61F N-acetylmuramoyl-L-alanine amidase AmiC OS=Citrobacter amalonaticus OX=35703 GN=amiC SS=EMBLWGS:MBE0394407 PC=UP000642697:Unassembled WGS sequenceMSGSNPPISRRRLLQGAGAMWLLSVSQVGLAAVSQVIAVRIWPASSYTRVTVESNQQLKYKQFALSNPERVVVDIEGVNLNSVLKGMAAQIRPDDPYIKSARVGQFDPQTVRMVFELKQNVKPQLFALAPVAGFKERLVMDLYPANAQDMQDPLLALLEDYNKGDLDKQVPPAQSGPQPGKAGRDRPIVIMLDPGHGGEDSGAVGKYKTREKDVVLQIARRLRALIEKEGNMKVYMTRNEDIFIPLKVRVAKAQKQRADLFVSIHADAFTSRQPSGSSVFALSTKGATSTAAKYLAQTQNASDLIGGVSKSGDRYVDHTMFDMVQSLTIADSLKFGKAVLNKLGRINNLHKNQVEQAGFAVLKAPDIPSILVETAFISNIEEERKLKTATFQQEVAESILAGIKAYFADGATLARRG>UPI00049EE6CA Sulfonate ABC transporter substrate-binding protein OS=Citrobacter amalonaticus OX=35703 GN=ILP74_13110 SS=EMBLWGS:MBE0396361 PC=UP000642697:Unassembled WGS sequenceMRNLLKARTAWLAFAGLLAFCGLTQAAEPSPDALRIGYQKGSISMVLAKSHQLLETRYPGTKISWIEFPAGPQMLEALNVGSIDLGSTGDIPPIFAQAAGADLVYVGVEPAKPKAEVILVPENSPIKTVAELKGHKVAFQKGSSSHNLLLRALQQAGLKFTDIQPTYLTPADARAAFQQGNVDAWAIWDPYYSAALLQGGVRVLKDGSDLKQTGSFYLAARPYAEKNGAFIQGVLDTFSQADALTLSQRQESIALLAKTMGLPEPVIASYLDHRPPTRISPVSDAVAALQQQTADLFYDNRLVPKKIDIRQRIWHPTQQEGKSL>UPI00049EE6FA 3,4-dihydroxy-2-butanone-4-phosphate synthase OS=Citrobacter amalonaticus OX=35703 GN=ribB SS=EMBLWGS:MBE0397418 PC=UP000642697:Unassembled WGS sequenceMNQTLLSSFGTPFERVEHALSALREGRGVMVLDDEDRENEGDMIFPAETMTVEQMALTIRHGSGIVCLCITEDRRKQLDLPMMVENNTSAYGTGFTVTIEAAVGVTTGVSAADRVTTVRAAIADGAKPSDLNRPGHVFPLRAQPGGVLTRGGHTEATIDLVTLAGFKPAGVLCELTNDDGSMARAPECIDFAGKHNMAVVTIEDLVAYRQAHERKAS>UPI00049EE761 DUF1471 domain-containing protein OS=Citrobacter amalonaticus OX=35703 GN=ILP74_16320 SS=EMBLWGS:MBE0396963 PC=UP000642697:Unassembled WGS sequenceMKTGYKLLIGALAFVATNAFAAELLTKAEFEKVESQYTLIGNINTSNETSTQDAKEDLLKKADEKGADVVVLTSGQTDNKIHGTANIYKKK>UPI00049EE7FE Dimethylsulfoxide reductase subunit B OS=Citrobacter amalonaticus OX=35703 GN=dmsB SS=EMBLWGS:MBE0393985 PC=UP000642697:Unassembled WGS sequenceMKQYGFYVDSSRCSGCKTCQVSCKDNKDLDVGPKFRRVYEYGGGSWVKEGESWHNDTFTYYLSIACNHCDEPVCVSGCPTGAMHKREEDGLVLVDDSICVGCRYCEMRCPYGAPQFDAKAKVMRKCDGCLDRLEQNLRPICVDSCPQRALDFGPIDELRAKYGSENEIAPLPAASFTHPNLIIKPHPKAKPTGDKEGAIMNMGEVRHA>UPI00049EE818 Histidine ABC transporter permease HisM OS=Citrobacter amalonaticus OX=35703 GN=hisM SS=EMBLWGS:MBE0394912 PC=UP000642697:Unassembled WGS sequenceMIEIIQEYWKSLLWTDGYRFTGVAITLWLLISSVVMGGILAVFLAIGRVSSNKFIQFPIWLFTYIFRGTPLYVQLLVFYSGMYTLEIVKGTDFLNAFFRSGLNCTVLALTLNTCAYTTEIFAGAIRSVPHGEIEAARAYGFSSFKMYRCIILPSALRIALPAYSNEVILMLHSTALAFTATVPDLLKIARDINSATYQPFTAFGIAAVLYLIISYVLISLFRRAERRWLQHVSSK>UPI00049EE84D FAD:protein FMN transferase ApbE OS=Citrobacter amalonaticus OX=35703 GN=apbE SS=EMBLWGS:MBE0394998 PC=UP000642697:Unassembled WGS sequenceMEMNVARAALLAVTLFFTGCDNAPDAAQTPAAAPVVLEGKTMGTFWRVSVVGVDAKSAAALQTKIQTQLDADDQLLSTYKNDSALMRFNQSKSLSPWPVNEAMADIVTSALRIGQKTQGAMDITVGPLVNLWGFGPDKQPVQIPTQAQIDAAKANSGLQHLSVINQAKQQYLQKDLPDLFVDLSTVGEGYAADHLAQLMEREGIARYLVSVGGALNSRGMNAEGHPWRVAIQKPTDRENAVQAVVDINGHGISTSGSYRNYYELNGKRLTHVIDPQTGRPIEHNLVSVTVIAPTALEADGWDTGLMVLGTEKAKEVVRREGLAVYMIIKEGEGFKTWMSPQFESFLIREQN>UPI00049EE905 Glycoside-pentoside-hexuronide family transporter OS=Citrobacter amalonaticus OX=35703 GN=ILP74_21810 SS=EMBLWGS:MBE0397991 PC=UP000642697:Unassembled WGS sequenceMTIHILSVKEKIGYGMGDAASHIIFDNVMLYMMFFYTDIFGIPAGFVGTMFLLARALDAISDPCMGLLADRTRSRWGKFRPWVLFGALPFGLVCVLAYSTPDLSLNGKMIYAAITYTLLTLLYTVVNIPYCALGGVITNDPTQRISLQSWRFVLATAGGMLSTVLMMPLVNLIGGDNKALGFQGGIAVLSVVAFLMLAFCFFTTKERIEAPPSTTSMREDLRDIWQNDQWRIVGLLTILNILAVCVRGGAMMYYVTWILGTPEVFVAFLTTYCVGNLIGSALAKPLTDWKCKVSIFWWTNAALAVVSLAMFFVPMQADITMFVFIFVIGVLHQLVTPIQWVMMSDTVDYGEWCNGKRLTGISFAGTLFVLKLGLALGGALIGWMLAGGGYDAAAKTQNNATINIIIALFTIVPAICYLLSAVIAKRFYTLKTPYLRNILDQLAQGARRNQQEFTHNEFQN>UPI00049EE91D Maleylacetoacetate isomerase OS=Citrobacter amalonaticus OX=35703 GN=maiA SS=EMBLWGS:MBE0395126 PC=UP000642697:Unassembled WGS sequenceMKLYSFFNSSASYRVRIALALKGIDYHTVGVNIRIGQQNELAYRRMNPVGLVPTLVTDDGEALGQSLAIIDWLDRHFPQSRLLPVSDPARTQVLEIVYAIACDIHPVNNLRVLRYLSEELKVSEEEKKRWYAHWIQQGLSAVEQLLRQSQSQNFCVGNAPTLADCCLVPQWANALRMGCDLSGYPRCKAVYDACTQLPAFIAAAPENQQDKISA>UPI00049EE921 Urea ABC transporter ATP-binding subunit UrtE OS=Citrobacter amalonaticus OX=35703 GN=urtE SS=EMBLWGS:MBE0395931 PC=UP000642697:Unassembled WGS sequenceMLQVNELNQYYGGSHILRGVTFDASPGEVTCLLGRNGVGKTTLLKCLMGLIPARSGTVTWQEKNITHRKPHQRVQAGIAYVPQGREIFPRLTVEENLLLGLSRFSSREAKAVPEEIYTLFPVLKEMKQRRGGDLSGGQQQQLAIGRALASRPQLLILDEPTEGIQPSVIKEIGQVIAQLARRGDMAILLVEQFYDFAAELADNYLLMSRGSIIQRGRGENMESEGVRGLVAI>UPI00049EE9E4 L-idonate 5-dehydrogenase OS=Citrobacter amalonaticus OX=35703 GN=ILP74_01025 SS=EMBLWGS:MBE0394104 PC=UP000642697:Unassembled WGS sequenceMEVKTQSCVVAGKRSVAVTQQNIEWNNKGTLVKITRGGICGSDLHYYQEGKVGNFTVKAPMILGHEVIGKVVHSDSETLREGQSIAINPSKPCGHCKYCLQHEENQCTEMRFFGSAMYFPHVDGGFTQFKTVDTAQCIPYPEQADEKVMAFAEPLAVAIHAAHEAGDLQGKKVFISGVGPIGCLIVSAVKTLGAAEVVCADVSPRSLSLAQQMGADTLVNPQHDALDQWKTEKGYFDISFEVSGHPSSITTCLEVTRAKGVMVQVGMGGAVPDFPMMMLIGKEIALKGSFRFTTEFNTAVSWLAHNVIDPLPLLSAEYPFTDLEQALIFASDKTQAAKVQLVFENEK>UPI00049EEAC2 Methionine ABC transporter ATP-binding protein MetN OS=Citrobacter amalonaticus OX=35703 GN=metN SS=EMBLWGS:MBE0397027 PC=UP000642697:Unassembled WGS sequenceMIKLSNITKVFHQGTRTIQALNNVSLHVPAGQIYGVIGASGAGKSTLIRCVNLLERPTEGSVQVGGQELTTLSESELTKARRQIGMIFQHFNLLASRTVFGNVALPLELDNTPKEEIKRRVTELLDLVGLGDKHDSYPANLSGGQKQRVAIARALASQPKVLLCDEATSALDPATTRSILELLKDINRRLGLTILLITHEMDVVKRICDCVAVISNGELIEQDTVSEVFSHPKTPLAQKFIQSTLHLDIPEDYLARLKAESTTDSVPMLRMEFTGQSVDAPLLSETARRFNVNNNIISAQMDYAGGVKFGIMLTEMHGTQEETQAAIAWLQEHHVKVEVLGYV>UPI00049EEB2A Adenine phosphoribosyltransferase OS=Citrobacter amalonaticus OX=35703 GN=apt SS=EMBLWGS:MBE0396828 PC=UP000642697:Unassembled WGS sequenceMTATAQQLEFLKNSIKSIQDYPKPGILFRDVTSLLEDPKAYALSIELLVERYKNAGITKVVGTEARGFLFGAPVALGLGVGFVPVRKPRKLPRETIAESYELEYGTDQLEIHLDAIHAGDKVLVVDDLLATGGTIEATVKLIRRLGGEVTDAAFIINLFDLGGEQRLAQQGINCYSLVPFPGH>UPI00049EEB73 Phenylalanine transporter OS=Citrobacter amalonaticus OX=35703 GN=pheP SS=EMBLWGS:MBE0396768 PC=UP000642697:Unassembled WGS sequenceMKDASSASDNGRFEASSEQSPTLQRGLQNRHIQLIALGGAIGTGLFLGIGPAIQMAGPAVLLGYGIAGVIAFLIMRQLGEMVVEEPVSGSFAHFAYKYWGPFAGFLSGWNYWVMFVLVGMAELTAAGIYMQYWLPEVPTWVWAASFFIIINAVNLVNVRLYGETEFWFALIKVLAIIGMIGFGLWLLFSGNGGERATIDNLWQHGGFLATGWKGLILSLAVIMFSFGGLELIGITAAEARDPHKSIPKAVNQVVYRILLFYIGSLVVLLALYPWVEVKSDSSPFVMIFHDLNSNVVASALNFVILVASLSVYNSGVYSNSRMLFGLSVQGNAPKFLTRVSRRGVPVNSLLLSGAITSLVVLINYLLPKEAFGLLMALVVATLLLNWIMICLAHLRFRAAMRRKGRDTQFKALLYPAGNYICIAFLAMILVLMCTIDDMRLSAMLLPVWVIFLFVAFKLSRKVR>UPI00049EEBA6 YibL family ribosome-associated protein OS=Citrobacter amalonaticus OX=35703 GN=ILP74_21525 SS=EMBLWGS:MBE0397934 PC=UP000642697:Unassembled WGS sequenceMKEVEKNEIKRLSDRLDAIRHQQADLSLVEAADKYAELEKEKATLEAEIARLRDVHSQKLSKEAQKLMNLPYRRAISKKEQADMGKLKKSVRGLVVVHPMTALGREMGLQEMTGFAKTEF>UPI00049EEBC0 Type 3 dihydrofolate reductase OS=Citrobacter amalonaticus OX=35703 GN=folA SS=EMBLWGS:MBE0397168 PC=UP000642697:Unassembled WGS sequenceMISLIAALAVDRVIGMENAMPWNLPADLAWFKRNTLNKPVVMGRHTWESIGRPLPGRKNIVISSQPGTDDRVQWVKSVDEAITACGDAPEIMVIGGGRVYEQFLPKAQKLYLTHIDAEVEGDTHFPDYEPDDWESVFSEFHDADGQNSHSYCFEILERR>UPI00049EEBC7 Thiamine/thiamine pyrophosphate ABC transporter permease ThiP OS=Citrobacter amalonaticus OX=35703 GN=thiP SS=EMBLWGS:MBE0397151 PC=UP000642697:Unassembled WGS sequenceMATRRQPLIAGWLIPGLCAATLMVTVALAAFLALWFNAPQAAWSSLWQDSYLWHVVRFSFWQAFLSAVLSVVPAIFLARALYRRRFPGRLALLRLCAMTLILPVLVAVFGILSVYGRQGWLASLWHVLGLEWTFSPYGLQGILLAHIFFNLPMASRLLLQSLENIPGEQRQLAAQLGIRGWQFFRFVEWPWLRRQIPPVAALIFMLCFASFATVLSLGGGPQATTIELAIYQALNFDYDPARAAMLALIQMVCCLALVLLSQRLSKAIAPGTTLVQGWRDPDDRLHSRLTDALLIVLALLLLLPPLLAVMVDGVNRHLLDVLAQPILWQAVWTSLRIALAAGLLCVILTMMLLWSSRELRARQQLLAGQALELSGMLILAMPGIVLATGFFLLLNNSIGLPESADGIVIFTNALMAIPYALKVLENPMRDITARYTMLCQSLGIEGWQRLKVVELRALKRPLAQALAFACVLSIGDFGVVALFGNDDFRTLPFYLYQQIGSYRSQDGAVTALLLLILCFILFTVIETLPGRNVKTD>UPI00049EEBEE SDR family oxidoreductase OS=Citrobacter amalonaticus OX=35703 GN=ILP74_01690 SS=EMBLWGS:MBE0394230 PC=UP000642697:Unassembled WGS sequenceMGIALVTGGSRGIGRATALQLADEGYTVAVNFHHNIRAATEVVNKIVAAGGNAFTLRADISDEAQVVAMFESIDRENEPLVALVNNAGILFEQSTIENLSAERINRVLATNVTGYFLCCREAVKRMSHKHGGHGGAIVNVSSAASRLGAPGEYVDYAASKGAVDSLTTGLALEVAAQGIRVNGVRPGLIYTEIHASGGEPGRVDRVKSMLPMQRGGQPEEVAQAIAWLLSEKASYVTGSFLELAGGK>UPI00049EEC41 Methylenetetrahydrofolate reductase OS=Citrobacter amalonaticus OX=35703 GN=metF SS=EMBLWGS:MBE0398344 PC=UP000642697:Unassembled WGS sequenceMSFFHANQREALNQSLAEVQGQINVSFEFFPPRTSEMEQTLWNSIDRLSSLKPKFVSVTYGANSGERDRTHSIIKGIKDRTGLEAAPHLTCIDATRDELRTIAQDYWNNGIRHIVALRGDLPAGSGKPDMYAADLVSLLKEVADFDISVAAYPEVHPEAKSAQADLLNLKRKVDAGANRAITQFFFDVESYLRFRDRCVSAGIDVEIIPGILPVSNFKQAKKFADMTNVRIPAWMSQMFNGLDDDAETRKLVGANIAMDMVKILSREGVKDFHFYTLNRAEMSYAICHTLGVRPGI>UPI00049EECA6 Peptide ABC transporter ATP-binding protein SapF OS=Citrobacter amalonaticus OX=35703 GN=sapF SS=EMBLWGS:MBE0395736 PC=UP000642697:Unassembled WGS sequenceMVETLLEVRNLSKTFRYRTGWFRRQTVEAVKPLSFTLRERQTLAIIGENGSGKSTLAKMLAGMVEPTSGELLIDDHPLEFGDYSFRSQRIRMIFQDPSTSLNPRQRISQILDFPLRLNTDLEPEQRRKQIIETMRMVGLLPDHVSYYPHMLAPGQKQRLGLARAMILRPKVIIADEALASLDMSMRSQLINLMLELQEKQGISYIYVTQHLGMMKHISDQVLVMHQGEVVERGSTADVLASPLHELTKRLIAGHFGEALTADAWRKDR>UPI00049EECC5 Neutral zinc metallopeptidase OS=Citrobacter amalonaticus OX=35703 GN=ILP74_04200 SS=EMBLWGS:MBE0394694 PC=UP000642697:Unassembled WGS sequenceMRWQGRRESDNVEDRRSSSGGGPSLGGPGFRLPSGKGGIILLIVVLVAGYYGVDLTGLMTGQPVSQQQSQRSISPNEDEAAKFTSVILATTEDSWGQQFEKMGRTYQPPKLVMYRGATRTGCGTGQSIMGPFYCPADSTVYIDLSFYDDMKDKLGADGDFAQGYVIAHEVGHHVQKLLGIEPKVRQMQQNASQAEINQLSVRMELQADCFAGVWGNSMQQQGVLESGDLEEALNAAEAIGDDRLQQQSQGRVVPDSFTHGTSEQRYRWFKRGFDSGNPAQCNTFGKGF>UPI00049EED18 SH3 domain-containing protein OS=Citrobacter amalonaticus OX=35703 GN=ILP74_18855 SS=EMBLWGS:MBE0397424 PC=UP000642697:Unassembled WGS sequenceMPKLRLIGFMLLALSATAVSHAEEKRYVSDELNTWVRSGPGDNYRLVGTVNAGEEVTLLQTDANTNYAQVKDSTGRTAWIPMKELNSTPSLRTRVPDLENQVKTLTDKLNNIDTTWNQRTADMQQKVSQSDSVINGLKEENQKLKNELIVAQKKVSAANLQLDDKQRTIIMQWFMYGGGVLGLGLLLGLILPHMIPSRKRKDRWMN>UPI00049EEDA4 Multidrug efflux pump-associated protein, AcrZ family OS=Citrobacter amalonaticus OX=35703 GN=ILP74_14215 SS=EMBLWGS:MBE0396572 PC=UP000642697:Unassembled WGS sequenceMLELLKSLVFAVIMVPVVMAIILGLIYGLGEVFNIFSGIGQKNQSRQNH>UPI00049EEE05 PTS sugar transporter subunit IIC OS=Citrobacter amalonaticus OX=35703 GN=ILP74_17610 SS=EMBLWGS:MBE0397191 PC=UP000642697:Unassembled WGS sequenceMIIEAMLIGILCYLGALSSPWLLGLTGGWYLITRPLISGTLVGIILGDMKTGIMIGVAVQAVYIAMVTPGGSMPADLNFVAYPAIALGILSGKGPEVAVALAATIGIAGTILFNAMMVLNSFWNHRADAALDRGDERGIYLNSAIWPQVTNFVLRFVPTFIAVYFGAQYISGFMDSLPKMVLSTMNVLGGILPAVGIAILLKQIIKNYTMLIYFLVGFVCIVFLKLNMVALVIVGSLLALIHYNYKPEPPQAANATSVTRDDEDEF>UPI00049EEE59 Arginine-ornithine antiporter OS=Citrobacter amalonaticus OX=35703 GN=arcD SS=EMBLWGS:MBE0394127 PC=UP000642697:Unassembled WGS sequenceMTTLDTSKPAADASTSQSEGKLKLPALTALVVSALIAAGVFSLPQNMAAKAGAGAILIGWGITFIGMLTLAFVFQTLAHRKSEVEGGVYGYARAGFGEYIGFNSAWGYWISAWIGNVSYYVVICSALGSFSALGFFGDGTTLSALIVGSILLWSLHFLICRGVQGAALLNLIGTVAKVVPLIMFVVLVSVAFQVRTFKIEFWGNEQLGSVMDQVKNIMLVTTWVFIGIEGAAMYSGRAMKKSDVGKATMIGFFISILLFVAVSVLSLGVLSQPELAQLKNPSTAGVLAAAVGPWGAALMNIGLIVSVGAALLAWTLLSAETAYMAGKDGTMPKFLGKENANKAPVNALLLTNGLTQLFLIIAHFQQAGYLALLLLATSMILIPYFLSGLYALKVAWQKDGYNRDEQHSITRDIIIGALATLYGAWLVYAAGMEYLLLSMILYALGIVFYVWARKEKNGRLFNPIEIVLAIMVVVAGIYAVYLLATGVLTLS>UPI00049EEF19 L-alanine exporter AlaE OS=Citrobacter amalonaticus OX=35703 GN=alaE SS=EMBLWGS:MBE0394538 PC=UP000642697:Unassembled WGS sequenceMFSPQSRLRHAVADTFAMVVYCSVVNMLIEIFLSGMTFEQSLSSRLVAIPVNILIAWPYGMYRDLFMKASRKVGSAGWLKNLADVLAYVTFQSPVYVAILLTVGADWHQITAAVSSNIVISMMMGAVYGYFLDYCRRLFKVSRYQQVKA>UPI00049EEF4B Heat shock protein HspQ OS=Citrobacter amalonaticus OX=35703 GN=hspQ SS=EMBLWGS:MBE0396339 PC=UP000642697:Unassembled WGS sequenceMIASKFGIGQQVRHSLLGYLGVVVDIDPVYSLAEPSPDELAVNDELRAAPWYHVVMEDDDGRPVHTYLAEAQLSSETQDEHPEQPSMDELAQTIRKQLQAPRLRN>UPI00049EF066 L,D-transpeptidase family protein OS=Citrobacter amalonaticus OX=35703 GN=ILP74_11425 SS=EMBLWGS:MBE0396046 PC=UP000642697:Unassembled WGS sequenceMKRASLITLMLIGAYSAIQAAWAVDYPLPPAGSRLVGQNQTYTVQEGDKNLQAIARRFDTAAMLILEANNTIAPVPKPGTLVTIPSQLLLPDAPREGIIVNLAELRLYYYPPGENIVQVYPIGIGLQGLETPVMETRVGQKIPNPTWTPTAGIRKRSLERGITLPPVVPAGPNNPLGLFALRLAHGNGEYLIHGTSQPDSVGLRVSSGCIRMNAPDIKALFSQVRTGTPVRVINEPVKYSVEPSGLRYVEVHRPLSPEEEQNVQTMPYVLPAGFSQFKATKEVDDALVDKALYRRAGYPVAVSAGQTPTVEATPAVQSAQNGSVQEETQTQ>UPI00049EF1E3 Heme exporter protein CcmB OS=Citrobacter amalonaticus OX=35703 GN=ccmB SS=EMBLWGS:MBE0395013 PC=UP000642697:Unassembled WGS sequenceMMWRIFRLELRVAFRHSAEIANPLWFFLIVITLFPLSIGPEPQLLARIAPGIIWVAALLASLLALERLFRDDLQDGSLEQLMLLPIPLPMVVLAKVMAHWMVTGLPLLILSPLVALLLGMDLYGWKIMALTLLLGTPTLSFLGAPGVGLTVGLKRGGVLLSVLVLPLTIPLLIFATAAMDAASMHLPVDGYMAILGALLAGSATLSPFATAAALRISLQ>UPI00049EF2C2 Glucose-1-phosphate adenylyltransferase OS=Citrobacter amalonaticus OX=35703 GN=glgC SS=EMBLWGS:MBE0397779 PC=UP000642697:Unassembled WGS sequenceMVSLEKNDRLMLARQLPLKSVALILAGGRGTRLKDLTNKRAKPAVHFGGKFRIIDFALSNCLNSGIRRIGVITQYQSHTLVQHIQRGWSFFSEEMNEFVDLLPAQQRMQGENWYRGTADAVTQNLDIIRRYKAEYVVILAGDHIYKQDYSRMLIDHVEKGARCTVACMPVPIEEASAFGVMAVDENDKIIEFVEKPANPPAMPGDATKSLASMGIYIFDADYLYELLEKDDSDEGSSHDFGKDIIPTVTKAGMAYAHPFPLSCVQSDPESEPYWRDVGTLEAYWKANLDLASVTPELDMYDQNWPIRTHMESLPPAKFVQDRSGSHGMTLNSLVSGGCIISGSVVVQSVLFPRVRVNSFCNIDSAVLLPEVWVGRSCRLRRCIIDRACVIPEGMVIGENAEEDARRFYRSEEGIVLVTREMLRKLQVKQE>UPI00049EF2DC YciN family protein OS=Citrobacter amalonaticus OX=35703 GN=ILP74_09685 SS=EMBLWGS:MBE0395718 PC=UP000642697:Unassembled WGS sequenceMRKETQPIDRETLLIEANKIIREHEDTLAGIVATGVTQRNGVLVFSGDYFLDEQGLPTPKSTAVFNMFKHLAHVLSEKYHLID>UPI00049EF300 Two-component system sensor histidine kinase AtoS OS=Citrobacter amalonaticus OX=35703 GN=atoS SS=EMBLWGS:MBE0394993 PC=UP000642697:Unassembled WGS sequenceMRFLRGLYPRRLRNQMILMALLMVIVPTLSIGYIVETEGRSAVLSEKEKKLSAVVHLLDQALGDRFTHFTALPRDERIQALNTELGPITERITQAFPGVGAGYYNKALDAIVTYAPSALYQNNVGVTIAADHPGREVMRSNAPVVFSGRQVRGDILNSMIPITRDGDVLGYIWANELTEDIQRQAWRMDVRIMAVLAAGLLCSLLLIVLFSRRLGANIDIITDGLSTLAQKTPAQLPNLPGELGQISRSVNALAQTLRETKTLNDLIIENAADGVIAIDRQGDVTTMNPAAEMITGYTLNELVGRPYATLFSDPHFASPVLDTLAHGTEHLAQEVSFPARDRTIELSVTTSRIHNPNGELIGALVIFSDLTARKETQRRLAQTERLATLGELMAGVAHEVRNPLTAIRGYVQIIRQQTSLPVHQEYLSVVLKEIDSINKVIQQLLDFSRPRQSQWQQVLLNSLIEETLILVQTSGVQARITFNFEQDTGLPAIVADRELLKQVILNLLINAVQAINARGEIRIRTWQYSATQQAVAIEDNGGGIDIALQKKIFDPFFTTKASGTGLGLALSQRIINAHQGDIHVASMPGCGATFTLILPINPQGNLSV>UPI00049EF30A Colanic acid biosynthesis pyruvyl transferase WcaK OS=Citrobacter amalonaticus OX=35703 GN=wcaK SS=EMBLWGS:MBE0395229 PC=UP000642697:Unassembled WGS sequenceMKLLILGNHTCGNRGDSAILRGLLDAIHRLEPDAEVDVMSRYPVSSSWLLNRPVMGDPLFLQMKQHNSAAGVVGRVKKILRRRYQHQVLLSRVTDTGKLRNIAIAQGFTDFVRLLAGYDAIVQVGGSFFVDLYGVPQFEHALCTFMAKKPLYMVGHSVGPFQDPQFNQLANYVFGHCDALILRESVSLELMKRSDITTEKVEHGVDTAWLVDHHDEDFTPGYAVQHWLSVAAQQKTVAITLRELAPFDKRLGTTQEAYEKAFAGVVNRILDEGYQVIALSTCTGIDSYNKDDRMVALNLRQYVSDPARYHVVMDELNDLEMGKILGACDLTVGTRLHSAIISMNFATPAIAINYEHKSAGIMQQLGMPEMAIDIRHLLDGSLQAMVADTLGQLPQINERLAQAVRREREQGYRMVESVLTRIGEGK>UPI00049EF369 Arginine N-succinyltransferase OS=Citrobacter amalonaticus OX=35703 GN=astA SS=EMBLWGS:MBE0396113 PC=UP000642697:Unassembled WGS sequenceMMVIRPIEHADISALMQLASKTGGGLTSLPANEATLAARIDRSLKTWRGELPKSEQGYVFVLEDLETGTVAGICAIEVAVGLNDPWYNYRVGTLVHASKELNVYNALPTLFLSNDHTGSSELCTLFLDPDWRKEGNGYLLSKSRFMFMAAFRDRFNEKVVAEMRGVIDEHGYSPFWQSLGKRFFSMEFTRADFLCGTGQKAFIAELMPKHPIYTHFLSDEAQAVIGEVHPQTAPARAVLEKEGFRYRNYIDIFDGGPTLECDIDRVRAIRKSRLIEVVEGQPATGDFPACLVANENYHHFRVMLVRADPHTERLVLTAAQLDALKCHAGDRVRFVRLCAEEKTV>UPI00049EF37F TRNA (Adenosine(37)-N6)-threonylcarbamoyltransferase complex transferase subunit TsaD OS=Citrobacter amalonaticus OX=35703 GN=tsaD SS=EMBLWGS:MBE0397440 PC=UP000642697:Unassembled WGS sequenceMRVLGIETSCDETGIAIYDDEKGLLANQLYSQVKLHADYGGVVPELASRDHVRKTVPLIQAALKEAGLSAKEIDAVAYTAGPGLVGALLVGATVGRSLAFAWNVPAIPVHHMEGHLLAPMLEDNPPEFPFVALLVSGGHTQLISVTGVGQYELLGESIDDAAGEAFDKTAKLLGLDYPGGPMLSKMASQGTAGRFVFPRPMTDRPGLDFSFSGLKTFAANTIRNNGGDDQTRADIARAFEDAVVDTLMIKCKRALDQTGFKRLVMAGGVSANRTLRAKLAEMMQKRRGEVFYARPEFCTDNGAMIAYAGMVRFKAGATADLGVTVRPRWPLAELPAA>UPI00049EF3C7 4-carboxymuconolactone decarboxylase OS=Citrobacter amalonaticus OX=35703 GN=pcaC SS=EMBLWGS:MBE0395779 PC=UP000642697:Unassembled WGS sequenceMQDEERYQQGMAVRRAVLGDAHVDRTVENLTPLNEEFQHFITRYAWGDIWSRPGLDRHTRSMITIAMLIALNREAELKMHLNAAFNNGVTREELKELIMHSALYCGLPAANATLHLAQQVFDQRDADGK>UPI00049EF528 Energy-coupling factor ABC transporter substrate-binding protein OS=Citrobacter amalonaticus OX=35703 GN=ILP74_07420 SS=EMBLWGS:MBE0395303 PC=UP000642697:Unassembled WGS sequenceMKKTLILLAMVIALVVLPFFINHGGEYGGSDGEAESQIQVVAPHYEPWFQPLYEPASGEIESLLFTLQGSLGAAVIFYILGYTKGRQRRDDRA>UPI00049EF61B 5'-deoxynucleotidase OS=Citrobacter amalonaticus OX=35703 GN=yfbR SS=EMBLWGS:MBE0394929 PC=UP000642697:Unassembled WGS sequenceMKQSHFFAHLSRLKLINRWPLMRNVRTENVSEHSLQVAMVAHALAAIKNRKFAGQVNAERIALLAMYHDASEVLTGDLPTPVKYFNSQIAQEYKAIEKIAQQKLVDMVPDELRDIFAPLIDEHAYSEDEKSIVKQADALCAYLKCLEELSAGNNEFLLAKTRLEKTLESRRSPEMDYFMEVFVPSFHLSLDEISQDSPL>UPI00049EF621 Uncharacterized protein OS=Citrobacter amalonaticus OX=35703 GN=ILP74_18385 SS=EMBLWGS:MBE0397335 PC=UP000642697:Unassembled WGS sequenceMAPTLIESYTKDLREWPHTGATLFVRSGYLLASTSLFKVSEHEILAPEDDIIRYILIERVGARIGDVELSNLILDHEDDGLDEQSTIQELFRAGIIDQTQNVTAGRVGLRNYSFVEDGQRIDCFQVAGAYIQPKSHRKGIMSRTYLFLLNWYEHLVCDDMQTIAGAQIWAGPMVRAGEVRIYNEKGESFEDVLGEKGIGRHTGFLPWNKGRMFDTSPWDPNKLQTTVQKFIVLIISRDSCQRIGFMT>UPI00049EF64B LacI family DNA-binding transcriptional regulator OS=Citrobacter amalonaticus OX=35703 GN=ILP74_21315 SS=EMBLWGS:MBE0397893 PC=UP000642697:Unassembled WGS sequenceMSKSTRPTISDVAKAAKTGKTSISRYLNGEKHLLSDALLARIEKAIADLDYRPSLMARGLKRGRTRLIGLIIADITNPYSVHVLSGIEAACREKGFTPLVCNTNNEVNQELHYLDLLRSYQVEGIVVNAVGMREEGLNRLQQSALPMVLIDRKIPDFACDVVGLDNTQAATTATEHLIEQGFEALLFLSEPLGMVNTRRERLSAFRATLARYPGVVAENAEVPLSENTQLDNTLRQFHQQHRGMRKAVISANGALTLQVARSLKRIGLNWGSDIGLLGFDELEWAELAGVGITTLKQPTWQIGYAAVEQVVRRIEGGSDAIHEQVFSGELIVRGSTAR>UPI00049EF733 CDF family cation-efflux transporter FieF OS=Citrobacter amalonaticus OX=35703 GN=fieF SS=EMBLWGS:MBE0398312 PC=UP000642697:Unassembled WGS sequenceMNQSYGRLVSRAAIAATVMASLLLLIKIFAWWYTGSVSILAALVDSLVDIAASLTNLLVVRYSLQPADDEHTFGHGKAESLAALAQSMFISGSALFLFLTGIQHLITPTPMNEPGVGVVVTIIALICTVVLVSFQRWVVKRTQSQAVRADMLHYQSDVMMNGAILVALGLAWYGWHRADALFALGIGIYILYSALRMGYDAVQSLLDRALPDEERQEIFDIVTSWPGVSGAHDLRTRQSGPTRFIQIHLEMEDNLPLVQAHVVAEQVEQAILRRFPGSDVIIHQDPCSVVPREGKRFELS>UPI00049EF739 Carbon starvation induced protein CsiD OS=Citrobacter amalonaticus OX=35703 GN=csiD SS=EMBLWGS:MBE0394549 PC=UP000642697:Unassembled WGS sequenceMNALTAVKPNAEDPGQPGNGFTLKPSAQSPRLLELTFSARTTEHFLRQVAQWPVQALEYKSFLRFKVGKILDDLCDNQLQPLLLKTLLDRAKGALLINAEGIDNVAQADDMVKLATAVAHLVGRSNFDAMSGQYYARFVVKNVDNSDSYLRQPHRVMELHNDGTYVEEITDYVLMMKIDEQNMTGGNSLLLHLDDWEHLDEYFSHPLARRPMRFAAPPSKNVSHDVYHPVFDVDQQGRPVMRYIDQFVQPKDFEEGVWLSELSDALETSKSILSVPVSVGKFLLINNLFWLHGRDRFTSHPDLRRELMRQRGYFAYSTHHYQTHQ>UPI00049EF78D ABC transporter ATP-binding protein OS=Citrobacter amalonaticus OX=35703 GN=ILP74_10640 SS=EMBLWGS:MBE0395902 PC=UP000642697:Unassembled WGS sequenceMTQPVLEIDDLHLSFPGYKADVHALSHVSLRIQRGEIVGVVGESGSGKSVTAMLTMRLLPEGSYRIHQGRVSLLGEDVLNASEKQMRQWRGARVAMIFQEPMTALNPTRRIGQQMVEVIRHHQTVSRAQAREKAITLLEEMQIPDAAGVMSRFPFELSGGMRQRVMIALAFSCEPALIIADEPTTALDVTVQLQVLRLLKHKARASGTAVLFISHDMAVVSQLCDRLYVMYAGSVIESGQTDAVIHHPTHPYSIGLLKCAPEQGEARQPLPAIPGTVPDLTRLPRGCAFRDRCFAAGPLCETVPAMSPHGAGDQHSACWYPHLENPHV>UPI00049EF7A8 Sulfate adenylyltransferase subunit CysN OS=Citrobacter amalonaticus OX=35703 GN=cysN SS=EMBLWGS:MBE0394457 PC=UP000642697:Unassembled WGS sequenceMNTTLAQQIANEGGVEAWMVAQQHKSLLRFLTCGSVDDGKSTLIGRLLHDTRQIYEDQLSSLHNDSKRHGTQGEKLDLALLVDGLQAEREQGITIDVAYRYFSTEKRKFIIADTPGHEQYTRNMATGASTCDLAILLIDARKGVLDQTRRHSFISTLLGIKHLVVAINKMDLVDYSEETFARIREDYLTFAEQLPGNLDIRFVPLSALEGDNVASQSVSMPWYSGPTLLEVLETVEIQRVVDSQPMRFPVQYVNRPNLDFRGYSGTLASGRVKVGQRVKVLPSGVESSVARIVTFDGDLDEAFAGEAITLVLKDEIDISRGDLLLAAEESLPAVQSAAVDVVWMAEQPLAPGQSYEIKIAGKKTRARVDHVQYQVDINNLTQREVDTLPLNGIGLVNLTFDEPLVLDTYQQNPVTGGLIFIDRLSNVTVGAGMVREPIVQETATPSEFSAFELELNALVRRHFPHWGARDLLGGK>UPI00049EF862 Antibiotic biosynthesis monooxygenase OS=Citrobacter amalonaticus OX=35703 GN=ILP74_18760 SS=EMBLWGS:MBE0397405 PC=UP000642697:Unassembled WGS sequenceMLTVIAEIRTRPGQHHRQAVLDQFAKIVPTVLKEEGCHGYAPMVDHAANVSFQTMAPDSIVMIEQWESIAHLEAHLQTPHMKAYSEAVKGDVLEMNIRILESGI>UPI00049EF8F9 ABC transporter permease OS=Citrobacter amalonaticus OX=35703 GN=ILP74_23250 SS=EMBLWGS:MBE0398254 PC=UP000642697:Unassembled WGS sequenceMNKVMTSEAIERAPDTRAVWQRLLCWEGFLFAVTLAVFIVNALASPYFLNIWNLSDATFNFTEKAIIVLPMAMLIIAREIDLSVASTLALSSTAMGFCAAAGMDTPLLVCVGLGTGLLCGLLNGILVTRFNLSSIVITIGTMSLYRGITYILLGDQALNAYPESFTWFGQGYVWGALSFEFALFIVLAIVFTFLLHKTNFGRRTYAIGNNPVGAWYSGINVKRHNLILFALVGLMAGLAAVLLTSRLGSTRPTIAMGWELAVVTMAVLGGVNILGGSGSMTGVIIAAFLMGLVTFGLSLLNVPGIVMSIIIGAMLIVVISLPILTRRMMQRRRI>UPI00049EF971 Divisome-associated lipoprotein YraP OS=Citrobacter amalonaticus OX=35703 GN=yraP SS=EMBLWGS:MBE0397520 PC=UP000642697:Unassembled WGS sequenceMKALSPIAVLISALLLQGCVAAAVVGTAAVGTKAATDPRSVGTQVDDGTLELRVNSALSKDAQIKKETRINVTAYQGKVLLVGQSPNSELSSRAKQIAMGVDGTTEVYNEIRQGEPIGMGTASNDTWITTKVRSQLLTSDQVKSSNVKVTTENGEVFLLGLVTEREAKAAADIASRVSGVKRVTTAFTFIK>UPI00049EF9A7 YheV family putative metal-binding protein OS=Citrobacter amalonaticus OX=35703 GN=ILP74_20390 SS=EMBLWGS:MBE0397713 PC=UP000642697:Unassembled WGS sequenceMAIRKRFIAGAKCPSCQAQDSLAMWRENNIDIVECVKCGHQMREADKEVRDHVRKEEQVIGIFHPD>UPI00049EFA03 Sigma factor-binding protein Crl OS=Citrobacter amalonaticus OX=35703 GN=crl SS=EMBLWGS:MBE0396976 PC=UP000642697:Unassembled WGS sequenceMTLPSGHPKSRLIKKFTALGPYIREGKCEDNRFFFDCLAVCVNVKPAPELREFWGWWMELEAHEKRFTYSYKFGLFDKAGDWKTVAIKDKEVVERLEYTLREFHEKLRELLNTLHLSLEPADDFRDEPVKLTA>UPI00049EFA21 HTH-type transcriptional activator AllS OS=Citrobacter amalonaticus OX=35703 GN=allS SS=EMBLWGS:MBE0397235 PC=UP000642697:Unassembled WGS sequenceMLDQETIRTFIRVAETESFSRAASSLHKTPAAISYRIKTLEEQVGTQLFLRTTRSVSLTLAGQHLLEHCRQWLNWLDAMPDELQQINAGVERQVNIVINNLLYQPQTTADLLTWLHQQFPFTRFQISRQVYMGVWDSLLYDDYQLAIGVTGSESLSNNISLLPLGDISWQFVVAQNHPLASHPATVLSDDMLRRYPAINIEDTSRTLTRRVAWLLSGQKEIKVPDLSTKLACHLSGLGVGFLPERLCRPYVESGALVARSVVNPRQPSPLSIAWKNAGSGKVVSEIAAIFKQKHGLVGGFLRMVDRPAERQ>UPI00049EFA30 Maltose O-acetyltransferase OS=Citrobacter amalonaticus OX=35703 GN=maa SS=EMBLWGS:MBE0396838 PC=UP000642697:Unassembled WGS sequenceMSEEKRKMIAGERYHPADETLRQDRLQARRFVHRYNHTAPDEKAERLAILHALLGQCDRPYIEPSFRCDYGYNIFLGKDFYANFDCVMLDVCPIIIGDNCMLAPGVHIYTATHPLDATERNSGLEFGKPVTIGNNVWIGGRAVINPGVSIGDNVVVASGAVVTKSVPANVVVGGNPARIIKTL>UPI00049EFA89 Dipeptide/tripeptide permease DtpA OS=Citrobacter amalonaticus OX=35703 GN=dtpA SS=EMBLWGS:MBE0395990 PC=UP000642697:Unassembled WGS sequenceMSTANKKPTESVSLNAFKQPKAFYLIFSIELWERFGYYGLQGIMAVYLVKQLGMSEADSITLFSSFSALVYGLVAIGGWLGDKILGTKRVIMLGAVVLAIGYALVAWSGHDAGIVYMGMAAIAVGNGLFKANPSSLLSTCYAKDDPRLDGAFTMYYMSVNIGSFFSMLATPWLAAHYGWSTAFALSVVGMLITVVNFAFCQRWVKNYGSKPDFEPINFRNLLLTIVGVIALIAIATWLLHNQQIARMVLGVIALGIVIIFGKEAFSMQGAARRKMIVAFILMLEAIIFFVLYSQMPTSLNFFAIRNVEHSILGIAFEPEQYQALNPFWIIIGSPILAAIYNKMGDTLPMPTKFAIGMVLCSGAFLILPLGAKFASDAGIVSVNWLIICYGLQSIGELMISGLGLAMVAQLVPQRLMGFIMGSWFLTTAGANIIGGYVASMMAVPENVTDPLMSLEVYGRVFLQIGVATAVIAVLMLLTAPKLNRMTQDDEPREKASKTATA>UPI00049EFA91 Methionine ABC transporter permease MetI OS=Citrobacter amalonaticus OX=35703 GN=metI SS=EMBLWGS:MBE0397028 PC=UP000642697:Unassembled WGS sequenceMSEPMMWLLVRGVWETLAMTFVSGFFGFVIGLPVGVLLYVTRPGQIIENAKLYRTLSAVVNIFRSIPFIILLVWMIPFTRVIVGTSIGLQAAIVPLTVGAAPFIARMVENALLEIPTGLIEASRAMGATPLQIVRKVLLPEALPGLVNAATITLITLVGYSAMGGAVGAGGLGQIGYQYGYIGYNATVMNTVLVLLVVLVYLIQFAGDRIVRTVTHK>UPI00049EFA96 P-hydroxybenzoic acid efflux pump subunit AaeA OS=Citrobacter amalonaticus OX=35703 GN=aaeA SS=EMBLWGS:MBE0397602 PC=UP000642697:Unassembled WGS sequenceMKTLTRKISRTAITLVLVILAFIAIFRAWVYYTESPWTRDARFSADVVAIAPDVAGLITAVKVHDNQLVKKDQVLFTIDQPRYQKALEEAEADVAYYQVLAQEKRQEAGRRNRLGVQAMSREEIDQANNVLQTVLHQLAKAQATRDLARLDLERTVIRAPADGWVTNLNVYTGEFITRGSTAVALVKQNSFYVLAYMEETKLEGVRPGYRAEITPLGSNKVLKGRVDSIAAGVTNASSTRDDKGMATIDSNLEWVRLAQRVPVRIQLDDQQENLWPAGTTATVVITGKQDRDENNDSFFRKMAHRLREFG>UPI00049EFAB3 Type 1 fimbrial protein OS=Citrobacter amalonaticus OX=35703 GN=ILP74_00420 SS=EMBLWGS:MBE0393991 PC=UP000642697:Unassembled WGS sequenceMKMNKVAMAVAFSAALGSMSVLADTTNGVIEFQGELVNTACGLAPGSSPVTVDFGQIPVSALANGARAGNVHQNIELQHCDITVAQTAEVTYTPTSTNPTDATLAAFTSGTASGAGIGLRDSASQDVVWGTATTPVQLVNGTNTIPFVAYVKAESASATVAAGSFQSTVNFEIAYQ>UPI00049EFBAF Amino acid ABC transporter permease OS=Citrobacter amalonaticus OX=35703 GN=ILP74_04745 SS=EMBLWGS:MBE0394790 PC=UP000642697:Unassembled WGS sequenceMTGFRWEIIQEYAPLFMEGAWMTIKCTIICVCLGTLWGLTLGLGRMAKAEHGFWKYALRYFVQYPVRFYVSAFRGTPLFVQIMVVHFALVPLFINPRDGILVTSGLMSADFARELRSGYGAFLSCIVAITLNAGAYVSEIFRAGIQSIDPGQMEASRALGMPWWKTMRKVILPQAFRRILPPLGNNAIAIVKDSSLASAIGLADLAYAARTVSGAYATYWEPYLTISLVYWVLTFLLAQLVNRLEKRFGKSDPH>UPI00049EFBB5 Outer membrane assembly protein AsmA OS=Citrobacter amalonaticus OX=35703 GN=asmA SS=EMBLWGS:MBE0395211 PC=UP000642697:Unassembled WGS sequenceMRRFLTTLMILLVVLVAGFSALVLLVNPNDFRAYMVQQVAARSGYQLQLDGPLRWHVWPQLSILSGRMVLTAEGAAEPLVRADNMRLDVALWPLLSHQLSVKQVMLKGAVIQLTPQTEAVRRENAPVAPKDNTLPDVAEDRGWSFDVASLRVADSVLVFQHEDDEQVTVRDIRLQMEQDAQHRGTFEFSGRVNRDQRDLTLAFNGTVDASDYPHNFTAGIQQLSWQLQGADLPKQGIQGQGQLQAQWQEEKKTLTFSQLNVTANDSALTGNVQVTLKEQPTWLVNLQFARLNLDNLLAPPENASSANGAVAQGGSQPPLARPVISSRVDEPAYQGLKGFSADIALKADNVTWRGMNFTDVSSRMTNQSDVLTIAELQGKLDGGMISLPGTLDTRAQTPQVVFQPRLDNVEIGTILKAFNYPIALTGKMSLAGDFSGVDIDAQSFRHSWQGKAHVDMRDTRMEGMNFQQLIQQAVERSGGDVQSMQSVDNATRLARFVTDLTLDHGKLTLDNMAGESAMLSLTGQGTLDLVGQTCDTQFNVRVLDGWKGESKLIDFLKATPVPLRVYGNWQALNYNLQVDQLLRKHMQDEAKRRLNDWAERNKDSRNGKDVKKLLDKL>UPI00049EFBCC Phosphoenolpyruvate carboxykinase (ATP) OS=Citrobacter amalonaticus OX=35703 GN=pckA SS=EMBLWGS:MBE0397752 PC=UP000642697:Unassembled WGS sequenceMRVKRLTPQDLKAYGISDVQDIVYNPSYETLYQEELDPSLEGYERGVLTNLGAVAVDTGIFTGRSPKDKYIVRDDTTRDTLWWSDKGKGKNDNKPLSQETWQHLKGLVTKQLSGKRLFIVDAFCGANADTRLSVRFITEVAWQAHFVKNMFIRPSDDELEGFEPDFIVMNGAKCTNPQWKEQGLNSENFVAFNLTERIQLIGGTWYGGEMKKGMFSVMNYLLPLKGIASMHCSANVGEKGDVAVFFGLSGTGKTTLSTDPKRRLIGDDEHGWDDDGVFNFEGGCYAKTIKLSKEAEPEIYNAIRRDALLENVTVRDDGTIDFDDGSKTENTRVSYPIYHIENIVKPVSKAGHATKVIFLTADAFGVLPPVSRLTANQTQYHFLSGFTAKLAGTERGVTEPTPTFSACFGAAFLSLHPTQYAEVLVKRMQASGAQAYLVNTGWNGTGKRISIKDTRAIIDAILNGSLDDAETFNLPMFDLAIPTELPGVDTRILDPRNTYGSPEQWQEKATALAKLFIENFEKYTDTPAGEALVSAGPKL>UPI00049EFC27 Cystine ABC transporter substrate-binding protein OS=Citrobacter amalonaticus OX=35703 GN=tcyJ SS=EMBLWGS:MBE0395472 PC=UP000642697:Unassembled WGS sequenceMKLALLGRQALMGVMAVALVAGMSAKTFADEGLLKKVKDRGTLLVGLEGTYPPFSFQGDDGKLTGFEVEFAEALAKHLGVKATLKPTKWDGMLASLDSKRIDVVINQVTISDERKKKYDFSTPYTVSGIQALVKKGNEGAIKTAADLKDKKVGVGLGTNYEEWLRQNVQGVDIRTYDDDPTKYQDLRVGRIDAILVDRLAALDLVKKTKDTLAVTGEAFSRQESGVALRKGNEDLLKAVDAAIAEMQKDGSLKALSEKWFGADITK>UPI00049EFC2B Carboxy terminal-processing peptidase OS=Citrobacter amalonaticus OX=35703 GN=prc SS=EMBLWGS:MBE0395568 PC=UP000642697:Unassembled WGS sequenceMNTFFRLTALAGLLAIAGQTFAVDDITRADQIPVLKEETQHATVSERVTSRFTRSHYRQFDLDSAFSAKIFDRYLNLLDYSHNVLLAGDVEQFAKKKGQLGDELRSGKLDVFYDLYNLAQKRRFERFQYALKVLERPMDFTGTDTFNLDRSKAPWPKDEAELNALWDGKVKFDELSLKLTGKDDKEIRETLTRRYKSAIRRLTQTNSEDVFSLAMTAFAREIDPHTNYLSPRNTEQFNTEMSLSLEGIGAVLQMDDDYTVINSMVAGGPAAKSKAISVGDRIVGVGQTGKSMVDVIGWRLDDVVALIKGPKGSKVRLEVLPAGKGTKTRIVTLTRERIRLEDRAVKMSVKTVGKEKVGVLDIPGFYVGLTDDVKVQLQKLEKQNVSSIVIDLRTNGGGALTEAVSLSGLFIPSGPVVQVRDNNGKVREDSDTDGVVYYKGPLVVLVDRFSASASEIFAAAMQDYGRALIVGEPTFGKGTVQQYRSLNRIYDQMLRPEWPALGSVQYTIQKFYRVNGGSTQRKGVTPDIIMPTGNEETETGEKFEDNALPWDSIDAATYVKSDDLTPFGPELLKDHNARIAQDPEFQYIMKDIARFNAMKDKRNIVSLNYAQREKENNEDDALRLARINDRFKREGKPVLKKLDDLPKDYQEPDPYLDETVKIALDLATLEKAKPAVQPTATK>UPI00049EFC5C GNAT family N-acetyltransferase OS=Citrobacter amalonaticus OX=35703 GN=ILP74_02315 SS=EMBLWGS:MBE0394340 PC=UP000642697:Unassembled WGS sequenceMNSFVEFFLLDRDELPSGFATELYRLRNRTFRERLNWKVECTDGLEKDQFDNENTTYLLGMHEGEILCGARFIDATQPTMISEIFHEYFDNITLPTDIPCCEVSRLFLDKERRDCGNLRGMPASKALFLAMIVYCMKKQYRGMFAVASRGMYAIFRHANWKIDVIQKGLSEKGETIYYIFMPASESAIESIISKDKASHWLREILQQLRRL>UPI00049EFC87 Ribonuclease HI OS=Citrobacter amalonaticus OX=35703 GN=rnhA SS=EMBLWGS:MBE0396995 PC=UP000642697:Unassembled WGS sequenceMLKQVEIFTDGSCLGNPGPGGYGAILRYRGREKTFNEGYILTTNNRMELMAAIVALEALKEHCEVILSTDSQYVRQGITQWIHNWKKRGWKTADKKPVKNVDLWKRLDAALGQHQIKWEWVKGHAGHPENERCDELARAAAMNPTQEDVGYQPEA>UPI00049EFD4A Propanediol utilization microcompartment protein PduT OS=Citrobacter amalonaticus OX=35703 GN=pduT SS=EMBLWGS:MBE0395271 PC=UP000642697:Unassembled WGS sequenceMSQAIGILELTSIAKGMEAGDAMLKSANVDLLVSKTICPGKFLLMLGGDVGAVQQAIETGNALAGEMLVDSLVLPNIHASVLPAISGLNSVDKRQAVGIVETWSVAACISAADRAVKASNVTLVRVHMAFGIGGKCYMVVAGDVSDVNNAVAVASESAGEKGLLVYRSVIPRPHEAMWRQMVEG>UPI00049EFDB6 YjcB family protein OS=Citrobacter amalonaticus OX=35703 GN=ILP74_00080 SS=EMBLWGS:MBE0393928 PC=UP000642697:Unassembled WGS sequenceMATLTTGVVLLRWQLLSAVLMFLASTLNIRFRRSDYIGLAVISSGLGVVAACWFATGLLGITLMDLSAIWNNIESVMVEVMSQTPPEWPMVLT>UPI00049EFE0A 2-hydroxy-3-oxopropionate reductase OS=Citrobacter amalonaticus OX=35703 GN=garR SS=EMBLWGS:MBE0397492 PC=UP000642697:Unassembled WGS sequenceMTMKVGFIGLGIMGKPMSKNLLKAGYSLVVSDRNPEAIADVIAAGAETASNAKAIAEQCDVIITMLPNSPHVKEVALGEGGIIEGAKPGTVLIDMSSIAPLASREISDALKAKGIDMLDAPVSGGEPKAIDGTLSVMVGGDKAIFDKYYDLLKAMAGSVVHTGDIGAGNVTKLANQVIVALNIAAMSEALTLATKAGVNPDLVYQAIRGGLAGSTVLDAKAPMVMDRNFKPGFRIDLHIKDLANALDTSHGVGAQLPLTAAVMEMMQALRADGLGTADHSALACYYEKLAKVEVTR>UPI00049EFE1B Oxygen-sensing cyclic-di-GMP phosphodiesterase OS=Citrobacter amalonaticus OX=35703 GN=dosP SS=EMBLWGS:MBE0395909 PC=UP000642697:Unassembled WGS sequenceMKLSDVKSTGDSIFIPALEQNMMGAVLINDNDEVLFFNSAAEKLWGYRREEVIGQGISMLIPHDLRAAHPDFIRHNREGGQPRVEGMSRELLLERKDGTKVWTRFALSKVNVEGKIFYLAFVRDASVEMEQKEQKRLLVLAVDHLDRPVIVLDPERRVVQCNRAFTEMFGYDITDAKGQQPDNLLNLPEEPSDNGLRLTQLLWKTTRDQDEFLVITRAGEKIWIKVSISQVYDRRNALQNLVMTFSDITEERQIRQLEGNILAAMCSSPPFHEMGDIICRNIEAVLRDTHVSLYALKDNVRQFWASSSKEIDAKEVNVWTLTIRQRDGEPAGTLLIKTAKGKETSAFIERVADISQHMAALALEQEKSRQHIEHLLQFDPLTGLPNRNHLHSYLDDLIHGAKGISPVVFLISVDHFQDVIDSLGYAVADQVLQQVINKIRERLRPDQYLSRIEGTQFVLVSDDNEMSNITQFADELINIGSEATLFDDKPFPLTLSIGISYEAGKNRDYLLSTAHNAMDFIRKAGGNGWQFFNPEMNLAVKERLQLGAALKNAIANNRLRLVYQPQIFAQTGELYGFEALARWSDPVHGHVPPNRFIPLAEETGEIENIGRWVVREACRQLSEWRQLSLTIPTLSVNLSALHFRSNQLPDQVSEAMVEFAIPGDQLTVEITESMMMEQDEEIFRRIEILRNMGVGLSIDDFGTGFSGLSRLVSLPVTELKIDKTFVDRCQTEKRIQSLLEAIITIGQSLNLIVIAEGVETKEQFELLRSMNCPVIQGYYFSRPIPADEIAAWMQTALPLKI>UPI00049EFEA7 Helix-turn-helix domain-containing protein OS=Citrobacter amalonaticus OX=35703 GN=ILP74_18540 SS=EMBLWGS:MBE0397366 PC=UP000642697:Unassembled WGS sequenceMSLPAESLIAYTLDKMNARLAASPRRDDGRIRNGLLFTGNVHDSIPRRLLLDTRLSPLDKMGWMMIRLYAQNNEGAVFPSYDELQLQLASPGKGKASRETVSRVLLMLRITGWLSLCKRVRDDKGRVRGNIYAQHDEPLTFSDAEMLDPRFLDTVADACLSKNRTISQTAREVLDDIKNDPTMRHYRSHLALIESRLDRPQTPSQMAKHHHRIPCPAPGSETELSHKRPGIAAKKQGSDTELSPERHNNSLSSESVLPVKTSSYGQVRKPNHYVRSITHSVNKNTYVPGKPVLPDSLHELVPAEDIAMLTAQLQALPEEQAQLVLLSLQKVMARQQLSNPVGWLLAVMKKAREGRLYAPRQGGDISDSVGQKTVQRPEQQQWKPEPRASARPVSEENIRDLVKKIREKIINS>UPI00049EFF69 YhdT family protein OS=Citrobacter amalonaticus OX=35703 GN=ILP74_19880 SS=EMBLWGS:MBE0397620 PC=UP000642697:Unassembled WGS sequenceMDKRFVQANKEARWALWLTLLYLAAWLAAAYIPGVTPGITGLPHWFEMACLLTPLIFILLCWAMVKFIYRDIPLEDDDAA>UPI00049EFFBB N-acetylmuramic acid 6-phosphate etherase OS=Citrobacter amalonaticus OX=35703 GN=murQ SS=EMBLWGS:MBE0394615 PC=UP000642697:Unassembled WGS sequenceMNLGALVSETRNPQTMDLDALSTLDLVHRFNQQDTLVAEAVKATLPDVASAVDAAAEALKSGGRIIYMGAGTSGRLGVLDASECPPTFGVPHGLVVGLIAGGPGALLKAVEGAEDNAQLGEDDLIALNLVPQDLVVGLAASGRTPYVIGGLKYARKTGCTTVAISCNPDSPIAQEADIAISPVVGPEALTGSTRLKSGTAQKLVLNMISTGAMVKFGKVYQNLMVDMKATNIKLMDRACRMVVEATGIARAEAETLLRQTDFDVKPAILMALSGLNADAAREKLAAHQGFLRAALADSSR>UPI00049EFFFF Ribosomal protein S18-alanine N-acetyltransferase OS=Citrobacter amalonaticus OX=35703 GN=rimI SS=EMBLWGS:MBE0394200 PC=UP000642697:Unassembled WGS sequenceMNTISSLSQSDLPAAFQIEKRAHAFPWSEHTFASNQGERYLNYQLTVEGRMAAFAITQVVLDEATLFNIAVDPDFQRRGLGKALLEHLIDELEKRGVLTLWLEVRASNVAAIALYESLGFNEATIRRNYYPTADGREDAIIMALPISM>UPI00049F0017 Two-component system response regulator GlrR OS=Citrobacter amalonaticus OX=35703 GN=glrR SS=EMBLWGS:MBE0394624 PC=UP000642697:Unassembled WGS sequenceMSRKPAHLLLVDDDPGLLKLLGMRLTSEGYSVVTAESGAEGLRVLNREKVDLVISDLRMDEMDGMQLFSEIQKVQPGMPVIILTAHGSIPDAVAATQKGVFSFLTKPVDKDALYQAIDEALEQSAPATDDSWRESIVTRSPLMLRLLEQARMVAQSDVSVLINGQSGTGKEIFAQAIHNASPRSSKPFIAINCGALPEQLLESELFGHSRGAFTGAVSNREGLFQAAEGGTLFLDEIGDMPAPLQVKLLRVLQERKVRPLGSNRDIDIDVRIISATHRDLPKAMARGEFREDLYYRLNVVSLKIPALAERTEDIPLLANHLLRQSAERHKPFVRAFSTDAMKRLMTASWPGNVRQLVNVIEQCVALTSSPVISDALVEQALEGENTALPTFVEARNQFELNYLRKLLQITKGNVTHAARMAGRNRTEFYKLLSRHELDANDFKE>UPI00049F010B Aconitate hydratase AcnA OS=Citrobacter amalonaticus OX=35703 GN=acnA SS=EMBLWGS:MBE0395722 PC=UP000642697:Unassembled WGS sequenceMSSTLREASKDTLQAKDKTYHYYSLPLAAKSLGDLTRLPKSLKVLLENLLRWQDGDSVTQDDIQALAGWLKNAHADREIAYRPARVLMQDFTGVPAVVDLAAMREAVKRLGGDTAKVNPLSPVDLVIDHSVTVDHFGDDDAFEENVRLEMERNHERYVFLKWGQQAFSRFSVVPPGTGICHQVNLEYLGKAVWSELQDGEWVAYPDTLVGTDSHTTMINGLGVLGWGVGGIEAEAAMLGQPVSMLIPDVVGFKLTGKLREGITATDLVLTVTQMLRKHGVVGKFVEFYGDGLDSLPLADRATIANMSPEYGATCGFFPIDGVTLDYMRLSGRSEDQIELVEKYAKAQGMWRNTGDEPIFTSTLALDMNDVEASLAGPKRPQDRVALGDVPKAFAASSELELNTAQKDRRPVEYVLNGQSYQLPDGAVVISAITSCTNTSNPSVLMAAGLLAKKAVTLGLKRQPWVKASLAPGSKVVSDYLAKAKLTPYLDELGFNLVGYGCTTCIGNSGPLPEPIETAIKKGDLTVGAVLSGNRNFEGRIHPLVKTNWLASPPLVVAYALAGNMNINLEKDPLGHDRKGDPVYLKDIWPSAQEIARAVDLVSSEMFRKEYAEVFEGTPEWKAINVDRSDTYGWQNDSTYIRLSPFFDEMLAQPAPVTDIHGARILAMLGDSVTTDHISPAGSIKADSPAGRYLQNHGVERKDFNSYGSRRGNHEVMMRGTFANIRIRNEMVPGVEGGMTRHLPGTEVLSIYDAAMQYQQENIPLAVIAGKEYGSGSSRDWAAKGPRLLGIRVVIAESFERIHRSNLIGMGILPLEFPQGVTRKTLGLTGEETIDIADLQQLKPGATVPVTLTRTDGNKEVVQCRCRIDTATELTYYQNDGILHYVIRNMLK>UPI00049F01B4 Choline transporter OS=Citrobacter amalonaticus OX=35703 GN=ILP74_15195 SS=EMBLWGS:MBE0396751 PC=UP000642697:Unassembled WGS sequenceMSDLPQSREKDKINPVVFYTSAGLILLFSLTTLFFSDFSAVWIGRTLNWVSQTFGWYYLLAATLYIVFVVFIACSRFGSVKLGPEQSKPEFSLLSWAAMLFAAGIGIDLMFFSVAEPVTQYMQPPEGAGQTIEAARQSMVWTLFHYGLTGWSMYALMGMALGYFSYRYNLPLTIRSALYPIFGKRINGPIGHSVDIAAVIGTIFGIATTLGIGVVQLNYGLSVLFDIPDSMAAKAALIVLSVVIATISVTSGVDKGIRVLSELNVLLALGLILFVLFMGDTSFLLNALVLNVGDYVNRFMGMTLNSFAFDRPVEWMNNWTLFFWAWWVAWSPFVGLFLARISRGRTIRQFVFGTLIIPFTFTLLWLSVFGNSALYEIIHGDAGFAQEAMVHPERGFYSLLAQYPAFTFSASVATITGLLFYVTSADSGALVLGNFTSKLKDINSDAPNWIRIFWSVAIGLLTLGMLMTNGISALQNTTVIMGLPFSFVIFFVMAGLYKSLKVEDYRRESASRHTAPRPMGVQDRLSWKKRLSRLMNYPGTRYTREMMKTVCYPAMEEVAQELRLRGARVELQSMPPEEGDNLGHLDLLVHMGDERNFIYQIWPQQYAVPGFTYRARSGKSTYYRLETFLLEGSQGNDLMDYSKEQVITDILDQYERHLNFIHLDREAPGNGVLFPGM>UPI00049F01EC Ammonia-dependent NAD(+) synthetase OS=Citrobacter amalonaticus OX=35703 GN=nadE SS=EMBLWGS:MBE0396106 PC=UP000642697:Unassembled WGS sequenceMTLQHEIIKALGAKPHINPEEEIRRSVDFLKAYLQRYPFLKSLVLGISGGQDSTLAGKLCQTAISELRAETGNDALQFIAVRLPYGVQADEQDCQDAIDFIQPDRVLTVNIKGAVLASEQALREAGIELSDFVRGNEKARERMKAQYSIAGMTSGVVVGTDHAAEAITGFFTKYGDGGTDINPLFRLNKRQGKQLLAALGCPEHLYKKAPTADLEDDRPSLPDEAALGVTYENIDDYLEGKAVDAAIAKIIEGWYLKTEHKRRPPITVFDDFWKQ>UPI00049F02A7 DedA family protein OS=Citrobacter amalonaticus OX=35703 GN=ILP74_17410 SS=EMBLWGS:MBE0397153 PC=UP000642697:Unassembled WGS sequenceMQALLEHFILQSAMYALIAVALVAFLESLALVGLILPGTVLMAGLGALIGSGELNFWHAWLAGIIGCLLGDWISFWLGWRFKKPLHRWSFMKKNKALLDKTEHALHQHSMFTILVGRFVGPTRPLVPMVAGMLDLPVAKFITPNIIGCLLWPPFYFLPGILAGAAIDIPAGMQSGEFKWLLLATAILLWFGGWLCWRLWRSGKAATDRLTHYLPKRRLLWLAPIVLGMGIVALVTLLRHPLMPMYLDILQKVVGY>UPI00049F02CE Cytochrome ubiquinol oxidase subunit I OS=Citrobacter amalonaticus OX=35703 GN=cydA SS=EMBLWGS:MBE0396599 PC=UP000642697:Unassembled WGS sequenceMLDIVELSRLQFALTAMYHFLFVPLTLGMAFLLAIMETVYVLSGKQIYKDMTKFWGKLFGINFALGVATGLTMEFQFGTNWSYYSHYVGDIFGAPLAIEGLMAFFLESTFVGLFFFGWDRLGKVQHMAVTWLVALGSNLSALWILVANGWMQNPIASDFNFETMRMEMVSFSELVLNPVAQVKFVHTVASGYVTGAMFILGISAYYLLKGRDIAFAKRSFAIAASFGMAAVLSVIVLGDESGYEMGDVQKTKLAAIEAEWETQPAPAAFTLFGIPDQDSQENKLAIQIPYALGIIATRSVDTPVIGLKDLMVQHEERIRNGMKAYELLEQLRAGSTDQAVRDQFNVMKKDLGYGLLLKRYTPNVSDATEAQIQQATKDSIPRVAPLYFAFRIMVACGFLLLAIIALSFWSVIRNRIGEKKWLLRAALYGIPLPWIAVEAGWFVAEYGRQPWAIGEVLPTAVANSSLTVGDLLFSMILICGLYTLFLVAELFLMFKFARLGPSSLKTGRYHYEQSTVASQPAR>UPI00049F0365 L-threonine 3-dehydrogenase OS=Citrobacter amalonaticus OX=35703 GN=tdh SS=EMBLWGS:MBE0397948 PC=UP000642697:Unassembled WGS sequenceMKALSKLKAEEGIWMTDVPEPEVGHNDLLIKIRKTAICGTDVHIYNWDEWSQKTIPVPMVVGHEYVGEVVGIGQEVKGFKIGDRVSGEGHITCGHCRNCRGGRTHLCRNTTGVGVNRPGCFAEYLVIPAFNAFKIPDNISDDLASIFDPFGNAVHTALSFDLVGEDVLVSGAGPIGIMAAAVAKHVGARNVVITDVNEYRLELARKMGITRAVNVSKESLNEVMDELGMTEGFDVGLEMSGAPPAFRAMLDTMNHGGRIAMLGIPPSDMSIDWTKVIFKGLFIKGIYGREMFETWYKMAALIQSGLDLSPIITHRFSIDEFQKGFDAMRSGQSGKVILSWN>UPI00049F0406 50S ribosomal protein L13 OS=Citrobacter amalonaticus OX=35703 GN=rplM SS=EMBLWGS:MBE0397592 PC=UP000642697:Unassembled WGS sequenceMKTFTAKPETVKRDWYVVDATGKTLGRLATELAIRLRGKHKAEYTPHVDTGDYIIVLNADKVAVTGNKRTDKVYYHHTGHIGGIKEATFEEMIARRPERVIEIAVKGMLPKGPLGRAMFRKLKVYAGNEHNHAAQQPQVLDI>UPI00049F0473 DeoR family transcriptional regulator OS=Citrobacter amalonaticus OX=35703 GN=ILP74_19270 SS=EMBLWGS:MBE0397503 PC=UP000642697:Unassembled WGS sequenceMSNTDASGDKRVSGTSERREQIIQRLRQQGSVQVNDLSGLFGVSTVTIRNDLAFLEKQGIAVRAYGGALICDSNTPGAEPSVEDKSSLNTAVKRSIAKAAVELIKPGHRVILDSGTTTYEIARLMRQHSDVIAMTNGMNVANALLEADGVELLMTGGHLRRQSQSFYGDQAEQSLQNYHFDMLFLGVDAIDLERGVSTHNEDEARLNRRMCEVAERIIVVTDSSKFNRSSLHKIIDTQRIHTIIVDEGIPEESLKEFHRSGVEVIIVQAAS>UPI00049F0478 Amidophosphoribosyltransferase OS=Citrobacter amalonaticus OX=35703 GN=purF SS=EMBLWGS:MBE0394907 PC=UP000642697:Unassembled WGS sequenceMCGIVGIAGVMPVNQSIYDALTVLQHRGQDAAGIITIDANNCFRLRKANGLVSDVFEARHMQRMQGNMGIGHVRYPTAGSSSASEAQPFYVNSPYGITLAHNGNLTNAHELRKKLFEEKRRHINTTSDSEILLNIFASELDNFRHYPLEADNIFAAIAATNRQIRGAYACVAMIIGHGMVAFRDPNGIRPLVLGKRDVGDGRTEYMVASESVALDTLGFEFLRDVASGEAVYITEKGQLFTRQCAENPVSNPCLFEYVYFARPDSFIDKISVYSARVNMGTKLGEKIAREWEDLDIDVVIPIPETSCDIALEIARILGKPYRQGFVKNRYVGRTFIMPGQQLRRKSVRRKLNANRAEFRDKNVLLVDDSIVRGTTSEQIIEMAREAGAKKVYLASAAPEIRFPNVYGIDMPTANELIAHGREVDEIRQIIGADGLIFQDLDDLIDAVRTENPDIQQFECSVFNGVYVTKDVDQQYLDFLDSLRNDDAKAVQLQNEVENLEMHNEG>UPI00049F047C Arginine ABC transporter substrate-binding protein OS=Citrobacter amalonaticus OX=35703 GN=artJ SS=EMBLWGS:MBE0396432 PC=UP000642697:Unassembled WGS sequenceMKKVLIAALIAGFSLSATAAQTIRFATEASYPPFESMDANNKIVGFDVDLANALCKEIDATCTFTNQAFDSLIPSLKFRRFDAVMAGMDITPEREKQVLFTTPYYDNSALFIGQQGKFTGVDQLKGKKVGVQNGTTHQKFIMDKHPEITTVPYDSYQNAKLDLQNGRIDSVFGDTAVVTEWLKDNPKLAPVGDKVTDKDYFGTGLGIAVRQGNEELQQKFNTALEKVKKDGTYETIYNKWFQK>UPI00049F0512 YoaK family small membrane protein OS=Citrobacter amalonaticus OX=35703 GN=yoaK SS=EMBLWGS:MBE0396153 PC=UP000642697:Unassembled WGS sequenceMRLGILFPVVIFITAVVFLAWFFIGGYAAPGA>UPI00049F057A DNA-binding transcriptional regulator OS=Citrobacter amalonaticus OX=35703 GN=ILP74_14920 SS=EMBLWGS:MBE0396697 PC=UP000642697:Unassembled WGS sequenceMSENSKPKGHFYEHRPEDKPQIFRTLRNIDLNLLTIFEAVYVHKGIVNAAKVLNLTPSAISQSIQKLRTIFPDPLFIRKGQGVTPTTYATHLHEYISQGLESILGALDLTGSYDKQRTITIGTTPSIGALVMPTIYHAIKTSHPQLHLRNIPVHDAETQLSQFQSDLIIDNHIYSNRAVENHVLFSDKIELVCRQNHPSLKRALTPEALDDAEHSLLMIEGQSFTLLRQRIQELFPDRQISFSSYNIFTLASLIAHSDLVGIMPTRFFTLFSHCWPLQRIPHPSLNHEKIDFSLHYNKLSLRDPVLENVIAVIRDAF>UPI00049F066F Enoyl-ACP reductase FabI OS=Citrobacter amalonaticus OX=35703 GN=fabI SS=EMBLWGS:MBE0395735 PC=UP000642697:Unassembled WGS sequenceMGFLSGKRILVTGLASKLSIAWGIAQAMHREGAELAFTYQNDKLKGRVEEFAAQLGSDIVLQCDVAEDESIDTLFAELAKAWPKFDGFVHSIGFAPGDQLDGDYVNAVTREGFKIAHDISSYSFVAMAKACRSMLNPGSALLTLSYLGAERAIPNYNVMGLAKASLEANVRYMANAMGPEGVRVNAISAGPIRTLAASGIKDFRKMLAHCEAVTPIRRTVTIEDVGNSAAFLCSDLSGGISGEVVHVDGGFSIAAMNELELK>UPI00049F0697 Ni(II)/Co(II) efflux transporter accessory subunit RcnB OS=Citrobacter amalonaticus OX=35703 GN=rcnB SS=EMBLWGS:MBE0395147 PC=UP000642697:Unassembled WGS sequenceMKNKLVVGALLLASSAVWAAPATAVPAKGIDQYELSGFIADFTHFKPGDTVPEMYRTDEYTIKQWNLRNLPAPDAGTHWTYMGGAYVLINDADGKIIKAYDGEIFYHR>UPI00049F06A2 23S rRNA pseudouridine(2605) synthase RluB OS=Citrobacter amalonaticus OX=35703 GN=rluB SS=EMBLWGS:MBE0395714 PC=UP000642697:Unassembled WGS sequenceMSEKLQKVLARAGHGSRREIESIIAAGRVSVDGKIATLGDRVEVTPGLKIRIDGHLISVKESAEQICRVLAYYKPEGELCTRNDPEGRPTVFDRLPKLRGARWIAVGRLDVNTCGLLLFTTDGELANRLMHPSREVEREYAVRVFGQVDDAKLRDLSRGVQLEDGPAAFKTIKFSGGEGINQWYNVTLTEGRNREVRRLWEAVGVQVSRLIRVRYGDIPLPKGLPRGGWTELDLAQTNYLRELVELEPETTSKVAVEKDRRRMKANQIRRAVKRHSQVSGGRRAGGRNNNG>UPI00049F06E7 PhnA family protein OS=Citrobacter amalonaticus OX=35703 GN=ILP74_00290 SS=EMBLWGS:MBE0393967 PC=UP000642697:Unassembled WGS sequenceMSLPHCPQCNSEYTYEDNGMYICPECAHEWNDAEPAHDSDELIVKDANGNLLADGDSVTVVKDLKVKGSSSMLKIGTKVKNIRLVEGDHNIDCKIDGFGPMKLKSEFVKKN>UPI00049F06FC GNAT family acetyltransferase OS=Citrobacter amalonaticus OX=35703 GN=ILP74_04395 SS=EMBLWGS:MBE0394730 PC=UP000642697:Unassembled WGS sequenceMEIRVFRQEDFEEVITLWERCDLLRPWNDPEMDIERKVNHDVSLFLVAEVNGEVVGTVMGGYDGHRGSAYYLGVHPEFRGRGIANALLNRLEKKLIARGCPKIQIMVREDNDVVLGMYERLGYEHADVLCLGKRLIEDEEY>UPI00049F070D Kinase OS=Citrobacter amalonaticus OX=35703 GN=ILP74_16010 SS=EMBLWGS:MBE0396907 PC=UP000642697:Unassembled WGS sequenceMAILSIGFSCFDQFFFLNEWPQENTKNFCHDFIESGGGPAANAAWLLGLWGEDVYYIGHLNQDLYGQRIIDEFAQAGVDTSQVVFSDEMITPLASVLVNRLTGSRTIITRKMQTPPSLTYDQKLKLDDLAERLIASDEPVTLLIDGHEAEISEYLIKKLPSARVVMDGGSLRDSNIKLAAWTDYFVVSEHFARDYMGYRALSTEAEIKAALIELNKICRGEAFITLGEKGCALLKNGMLQIVPAWLCNAVDTTGAGDVFHGAFTYGVHYSWHIDNIILFASLTAAISIEKKGVRESMPDLAVVHNSLNSYERNLKQYFGE>UPI00049F075C Citrate synthase OS=Citrobacter amalonaticus OX=35703 GN=ILP74_14550 SS=EMBLWGS:MBE0396632 PC=UP000642697:Unassembled WGS sequenceMADTKAKITLNGDTAIELDVLKGTLGQDVIDIRSLGSKGMFTFDPGFTSTASCESKITFIDGDEGILLHRGFPIDQLATESNYLEVCYILLNGEKPTQAEYDAFRTTVTRHTMIHEQITRLFHAFRRDSHPMAVMCGITGALAAFYHDSLDVNNPRHREIAAFRLLSKMPTMAAMCYKYSIGQPFVYPRNDLSYAGNFLNMMFSTPCETYEVNPVLERAMDRILILHADHEQNASTSTVRTAGSSGANPFACIAAGIASLWGPAHGGANEAALKMLEEISSVKHIPEFVRRAKDKNDSFRLMGFGHRVYKNYDPRATVMRETCHEVLKELGTKDDLLEVAMELEHIALNDPYFIEKKLYPNVDFYSGIILKAMGIPSSMFTVIFAMARTVGWIAHWSEMHTDGMKIARPRQLYTGYEKRDFKSDLKH>UPI00049F079B Trigger factor OS=Citrobacter amalonaticus OX=35703 GN=tig SS=EMBLWGS:MBE0396865 PC=UP000642697:Unassembled WGS sequenceMQVSVETTQGLGRRVTITIAADSIETAVKSELVNVAKKVRIDGFRKGKVPMNVVAQRYGASVRQDVLGDLMSRNFVDAIIKEKINPAGAPNYVPGEYKLGEDFTYAVEFEVYPEVELTGLDTIEVEKPVVEVTDADVDVMLDTLRKQQATWKDKDGAADAEDRVTLDFTGSVDGEEFEGGKASDFVLAMGQGRMIPGFEDGIKGHKAGEEFTIDVTFPEDYHAENLKGKAAKFAINLKKVEERELPELTEEFIKRFGVEDGSVAGLRTEVRKNMERELKGAVRNRVKSQAIDGLVKANDIDVPAALIDSEIDVLRRQAAQRFGGNEKQALELPRELFEEQAKRRVVVGLLLGEVIRTNELKADEDRVKGLIEEMASAYEDPKEVIEFYSKNKELMDNMRNVALEEQAVEAVLAKAKVTEKATTFNELMNQQA>UPI00049F07C3 Glycerol uptake facilitator protein GlpF OS=Citrobacter amalonaticus OX=35703 GN=glpF SS=EMBLWGS:MBE0398327 PC=UP000642697:Unassembled WGS sequenceMSQTSTLKGQCIAEFLGTGLLIFFGVGCVAALKVAGASFGQWEISVIWGLGVAMAIYLTAGVSGAHLNPAVTIALWLFACFDKRKVVPFILSQFAGAFCAAALVYGLYYNLFADFEQTHHIVRGSIESVDLAGTFSTYPNPHINFVQAFAVEMVITAILMGLILALTDDGNGVPRGPLAPLLIGLLIAVIGASMGPLTGFAMNPARDIGPKAFAWLAGWGNVAFTGGKDIPYFLVPLFGPIVGAILGAFAYRKLIGRHLPCDICAVEEKETTTTSQQKASL>UPI00049F081F Formate C-acetyltransferase OS=Citrobacter amalonaticus OX=35703 GN=pflB SS=EMBLWGS:MBE0396393 PC=UP000642697:Unassembled WGS sequenceMSELNEKLATAWEGFTKGDWQNEVNVRDFIQKNYTPYEGDESFLAGATDATTALWDSVMEGVKQENRTHAPVDFDTSVASTITSHDAGYINKALEKIVGLQTEAPLKRAIIPFGGIKMVEGSCKAYNRELDPMLKKIFTEYRKTHNQGVFDVYTKDILNCRKSGVLTGLPDAYGRGRIIGDYRRVALYGIDFLMKDKYAQFVSLQSDLENGVNLEATIRLREEIAEQHRALGQIKEMAAKYGCDISGPATNAQEAIQWTYFGYLAAVKSQNGAAMSFGRVSTFLDAYIERDLKAGKITEQDAQEMIDHLVMKLRMVRFLRTPEYDELFSGDPIWATESIGGMGVDGRTLVTKNSFRFLNTLYTMGPSPEPNITVLWSEKLPLNFKKFAAKVSIDTSSLQYENDDLMRPDFNNDDYAIACCVSPMVVGKQMQFFGARANLAKTMLYAINGGVDEKLKMQVGPKSEPIKGDVLNYDEVMERMDHFMDWLAKQYVTALNVIHYMHDKYSYEASLMALHDRDVIRTMACGIAGLSVAADSLSAIKYAKVKPIRDEDGLAIDFEIEGEYPQFGNNDARVDDMAVDLVERFMKKIQKLKTYRDAIPTQSVLTITSNVVYGKKTGNTPDGRRAGAPFGPGANPMHGRDQKGAVASLTSVAKLPFAYAKDGISYTFSIVPNALGKDDEVRKTNLAGLMDGYFHHEASIEGGQHLNVNVMNREMLLDAMEHPEKYPQLTIRVSGYAVRFNSLTKEQQQDVITRTFTQTM>UPI00049F083A Glutathione-disulfide reductase OS=Citrobacter amalonaticus OX=35703 GN=gorA SS=EMBLWGS:MBE0397855 PC=UP000642697:Unassembled WGS sequenceMSKHYDYIAIGGGSGGIASINRAAMYGQKCALIEAKALGGTCVNVGCVPKKVMWHAAQIREAIHLYGPDYGFDTTINKFNWDTLIASRTAYIDRIHTSYDNVLGKNNVDVIKGFARFVDAKTIEVNGETITADHILIATGGRPSHPDIPGVEYGIDSDGFFELPALPERVAVVGAGYIAVELAGVINGLGAKTHLFVRKHAPLRSFDPMITETLVEVMNAEGPTLHTHAIPKAVVKNADGSLTLELEDGRSETVDCLIWAIGREPATDNFNLAATGVKTNDKGYIVVDKYQNTSVDGIYAVGDNTGAVELTPVAVAAGRRLSERLFNNKPDEHLDYSNIPTVVFSHPPIGTVGLTEPQAREQYGDAQVKVYKSSFTAMYTAVTSHRQPCRMKLVCVGPEEKIVGIHGIGFGMDEMLQGFAVALKMGATKKDFDNTVAIHPTAAEEFVTMR>UPI00049F08BA PTS sugar transporter subunit IIB OS=Citrobacter amalonaticus OX=35703 GN=ILP74_24190 SS=EMBLWGS:MBE0398424 PC=UP000642697:Unassembled WGS sequenceMNITLARIDDRLIHGQVTTVWSKVANAQRIIICNDDVYNDEVRRTLLRQAAPPGMKVNVVNIEKAVAVYHNPQYQDETVFYLFTRPQDALAMVKQGVKIATLNIGGMAWRPGKKQLTKAVSLDDDDINAFRELDKFGVTLDLRVVASDPSINILEKIKEQSFAE>UPI00049F08E9 Phosphate ABC transporter permease PstA OS=Citrobacter amalonaticus OX=35703 GN=pstA SS=EMBLWGS:MBE0398087 PC=UP000642697:Unassembled WGS sequenceMATLEMQTSAELAESRRKMQAKRRMKNRIALTLSMATMAFGLFWLVWILMSTISRGIDGMSLALFTEMTPPPNTAGGGLANALAGSGLLILWATVFGTPLGIMAGIYLAEYGRKSWLAEVIRFINDILLSAPSIVVGLFVYTIVVAQMEHFSGWAGVIALALLQVPIVIRTTENMLKLVPDSLREAAYALGTPKWKMISAITLKASVSGIMTGILLAIARIAGETAPLLFTALSNQFWSTDMMQPIANLPVTIFKFAMSPFAEWQQLAWAGVLIITLCVLLLNILARVIFAKSKHG>UPI00049F094C Uncharacterized protein OS=Citrobacter amalonaticus OX=35703 GN=ILP74_11310 SS=EMBLWGS:MBE0396024 PC=UP000642697:Unassembled WGS sequenceMEIDLDNLVFNGLEEAQERNAERLDDADKKAESVIADDDCGDACKI>UPI00049F0A5D Helix-turn-helix domain-containing protein OS=Citrobacter amalonaticus OX=35703 GN=ILP74_15285 SS=EMBLWGS:MBE0396766 PC=UP000642697:Unassembled WGS sequenceMKPDTHVINIINAVSDTSRILHARERQIISLGHHAESMTFVLHKGTVSVYRSHDQLLLKHLKAPMIVGMNSLMNTNADFFYKANGEILFEIIPTLHVMSVIAEKNLWQDASYTYMYAIDKLLEAHRTSAGLSTYELIRTNLIALLAEKEELRLAVNASDYIQEKTHLSRSRVMKILSDLRLGGHIEMARGILTKINRLPEQY>UPI00049F0B51 Carbamate kinase OS=Citrobacter amalonaticus OX=35703 GN=ILP74_17860 SS=EMBLWGS:MBE0397239 PC=UP000642697:Unassembled WGS sequenceMKKKLVIALGGNALLQRGEILSAENQQRSIQVFAQRVATLARDYQLVIVHGNGPQVGLLALQNAAYTESPAWPLDILVAESQGMIGAAIAQALTQNTGGCPVTTLMTRVEVDPQDEAFAAPGKYIGPVYQPDQQAELEQRYGWTMKADGQYIRRVVPSPIPQNILDGEAIQMLMEAGHTVICCGGGGVPVVAQGNGYVGTEAVIDKDLTAAVLANTINADHLLILTDADAVYEHWGTPQARALRHVTTEELAPFAAPDGAMGPKAAAVIQFVKQTGRSAFIGALKDAPQILTGEKGTCVTQ>UPI00049F0BEF GntP family permease OS=Citrobacter amalonaticus OX=35703 GN=ILP74_12870 SS=EMBLWGS:MBE0396317 PC=UP000642697:Unassembled WGS sequenceMPLIIVVAGIALLLLLTIKIKLNTFVSLIIVSIVVAIASGMDLNKVVTSVESGLGGTLGHIGLIFGFGVMLGRLLADAGGAQRIALTMLNYFGKKRLDWAVVCSAFIVGIALFFEVGLILLVPILFAIAREAKISPMFMCVPMLSGLLVAHGFLPPHPGPTVIAREYGADVGLVLIYGIIVGIPTFILCGPILNKFCQRIIPDAFKKEGNIASLGATRRFSENEMPGFGISFLTAMLPVILMAVVTIIQMTHAKNASESGTFYNVLLFLGNSTIAMLISLLFAIYTMGLGRGKTIPELMDSCGKAIAGIAGLLLIIGGGGAFKQVLIDSGVGQYISTLVSGMDINPILMAWGVAAFLRICLGSATVAAISTAGLVIPLLAVHPNTNLALITLATGAGSCICSHVNDASFWMIKDFFGLTTKETLLSWTLMSTLLSICGLIFILLASMVL>UPI00049F0C06 Sigma-E factor regulatory protein RseB OS=Citrobacter amalonaticus OX=35703 GN=rseB SS=EMBLWGS:MBE0394605 PC=UP000642697:Unassembled WGS sequenceMKQLWFAMSLVTGSLFFSVNASANTASGALLQQMNVASQSLNYELSFVSITKQGVESLRYRHARLENRPLAQLLQMDGPRREVVQRGNEISYFEPGLEPFTLNGDYIVDSLPSLIYTDFKRLAPYYDFIAVGRTRIADRLCEVIRVVARDGTRYSYIVWMDTETKLPMRVDLLDRDGETLEQFRVIAFTVNQDAGSGMQTLAKASLPPLLSVPAGEKAKFSWNPTWLPKGFSEVSSGRRPLPTMDNMPIESRLYSDGLFSFSINVNRATQASTDQMLRTGRRTVSTSVRDNAEITIVGELPPQTAKRIADTIKFGAAQ>UPI00049F0C33 Large-conductance mechanosensitive channel protein MscL OS=Citrobacter amalonaticus OX=35703 GN=mscL SS=EMBLWGS:MBE0397667 PC=UP000642697:Unassembled WGS sequenceMSIIKEFREFAMRGNVVDLAVGVIIGAAFGKIVSSLVADIIMPPLGLLIGGIDFKQFAVTLRDAQGDIPAVVMHYGVFIQNIFDFVIVAFAIFMAIKLINKLNRKKEEPAAAPAPTKEEVLLTEIRDLLKEQNNRS>UPI00049F0C7B Acetyl-CoA carboxylase biotin carboxylase subunit OS=Citrobacter amalonaticus OX=35703 GN=accC SS=EMBLWGS:MBE0397617 PC=UP000642697:Unassembled WGS sequenceMLDKIVIANRGEIALRILRACKELGIKTVAVHSSADRDLKHVLLADETVCIGPAPSVKSYLNIPAIISAAEITGAVAIHPGYGFLSENANFAEQVERSGFIFIGPKADTIRLMGDKVSAITAMKKAGVPTVPGSDGPLTDDMDANRAHAKRIGYPVIIKASGGGGGRGMRVVRGDAELAQSISMTKAEAKAAFSNDMVYMEKYLENPRHIEIQVLADGQGNAIYLAERDCSMQRRHQKVVEEAPAPGITPELRRFIGERCAKACVDIGYRGAGTFEFLFENGEFYFIEMNTRIQVEHPVTEMITGVDLIKEQLRIAAGQPLSIKQEEVQVKGHAVECRINAEDPNTFLPSPGKITRFHAPGGFGVRWESHIYAGYTVPPYYDSMIGKLICYGETRDVAIARMKNALQELIIDGIKTNVDLQTRIMNDEHFQQGGTNIHYLEKKLGLTEK>UPI00049F0D94 YceK/YidQ family lipoprotein OS=Citrobacter amalonaticus OX=35703 GN=ILP74_22170 SS=EMBLWGS:MBE0398060 PC=UP000642697:Unassembled WGS sequenceMIRNVLLTLMMCSGMVLLSGCSSVMSHTGGKEGTYPGTRASATMIGDDETNWGTKSLAILDMPFTAVMDTILLPWDLFRKDNSVRSRVERSEERTRITNSVIPPAKMPAP>UPI00049F0DF1 Ethanolamine utilization ethanol dehydrogenase EutG OS=Citrobacter amalonaticus OX=35703 GN=eutG SS=EMBLWGS:MBE0394720 PC=UP000642697:Unassembled WGS sequenceMQAELQTALFQAFDTLNLQRMKTFSVPPVTLCGLGALSSCGQEAQSRGLSHLFVMVDSFLHQAGMTASLERSLAMKGVAMTVWPCPMGEPCITDVCAAVAQLRESKCDGVVAFGGGSVLDAAKAVALLVTNPHQTLAQMTEYSSLRPRLPLIAVPTTAGTGSETTNVTVIIDAATGRKQVLAHATLMPDVAILDAALTEGVPPHVTAMTGIDALTHAVEAYSALNASPFTDSLAIGAIAMIGKSLPKAVGYGHDLAARESMLLASCMAGMAFSSAGLGLCHAMAHQPGATLHIPHGQANAMLLPTVMGFNRMVCRERFSHIGRALTNKKADDRDAIAAVSELIAEVGLNKRLADVGAKPEHYSVWAQAALEDICIRSNPRTATQSNIIELYAAAQ>UPI00049F0DFC OmpA family lipoprotein OS=Citrobacter amalonaticus OX=35703 GN=ILP74_21310 SS=EMBLWGS:MBE0397892 PC=UP000642697:Unassembled WGS sequenceMKKRVLVIAAIVSGALAVSGCTTNPYTGEREAGKSGIGAGIGSLVGAGIGALSSSKKDRGKGALIGAAAGAALGGGVGYYMDVQEAKLRDKMQGTGVSVTRSGDNIILNMPNNVTFDSSSATLKPAGANTLTGVAMVLKEYEKTAVNVIGYTDSTGGQDLNMRLSQQRADSVASALITQGVAANRIRTSGMGPANPIASNSTAEGKAQNRRVEITLSPIQ>UPI00049F0E01 Xanthine phosphoribosyltransferase OS=Citrobacter amalonaticus OX=35703 GN=gpt SS=EMBLWGS:MBE0396978 PC=UP000642697:Unassembled WGS sequenceMSEKYVVTWDMLQIHARKLANRLMPSEQWKGIIAVSRGGLVPGALLARELGIRHVDTVCISSYDHDNQRELKVLKRAEGDGEGFIVIDDLVDTGGTAVAIREMYPKAHFVTIFAKPAGRPLVDDYVIDIPQDTWIEQPWDMGVTFVPPISGR>UPI00049F0E5E YbgS-like family protein OS=Citrobacter amalonaticus OX=35703 GN=ILP74_14290 SS=EMBLWGS:MBE0396585 PC=UP000642697:Unassembled WGS sequenceMKMTKLASLFLTATLSLASGAALAAETSAQTNNGQANAAADAGQVAPDAKENVAPNNVDNDNINSGGTMLHPNGSSMNHEGMTKDEVHKNTMCKDGRCPDVNKKVETGNGINNDVDTKTDGTTQ>UPI00049F0E90 6-phosphofructokinase OS=Citrobacter amalonaticus OX=35703 GN=ILP74_23180 SS=EMBLWGS:MBE0398242 PC=UP000642697:Unassembled WGS sequenceMKIGIVISGGDVSGINNFIFQIARLADAKITLFNGGIPGLLEKNHHDVAWRDLVDFSIAAIPLITSGRTTRKLQRNEYETIARKLKSLHIDVLIMAGGDGSLQFLNTLSEFDINCFGVGMTIDNDVYGSHYTIGFSTACEQIIKEVSRLRNTGRALPGRVFMVELLGGYCGELTLQSAIKCNADIALIPECQMSLDALAERVRQKLTVQNSVIILCSEGYTREYSPGFQGAIDTMIKQLEPRIGERIRKTIVGYGLRNGDPTCEEIYQGTIMASEVVRCIQSGMKNKAIIINQSNKPIPIDLVSMKKRLVDTEGHHYKLAKQLNII>UPI00049F0ECA Preprotein translocase subunit SecA OS=Citrobacter amalonaticus OX=35703 GN=secA SS=EMBLWGS:MBE0397123 PC=UP000642697:Unassembled WGS sequenceMLIKLLTKVFGSRNDRTLRRMRKAVAVINAMEPEMEKLSDDELKAKTGEFRARLEKGESVESLIPEAFAVVREASKRVFGMRHFDVQLLGGMVLNDRCIAEMRTGEGKTLTATLPAYLNALSGKGVHVVTVNDYLAQRDAENNRPLFEFLGMTVGINLPGMPAPAKREAYAADITYGTNNEYGFDYLRDNMAFSPEERVQRKLHYALVDEVDSILIDEARTPLIISGPAEDSSEMYKKVNKIIPHLIRQEKEDSDSFQGEGHFSVDEKARQVNLTERGLVLIEELLVKEGIMDEGESLYSPGNIMLMHHVTAALRAHALFTRDVDYIVKDGEVIIVDEHTGRTMQGRRWSDGLHQAVEAKEGVDIQNENQTLASITFQNYFRLYEKLAGMTGTADTEAFEFSSIYKLDTVVVPTNRPMIRKDMPDLVYMTEAEKIQAIIEDIKERTANGQPVLVGTISIEKSEVVSNELTKAGIKHNVLNAKFHANEAAIVAQAGYPAAVTIATNMAGRGTDIVLGGSWQAEVAALENPTPEQIAQIKADWQVRHEAVLASGGLHIIGTERHESRRIDNQLRGRSGRQGDAGSSRFYLSMEDALMRIFASDRVSGMMRKLGMKPGEAIEHPWVTKAIANAQRKVESRNFDIRKQLLEYDDVANDQRRAIYTQRNELLDVSDVSETINSIREDVFKATIDAYIPPQSLEEMWDIPGLQERLKNDFDLEMPIAEWLDKEPELHEETLRERILAQAIDVYQRKEEVVGAEMMRHFEKGVMLQTLDSLWKEHLAAMDYLRQGIHLRGYAQKDPKQEYKRESFSMFASMLESLKYEVISTLSKVQVRMPEEVEAMEQQRREEAERLAQMQQLSHQDDDSAAAADLAAQTGERKVGRNDPCPCGSGKKYKQCHGRLS>UPI00049F0EED D-glycero-beta-D-manno-heptose 1,7-bisphosphate 7-phosphatase OS=Citrobacter amalonaticus OX=35703 GN=gmhB SS=EMBLWGS:MBE0397026 PC=UP000642697:Unassembled WGS sequenceMAKSVPAIFLDRDGTINVDHGYVHEIDEFEFIDGVIDAMRELKTLGYALVVVTNQSGIARGKFTEAQFETLTEWMDWSLADRDVDLDGIYYCPHHPQGSVEEYRQVCDCRKPHPGMLISARDFLHIDMAASYMVGDKLEDMQAAAAAGVGTKVLVRTGKPITAEAENAADWVLNSLADLPSAIKKQQK>UPI00049F0F44 GntR family transcriptional regulator OS=Citrobacter amalonaticus OX=35703 GN=ILP74_10865 SS=EMBLWGS:MBE0395942 PC=UP000642697:Unassembled WGS sequenceMTVETQLNPTQPVNQQIYRILRRDIVHCLIAPGTPLSEKEVSVRFDVSRQPVREAFIKLAENGLIQIRPQRGSYVNKISLSQVRNGCFVRQAIECAVARRAAAQITDSQCYQLEQNLNQQRIAIERKQLNDFFELDDNFHQKLATIADCQLAWDTIENIKATIDRVRYMSLDHVSPPEMLLRQHLDIFSALEKHDADAVEKAMTQHLQEIGESVLLIRQENSDWFSEE>UPI00049F0FC9 Conjugal transfer protein TraG N-terminal domain-containing protein OS=Citrobacter amalonaticus OX=35703 GN=ILP74_18360 SS=EMBLWGS:MBE0397330 PC=UP000642697:Unassembled WGS sequenceMTTNSYLEYFLTLLGWVINNGLWNVLLSTGLFVLPLAFKVVGIWLKVREEGEDEGNKGMLSLPRIENALYAGFLVMIACCVPLINVSLSTMQYDTTRAKSCGVWTPKAPDESGYAGVVTSLNDQTAAAPVWWVLIHKLSKGVTQAAVATIPCRPDMRQIRFEVQHTRIDNKALAQELQDFTNDCYSVALYLWKQQDQGQTKDKTTLRDIEWIGSSTFLSRYYPSLQSKLPRAAFPWTDSRDSGRPNTGRGGYPTCSEWWSSADTGLKARVKDQADPDMWLRISAAMKMSGFNDSDYQEAVIRRLVSPESLTVSQNGHVYAGYGGNADFTLDNAAARVASVAGTSLGSLAAFPAFDAMRQALPMVQAILLMAIYVMLPLILAFAAYEFKTVITLTFVMFALNFLTFWWELARWLDSWLLTALYSSDTHSRFNMAGLQNSTDDLIMNLVMGTMFLVLPAVWLGALSWAGVTIGNALSSAYQQGTAKAESSGGKLGEAGANAVANKAMK>UPI00049F0FFE Carboxymuconolactone decarboxylase family protein OS=Citrobacter amalonaticus OX=35703 GN=ILP74_23705 SS=EMBLWGS:MBE0398342 PC=UP000642697:Unassembled WGS sequenceMHSERFITGQNMLQQVDGKGGDAVVESLKDIAPDFARYLIEFPFGDIYSRPGLDLRSREIATVAALTALGNAAPQLKVHIAAALHVGLTQEEIIEVMMQMAVYAGFPAALNGLFAAKEVFAGR>UPI00049F104C Phosphoglycolate phosphatase OS=Citrobacter amalonaticus OX=35703 GN=gph SS=EMBLWGS:MBE0397735 PC=UP000642697:Unassembled WGS sequenceMDKLQDIRGVAFDLDGTLVDSAPGLSAAVDMALYALELPVAGEERVITWIGNGADVLMERALTWSRQERATLRKTMGKPPVDEDIPAEEQVRILRKLFDRYYGDVAEEGTFLFPDVADTLGALHAKGLPLGLVTNKPTPFVAPLLDALDISKYFSVVIGGDDVQNKKPHPDPLLLVASKLGIAPEQLLFVGDSRNDIQAAKAAGCPSVGLTYGYNYGEPIALSQPDVMYDRINELLPALGLPHSENQESKND>UPI00049F1069 Glycerate 2-kinase OS=Citrobacter amalonaticus OX=35703 GN=garK SS=EMBLWGS:MBE0397491 PC=UP000642697:Unassembled WGS sequenceMKIVIAPDSYKESLSATEVAQAIEKGFREIFPDAQYVSVPVADGGEGTVEAMIAATHGSAHSALVTGPLGEKVNANWGMSGDGKTAFIEMAAASGLALVPPEKRNPLITTSRGTGELILQALDSGASNIIIGIGGSATNDGGAGMVQALGAKLCDANGTEIGYGGGSLNTLNTIDVSGLDPRIKHCAIRVACDVTNPLVGEQGASRIFGPQKGATEPLILELDRNLAHYADIIKKSLGVDVKNVPGAGAAGGMGAALMAFLGAELKSGIEIVTQALNLEEHIHDCTLVVTGEGRIDSQSIHGKVPVGVANVAKKYHKPVIGIAGSLTRDVGVVHQYGIDAVFSVLTSIGTLEEAFRGAFDNIYRASRNIAATLAVGMRSAG>UPI00049F1095 Elongation factor G OS=Citrobacter amalonaticus OX=35703 GN=fusA SS=EMBLWGS:MBE0397703 PC=UP000642697:Unassembled WGS sequenceMARTTPIARYRNIGISAHIDAGKTTTTERILFYTGVNHKIGEVHDGAATMDWMEQEQERGITITSAATTAFWSGMAKQYEPHRVNIIDTPGHVDFTIEVERSMRVLDGAVMVYCAVGGVQPQSETVWRQANKYKVPRIAFVNKMDRMGANFLKVVGQIKTRLGANPVPLQLAIGAEEAFTGVVDLVKMKAINWNEADAGVTFTYEDIPADMQELAEEWHQNLIESAAEASEELMEKYLGGEELTEEEIKKALRQRVLNNEIILVTCGSAFKNKGVQAMLDAVIDYLPSPVDVPAINGILDDGKDTPAERHASDEEPFSALAFKIATDPFVGNLTFFRVYSGVVNSGDTVLNSVKAARERFGRIVQMHANKREEIKEVRAGDIAAAIGLKDVTTGDTLCDPDAPIILERMEFPEPVISIAVEPKTKADQEKMGLALGRLAKEDPSFRVWTDEESNQTIIAGMGELHLDIIVDRMKREFNVEANVGKPQVAYREAIRAKVTDIEGKHAKQSGGRGQYGHVVIDMYPLEPGSNPKGYEFINDIKGGVIPGEYIPAVDKGIQEQLKAGPLAGYPVVDMGVRLHFGSYHDVDSSELAFKLAASIAFKEGFKKAKPVLLEPIMKVEVETPEENTGDVIGDLSRRRGMLRGQESEVTGVKIHAEVPLSEMFGYATQLRSLTKGRASYTMEFLKYDDAPNNVAQAVIEARGK>UPI00049F109F 4-hydroxy-tetrahydrodipicolinate reductase OS=Citrobacter amalonaticus OX=35703 GN=dapB SS=EMBLWGS:MBE0397186 PC=UP000642697:Unassembled WGS sequenceMHDAQVRVAIAGAGGRMGRQLIQAALQMDGVALGAALEREGSSLLGSDAGELAGVGKTGVTVQSRLEAVKDDFDVFIDFTRPEGTLNHLAFCRQHGKGMVIGTTGFDDAGKQAIRDAAQDIGIVFAANFSVGVNVMLKLLEKAAKVMGDYTDIEIIEAHHRHKVDAPSGTALAMGEAIAHALDKDLKECAVYSREGYTGERVPGTIGFATVRAGDIVGEHTAMFADIGERVEITHKASSRMTFANGAVRSALWVSAKKNGLFDMRDVLDLNNL>UPI00049F1127 Hsp33 family molecular chaperone HslO OS=Citrobacter amalonaticus OX=35703 GN=hslO SS=EMBLWGS:MBE0397751 PC=UP000642697:Unassembled WGS sequenceMPQHDQLHRYLFENFAVRGELVTVSETLQQILENHTYPQPVKTVLAELLVATSLLTATLKFAGDITVQLQGDGPLSLAVINGNNNQQMRGVARVQGEIPEDADLKTLVGNGYLVITITPEEGERYQGVVGLEGDTLAACLEDYFLRSEQLPTRLFIRTGDVDGKAAAGGMLLQVMPAQDAQADDFNHLATLTETIKAEELLTLPANEVLWRLYHEEEVTLYDPQDVEFKCTCSRERCAGALKTLPDEEVDSILAEEGEIDMHCDYCGSHYLFNAMDIAEIRNNASPADPQVH>UPI00049F12E2 YijD family membrane protein OS=Citrobacter amalonaticus OX=35703 GN=ILP74_23860 SS=EMBLWGS:MBE0398371 PC=UP000642697:Unassembled WGS sequenceMKQSVQDKGTLLLALVAGLSINGTFAALFSSIVPFSVFPIISLVLTVYCLHQRYLNRTMPVGLPGLAAACFILGVLLYSTVVRAEYPDIGSNFFPAVLSVILVFWIGVKMRNRKQEVSE>UPI00049F1397 DNA polymerase III subunit psi OS=Citrobacter amalonaticus OX=35703 GN=ILP74_01525 SS=EMBLWGS:MBE0394199 PC=UP000642697:Unassembled WGS sequenceMTTRRDWQLQQLGITQWSLRRPGALQGEIAISIPAHVRLVMVGETLPPLTEPLVSDILRALTLSPDQVLQVTPERVAMLPQGSRCNSWRLGTDAPLTLEGAQIATPAFDELRVNPTARAALWQQICTHEHDFFPQSE>UPI00049F139F Sec-independent protein translocase subunit TatC OS=Citrobacter amalonaticus OX=35703 GN=tatC SS=EMBLWGS:MBE0398186 PC=UP000642697:Unassembled WGS sequenceMAVEDTQPLITHLIELRKRLLNCIIAVIVIFLALVYFANDIYHLVSSPLIKQLPQGATMIATDVASPFFTPIKLTFMVSLILSAPVILYQVWAFIAPALYKHERRMVVPLLISSSLLFYIGMAFAYFVVFPLAFGFLANTAPEGVQVSTDIASYLSFVMALFMAFGVSFEVPVAIVLLCWMGVTTPEDLRKKRPYVLVGAFVVGMLLTPPDVFSQTLLAIPMYFLFEVGVFFSRFYVGKRQTRDEDNEAESEAEAEKAEKTEE>UPI00049F13FB YfcL family protein OS=Citrobacter amalonaticus OX=35703 GN=ILP74_05175 SS=EMBLWGS:MBE0394869 PC=UP000642697:Unassembled WGS sequenceMIAEFESRILALIDDMVEHASDDELFASGYLRGHLTLAVAELEGTDDHSAQAVYANVTRSLENAIGAGELSPRDQALVKEMWDALYQKVAHQGV>UPI00049F1438 Flavodoxin OS=Citrobacter amalonaticus OX=35703 GN=ILP74_02805 SS=EMBLWGS:MBE0394433 PC=UP000642697:Unassembled WGS sequenceMAEIGIFVGTMYGNALLVAEEAEAILTAQGHNATVFEDPELTDWQKYQDKIALVVTSTTGQGDLPDSIVPLFQGIKDQLGFQPNLRYGVIALGDSSYVNFCNGGKQFDALLQEQSAQRVGEMLLIDASEHPEPESESNPWVEHWGTLLS>UPI00049F1443 RnfH family protein OS=Citrobacter amalonaticus OX=35703 GN=ILP74_03505 SS=EMBLWGS:MBE0394564 PC=UP000642697:Unassembled WGS sequenceMSPKIVVEVAYALPEKQYLQPVTLQPGATVEEAIRASGLLELRTDIDLTKNKVGIYSRPVKLADVLQDGDRVEIYRPLIADPKELRRQRAEKSAKK>UPI00049F1463 Methylaspartate mutase subunit S OS=Citrobacter amalonaticus OX=35703 GN=ILP74_14405 SS=EMBLWGS:MBE0396603 PC=UP000642697:Unassembled WGS sequenceMKKSTLVIGVIGADCHAVGNKVLDRVFTAHDFRVINLGVMVSQDEYIDAAIETGADAIVVSSIYGHGDIDCLGLRERCIERGIGDILLYVGGNLVVGKHDFADVEAKFKEMGFQRVFAPSHDLEDVCQLMAADINKRHGIEKRCLEEAI>UPI00049F152F DNA polymerase III subunit beta OS=Citrobacter amalonaticus OX=35703 GN=dnaN SS=EMBLWGS:MBE0398071 PC=UP000642697:Unassembled WGS sequenceMKFTVEREHLLKPLQQVSGPLGGRPTLPILGNLLLQVADGALSLTGTDLEMEMVARVALIQPHEPGATTVPARKFFDICRGLPEGAEIAVQLEGDRMLVRSGRSRFSLSTLPAADFPNLDDWQSEVEFTLPQATMKRLIEATQFSMAHQDVRYYLNGMLFETEGEELRTVATDGHRLAVCSMPVGQSLPNHSVIVPRKGVIELMRMLDGGDNPLRVQIGSNNIRAHVGDFIFTSKLVDGRFPDYRRVLPKNPDKHLEAGCDILKQAFARAAILSNEKFRGVRLYVSENQLKITANNPEQEEAEEILDVTYPGTEMEIGFNVSYVLDVLNALKCENVRIMLTDSVSSVQIEDAASQSAAYVVMPMRL>UPI00049F1537 Isoprenoid biosynthesis glyoxalase ElbB OS=Citrobacter amalonaticus OX=35703 GN=elbB SS=EMBLWGS:MBE0397576 PC=UP000642697:Unassembled WGS sequenceMKKIGVVLSGCGVYDGAEIHETVLTLLSIARSGAQAVCFAPDKAQADVINHLTGEPMAETRNVLIEAARITRGNIRPLSQAVSADLDALIVPGGFGAAKNLSNFASQGSECRVDPDLAGLALAMHQSGKPLGFMCIAPAMLPKIFDFPLRLTIGTDIDTAEVLEEMGAEHVPCPVDDIVVDEDNKIVTTPAYMLAQDIAQAAMGIEKLVSRVLVLAE>UPI00049F1623 HAMP domain-containing protein OS=Citrobacter amalonaticus OX=35703 GN=ILP74_01305 SS=EMBLWGS:MBE0394159 PC=UP000642697:Unassembled WGS sequenceMPKSLSLRNTLLVLLSLITLLLLLTGGMGIYASTRIITSWIYYGVMTATTLTAIALLAVVWLLLRNKLLKPLDNVVEQLERLATGDLSAAESRYASAEFNRLQAALEGMRMALSESVRRVRDASSQIDTGSRELTAGNIHLATRTESTATSLEQTAASMEELTATVKQNAENADQAHQLAKSVSDTADRGSEMVCYVIEKMRDISGSANRIADILGVIDGIAFQTNILALNASVEAARAGEQGRGFAVVAGEVRNLASRSAEAAKEIRTLIGDSQSQVGEGSDLAMQAGETMDEIASEVMRMTKLMREIASASQEQSRGIEQVNIAVSQMDETAQQNAALVQQSSAATRSLEEQSHALIEAMASFKLQTA>UPI00049F162C YdcH family protein OS=Citrobacter amalonaticus OX=35703 GN=ILP74_10230 SS=EMBLWGS:MBE0395823 PC=UP000642697:Unassembled WGS sequenceMFPEYRDLISRLKSENPRFLSLFEKHNNLDHEIARLEGSDGRGYNLEVVRLKKQKLQLKDDMLKILQKESVDAG>UPI00049F16AA Macrodomain Ori organization protein MaoP OS=Citrobacter amalonaticus OX=35703 GN=maoP SS=EMBLWGS:MBE0398117 PC=UP000642697:Unassembled WGS sequenceMAESFTTTNRYFDNKYYPRGFSRHGDFTIKEAQLLERHGYAFNELDLGKREPVTEEEKLFVAVCRGEREPVTEAERVWSKYMTRIKRPKRFHTLSGGKPQMEGAEDYTESDD>UPI00049F16C9 As(III)-sensing metalloregulatory transcriptional repressor ArsR OS=Citrobacter amalonaticus OX=35703 GN=arsR SS=EMBLWGS:MBE0394312 PC=UP000642697:Unassembled WGS sequenceMPQLSSLQLFKNLSDETRLGIVLLLREMGELCVCDLCTALEQSQPKISRHLAMLRESGLLLDRKQGKWVHYRLSPHIPSWAAQVIEQAWLSQQDDVQAIARKLASANCSGSGKAVCI>UPI00049F1730 Glutamine ABC transporter permease GlnP OS=Citrobacter amalonaticus OX=35703 GN=glnP SS=EMBLWGS:MBE0396523 PC=UP000642697:Unassembled WGS sequenceMQFDWSAIWPAIPILLQGAKMTLWISILGLAGGIIIGLLAGLARTYGGWIANHVSLVFIEVIRGTPIVVQVMYIYFALPMAFPDLRIDTFSAAVITIMINSGAYIAEITRGSVLSIHKGFSEAGLALGLSRRETIRHVIMPLALRRMLPSLGNQLIISIKDTSLFIVIGAAELTRSGQEIIAGNFRALEIWTAVGVIYLIITQVLNIVLRSLERRMKIL>UPI00049F17CF LysR family transcriptional regulator OS=Citrobacter amalonaticus OX=35703 GN=ILP74_02305 SS=EMBLWGS:MBE0394338 PC=UP000642697:Unassembled WGS sequenceMKDINFDFKQLQAFLAVIETGSFTAAAKKLNLTQSSISQQIANLENGLKTEVINRSQRPIQMTIAGQALYPLGKKIIDSGVYLQEHINAISHGHISHLKIGFVDSIGKSIGLDILTFLQPQVKHIFQVTGTASGLLSALNTGSINLAITMLHTEMPPNVRMYPLIEEEFLCVCPKAWPETRLEELCKNRDYIAYTRNTPTGIQTLNWLKWNNLSPSIQFEMDNADDILKLISCGYGWTLTTPLFITTLPAFTDSLKIIRINNRKERRKIVLLCKDDELSEFYKNLAIEVQSILETKLEQGFYSKLNADVPS>UPI00049F181C Hemolysin III family protein OS=Citrobacter amalonaticus OX=35703 GN=ILP74_01710 SS=EMBLWGS:MBE0394234 PC=UP000642697:Unassembled WGS sequenceMVQKPLIAQGYSLAEEIANSISHGIGLVFGIVGLVLLLVQAVDMNASATAITSYSLYGGSMILLFLASTLYHAIPHQRAKIWLKKFDHCAIYLLIAGTYTPFLLVGLNSPLARGLMIVIWSLALLGILFKLTIAHRFKVLSLVTYLTMGWLSLIVVYQLAIKLAVGGVTLLAVGGVVYSLGVIFYVSKRIPYNHAIWHGFVLGGSVCHFLAIYLYVGQA>UPI00049F1834 Sodium:alanine symporter family protein OS=Citrobacter amalonaticus OX=35703 GN=ILP74_17725 SS=EMBLWGS:MBE0397214 PC=UP000642697:Unassembled WGS sequenceMPDFFTFINDVLWGSVMIYLLFGAGCWFTWRTGFVQFRYIRQVGRSLKNSINPQPGGLTSFQSLCTGLAARIGSGNLAGVALAITAGGPGAVFWMWIAAIIGMATSFAECALAQLYKERDRHGQFRGGPAWYMARGLGMRWMGVLFAIFLLIAYGLVFNSVQANSVSRALKFAFDFPPVATGIVMAIAALLVIVRGIKGVARMMQWFVPVMALMWVVTSLVVCLINIDQLPDIIISIVKSAFGWQEAAGGVAGYTLSQAITSGFQRSMFSNEAGMGSTPNAAAAAASWPPHPAAQGIVQMIGIFIDTLIVCSATAMLVLLAGNDATYAPMEGIQLVQKAMNTLVGEWGAAFVAVIVILFAFSSIVVNYIYAENNLYFLKLDNKRFIWALRITSCSTVVVGTLLSFPLLWQLADIIMACMAITNLTAILLLSPVVRTLASDYLRQRKLGVRPVFDPLRYPDIGQQLAPDAWDDIPRD>UPI00049F1887 Murein tripeptide/oligopeptide ABC transporter ATP binding protein OppF OS=Citrobacter amalonaticus OX=35703 GN=oppF SS=EMBLWGS:MBE0395691 PC=UP000642697:Unassembled WGS sequenceMNAVDEKRKVLLEIADLKVHFDIKDGKQWFWQPSKTLKAVDGVTLRLYEGETLGVVGESGCGKSTFARAIIGLVKATDGRVAWLGKDLLGMNPDEWRDVRSDIQMIFQDPLASLNPRMTIGEIIAEPLRTYHPKLPRQEVRDRVKAMMMKVGLLPNLINRYPHEFSGGQCQRIGIARALILEPKLIICDEPVSALDVSIQAQVVNLLQQLQREMGLSLIFIAHDLAVVKHISDRVLVMYLGHAVELGTYDEVYHNPLHPYTKALMSAVPIPDPDLEKNKTIQLLEGELPSPINPPSGCVFRTRCPIAGPECAKTRPVLEGSFRHAVSCLKVDPL>UPI00049F1895 Efflux MFS transporter YdeE OS=Citrobacter amalonaticus OX=35703 GN=ydeE SS=EMBLWGS:MBE0395930 PC=UP000642697:Unassembled WGS sequenceMNKTLRRSTLALLASSLLLTIGRGATLPFMTIYLSRQYDLSVDLIGYAMTIALTIGVVFSLGFGILADKFDKKRYMLLAISAFAFGFIAIPLVDSVMLVVLLFALINCAYSVFATVLKAWFADHLSPGAKAKIFSLNYTVLNIGWTVGPPLGTLLVMQSINLPFWLAAFCSAFPLVFIQFLVKRSAAAVNSENSVAWSPAVLLHDKALFWFTLSAFLASFVSGAFASCLSQYVMVVADGNFAEKVVAVVLPVNAAVVVSLQYAVGRRLTPANIRPLMGFGTVCFVIGLGGFMISGNSLLLWGVSAAIFTLGEVIYAPGEYMLIDNIAPPGMKASYFSAQSLGWLGAAFNPLASGVILTSFPAWTLFIVLMVAIIVAWALMLKGMRVRPWGQPALC>UPI00049F18C1 HlyC/CorC family transporter OS=Citrobacter amalonaticus OX=35703 GN=ILP74_00935 SS=EMBLWGS:MBE0394088 PC=UP000642697:Unassembled WGS sequenceMLNSIFIILCLIAVSAFFSISEISLAASRKIKLKLLADEGNLNAQRVLKMQENPGMFFTVVQIGLNAVAILGGIVGDAAFSPAFNSLFSRYMSPELSEQLSFILSFSLVTGMFILFADLTPKRIGMIAPEAVALRIINPMRFCLFVFTPLVWFFNGLANVIFRIFKLPMVRKDDITSDDIYAVVEAGALAGVLRKQEHELIENVFELESRTVPSSMTSRENVIWFDLHEDEQSLKNKVAEHPHSKFLVCNEDIDHIIGYVDSKDLLNRVLANQSMALNSGVQIRNTLIVPDTLTLSEALESFKTAGEDFAVIMNEYALVVGIITLNDVMTTLMGDLVGQGLEEQIVARDENSWLIDGGTPIDDVMRVLDIDEFPQSGNYETIGGFMMFMLRKIPKRTDSVKFSGYKFEVVDIDNYRIDQLLVTRLDSKPSVLVPKLPDVQGDVKA>UPI00049F1A3F Peptidoglycan glycosyltransferase/peptidoglycan DD-transpeptidase MrcA OS=Citrobacter amalonaticus OX=35703 GN=mrcA SS=EMBLWGS:MBE0397746 PC=UP000642697:Unassembled WGS sequenceMKFVKYLLILAVCCILLGAGSIYGLYRYIEPQLPDVATLKDVRLQIPMQVYSADGELIAQYGEKRRIPVTLNQIPPEMVKAFIATEDSRFYEHHGVDPVGIFRAASVALFSGHASQGASTITQQLARNFFLSPERTLMRKIKEVFLAIRIEQLLSKDEILELYLNKIYLGYRAYGVGAAAQVYFGKSVDQLDLGEIAVIAGLPKAPSTFNPLYSMDRATARRNVVLSRMLSEGYITQAQYDQARNEPIDANYHAPEIAFSAPYLSEMVRQEMYNRYGENAYEDGYRIYTTITRKVQQAAQQAVRNNVLDYDMRHGYRGPANVLWKVGETAWDSKKITDTLKALPTYGPLLPAVITSANPQEATATLADGTSVSLRMEGIRWARPYRSDTQQGPTPRKVTDAVQTGQQIWVRKMGEAWWLAQVPDVNSALVSINPKNGAVLALVGGFDFNQSKFNRATQALRQVGSNIKPFLYTAAMDKGLTLASMLNDVPISRWDAGAGSDWRPKNSPPQYAGPIRLRQGLGESKNVVMVRAMRAMGVDYAAEYLQRFGFPAQNIVRTESLALGSASFTPLQVARGYAVMSNGGFLVDPYFISKIETDQNGVIFEAKPKIACPECDIPVIYGDTQKSNVLENSNVEDVSISGEQQNAAVPMPKLEQANQALVAQTGAQEYAPHVINTPLAFLIKSALNTNIFGEPGWMGTGWRAGRDLNRHDIGGKTGTTNSSKDAWFSGYGPGVVTSVWIGFDDHRRDLGRTTASGAIKDQISGYEGGAKSAQPAWDAYMKVVLEGVPEEPLTPPPGIITVNIDRSTGQLANGGNSRAEYFIEGTQPTQQAVHEVGTTITDGGETHELF>UPI00049F1A96 Protein-serine/threonine phosphatase OS=Citrobacter amalonaticus OX=35703 GN=pphA SS=EMBLWGS:MBE0395560 PC=UP000642697:Unassembled WGS sequenceMKQPEKSYQMIEGAQWRHIWVVGDIHGCFALLMAKLRACRFDPWQDLLVSVGDLIDRGPDSLRCLQLLHKRWMVAVRGNHEQMAIDALRTSQWSLWTMNGGGWFTALPEQKRHHATLALEYCQHLPWILELHCREATHVVAHADYPEDIYQWQKPVNLTEVLWRRARLSAHLAGEGTAIAGADHFWFGHTPLRQRVDSDNLHYIDTGAVFGGELTLVQLQ>UPI00049F1AAE CidA/LrgA family protein OS=Citrobacter amalonaticus OX=35703 GN=ILP74_00135 SS=EMBLWGS:MBE0393939 PC=UP000642697:Unassembled WGS sequenceMAVAISRVTPAVLQRLQVPVQVLLYAGLFIVAQYLVSWLHLPLPANLVGMVLMLALIVCRILPLSWVRAGARWLLAEMLLFFVPAVVAVVNYAHLLLVDGWRIFSVIAISTLMVLGATAWVVDKVYRYEISRLNRD>UPI00049F1B08 MipA/OmpV family protein OS=Citrobacter amalonaticus OX=35703 GN=ILP74_11920 SS=EMBLWGS:MBE0396140 PC=UP000642697:Unassembled WGS sequenceMTKLKLLALGVLIASSATVVHAESNLTLGAGVGVVEHPYKDYDADVYPVPVINYEGDNFWFRGLGGGYYLWNDAADKLSITAYWSPMYFKPGDSDDRQMRRLDKRKSTMMAGLSYSHHTEYGFLRTTLAGDTLDNSNGIVWDLAWLYRYTNGGLTLTPGIGVEWNSENQNDYYYGVSRKESSRSGMRGYNPDDSWNPYLELSANYNFLGDWSVYGTARYTRLSDEITDSPMVDKSWTGLISTGITYKF>UPI00049F1B3C Serine O-acetyltransferase OS=Citrobacter amalonaticus OX=35703 GN=cysE SS=EMBLWGS:MBE0397939 PC=UP000642697:Unassembled WGS sequenceMPCEELDIVWNNIKTEARALADCEPMLASFYHATLLKHENLGSALSYMLANKLASPIMPAIAIREVVEEAYAADPEMIASAACDIQAVRTRDPAVDKYSTPLLYLKGFHALQAYRIGHWLWNEGRRALAIFLQNQVSVTFQVDIHPAAKIGRGIMLDHATGIVVGETAVIEDDVSILQSVTLGGTGKTSGDRHPKIREGVMIGAGAKILGNIEVGRGAKIGAGSVVLQPVPPHTTAAGVPARIVGKPDSDKPSMDMDQHFNGIHHTFEYGDGI>UPI00049F1BE0 DUF2474 domain-containing protein OS=Citrobacter amalonaticus OX=35703 GN=ILP74_10320 SS=EMBLWGS:MBE0395841 PC=UP000642697:Unassembled WGS sequenceMQHSVWKRLMWLVILWGGSVLALAAVGMFFRLIMTAAGFKSH>UPI00049F1D64 Primosomal replication protein N OS=Citrobacter amalonaticus OX=35703 GN=priB SS=EMBLWGS:MBE0394072 PC=UP000642697:Unassembled WGS sequenceMTNRLVLSGTVCRTPLRKVSPSGIPHCQFVLEHRSVQEEAGFHRQAWCQMPVIVSGHENQAITHSITVGSAVTVQGFISCHKAKNGLSKMVLHAEQIELIDSGD>UPI00049F1D69 Fluoride efflux transporter CrcB OS=Citrobacter amalonaticus OX=35703 GN=crcB SS=EMBLWGS:MBE0396701 PC=UP000642697:Unassembled WGS sequenceMLQLLLAVFIGGGTGSVARWMLSMRFNPMHQAIPIGTLTANLLGAFIIGVGLAWFSRMTNIDPVWKVLITTGFCGGLTTFSTFSAEVVFLLQEGRVGWAMLNVLVNLLGSFAMTALAFWLFSASSAH>UPI00049F1DCB Cytochrome c biogenesis heme-transporting ATPase CcmA OS=Citrobacter amalonaticus OX=35703 GN=ccmA SS=EMBLWGS:MBE0395012 PC=UP000642697:Unassembled WGS sequenceMLEARKLLCERDERVLFSDLSFQVNAGEWIQVTGGNGAGKTTLLRLLTGLSRPDAGEVCWQEQPLHRVRDSYHQNLLWIGHQPGIKTRLTALENLRFFHQDGDIAKCLAALAQAGLAGYEDIPVNQLSAGQQRRVALARLWLTRATLWILDEPFTAIDVNGVERLTQRMAQHTDEGGIVILTTHQPMNVATDRIRRIALTHERAGQ>UPI00049F1DCE Cell division protein FtsP OS=Citrobacter amalonaticus OX=35703 GN=ftsP SS=EMBLWGS:MBE0397398 PC=UP000642697:Unassembled WGS sequenceMSLSRRQFIQASGIALCAGAVPLRAHAAGQQQPLPVPPLLESRRGQPLFMTLQRSHWSFTQGTRAPVWGINGRYLGPTIRVWKGDDVKLIYSNRLAENVSMTIAGLQVPGPLMGGPARMMSPNADWAPVLPIRQNAATLWYHANTPNRTAQQVYNGLAGMWLVEDEVSKSLPIPNHYGVDDFPIIIQDKRLDNFGTPEYSEPGSGGFVGDTLLVNGVQSPYVEVSRGWVRLRLLNASNSRRYQLQMSDGRALHVISGDQGFLPAPVSVKQLSLAPGERREILVDMTNGDEVSITCGEAASIVDRIRGFFEPSSILVSTLVLTLRPTGLLPLVTDSLPMRLLPTEIMSGTPIRSRDISLGDDPGINGQLWDVNRIDITAQQGSWERWTVRADMPQSFHIEGVSFLVRNVNGAMPFPEDRGWKDTVWVDGQVELLVYYGQPSWAHFPFYFHSQTLEMADRGSIGQILVNPAA>UPI00049F1E79 Carnitine operon protein CaiE OS=Citrobacter amalonaticus OX=35703 GN=caiE SS=EMBLWGS:MBE0397182 PC=UP000642697:Unassembled WGS sequenceMSFYAFEGLIPVVHPEAYVHPSAVLIGDVIVGAGVYVGPLASLRGDYGRLILEAGSNLQDGCIMHGYCDTDTVVRENGHIGHGAILHGCVIGRDALVGMNSVIMDGAVIGDESIVAAMSFVKAGFTGAARQLLVGTPARVMREVSEQELHWKRLNTKEYQDLAVRCRASLRETSPLTAVEANRPRLKGTTEVKPKSAM>UPI00049F1EE9 PTS glucitol/sorbitol transporter subunit IIC OS=Citrobacter amalonaticus OX=35703 GN=srlA SS=EMBLWGS:MBE0394508 PC=UP000642697:Unassembled WGS sequenceMIETITHGAEWFIGLFQKGGEVFTGMVTGILPLLISLLVIMNALINFIGQQRIERFAQRCAGNPLSRYLILPFIGTFVFCNPMTLSLGRFMPEKYKPSYYAAASYSCHSMNGLFPHINPGELFVYLGIASGLTTLGLPLGPLAVSYLLVGVVTNFFRGWVTDLTTAIFEKKMGIQLEQKVHLAGAAS>UPI00049F1FC0 23S rRNA pseudouridine(2604) synthase RluF OS=Citrobacter amalonaticus OX=35703 GN=rluF SS=EMBLWGS:MBE0398428 PC=UP000642697:Unassembled WGS sequenceMLPDSSTRLNKYISESGICSRREADRFIEQGNVFINGKRATIGDQVMPGDVVKVNGRLIEPREAEDLVFIALNKPVGIVSTTEDSERDNIVDFVNHSKRIFPIGRLDKDSQGLIFLTNHGDLVNKILRAGNDHEKEYLVTVDKPVTDEFIRGMSAGVPILGTVTKKCKVKKEAPFVFRITLIQGLNRQIRRMCEHFGYEVVKLERTRIMNVGLSGLPLGEWRDLTDDELITLFKLIENSSSEAKPKAKAKPKTAGIKRPVVKIEKTANKEKARPAANGKRFTSPGRKKKGR>UPI00049F1FFE ABC transporter permease OS=Citrobacter amalonaticus OX=35703 GN=ILP74_23825 SS=EMBLWGS:MBE0398366 PC=UP000642697:Unassembled WGS sequenceMANVLSLRFPRSVEGWLGWVIVLMLAFFSLMSNEFLSIQNLLDLTESYAVTGIFALGLFVVLVTGGIDISFAAVASVVQYVVASLLLSGTMTSPLLCLGFAIALGVIFGLINAILIYYLNVVSIIITISMQSLLFGMLMWLTNGHSIYDLPDWWITQRSLFSFGFEGETYQVGLPLVVMLAMACFTWMLMNKTHIGRQLYAVGGSTESARRIGIRVSLIYLFAYGYLGAAAAIGGMLQAYRMSEVVPNALVGGELDVLAAAVLGGASLSGGRGSVIGTLMGVFLIGILKNGLNLIGVSSYFVNIVIGVVIVSAICVTHYKKRKETDVGFV>UPI00049F2010 Endonuclease/exonuclease/phosphatase family protein OS=Citrobacter amalonaticus OX=35703 GN=ILP74_14065 SS=EMBLWGS:MBE0396543 PC=UP000642697:Unassembled WGS sequenceMTQSTRNFSFTVLTINTHKGFTAFNKRFILPELRDAVRTVGADIVCLQEVMGAHEVHPLHVENWPDTTHYEFLADTMWSDYAYGRNAVYPQGHHGNAVLSRYPIEHYENRDVSVGSSEKRGVLYCRIVPPQLGKPIHVMSVHLGLREAHRQAQLTLLTDWVNALPDGEPVVVAGDFNDWRQKANHSLKAAGLEEIFTRARGRPARTFPVSLPLLRLDRIYVKNANASSPTALPLRNWRHLSDHAPLSAEIHL>UPI00049F205C YdbL family protein OS=Citrobacter amalonaticus OX=35703 GN=ILP74_10115 SS=EMBLWGS:MBE0395801 PC=UP000642697:Unassembled WGS sequenceMKRTFILAALILSLLSGSAFALTLNEARTQGRVGETFNGYLVALKQDAQTLALVSEINKARSASYQQLAESHNIPVDEVAKMAGQKLVERAKPGEYVQGINGKWLRK>UPI00049F211F Glutathione transferase GstA OS=Citrobacter amalonaticus OX=35703 GN=gstA SS=EMBLWGS:MBE0395991 PC=UP000642697:Unassembled WGS sequenceMKLFYKPGACSLASHITLRESGKDFTLDGVDLMKKRLENGDDFFAVNPKGQVPALLLDDGTLLTEGVAIMQYLADSVPDRQLLAPVSSISRYKTLEWLNYIATELHKGFTPLFRPDTPEEYKPTVRALLEKKMQYVDAALKEGQWICGSRFTIADAYLFTVLRWAYAVKLNMDGLENIAAYMKRVAERPGVAAALKAEGIQ>UPI00049F2134 DedA family protein OS=Citrobacter amalonaticus OX=35703 GN=ILP74_03250 SS=EMBLWGS:MBE0394516 PC=UP000642697:Unassembled WGS sequenceMSDGLSLLSLFASSFLSATLLPGNSEVVLIAMLLAGLSHPWVLVLTATMGNSLGGLTNVILGRFFPLRKTSRWQEKATGWLKRYGAVTLLLSWMPVVGDLLCLLAGWMRLSWGPVLFFLCLGKALRYIVVAAATVQGITWWH>UPI00049F21CD L-ribulose-5-phosphate 4-epimerase OS=Citrobacter amalonaticus OX=35703 GN=araD SS=EMBLWGS:MBE0398362 PC=UP000642697:Unassembled WGS sequenceMNTLKQHVLEANLSLPAHKLVTFTWGNVSGIDREKGIMVIKPSGVEYNHMGLEDMVVVDVQSGNVIEGNKKPSSDTDTHLALYRAFPTIGGIVHTHSRHATIWSQAGKDLMALGTTHADYFYGTIPCTRPMHDDEINGRYEYETGSVIIETFAQRGLSPEQIPAVLVNSHGPFAWGSDPANAVHNAVVLEEIAYMNLFTQQLQPQIPAMQPTLLDKHFLRKHGANAYYGQ>UPI00049F2207 Beta-hydroxyacyl-ACP dehydratase OS=Citrobacter amalonaticus OX=35703 GN=ILP74_20970 SS=EMBLWGS:MBE0397827 PC=UP000642697:Unassembled WGS sequenceMIPHEIERHQAQPQQVEIVLHLDPSLFWFSGHFAVQPLLPGVAQMDWVMHYATTLLAPGWRFHSIQNVKFQAPLLPENTVTLTLSWQEARQILTFSYQRHDGDARHTASSGKIRLCR>UPI00049F2261 Cytidine deaminase OS=Citrobacter amalonaticus OX=35703 GN=cdd SS=EMBLWGS:MBE0395119 PC=UP000642697:Unassembled WGS sequenceMHPRFHTAFSQLADNLQSALAPILADTHFPASLTAEQVSMLKSATGLDEDALAFALLPLAAACACTPLSNFNVGAIARGVSGTWYFGANMEFLGATMQQTVHAEQSAISHAWLRGEKGLAAITVNYTPCGHCRQFMNELNSGLDLRIHLPGREPHTLRDYLPDAFGPKDLDIKTLLMDEQDHGFAPQGDALTQAAIAAASRSHMPYSQSPSGVALECKDGRIFSGSYGENAAFNPTIPPLQGALIMLNLNGYGYADIQRAVLAEKADAPLIQWDATAATLKALGCSNIDRVLLG>UPI00049F22C6 TRNA (Guanosine(18)-2'-O)-methyltransferase TrmH OS=Citrobacter amalonaticus OX=35703 GN=trmH SS=EMBLWGS:MBE0397981 PC=UP000642697:Unassembled WGS sequenceMNLQRYARICEMLARRQPDLTVCMEQVHKPHNVSAIIRTADAVGVHEVHAVWPGSRMRTMASAAAGSNSWVQVKTHRTIGDAVTHLKGRGMQVLATHLSDNAVDFREIDYTRPTCILMGQEKTGITQEALDLADQDIIIPMIGMVQSLNVSVASALILYEAQRQRQNAGMYLRENSMLPEGEQQRLLFEGGYPVLAKVAKRKGLPYPHINQQGAIEADDAWWSTMQAAK>UPI00049F22E0 5-amino-6-(5-phospho-D-ribitylamino)uracil phosphatase YigB OS=Citrobacter amalonaticus OX=35703 GN=yigB SS=EMBLWGS:MBE0398165 PC=UP000642697:Unassembled WGS sequenceMRFYRSPGPISAITFDLDDTLYDNRPVIQRTEQEALAFVQNYHPALRTLQNTDLQRLRQAVREAEPEIYHDVTRWRHRAVERAMLNAGLSAQEAVAGANAAMMNFAKWRSQVDVPQETHDTLKALARKWPLVAITNGNAQPELFGLGDYFEFVLRAGPDGRSKPFSDMYFLAAEKLTVPIGEILHVGDDLTTDVAGAIRSGLQACWIKPENADLMHTFDSRLLPHIEISQLASLTSLI>UPI00049F22E2 DNA polymerase III subunit delta OS=Citrobacter amalonaticus OX=35703 GN=holA SS=EMBLWGS:MBE0396686 PC=UP000642697:Unassembled WGS sequenceMIRLYPEQLRAQLSEGLRAAYLLLGNDPLLLQEGQDAIRQVAATQGFDEHHTFTLDNNTDWNALFSQCQAMSLFASRQTLLLLLPENGPNAAINEQLATLVKLLHDDLLLIVRGNKLSKAQENAAWFKALTDRSVQISCQTPEQAQLPRWVAARAKQNNLQLDDAANQLLCYCYEGNLLALAQALERLSLLWPDGKLTLPRVEQAVNDAAHFTPFHWVDALIMGKSKRALHILQQLRLEGSEPVILLRTLQRELLLLVTLKRQSAHTPLRTLFDKHRVWQNRRGMIGDALNRLSPAQLRQAVQLLTRTEITLKQDYGQSVWAELEGLSLLICHKALAEVFIDG>UPI00049F23A7 UbiD family decarboxylase OS=Citrobacter amalonaticus OX=35703 GN=ILP74_24565 SS=EMBLWGS:MBE0398481 PC=UP000642697:Unassembled WGS sequenceMAFDDLRSFLQALDDQGQLLKISEEVNAEPDLAAAANATGRIGDGAPALWFDNIRGFTDARVAMNTIGSWQNHAISLGLPPNTPVKKQIDEFIRRWDKFPVTPERRANPAWAENSVDGEAINLFDILPLFRLNDGDGGFYLDKACVVSRDPLDPDNFGKQNVGIYRMEVKGKRKLGLQPVPMHDIALHLHKAEERGEDLPIAITLGNDPIITLMGATPLKYDQSEYEMAGALRESPYPIATAPLTGFDVPWGSEVILEGVIESRKREIEGPFGEFTGHYSGGRNMTVVRIDKVSYRSKPIFESLYLGMPWTEIDYLMGPATCVPLYQQLKAEFPEVQAVNAMYTHGLLAIISTKKRYGGFARAVGLRAMTTPHGLGYVKMVIMVDEDVDPFNLPQVMWALSSKVNPAGDLVQLPNMSVLELDPGSSPAGITDKLIIDATTPVAPDNRGHYSQPVCDLPETKAWAEKLTAMLANRK>UPI00049F240B Acyl-CoA thioester hydrolase YciA OS=Citrobacter amalonaticus OX=35703 GN=yciA SS=EMBLWGS:MBE0395697 PC=UP000642697:Unassembled WGS sequenceMTTTTHAPQGELVLRTLAMPADTNANGDIFGGWLMSQMDIGGAIQAKEIAHGRVVTVRVEGMSFLRPVAVGDVVCCYARCVKRGTTSVSINIEVWVKKVASEPIGQRYKATEALFIYVAVDPDGKPRPLPVE>UPI00049F2474 Na/Pi cotransporter family protein OS=Citrobacter amalonaticus OX=35703 GN=ILP74_24150 SS=EMBLWGS:MBE0398416 PC=UP000642697:Unassembled WGS sequenceMLTLLHLLSAVALLVWGTHIVRTGVMRVFGARLRTVLSRSVEKKPLAFCAGIGVTALVQSSNATTMLVTSFVAQDLVALTPALVIVLGADVGTALMARILTFDLSWLSPLLIFIGVIFFLGRKQSRAGQLGRVGIGLGLILLALELIVQAVTPITQANGVQVIFASLTGDIMLDALIGAMFAIISYSSLAAVLLTATLTAAGIISFPVALCLVIGANLGSGLLAMLNNSAANAAARRVALGSLLFKLVGSLIILPFVHPLANLMDELPLAKSELVIYFHVFYNLVRCLAMIPFAEPMARFCKRIIRDEPELDAHLKPKHLDVSALDTPTLALANAAREALRIGDAMEQMMEGLKKVMHGEPREEKELRKIADDINVLYTAIKLYLARMPKEELAEEESRRWAEIIEMSLNLEQASDIVERMGSEIADKSLAARRAFSIEGLKELDALYDQLLSNLQLAMSVFFSGDVTSARRLRRSKHRFRILNRRYSHAHVDRLHQQNVQSIETSSLHLGLLGDMQRLNSLFCSVAYSVLEQPDEDDERDDY>UPI00049F248E D-serine/D-alanine/glycine transporter OS=Citrobacter amalonaticus OX=35703 GN=cycA SS=EMBLWGS:MBE0394078 PC=UP000642697:Unassembled WGS sequenceMVDQVKVAADEQAPTEQSLRRNLTNRHIQLIAIGGAIGTGLFMGSGKTISLAGPSIIFVYMIIGFMLFFVMRAMGELLLSNLEYKSFSDFASDLLGPWAGYFTGWTYWFCWVVTGMADVVAITAYAQFWFPGLSDWVASLAVVILLLSLNLATVKMFGEMEFWFAMIKIVAIVSLIIVGLVMIAMHFQSPTGVEASFAHLWNDGGWFPKGISGFFAGFQIAVFAFVGIELVGTTAAETKDPEKSLPRAINSIPIRIIMFYVFALIVIMSVTPWSSVVPDKSPFVELFVLVGLPAAASVINFVVLTSAASSANSGVFSTSRMLFGLAQEGVAPKAFAKLSKRAVPAKGLTFSCICLLGGVVMLYVNPSVIGAFTMITTVSAILFMFVWTIILCSYLVYRKQRPHLHEKSIYKMPLGKLMCWVCMAFFVFVLVLLTLEDDTRQALLVTPLWFIALGLGWWLIGKKRMAGVR>UPI00049F2556 SPFH/Band 7/PHB domain protein OS=Citrobacter amalonaticus OX=35703 GN=ILP74_15490 SS=EMBLWGS:MBE0396805 PC=UP000642697:Unassembled WGS sequenceMLIVIPILIFVALVIVGAGVKIVPQGYQWTVERFGRYTKTLQPGLSIVVPFMDRIGRKINMMEQVLDIPSQEVISKDNANVTIDAVCFIQVIDAPRAAYEVSNLELAIINLTMTNIRTVLGSMELDEMLSQRDSINTRLLHIVDEATNPWGIKVTRIEIRDVRPPAELISSMNAQMKAERTKRAYILEAEGVRQAEILKAEGEKQSQILKAEGERQSAFLQAEARERSAEAEARATKMVSEAIASGDIQAVNYFVAQKYTEALQQIGSSSNSKVVMMPLDASSLMGSIAGITELIKDSASERNK>UPI00049F2583 1-acyl-sn-glycerol-3-phosphate acyltransferase OS=Citrobacter amalonaticus OX=35703 GN=ILP74_20945 SS=EMBLWGS:MBE0397822 PC=UP000642697:Unassembled WGS sequenceMKGVISTLNRLWRIAMTGFCFALFGLGGLLLSVVWFNVLLVFVWDNARRRRIARRSIAASFRLFLTVTRVLGVLDYQMNGVDILRQERGCLVVANHPTLIDYVLLASVMPETDCLVKSALLKNPFLSGVVRAADYLVNSQADALLPASQQRLAQGDTILIFPEGTRTRPGETMTLQRGAANIAVRCGSDIRVVTIHCSQRMLDKESKWYQVPPVKPLFTVEVRERVKIDHFYDANLQEPALAARQLNRHLLLQLQPGTLPLSGINDASALS>UPI00049F25F0 Lytic murein transglycosylase B OS=Citrobacter amalonaticus OX=35703 GN=mltB SS=EMBLWGS:MBE0394509 PC=UP000642697:Unassembled WGS sequenceMFKRRYVALLPLFVLLAACSSKPKPQEPQTTTGTPSGGFLLEPQHNVMQMGGDFANNPNAQQFIDKMVNKHGFNRQQLQEILSQAKRLDYVLRLMDRQAPTTQPPAGPNGAWLRYRKQFITPDNVQNGVAFWNQYEDALNRAWQVYGVPPEIIVGIIGVETRWGRVMGKTRILDALATLSFNYPRRAEYFSGELETFLLMARNEQDDPLDLKGSFAGAMGYGQFMPSSYKEYAVDFNGDGHINLWDPVDAIGSVANYFKAHGWVKGDAVAVMANGQAPGLANGFKTQYSLSQLAAAGLTPQQPLGNHQQVSLLRLDIGTGYQYWYGLPNFYAITRYNHSTHYAMAVWQLGQAVALARVQ>UPI00049F265C Galactarate dehydratase OS=Citrobacter amalonaticus OX=35703 GN=garD SS=EMBLWGS:MBE0397495 PC=UP000642697:Unassembled WGS sequenceMANIDIRQESPSAFYIKVHETDNVAIIVNDNGLKAGTRFPDGLELIEHIPQGHKVALVDIPVHGEIIRYGEVIGYAVRDIPRGSWIDESMVELPTAPPLNTLPLATKVPEPLPPLEGYTFEGYRNADGSVGTKNLLGITTSVHCVAGVVDYVVKIIERDLLPKYPNVDGVVGLNHLYGCGVAINAPAAVVPIRTIHNISLNPNFGGEVMVIGLGCEKLQPERLLEGTDDVQSIPVDSASIVSLQDEKHVGFRSMVDDILQVAERHLVKLNLRQRETCPASELVVGMQCGGSDAFSGVTANPAVGYASDLLVRCGATVMFSEVTEVRDAIHLLTPRTINEAVGKRLLEEMAWYDNYLDMGKTDRSANPSPGNKKGGLANVVEKALGSIAKSGKSAIVEVLSPGQRPTKRGLIYAATPASDFVCGTQQVASGITVQVFTTGRGTPYGLMAVPVIKMATRTELAKRWYDLMDINAGTIATGEETIEEVGQKLFEFILDVASGRKKTFSDQWGLHNQLAVFNPAPVT>UPI00049F2670 Aminoacyl-tRNA hydrolase OS=Citrobacter amalonaticus OX=35703 GN=pth SS=EMBLWGS:MBE0395644 PC=UP000642697:Unassembled WGS sequenceMTIKLIVGLANPGAEYAATRHNAGAWYVDLLAERLRAPLREEPKFFGYTSRVSLEGEDVRLLVPTTFMNLSGKAVGAMASFYRINPDEILVAHDELDLPPGVAKFKLGGGHGGHNGLKDIISKLGNNPNFHRLRVGIGHPGDKNKVVGFVLGKPPVSEQKLIDDAIDEAARCTEVWFKDGLTKATNRLHAFKAQ>UPI00049F267D Class II fumarate hydratase OS=Citrobacter amalonaticus OX=35703 GN=fumC SS=EMBLWGS:MBE0395973 PC=UP000642697:Unassembled WGS sequenceMVTVRSEKDSMGAIDVPADKLWGAQTQRSLEHFRISTEKMPVSLIHALALTKRAAAKVNDDLGLLAADKAAAIMQAADEVLADKHAEEFPLAIWQTGSGTQSNMNMNEVLANRASELLGGVRGMERKVHPNDDVNKSQSSNDVFPTAMHVAALLALRNHLIPQLQVLLKTLNEKSDAFADIVKIGRTHLQDATPLTLGQEISGWVAMLEHNLRHIENSLPHVAELALGGTAVGTGLNTHPEYARRVADELAAITQAPFVTAPNKFEALATCDALVHAHGALKGLAASLMKIANDVRWLASGPRCGIGEIAIPENEPGSSIMPGKVNPTQCEALTMLCCQVMGNDVAINIGGASGNFELNVFRPMVIHNFLQSVRLLADGMESFNEHCAVGIEPNRERINQLLNESLMLVTALNTHIGYDKAAEIAKKAHKEGLTLKAAALALGYLTEVEFDSWVRPEQMVGSMKPGR>UPI00049F2944 EAL domain-containing protein OS=Citrobacter amalonaticus OX=35703 GN=ILP74_11585 SS=EMBLWGS:MBE0396075 PC=UP000642697:Unassembled WGS sequenceMNVSLDNVYHSELYFQPARNVQRKLIGLSVIANFVSEDGDVRIPTELVLPRLSPEEQCQLFIEKLALIETCQHFFIQHKLVAWIYITPAVVPLLLTNSECVSAVKRFSFLELMINENFPELSEGKENKTLHALAERFPLVLANFGAGESANKAIFDGLFKRIILDRNFVHRRATRLSFEPFMRAILTQVSPYCESLMIAGIDSEAMLTRVTPFGFSGMQGGLWPAVSASQVTQLLHG>UPI00049F297A GMP reductase OS=Citrobacter amalonaticus OX=35703 GN=ILP74_17220 SS=EMBLWGS:MBE0397118 PC=UP000642697:Unassembled WGS sequenceMRIEEDLKLGFKDVLIRPKRSTLKSRSDVELERQFTFKHSGQTWSGVPIIAANMDTVGTFAMATALASFDVLTAVHKHYTVEDWTAFVSSASADVLKHVMVSTGTSDADFEKTKQILALDPALNFVCIDVANGYSEHFVQFVSKAREAWPTKTICAGNVVTGEMCEELVLSGADIVKVGIGPGSVCTTRVKTGVGYPQLSAVIECADAAHGLGGMIVSDGGCTMPGDVAKAFGGGADFVMLGGMLAGHEESGGKIVEENGEKFMLFYGMSSESAMTRHVGGVAQYRAAEGKTVKLPLRGPVENTARDILGGLRSACTYVGASRLKELTKRTTFIRVQEQENRVFNSL>UPI00049F29AA Rho-binding antiterminator OS=Citrobacter amalonaticus OX=35703 GN=rof SS=EMBLWGS:MBE0397038 PC=UP000642697:Unassembled WGS sequenceMSMNDTYQPINCDDYDNLELACQHHLLLTLELKDGEVLQAKANDLVSRKNVEYLITEVAGETRELRLDKIVSFSHPEIGTVVVSES>UPI00049F2A9C Protein bax OS=Citrobacter amalonaticus OX=35703 GN=ILP74_21420 SS=EMBLWGS:MBE0397914 PC=UP000642697:Unassembled WGS sequenceMILTPIRRYGAMILMLLTIVFSGEVLAKTHTTTSSQKSHVTKTSNKQVSSKQEYSRNSAKSSSLPDLRKYPSGTPRKKAFLRTVMPYITSQNAAITADRNWLISKQYQNRWSPTERKRMKDIAKRYKVKWSGNTRSIPWNTLLERVDIIPTSMVATMAAAESGWGTSKLARSNNNLFGMKCAKGSCNNAPGKVKGYSQFDSVKESVSAYVINLNTHPAYASFRKSRAQLRKADQEVTATAMIHKLKGYSTKGQSYNNYLFAMYQDNQRLIAAHM>UPI00049F2AED 50S ribosomal protein L1 OS=Citrobacter amalonaticus OX=35703 GN=rplA SS=EMBLWGS:MBE0398382 PC=UP000642697:Unassembled WGS sequenceMAKLTKRMRVIREKVDATKQYDINEAISLLKELATAKFVESVDVAVNLGIDARKSDQNVRGATVLPHGTGRSVRVAVFTQGANAEAAKAAGAELVGMEDLAEQIKKGEMNFDVVIASPDAMRVVGQLGQVLGPRGLMPNPKVGTVTPNVAEAVKNAKAGQVRYRNDKNGIIHTTIGKVDFDADKLKENLEALLVALKKAKPTQAKGVYIKKISISTTMGAGVAVDQAGLSASAN>UPI00049F2B69 Acetate uptake transporter OS=Citrobacter amalonaticus OX=35703 GN=satP SS=EMBLWGS:MBE0397210 PC=UP000642697:Unassembled WGS sequenceMGNTKLANPAPLGLMGFGMTTILLNLHNAGFFALDGIILAMGIFYGGIAQIFAGLLEYKKGNTFGLTAFTSYGSFWLTLVAILLMPKMGLTEAPNAQFLGAYLGLWGVFTLFMFFGTLTGARMLQFVFLSLTVLFALLAVGNIAGNEAVIHFAGWVGLVCGASAIYLAMGEVLNEQFGRTILPIGEKH>UPI00049F2B9B Iron-sulfur cluster insertion protein ErpA OS=Citrobacter amalonaticus OX=35703 GN=erpA SS=EMBLWGS:MBE0397068 PC=UP000642697:Unassembled WGS sequenceMSDDVALPLQFTEAAANKVKSLIADEENPNLKLRVYITGGGCSGFQYGFTFDDQVNEGDMTIEKQGVGLVVDPMSLQYLVGGSVDYTEGLEGSRFVVTNPNAKSTCGCGSSFSI>UPI00049F2CDA ABC transporter permease OS=Citrobacter amalonaticus OX=35703 GN=ILP74_10360 SS=EMBLWGS:MBE0395848 PC=UP000642697:Unassembled WGS sequenceMHSERAPFFLKLAAWGGVVFLHFPILIIAAYAFNSEDAAFSFPPQGLTLRWFSVAAQRSDILDAVTLSLKIAALSTAIALVLGTLAAAALWRRDFFGKNAISLLLLLPIALPGIVTGLALLTAFKTVNLDPGFFTIVVGHATFCVVVVFNNVIARFRRTSWSLVEASMDLGANGWQTFRYVVLPNLGSALLAGGMLAFALSFDEIIVTTFTAGHERTLPLWLLNQLGRPRDVPVTNVVALLVMLVTTIPILGAWWLTREGDSVAGNGQ>UPI00049F2D47 Multiple antibiotic resistance transcriptional regulator MarR OS=Citrobacter amalonaticus OX=35703 GN=marR SS=EMBLWGS:MBE0395925 PC=UP000642697:Unassembled WGS sequenceMKSTSDLFNEIIPLGRLIHMVNQKKDRLLNDYLSPLDITAAQFKVLCSIRCAGCITPVELKKVLSVDLGALTRMLERLVCKGWIDRLPNPHDKRGVLVKLTEHGAAICEQCHQLVGQDLHQELTKNLTADEVATLEHLLKKVLP>UPI00049F2D74 Holliday junction resolvase RuvX OS=Citrobacter amalonaticus OX=35703 GN=ruvX SS=EMBLWGS:MBE0397260 PC=UP000642697:Unassembled WGS sequenceMSETLLAFDFGTKSIGVAIGQRVTGTARALPAIKAQDGTPDWNLIERLLKEWQPDEIIVGLPLNMDGTEQPLTARARKFANRIHGRFGVTVTLHDERLSTVEARSGLFEQGGYRALNKGKIDSASAVIILESYFEQGY>UPI00049F2DB7 CidA/LrgA family protein OS=Citrobacter amalonaticus OX=35703 GN=ILP74_06470 SS=EMBLWGS:MBE0395121 PC=UP000642697:Unassembled WGS sequenceMSKTLNIIWQYLRAFVLIYACLYAGIFIASLLPVAISGSIIGMLILFVLLALQIIPAKWVNPGCYVLIRYMALLFVPIGVGVMQYFDLLRAQFGPVVVSCAISTLVVFLVVSWSSHLVHGERKVVGQEGSKE>UPI00049F2DD5 Asparaginase OS=Citrobacter amalonaticus OX=35703 GN=ansA SS=EMBLWGS:MBE0396130 PC=UP000642697:Unassembled WGS sequenceMQKKSIYVAYTGGTIGMQRSEQGYIPVSGHLQRQLALMPEFHRPEMPDFTIHEYAPLMDSSDMTPEDWQHIADDIKTHYDEYDGFVILHGTDTMAFTASALSFMLENLGKPVIVTGSQIPLAELRSDGQINLLNALYVAANYPINEVTLFFNNRLFRGNRTTKAHADGFDAFASPNLQPLLEAGIHIRRLGTPLAPRSEGELIVHPITPQPIGVVTIYPGISADVVRNFLRQPVKALILRSYGVGNAPQNKEFLKELSEASERGIVVVNLTQCMSGKVNMGGYATGNALAHAGVVGGADMTVEATLTKLHYLLSKGLDTDAIREAMTQNLRGELTPDD>UPI00049F2E43 Ribosome modulation factor OS=Citrobacter amalonaticus OX=35703 GN=rmf SS=EMBLWGS:MBE0396351 PC=UP000642697:Unassembled WGS sequenceMKRQKRDRLERAHQRGYQAGIAGRSKEMCPYQTLNQRSYWLGGWREAMADRVVMA>UPI00049F2E87 Replicative DNA helicase OS=Citrobacter amalonaticus OX=35703 GN=dnaB SS=EMBLWGS:MBE0398472|EMBLWGS:MBE0393924 PC=UP000642697:Unassembled WGS sequenceMAGNKPFNKQQTDVRDRDPQVAGLKVPPHSIEAEQSVLGGLMLDNERWDDVAERVVAEDFYTRPHRHIFTEMGRLQETGSPIDLITLAESLERQGQLDSVGGFAYLAELSKNTPSAANISAYADIVRERAVVRDMIAVAHEIADAGFDPQGRTSEDLLDLAESRVFKIAESRANKDEGPKNIADVLDATVARIEQLFQQPHDGVTGVNTGYDDLNKKTAGLQPSDLIIVAARPSMGKTTFAMNLVENAAMLQDKPVLIFSLEMPSEQIMMRSLASLSRVDQTRIRTGQLDDEDWARISGTMGILLEKRNIYIDDSSGLTPTEVRSRARRIAREHGGIGLIMIDYLQLMRVPSLSDNRTLEIAEISRSLKALAKELHVPVVALSQLNRSLEQRADKRPVNSDLRESGSIEQDADLIMFIYRDEVYHENSDLKGIAEIIIGKQRNGPIGTVRLTFNGQWSRFDNYAGPQYDDE>UPI00049F2F49 Acyl-CoA thioesterase II OS=Citrobacter amalonaticus OX=35703 GN=tesB SS=EMBLWGS:MBE0396849 PC=UP000642697:Unassembled WGS sequenceMSQALNNLLTLLNLEKIEEGLFRGQSEDLGLRQVFGGQVVGQALYAAKETVPEERLVHSFHSYFLRPGDSQRPIIYDVEVLRDGNSFSARRVAAIQNGKPIFYMTASFQAPEPGFEHQKTMPHAPAPEGLTSETDIARSVAHQLPPILKDKFLCDRPLEVRPVEFHNPLKGHVAEPTRQVWIRANGTLPNDIRVHQYLLGYASDLNFLPVALQPHGIGFLEKGIQIATIDHSMWFHRPFDLNEWLLYSVESTSASSARGFVRGEFYTQDGKLVASTVQEGVMRNHNE>UPI00049F2F9E VOC family protein OS=Citrobacter amalonaticus OX=35703 GN=ILP74_12845 SS=EMBLWGS:MBE0396312 PC=UP000642697:Unassembled WGS sequenceMFSYIMLGTNDLPRAIGFYDPLMELLGHPKAGRGEEGASWGMFSENHTTGLCVGKPFDGQAAGVGNGTMVALNARSVEHIQQLHALALRLGGADEGAPGHRPQYGQGFHSAYVRDPDGNKLAFVYYADEC>UPI00049F2FAE DUF418 family protein OS=Citrobacter amalonaticus OX=35703 GN=ILP74_06425 SS=EMBLWGS:MBE0395112 PC=UP000642697:Unassembled WGS sequenceMERNVTLDFVRGVAILGILLLNISAFGLPKAAYLNPAWYGNITASDAWTWAILDLFAQVKFLTLFALLFGAGLQMLLPRGKRWIQSRLTLLVLLGFIHALLFWDGDILLAYGLVGLICWRLVRDAPSVKSLFNTGVMLYLVGIAVLLLLGAISGSETNRAWTPDASALLYEQYWKVNGGVEAISNRVDALSNSLLALGAQYGWQLAGMMLLGAALMRSGWLKGQFRLSHYRRSGFLLIAVGLLINLPAVVVQWQLEWSYRWCAFLLQAPRELSAPFQTLGYTALMFGYWPQLSRSKIAHAIACVGRMALTNYLLQTVICTTLFYQFGLFMKFDRLELLLFVIPVWLANLLFSVIWLRFLPQGPMEWLWRQLTLRASGTSLSHTSR>UPI00049F30CC GTPase HflX OS=Citrobacter amalonaticus OX=35703 GN=hflX SS=EMBLWGS:MBE0394057 PC=UP000642697:Unassembled WGS sequenceMFDRYDAGEQAVLVHIYFSQDKDMEDLQEFESLVSSAGVEAMQVITGSRKAPHPKYFVGEGKAVEIAEAVKATGAAVVLFDHALSPAQERNLERLCECRVIDRTGLILDIFAQRARTHEGKLQVELAQLRHLATRLVRGWTHLERQKGGIGLRGPGETQLETDRRLLRNRILQIQSRLEKVEKQREQGRQSRIKADVPTVSLVGYTNAGKSTLFNQITEARVYAADQLFATLDPTLRRIDVADVGETVLADTVGFIRHLPHDLVAAFKATLQETRQATLLLHVVDAADVRVQENIDAVDTVLEEIDAHEIPSLMVMNKIDMLDDFEPRIDRDEENKPIRVWLSAQTGVGIPQLFQALTERLSGEVAQHTLRLPPQEGRLRSRFYQLQAIEKEWMEDDGSVSLQVRLPIVDWRRLCKQEPALEDYVV>UPI00049F3183 L-carnitine/gamma-butyrobetaine antiport BCCT transporter OS=Citrobacter amalonaticus OX=35703 GN=caiT SS=EMBLWGS:MBE0397177 PC=UP000642697:Unassembled WGS sequenceMKNEKRKSGIEPKVFFPPLIIVGILCWLTVRDLDAANVVINAVFSYVTNVWGWAFEWYMVIMLIGWFWLVFGPYAKKKLGDEKPEFSTASWIFMMFASCTSAAVLFWGSIEIYYYISTPPFGLEPNSTGAKEIGLAYSLFHWGPLPWATYSFLSVAFAYFFFVRKMDVIRPSSTLVPLVGEKHAKGLLGTIIDNFYLVALIFAMGTSLGLATPLVTECMQYLFGIPHTLQLDAIIITCWIILNAICVACGLQKGVRIASDVRSYLSFLMLGWVFIVSGASFIMNYFTDSVGTLLMYLPRMLFYTDPIAKGGFPQGWTVFYWAWWVIYAIQMSIFLARISRGRTVRELCFGMVLGLTASTWILWTVLGSNTLLLMDKNIINIPQLIADHGVPRAIIETWAALPLSTATMWGFFILCFIATVTLINACSYTLAMSTCREVRDGEEPPLLVRIGWSVLVGVIGIVLLALGGLKPIQTAIIAGGCPLFFVNIMVTLSFIKDAKVHWKDK>UPI00049F31B4 DNA topoisomerase IV subunit B OS=Citrobacter amalonaticus OX=35703 GN=parE SS=EMBLWGS:MBE0397407 PC=UP000642697:Unassembled WGS sequenceMTQTYNADAIEVLTGLEPVRRRPGMYTDTTRPNHLGQEVIDNSVDEALAGHAKRVDVILHADQSLEVIDDGRGMPVDIHPEEGVPAVELILCRLHAGGKFSNKNYQFSGGLHGVGISVVNALSKRVEVNVRRDGQVYNIAFENGDKVQDLQVVGTCGKRNTGTSVHFWPDETFFDSPRFSVSRLTHVLKAKAVLCPGVEITFKDEVNNSEQRWCYQDGLNDYLREAVNGLPTLPEKPFIGNFSGDTETVDWALLWLPEGGELLTESYVNLIPTMQGGTHVNGLRQGLLDAMREFCEYRNILPRGVKLSAEDIWDRCAYVLSVKMQDPQFAGQTKERLSSRQCAAFVSGVVKDAFSLWLNQNVQSAEQLAEMVISSAQRRLRAAKKVVRKKLTSGPALPGKLADCTAQDLNRTELFLVEGDSAGGSAKQARDREYQAIMPLKGKILNTWEVSSDEVLASQEVHDISVAIGIDPDSDDLSQLRYGKICILADADSDGLHIATLLCALFVKHFRTLVKHGHVYVALPPLYRIDLGKEVYYALTEEEKAGVLEQLKRKKGKPNVQRFKGLGEMNPMQLRETTLDPNTRRLVQLVIDDEDDQRTNAMMDMLLAKKRSEDRRNWLQEKGDLADIDG>UPI00049F323E Inorganic phosphate transporter PitA OS=Citrobacter amalonaticus OX=35703 GN=pitA SS=EMBLWGS:MBE0397848 PC=UP000642697:Unassembled WGS sequenceMLHLFAGLDLHTGLLLLLALAFVLFYEAINGFHDTANAVATVIYTRAMRSQLAVVMAAVFNFFGVLLGGLSVAYAIVHMLPTDLLLNMGSSHGLAMVFSMLLAAIIWNLGTWYFGLPASSSHTLIGAIIGIGLTNALMTGTSVVDALNIPKVIGIFASLIVSPIVGLVVAGGLIFLLRRYWSGTKKRARIHLTPAEREKKDGKKKPPFWTRIALILSAIGVAFSHGANDGQKGIGLVMLVLIGVAPAGFVVNMNASGYEITRTRDAINNVEIYFQQHPDLLKQVTGVDQLIPSPEPAATQPAEFHCHPANTLNALDRAKAMLSNNVESYDKLSVEQRGQLRRIMLCISDTTDKVTKLPGVSADDQRLLKKLKTDMLSTIEYAPIWIIMAVALALGIGTMIGWRRVATTIGEKIGKKGMTYAQGMSAQMTAAVSIGLASYTGMPVSTTHVLSSSVAGTMVVDGGGLQRKTVTSILMAWVFTLPAAILLSGTLYWISLKLI>UPI00049F3306 Gnt-II system L-idonate transporter OS=Citrobacter amalonaticus OX=35703 GN=idnT SS=EMBLWGS:MBE0394106 PC=UP000642697:Unassembled WGS sequenceMPLIIIAAGVALLLVLMIGFKVNGFIALVLVAAVVGFAEGMDAQAVLHSIQNGIGGTLGGLAMILGFGAMLGKLISDTGAAQRIATTLIGTFGKKRVQWALVVTGLVVGLAMFFEVGFVLLLPLVFTVVASSGLPLLYVGVPMVAALSVTHCFLPPHPGPTAIATIFEASLGTTLLYGFIITIPTVIVAGPLFSKLLTRFEKAPPEGLFNPHLFTEEEMPSFWNSIFAAVIPVILMAVAAVCEITLPKTNSVRVFFEFIGNPAVALFIAIVLAIFTLGRRNGRTIEQIMDIVGDSIGAIAMIVFIIAGGGAFKQVLVDSGVGQYISHLMTGTSLSPLLMCWTVAALLRIALGSATVAAITTAGVVLPIINVTHADPALMVLATGAGSVIASHVNDPGFWLFKGYFNLSVGETLRTWTVMETLISIMGLLGVLALNAVLH>UPI00049F3385 Glycerophosphodiester phosphodiesterase OS=Citrobacter amalonaticus OX=35703 GN=glpQ SS=EMBLWGS:MBE0394976 PC=UP000642697:Unassembled WGS sequenceMKKTLKNLSLTLMMAGAVMGSHALAASNTDKVVIAHRGASGYLPEHTLPAKAMAYAQGADYLEQDLVMTKDDQLVVLHDHYLDRVTDVAERFPDRARKDGRYYAIDFTLDEIKSLKFTEGFDLENGKKVQTYPGRFPMGKSDFRVHTFAEEIEFVQGLNHSTGKNIGIYPEIKAPWFHHQEGKDIATKTLEVLKQYGYSDKQDKVYLQSFDVAELKRIKNELEPKMGMDLNLVQLIAYTDWNETQEKQPDGSWVNYNYDWMFKPGAMKQVAEYADGIGPDYHMLIDEKSTKGNIVLTDMVKEAHQNKMAVHPYTVRADQLPEYTTDVNQLYDILYNKAGVDGLFTDFPDKAVMFLRKE>UPI00049F33B1 Protein YohO OS=Citrobacter amalonaticus OX=35703 GN=ILP74_06570 SS=EMBLWGS:MBE0395139 PC=UP000642697:Unassembled WGS sequenceMSVAKIGVITLFLLMAIGGIGGVMLAGYTFILRAG>UPI00049F341E NUDIX hydrolase YfcD OS=Citrobacter amalonaticus OX=35703 GN=yfcD SS=EMBLWGS:MBE0394921 PC=UP000642697:Unassembled WGS sequenceMVEQRRLASTEWVDIVNEDNEVIAQSSREQMRAQGLRHRATYIVVHDGMGKILVQRRTETKDFLPGMLDATAGGVVQADEQLLDSARREAEEELGIAGVPFAEHGQFYFEDKNCRVWGALFSCVSHGPFALQEEEVSEVCWLTPEEITARCDEFTPDSLKALALWMTRNAKNEAARSESQQEKQEEAE>UPI00049F34D0 DUF202 domain-containing protein OS=Citrobacter amalonaticus OX=35703 GN=ILP74_22150 SS=EMBLWGS:MBE0398056 PC=UP000642697:Unassembled WGS sequenceMKISRLGEAPDYRFSLANERTFLAWIRTALGFLAAGVGLDQLAPDFATPVIRELLALLLCLFAGGLAIYGYLRWLRNEKAMRLKEDLPYTRSLLIISLILTIVAIVVMALVLYAG>UPI00049F354B Sugar ABC transporter permease OS=Citrobacter amalonaticus OX=35703 GN=ILP74_09835 SS=EMBLWGS:MBE0395748 PC=UP000642697:Unassembled WGS sequenceMARLFSGRSDMPFAMLLLAPSLILLGGLVAWPMLSNIEISFLRLPLNPRINATFVGVANYVRILSDPDFWHSLWMTVWYTALVVTGSTVLGLGVAMFFNREFRLRKTARSLVILSYVTPSISLVFAWKYMFNSGYGIVNYLGVDLLHLYDQAPLWFDNPGSSFVLVVLFAIWRYFPYAFISFLAILQTIDKSLYEAAEMDGANAWQRFRIVTLPAIMPVLATVVTLRTIWMFYMFADVYLLTTKVDILGVYLYKTAFAFNDLGKAAAISVVLFIIIFAVILLTRNRVNLNGNK>UPI00049F3605 Metal-binding protein ZinT OS=Citrobacter amalonaticus OX=35703 GN=zinT SS=EMBLWGS:MBE0395610 PC=UP000642697:Unassembled WGS sequenceMAIHWKKLSVSLGMLWFSSYAFAHGDHAHGVPLTEMEQKAAEGEFADSNVKDRALTDWDGMWQSVYPYLVSGELDPVFKQKAAKDKGKTAEEVKAYYRKGYATEVDTIGIENGVMEFHTGKQVASCQYDYAGYKILTYTSGKKGVRYLFECKDANSKAPKFVQFSDHIIAPRASNHFHIFMGNTSQQALLEEMDNWPTYYPYQLKAQAVVDEMLHH>UPI00049F364D PTS fructose-like transporter subunit IIB OS=Citrobacter amalonaticus OX=35703 GN=ILP74_23765 SS=EMBLWGS:MBE0398354 PC=UP000642697:Unassembled WGS sequenceMSVSTGRFLVAVTACVSGVAHTYMAAERLEKLCQQEKWTIKIETQGALGTENRLTEEDIRRADAVLLITDIELAGAERFTQCRYVQSGINAFLREPQRVMSAVRKLLSAPQYTHLILD>UPI00049F36DC Fimbrial usher protein StbD OS=Citrobacter amalonaticus OX=35703 GN=stbD SS=EMBLWGS:MBE0394367 PC=UP000642697:Unassembled WGS sequenceMKLSSFFPLLLVGVVLAQPAWSACKRVTSANDLSQTAKDAGYIGASWGGVGDSEVKGKLGLPGVITLSSGTGFQTEGTLLASSTASFVPNGRTQGVNANQIYFRCDVAEIGKVYEYYATNGDDAWGGREAVAGIEGAYYTYVKNVALRLTNLKTGQYYSRYWQTRLIPESEMFNDGTYIYIPGSAFSDVFVELFRVDDASKGVNGSNRYGYTYAGPAGYIAFHGGGMSSGLFDGADSRTNYDGWGAMQWPGGWTLTSQSYFVRGAACRIDDYPAIVRLPPISVGELSGGGTAQTPFNITVECETGAISGTAVSTTSKANVAMGFLVNNQTAANAANQLGLKTGSGAWTWLLDNHYGVSGVASGVGIRIYSEKLGGSAINLLPNLTSTATGNAGGWYGYADLTSKTSTSGSTELYNGEFTASLEAIPGEAITAGSVYAQLQVVVSFQ>UPI00049F3703 Spermidine N1-acetyltransferase OS=Citrobacter amalonaticus OX=35703 GN=speG SS=EMBLWGS:MBE0395949 PC=UP000642697:Unassembled WGS sequenceMTSALSVKLRPLEREDLRFVHQLDNNASVMRYWFEEPYEAFVELSDLYDKHIHDQSERRFVVECDGEKAGLVELVEINHVHRRAEFQIIISPEYQGKGLASRAAKLAMDYGFTVLNLYKLYLIVDKENEKAIHIYRKLGFMVEGELIHEFFINGEYRNTIRMCIFQHQYLAEHRSPGSTMLKPTAQ>UPI00049F378C Amino acid permease OS=Citrobacter amalonaticus OX=35703 GN=ILP74_22685 SS=EMBLWGS:MBE0398154 PC=UP000642697:Unassembled WGS sequenceMAENKPELQRGLEARHIELIALGGTIGVGLFMGAASTLKWAGPSVLLAYIVAGLFVFFIMRSMGEMLFLEPVAGSFAVYAHRYMSPFFGYLTAWSYWFMWMAVGISEITAIGVYVQFWFPEMAQWIPALIAVGLVALANLAAVRLYGEIEFWFAMIKVTTIIVMIVVGLGVIFFGLGNGGQPIGFGNLTGHGGFFAGGWKGFLTALCIVVASYQGVELIGITAGEAKNPQVTLRSAVGKVLWRILIFYVGAIFVIVTIFPWNEIGSNGSPFVLTFAKIGITTAAAIINFVVLTAALSGCNSGMYSCGRMLYALAKNRQLPAAMGKVSRHGVPVAGVALSIAILLVGSCLNYIIPNPQRVFVYVYSASVLPGMVPWFVILISQLRFRRAHKAAMASHPFRSILFPWANYLTMAFLVCVLIGMYFNEDTRMSLFVGIIFLLSVTAVYKVFGLNRQGIAQKTGE>UPI00049F389D Galactose/proton symporter OS=Citrobacter amalonaticus OX=35703 GN=galP SS=EMBLWGS:MBE0397254 PC=UP000642697:Unassembled WGS sequenceMPDNKKQGRSNKAMTFFVCFLAALAGLLFGLDIGVIAGALPFITDEFQISAHTQEWVVSSMMFGAAVGAVGSGWLSFKLGRKKSLMIGAILFVAGSLFSAAAPNVEVLILSRVLLGLAVGVASYTAPLYLSEIAPEKIRGSMISMYQLMITIGILGAYLSDTAFSYSGAWRWMLGVIIIPAILLLIGVFFLPDSPRWFAAKRRFVDAERVLLRLRDTSAEAKRELDEIRESLQVKQSGWALFKENSNFRRAVFLGVLLQVMQQFTGMNVIMYYAPKIFEMAGYTNTSEQMWGTVIVGLTNVLATFIAIGLVDRWGRKPTLTLGFLVMAAGMGILGTMMHVGIHSPSAQYFAIAMLLMFIIGFAMSAGPLIWVLCSEIQPLKGRDFGITCSTATNWIANMIVGATFLTMLQNLGNANTFWVYAGLNVLFILLTLWLVPETKHVSLEHIERNLMKGRKLREIGAHD>UPI00049F38A4 Type II toxin-antitoxin system VapC family toxin OS=Citrobacter amalonaticus OX=35703 GN=ILP74_23110 SS=EMBLWGS:MBE0398228 PC=UP000642697:Unassembled WGS sequenceMQKGPVLFDTNILIDLFSGRQEAQQVLEAYPPQNAISLVTWMEVMVGAKKYHQEYRTRVALSAFNIIGVSQEIAERSVNLRQEYGMKLPDAIILATAQIHRFALVTRNTRDFAGIPGVITPYQLQAGR>UPI00049F395D Dihydromonapterin reductase OS=Citrobacter amalonaticus OX=35703 GN=folM SS=EMBLWGS:MBE0395968 PC=UP000642697:Unassembled WGS sequenceMGTQHPLPILVTGGGRRIGLAIAWHFINQKQPVIVSYRTHYPAIDGLTKAGALCIQADFSTNEGILTFADTVKERASGLRAIVHNASAWMAEKPGTPLSDVLACMMQIHVNAPYLLNHALEGMLRGHGHAAADIIHFTDYVVEKGSDKHIAYAASKAALDNMTRSFARKLAPEVKVNAIAPSLILFNEGDDAEYRQQALNKSLMKTAPGEKEVIDLIDYLLTSCFVTGRSFAVDGGRHLR>UPI00049F3A41 DUF1249 family protein OS=Citrobacter amalonaticus OX=35703 GN=ILP74_18785 SS=EMBLWGS:MBE0397410 PC=UP000642697:Unassembled WGS sequenceMKRYTPDFPEMMRLCETNFSQLRRLLPRNDAPGETVSYQVGNAQYRITIVESTRYTTLVTIEQTAPTITYWSLPSLTVRLYHDAMVAEVCSSQQIFRFKARYDYPNKKLHQRDEKHQINQFLADWLRYCLAHGAMAIPVY>UPI00049F3B76 TRNA (Adenosine(37)-N6)-threonylcarbamoyltransferase complex ATPase subunit type 1 TsaE OS=Citrobacter amalonaticus OX=35703 GN=tsaE SS=EMBLWGS:MBE0394052 PC=UP000642697:Unassembled WGS sequenceMMNRVIPLPDEQATLDLGQRVAKACNGATVIYLYGDLGAGKTTFSRGFLQALGHSGNVKSPTYTLVEPYSLDNLMVYHFDLYRLADPEELEFMGIRDYFANDAICLVEWPQQGKGVLPDPDVEIHIDYQAQGREARIRAVSSSGDSLLARLAG>UPI00049F3BA3 C4-dicarboxylate transporter DctC OS=Citrobacter amalonaticus OX=35703 GN=dctA SS=EMBLWGS:MBE0397869 PC=UP000642697:Unassembled WGS sequenceMKISLFKSLYFQVLTAIAIGILLGHYYPELGAQMKPLGDAFVKLIKMVIAPVIFCTVVTGIAGMESMKAVGRTGAVALLYFEIVSTIALIIGLIIVNVVQPGAGMNVDPATLDAKAVAIYAEQAKDQGIVGFLMDIIPGSVIGAFASGNILQVLLFAVMFGFALHRLGSKGQLIFNVIESFSQVIFGIINMIMRLAPIGAFGAMAFTIGKYGVGTLVQLGQLIVCFYITCILFVVVVLGSIARATGFSIFKFIRYIREELLIVLGTSSSESALPRMLDKMEKLGCRKSVVGLVIPTGYSFNLDGTSIYLTMAAVFIAQATNSHMDIFHQITLLVVLLLSSKGAAGVTGSGFIVLAATISAVGHLPVAGLALILGIDRFMSEARALTNLVGNGVATVVVAKWVKELDHKKLDDVLNNRAPEGKTHEISS>UPI00049F3BEE DNA polymerase II OS=Citrobacter amalonaticus OX=35703 GN=polB SS=EMBLWGS:MBE0397159 PC=UP000642697:Unassembled WGS sequenceMAQAGFILTRHWRDTSQGTEVSFWLATDSGPLQVTLAPQESVAFIPTALTARVTSLLSSENGYRLTPLNLKDFHRQPVSGLYCRSHRQLMRLEKLLRENGVTVYEGDVRPPERYLMERFITSPVWVEGETRNGAIVNARLKPHPDYRPPLKWLSLDIETTRHGELYCIGLEGCGQRIVYMLGPENGDSSALDFQLEYVNSRPQLLEKLNEWVARYDPDLIIGWNLVQFDLRVLQKHAERYRIPLRFGRDNSELEWREHGFKNGVFFAQAKGRLIVDGIEALKSAFWNFSSFSLETVSQELLGEGKSIDNPWDRMDEIDRRFAEDKPALATYNLKDCELVTRVFHKTEIVPFLLERSTVNGLPVDRHGGSVAAFGHLYFPRMHRAGYVAPNLGEVPPHASPGGYVMDSRPGLYDSVLVLDYKSLYPSIIRTFLIDPVGLVEGMAHPDPEHSTEGFLDAWFSREKHCLPEIVTSIWQGRDDAKRHGNKPLSQALKIIMNAFYGVLGTTACRFFDPRLASSITMRGHAIMRQTKALIEAQGYDVIYGDTDSTFVWLKRAHTEEEAAKIGRALVAHVNAWWTQSLQEKNLTSALELEYETHFCRFLMPTIRGADTGSKKRYAGLIQEGENQRMVFKGLETVRTDWTPLAQQFQQELYLRIFRHEPYQDYVRETIDRLMAGELDERLVYRKRLRRPLSEYQRNVPPHVRAARLADEENLKRGRPAQYQNRGTIHYVWTLNGPEPKDYQHSPLDYEHYLTRQLQPVAEGILPFIDDNFATLLTGQLGLF>UPI00049F3D6D General stress protein OS=Citrobacter amalonaticus OX=35703 GN=ILP74_12630 SS=EMBLWGS:MBE0396270 PC=UP000642697:Unassembled WGS sequenceMANHRGGSGNFAEDRERASEAGKKGGQHSGGNFKNDPQRASEAGKKGGKSSGGSRNS>UPI00049F3DBA NAD(+) kinase OS=Citrobacter amalonaticus OX=35703 GN=nadK SS=EMBLWGS:MBE0394567 PC=UP000642697:Unassembled WGS sequenceMNNHFKCIGIVGHPRHPTALTTHEMLYRWLCTKGYEVIVEQQIAHELQLKNVKIGTLAEIGQQADLAVVVGGDGNMLGAARTLARYDIKVIGINRGNLGFLTDLDPDNAQQQLADVLEGHYVAEKRFLLEAQVCQKDCQKRISTAINEVVLHPGKVAHMIEFEVYIDESFAFSQRSDGLIISTPTGSTAYSLSAGGPILTPSLDAITLVPMFPHTLSARPLVINSSSTIRLRFSHRRNDLEISCDSQIALPIQEGEDVLIRRCDYHLNLIHPKDYSYFNTLSTKLGWSKKLF>UPI00049F3EEF Nickel-responsive transcriptional regulator NikR OS=Citrobacter amalonaticus OX=35703 GN=nikR SS=EMBLWGS:MBE0397843 PC=UP000642697:Unassembled WGS sequenceMQRVTITLDDDLLETLDSLSQRRGYNNRSEAIRDILRGALAQETTQEHGTQGFAVLSYVYEHEKRDLASRIVSTQHHHHDLSVATLHVHINHDDCLEIAVLKGDMGDVQHFADDVIAQRGVRHGHLQCLPKEE>UPI00049F3F4D Protoheme IX farnesyltransferase OS=Citrobacter amalonaticus OX=35703 GN=cyoE SS=EMBLWGS:MBE0396873 PC=UP000642697:Unassembled WGS sequenceMMFKQYLQVTKPGIIFGNLISVIGGFLLASKGSIDYPLFIYTLVGVSLVVASGCVFNNYIDRDIDRKMERTKNRVLVRGLISPKVSLVYATLLGIAGFMLLWFGANPLACWLGVMGFVVYVGVYSLYMKRHSVYGTLIGSLSGAAPPVIGYCAVTGEFDSGALILLAIFSLWQMPHSYAIAIFRFKDYQAANIPVLPVVKGISVAKNHITLYIIAFAIATLMLSLGGYAGYKYLVVAAAVSVWWLGMALRGYKVEDDRVWARKLFGFSIIAITALSVMMSVDFMVPDSHNLLTYVW>UPI00049F3F72 Glycine cleavage system protein GcvH OS=Citrobacter amalonaticus OX=35703 GN=gcvH SS=EMBLWGS:MBE0394228 PC=UP000642697:Unassembled WGS sequenceMSNVPAELKYSKEHEWLRKEADGSYTVGITEHAQELLGDMVFVDLPEVGTTVSAGDDCAVAESVKAASDIYAPIGGEIVAVNDALSDSPELVNSEPYADGWIFKIKASDESELDSLLDATAYEALLEDE>UPI00049F3FAC Urease accessory protein UreG OS=Citrobacter amalonaticus OX=35703 GN=ureG SS=EMBLWGS:MBE0397439 PC=UP000642697:Unassembled WGS sequenceMSEYKHPLRVGVGGPVGSGKTALLEALCKAMRDRWQLAVVTNDIYTKEDQRILTEAGALAPERIVGVETGGCPHTAIREDASMNLAAVEALSEKFGNLDLIFVESGGDNLSATFSPELADLTIYVIDVAEGEKIPRKGGPGITKSDFLVINKTDLAPYVGASLEVMERDTLRMRGERPWTFTNLKSGDGLATIIAFLEDRGMLRV>UPI00049F4034 Cobyrinate a,c-diamide synthase OS=Citrobacter amalonaticus OX=35703 GN=ILP74_07355 SS=EMBLWGS:MBE0395290 PC=UP000642697:Unassembled WGS sequenceMAAKQYAFVLAGTGSGCGKTTVTLGLLNVMKQRGLRVQPCKVGPDYLDTAWHTAISGTASRNLDSFMLPEPVLNALFREQMQDADIAVIEGVMGLYDGYGTDPDYCSTAAMAKQLGCPVILLVDGKAVSTSIAATVMGFQHFDPTLNIAGVIVNRVNSESHFQLLKTAIEHYCAVPVLGYVPRVDGVALPERHLGLVTARESVVNQQSWQDFAARLESTLDIDRLLALSHLQALPPGEWPERPAPNAGEGLTLAMADDEAFNFYYPDNVALLARTGVNIVRFSPLHDRELPDCQMVWLGGGYPELHASALAANTTMLTSLREAHQRGVAIYAECGGLMYLGSLLEDADGVEHRMADILPGRSKMGKRLTRFGYCEAQALQPTLLAAEGDVLRGHEFHYSDFSPETPAVLACRKVRDGQTVQAWSGGWRTGNTFASYLHVHFAQRPLMLNHWLNAAREAL>UPI00049F40BD Thiol:disulfide interchange protein DsbE OS=Citrobacter amalonaticus OX=35703 GN=dsbE SS=EMBLWGS:MBE0395018 PC=UP000642697:Unassembled WGS sequenceMKRNVLLIPFVIFLVIAAALLWQLARNAEGDTPTNLESALIGKPVPTFRLESLENPGKHYEADVLTQGKPVLLNVWATWCPTCRAEHQYLNQLSAQGIRVVGLNYKDDRQKAIVWLKELGNPYALSLFDGDGMLGLDLGVYGAPETFLIDGKGIIRYRHAGDLNARVWESEIKPLWEKYSKEAGQ>UPI00049F40D5 Transcriptional regulator OS=Citrobacter amalonaticus OX=35703 GN=ILP74_09085 SS=EMBLWGS:MBE0395606 PC=UP000642697:Unassembled WGS sequenceMPKNDENSLLSQLDTIAKGLSETFSPFCEVVVHDLKDPEHAIMSIHNNLSGREAGQPATELGLARIASPDFPEIIANYGNQFADGRPVKSTSIGIKDEKGNYVAALCLNVDMTLFRGMQSALARFTETESSPVREHLDPGSTEVIRQRIDDFAAKRATTARALKTEDRKVLIQQLRKEGLLNVRKSMDTVAQHLGVSRATAYLYARQSG>UPI00049F4203 Nramp family divalent metal transporter OS=Citrobacter amalonaticus OX=35703 GN=ILP74_04610 SS=EMBLWGS:MBE0394767 PC=UP000642697:Unassembled WGS sequenceMTNDRVESSSGRAARKLRLTLMGPAFVAAIGYIDPGNFATNIQAGASFGYKLLWVVVWANLMAMLIQVLSAKLGIATGKNLAEQIRDHYPRPVVWFYWVQAEIIAMATDLAEFIGAAIGFKLILGVSLLQGAVLTGIATFLILMLQRRGQKPLEKVIGGLLLFVAMAYIVELIFSQPNFAQLSKGMIIPDLPNSEAVFLAAGVLGATIMPHVIYLHSSLTQHLHGGTRQQRYAATKWDVAIAMTIAGFVNLAMMATAAAAFHFSGHTGVADLDQAYLTLEPLLSHAAATVFGLSLVAAGLSSTVVGTLAGQVVMQGFVRFHIPLWVRRTITMMPSFIVILIGLDPTRILVMSQVLLSFGIALALVPLLIFTSDSKLMGDLVNTRWVKQTGWMIVVLVVALNLWLLVGTVLGL>UPI00049F42B0 GFA family protein OS=Citrobacter amalonaticus OX=35703 GN=ILP74_10125 SS=EMBLWGS:MBE0395803 PC=UP000642697:Unassembled WGS sequenceMTEKRNAQCHCGAVKFTVQLSDGFNTIRRCNCSFCRMRGAVVVSAPLMGITVLSGQDKLTEYRFNTGTARHFFCSVCGIYTFHQRRSSPDQYGVNVACIENVSPFDFACVEVTDGVNHPSDGASNGVVGYLRYQAK>UPI00049F42C6 EnvZ/OmpR regulon moderator MzrA OS=Citrobacter amalonaticus OX=35703 GN=mzrA SS=EMBLWGS:MBE0397471 PC=UP000642697:Unassembled WGS sequenceMLKPRITLKQLAWSTAFLLTLSAMLLVWSTVRQQESTLAIRAVHQGASMPDGFSIWHHLDANGIRFKSITPKNDTLLITFDSSAQSAAAKAVLDKTLPRGYIIAQQDDNNQAVQWLSRLRDTPHRVG>UPI00049F42D5 Oxidoreductase OS=Citrobacter amalonaticus OX=35703 GN=ILP74_11075 SS=EMBLWGS:MBE0395980 PC=UP000642697:Unassembled WGS sequenceMSDNIRVGLIGYGYASKTFHAPLIDGTPGLELAVVSSSDAAKVKADWPSVAVVSEPKHLFNDPNIDLIVIPTPNDTHFPLAKAALEAGKHVVVDKPFTVTLSQARELDALARSLGRVLSVFHNRRWDSDFLTLKAMLAEGVLGEVAYFESHFDRFRPQVRDRWREQGGPGSGIWYDLAPHLLDQAINLFGLPVSMTVDLAQLRPGAQSTDYFHATLSYPQRRVILHGTMLAAAESARYIVHGSRGSYVKYGLDPQEERLKNGERLPQEDWGYDMRDGIVTRVEGETRVEETWLTVPGNYPAYYAGIRDALNGHGENPVPASQAIQIMELIELGIESAKHRSTLCLA>UPI00049F4327 Trans-2-enoyl-CoA reductase family protein OS=Citrobacter amalonaticus OX=35703 GN=ILP74_22560 SS=EMBLWGS:MBE0398130 PC=UP000642697:Unassembled WGS sequenceMIIKPKIRGFICTTTHPTGCKVNVEKQIEYVKQNGKIENGPSRVLVIGASTGYGLASRISAAFGSGAATIGVFFEKPGTESKPGSAGWYNAAAFDEAAKREGLYSKSINGDAFSDECRDEVIKLIKEDLGQIDLVVYSLASPVRKMPKTGEVVRSALKPIGEVYTSKAIDTNKDQITTASIEPATEEEVQNTVTVMGGEDWELWIDALRQANVLADGVKTVAYSYIGTDLTWPIYWHGALGKAKEDLDRAAGALRNQLSSINGTANVAVLKSVVTQASSAIPVMPLYISMVFKLMKEQGIHEGCIEQINRLMTTSLYGDKAALDDHQRIRMDDWELREDIQQACRDLWPLITTENLAQETDYAGYKLEFLNLFGFGLDEVDYEADVNPEVEFDVVTL>UPI00049F43C5 Glyoxylate/hydroxypyruvate reductase GhrB OS=Citrobacter amalonaticus OX=35703 GN=ghrB SS=EMBLWGS:MBE0397897 PC=UP000642697:Unassembled WGS sequenceMKPSIILYKALPDDLLHRLEEHFTVTQVPDLSPQTVEQHAQAFASAEGLLGSSQTVDTALLEKMPTLRATSTISVGYDNFDVDALNARKVLLMHTPTVLTETVADTIMALVLSTARRVVEVAERVKVGEWTSSIGPDWFGIDVHHKTLGIVGMGRIGLALAQRAHFGFNMPILYNARRHHQEAEERFNARYCDLNTLLQESDFVCLILPLTDETHHLFGAEQFAKMKSSAIFINAGRGPVVDENALIAALQQGEIHAAGLDVFEQEPLPVDSPLLSLSNVVAVPHIGSATHETRYNMAACAVDNLIDALQGKVEKNCVNPQVAG>UPI00049F440E L-methionine sulfoximine/L-methionine sulfone acetyltransferase OS=Citrobacter amalonaticus OX=35703 GN=mddA SS=EMBLWGS:MBE0395857 PC=UP000642697:Unassembled WGS sequenceMSIRFASKEDCAAIAEIYNHAVLHTAAIWNDQTVDTDNRIAWYEARQALGYPVLVSVEGDVVTGYASFGDWRNFDGFRHTVEHSVYVHPDHQGKGLGRQLLSRLIEEARRCGKHVMVAGIESQNHASLHLHETLGFNTTAQMPQVGTKFGRWLDLTFMQLQLDDRHEPDAHG>UPI00049F4466 Cation transport regulator ChaB OS=Citrobacter amalonaticus OX=35703 GN=chaB SS=EMBLWGS:MBE0395664 PC=UP000642697:Unassembled WGS sequenceMPYQAKKDLPDSVQHVLPAHAQEIYKEAFNSAWEQYKDKADRRDDASREETAHKVAWSAVKKEYAKGEDEKWHKKS>UPI00049F4469 Protein translocase subunit SecF OS=Citrobacter amalonaticus OX=35703 GN=secF SS=EMBLWGS:MBE0396897 PC=UP000642697:Unassembled WGS sequenceMAQEYTVEQLNHGRKVWDFMRWDYWAFGISGLLLVLAIIVMGVRGFNWGLDFTGGTVIEITLEKPAEMDVMRDALEKAGFVDPLLQNFGSSHDIMVRMPPTEGANGGQVLGSKVLSVINESTNQNAAVKRIEFVGPSVGADLAQTGAMALLAALISILVYVGIRFEWRLAAGVVIALAHDVIITLGILSLFHIEIDLTIVASLMSVIGYSLNDSIVVSDRIRENFRKIRRGTPYEIFNVSLTQTLHRTLITSGTTLMVILMLFLFGGPVLEGFSLTMLIGVSIGTASSIYVASALALKLGMKREHMLQQKVEKEGADQPSILP>UPI00049F447A Dienelactone hydrolase family protein OS=Citrobacter amalonaticus OX=35703 GN=ILP74_22830 SS=EMBLWGS:MBE0398178 PC=UP000642697:Unassembled WGS sequenceMTSTPQTGFAPAASPIASTSVHTPDDAIVAGFTSIPSQGDEMPAFHARPKKSNGPLPVVIVVQEIFGVHEHIRDICRRLALDGYLAIAPELYFREGDPNDFADIPTLLSGLVAKVPDSQVLADLDHVASWASRNGGDVHRLMITGFCWGGRITWLYAAHNPQLKAAVAWYGKLVGDKSLNSPKHPVDVATDLNAPVLGLYGGQDNSISQESVETMRQALRAANANTEIVVYPDAGHAFNADYRSSYHEESAKDGWQRMLAWFAQYGSKK>UPI00049F44D3 Class 1 fructose-bisphosphatase OS=Citrobacter amalonaticus OX=35703 GN=fbp SS=EMBLWGS:MBE0394158 PC=UP000642697:Unassembled WGS sequenceMKTLGEFIVEKQHEFSHATGELTALLSAIKLGAKIIHRDINKAGLVDILGASGAENVQGEVQQKLDLFANEKLKAALKARDIVAGIASEEEDEIVVFEGCEHAKYVVLMDPLDGSSNIDVNVSVGTIFSIYRRVTPVGTPVTEEDFLQPGNKQVAAGYVVYGSSTMLVYTTGCGVHAFTYDPSLGVFCLCQERMRFPEKGNTYSINEGNYIKFPNGVKKYIKFCQEEDKSTQRPYTSRYIGSLVADFHRNLLKGGIYLYPSTASHPDGKLRLLYECNPMAFLAEQAGGKASDGKERILDITPESLHQRRSFFVGNNHMVEDVERFIREFPDA>UPI00049F45B3 Flavodoxin FldB OS=Citrobacter amalonaticus OX=35703 GN=fldB SS=EMBLWGS:MBE0394238 PC=UP000642697:Unassembled WGS sequenceMNIGLFYGSSTCYTEMAAEKIRDIIGPELVTLHNLKDDAPALMEQYDVLILGIPTWDFGEIQEDWEAVWDQLDELNLEGKIVALYGMGDQLGYGEWFLDALGMLHDKLSTKGVKFVGYWPTEGYEFTSPKPVIADGQLFVGLALDETNQYDLSDERIQAWCEQILGEMAEHYS>UPI00049F4612 Helix-hairpin-helix domain-containing protein OS=Citrobacter amalonaticus OX=35703 GN=ILP74_15770 SS=EMBLWGS:MBE0396859 PC=UP000642697:Unassembled WGS sequenceMKHGIKALLITLSFACAGMTQSALAASSTAAAKSPAVETSAQAPVAGQNKSAVPAKASTEEGTRVSINTASAEELARAMNGVGLKKAQAIVSYRDEYGPFKTVEDLKQVPGMGNALVERNLSALTL>UPI00049F46BB 16S rRNA (Guanine(1207)-N(2))-methyltransferase RsmC OS=Citrobacter amalonaticus OX=35703 GN=rsmC SS=EMBLWGS:MBE0394198 PC=UP000642697:Unassembled WGS sequenceMSAFTPASEVLLRHSDDFEQSRILFAGDLQDDLPARFESADRRVHTQQFHHWQVLSRQMGDNARFSLVAQAQDVADCDTFIYYWPKNKPEAQFQLMNILSLLPVGTDIFVVGENRSGVRSAEQMLAEYAPLNKIDSARRCGLYHGRLETQPMFDADKFWGEYHVDGLTIKTLPGVFSRDGLDVGSQLLLSTLTPHTKGKVLDVGCGAGVLSVALASHSPKVRLTLCDVAAPAVEASRATLAANGIEGEVFASNVFSEVTGRFDMIISNPPFHDGMQTSLDAAQSLIRGAVRHLNSGGELRIVANAFLPYPQVLDETFGFHEVIAQTGRFKVYRTVMTRQAKKA>UPI00049F4779 HMP-PP phosphatase OS=Citrobacter amalonaticus OX=35703 GN=cof SS=EMBLWGS:MBE0396856 PC=UP000642697:Unassembled WGS sequenceMARLAAFDMDGTLLMPNHLLGDETLSTLARLRERDITLTFATGRHVLEMRHILGTFSLDAFLITGNGTRIHSLEGEVLHRQDLDPAVAEIVLHQHWDTQASMHVFNDNGWFTGQEIPEMLHAHVYSGFRYQIVDVARIPADRVTKVCFCGDHDDLTRLKIQLEEVLGARAHLCYSAIDCLEVLPVGCNKGSALEVLSGHLGLSLAECMAFGDAMNDREMLGSVGRGLIMGNAMPQLIAELPHLPVIGHCRNQAVSHFLTHWLDYPNLPYSPE>UPI00049F4793 Agmatinase OS=Citrobacter amalonaticus OX=35703 GN=speB SS=EMBLWGS:MBE0397249 PC=UP000642697:Unassembled WGS sequenceMSTLGHQYDNSLVSNAFGFLRLPLNFQPYDSDADWVITGVPFDMATSGRAGGRHGPAAIRQVSTNLAWEHNRFPWNFDMRERLNVVDCGDLVYAFGDAREMSEKLQAHAEKLLAAGKRMLSFGGDHFVTLPLLRAHAKHFGKMALVHFDAHTDTYANGCEFDHGTMFYTAPNEGLIDPNHSVQIGIRTEFDKDNGFTVLDACQVNDRGVDDIIAQVKQIVGDMPVYLTFDIDCLDPAFAPGTGTPVIGGLTSDRAIKLVRGLKDLNIVGMDVVEVAPAYDQSEITALAAATLALEMLYIQAAKKGE>UPI00049F486C Dihydrodipicolinate synthase family protein OS=Citrobacter amalonaticus OX=35703 GN=ILP74_12860 SS=EMBLWGS:MBE0396315 PC=UP000642697:Unassembled WGS sequenceMRKFSGIIPPVSSTFHRDGTIDKTAMRQVADFLINKGVDGLFYLGTGGEFSQMNTAQRMAFAEEAVAVVGGRVPVLIGVGSPSTDEAVKLAQHAQACGADGVVAINPYYWKVASRNLDDYYQQIARSVTLPVILYNFPDLTGQDLTPETVKRLALQNENIVGIKDTIDSVGHLRTMINTVKSVRPSFSVFCGYDDHLLNTLLLGGDGAISASANFAPELSVGIYHAWREGDLATAATLNQKLLQLPAIYALETPFVSLIKYSMQCVGLPVETYCLPPILDVSEEAKEKVHALLVAQGIISL>UPI00049F492D Stringent starvation protein A OS=Citrobacter amalonaticus OX=35703 GN=sspA SS=EMBLWGS:MBE0397589 PC=UP000642697:Unassembled WGS sequenceMAVAANKRSVMTLFSGPTDIYSHQVRIVLAEKGVSFEIEHVEKDNPPQDLIDLNPNQSVPTLVDRELTLWESRIIMEYLDERFPHPPLMPVYPVARGESRLYMHRIEKDWYTLMNVIVNGSASEADVARKQLREELQAIAPVFGQKPYFLSDEFSLVDCYLAPLLWRLPQLGIEFSGAGAKELKGYMTRVFERDSFLASLTEAEREMRLGRG>UPI00049F495A YafY family transcriptional regulator OS=Citrobacter amalonaticus OX=35703 GN=ILP74_15950 SS=EMBLWGS:MBE0396895 PC=UP000642697:Unassembled WGS sequenceMTRRADRLFQIVQILRGRRLTTAALLAERLEVSERTIYRDIRDLSLSGVPVEGEAGSGYRLMAGFDLPPLMLTHRESEALIAAIRLLHTWGGDLLSRELESAQEKVLAILPEESRRKAEQARIFAPDFGRQQHSRSAFDMIHRAVSTQQVLALHYRDEAGQLTWRDVQPLGIFFQGEFWLLVAWCERREDYRCFRVDRCLSITPLDRRFSECADRSLSDFLRKIRDETHA>UPI00049F4A05 DUF1176 domain-containing protein OS=Citrobacter amalonaticus OX=35703 GN=ILP74_04260 SS=EMBLWGS:MBE0394705 PC=UP000642697:Unassembled WGS sequenceMRYRVIFLFLLGLIPARLLWAAPAQQAFSDWQVTCNNQNFCVARNTGEHGGLVMTLSRSAGAHTDAVLRLDLGGLTAPTNEADIAPRLLLDGKPLALSPDHWRMTPWHLMTDDPATIAEFLLTIQDGKAITLQKGNQTISLAGLKAALLFIDAQQKRVGSETAWIEKGDDPPLSVPPAPALKGVAVVNPTPTPLSLDERNDLLDYGNWRINGIKCSLDPSRREVRVTALTDDKALLMIACEAGAYNTIDLAWIVSRKKPLASHAVRLRLPFNSGAESNDMELMNAVFDEKSRELVTLAKGRGLTDCGIQTRWRFDGQRFRLVRYAEEPSCDGWHGPDAWPTLWITR>UPI00049F4AC8 Beta-ketoacyl-ACP synthase II OS=Citrobacter amalonaticus OX=35703 GN=fabF SS=EMBLWGS:MBE0396205 PC=UP000642697:Unassembled WGS sequenceMSKRRVVVTGLGMLSPVGNTVESTWKALLAGQSGISLIDHFDTSAYATKFAGLVKDFNCDDIISRKEQRKMDAFIQYGIVAGVQAMQDSGLEVTEENASRIGAAIGSGIGGLGLIEENHSSLVNGGPRKISPFFVPSTIVNMVAGHLTIMYGLRGPSISIATACTSGVHNIGHAARIIAYGDADAMVAGGAEKASTPLGVGGFGAARALSTRNDNPQAASRPWDKERDGFVLGDGAGMVVLEEYEHAKARGAKIYAEVVGFGMSSDAYHMTSPPENGAGAALAMVNALRDAAIEPGQIGYVNAHGTSTPAGDKAEAQAVKSVFGDAASRVMVSSTKSMTGHLLGAAGAVESIYSILALRDQAVPPTINLDNPDEGCDLDFVPHEARQVSGMEYTLCNSFGFGGTNGSLIFKKV>UPI00049F4AFE Outer membrane protein OmpW OS=Citrobacter amalonaticus OX=35703 GN=ompW SS=EMBLWGS:MBE0395701 PC=UP000642697:Unassembled WGS sequenceMKKLTVAALAVCTLLSGSVYAHEAGEFFIRAGSATVRPTEGADGTLGHLGGFNVSNNTQLGLTFTYMATDNIGVELLAATPFRHKVGTQATGDIATVHHLPPTLMAQWYFGDSSSKLRPYVGAGVNYTTFFDTKFNDDGQNAGLSDLSLKDSWGVAGQVGLDYLINRDWLINMSVWYMDIDTEAKYKSNGAVLPEGRYNDNIRLDPWVFMFSAGYRF>UPI00049F4B0B MurR/RpiR family transcriptional regulator OS=Citrobacter amalonaticus OX=35703 GN=ILP74_08775 SS=EMBLWGS:MBE0395546 PC=UP000642697:Unassembled WGS sequenceMNMLEKIQSQLEHLSKSERKVADVIIASPERTIHCSIATLAQEANVSEPTVNRFCRSMETRGFPDFKLHLAQSLANGTPYVNRNVDEDDSVESYTGKIFESAMASLDHVRQSLDKSAINRAVDLLTQAKKIAFFGLGSSAAVAHDAMNKFFRFNVPVIYSDDVVLQRMSCMNCSDDDVVVLISHTGRTKSLVELAQLARENDAMVIALTSADTPLAREATLAITLDVPEDTDIYMPMVSRLAQLTVIDVLATGFTLRRGAKFRDNLKRVKEALKESRFDKELLIKSNDR>UPI00049F4BA6 Flagellar basal-body rod protein FlgG OS=Citrobacter amalonaticus OX=35703 GN=flgG SS=EMBLWGS:MBE0396222 PC=UP000642697:Unassembled WGS sequenceMISSLWIAKTGLDAQQTNMDVIANNLANVSTNGFKRQRAVFEDLLYQTIRQPGAQSSEQTTLPSGLQIGTGVRPVATERLHSQGNLSQTNNSKDVAIKGQGFFQVLLPDGTSAYTRDGSFQVDQNGQLVTAGGFQVQPAITIPANALSITIGRDGVVSVTQQGQAAPVQVGQLNLTTFMNDTGLESIGENLYTETQSSGAPNESTPGLNGAGLLYQGYVETSNVNVAEELVNMIQVQRAYEINSKAVSTTDQMLQKLTQL>UPI00049F4D40 Molybdate ABC transporter permease subunit OS=Citrobacter amalonaticus OX=35703 GN=modB SS=EMBLWGS:MBE0396570 PC=UP000642697:Unassembled WGS sequenceMILTDPEWQAVLLSLKVSSLAVLFSLPFGIFFAWLLVRCTFPGKALLDSVLHLPLVLPPVVVGYLLLVSMGRRGFIGQWLYDWFGITFAFSWRGAVLAAAVMSFPLMVRAIRLALEGVDLKLEQAARTLGAGRWRVFFTITLPLTLPGIIVGTVLAFARSLGEFGATITFVSNIPGETRTIPSAMYTLIQTPGGESAAARLCLISIVLALISLLISEWLARLSRERTGR>UPI00049F4D64 Esterase YqiA OS=Citrobacter amalonaticus OX=35703 GN=yqiA SS=EMBLWGS:MBE0397408 PC=UP000642697:Unassembled WGS sequenceMSTLLYLHGFNSSPRSAKACLLKNWLHEHYPHVEMVVPQLPPYPADAAEMLESIVLEHGGEALGVVGSSLGGYYATWLSQCFMLPAVVVNPAVRPFELLTDYLGQNENPYTGQQYVLESRHIYDLKVMQIDPLEASDLIWLLQQTGDEVLDYRQAVAYYASCRQTVIEGGNHAFTGFEDYFNPIVDFLGLHSC>UPI00049F4E45 PTS lactose/cellobiose transporter subunit IIA OS=Citrobacter amalonaticus OX=35703 GN=ILP74_23985 SS=EMBLWGS:MBE0398387 PC=UP000642697:Unassembled WGS sequenceMEELETIIMELLVNAGAARSQALTALQLARKGDFAGAEQAMEESRDYVKLAHKIQTQLIGIDEGTGKLPVNLITVHSQDHLMNAMVIQDLAGDMIELYRRIPLAN>UPI00049F4E54 Energy-coupling factor ABC transporter ATP-binding protein OS=Citrobacter amalonaticus OX=35703 GN=ILP74_07430 SS=EMBLWGS:MBE0395305 PC=UP000642697:Unassembled WGS sequenceMLATTELWFRYQDAQVLKGLTLDFSQHAVTGLVGANGCGKSTLFMNLSGLLRPQQGAVLWQGKPLDYSKRGLLALRQQVATVFQDPDQQIFYTDIDSDIAFSLRNLGVEEGEIARRVDEALTLVDAQHFRQQPIQCLSHGQKKRVAIAGALVLRAKYLLLDEPTAGLDPSGRSQMIDIIKRIVAQGNHVVISSHDIDLIYEVSNAVYVLRHGEVLAHGEPGEVFARTELIEEAGLTQPWLVKLHAELGMPLCKTEDEFFRCMRQRAIKEAS>UPI00049F4EB7 Phosphopyruvate hydratase OS=Citrobacter amalonaticus OX=35703 GN=eno SS=EMBLWGS:MBE0394443 PC=UP000642697:Unassembled WGS sequenceMSKIVKVIGREIIDSRGNPTVEAEVHLEGGFVGMAAAPSGASTGSREALELRDGDKARFMGKGVLKAVGAVNGPIAQAILGKDAKDQAGIDKIMIDLDGTENKSNFGANAILAVSLANAKAAAAAKGMPLYEHIAELNGTPGKYSMPVPMMNIINGGEHADNNVDIQEFMIQPVGAKTLKEAVRMGSEVFHNLAKVLKAKGMNTAVGDEGGYAPNLGSNAEALAVIAEAVKAAGYELGKDITLAMDCAASEFYKDGKYVLAGEGNKAFTSEEFTHFLEDLTKQYPIVSIEDGLDESDWDGFAYQTKVLGDKIQLVGDDLFVTNTKILKEGIEKGIVNSILIKFNQIGSLTETLAAIKMAKDAGYTAVISHRSGETEDATIADLAVGTAAGQIKTGSMSRSDRVAKYNQLIRIEEALGEKAPYNGRKEIKGQ>UPI00049F4EFD PTS sugar transporter subunit IIC OS=Citrobacter amalonaticus OX=35703 GN=ILP74_21970 SS=EMBLWGS:MBE0398022 PC=UP000642697:Unassembled WGS sequenceMDTLVFASLMGLYYWFARLRLGYTFSAMLLQPVVVAVFVGLLLGNMQTAMIIGAGMQLVYLGVTSTPGGNVPSDPALAACISIPIAVKAGMDPNLAIALAIPFGVIGVFLDQLRRTLNAAWVHMADKHAETANMSGIMRCAFLYPALLGLALRFPVVFAANYFGQDVVESFLKLMPHWLTHSFEIMGGILPALGFAITIMVIGKKSLLPWFIGGFFAVLYLKVDIMAMAIFGTCVAFLIKGLAKNEGAA>UPI00049F4FB8 U32 family peptidase OS=Citrobacter amalonaticus OX=35703 GN=ILP74_19395 SS=EMBLWGS:MBE0397527 PC=UP000642697:Unassembled WGS sequenceMELLCPAGNLPALKAAIENGADAVYIGLKDDTNARHFAGLNFTEKKLQEAVSFVHQHRRKLHIAINTFAHPDGYARWQRAVDMAAQLGADALILADLAMLEYAAVRYPHIERHVSVQASATNEEAINFYHRNFDVARVVLPRVLSIHQVKQLARVTPVPLEVFAFGSLCIMAEGRCYLSSWLTGESPNTVGACSPARFVRWQQTPQGLESRLNEVLIDRYQDGENAGYPTLCKGRYLVDGERYHALEEPTSLNTLELLPELLAANIASVKIEGRQRSPAYVSQVAKVWRQAIDRCVADPQNYTPQAAWMETLGAMSEGTQTTLGAYHRKWQ>UPI00049F4FE2 Uncharacterized protein OS=Citrobacter amalonaticus OX=35703 GN=ILP74_00875 SS=EMBLWGS:MBE0394076 PC=UP000642697:Unassembled WGS sequenceMPGRFELKPTLAKIWHAPDNFRIMDPLPSMHRRGIIIAAIVLVVGFLLPSSDTNDTPAVTRNAQLDLQSQSQPPTEAQLQAQLVAPQNDPGQVAPVAPEPIQEGQPEEQPQSQQPQTQPFQQDSGIEQQWRSYRVEPGKTMAQLFRDHGLPPTDVYAMAQVEGAGKPLSTLQSGQMVQIRQNANGVVTGLTIDTGNNQQVLFTRQPDGSFIRVR>UPI00049F5020 GpW family protein OS=Citrobacter amalonaticus OX=35703 GN=ILP74_06120 SS=EMBLWGS:MBE0395055 PC=UP000642697:Unassembled WGS sequenceMASQSDLDSARAALHDLMTGKRVATVQKDGRRVEFTVTSVSDLKKYIADLEVQVGITQRRRGPAGFYV>UPI00049F5065 LysR family transcriptional regulator OS=Citrobacter amalonaticus OX=35703 GN=ILP74_07190 SS=EMBLWGS:MBE0395257 PC=UP000642697:Unassembled WGS sequenceMKPLLDVLIILDALEKEGSFAAASAKLYKTPSALSYTVHRLESDLNIQILDRSGHRARFTRTGQMLLEKGREVLHTVRELEKQAIKLHEGWENELVIGVDDTFPFSLLAPLIESFYQHHSVTRLKFINGVLGGSWDALIQGRADIIVGAMHEPPSSSDFSVARLGELEQIFAVAPHHPLAEEAEPLSRSTIKRYRAIVVGDSSPLAAATATQLLDDQEAITVFDFKTKLELQISGLGCGYLPRYLAQRFLESGALIEKKVAAQILFEPVWMGWNEQTAGLASAWWRDAILANSAIAGIYKKDDSEKSNI>UPI00049F513A IMP dehydrogenase OS=Citrobacter amalonaticus OX=35703 GN=guaB SS=EMBLWGS:MBE0394670 PC=UP000642697:Unassembled WGS sequenceMLRIAKEALTFDDVLLVPAHSTVLPNTADLSTQLTKTIRLNIPMLSAAMDTVTEARLAIALAQEGGIGFIHKNMSIERQAEEVRRVKKHESGVVTDPQTVLPTTTLREVKELTERNGFAGYPVVTEDNELVGIITGRDVRFVTDLSQPVSVYMTPKERLVTVREGEARDVVLAKMHEKRVEKALVVDDSFHLLGMITVKDFQKAERKPNSCKDEHGRLRVGAAVGAGAGNEERVDALVAAGVDVLLIDSSHGHSEGVLQRIRETRAKYPDLQIIGGNVATGAGARALAEAGVSAVKVGIGPGSICTTRIVTGVGVPQITAVSDAVEALEGMGIPVIADGGIRFSGDIAKAIAAGASAVMVGSMLAGTEESPGEIELYQGRSYKSYRGMGSLGAMSKGSSDRYFQSDNAADKLVPEGIEGRVAYKGRLKEIIHQQMGGLRSCMGLTGCGTIDELRTKAEFVRISGAGIQESHVHDVTITKESPNYRMGS>UPI00049F5191 DUF3313 domain-containing protein OS=Citrobacter amalonaticus OX=35703 GN=ILP74_10290 SS=EMBLWGS:MBE0395835 PC=UP000642697:Unassembled WGS sequenceMRTHTLFKVAVLTGLLALSGCASKVTQPDKYSGFLKDYSGLKETKSATGHPVLRWVDPNFSDTKYDNIVWNPITYYPVPKPSTQVGQNVLDQLLNYTNTKMKTSIGQRKPLVTTPGPRSLIFRGAITGVDTSKEGLQFYEVVPVALVVAGTQMATGHRTMDTHLYFEGELIDAATNKPVIKVVRQGEGKDLNNQNTPMAFETLKQVVDDMATDATMFDVNQK>UPI00049F51F9 Cell division ATP-binding protein FtsE OS=Citrobacter amalonaticus OX=35703 GN=ftsE SS=EMBLWGS:MBE0397807 PC=UP000642697:Unassembled WGS sequenceMIRFEHVSKAYLGGRQALQGVTFHMQPGEMAFLTGHSGAGKSTLLKLICGIERPSAGKILFSGHDITRLKNREVPFLRRQIGMIFQDHHLLMDRTVFDNVAIPLIIAGASGDDIRRRVSAALDKVGLLDKAKNFPIQLSGGEQQRVGIARAVVNKPAVLLADEPTGNLDDALSEGILRLFEEFNRVGVTVLMATHDIGLISRRSYRMLTLSDGHLHGGEARE>UPI00049F5230 Ribonuclease III OS=Citrobacter amalonaticus OX=35703 GN=rnc SS=EMBLWGS:MBE0394608 PC=UP000642697:Unassembled WGS sequenceMNPIVINRLQRKLGYTFTHQELLQQALTHRSASSKHNERLEFLGDSILSFVIANALYHRFPRVDEGDMSRMRATLVRGNTLAELAREFDLGECLRLGPGELKSGGFRRESILADTVEALIGGVFLDSDIQTVEKLILNWYQTRLDEISPGDKQKDPKTRLQEYLQGRHLPLPSYLVVQVRGEAHDQEFTIHCQVSGLSEPVVGTGSSRRKAEQAAAEQALKKLELE>UPI00049F5263 ATP-dependent Clp protease adapter ClpS OS=Citrobacter amalonaticus OX=35703 GN=clpS SS=EMBLWGS:MBE0396413 PC=UP000642697:Unassembled WGS sequenceMGKTNDWLDFDQLAADKVRDALKPPSMYKVILVNDDYTPMEFVIDVLQKFFSYDVERATQLMLAVHYHGKAICGVFTAEVAETKVAMVNTYARENEHPLLCTLEKA>UPI00049F527D DGTPase OS=Citrobacter amalonaticus OX=35703 GN=dgt SS=EMBLWGS:MBE0397064 PC=UP000642697:Unassembled WGS sequenceMSQIDFRKKINWHRRFRSPQGVKTEHEILRIFESDRGRIINSPAIRRLQQKTQVFPLERNAAVRTRLTHSMEVQQVGRYIAKEILSRLKEQKLLEPYGLDELTGPFESIVEMSCLMHDIGNPPFGHFGEAAINDWFRQRLHPADAESQPLTEDRCIVATLRLREGEESLNDIRRKVRQDLCHFEGNAQGIRLVHTLMRMNLTWAQVGGILKYTRPAWWRGETPATHNYLMKKPGYYLSEEPYIARLRKELDLAVYSRFPLTWIMEAADDISYCVADLEDAVEKRIFSVEQLYHHLYEAWGEHEKGSLFAQVVENAWDKSRANYLSRSTEDQFFMYLRVNTLNKLVPYAAQRFIDNLEQIFDGRFNQALLEDGSSFSRLLKIYKNVAMKHVFSHPDVEQLELQGYRVISGLLDIYRPLLSLPLADFSELVEKERLQRFPIESRLFQKLSTRHRLAYVEAVGKLTPDSPEYPVLEYYYRCRLIQDYISGMTDLYAWDEYRRLMAVEQ>UPI00049F52A9 DUTP diphosphatase OS=Citrobacter amalonaticus OX=35703 GN=dut SS=EMBLWGS:MBE0397971 PC=UP000642697:Unassembled WGS sequenceMMKKIDVKILDPRVGKQFPLPTYATSGSAGLDLRACLDDAVELAPGATTLLPTGLAIHIADPSLAAVILPRSGLGHKHGIVLGNLVGLIDSDYQGQLMVSVWNRGQDSFTIEPGERIAQMVFVPVVQAEFNLVEEFDATHRGEGGFGHSGRK>UPI00049F530D Glutamate/aspartate ABC transporter ATP binding protein GltL OS=Citrobacter amalonaticus OX=35703 GN=gltL SS=EMBLWGS:MBE0396681 PC=UP000642697:Unassembled WGS sequenceMITLKNVSKWYGHFQVLTDCSTEVKKGEVVVVCGPSGSGKSTLIKTVNGLEPVQKGEITVNGIMVNDKKTDLAKLRSRVGMVFQHFELFPHLSIIENLTLAQVKVLKRDKAPAREKAQKLLERVGLAAHANKYPAQLSGGQQQRVAIARALCMDPIAMLFDEPTSALDPEMINEVLDVMVELANEGMTMMVVTHEMGFARKVANRVIFMDEGKIVEDSPKEEFFANPKSERAKDFLAKILH>UPI00049F5328 Peroxiredoxin OS=Citrobacter amalonaticus OX=35703 GN=ILP74_15990 SS=EMBLWGS:MBE0396903 PC=UP000642697:Unassembled WGS sequenceMVLVTRQAPDFTAAAVLGSGEIVENFNFKQHTNGKTTVLFFWPMDFTFVCPSELIAFDKRYEEFQKRGVEVVGVSFDSEFVHNAWRNTPVDKGGIGAVKYAMVADVKREIQKAYGIEHPEAGVALRGSFLIDANGVVRHQVVNDLPLGRNIDEMLRMVDALQFHEEHGEVCPAQWEKGKEGMNASPDGVAKYLTENVSSL>UPI00049F5347 NADP(+)-dependent aldehyde reductase OS=Citrobacter amalonaticus OX=35703 GN=ybbO SS=EMBLWGS:MBE0396801 PC=UP000642697:Unassembled WGS sequenceMQKSVLITGCSSGIGLESALELKRQGFQVLAGCRKPDDVTRMNNMGFTGVLIDLDSPESVDRAADEVIALTDNCLYGIFNNAGYGVYGPLPTISRAQMEQQFSANFFGAHQLTMRLLPAMLPHGEGRIVMTSSVMGLISTPGRGAYAASKYALEAWSDALRMELRHSGIKVSLIEPGPIRTRFTENVNQTQNDKPVENPGIAARFTLDPEAVVAKVRHAFISDKPKLRYPVTLVTWAVMLLKRLLPGRLMDKILHG>UPI00049F539A Sugar efflux transporter SetB OS=Citrobacter amalonaticus OX=35703 GN=setB SS=EMBLWGS:MBE0395097 PC=UP000642697:Unassembled WGS sequenceMHNTPAAASPKTFDLTSTAFLIVAFLTGIAGALQTPTLSLFLTDEVHARPAMVGFFFTGSAVIGILVSQFLAGRSDKKGDRKKLIVFCCALGMLACVLFAWNRNYFILLFVGVFLSSFGSTANPQMFALAREHADRTGREAVMFSSILRAQVSLAWVIGPPLAYALAMGFSFTVMYLSAAVAFVVCGAMVWFFLPSMRKETALAIGTLEAPRRNRRDTLLLFAICTLMWGTNSLYIINMPLFIINELHLPEKLAGVMMGTAAGLEIPTMLIAGYFAKRLGKRLLMCIAVAAGFCFYTGMLVAHSPVVLLGLQLLNAIYIGILGGIGMLYFQDLMPGQAGSATTLYTNTIRVGWIIAGSLAGIAAEIWNYHAVFWFALVMIVATMACLSRIKDV>UPI00049F53C3 YjaG family protein OS=Citrobacter amalonaticus OX=35703 GN=ILP74_24050 SS=EMBLWGS:MBE0398400 PC=UP000642697:Unassembled WGS sequenceMLQNPIHLRLERLESWQHVTFMACLCERMYPNYAMFCQQTGFGDGQVYRRILDLIWETLTVKDAKVNFDSQLEKFEEAIPAADDYDLYGVYPAIDACVALSELVHSRLSGETLEHAIEVSKTSITTVAMLEMTQAGREMSDEELKDNPAVEQEWDIQWEIFRLLADCEERDIELIKGLRADLREAGESNIGINLQQ>UPI00049F5403 MFS transporter OS=Citrobacter amalonaticus OX=35703 GN=ILP74_13590 SS=EMBLWGS:MBE0396452 PC=UP000642697:Unassembled WGS sequenceMQNRLQQGARLGRQALLFPLCLVLYEFSTYIGNDMIQPGMLAVVEQYQAGIDWVPTSMTAYLAGGMFLQWLLGPLSDRIGRRPVMLAGVVWFIVTCLATLLAQSIEQFTLLRFLQGISLCFIGAVGYAAIQESFEEAVCIKITALMANVALIAPLLGPLVGAAWVHVLPWEGMFVLFAALAAIAFFGLQRAMPETATRIGEPLSLKALGNDYRLVLKNGRFVAGALALGFVSLPLLAWIAQSPIIIISGEQLSSYEYGLLQVPIFGALIAGNLMLARLTARRTVRSLIIMGGWPIVLGLLIAAAATVVSSHAYLWMTAGLSVYAFGIGVANAGLVRLTLFASDMSKGTVSAAMGMLQMLIFTVGIELSKHAYLLGGNGLFSLFNLASGMLWLLLMFIFLKDKQAGNTREG>UPI00049F54DB Transcriptional regulator AsnC OS=Citrobacter amalonaticus OX=35703 GN=asnC SS=EMBLWGS:MBE0398104 PC=UP000642697:Unassembled WGS sequenceMENYQIDNLDRGILDALMENARTAYAELAKQFGVSPGTIHVRVEKMKQAGIITGARIDVSPKQLGYDVGCFIGIILKSAKDYPSALARLESLDEVTEAYYTTGHYSIFIKVMCRSIDALQHVLINKIQTIDEIQSTETLIVLQNPIMRTIKP>UPI00049F54E9 Biofilm formation regulator BssS OS=Citrobacter amalonaticus OX=35703 GN=bssS SS=EMBLWGS:MBE0396239 PC=UP000642697:Unassembled WGS sequenceMEKNNEVIQTHPLVGWDISTVDSYDALMLRLHYQTPNRPETDGTEVGQTLWLTTDVARQFISILEAGIAKIESGDYQANEYRRH>UPI00049F55A9 Outer membrane lipid asymmetry maintenance protein MlaD OS=Citrobacter amalonaticus OX=35703 GN=mlaD SS=EMBLWGS:MBE0397560 PC=UP000642697:Unassembled WGS sequenceMQTKKNEIWVGIFLLAALLAALFICLKAANVTSMRTEPTYTIYATFDNIGGLKVRSPVRIGGVVVGRVADISLDPKTYLPRVTLDIEERYNHIPDTSSLSIRTSGLLGEQYLALNVGFEDPELGTSILKDGGTIQDTKSAMVLEDMIGQFLYNSNSKGDDNKNSGDASAPTEGHNEATGPAGTTN>UPI00049F55BD 6-carboxytetrahydropterin synthase QueD OS=Citrobacter amalonaticus OX=35703 GN=queD SS=EMBLWGS:MBE0394451 PC=UP000642697:Unassembled WGS sequenceMSTTLFKDFTFEAAHRLPHVPEGHKCGRLHGHSFMVRLEITGEVCPHTGWIMDFAELKAAFKPTYDRLDHYYLNDIPGLENPTSEVLAKWIWDQVKPVVPLLSAVMVKETCTAGCVYRGE>UPI00049F55FF Winged helix-turn-helix transcriptional regulator OS=Citrobacter amalonaticus OX=35703 GN=ILP74_00910 SS=EMBLWGS:MBE0394083 PC=UP000642697:Unassembled WGS sequenceMSASTLSQQLRDGNLFAEQCPSREVLKHVTSRWGVLILVALRDGTHRFSDLRRKMGGVSEKMLAQSLQALEQDGFINRVSYPVVPPHVEYSLTPLGEQVSDKVAALADWIELNLPQVLAQREERAA>UPI00049F5635 Glyceraldehyde-3-phosphate dehydrogenase OS=Citrobacter amalonaticus OX=35703 GN=gapA SS=EMBLWGS:MBE0396136 PC=UP000642697:Unassembled WGS sequenceMTIKVGINGFGRIGRIVFRAAQKRSDIEIVAINDLLDADYMAYMLKYDSTHGRFDGTVEVKDGHLIVNGKKIRVTAERDPANLKWDEVGVDVVAEATGIFLTDETARKHITAGAKKVVLTGPSKDNTPMFVKGANFDKYAGQDIVSNASCTTNCLAPLAKVINDNFGIIEGLMTTVHATTATQKTVDGPSHKDWRGGRGAAQNIIPSSTGAAKAVGKVLPELNGKLTGMAFRVPTPNVSVVDLTVRLEKAASYEDIKKAIKAASEGPMKGVLGYTEDDVVSTDFNGEVCTSVFDAKAGIALNDNFVKLVSWYDNETGYSNKVLDLIAHISK>UPI00049F5642 Membrane integrity-associated transporter subunit PqiA OS=Citrobacter amalonaticus OX=35703 GN=pqiA SS=EMBLWGS:MBE0396354 PC=UP000642697:Unassembled WGS sequenceMCEHHHAAKHILCSQCDMLVALPHLEHGQKAACPRCGTTLTVMWDAPRQRPTAYALVALFMLLLSNLFPFVNMNVAGVSSEVTLMQIPGVLFSENYASLGTFFLLFVQLVPAFCLLTILLLVNRVQMPARLKAQLARILFQLKSWGMAEIFLAGVLVSFVKLMAYGDIGVGSSFIPWCFFCILQLRAFQCVDRRWLWDDIAPMPVIKQTLTPGVPGIRQGLRSCSCCTAILPADEHLCPRCETKGYVRRRNSLQWTLALLFTSIMLYLPANILPIMITDLLGSKLPSTILEGVVLIWSEGSYPVAAVIFIASIMVPTLKMIAIAWLCWDAKGHGKRDSERMHLIYEVVEFVGRWSMIDVFVIAVLSALVRMGGLMNIYPAMGALMFALVVIMTMFSAMTFDPRLSWDREPEPGHEES>UPI00049F569D Cytochrome o ubiquinol oxidase subunit II OS=Citrobacter amalonaticus OX=35703 GN=cyoA SS=EMBLWGS:MBE0396869 PC=UP000642697:Unassembled WGS sequenceMRLRKYNKSLGWLSLIAGTALLSGCNSALLDPKGQIGLEQRSLILTAFGLMLIVVIPAILMAVGFAWKYRASNKDAKYSPNWSHSNKVEAVVWTVPILIIIFLAVLTWKTTHALEPSKPLAHDEKPITIEVVSMDWKWFFIYPEQGIATVNEIAFPANTPVYFKVTSNSVMNSFFIPRLGSQIYAMAGMQTRLHLIANEAGTYDGISASYSGPGFSGMKFKAIATEDRAAFDQWVAKAKQSPNTMSDMAAFEKVAVPSEYNQVEYFSNVKPDLFKDVINKFMAHGQSMDMTQPEGEHSSHEGMEGMDMSHAEAANSRG>UPI00049F56C1 YebC/PmpR family DNA-binding transcriptional regulator OS=Citrobacter amalonaticus OX=35703 GN=ILP74_08725 SS=EMBLWGS:MBE0395536 PC=UP000642697:Unassembled WGS sequenceMAGHSKWANTRHRKAAQDAKRGKIFTKIIRELVTAAKLGGGDPDANPRLRAAIDKALANNMTRDTLNRAIARGVGGDDDANMETIIYEGYGPGGTAVMIECLSDNRNRTVAEVRHAFSKTGGNLGTDGSVSYLFSKKGVISFEKGDEDAIMEAALEAGAEDVVTFDDGAIDVYTAWEEMGKVRDALEAAGLKADSSEVSMIPSTKAEMDAETAPKLMRLIDMLEDCDDVQEVYHNGEISDEVAATLE>UPI00049F56F0 Ferredoxin family protein OS=Citrobacter amalonaticus OX=35703 GN=ILP74_11550 SS=EMBLWGS:MBE0396068 PC=UP000642697:Unassembled WGS sequenceMSQENRVNVDVKLGVNKFHVDEGHPHIILAANPDSKEFQKLLNACPAGLYKQDEAGTIHFDSAGCLECGTCRVLCGETILEKWEYPAGTFGVDFRYG>UPI00049F57B7 L-threonine dehydrogenase OS=Citrobacter amalonaticus OX=35703 GN=yiaY SS=EMBLWGS:MBE0397924 PC=UP000642697:Unassembled WGS sequenceMAASTFYIPSVNIIGADSLKDAMNAMVEYGFRRTLIVTDSVLTKLGMAGDIQKALQKHDIFSVIFDGTHPNPTTINVADGLKILKENDCDSVISLGGGSPHDCAKGIALVAANGGDIRDYEGVDRSAKPQLPMIAINTTAGTASEMTRFCIITDVDRHIKMAIVDKHVTPLLSVNDSSLMIGMPKSLTAATGMDALTHAIEAYVSVAATPITDACALKAMTMISDNLVVAVESGGNVHAREAMAYAQFLAGMAFNNASLGYVHAMAHQLGGFYDLPHGVCNAVLLPHVQVFNSQVAAARLRDCAAAMGVQVAGMTDAEGAEACIEAIRALAQQVNIPAGLRALGVKEEDIPVLAANALKDACGLTNPIQATHDEIMAIYRAAM>UPI00049F57BA Flagellar type III secretion system protein FliR OS=Citrobacter amalonaticus OX=35703 GN=fliR SS=EMBLWGS:MBE0395382 PC=UP000642697:Unassembled WGS sequenceMLQVTSDQWLQWLSLYFWPLLRVLALITTAPILSERAVPKRVKLGLGIIITLVIAPSLPPNDVPIFSFNALWLAMQQILIGIALGFTMQFAFAAVRTAGEIIGLQMGLSFATFVDPASHLNMPVLARIIDMLAMLLFLTFDGHLWLISLLVDTFHTLPIGGNPVNSNAFLALARAGSLIFLNGLMLALPVITMLLTLNLALGLLNRMAPQLSVFVIGFPLTLTVGIALIAALMPLIAPFCEHLFSEIFNLLADIVSELPVNNSP>UPI00049F57D8 Phosphatidylglycerol--membrane-oligosaccharide glycerophosphotransferase OS=Citrobacter amalonaticus OX=35703 GN=opgB SS=EMBLWGS:MBE0394183 PC=UP000642697:Unassembled WGS sequenceMSELLSIALFLASVLIYACKAGRNTWWFIATLTVLGLFVILNITLFASDYFTGDGINDAVLYTLTNSLTGAGVGKYILPGIGIALALVAVFGTLGWVLRRRRHHPHHFGYSLLALLLALGSVDASPAFRQISELVKSQTREGDPDFAAYYKEPAKSIPNPKLNLVYIYGESLERTYFDNEAFPELTPELGALKNEGMDFSHTQQLPGTDYTIAGMVASQCGIPLFAPFEGNASASVSSFFPQNICLGDILKNSGYQNHFVQGANLRFAGKDVFLKSHGFDYLYGAEELKSVVADPNYRNDWGFYDDTVLDEAWKKFEELSRSGQRFSLFTLTVDTHHPDGFISRACQRKRYDFDGKPNQSFSAVSCSQENIAAFINKIKASPWFKNTVIVVSSDHLAMNNSAWKYLNKQDRNNLFFVIRGDKPQQETLAVKRNTMDNGATVLDILGGDNFIGLGRSSLSGQSMSEVFLNSKEKILAMKPDIIRLWNFPKEMKDFTVDRDKNMIAFSGSHFRLPLLLRVSDKRVEPLPESEYSAPLRYQLADFAPRDNFVWVDRCYKMGQLWSPELALSTDWCVSQGQLGGEQIVQHVDKAQWKGRTAFKDTMIDMERYKGNVDTLKIVDNDIRYKADSFIFNVAGAPEEVKQFSGISRPESWGRWSNAQLAEEVKIEYKQPLPKKFDLVITAKAFGDNANRPIPVRVGKEEQTLVLGHDVTTTTLHFDNPTEADTLVIVPPDPVPTNEGNILGHSPRKLGIGMVEIKVVSAQG>UPI00049F5908 Esterase OS=Citrobacter amalonaticus OX=35703 GN=yjfP SS=EMBLWGS:MBE0394068 PC=UP000642697:Unassembled WGS sequenceMIEIETCQLAEHHLLHAFPSGQRSTLLPCIVFYHGFTSSSLVYSYFAVALAQAGFRVIMPDAAGHGARFNGDEQARMGHFWQILQQSMQEFTALRAALRAENWLLEERLAVGGASMGAMTALGIMTQHPEVKCVASLMGSGYFTRLARTLFPPCALDTPARQEEFTHIIAPLAKWDVSQQLARLADRPLLLWHGQDDDVVPAAESFRLQQAMIQAGLDHNLTCQWQAGVRHRITPEALAATVSFFRQHL>UPI00049F5935 DUF805 domain-containing protein OS=Citrobacter amalonaticus OX=35703 GN=ILP74_19145 SS=EMBLWGS:MBE0397479 PC=UP000642697:Unassembled WGS sequenceMNWYLSVIKNYTGFSGRARRKEYWMFVLINMIICAVLNVIQAVIGMETPYISIIYSLGILLPSIAVAIRRLHDTERSGWWLLLSLIPIIGTIVIIVFLCQNGTAGANRFGADPKQNEIN>UPI00049F59BF DNA-binding transcriptional repressor OS=Citrobacter amalonaticus OX=35703 GN=ILP74_03160 SS=EMBLWGS:MBE0394503 PC=UP000642697:Unassembled WGS sequenceMKPRQRQAAILEHLQKQGKSSVEELAQYFDTTGTTIRKDLVVLENAGTVIRTYGGVMLNKDESDPPIDHKTLINTHKKERIAEAAVRFIHDGDSIILDAGSTVLQMVPMLTRFSNITVMTNSLHIVNALSELDNEQTILMPGGTFRKKSASFHGQLAENAFEQFSFDKLFMGTDGIDLSAGVTTFNEVYTVSKAMCNAAREVILMADSSKFGRKSPNVVCSLESVDKLITDAGIDPAFRQALEEKGIEVIITGETNE>UPI00049F5A08 Sulfofructosephosphate aldolase OS=Citrobacter amalonaticus OX=35703 GN=yihT SS=EMBLWGS:MBE0398217 PC=UP000642697:Unassembled WGS sequenceMTTYTLKDITRPSGGFAMLAVDQREAMRLMFAAAGTPTPVTDQHLTDFKVNAAKILSPYASAILLDQQFCYRQAVEQNAVAKSCAMIVAADAFIPGNGIPVDSVVIDKTINPQAVKQDGAKALKLLVLWRSDEDAQQRLEMVKEFNTLCHSQGLLSIIEPVVRPPRRGDQFDREQAIIDAAKELGDSGADLYKVEMPLQGKGTQQALLSASQHLNEHINMPWVILSSGVDEKLFPRAVSVAMSAGASGFLAGRAVWSSVIGLPDSEMMLRDVSAPKLQRLGEIVDEMMARRR>UPI00049F5A4A ATP-independent periplasmic protein-refolding chaperone OS=Citrobacter amalonaticus OX=35703 GN=spy SS=EMBLWGS:MBE0396109 PC=UP000642697:Unassembled WGS sequenceMRKLTALFVASTLALGAANLAHAADTTTAAPADAKPMMQHKGKFGPHHDMMFKDLNLTDAQKQQIRDIMKDKRDQMKRPPVEERRAMHDIIASDTFDKAKAEAQITKMEEQRKANMLAHMETQNKIYNILTPEQKKQFNANFEKRLTERPARDGKMPVPAE>UPI00049F5ACE Cytochrome c maturation protein CcmE OS=Citrobacter amalonaticus OX=35703 GN=ccmE SS=EMBLWGS:MBE0395016 PC=UP000642697:Unassembled WGS sequenceMNIRRKNRLWIACGVLVGLALTITLVLYALRANIDLFYTPGEILYGKRETQQMPEVGQRLRVGGMVMPGSVKRDPNSLKVNFSIYDAEGVVDVTYEGILPDLFREGQGVVVQGELGEKNHIQAKEVLAKHDENYTPPEVEKAMQDNHRRPESVYKDKTS>UPI00049F5AF4 NADH-quinone oxidoreductase subunit M OS=Citrobacter amalonaticus OX=35703 GN=nuoM SS=EMBLWGS:MBE0394943 PC=UP000642697:Unassembled WGS sequenceMLLPWLILIPFIGGFLCWQTERFGVKVPRWIALITMGLTLALGLQLWLQGGYSLTQSAGIPQWQSEFVLPWIPRFGISIHLAIDGLSLLMVVLTGLLGVLAVLCSWREIEKYQGFFHLNLMWILGGVIGVFLAIDMFLFFFFWEMMLVPMYFLIALWGHKASDGKTRITAATKFFIYTQASGLVMLIAILALVFVHHNATGVWTFNYEDLLKTPMSHGVEYLLMLGFFIAFAVKMPVVPLHGWLPDAHSQAPTAGSVDLAGILLKTAAYGLLRFSLPLFPNASAEFAPIAMWLGVIGIFYGAWMAFTQYDIKRLIAYTSVSHMGFVLIAIYTGSQLAYQGAVIQMIAHGLSAAGLFILCGQLYERLHTRDMRMMGGLWGKMKWLPALSMFFAVATLGMPGTGNFVGEFMILFGSYQVVPVITVISTFGLVFASVYSLAMLHRAYFGKAKSQIAQQELPGMSLRELFIILLLVVLLVLLGFYPQPILDTSHSAMSNIQQWFVNSVTTTRP>UPI00049F5B1E Protein/nucleic acid deglycase OS=Citrobacter amalonaticus OX=35703 GN=yhbO SS=EMBLWGS:MBE0397522 PC=UP000642697:Unassembled WGS sequenceMSKKIAVLITDEFEDSEFTSPAAEFRQAGHEVITIEKQAGKTVKGKKGEASVTIDKAIDEVRPADFDALLLPGGHSPDYLRGDDRFVTFTRDFVNTGKPVFAICHGPQLLISADVIRGRKLTAVKPIIIDVKNAGAEFYDQEVVVDKDQLVTSRTPDDLPAFNREALRILGA>UPI00049F5C19 Lactaldehyde reductase OS=Citrobacter amalonaticus OX=35703 GN=fucO SS=EMBLWGS:MBE0398253 PC=UP000642697:Unassembled WGS sequenceMSFMLALPKISLHGAGAIGDMVNLVANKQWGKALIVTDGQLVKLGLLDSLFAALDEHKMSYHLFDEVFPNPTEALVQKGYAAYQDANCDYLIAFGGGSPIDTAKAVKILTANPGPSTAYSGVGKVKNPGVPLVAINTTAGTAAEMTSNAVIIDSGRQVKEVIIDPNIIPDIAVDDASVMLEIPASVTAATGMDALTHAVEAYVSVGAHPLTDANALEAIRLINLWLPKAVDDGHNLEAREQMAFGQYLAGMAFNSAGLGLVHALAHQPGATHNLPHGVCNAILLPIIENFNRPNAVARFARIAQAMGVDTRGMSDEAASMEAINAIRALSKRVGIPAGFSQLGVTKEDIEGWLDKALADPCAPCNPRTASRDEVRELYLEAL>UPI00049F5CFB DNA-binding protein OS=Citrobacter amalonaticus OX=35703 GN=ILP74_02420 SS=EMBLWGS:MBE0394360 PC=UP000642697:Unassembled WGS sequenceMTRRIDYLIEKYHFTEINESPRIASQWKEVLAECQQENAGVEERLRIALLNVDYVTSFELPFRLLLTRTPQLIDKLRKEFALTQKNVLINDKRRGQVYSINADLSRVPDAFRYRLSSRIRRMDEETITTAPYQQVASQTKHPEERLRLALESGLQVNALDGLFWLGIQRIAADIQRLRASGMPILASDVEVFDSLTGTRRTVTAYHL>UPI00049F5D7C Galactose-1-epimerase OS=Citrobacter amalonaticus OX=35703 GN=galM SS=EMBLWGS:MBE0396582 PC=UP000642697:Unassembled WGS sequenceMLNETPALAPDGQPYRLLTLRNDAGMVVTLMDWGATLLSARIPLSDGSVREALLGCASPEHYQDQSAFLGASIGRYANRIADSRYTFSGETVSLLPSQGVNQLHGGPNGFDKRRWQIVNQNERQVLFALTSDDGDQGFPGNLCATAQYRLTDDNRISITYRATVDKPCPVNLTNHVYFNLDGDQTDVRNHKLQLLADEYLPVDEGGIPRNGLKPVAGTSFDFRTAKVIASEFLADDDQRKVKGYDHAFLLQAKGDSKKPVALLTSQDGKLQMEVYTSAPALQFYSGNFLGGTSSRGPNAYDDYQGLALESEFLPDSPNHPEWPQPDCVLRPGEEYASLTEYRFIPS>UPI00049F5DFD Pyruvate kinase PykF OS=Citrobacter amalonaticus OX=35703 GN=pykF SS=EMBLWGS:MBE0396044 PC=UP000642697:Unassembled WGS sequenceMKKTKIVCTIGPKTESEEMLTKMLDAGMNVMRLNFSHGDYAEHGQRIQNLRNVMSKTGKKAAILLDTKGPEIRTIKLEGGNDVSLKAGQTFTFTTDKSVVGNNEIVAVTYEGFTSDLSVGNTVLVDDGLIGMEVTAIEGNKVICKVLNNGDLGENKGVNLPGVSIALPALAEKDKQDLIFGCEQGVDFVAASFIRKRSDVVEIREHLKAHGGEKIQIISKIENQEGLNNFDEILEASDGIMVARGDLGVEIPVEEVIFAQKMMIEKCIRARKVVITATQMLDSMIKNPRPTRAEAGDVANAILDGTDAVMLSGESAKGKYPLEAVTIMATICERTDRVMTSRLDFNNDSRKLRITEAVCRGAVETAEKLEAPLIVVATQGGKSARAVRKYFPDATILALTTNEVTARQLVLSKGVVAHLVKEIASTDDFYRLGKEVAQQSGLAQKGDVVVMVSGALVPSGTTNTASVHVL>UPI00049F5E0D Outer membrane protein assembly factor BamC OS=Citrobacter amalonaticus OX=35703 GN=bamC SS=EMBLWGS:MBE0394692 PC=UP000642697:Unassembled WGS sequenceMAYSVQKSRLARVAGVSLVLLLAACSSDSRYKRQVSGDESYLEAAPLAELHAPAGMILPVTTSDYNIPVTNGSGAVGKALDIRPPAQPLALVSGARTQFAGDTATLLVENGRGNTLWPQVVSVIQSKNYTIEKRDDASQTLTTGWVDWNRLDEDEQYRGRYQISVKPQGYQQAVMVKLVNLEQAGKPVADAASLQRYSTEMMNVISAGLDKTATDAANAAQNRSAATMDVQSAADDTGLPMLVVRGPFNVVWQRLPAALEKVGMKVTDSTRSQGSMAVTYKPLSDSGWQELGARDPGLSSGDYKLQVGDLDNRSSLQFIDPKGHTLTQSQNDALVAIFQAAFSK>UPI00049F5E18 Tyrosine-type recombinase/integrase OS=Citrobacter amalonaticus OX=35703 GN=ILP74_02380 SS=EMBLWGS:MBE0394353 PC=UP000642697:Unassembled WGS sequenceMNRRRYLTGKEVQAMMQAARHGATGERDYCLILLAFRHGMRISELLDLHYRDLDLNEGRINVRRLKNGFSTIHPLRFDEREAVERWSQERAGWRGAGRTDAVFISRRGTPLSRQQAYRIIRAAGVDAGTVTHTHPHMLRHACGYELAERGADTRLIQDYLGHRNIRHTVRYTASNAARFAGLWERSSLLEVISQEKKK>UPI00049F5EB2 Uroporphyrinogen-III C-methyltransferase OS=Citrobacter amalonaticus OX=35703 GN=cobA SS=EMBLWGS:MBE0396614 PC=UP000642697:Unassembled WGS sequenceMKNSGKVWLVGAGPGDISLLTLKALQCIQQADVIIYDRLVNPKMLEWCSASCTCINVGKSPGYHSVPQEEINALLIQHARTQRNVVRLKGGDPYVFGRGAEEVECLVEEGIAFEVVPGISSAIGGLACAGIPVTHRDLASGFHVVTGHTREGNQQQDWRQLAKLNGTLVIVMGIANLPFICEELLMGGKSPATPAAIVMSATRDNQRRLTCTLGTLLTKAQEANIVPPALIVVGDVVRLSDRLSFIPEVMERAYSERCS>UPI00049F5EF8 DNA-binding transcriptional regulator DecR OS=Citrobacter amalonaticus OX=35703 GN=decR SS=EMBLWGS:MBE0396854 PC=UP000642697:Unassembled WGS sequenceMLDKIDRKLLALLQQDCTLSLQALADAVNLTTTPCWKRLKRLEDEGILVGKVALLDPEKLGLGLTAFVLIKTQHHSSDWYCRFVTVVSEMPEVLGFWRMAGEYDYLMRVQVADMKRYDDFYKRLVNSVPGLSDVTSSFAMEQIKYTTALPIE>UPI00049F5EFF Exodeoxyribonuclease X OS=Citrobacter amalonaticus OX=35703 GN=exoX SS=EMBLWGS:MBE0395554 PC=UP000642697:Unassembled WGS sequenceMLRIIDTETCGLQGGIVEIASVDVVDGKIVNPMSHLVRPDRPISPQAMAIHRITEAMVADKPWIEEVIPHYYGSEWYVAHNASFDRRVLPDMPGEWICTMKLSRRLWPGIKYSNMALYKSRKLNVQTPAGLHHHRALYDCYITAALLIDIMNTSGWTAEQMVDITGRPGLLTTFTFGKYRGKAVSDVAERDPGYLRWLFNNLDSMSPELRLTLKHYLDNA>UPI00049F5F07 2-C-methyl-D-erythritol 2,4-cyclodiphosphate synthase OS=Citrobacter amalonaticus OX=35703 GN=ispF SS=EMBLWGS:MBE0394462 PC=UP000642697:Unassembled WGS sequenceMRIGHGFDVHAFGGEGPIIVGGVRIPYEKGLLAHSDGDVALHALTDALLGAAALGDIGKLFPDTDPAFKGADSRELLREAWRRIQAKGYTLGNVDVTIIAQAPKMLPHIPQMRVFIAEDLGCHMDDVNVKATTTEKLGFTGRGEGIACEAVALLIKAAK>UPI00049F5F4F Substrate-binding domain-containing protein OS=Citrobacter amalonaticus OX=35703 GN=ILP74_23815 SS=EMBLWGS:MBE0398364 PC=UP000642697:Unassembled WGS sequenceMKKLLLPCLITVALSSTAAWAEQSTAPQKANKPFTMGVVVKVGGIPWFNVMEQGIKEEGKALGVNAWQVGPTTADPAEQVRAIEDLIAKKVDVIGVVPNDAKVLEPVLKRAQEAGIKVITHESPDQANADWDFELLNTQTMGANHMKDMAKCMGEEGKYAMFVGSLTVPLVNDWANAAIAYQKEHYPKMTLVEDRFGVAESVDDSMRTANDLMSKYKDLKGIMSFGSQGPIGAGRAIDKRKKNDAICVFGTFTPGQGIKLLEKGAIDGGYISNPMIAGKVFVQVATAMMNGEPIKDGVKIGDMGEIKVQNNTILSDNPEKLDVENTRRLVKLGL>UPI00049F5F7E NADPH-dependent 2,4-dienoyl-CoA reductase OS=Citrobacter amalonaticus OX=35703 GN=ILP74_19045 SS=EMBLWGS:MBE0397460 PC=UP000642697:Unassembled WGS sequenceMRYPSLFAPLDLGFTRLKNRVLMGSMHTGLEEYPDGAERLAAFYAERARHGVALIVTGGIAPASSGVTMEGGATLNDAREVPHHRIITDAVHREGGKIALQILHTGRYSYQPQLVAPSAIQAPINRFTPHALSHEEILQLIEDFAHCAQLAREAGYDGVEVMGSEGYLINEFLALRTNQRDDEWGGDYTNRMRFAIEVVRAVRQRVGNDFIIIYRLSMLDLVEKGGTFAETVQLAQAIEAAGATLINTGIGWHEARIPTIATPVPRGAFSWVTRKLKGHVSLPLVTTNRINDPQVADEILARGDADMVSMARPFLADAELLSKAQSGRADEINTCIGCNQACLDQIFVGKITSCLVNPRACHETKMPVVPASTIKNLAVVGAGPAGLAFAINAAARGHHVTLFDALGEIGGQFNIAKQIPGKEEFYETLRYYRRMIDVTGVTLKLNHYVAADDLQAFDEVILASGIEPRRPPIDGIDHPKVLTYLDVLRDKTPVGNRVAIVGCGGIGFDTAMYLSQPGEPTSQNIADFCVEWGIDTSLQQAGGLRPEGPHLARSPRQIVMLQRKASKPGEGLGKTTGWIHRTTLLSRGVKMIPAVSYQKIDDTGLHVLIGGEAQTLEVDNVIICAGQEPRRELAEPLHAAGKTVHLIGGCDVAMELDARRAIAQGTRLALGI>UPI00049F5FA8 6-phospho-beta-glucosidase OS=Citrobacter amalonaticus OX=35703 GN=ILP74_03115 SS=EMBLWGS:MBE0394494 PC=UP000642697:Unassembled WGS sequenceMAVFPQGFLWGGALAANQAEGAYREGGKGLTTVDMIPHGEHRMPVKLGQEKRFQLRNDEFYPSHQAIDFYHRYKDDIALMAEMGFTVFRTSIAWSRIYPNGDELTPNEEGIAFYRAVFAECKKYGIEPLVTLCHFDVPMHLVTEYGSWRNRKMVEFFTRYARTCFEAFNGLVKYWLTFNEINIMLHSPFSGAGLVFEEGENQDQVKYQAAHHELIASALATKIAHEINPQNQVGCMLAGGNFYPYSCKPEDVWTALEKDRENLFFIDVQARGAYPAYSARVFREKGVVIEKAPADDAILKNTVDFVSFSYYTSRCASAEMNTKNSSAANVVKSMRNPYIQVSDWGWGIDPLGLRITMNMMYDRYQKPLFLVENGLGAKDEIDADGHINDDYRISYLREHIRAMADAIEDGVPLMGYTTWGCIDLVAASTGEMSKRYGFVYVDRDDAGNGTLARTRKKSFWWYKKVIASNGQDLA>UPI00049F5FFB ImmA/IrrE family metallo-endopeptidase OS=Citrobacter amalonaticus OX=35703 GN=ILP74_18090 SS=EMBLWGS:MBE0397281 PC=UP000642697:Unassembled WGS sequenceMIVTQDFNPDWVSPPGDTIIDLMDEHGLSDEQLSRKMGLPLPKGQKLLKGEICLDEVIATKLQDIFNVSISFWLKREMAYRSQVAYINNVNDEWLASLPVKDMTKLGWIAKCSSKELKLKKCLEFFDINSVRDFYDKINTEAPLVAFRKSLSFKTEPMADLAWLTRAAQISKQRDHVAWSKEKLKSLIPEIRKLTTEPKLSTFIPALEQKLASAGVSFVVLPTPAGCRASGATCFFEYNKPTVIVSFRYLTDDHFWFTLFHELGHLILHDELSVRLEGDIDVSNKDEQEANRFAADTLIPCEFRKQLENCSVKNWKPILRIARKIGVSKGICLGYLQHREIIPYTHLNKFKVRYKKEDII>UPI00049F6064 Tryptophan--tRNA ligase OS=Citrobacter amalonaticus OX=35703 GN=trpS SS=EMBLWGS:MBE0397734 PC=UP000642697:Unassembled WGS sequenceMTKPIVFSGAQPSGELTIGNYMGALRQWVNMQDDYHCIYCIVDQHAITVRQDAQQLRKATLDTLALYLACGIDPEKSTIFVQSHVPEHAQLSWALNCYTYFGELSRMTQFKDKSARYAENINAGLFDYPVLMAADILLYQTNLVPVGEDQKQHLELSRDIAQRFNALYGDIFKVPEPFIPKSGARVMSLLEPTKKMSKSDDNRNNVIGLLEDPKSVVKKIKRAVTDSEEPPVVRYDVQNKAGVSNLLDILSAVTGQSIPELEKQFEGKMYGHLKGEVADAVSGMLTELQERYYRYRNDEAFLQQVMKDGAEKAGARASATLKAVYEAIGFVARP>UPI00049F60F0 Diacylglycerol kinase OS=Citrobacter amalonaticus OX=35703 GN=ILP74_24355 SS=EMBLWGS:MBE0398457 PC=UP000642697:Unassembled WGS sequenceMANNTTGFTRIIKAAGYSWKGFRAAWINEAAFRQESVAALLAVAIACWLDVDAITRVLLIGSVMLVMIVEILNSAIEAVVDRIGSDYHELSGRAKDMGSAAVLLSIIVALITWGLLLWPHFR>UPI00049F6142 Phospholipase A OS=Citrobacter amalonaticus OX=35703 GN=pldA SS=EMBLWGS:MBE0398170 PC=UP000642697:Unassembled WGS sequenceMRAILAWLLPAALLPLAAYAQEATVKEVHDAPAVRGSIIANMLQEHDNPFTLYPYDTNYLIYTNTSDMNKEAISSYNWSENARKDEVKFQLSLAFPIWRGIMGPNSVLGASYTQKSWWQLSNTKESSPFRETNYEPQLFLGFATDYRVAGWTLRDVEMGYNHDSNGRSDPTSRSWNRLYTRLMAENGNWLVEVKPWYVIGSTDDNPDITKYMGYYQLKVGYHLGDAVLSAKGQYNWNTGYGGAELGLSYPMTKHIRLYTQVYSGYGESLIDYNFNQTRVGVGVMLNDIF>UPI00049F62EA DUF4160 domain-containing protein OS=Citrobacter amalonaticus OX=35703 GN=ILP74_18505 SS=EMBLWGS:MBE0397359 PC=UP000642697:Unassembled WGS sequenceMPVILRINGFRFFFYSNEGNPLEPAHIHVMKAGSEAKFWLTPSVALANNDGFNSRVLKELTGIVEDNQALFLEAWNDYFS>UPI00049F636C YqgE/AlgH family protein OS=Citrobacter amalonaticus OX=35703 GN=ILP74_17970 SS=EMBLWGS:MBE0397259 PC=UP000642697:Unassembled WGS sequenceMNLQHHFLIAMPALQDPIFRRSVVYICEHNEDGAMGIIVNKPLENLQIEGILEKLKIAPEPRDPAIRLDKAVMLGGPLAEDRGFILHTPPSRFASSIRISDNTVITTSRDVLETLGTNEQPADVLVALGYSSWEKGQLEQELLDNAWLTAPADLNILFKTPIADRWREAARLIGIDILTMPGVAGHA>UPI00049F637E Class II fructose-bisphosphate aldolase OS=Citrobacter amalonaticus OX=35703 GN=ILP74_23195 SS=EMBLWGS:MBE0398245 PC=UP000642697:Unassembled WGS sequenceMSLYNFNEILKIGQERNFKAIGSFNLHCIEMLPAFFKAAQKTNSPLMIQISTGTAEYLGYRLLVDAVRSLAESENVPTCLHLDHCSDISAIETAMNAGFSSVMYDGSHLELEENIGNTRIVVEMARPRNITVEGELGAIGGSEDGKAVAAEDICFTTVEDAKRFVEETRVDMLAVSVGTVHGLYTGKAQIQHQRLQEISAATGVPLVLHGGTGVSDDDMRLAVTEGINKVNVGTEMNVQWVDQCKNTFEKGKVNDSVRKFLIPANNAVTQVLMEKIALFK>UPI00049F6392 DUF3811 domain-containing protein OS=Citrobacter amalonaticus OX=35703 GN=ILP74_24215 SS=EMBLWGS:MBE0398429 PC=UP000642697:Unassembled WGS sequenceMALPRITQKEMTEREQRELKTLLDRARIAHGRQLTNAETNSVKKEYIDKLMVLREAEAKKARQLKKKQAYKPDAEASFSWSANTPTRGRR>UPI00049F642A Dipeptide/tripeptide permease DtpB OS=Citrobacter amalonaticus OX=35703 GN=dtpB SS=EMBLWGS:MBE0397851 PC=UP000642697:Unassembled WGS sequenceMNTSAPTGLLQQPRPFFMIFFVELWERFGYYGVQGILAVFFVKQLGFSQEQAFITFGAFAALVYGLISIGGYVGDHLLGTKRTLVLGAVVLALGYFMTGMSLLKPDLIFIALGTIAVGNGLFKANPASLLSKCYPPKDPRLDGAFTLFYMSINIGSLLSLSLAPVIADKFGYAVTYNLCGAGLIVALLVYFACRGMVKDIGSEPDHLPMSFRNLLYVLIGTVAMVFLCAWLMHNVKIANLVLIVLSIAVTIIFFRQAFRLDKTGRNKMFVAFILMLEAVLFYILYAQMPTSLNFFAINNVHHEILGFTINPVSFQALNPFWVVVASPVLAAIYTRLGSKGKDLTMPMKFTLGMFLCSLGFLTAAAAGMWFADAQGLTSPWFIVLVYLFQSLGELLISALGLAMVAALVPQHLMGFILGMWFLTQAAAFLLGGYVATFTAVPENITDPLQTLPIYTGVFSKIGLVTLAVTVVMALMVPWLNRMINTPDTAQ>UPI00049F6458 Uncharacterized protein OS=Citrobacter amalonaticus OX=35703 GN=ILP74_04060 SS=EMBLWGS:MBE0394666 PC=UP000642697:Unassembled WGS sequenceMEIPELTDDAVVELAREGGVAFMPKLARQRKIALSTLSTVQRQRVADILRQSLTVGMPPGQTGSPGRGDQRYFRIQIIWTQHNQAGVTDIIVLVPENDAPASLVDLWQKGEACICD>UPI00049F6590 Terminase small subunit OS=Citrobacter amalonaticus OX=35703 GN=ILP74_06110 SS=EMBLWGS:MBE0395053 PC=UP000642697:Unassembled WGS sequenceMEVNKKRLSDIFGVSVRTIQNWQDQGMPVARGGGKGNEVLYDSAAAIEWYSARDAEIENEKLRKEVEDLRIASESDLQPGTIEYERHRLTRAQADAQELKNAKDTAEVVETAFCTFVLSRIAGEIASILDGIPLSVQRRFPELENRHIDFLKKDIIKAMNKAAALDEIIPGLLSEYIEQSG>UPI00049F65AC TlpA family protein disulfide reductase OS=Citrobacter amalonaticus OX=35703 GN=ILP74_12665 SS=EMBLWGS:MBE0396277 PC=UP000642697:Unassembled WGS sequenceMRWLNVFILSLAVLLSGCKEEKLAVGETAPALAAFDLQGQESGLDRWQGKSIYLNFWSAGCGGCLAEMDSLETLSQKWGDSVVVVAVNTDPGTVNLDALLAKHQVSYPVLRDQMKITQERYQVIGTPTSVLIDPQGRVLEWHQGMRKPAELAATFARLAGQ>UPI00049F671F Ribosome-associated protein YbcJ OS=Citrobacter amalonaticus OX=35703 GN=ybcJ SS=EMBLWGS:MBE0396787 PC=UP000642697:Unassembled WGS sequenceMATFSLGKHPHVELCDLLKLEGWSESGAQAKMAIAEGQVKVDGAVETRKRCKIVAGQTVSFAGQTITVTA>UPI00049F672C Threonine synthase OS=Citrobacter amalonaticus OX=35703 GN=thrC SS=EMBLWGS:MBE0397216 PC=UP000642697:Unassembled WGS sequenceMKLYNLKDHNEQVSFAQAVTQGLGKNQGLFFPHDLPEFNLTEVDEMLNQDFVSRSTKILSAFIGDEIPQEILEERVRAAFAFPAPVAQVEGDVGCLELFHGPTLAFKDFGGRFMAQMLTHISGDKPVTILTATSGDTGAAVAHAFYGLPNVRVVILYPNGKISPLQEKLFCTLGGNIETVAIDGDFDACQALVKQAFDDEELKVALGLNSANSINISRLLAQICYYFEAVAQLPQEARNQLVVSVPSGNFGDLTAGLLAKSLGLPVKRFIAATNVNDTVPRFLQDGQWAPKATQATLSNAMDVSQPNNWPRVEELFRRKIWRLNELGYAAVDDETTQETMRELKAKGYTSEPHAAVAYRALRDQLNPGEYGLFLGTAHPAKFKESVEAILNETLDLPKELAERADLPLLSQHLPADFAALRKLMMTR>UPI00049F6774 LacI family DNA-binding transcriptional regulator OS=Citrobacter amalonaticus OX=35703 GN=ILP74_09880 SS=EMBLWGS:MBE0395756 PC=UP000642697:Unassembled WGS sequenceMTPTIYDIARVAGVSKSTVSRVLNKQTNISPEALEKVQRAIDELQYQPNKLARALTSSGFDAIMVISTRSTKTTAGNPFFSEVLHAITAKAEEEGFDVILQTSRSTEDDLQKCESKIKQKMIKGIIMLSSPADETFFERLDKYDIPVVVIGKVEGQYTHVYSVDTDNYRDSIALTDALIDSGHQNIACLHAPLEYHVSVDRVNGYKRSLATHQLAENKRWIIDGGYTHESALEAARALLNQHPLPDAVFATDSIKILSLYRAASERKIAIPQQLAVVGYSNEMLSFILTPPPGGIDVPTRELGEQSCDLLFKHIAGKTDARSITVETRISLTASLA>UPI00049F6788 Transcriptional regulator BolA OS=Citrobacter amalonaticus OX=35703 GN=bolA SS=EMBLWGS:MBE0396866 PC=UP000642697:Unassembled WGS sequenceMMIREQIEEKLRAAFQPVFLEVVDESYRHNVPAGSESHFKVVLVSDRFTGERFLNRHRMIYGTLTAELSTTVHALALHTYTIKEWEGLQDTIFASPPCRGAGSIA>UPI00049F6798 MFS transporter OS=Citrobacter amalonaticus OX=35703 GN=ILP74_08665 SS=EMBLWGS:MBE0395524 PC=UP000642697:Unassembled WGS sequenceMKTRKIGLVNYFAYGSGDFLGAGTTALTAAWLLYFYTTFCGLSPIEATFIFAAARVLDAVVSPLMGFLTDNFGSTWLGKRFGRRKFFILLGIPCVFSYSLMWVGDMGFWYYLLTYLLFDIVYTMILVPYETLVPEMTDDFKQKTKFSGARIGMAQMSAILASFLPGILLTAFGKDNPISFFYASLVFSVLCALMLTFVWFFTWERPREEWTEAALRAEEEKKSLTFSQSMNRLFVELSSTLRIKIFRQHLGMYLGGYIAQDVFNAVFTYYVVFVLMQEASMASNLLGTMAIFQFIAVIAMIPLCIRFGPAPSYRMVVVLFGLSSLSYAVLYYAGLSDIYSLLLLVSAVAGLGRGGINYVPWNTYTYIADVDEVITGQRREGIFAGIMTLTRKASQAGAVMLVGIVMQMSGFVSGQSTQPEAVSHTILLILSCGTLLVLACGFLVSLRFKLNLQTHSTLREETVKMRESGRTMPESITPQARATVEMLAGLPYESLWGNNNIGYLNRNKPAAPSLKQAAIWNSTYHRG>UPI00049F67BC Putrescine ABC transporter permease PotH OS=Citrobacter amalonaticus OX=35703 GN=potH SS=EMBLWGS:MBE0396439 PC=UP000642697:Unassembled WGS sequenceMSTLEPPARVEKPGGFTFWLARVQMKHGRKLVIALPYVWLILLFLLPFLIVFKISLAEMARAIPPYTELMEWADGQLSITLNLGNFLQLTDDPLYFDAYLQSLQVAGISTLCCLLLGYPLAWAVAHSKPSTRNILLLLVILPSWTSFLIRVYAWMGILKNNGVLNNFLLWLGVIDQPLTILHTNLAVYIGIVYAYLPFMVLPIYTALTRIDYSLVEASLDLGARPLKTFFSVIVPLTKGGIIAGSMLVFIPAVGEFVIPELLGGPDSIMIGRVLWQEFFNNRDWPVASAVAIVMLLLLIVPIMWFHKHQQKSGGAHG>UPI00049F67C5 DNA topoisomerase III OS=Citrobacter amalonaticus OX=35703 GN=ILP74_18520 SS=EMBLWGS:MBE0397362 PC=UP000642697:Unassembled WGS sequenceMQLFLCEKPSQAKDIARVLGISKREQGFISGGNIVVTWAVGHLLETASPEAYGEQYGRPWRADVLPVLPETWEMVVKEQTKSQFTVISKLLKKASEVVIATDADREGEVIARELLEYCRYSGAVRRLWLSALDEASVKEALSNILPGEKTALLYDAGKGRSQADWLIGMNLTRLYTLKARDSGVSEVLSVGRVQTPTLAMVVNRDNEITSFVPKPWWQVHALIEKEGVRFRAGWVPVEQYCDEEKRCINPQAARAVGQLCQQQGRATVLEVTQKREKTAAPLCFDLGTLQQVCSRKFGMGANDVLAIAQALYETHKATTYPRTDCGFLPTSMQQEIPDVLAAVAKSDPAVAPVLNQLDRQFVSRVWNDKKITAHHAIIPTRQAFDLSRLSADEQKVYHLIRQHYFAQFLPLQESDVTEASFNIGGQLFRTRGKVGVVTGWKSLFQAEKDDDEEDVDGDSMALPALVKGDICAVTGSEVKDMKTSPPKPFTEGTLIAAMKNAASFVSDPKLKKVLRDNAGLGTEATRAAVLETLFKRHYLEKKGKHIHSTQMARELIAALPETLTSPGMTALWEQALDDISQGKMSLAVFMQKQLQWTRHLVEKGRQDSVKITAPVTPPCPLCKGPTRKRKGKNGDFWGCIRYPDCEGIISTGKKKAAKRKKTSVKAKTE>UPI00049F6887 Aromatic acid/H+ symport family MFS transporter OS=Citrobacter amalonaticus OX=35703 GN=ILP74_06480 SS=EMBLWGS:MBE0395123 PC=UP000642697:Unassembled WGS sequenceMTQRRELQALIDAAPVSKTQWRVIICCFLVVMLDGFDTAAIGFIAPDIRTHWQLTAGDLAPLFGAGLLGLTAGALLCGPLSDRFGRKRVIELCVALFGALSLISAFSPDLQTLVILRFLTGLGLGGAMPNTITMTSEYLPARRRGALVTLMFCGFTLGSAMGGIVSAQLVPVIGWHGILVLGGVLPLMLFFVLLAVLPESPRWQVRRQLPQATIARTVSAITGERYDNTQFYLQEAAAIAKGSIRQLFVGRQLPITLMLWVVFFMSLLIIYLLSSWMPTLLNHRGIDLQQASWVTAAFQIGGTLGALALGVLMDKHNPFRVLATSYALGAVCIVMIGLSENGLWLMALAIFGTGVGISGSQVGLNALTATLYPTQSRATGVSWSNAVGRCGAIVGSLSGGVMMAMNFSFDTLFFIIAVPAAISAVMLAVLTVVVRQSASVPDAMPHASVVNE>UPI00049F68BF Histidine ABC transporter ATP-binding protein HisP OS=Citrobacter amalonaticus OX=35703 GN=hisP SS=EMBLWGS:MBE0394913 PC=UP000642697:Unassembled WGS sequenceMSENKLNVIDLHKRYGEHEVLKGVSLQANAGDVISIIGSSGSGKSTFLRCINFLEKPSEGSIVVNGQNINLVRDKDGQLKVADKNQLRLLRTRLTMVFQHFNLWSHMTVLENVMEAPIQVLGLSKQEARERATKYLAKVGIDERAQGKYPVHLSGGQQQRVSIARALAMEPEVLLFDEPTSALDPELVGEVLRIMQQLAEEGKTMVVVTHEMGFARHVSSHVIFLHQGKIEEEGNPEQLFGNPQSPRLQQFLKGSLK>UPI00049F68C5 DNA damage-inducible protein I OS=Citrobacter amalonaticus OX=35703 GN=dinI SS=EMBLWGS:MBE0396238 PC=UP000642697:Unassembled WGS sequenceMRIEVTIAKTSPLPAGAIDALAGELSRRITHHFPDNDGNVTVRYAAANNLSVIGATKEDKERISEILQETWESADDWFIKE>UPI00049F69C4 NAAT family transporter YchE OS=Citrobacter amalonaticus OX=35703 GN=ychE SS=EMBLWGS:MBE0395686 PC=UP000642697:Unassembled WGS sequenceMIQTLFDFPVYFKFFIGLFALVNPVGIIPVFISMTSYQTAAARNKTNLTANLSVAIILWTSLFLGDGILQLFGISIDSFRIAGGILVVTIAMSMISGKLGEDKQNKQEKSETAIRESVGVVPLALPLMAGPGAISSTIVWGTRYHSVMYLLGFFVAIALFALCCWGLFRMAPWLVRVLGQTGINVITRIMGLLLMALGIEFIVTGIKAIFPGLLS>UPI00049F69F4 3-deoxy-manno-octulosonate cytidylyltransferase OS=Citrobacter amalonaticus OX=35703 GN=kdsB SS=EMBLWGS:MBE0396378 PC=UP000642697:Unassembled WGS sequenceMSFVVIIPARYASTRLPGKPLQDINGKPMIVHVLERARESGAERIIVATDHEEVARAVEVAGGEVCMTRVDHQSGTERLAEVVEKCGFSDDTVIVNVQGDEPMIPAIIIRQVAENLAQRQVGMATLAAPIHSAEEAFNPNAVKVVLDAEGYALYFSRATIPWDRDRFAKSRETVGDNFLRHLGIYGYRAGFIRRYVSWEPSPLENIEMLEQLRVLWYGEKIHVAVAKEVPGTGVDTAEDLERVRAEMR>UPI00049F6A4A 7-cyano-7-deazaguanine/7-aminomethyl-7-deazaguanine transporter OS=Citrobacter amalonaticus OX=35703 GN=ILP74_20910 SS=EMBLWGS:MBE0397815 PC=UP000642697:Unassembled WGS sequenceMAPFTQSQRVKALFWLSLFHLLVITSSNYLVQLPVSIFGFHTTWGAFSFPFIFLATDLTVRIFGAPLARRIIFAVMMPALLISYVISSLFYMGSWQGFGALMHFNLFVARIAVASFMAYALGQILDVHVFNRLRQNRRWWMAPTASTLFGNVSDTLAFFFIAFWRSPDAFMAEHWMEIAIVDYCFKVLISILFFLPMYGVLLNMLLKKLADKSEIPAMQTS>UPI00049F6A7B 30S ribosomal protein S7 OS=Citrobacter amalonaticus OX=35703 GN=rpsG SS=EMBLWGS:MBE0397704 PC=UP000642697:Unassembled WGS sequenceMPRRRVIGQRKILPDPKFGSELLAKFVNILMVDGKKSTAETIVYSALETLAQRSGKSELEAFEVALENVRPTVEVKSRRVGGSTYQVPVEVRPVRRNALAMRWIVEAARKRGDKSMALRLANELSDAAENKGTAVKKREDVHRMAEANKAFAHYRW>UPI00049F6A88 NAD(P)H-dependent glycerol-3-phosphate dehydrogenase OS=Citrobacter amalonaticus OX=35703 GN=gpsA SS=EMBLWGS:MBE0397940 PC=UP000642697:Unassembled WGS sequenceMNQSNASMTVIGAGSYGTALAITLARNGHQVVLWGHDPKHIATLEHDRCNVAFLPDVPFPDTLRLESDLATALAASRDILVVVPSHVFGEVLRQIKPLLRPDARLVWATKGLEAETGRLLQDVAREALGDKIPLAVISGPTFAKELAAGMPTAISLASTDDTFADDLQQLLHCGKSFRVYSNPDFIGVQLGGAVKNVIAIGAGMSDGIGFGANARTALITRGLTEMSRLGAALGADPQTFMGMAGLGDLVLTCTDNQSRNRRFGMMLGQGMDVQGAQDKIGQVVEGYRNTKEVRELAHRFGVEMPITEEIYQVLYCGKNAREAALTLLGRARKDERSSH>UPI00049F6AC9 GTP cyclohydrolase I FolE OS=Citrobacter amalonaticus OX=35703 GN=folE SS=EMBLWGS:MBE0395111 PC=UP000642697:Unassembled WGS sequenceMSSLSKEAALVHEALVARGLETPLRPPVHEMDNETRKRLISGHMTEIMQLLNLDLSDDSLMETPHRIAKMYVDEIFSGLDYANFPKITVIENKMKVDEMVTVRDITLTSTCEHHFVTIDGKATVAYIPKESVIGLSKINRIVQFFAQRPQVQERLTQQILTALQTLLGTNNVAVSIDAVHYCVKARGIRDATSATTTTSLGGLFKSSQNTRQEFLRAVRHHN>UPI00049F6B60 Inner membrane protein YpjD OS=Citrobacter amalonaticus OX=35703 GN=ILP74_03535 SS=EMBLWGS:MBE0394570 PC=UP000642697:Unassembled WGS sequenceMPVFALLALVAYSVSLALIVPALLQKNSGWRRMAILSAVVALVCHAIALEARILPGGDSGQNLSLLNVGSLVSLMICTVMTIVASRNRGWLLLPVVYAFALINLAFATFMPNEFITHLEATPGMMVHIGLSLFSYATLIIAALYALQLAWIDYQLKNKKLAFNNEMPPLMSIERKMFHITQIGVVLLTLTLCTGLFYMHNLFSMENIDKAVLSIIAWFVYIVLLWGHYHEGWRGRRVVWFNVAGAGILTLAYFGSRILQQFVS>UPI00049F6B73 ClpXP protease specificity-enhancing factor OS=Citrobacter amalonaticus OX=35703 GN=sspB SS=EMBLWGS:MBE0397588 PC=UP000642697:Unassembled WGS sequenceMDLSQLTPRRPYLLRAFYEWLLDNQLTPHLVVDVTLPGVHVPMEYARDGQIVLNIAPRAVGNLELANDEVRFNARFGGVPRQVSVPLAAVLAIYARENGAGTMFEPEAAYDEDVASLNDDDATSGAESETVMSVIDGDKPDHDDDNNPDDDPPPPRGGRPALRVVK>UPI00049F6BCE Threonine/serine exporter OS=Citrobacter amalonaticus OX=35703 GN=ILP74_01450 SS=EMBLWGS:MBE0394187 PC=UP000642697:Unassembled WGS sequenceMGIIDFILALMQDMLLSAIPALGFAMVFNVPHRALPWCALLGALGHGSRMVMMTAGFNIEWSTFVASLLVGSIGIQWSRWYLAHPKVFTVAAVIPMFPGISAYTAMISAVKIGHFGYSEPLMITLLTNFLKASSIVGALSIGLSVPGLWLYRKRPRV>UPI00049F6C2B TRNA uridine-5-carboxymethylaminomethyl(34) synthesis GTPase MnmE OS=Citrobacter amalonaticus OX=35703 GN=mnmE SS=EMBLWGS:MBE0398077 PC=UP000642697:Unassembled WGS sequenceMSHNDTIVAQATPPGRGGVGILRISGLKAREVAEAVLGKLPKPRYADYLPFKDADGTALDQGIALWFPGPNSFTGEDVLELQGHGGPVILDLLLKRILTLPGLRIARPGEFSERAFLNDKLDLAQAEAIADLIDASSEQAARSALNSLQGAFSTRVNHLVEALTHLRIYVEAAIDFPDEEIDFLSDGKIEAQLNGVIADLDAVRAEARQGSLLREGMKVVIAGRPNAGKSSLLNALAGREAAIVTDIAGTTRDVLREHIHIDGMPLHIIDTAGLREASDEVERIGIERAWQEIEQADRVLFMVDGTTTDAVDPADIWPDFIARLPSKLPITVVRNKADITGEPLGISEVNGHSLVRLSARTGEGVDVLRNHLKQSMGFDTNMEGGFLARRRHLQALADAAEHLQQGKAQLLGAWAGELLAEELRLAQQSLSEITGEFTSDDLLGRIFSSFCIGK>UPI00049F6C30 Threonylcarbamoyl-AMP synthase OS=Citrobacter amalonaticus OX=35703 GN=ILP74_09660 SS=EMBLWGS:MBE0395713 PC=UP000642697:Unassembled WGS sequenceMSQFFYIHPDNPQQRLINQAVEIVRKGGVIVYPTDSGYALGCKIEDKGAMERICRIRQLPDGHNFTLMCRDLSELSTYSFVDNVAFRLIKNNTPGNYTFILKGTKEVPRRLLQEKRKTIGLRVPSNPIALELLQTLGEPMLSTSLMLPGSDFTESDPEEIKDRLEKQVDLIIHGGYLGQQPTTVIDLTDDSPVVLREGVGDVKPFL>UPI00049F6C9D DsrE family protein OS=Citrobacter amalonaticus OX=35703 GN=ILP74_09405 SS=EMBLWGS:MBE0395667 PC=UP000642697:Unassembled WGS sequenceMQKIVIIANGAAYGSESLFNSLRLAIALREQASDLDLRLFLMSDAVTAGLRGQKPTEGYNIQQMLEILTAQNVPVKLCKTCTDGRGITTLPLIDGVEVGTLVELAQWTLAADKVLTF>UPI00049F6CA3 Type 1 fimbrial protein OS=Citrobacter amalonaticus OX=35703 GN=ILP74_12510 SS=EMBLWGS:MBE0396251 PC=UP000642697:Unassembled WGS sequenceMLLSVTRKTSLPLLALLALFSFTAMADAGGQGGVIHFTGQIVEPPCNVSLEQQRLAMSCYNTGRTQTRYYSPQELLKAPQHFKQIAAVNLHYLDEKKKLAVMDISYR>UPI00049F6CE5 NADP-specific glutamate dehydrogenase OS=Citrobacter amalonaticus OX=35703 GN=gdhA SS=EMBLWGS:MBE0396125 PC=UP000642697:Unassembled WGS sequenceMDQTCSLESFLNHVQKRDPNQTEFAQAVREVMTTLWPFIEQNPRYREMSLLERLVEPERVIQFRVVWLDDRNQVQVNRAWRVQFNSAIGPYKGGMRFHPSVNLSILKFLGFEQTFKNALTTLPMGGGKGGSDFDPKGKSEGEVMRFCQALMTELYRHLGPDTDVPAGDIGVGGREVGFMAGMMRKLSNNSACVFTGKGLSFGGSLIRPEATGYGLVYFTEAMLKRHGLGFEGMRVAVSGSGNVAQFAIEKAMEFGARVVTASDSSGTVVDESGFTKEKLARLCEIKASRDGRVADYAREFGLTYLEGKQPWSVPVDIALPCATQNELDVEAARVLIANGVKAVAEGANMPTTIEATDLFLEAGVLFAPGKAANAGGVATSGLEMAQNAARLSWKAEKVDARLHHIMLDIHHACVEYGGESKQTNYVRGANISAFVKIADAMFGQGVI>UPI00049F6D5C Maltoporin OS=Citrobacter amalonaticus OX=35703 GN=ILP74_24330 SS=EMBLWGS:MBE0398452 PC=UP000642697:Unassembled WGS sequenceMMITLRKLPLAVAVAAGIMSVQAMAVDFHGYARSGIGWTGSGGEQQCFQATGAQSKYRLGNECETYAELKLGQEVWKEGDKSFYFDTNVAYSVAQQNDWEATDPAFREANVQGKNLIDWLPGSTIWAGKRFYQRHDVHMIDFYYWDISGPGAGIENIDLGFGKLSLAATRSQEAGGSYIFSSNDIYDRYKDTANDVFDVRLAGLETNPDGVLELGVDYGRANKTDGYSYADGATKDGWMFTAEHTQSMLKGYNKFVVQYATDAMTTQGKGIPQGSFTGNNYNPDTEIGVVNNEINNNGSLVRILDHGAISLGDRWDLMYVGMYQDIDRDDNNGSTWYTVGVRPMFKWTPIMSTLMEIGYDNVKSQRTDDTNNQYKITLAQQWQAGDSIWSRPAIRVFATYAKWDEKWGYDNGIAYSDTSARTYSRGDNDEWSFGAQMEIWW>UPI00049F6E16 Restriction endonuclease OS=Citrobacter amalonaticus OX=35703 GN=ILP74_18470 SS=EMBLWGS:MBE0397352 PC=UP000642697:Unassembled WGS sequenceMSAPDSNGLLMAHPYGAVILIVLFLVVMAFLNLRWREKASTRRHRRYRATAGRVLNKLNSLPGDGQRLTYLRKISPYVFEELLLSAFERQGLAVVRNASYSGDGGLDGQVIIDGEYWLIQAKRYSRAVSPAHVEDFDRLLLQSGRRGLFIHTGRTGKMSRTLRTASPRLRIISGQRLLAILAGQDVRQYL>UPI00049F6E39 Malate dehydrogenase OS=Citrobacter amalonaticus OX=35703 GN=mdh SS=EMBLWGS:MBE0397597 PC=UP000642697:Unassembled WGS sequenceMKVAVLGAAGGIGQALALLLKTQLPSGSELSLYDIAPVTPGVAVDLSHIPTAVKIKGFSGEDATPALHGADVVLISAGVARKPGMDRSDLFNVNAGIVKNLVQQIATTCPKACIGIITNPVNTTVAIAAEVLKKAGVYDKNKLFGVTTLDIIRSNTFVAELKGKLPTEVEVPVIGGHSGVTILPLLSQIPGVSFTDQEVADLTKRIQNAGTEVVEAKAGGGSATLSMGQAAARFGLSLVRALQGEKGVVECAYVEGDGQYARFFSQPLLLGKNGVEERQSIGKLSAFEQNALEGMLDTLKKDIQLGEEFVNK>UPI00049F6E44 Uncharacterized protein OS=Citrobacter amalonaticus OX=35703 GN=ILP74_18095 SS=EMBLWGS:MBE0397282 PC=UP000642697:Unassembled WGS sequenceMSYENTNPRRHVAVPDIIKKLEYFSEHDFLLKGLKSEMHFVTFAKQIADSIRRIEYVEKISPSHKKIHELRADPNSEIFDPLRAAYIHLNKGEYDEACWNVFLATHFGKNISTKWQLCRDIYSGLGTEIWSWYKITDDFVGFEKWYNDASQELVRNSTLRQYGNHRKYETLKANSKRSIPKVFRSYLDFIGNTKSHEARFEEAKTIAKTPEDLFSLLYSNMNSVLSFGRTAKFDYLTMLKKISLLDVEPGHLFLRGSTGPVKGCRLLFNGDKLNGDRVDLLDEKLKALAEILDIPYLKMQVLEDSLCNWQKSPSEYLYFGG>UPI00049F6E7A YqcC family protein OS=Citrobacter amalonaticus OX=35703 GN=ILP74_02795 SS=EMBLWGS:MBE0394431 PC=UP000642697:Unassembled WGS sequenceMTTHDRVRQQLHALEALLREHQHWRMDEPEAHLFTSTQPFCMDTMEPIEWLQWVLIPRMHALLDSAQPLPEAFAVAPYYEMALTADHPQREILLEALQALDALFAQDKS>UPI00049F6F20 Succinyl-diaminopimelate desuccinylase OS=Citrobacter amalonaticus OX=35703 GN=dapE SS=EMBLWGS:MBE0394698 PC=UP000642697:Unassembled WGS sequenceMSCPVIELTQQLIRRPSLSPDDAGCQALMIERLRAIGFTVERMDFADTQNFWAWRGQGETLAFAGHTDVVPAGDADRWINPPFEPTIRDGMLFGRGAADMKGSLAAMVVAAERFVAQHPNHKGRLAFLITSDEEASAKNGTVKVVEALMARNERLDYCLVGEPSSTEVVGDVVKNGRRGSLTCNLTIHGVQGHVAYPHLADNPVHRAAPMLNELVNIEWDQGNEFFPATSMQIANVQAGTGSNNVIPGELFVQFNFRFSTELTDEMIKARVHALLEKHQLRYTVDWWLSGQPFLTDRGKLVDAVVNAIEHYNEIKPQLLTTGGTSDGRFIARMGAQVVELGPVNATIHKINECVNAADLQLLARMYQRIMEQLVA>UPI00049F6F28 Cation/acetate symporter ActP OS=Citrobacter amalonaticus OX=35703 GN=actP SS=EMBLWGS:MBE0393941 PC=UP000642697:Unassembled WGS sequenceMKRVLTALAATLPFAANAADAISGAVQRQPTNWQAIVMFLIFVVFTLGITYWASKRVRSRSDYYTAGGNITGFQNGLAIAGDYMSAASFLGISALVFTSGYDGLIYSLGFLVGWPIILFLIAERLRNLGRYTFADVASYRLKQGPIRILSACGSLVVVALYLIAQMVGAGKLIELLFGLNYHIAVVLVGVLMMMYVLFGGMLATTWVQIIKAVLLLFGASFMAFMVMKHVGFSFNNLFTEAMAVHPKGSAIMSPGGLVKDPISALSLGLGLMFGTAGLPHILMRFFTVSDAREARKSVFYATGFMGYFYILTFIIGFGAIMLVGANPAYKDAAGALIGGNNMAAVHLANAVGGNLFLGFISAVAFATILAVVAGLTLAGASAVSHDLYANVFRKGATEREELRVSKITVLVLGVIAIILGVLFENQNIAFMVGLAFAIAASCNFPIILLSMYWSKLTTRGAMMGGWLGLLTAVVLMVLGPTIWVQILGHEKAIFPYEYPALFSITVAFLGIWFFSATDNSTEGNREREQFRAQFIRSQTGFGVEQGRAH>UPI00049F6FBF Transketolase OS=Citrobacter amalonaticus OX=35703 GN=tkt SS=EMBLWGS:MBE0397247 PC=UP000642697:Unassembled WGS sequenceMSSRKELANAIRALSMDAVQKAKSGHPGAPMGMADIAEVLWRDFLNHNPSNPSWADRDRFVLSNGHGSMLIYSLLHLTGYDLPMSELQNFRQLHSKTPGHPEVGYTAGVETTTGPLGQGIANAVGMAIAEKTLAAQFNRPGHDIVDHYTYAFMGDGCMMEGISHEVCSLAGTLKLGKLVAFYDDNGISIDGHVEGWFTDDTAKRFEAYGWHVVRGVDGHDADAIKRAVEEARAVTDKPSLLMCKTIIGFGSPNKAGTHDSHGAPLGDAEIALTREQLGWKYAPFEIPSEIYAQWDAKEAGQAKESAWNEKFAAYAKAFPQEAAEFTRRMKGEMPSDFDAKANEFIAKLQANPAKIASRKASQNAIEAFGPLLPEFLGGSADLAPSNLTLWSGSKAINEDAAGNYIHYGVREFGMTAIANGISLHGGFLPYTSTFLMFVEYARNAVRMAALMKQRQVMVYTHDSIGLGEDGPTHQPVEQVASLRVTPNMSTWRPCDQVESAVAWKYGVERQDGPTALILSRQNLAQQERTEEQLANIARGGYVLKDCAGQPELIFIATGSEVELAVAAWDKLTAEGVKARVVSMPSTDAFDKQDAAYRESVLPKAVTARVAVEAGIADYWFKYVGLNGAIVGMTTFGESAPAELLFEEFGFTVDNVVAKAKALL>UPI00049F70A2 DeoR/GlpR transcriptional regulator OS=Citrobacter amalonaticus OX=35703 GN=ILP74_09745 SS=EMBLWGS:MBE0395730 PC=UP000642697:Unassembled WGS sequenceMNSRQQKILQMVIDKGQMSVSELAKITGVSEVTIRQDLNTLEKQSFLRRAHGFAVSLDSEDVETRMMTNYTLKRQLAEFAASLVSPGESVFIENGSSNALLARTLAEQKDVTIITVSSYIAHLLKETPCEVILLGGIYQKKSESMVGPLTRQFIQQVHFSKAFIGIDGWQTETGFTGRDMMRADVVNAVLEKGSEAIVLTDSSKFGAVHPYTLGPIERFSRVITDARISASDRALMEQRGLTVNIVDAE>UPI00049F70DC Tail assembly protein OS=Citrobacter amalonaticus OX=35703 GN=ILP74_06205 SS=EMBLWGS:MBE0395072 PC=UP000642697:Unassembled WGS sequenceMSEILTRIELYGVLGKIFGRTHYRLIRTTGEAAYSLTKTINGFEKYLNTSRMRGITYAVYKGKNNIGVDDLGFPVTGEVIKIIPVIIGSKKAGLLQTILGVVLVAVGAIATYFGGGAVGVPLMKFGAALAIGGVIQMLSPQSTGLASKQDAANQASYAFGGVTNTAAQGYPVPLLYGKRRIGGAIISAGIYVEDQQ>UPI00049F7170 Propanediol dehydratase small subunit PduE OS=Citrobacter amalonaticus OX=35703 GN=pduE SS=EMBLWGS:MBE0395283 PC=UP000642697:Unassembled WGS sequenceMNTDAIESMVRDVLSRMNSLQGDTSAPAAASGTTTQTAKVTDYPLASKHPEWVKTATNKTLDEFTLENVLSNKVTAQDMRITPETLRIQAAIAKDAGRDRLAMNFERAAELTAVPDDRILEIYNALRPYRSTKEELLAIADDLENRYQAKICAAFVREAAVLYVERKKLKGDD>UPI00049F717F HTH-type transcriptional regulator CysB OS=Citrobacter amalonaticus OX=35703 GN=cysB SS=EMBLWGS:MBE0395720 PC=UP000642697:Unassembled WGS sequenceMKLQQLRYIVEVVNHNLNVSSTAEGLYTSQPGISKQVRMLEDELGIQIFARSGKHLTQVTPAGQEIIRIAREVLSKVDAIKSVAGEHTWPDKGSLYIATTHTQARYALPNVIKGFIERYPRVSLHMHQGSPTQIAEAVSKGNADFAIATEALHLYDDLVMLPCYHWNRSIVVTPEHPLANKGSVTIEELAQYPLVTYTFGFTGRSELDTAFNRAGLTPRIVFTATDADVIKTYVRLGLGVGVIASMAVDPLSDPDLVRVDAHDVFSHSTTKIGFRRSTFLRSYMYDFIQRFAPHLTRDVVDTAVALRSNEEIEEMFKDIKLPEK>UPI00049F7235 Protein-L-isoaspartate O-methyltransferase OS=Citrobacter amalonaticus OX=35703 GN=pcm SS=EMBLWGS:MBE0394465 PC=UP000642697:Unassembled WGS sequenceMVSRRVQTLLDQLRAQGIRDEQVLNALAAVPREKFIDEAFEHKAWDNIALPIGQGQTISQPYMVARMTELLELTPQSRVLEIGTGSGYQTAILAHLVQQVCSVERIKSLQWQARRRLKQLDLHNVSTRHGDGWQGWQARAPFDAIIVTAAPPEIPTALMTQLDEGGILVLPVGDEHQFLKRVRRRGGEFIIDTVEAVRFVPLVKGELA>UPI00049F7381 NADH-quinone oxidoreductase subunit NuoE OS=Citrobacter amalonaticus OX=35703 GN=nuoE SS=EMBLWGS:MBE0394935 PC=UP000642697:Unassembled WGS sequenceMHENQQPQTEAFELSAAEREAIEHEKHHYEDPRAASIEALKIVQKQRGWVPDGAIHAIADVLGIPASDVEGVATFYSQIFRQPVGRHVIRYCDSVVCHINGYQGIQAALEKKLNIKPGQTTFDGRFTLLPTCCLGNCDKGPNMMIDEDTHAHLTPEGIPELLERYK>UPI00049F744B Gentisate 1,2-dioxygenase OS=Citrobacter amalonaticus OX=35703 GN=gtdA SS=EMBLWGS:MBE0395124 PC=UP000642697:Unassembled WGS sequenceMSDHNQDVKNSRQQFYQHISGQNLTPLWESLHHLVPQTPNPTCAPAYWNYQEIRPLLLESGNLIGAKEAIRRVLVLENPMLRGQSSITSTLYAGLQLIMPGEVAPSHRHNQSALRFIVEGKGAFTAVDGERTQMHTGDFILTPQWQWHDHGNPGDEPVVWLDGLDLPLVNILGCGFAEDYPEDQQPVSRKEGDYLPRYAANMLPLRHQKGNSSPIFNYRYDRSRDALHDLTRLGDADEWDGYKMRYVNPVTGGYPMPSMGTFLQLLPKGFTSRVARTTDSTIYHVVEGAGDVTVGNETFHFSAKDIFVVPTWHGVSFKTTDETVLFSFSDRPVQEALGLFREARY>UPI00049F74C2 Cytochrome o ubiquinol oxidase subunit I OS=Citrobacter amalonaticus OX=35703 GN=cyoB SS=EMBLWGS:MBE0396870 PC=UP000642697:Unassembled WGS sequenceMFGKLTLDAVPFHEPIVMVTIAAIIIGGAALLGLITYFGKWTYLWKEWLTSVDHKRLGIMYVIVAIVMLVRGFADAIMMRSQQALASAGEAGFLPPHHYDQIFTAHGVIMIFFVAMPFVIGLMNLVVPLQIGARDVAFPFLNNLSFWFTVVGVILVNLSLGVGEFAQTGWLAYPPLSGIEYSPSVGVDYWIWAVQLSGIGTTLTGINFFVTIIKMRAPGMTMFKMPVFTWASLCANILIIASFPILTVTVALLTLDRYLGTHFFTNDMGGNMMMYINLIWAWGHPEVYILILPVFGVFSEIAATFSRKRLFGYTSLVWATVCITVLSFIVWLHHFFTMGAGANVNAFFGITTMIIAIPTGVKIFNWLFTMYQGRIVFHSAMLWTIGFIVTFSVGGMTGVLLAVPGADFVLHNSLFLIAHFHNVIIGGVVFGCFAGMTYWWPKAFGFTLNETWGKRAFWFWIIGFFVAFMPLYVLGFMGMTRRLSQQIDPQFHPMLVVAACGAALIAVGILCQFIQMYVSIRDREQNRDLTGDPWGGRTLEWATSSPPPFYNFAVVPHVHERDAFWEMKEKGEAYKQPAHYEEIHMPKNSGAGIVITAFATLFGFAMIWHIWWLAIASFAGMIISWIVKSFDEDVDYYVPVPEIEKLENQHFDEITKAGLKNGN>UPI00049F75FE Bifunctional adenosylcobinamide kinase/adenosylcobinamide-phosphate guanylyltransferase OS=Citrobacter amalonaticus OX=35703 GN=cobU SS=EMBLWGS:MBE0395307 PC=UP000642697:Unassembled WGS sequenceMMILVTGGARSGKSRHAEALIADFPQVLYIATSQIFDDEMAARIQHHRDGRPAHWRTAERWQHLDALITADNAPDEAILLECITTMVTNLLFALGGDSDPDSWDYAALETAIEAEIQTLIAACQRCPARVVLVTNEVGMGIVPENRLARHFRDIAGRVNQRLAAAADEVWLVVSGIGVKIK>UPI00049F763A Re/Si-specific NAD(P)(+) transhydrogenase subunit beta OS=Citrobacter amalonaticus OX=35703 GN=pntB SS=EMBLWGS:MBE0395964 PC=UP000642697:Unassembled WGS sequenceMSGGLVTAAYIVAAILFIFSLAGLSKHETSQQGNNFGIAGMAIALIATIFGPDTGNVAWILVAMIIGGAIGIRLAKKVEMTEMPELVAILHSFVGLAAVLVGFNSYLYHEAGLEPILVNIHLTEVFLGIFIGAVTFTGSVVAFGKLRGKISSKPLMLPNRHKMNLAALVVSFLLLVVFVRTESVGLQVLALLVMTIIALAFGWHLVASIGGADMPVVVSMLNSYSGWAAAAAGFMLSNDLLIVTGALVGSSGAILSYIMCKAMNRSFISVIAGGFGTDGTASSGDEEVGEHREISAEETADMLKNSHTVIITPGYGMAVAQAQYPVAEITEKLRARGIKVRFGIHPVAGRLPGHMNVLLAEAKVPYDIVLEMDEINDDFADTDTVLVIGANDTVNPAAQDDPNSPIAGMPVLEVWKAQNVIVFKRSMNTGYAGVQNPLFFKENTHMLFGDAKASVDAILKAL>UPI00049F7779 Spermidine/putrescine ABC transporter permease PotC OS=Citrobacter amalonaticus OX=35703 GN=potC SS=EMBLWGS:MBE0396177 PC=UP000642697:Unassembled WGS sequenceMIGRLLRGGFMTAIYAYLYIPIIILIVNSFNSSRFGINWQGFTTKWYSLLMNNDSLLQAAQHSLTMAIFSATFATAIGSLTAVALYRYRFRGKPFVSGMLFVVMMSPDIVMAISLLVLFMLLGIQLGFWSLLFSHITFCLPFVVVTVYSRLKGFDVRMLEAAKDLGASEMTILRKIILPLAMPAVAAGWLLSFTLSMDDVVVSSFVTGPGYEILPLKIYSMVKVGVSPEVNALATILLVLSLVLVIASQLIARDKTKVQGTLK>UPI00049F77D4 Hydrogenase 2 operon protein HybA OS=Citrobacter amalonaticus OX=35703 GN=hybA SS=EMBLWGS:MBE0397380 PC=UP000642697:Unassembled WGS sequenceMNRRNFIKAASGGALLLGAAPSISHAAAENRPPIPGSLGMLYDSTLCVGCQACVTKCQDINFPARNPEGEQTWSNNDKLSPYTNNIIQVWRSGTGVNKDQEENGYAYIKKQCMHCVDPNCVSVCPVSALKKDPKTGIVHYNKDVCTGCRYCMVACPYNVPKYDYNNPFGALHKCELCNQKGVERLDKGGLPGCVEVCPAGAVIFGTREELMAEAQKRLALKPGSEYHYPRQTLKTGDTYVHTVPKYYPHLYGEKEGGGTQVLVLTGVPYENLDLPKLDEISTGARSEHVQHTLYKGMMLPLAVLAGLTVLVRRNTKNDHHDGGDDHES>UPI00049F77EB 2-methylcitrate synthase OS=Citrobacter amalonaticus OX=35703 GN=prpC SS=EMBLWGS:MBE0396960 PC=UP000642697:Unassembled WGS sequenceMSDTTILQNNTHVIKPKKSVALSGVPAGNTALCTVGKSGNDLHYRGYDILDLARQCEFEEVAHLLIHGKLPTRDELQAYKSKLKALRGLPANVRTVLEALPAASHPMDVMRTGVSALGCTLPEKEGHTVSGARDIADKLLASLSSILLYWYHYSHNGERIQPETDDDSIGGHFLHLLHGEKPTQSWEKAMHISLVLYAEHEFNASTFTSRVIAGTGSDVYSAIIGAIGALRGPKHGGANEVSLEIQQRYETPDEAEADIRKRVENKEVVIGFGHPVYTIADPRHQVIKRVAKQLSEEGGSLKMYNIADRLEEVMWETKKMFPNLDWFSAVSYNMMGVPTEMFTPLFVIARVTGWAAHIIEQRQDNKIIRPSANYTGPDDREFVPIDQRQ>UPI00049F7859 Argininosuccinate synthase OS=Citrobacter amalonaticus OX=35703 GN=argG SS=EMBLWGS:MBE0397541 PC=UP000642697:Unassembled WGS sequenceMTTILKHLPVGQRIGIAFSGGLDTSAALLWMRQKGAVPYAYTANLGQPDEEDYDEIPRRAMEYGAENARLIDCRKQLVAEGIAAIQCGAFHNTTGGLTYFNTTPLGRAVTGTMLVAAMKEDGVNIWGDGSTYKGNDIERFYRYGLLTNAELQIYKPWLDTDFIDELGGRHEMSEFMIACGFDYKMSVEKAYSTDSNMLGATHEAKDLEFLNSSVKIVNPIMGVKFWDENVKIAAEEVTIRFEQGHPVALNGKTFSDDVEMMLEANRIGGRHGLGMSDQIENRIIEAKSRGIYEAPGMALLHIAYERLLTGIHNEDTIEQYHAHGRQLGRLLYQGRWFDSQALMLRDGLQRWVASQITGEVTLELRRGNDYSILNTVSDNLTYKPERLTMEKGDSVFSPDDRIGQLTMRNLDITDTREKLFGYAQSGLLSASSATGLPQVENLENKAK>UPI00049F78B8 Transcription modulator YdgT OS=Citrobacter amalonaticus OX=35703 GN=ydgT SS=EMBLWGS:MBE0395982 PC=UP000642697:Unassembled WGS sequenceMTVQDYLLKFRKISSLESLEKLFDHLHYTLTDDHDIINMYRAADHRRAELVSGGRLFDVGQVPKSVWHYVQ>UPI00049F7907 Carbonate dehydratase OS=Citrobacter amalonaticus OX=35703 GN=can SS=EMBLWGS:MBE0397089 PC=UP000642697:Unassembled WGS sequenceMKDIDTLISNNALWSKMLVEEDSGFFEKLAQAQKPRFLWIGCSDSRVPAERLTGLEPGELFVHRNVANLVIHTDLNCLSVVQYAVDVLEVEHIIICGHYGCGGVQAAVDNPELGLIDNWLLHIRDIWFKHSSLLGEMPQERRMDTLCELNVMEQVYNLGHSTIMQSAWKRGQKVTIHGWAYGIHDGLLRDLEVTATSRATLEQRYRQGVSNLSQKHVNHK>UPI00049F7958 Phosphate response regulator transcription factor PhoB OS=Citrobacter amalonaticus OX=35703 GN=phoB SS=EMBLWGS:MBE0396916 PC=UP000642697:Unassembled WGS sequenceMARRILVVEDEAPIREMVCFVLEQNGFQPVEAEDYDSAVNQLNEPWPDLILLDWMLPGGSGLQFIKHLKREAMTRDIPVMMLTARGEEEDRVRGLETGADDYITKPFSPKELVARIKAVMRRISPMAVEEVIEMQGLSLDPTSHRVMTGDNPLDMGPTEFKLLHFFMTHPERVYSREQLLNHVWGTNVYVEDRTVDVHIRRLRKALEASGHDRMVQTVRGTGYRFSTRF>UPI00049F79B1 RNA polymerase sigma factor RpoD OS=Citrobacter amalonaticus OX=35703 GN=rpoD SS=EMBLWGS:MBE0397442 PC=UP000642697:Unassembled WGS sequenceMEQNPQSQLKLLVTRGKEQGYLTYAEVNDHLPEDIVDSDQIEDIIQMINDMGIQVMEEAPDADDLLLAENTTSTDEDAEEAAAQVLSSVESEIGRTTDPVRMYMREMGTVELLTREGEIDIAKRIEDGINQVQCSVAEYPEAITYLLEQYDRVEAEEARLSDLITGFVDPNAEEDLAPTATHVGSELSQEDLDDDEDEDEEEDDDSSDDDNSIDPELAREKFGELRTQYEVTRDTIKAKGRSHADAQAEILKLSEVFKQFRLVPKQFDYLVNSMRVMMDRVRTQERLIMKLCVEQCKMPKKNFITLFTGNETSETWFNAAIAMNKPWSEKLHDVADDVHRGLQKLQQIEEETGLTIEQVKDINRRMSIGEAKARRAKKEMVEANLRLVISIAKKYTNRGLQFLDLIQEGNIGLMKAVDKFEYRRGYKFSTYATWWIRQAITRSIADQARTIRIPVHMIETINKLNRISRQMLQEMGREPTPEELAERMLMPEDKIRKVLKIAKEPISMETPIGDDEDSHLGDFIEDTTLELPLDSATTESLRAATHDVLAGLTAREAKVLRMRFGIDMNTDHTLEEVGKQFDVTRERIRQIEAKALRKLRHPSRSEVLRSFLDD>UPI00049F7ADB SDR family oxidoreductase OS=Citrobacter amalonaticus OX=35703 GN=ILP74_24200 SS=EMBLWGS:MBE0398426 PC=UP000642697:Unassembled WGS sequenceMQTWLNLQDKVIIVTGGASGIGLAIVEELLAQGANVQMADIHGGEAKHEGNNDYHFWPTDISSAKEVNHTVDEIIQRFGRIDGLVNNAGVNFPRLLVDEKAPAGKYELNEAAFEKMVNINQKGVFLMSQAVARQMVKQRNGVIVNVSSESGLEGSEGQSCYAATKAALNSFTRSWSKELGKHGIRVVGIAPGILEKTGLRTPEYEEALAWTRNITVDQLREGYSKNAIPIGRSGRLSEVADFVCYLLSERASYITGVTTNIAGGKTRG>UPI00049F7B5B YebC/PmpR family DNA-binding transcriptional regulator OS=Citrobacter amalonaticus OX=35703 GN=ILP74_09235 SS=EMBLWGS:MBE0395634 PC=UP000642697:Unassembled WGS sequenceMGRKWANIVAKKTAKDGATSNVYAKFGVEIYAAAKQGEPDPELNTSLKFVIERAKQAQVPKHVIDKAIDKAKGGGDETFVPGRYEGFGPNGAMVIAETLTSNVNRTIANVRTIFNKKGGNIGAAGSVSYMFDNTGVIVFKGTDPDHIFEILLEAEVDVRDVTEEEGNIVIYTEPTDLHKGIAALKAAGITEFSTTELEMIAQSEVELSPEDLEIFEGLVNALEDDDDVQKVYHNVANL>UPI00049F7B7E Ethanolamine ammonia-lyase subunit EutC OS=Citrobacter amalonaticus OX=35703 GN=eutC SS=EMBLWGS:MBE0394724 PC=UP000642697:Unassembled WGS sequenceMDQKQIEEIVRSVMASMGQTQPQTAAPSQSCDKPQCAAPSTTESCALDLGSAEAKAWIGVENPHRAEVLTELRRSTAARVCTGRAGPRPRTQALLRFLADHSRSKDTVLKEVPEEWVKAQGLLEVRSEISDKNLYLTRPDMGRRLSQEAIEALKSQCVANPDVQVVVSDGLSTDAITANYEEILPPLLSGLKQAGLKVGTPFFVRYGRVKIEDQIGEILGAKVVILLVGERPGLGQSESLSCYAVYSPRVATTVEADRTCISNIHQGGTPPVEAAAVIVDLAKRMLEQKASGINMTR>UPI00049F7B98 Formate-dependent uric acid utilization protein AegA OS=Citrobacter amalonaticus OX=35703 GN=aegA SS=EMBLWGS:MBE0394703 PC=UP000642697:Unassembled WGS sequenceMNRFIMANSQQCLGCHACEVACVLAHNEEQHVLSQRHYQPRITVIRHQHQRSAVTCHHCEDAPCARSCPNGAISHVNDSVQVNQQKCIGCKSCVVACPFGTMQIVLTPVANDQVKATAHKCDLCQGREKGPACVENCPADALQLVTEGSLTRLAKARRLRTARQENQPWHADATATAFPAMSKIEQMHATPARGEPDKLAIDARKVSFDEIYLPFRTAQAEREASRCLKCGEHSICEWTCPLHNHIPQWIELVKAGNIDAAVELSHQTNCLPEITGRVCPQDRLCEGACTVRDEHGAVTIGNIERYISDRALGNGWRPDLSHVQKVDKRVAIIGAGPAGLACADVLARHGVSATVFDRHPEIGGLLTFGIPAFKLDKSLLARRREIFSAMGIHFELNCEVGRDVKMEALLEDYDAVFVGVGTYRSMKADLPNEEAPGVYDALPFLIANTKQVMGLDECPEEPYINTAGLNVVVLGGGDTAMDCVRTALRHGASQVTCAYRRDEANMPGSKKEVKNAREEGAKFEFNVQPVDLELSADGRVCGIRFLRTQLGEPDAQGRRRPVPIAGSEFVMPADAVIMAFGFHPHGMPWLESHGVQVDSWGRIAANVESAYRYQTTNPKIFAGGDAVRGADLVVTAMAEGRHAAQGIIDWLGR>UPI00049F7C8E 3-hydroxybenzoate 6-monooxygenase OS=Citrobacter amalonaticus OX=35703 GN=ILP74_06500 SS=EMBLWGS:MBE0395127 PC=UP000642697:Unassembled WGS sequenceMAKVTSAIIVGGGIGGAATALSLARQGIKVMLLEKAHEIGEIGAGIQLGPNAFSALDSLGVGDVARQRAVFTDHITMMDAVNAEEVVHIETGQAFRDHFGGPYAVIHRVDIHASVWESVLTHPNVEYRTSTNVVDIRQTADDVTVFDEQGNSWTADILVGCDGVKSVVRQSLLGDSPRVTGHVVYRAVIDCADMPEDLRINAPVLWAGPHCHLVHYPLRGGKQYNLVVTFHSRQQEEWGVKDGSKEEVLSYFAGIHPRPRQMLDKPTTWRRWSTADREPVEKWGTERITLVGDAAHPVAQYMAQGACMALEDAVTLGKALEQCDGDAAKAFALYESVRIPRTARIVWSTREMGRVYHAAGVERQVRNLLWKGKSQAEFYRGMEWLYGWKEDNCLLPR>UPI00049F7CAB Bifunctional chorismate mutase/prephenate dehydratase OS=Citrobacter amalonaticus OX=35703 GN=pheA SS=EMBLWGS:MBE0394584 PC=UP000642697:Unassembled WGS sequenceMTSENPLLALRDKISALDAKLLALLAERRGLAVEVGKAKLESHRPVRDIDRERDLLDRLITLGKAHHLDAHYITRLFQLIIEDSVLTQQALLQQHLNKINPHSARVAFLGPKGSYSHLAARQYAARHFEQFIESGCTKFADIFNQVETGQADYAVVPIENTSSGGINDVYDLLQHTSLSIVGEMTVTIDHCVLVSGTTDLDTIETVYSHPQPFQQCSKFLSRYPHWKIEYTESTSAAMEKVAQANSPHVAALGSEAGGVLHGLQVLERIEANQTQNITRFIVLARKAINVSDQVPAKTTLLMATGQQAGALVEALLVMRNHNLIMTKLESRPIHGNPWEEMFYLDIQANLESPQMQKALKELGEITRSMKVLGCYPSENVVPVDPS>UPI00049F7D04 NADH-quinone oxidoreductase subunit NuoF OS=Citrobacter amalonaticus OX=35703 GN=nuoF SS=EMBLWGS:MBE0394936 PC=UP000642697:Unassembled WGS sequenceMKDIIRTPETHPLTWRLRDDKQPVWLDEYRSKNGYEGARKALTGLSPDEIVNQVKDAGLKGRGGAGFSTGLKWSLMPKDESMNIRYLLCNADEMEPGTYKDRLLMEQLPHLLVEGMLISAFALKAYRGYIFLRGEYIEAAVHLRRAIAEATEAGLLGKNIMGTGFDFELFVHTGAGRYICGEETALINSLEGRRANPRSKPPFPATSGAWGKPTCVNNVETLCNVPAILANGVEWYQNISKSKDAGTKLMGFSGRVKNPGLWELPFGTTAREILEDYAGGMRDGLKFKAWQPGGAGTDFLTEAHLDLPMEFESIGKAGSRLGTALAMAVDHEIGMVPLVRNLEEFFARESCGWCTPCRDGLPWSVKILRALERGEGQPGDIETLEQLCRFLGPGKTFCAHAPGAVEPLQSAIKYFREEFEAGIKQSFSNTHAIGGIQPNLLKERW>UPI00049F7D54 Aspartate 1-decarboxylase autocleavage activator PanM OS=Citrobacter amalonaticus OX=35703 GN=panM SS=EMBLWGS:MBE0397802 PC=UP000642697:Unassembled WGS sequenceMKLTIIRLEHFSDQDLIDLGKIWPEYSAASLSVDETHRIYAARFNERLLGAVRVTLSGTQGALDSLRIRDVTRRRGVGKYLVEEVIHDNPNVSSWWMADVGVEDQGVMAAFMQVLGFTAQQNGWEKR>UPI00049F7DAC DUF1971 domain-containing protein OS=Citrobacter amalonaticus OX=35703 GN=ILP74_09050 SS=EMBLWGS:MBE0395599 PC=UP000642697:Unassembled WGS sequenceMLQIPQNHIHTRATPFWNKETAPAGIFERHLDKGTRPGVYPRLSVMQGAVKYLGYADEHSPEAETVMIIEAGQFGVFPPEKWHNIEVMSDDTYFNIDFFVAPEVLMESANQRKVIHTGKK>UPI00049F7E20 Transcriptional regulator GutM OS=Citrobacter amalonaticus OX=35703 GN=gutM SS=EMBLWGS:MBE0394504 PC=UP000642697:Unassembled WGS sequenceMVSALVTVAVIAWCAQLALGYWQISRFNNAFDRLCQQGRVGVGRSGGRFKPRVVVAVAVDDNQRVTDTLFMKGLTVFARPGKIADIQGKHLNELQPDVIFPHDPLSQNALSLALNLKHG>UPI00049F7E93 L-methionine/branched-chain amino acid transporter OS=Citrobacter amalonaticus OX=35703 GN=yjeH SS=EMBLWGS:MBE0394027 PC=UP000642697:Unassembled WGS sequenceMSGLKQELGLAQGIGLLSTSLLGTGVFAVPALAALVAGNNSLWAWPVLIVLVFPVAIVFALLGRHYPSAGGVAHFVGMAFGPHLERVTGWLFLSVIPVGLPAALHIAAGFGQAMFGWHGWQLLLAELGTLALVWFIGSRGASSSANLQTVIAGLIVALIVAIWWAGDLTPAEIPFPAPADIEMSGLFSALSVMFWCFVGLEAFAHLASEFKDPERDFPRALMMGLLLAGSVYWACTVVVLHFDAYGETMAAAASLPEIVVQLFGVQALWVACVIGYLACFASLNIYIQSFARLVWSQAQYKPDHYLARLSSRHIPRNALNAVLGCCVVSTLCIYALEINLDALIVYANGIFIMIYLLCMLAGCRLLKGRYRALAMVGGLLCLLLLAMVGWKSLYALIMLAILWLFLPQRKKVEHHL>UPI00049F7EAE Esterase FrsA OS=Citrobacter amalonaticus OX=35703 GN=frsA SS=EMBLWGS:MBE0396977 PC=UP000642697:Unassembled WGS sequenceMSQANLSEILFKPRFKHPETSTLVRRFNAGTQPPVQSALDGKNIPHWYRMINRLMWIWRGVDPREILDVQARIVMSDAERTDADLYDTVIGYRGGNWIYEWAKQAMDWQQKACQETDPERSGRYWLHASTLYNIAAYPHLKGDDLAEQAQALSNRAYEEAAQRLPGTLREMEFSVPGGSPVTGFLHMPKGDGPFPTVLMCGGLDSMQTDYYTLYERYFAPRGIAMLTLDMPSVGFSSKWKLSQDSSLLHQHVLKALPNVPWVDHTRVAAFGFRFGANVAVRLAYLESPRLKAVACLGPVVHALLSDPLRQGAVPEMYLDVLASRLGMHDASDEALRVELNRYSLKVQGLLGRRCPTPMLSGFWKNDPFSPEEESRLITSSSSDGKLLEIPFNPVYRNFDNALQEITRWIEKRLC>UPI00049F7ECD Two-component system response regulator BasR OS=Citrobacter amalonaticus OX=35703 GN=basR SS=EMBLWGS:MBE0393974 PC=UP000642697:Unassembled WGS sequenceMKILIVEDDTLLLQGLILAAQTEGYACDGVSTARAAEQCLETGHYSLVVLDLGLPDEDGLHFLARIRQKKYTLPVLILTARDTLGDKISGLDVGADDYLVKPFALEELHARIRALLRRHNNQGESELVVGNLTLNMGRRQVWKEGEELILTPKEYALLSRLMLKSGSPVHREILYNDIYNWDNEPSTNTLEVHIHNLRDKVGKSRIRTVRGFGYMLVATATEES>UPI00049F7ED8 Acyl carrier protein OS=Citrobacter amalonaticus OX=35703 GN=ILP74_20950 SS=EMBLWGS:MBE0397823 PC=UP000642697:Unassembled WGS sequenceMQALYLEIKNLIITTLNLDELSTDDIDTEAALFGDGLGLDSIDALELGLAVKNQYGVVLSAESEEMRQHFFSVATLASFINAQRA>UPI00049F7F4D DUF1158 domain-containing protein OS=Citrobacter amalonaticus OX=35703 GN=ILP74_15225 SS=EMBLWGS:MBE0396756 PC=UP000642697:Unassembled WGS sequenceMKHPLESLMTAAGILLMAFLSCLLLPAPSLGLVLAQKLVATFHLMDLNQFYTLLFCLWFLALGAVEYFVIRYVWRRWFSLAH>UPI00049F7F58 Acyl-ACP--UDP-N-acetylglucosamine O-acyltransferase OS=Citrobacter amalonaticus OX=35703 GN=lpxA SS=EMBLWGS:MBE0397045 PC=UP000642697:Unassembled WGS sequenceMIDNSAFVHPTAIVEEGASLGANVHIGPFCLVGPHVEIGEGTVLKSHVVVNGHTKIGRDNEIYQFASIGEVNQDLKYAGEPTRVEIGDRNRIRESVTIHRGTVQGGGLTKVGSDNLLMINAHVAHDCTIGDRCILANNATLAGHVSLDDYVIIGGMTAVHQFCIIGAHVMVGGCSGVAQDVPPYVIAQGNHATPFGVNIEGLKRRGFTREAITAIRNAYKALYRSGKTLEDVKPEIAELAKQYPEVQAFSDFFARSTRGLIR>UPI00049F7FA4 NAD-dependent dihydropyrimidine dehydrogenase subunit PreA OS=Citrobacter amalonaticus OX=35703 GN=preA SS=EMBLWGS:MBE0394799 PC=UP000642697:Unassembled WGS sequenceMLTKDLSITFCGVKFPNPFCLSSSPVGNCYEMCAKAYDSGWGGIVFKTIGFFIANEVSPRFDHLIKEDTGFIGFKNMEQIAEHPLAENLAAIRRLKQDYPDKVLIASIMGENEQQWQDLARLVEEAGADMIECNFSCPQMTSHAMGSDVGQSPELVEKYCQAVKRGSSLPMLAKMTPNIGDMCEVALAAKRGGADGIAAINTVKSITNIDLKRKLGLPVVNGKSSISGYSGKAVKPIALRFIQQLRTHPELKDFPISGIGGIETWEDAAEFLLLGAATLQVTTGIMQYGYRIVEDMISGLSHYLNDQGFDSLDEMVGLANANIVPAEDLDRSYIVYPHINQEKCVGCGRCYISCFDGGHQAMEWNEQTRTPHCNTEKCVGCLLCGHVCPVACISLGEVVFKPGEKAHSVTL>UPI00049F80B6 Dipeptide ABC transporter ATP-binding protein OS=Citrobacter amalonaticus OX=35703 GN=dppD SS=EMBLWGS:MBE0397883 PC=UP000642697:Unassembled WGS sequenceMALLNVDKLSVHFGDVGSEFRAVDRISYSVNQGEVVGIVGESGSGKSVSSLAIMGLIDYPGRVMAENLLFNGQDLKRISEKERRNLVGAEVAMIFQDPMTSLNPCYTVGFQIMEAIKVHQGGNKQTRRQRAIDLLNQVGIPDPASRLDVYPHQLSGGMSQRVMIAMAIACRPKLLIADEPTTALDVTIQAQIIELLLELQQKENMALVLITHDLALVAEAAHKIIVMYAGQVVETGAAHDIFRAPRHPYTQALLRALPEFAQDKARLASLPGVVPGKYDRPLGCLLNPRCPYATDKCRSEEPELALLDGGRQSKCHYPLDDAGRPTL>UPI00049F8176 Glycoside hydrolase family 10 protein OS=Citrobacter amalonaticus OX=35703 GN=ILP74_10690 SS=EMBLWGS:MBE0395911 PC=UP000642697:Unassembled WGS sequenceMDICSRAVCSLSRVKKISTLVVATIFLASCSSKPPVSLVTPPKSTPTSPYKTQKLNVPVRGVWLATVSRLDWPPVASVNVSSATTRIAMQKKALTDKLDNLQSLGINTVFFQVKPDGTALWPSSILPWSDMLTGKIGENPGYDPLQFMLDEAHKRGMRVHAWFNPYRVSTNVKPSTVNELNRTVSLQPASVFVLHRDWIRTAGDRYVLDPGIPEARDWITSIVAEVVKRYPVDGVQFDDYFYAESPGSALNDNLTFRRYGQGFSSKADWRRHNTQLLIEQVSRTIRQINPDVEFGVSPAGVWRNRSHDPAGSDTRGAAAYDESYADTRLWVQQGLLDYIAPQIYWPFSRDAARYDVLAKWWADVVKPTKTRLYIGVALYKVGEPSRNEPDWTVNGGVPELKKQLDLNESMPQISGTILFRENNLNQPQAQQAVSYLRNRWSK>UPI00049F8245 Chemotaxis response regulator protein-glutamate methylesterase OS=Citrobacter amalonaticus OX=35703 GN=ILP74_08615 SS=EMBLWGS:MBE0395514 PC=UP000642697:Unassembled WGS sequenceMSKIRVLSVDDSALMRQIMTEIINSYSDMEMVATAPDPLVARDLIKKYNPDVLTLDVEMPRMDGLDFLEKLMRLRPMPVVMVSSLTGKGSEVTLRALELGAIDFVTKPQLGIREGMLAYSEMIAEKVRTASRARLTAHKPLAAPVTLKAGPLLSSEKLIAIGASTGGTEAIRHVLQPLPLSSPAVIITQHMPPGFTRSFAERLNKLCQITVKEAEDGERVLPGHAYIAPGDKHMELARSGANYQIKIHDGPPVNRHRPSVDVLFHSVAIHAGRNAVGVILTGMGNDGAAGMLAMHQAGAWTIAQNEASCVVFGMPREAINMGGVSEVVDLSQVSQQMLAKISAGQAIRI>UPI00049F82E2 Signal peptidase II OS=Citrobacter amalonaticus OX=35703 GN=lspA SS=EMBLWGS:MBE0397198 PC=UP000642697:Unassembled WGS sequenceMSKPLCSTGLRWLWLVVVVLIIDLGSKYLILQNFALGDTVPLFPSLNLHYARNYGAAFSFLADSGGWQRWFFAGIAIGICVILAVMMYRSSATQKLNNIAYALIIGGALGNLFDRLWHGFVVDMIDFYVGDWHFATFNLADSAICIGAAMIVLEGFLPKKQA>UPI00049F84CE CNNM family magnesium/cobalt transport protein CorC OS=Citrobacter amalonaticus OX=35703 GN=corC SS=EMBLWGS:MBE0396676 PC=UP000642697:Unassembled WGS sequenceMSDDNSHSSDTLNSKKGFFSLLLSQLFHGEPKNRDELLALIRDSGQNDLIDEDTRDMLEGVMDIADQRVRDIMIPRSQMITLKRNQTLDECLDVIIESAHSRFPVISEDKDHIEGILMAKDLLPFMRSDAEAFSMEKVLRQAVVVPESKRVDRMLKEFRSQRYHMAIVIDEFGGVSGLVTIEDILELIVGEIEDEYDEEDDIDFRQLSRHTWTIRALAPIEDFNDAFGTSFSDEEVDTIGGLVMQAFGHLPARGETIDIDGYQFKVAMADSRRIIQVHVRIPDDSPQPKLDE>UPI00049F84E4 Biofilm formation regulator BssR OS=Citrobacter amalonaticus OX=35703 GN=bssR SS=EMBLWGS:MBE0396458 PC=UP000642697:Unassembled WGS sequenceMFVDRLRTDLLNKLINARIDLAAYLQLRMAKGYMSVSENDHLRENFFELNRELHDKSLRLNLHLDQEEWDALHHAEGALAAAAVCLMSGHHDCPNFIAVNAEKLNTCLTTLTLSIQSLQAHSTLEDA>UPI00049F8505 Selenium-dependent hydroxylase accessory protein YqeC OS=Citrobacter amalonaticus OX=35703 GN=yqeC SS=EMBLWGS:MBE0394253 PC=UP000642697:Unassembled WGS sequenceMSIPGHASLFFDLGAQKRPTVISIVGAGGKTSTLFWLAHLFQLSGRRVFITTTTHMFLPDHSWPTLFCREPAGLPYHVLMQPIITCFRSWKAPLGKARGFSPDAIDALARRSECDVVLVEADGARGMPLKAPDEHEPCIPESSCCVIAVMGGHLLGKRVGAKQVHRWSPFAGMTGLTEGSPLSLKALVRLVQHPQGAFKNAPPASRRVWFLNRFSQSENAIDERELIQLLSDGDVQAIWLGNAQETPAITRRFVR>UPI00049F8512 Peptidylprolyl isomerase SurA OS=Citrobacter amalonaticus OX=35703 GN=surA SS=EMBLWGS:MBE0397164 PC=UP000642697:Unassembled WGS sequenceMKNWKTLLLGIAMIANTSFAAPQVVDKVAAVVNNGVVLESDVDGLMQSVKLNANQAGQQLPDDATLRHQILERLIMDQIVLQMGQKMGVKISDEQLDQAIANIAKQNNMTLDQMRSRLAYDGLSYSTYRNQIRKEMTISEVRNNEVRRRVTILPQEVDALAQQVGNQNDASTELNLSHILIPLPENPTSDQVNEAESQARSIVDQARNGSDFGKLAITYSADQQALKGGQMGWGRIQELPGIFAQALSTAKKGDIVGPIRSGVGFHILKVNDMRGQSQSISVTEVHARHILLKPSPIMTDQQARLKLEQIAADIKSGKTTFAAAAKEFSQDPGSANQGGDLGWAAADIFDPAFRDALTRMNKGQMSAPVHSSFGWHLIELLDTRNVDKTDAAQKDRAYRMLMNRKFSEEAATWMQEQRASAYVKILSN>UPI00049F8713 Transcriptional repressor MprA OS=Citrobacter amalonaticus OX=35703 GN=mprA SS=EMBLWGS:MBE0394523 PC=UP000642697:Unassembled WGS sequenceMDSSFTPIEQMLKFRASRHEDFPYQEILLTRLCMHMQGKLLDNRNKMLKAQGINETLFMALITLESQENHSIQPSELSCALGSSRTNATRIADELEKRGWIERHESDNDRRCLHLQLTDKGHAFLREVLPPQHNCLHQLWSALNTAEKEQLEHITRKLLTRLDQMDQEGAILEALS>UPI00049F87FB Class I fumarate hydratase OS=Citrobacter amalonaticus OX=35703 GN=fumB SS=EMBLWGS:MBE0393980 PC=UP000642697:Unassembled WGS sequenceMSNKAFFYQAPFPMGKDETEYYLLTSDYVSVSEFEGESILKVEPQALTLLAQQAFHDASFMLRPEHQQQVASILHDPEASENDKYVALQFLRNSEIAAKGILPTCQDTGTAIIMGKKGQRVWTGGGDEAALSKGVYNTYIEDNLRYSQNAALDMYKEVNTGTNLPAQIDLYTVDGDEYKFLCVAKGGGSANKTYLYQETKALLTPGKLKNFLVEKMRTLGTAACPPYHIAFVIGGTSAESTLKTVKLASTHYYDALPTEGNEHGQAFRDTQLEQELLEEAQKLGLGAQFGGKYFAHDIRVIRLPRHGASCPVGMGVSCSADRNIKAKINREGIWIEKLEHNPGQYIPEALRQAGEGEVVKVDLNRPMKEILAQLSQYPVSTRLSLTGTIIVGRDIAHAKLKERIDSGEGLPQYIKDHPIYYAGPAKTPAGYPSGSLGPTTAGRMDSYVDLLQSHGGSMIMLAKGNRSQQVTDACHKHGGFYLGSIGGPAAVLAQQSIKHLECVEYPELGMEAIWKIEVEDFPAFILVDDKGNDFFQQIVNKQCANCTK>UPI00049F886D Molecular chaperone OsmY OS=Citrobacter amalonaticus OX=35703 GN=osmY SS=EMBLWGS:MBE0394203 PC=UP000642697:Unassembled WGS sequenceMTMTRLKISKTLLAVMLTSAVATGSAYAENTTMDKAQSGVESAGQKVDSSMNKVGNFMDDSAITAKVKAALVDHENIKSTDISVKTEQKVVTLSGFVESQAQAEAAVTVAKGVEGVTSVSDKLHVRDSKSDSVKGYAGDTATTSEIKAKLLADDIVPSRKVKVETTDGVVQLSGTVDSQAQIERAESIAKAVDGVKSVKNDLKAQ>UPI00049F8A85 Peroxiredoxin OsmC OS=Citrobacter amalonaticus OX=35703 GN=osmC SS=EMBLWGS:MBE0395900 PC=UP000642697:Unassembled WGS sequenceMTIHKKGQAHWEGDIKRGKGTVSTESGVLNQQPYGFNTRFEGAKGTNPEELIGAAHAACFSMALSLMLGEAGFTPEAIDTTADVSLDKVDAGFAITKIALHSEVNVPGIDAATFDGIIQKAKAGCPVSQVLKAEITLDYQLKS>UPI00049F8AAE Cyclic-guanylate-specific phosphodiesterase OS=Citrobacter amalonaticus OX=35703 GN=pdeH SS=EMBLWGS:MBE0397866 PC=UP000642697:Unassembled WGS sequenceMINQVIQQRSNSEASVESLQDRRFWLQCERAYTYQPIYRTDGRLLAVELLTVVTHPDNPTQRIAPDRYFAGVAVRHRIDIVKEQLQLLEQKTDFFQRHELLASVNVDGPTLLAMRQQPNMVQMIERMPWLRFELVEHIHLPKESSFASMCEFGPLWLDDFGTGMANFSALNEVRYDYIKVARELFVMLRQTPEGRNLFTMLLQLMNRYCRGVIVEGVETLEEWRDVQRSPAFAAQGYFLSRPVPFACLDEVILSL>UPI00049F8B21 DNA-binding transcriptional regulator CsiR OS=Citrobacter amalonaticus OX=35703 GN=csiR SS=EMBLWGS:MBE0394544 PC=UP000642697:Unassembled WGS sequenceMTAISHPTAIDGYRWLKNDIIRGVYQPDEKLRMSLLTSRYSLGVGPLREALSHLVAERLVTVVNQKGYRVASMSEQELLDIFDARANMEAMLVSLAIERGGDEWEAEILARAHMLSKLEASDASEHLLDEWDLRHQAYHTAIVAGCGSQYLLQMRERLFDLAARYRFIWLRKTVLSVEMLEDKHIQHQTLTEAILARDATRASELMRQHLLTPIPIIQQAMTGKLLAQKS>UPI00049F8BDC ADP-ribosylglycohydrolase family protein OS=Citrobacter amalonaticus OX=35703 GN=ILP74_06640 SS=EMBLWGS:MBE0395153 PC=UP000642697:Unassembled WGS sequenceMKAERILGALYGQALGDAMGMPSELWPRTRVKAHFGWIDRFLPGPKENNAACYFGRAEFTDDTSMALCLADALLERDGEIDPDLIGRNILDWALRFDAFNKNVLGPTSKIALNAIRDGKPVAELENNGVTNGAAMRVSPLGCLLPARDLDAFIEDIALASSPTHKSDLAIAGAVVVAWAISRAIEGDSWAAIVDSLPAIALHAQQKRITTFSASLCARLEMALKIVRNADGAEAASEQLYQVIGAGTSTIESVACAIAMVELAQTDPNRCAILCANLGGDTDTIGAMATAICGALQGIGAINPVWKQELDTVNQLDFNRYATALARLRQRREAS>UPI00049F8CB4 Protein MgtR OS=Citrobacter amalonaticus OX=35703 GN=mgtR SS=EMBLWGS:MBE0393937 PC=UP000642697:Unassembled WGS sequenceMNRSPDTIVALIFFLMGLLVLLLAIWQILF>UPI00049F8D45 Pyruvate dehydrogenase complex transcriptional repressor PdhR OS=Citrobacter amalonaticus OX=35703 GN=pdhR SS=EMBLWGS:MBE0397108 PC=UP000642697:Unassembled WGS sequenceMAYSKIRQPKLSDVIEQQLEFLILEGTLRPGEKLPPERELAKQFDVSRPSLREAIQRLEAKGLLLRRQGGGTFVQSSLWQSFSDPLVELLSDHPESQFDLLETRHALEGIAAYYAALRSTDEDKTRIRELHHAIELAQESGDRDAESDAVLQYQIAVTEAAHNVVLLHLLRCMEPMLAQNVRQNFELLYSRREMLPLVSGHRTRIFEAIIAGKPEEAREASHRHLAFIEEILLDRSREESRRERALRRLEQRKN>UPI00049F8E3A HlyD family efflux transporter periplasmic adaptor subunit OS=Citrobacter amalonaticus OX=35703 GN=ILP74_03475 SS=EMBLWGS:MBE0394559 PC=UP000642697:Unassembled WGS sequenceMKTSQPDATHDALDDSREREFSGASRIIWLTAILCLLLAVWAWFGILDEVSTGTGKVIPSSREQVLQSLDGGILAELMVHEGDQVQAGQVLARMDPTRSESNVGESAARYRASLASSQRLTAEVSDKPLVFSDELNAWPDLLASETRLYTSRRAQLADAQSELKEALTLVNKELAITERLAKSGAASHVEVLRLQRQKSDIGLKLTDLRSQYYVQAREALSKANAEVAMLSAIIKGREDSVTRLTVRSPVRGIVKNIQVTTIGGVIPPNGEMMEIVPVDDHLLIETRLSPRDIAFIHPGQRALVKITAYDYAIYGGLEGVVETISPDTIQDKVKPEIFYYRVFIRTHQDYLQNKLGRRFSIVPGMIATVDIKTGEKSIVDYLIKPFNRAKEALRER>UPI00049F8E58 7-carboxy-7-deazaguanine synthase QueE OS=Citrobacter amalonaticus OX=35703 GN=queE SS=EMBLWGS:MBE0394450 PC=UP000642697:Unassembled WGS sequenceMQYPINEMFQTLQGEGYFTGVPAIFIRLQGCPVGCAWCDTKHTWDKLEDREVSLYSILAKTKESDKWGAASSEDLLAVIGRQGYTARHVVITGGEPCIHDLMPLTDLLEKNGFSCQIETSGTHEVRCTPNTWVTVSPKVNMRGGYDVLSQALERANEIKHPVGRVRDIEALDELLATLSDDKPRVIALQPISQKEDATRLCIDTCIARNWRLSMQTHKYLNIA>UPI00049F8E9C Bifunctional D-glycero-beta-D-manno-heptose-7-phosphate kinase/D-glycero-beta-D-manno-heptose 1-phosphate adenylyltransferase HldE OS=Citrobacter amalonaticus OX=35703 GN=hldE SS=EMBLWGS:MBE0397421 PC=UP000642697:Unassembled WGS sequenceMKVTLPEFERAGVLVVGDVMLDRYWYGPTSRISPEAPVPVVKVDTIEERPGGAANVAMNIASLGANSRLVGLTGIDDAARALSKTLADVNVKCDFVSVPTHPTITKLRVLSRNQQLIRLDFEEGFEGVDPQPLHERINQALSSIGALVLSDYAKGALASVQQMIALARKAGVPVLIDPKGTDFARYRGATLLTPNLSEFEAVAGKCKSEEEIVERGMKLIADFELSALLVTRSEQGMTLLQPGKAPLHMPTQAQEVYDVTGAGDTVIGVLAATLAAGNSLEEACFFANAAAGVVVGKLGTSTVSPIELENAVRGRAETGFGVMTEDELKQAVASARKRGEKVVMTNGVFDILHAGHVSYLANARKLGDRLIVAVNSDASTKRLKGETRPVNPLEQRMIVLAALEAVDWVVSFEEDTPQRLIAGVLPDLLVKGGDYKPEEIAGSEEVWANGGEVLVLNFEDGCSTTNIIKKIQKDSDK>UPI00049F8EBD Ferritin-like domain-containing protein OS=Citrobacter amalonaticus OX=35703 GN=ILP74_09610 SS=EMBLWGS:MBE0395703 PC=UP000642697:Unassembled WGS sequenceMNAIEHYHDWLRDAHAMEKQAESMLESMAGRIDNYPDLRSRIEQHISETKHQITILEEILDRNNISRSVIKDSMSKMAALGQSIGGIFPSDEIVKGSISGYVFEQFEIACYTSLLAAAQKAGDTASIPAIESILNEEKQMADWLIKHIPQTTEQFLLRSETDGVEAKK>UPI00049F8FC3 OsmC family protein OS=Citrobacter amalonaticus OX=35703 GN=ILP74_20425 SS=EMBLWGS:MBE0397720 PC=UP000642697:Unassembled WGS sequenceMQARVKWVEGLTFLGESASGHQILMDGNSGDKAPSPMEMVLMAAGGCSAIDVVSILQKGRQDVTNCEVKLTSERREEAPRLFTHINLHFIVTGNDLKDAAVSRAVDLSAEKYCSVALMLGKAVNITHSYEVIAA>UPI00049F904E Co2+/Mg2+ efflux protein ApaG OS=Citrobacter amalonaticus OX=35703 GN=apaG SS=EMBLWGS:MBE0397166 PC=UP000642697:Unassembled WGS sequenceMINSPRVCIQVQSVYIEAQSSPEDDRYVFAYTVTIRNLGRAPVQLLGRYWLITNGHGRETEVQGEGVVGVQPHIEPGEEYQYTSGAVIETPLGTMQGHYEMIDENGVAFTIDIPVFRLAVSTLIH>UPI00049F9062 UMP kinase OS=Citrobacter amalonaticus OX=35703 GN=pyrH SS=EMBLWGS:MBE0397055 PC=UP000642697:Unassembled WGS sequenceMATNAKPVYKRILLKLSGEALQGSEGFGIDASILDRMAQEIKELVELGIQVGVVIGGGNLFRGAGLAKAGMNRVVGDHMGMLATVMNGLAMRDALHRAYVNARLMSAIPLNGVCDNYSWAEAISLLRNNRVVILSAGTGNPFFTTDSAACLRGIEIEADVVLKATKVDGVFTADPAKDPAATMYEQLTYNEVLDKELKVMDLAAFTLARDHKLPIRVFNMNKPGALRRVVMGEKEGTLITE>UPI00049F9090 Flagellar basal body L-ring protein FlgH OS=Citrobacter amalonaticus OX=35703 GN=flgH SS=EMBLWGS:MBE0396221 PC=UP000642697:Unassembled WGS sequenceMQKNAAHAYPIMALLVVSLSGCAWIPSTPLVQGATTAQPVPGPTPVANGSIFQSAQPINYGYQPLFEDRRPRNIGDTLTIVLQENVSASKSSSANASRDGKTNFGFDTVPRYLQGLFGNARADVEASGGNTFNGKGGANASNTFSGTLTVTVDQVLVNGNLHVVGEKQIAINQGTEFIRFSGVVNPRTISGSNSVPSTQVADARIEYVGNGYINEAQNMGWLQRFFLNLSPM>UPI00049F911D (2E,6E)-farnesyl diphosphate synthase OS=Citrobacter amalonaticus OX=35703 GN=ispA SS=EMBLWGS:MBE0396884 PC=UP000642697:Unassembled WGS sequenceMDFSQQLQACVEQANQALSRFIAPLPFQNTPVVETMQYGALLGGKRLRPFLVYATGQMFGISLNTLDAPAAAVECIHAYSLIHDDLPAMDDDDLRRGLPTCHVKFGEANAILAGDALQTLAFSIISDAPMPEVADRDRIAMISELASASGIAGMCGGQALDLDAEGKQVPLEALEKIHRHKTGALIRAAVRLGALSAGEKGRNALPILDKYAESIGLAFQVQDDILDVVGDTATLGKRQGADQQLGKSTYPALLGLEQARNKARDLIAEARQSLNQLAAQSLDTSALEALADYIIQRNK>UPI00049F9175 RpoE-regulated lipoprotein OS=Citrobacter amalonaticus OX=35703 GN=ILP74_04405 SS=EMBLWGS:MBE0394732 PC=UP000642697:Unassembled WGS sequenceMKSLRVVLCAMPLVLTGCSTLSSVNWSAANPWNWFGSSTEVTEQGVGALTASTPLNEQAIADALDGDYRLRSGMKTENGNVVRFFEAMNGDKVAMVIHGEQGNISRIDVLDSGIPSDAGVEIGTPFSDLYSKAFGNCQPASHAEQSAVECKAEGSQHISYLFTGEWKGPEGLMPPDDTLKAWKVSKIIWRR>UPI00049F9228 Universal stress protein UspB OS=Citrobacter amalonaticus OX=35703 GN=uspB SS=EMBLWGS:MBE0397849 PC=UP000642697:Unassembled WGS sequenceMISTVALFWALCVVCIVNMARYFSSLRALLVVLRGCDPLLYQYVDGGGFFTSHGQPNKQVRLVWYIYAQRYRDHHDDEFIRRCERVRRQFILTSALCGLVVVSLIALLIWH>UPI00049F92AA Translesion error-prone DNA polymerase V subunit UmuC OS=Citrobacter amalonaticus OX=35703 GN=umuC SS=EMBLWGS:MBE0397328 PC=UP000642697:Unassembled WGS sequenceMFALVDVNSFYASCETVFRPDLRGKPVVVLSNNDGCIIARSAAAKSLGLKMGDPWFKVGREAERRGVVAFSSNYSLYADMSDRVMTILQMLAPRVEIYSIDEAFCDLTGVSGLLSLEVFGHQIREQIRRRTHLTVGVGIGPTKTLAKLAQYASKRWPATRGVVDLSNIGRQRKLMALVPVEEVWGIGRRLGKKLQLMGINNALQLSELSPSFIRKQFSVVVERTVRELNGTPCLGLEEFTAPKEQIICSRSFGEKPTDEFSIHQAVCAHAERAAEKLRAEHQFCKRVAVFISTSPFAENESFYKNQAVTELAVPARDSRDIIKAAVRALETIFIPGHRYQRAGVILTDFRSAAVPQLTLFGDLQPHRNSDELMTLIDSINSSGKGSVWFAGQGIKSDATGWKMRRERLSPAFTTRLDDIVRVR>UPI00049F92C5 GntR family transcriptional regulator OS=Citrobacter amalonaticus OX=35703 GN=ILP74_06630 SS=EMBLWGS:MBE0395151 PC=UP000642697:Unassembled WGS sequenceMEQAHTQLIAQLNERISAVDNTPLYIKFAETVKNAVRSGVIAHGNILPGERDLSQLTGVSRITVRKAMQALEEEGVVTRARGYGTQINNIFEYSLKEARGFSQQVVLRGKKPDTLWVNKRVVKCPEEVAQQLAIAAGSDVFLLKRIRYVDEDAVSIEESWVPAHLIHDADEIGISLYDYFRSQHIHPQRTRSRVSAKMPDAEFQSHIQMDSKVPVLVIKQVALDQQQRPIEYSISYCRSDLYVFVCEE>UPI00049F92F6 Flagellar type III secretion system pore protein FliP OS=Citrobacter amalonaticus OX=35703 GN=fliP SS=EMBLWGS:MBE0395383 PC=UP000642697:Unassembled WGS sequenceMRRLLSLTLTGLWLISPAALAQLPGLVSQPLPGGGQSWSLPVQTLVFITSLTFIPAILLMMTSFTRIIIVFGLLRNALGTPSAPPNQVLLGLALFLTFFIMSPVIDKIYVEAYQPFSEEKISMQEALDKGAQPLREFMLRQTREADLALFARLANSGPLQGPEAVPMRILLPAYVTSELKTAFQIGFTIFIPFLIIDLVIASVLMALGMMMVPPATIALPFKLMLFVLVDGWQLLVGSLAQSFYS>UPI00049F93AC Adenosine deaminase OS=Citrobacter amalonaticus OX=35703 GN=add SS=EMBLWGS:MBE0395979 PC=UP000642697:Unassembled WGS sequenceMIDNSLPLTDIHRHLDGNIRAQTILDLGRQFNLPLPAQTLETLIPHVQVTATEPDLVSFLSKLDWGVKVLASLDACRRVAFENIEDAARNGLHYVELRFSPGYMAMTHQLPVAGVVEAVIAGVHEGCKTFGVQAQLIGIMSRTFGEAACLQELEALLAHRDRITALDLAGDELGFPGSLFLSHFNRARDAGWHITVHAGEAAGPESIWQAIRELGAERIGHGVKAVEDPALMDFLAEQRIGIESCLTSNIQTSTVASLSAHPLKTFLEHGVLASLNTDDPAVQGVDIIHEYTVAAPAAGLSREQIRQAQINGLEIAFLNAAEKQALREKVAAA>UPI00049F93B8 Iron export ABC transporter permease subunit FetB OS=Citrobacter amalonaticus OX=35703 GN=fetB SS=EMBLWGS:MBE0396803 PC=UP000642697:Unassembled WGS sequenceMNEHNITNESLALAMMLVVVAMLISHKEKLALEKDILWSVARAVVQLIIVGYVLKYIFAVNHAVLTLVMVLFICFNAAYNAQKRSKYIDKAFVSSFIAITTGAGLTLAVLVLSGSIEFIPMQVIPISGMIAGNAMVAVGLCYNNLGQRFSSEQQQIQEKLSLGATPKMASAGLIRDSIRASLIPTIDSAKTVGLVSLPGMMSGLIFAGIDPVKAIKYQIMVTFMLLSTASLSTIIACYLTYRKFYNSRHQLVVTRLKKS>UPI00049F94A4 Peptidylprolyl isomerase A OS=Citrobacter amalonaticus OX=35703 GN=ppiA SS=EMBLWGS:MBE0397727 PC=UP000642697:Unassembled WGS sequenceMLKSTLAAVAAVFALSALSPAALAAKGDPHVLLTTSAGNIELELNSQKAPVSVQNFVDYVNNGFYNNTTFHRVIPGFMVQGGGFTEQMQQKKPNPPIKNEADNGLRNTRGTIAMARTADKDSATSQFFINVADNAFLDHGQRDFGYAVFGKVVKGMEVADKISQVPTHDVGPYQNVPSKPVVILSAKVLP>UPI00049F94B3 Transcription/translation regulatory transformer protein RfaH OS=Citrobacter amalonaticus OX=35703 GN=rfaH SS=EMBLWGS:MBE0398188 PC=UP000642697:Unassembled WGS sequenceMQSWYLLYCKRGQLQRAQEHLERQAVSCLTPMITLEKMVRGKRTAVSEPLFPNYLFVEFDPEVIHTTTINATRGVSHFVRFGASPAIVPATVIHQLSVYKPEGIVDPETPYPGDRVVITEGAFEGLQAIFTEPDGEARSMLLLNLLNKEVKQSVKNTGFRKL>UPI00049F9510 Ethanolamine utilization microcompartment protein EutS OS=Citrobacter amalonaticus OX=35703 GN=eutS SS=EMBLWGS:MBE0394711 PC=UP000642697:Unassembled WGS sequenceMDKERIIQEFVPGKQVTLAHLIAHPGEELAKKIGVPEAGAIGIMTLTPGETAMIAGDLAMKAADVHIGFLDRFSGALVIYGSVGAVEEALLQTVSGLGRLLHFTLCDLTKS>UPI00049F9589 Polyribonucleotide nucleotidyltransferase OS=Citrobacter amalonaticus OX=35703 GN=pnp SS=EMBLWGS:MBE0397534 PC=UP000642697:Unassembled WGS sequenceMLNPIVRKFQYGQHTVTLETGMMARQATAAVMVSMDDTAVFVTVVGQKKAKPGQDFFPLTVNYQERTYAAGRIPGSFFRREGRPSEGETLIARLIDRPVRPLFPEGFVNEVQVIATVVSVNPQVNPDIVAMIGASAALSLSGIPFNGPIGAARVGYINDQYVLNPTQDELKESKLDLVVAGTEAAVLMVESEAELLSEDQMLGAVVFGHEQQQVVIKEINELVKEAGKPRWDWQPEAVNEALNARVAALAESRLSDAYRITDKQERYAQVDVIKSETIATLVAEDESLDANELGEILHAIEKNVVRSRVLAGEPRIDGREKDMIRGLDVRTGVLPRTHGSALFTRGETQALVTATLGTTRDAQSLDELMGERTDNFLFHYNFPPYCVGETGMVGSPKRREIGHGRLAKRGVLAVMPDLDKFPYTVRVVSEITESNGSSSMASVCGASLALMDAGVPIKAAVAGIAMGLVKEGDNYVVLSDILGDEDHLGDMDFKVAGSRDGISALQMDIKIEGITKEIMQVALNQAKGARLHILGVMEQAINAPRGDISQFAPRIHTIKISPDKIKDVIGKGGSVIRALTEETGTTIEIEDDGTVKIAATDGEKAKYAIRRIEEITAEIEVGRIYNGKVTRIVDFGAFVAIGGGKEGLVHISQIADKRVEKVTDYLQMGQEVPVKVLEVDRQGRVRLSIKEATEQSQPAAAPEAPVSEQGE>UPI00049F9595 PTS N-acetylgalactosamine transporter subunit IIB OS=Citrobacter amalonaticus OX=35703 GN=agaB SS=EMBLWGS:MBE0397512 PC=UP000642697:Unassembled WGS sequenceMSSPNILLTRIDNRLVHGQVGVTWTSTIGANLLVVVDDDVAQDEIQQKLMSITAETYGFGIRFFSIEKTINVIGKAAPHQKIFLICRTPQTVRKLLEGGITLNDVNVGNMHFSEGKKQISSKVYVNEQDLNDLQFIKKHGVNIFIQDVPGDQKEAIPE>UPI00049F95DF N-acetylmuramoyl-L-alanine amidase AmiA OS=Citrobacter amalonaticus OX=35703 GN=amiA SS=EMBLWGS:MBE0394729 PC=UP000642697:Unassembled WGS sequenceMSTFKPLKTLTSRRQVLKAGLAALTLTGMANATAKEAPLKTSNGHSQPATKKSGGKRIVVLDPGHGGIDTGAIGRNGSKEKHVVLAIAKNVRSILRSQGIDCRLTRSGDTFIPLYDRVEIAHKHGADLFMSIHADGFTNPSAAGASVFALSNRGASSAMAKYLSDRENRADEVAGKKTTDKDHLLQQVLFDLVQTDTIKNSLTLGSHILRKIKPVHKLHSRNTEQAAFVVLKSPSIPSVLVETSFITNPEEERLLGTTAFRQKIATAIANGVISYFHWFDNQKAHSKKR>UPI00049F9700 Octaprenyl diphosphate synthase OS=Citrobacter amalonaticus OX=35703 GN=ispB SS=EMBLWGS:MBE0397555 PC=UP000642697:Unassembled WGS sequenceMNLEKINELTAQDMAGVNATILEQLNSDVQLINQLGYYIVSGGGKRIRPMIAVLAARAVGYQGNAHVTIAALIEFIHTATLLHDDVVDESDMRRGKATANAAFGNAASVLVGDFIYTRAFQMMTSLGSLKVLEVMSEAVNVIAEGEVLQLMNVNDPDITEDSYMRVIYSKTARLFEAAAQCSGLLAGCSEAEEKGLQDYGRYLGTAFQLIDDLLDYNADGEQLGKNVGDDLNEGKPTLPLLHAMRNGTPEQAQMIRHAIEQGNGRHLLEPVLEAMNACGSLEWTRQRAEEEADKAIAALQVLPDSQWREALIGLAHIAVQRDR>UPI00049F977F Met regulon transcriptional regulator MetJ OS=Citrobacter amalonaticus OX=35703 GN=metJ SS=EMBLWGS:MBE0398339 PC=UP000642697:Unassembled WGS sequenceMAEWSGEYISPYAEHGKKSEQVKKITVSIPLKVLKILTDERTRRQVNNLRHATNSELLCEAFLHAFTGQPLPNDADLRKERSDEIPEAAKEIMRELGIDPETWEY>UPI00049F9917 Basic amino acid antiporter YfcC OS=Citrobacter amalonaticus OX=35703 GN=yfcC SS=EMBLWGS:MBE0394922 PC=UP000642697:Unassembled WGS sequenceMSAVTETQPARKWAMPDTLVIIFFVAILTSLATWVVPVGMFDSQEVQYQVDGQTKTRKVVDPHSFRILTNEAGEAQYHPVKFFTTGDESPGLMNFPFEGLTSGSKFGTAVGIIMFMLVIGGAFGIVMRTGTIDNGILALIRHTKGNEVLFIPVLFILFSLGGAVFGMGEEAVAFAIIIAPLMVRLGYDSITTVLVTYIATQIGFASSWMNPFCVVVAQGIAGVPVLSGSGLRIVVWVISTLIGLTFTLVYASRVKKNPLLSRVHESDRFFREQQDDVVERRFTIGDWLVLLVLTGVMVWVVWGVIVNAWFIPEIASQFFTMGLVIGIIAVVFRLNGMTVNIMASSFTEGARMMIAPALLVGFAKGILLLVGNGEAGDASVLNTLLHSIANGISGLDNAVAAWFMLLFQAVFNFFVTSGSGQAALTMPLLAPLGDLVGVNRQVTVLAFQFGDGFSHIIYPTSASLMATLGVCRVDFRNWLKVGATLLGLLFIMSSVVVIGAQIMGYH>UPI00049F9962 Na(+)/H(+) antiporter NhaB OS=Citrobacter amalonaticus OX=35703 GN=nhaB SS=EMBLWGS:MBE0395619 PC=UP000642697:Unassembled WGS sequenceMELSWGRALWRNFLGQSPDWYKLALLIFLIVNPLIFIINPFVAGWLLVAEFIFTLAMALKCYPLLPGGLLAIEAVIIGMTSAAHVREEVANNLEVLLLLMFMVAGIYFMKQLLLFIFTRLLLSIRSKMLLSLAFCVAAAFLSAFLDALTVVAVVISVAVGFYGIYHRVASSRGDDSDILDDSHIDQHYKTVLEQFRGFLRSLMMHAGVGTALGGVMTMVGEPQNLIIAKAAGWHFGDFFLRMSPVTVPVLICGLLTCVLVEKLRWFGYGETLPEKVRDVLQQFDDQSRHQRSRQDRVKLIVQAIIGVWLVIALALHLAEVGLIGLSVIILATSLTGVTDEHAIGKAFTESLPFTALLTVFFSIVAVIIDQHLFSPIIQYVLQASEHAQLTLFYLFNGLLSSISDNVFVGTIYINEAKAAMESGAISLQQYELLAVAINTGTNLPSVATPNGQAAFLFLLTSALAPLIRLSYGRMVWMALPYTIVLTLVGLLCVEFTLTPMTEWMMQTGWLATLS>UPI00049F9973 DUF1283 family protein OS=Citrobacter amalonaticus OX=35703 GN=ILP74_10895 SS=EMBLWGS:MBE0395948 PC=UP000642697:Unassembled WGS sequenceMNITLRKRLCLTAMLLLGAVVYTATAQAETSRLVIESGDSALSRQQAAMQKEQWDDTRSLRQKVNKRAEKEWDKADAAFDNRDNCEQSANLNAYWEPNTLRCLDRRTGRVIAP>UPI00049F9994 Flagellar hook assembly protein FlgD OS=Citrobacter amalonaticus OX=35703 GN=flgD SS=EMBLWGS:MBE0396225 PC=UP000642697:Unassembled WGS sequenceMSIAVNVNDPTNTGVKSSSNGSSLSGSNAADLQSSFLTLLVAQLKNQDPTNPMQNNELTTQLAQISTVSGIEKLNTTLGSISGQIDNNQSLQASTLIGHGVMIPGTTVLAGKGTEEGATTTTTPFGVELQQPADKVTATITDKDGKVVRTIEIGALKAGVHTFTWDGTLTDGTTAVNGSYNVAIHASNGSTQLVAQPLQFALVQGVIRSNGGNTLDLGTYGTTTLDEVRQII>UPI00049F9B62 DNA polymerase III subunit theta OS=Citrobacter amalonaticus OX=35703 GN=ILP74_08830 SS=EMBLWGS:MBE0395556 PC=UP000642697:Unassembled WGS sequenceMHKNLAQLEQAEMDKVNVDLAAAGVAFKERYNMPVVAEVVEREQPAHLRDWFRERLIAHRLASVSLSRLPYEPKPK>UPI00049F9BB2 HlyD family efflux transporter periplasmic adaptor subunit OS=Citrobacter amalonaticus OX=35703 GN=ILP74_21065 SS=EMBLWGS:MBE0397846 PC=UP000642697:Unassembled WGS sequenceMDNIKRHLTWWVVGALVVIAAGVWWGLRPAGVPDGFAASNGRIEATEVDIATKIAGRIDTILVTEGQFVRQGEVLAKMDTRVLQEQRLEAIAQIKEAESAVAAARALLEQRQSETRAAQSVVKQREAELDSVSKRHVRSRSLSQRGAVSAQQLDDDRAAAESARAALESAKAQVSATKAAIEAARTSIIQAQTRVDAAQATERRIVADIEDSELKAPRDGRVQYRVAEPGEVLAAGGRVLNMVDLSDVYMTFFLPTEQAGLLKIGGEARLVLDAAPDLRIPATISFVASVAQFTPKTVETSDERLKLMFRVKARIPPELLQQHLEYVKTGLPGMAWVRLNEQLPWPDSLAVRLPQ>UPI00049F9C5A Uncharacterized protein OS=Citrobacter amalonaticus OX=35703 GN=ILP74_08465 SS=EMBLWGS:MBE0395486 PC=UP000642697:Unassembled WGS sequenceMHIFSHDQYFIAGLQQILFFTGLDKSPEIVVFDPGGGTVYITNSIECLRADTMDTLTHFTQLRCYSLTRNAPLTEYFYVLDQLKQNKRIPLHTRSLSNRERIIIEYYLAGLGKRAIARNMLLSEGAISNSQLRALRKMNMKNIALFLQVMRNWCVFRAKYSCERSITFLP>UPI00049F9CAB Molecular chaperone Skp OS=Citrobacter amalonaticus OX=35703 GN=skp SS=EMBLWGS:MBE0397048 PC=UP000642697:Unassembled WGS sequenceMKKWLLAAGLGLAMVTSAQAADKIAIVNMGSLFQQVAQKTGVSNTLENEFKGRASELQRMESDLQSKMQRLQSMKAGSDRTKLEKDVMAQRQTFSQKAQAFEQDRARRSNEERGKLVTRIQTAVKSVASSQSIDLVVDANTVAYNSSDVKDITADVLKQVK>UPI00049F9CB3 Galactose/glucose ABC transporter substrate-binding protein MglB OS=Citrobacter amalonaticus OX=35703 GN=mglB SS=EMBLWGS:MBE0395114 PC=UP000642697:Unassembled WGS sequenceMNKKVLTLSAVMASMLFGAAAHAADTRIGVTIYKYDDNFMSVVRKAIEADAKTAPDVQLLMNDSQNDQSKQNDQIDVLLAKGVKALAINLVDPAAAGTVIEKARGQNVPVVFFNKEPSRKALDSYDKAYYVGTDSKESGIIQGDLIAKHWAANQGWDLNKDGQIQFVLLKGEPGHPDAEARTTYVIKELNDKGIKTEQLQLDTAMWDTAMAKDKMDAWLSGPNANKIEVVIANNDAMAMGAVEALKAHNKTSVPVFGVDALPEALALVKSGALAGTVLNDANNQAKATFDLAKNLADGKGAADGTEWKIENKIVRVPYVGVDKDNLAEFTKK>UPI00049F9D0E L(+)-tartrate dehydratase subunit beta OS=Citrobacter amalonaticus OX=35703 GN=ttdB SS=EMBLWGS:MBE0397431 PC=UP000642697:Unassembled WGS sequenceMKKILTTPIKAEDLEDIRVGDVIYLTGTLVTCRDVCHRRLIELKRPIPYDLNGKAIFHAGPIVRKNGEKWEMVSVGPTTSMRMEAFEKEFIEQTGVKLVVGKGGMGPLTEEGCQKFKALHVIFPAGCAVLAATQVEEIEEVHWMELGMPESLWVCRVKEFGPLIVSIDTHGNNLIAENKKQFAERRGPIVDEICEHVHYIK>UPI00049F9DA5 TonB-dependent siderophore receptor OS=Citrobacter amalonaticus OX=35703 GN=ILP74_15135 SS=EMBLWGS:MBE0396739 PC=UP000642697:Unassembled WGS sequenceMNKKIHSLALLVNLGIYGVALPAMAEETADSTAVSHEDTIVVTAAQQNLQAPGVSTITADEIRKNPPARDVSEIIRTMPGVNLTGNSTSGQRGNNRQIDIRGMGPENTLILIDGKPVTSRNSVRLGWRGERDTRGDTSWVPPEMIERIEVLRGPAAARYGNGAAGGVVNIITKKGSNEWHGSWNTYFNAPEHKEEGATKRTNFTLNGPLGGDFSFRLFGNLDKTQADARNINQGHQSERTGTYADTLPAGREGVINKDVNGVVRWDFAPMQSIELEAGYSRQGNLYAGDTQNTNTNQLVKDNYGKETNRLYRQNYSLTWNGGWDNGVTTTNWVQYEHTRNSRTPEGLAGGTEGIFDPKASQKYVDADLNDVTLHSEISMPFDLLVNQNLTLGTEWTQQRMKDMLSNSQTFMGGDIPGSSSTDRSPYSKAEIFSLFAENNMELTDSTMLTPGLRFDHHSIVGDNWSPSLNLSQGLGDDFTLKMGIARAYKAPSLYQTNPNYILYSKGQGCYATGAATGIGCYMMGNDDLKAETSINKEIGLEFKHDGWLAGVTWFRNDYRNKIEAGTVPMSRTSITNKGKTTYTDIYQWENVPKAVVEGLEGTLNVPVSNTVNWTNNITYMLQSKNKETGERLSIIPEYTLNSTLSWQVHQDVSLQSTFTWYGKQEPKKYDYQGKPVTGSDKQSVSPYSIVGLSATWDVTKNVSLTGGVDNVFDKRLWREGNAQTTGSTTDVSYMRGAGAYTYNEPGRTWYMSVNTHF>UPI00049F9EE8 LysR family transcriptional regulator OS=Citrobacter amalonaticus OX=35703 GN=ILP74_00130 SS=EMBLWGS:MBE0393938 PC=UP000642697:Unassembled WGS sequenceMDIRTLRYFVEVVRQQSFTRAAEKLFVTQPTISKMLKNLEDELNCTLLIRDGRKLLLTDTGRVVFERGLAILAEFRQLEAELGDINHLNKGLLRLGIPPMVGMLMAGPIGLFRQRYPGVELKVSEFGGLTVQQAVMNGELDVAMTALPVEEESGLATLPVFSHPLCVLVPRSGQWTTCQSVSPEALAEHPLLIYNEDFALSRQLMQLFSEHDVKPRIAVRSGQWDFLAAMVQAGVGIAILPEPICERLDKNTLRWIPLESELRWQLGMIWREGVYLSHSAKAWLSCCEGFWAEQVPSVAR>UPI00049F9EFF DNA uptake porin HofQ OS=Citrobacter amalonaticus OX=35703 GN=hofQ SS=EMBLWGS:MBE0397741 PC=UP000642697:Unassembled WGS sequenceMKQWILVVLIAILQPVQAGKNQNVTLVVDDVPVTQVLQALAEQERKNLVISPDVSGVVSLHLTDVPWKQALQTVVKSAGLVLRQEGAILHVHSESWQSEEAARQEAEVARRQANLPLENRHIALHYADATELAKAGDKLLSAKGSLTVDKRTNRLLVRDNSPTLALVEQWVAQMDLPIEQVELAAHIVTINEKSLRELGVKWTLAEAEKAGAVGQVTTIASDLSVANATTRVGFNIGRINGRLLDLELSALEQQQQLDIIASPRLLASHLQPASIKQGSEIPYQVSSGESGATSVEFKEAVLGMEVTPTVLPKGRIRLKLRISQNMPGQMLQQADGEVLAIDKQEIETQVEVKSGETLALGGIFSNKNKTGKDSIPLLGDIPWFGQLFRHDGKENERRELVVFITPRLVSTE>UPI00049F9FF2 GntR family transcriptional regulator OS=Citrobacter amalonaticus OX=35703 GN=ILP74_01420 SS=EMBLWGS:MBE0394181 PC=UP000642697:Unassembled WGS sequenceMSRSQNLRHNVINQVIDDMARGHLPSPLPSQSALAEMYNISRTTVRHMLNHLSECGVLTLVGNDHVITRSPEHDDGFACTTASMAEQNRIFEQAFYTMINQRQLRAGETFSELQLARAAGVSPVVVREYLLKFGRYDLIQSEKRGQWSMKKFDQSYAEQLFELREMLETHALQHFLNLPDDDPRWLQAKMLLERHRTLRDSVGSDFRMFSLLDRDFHALLLSAADNIFFNQSLEIISVIFHFHYQWDERDLKQRNIIAIDEHMTILSALICRSDLDALLALRNHLNTAKQSMIRSIRQENE>UPI00049FA060 DUF496 family protein OS=Citrobacter amalonaticus OX=35703 GN=ILP74_07235 SS=EMBLWGS:MBE0395266 PC=UP000642697:Unassembled WGS sequenceMETTKPSFQDVLEFVRLFRRKNKLQREIQDVEKKIRDNQKRVLLLDNLSDYIKPGMSVEAIQGIIASMKSDYEDRVDDYIIKNAEISKERRDISKKLKAMGEMKNGDAKAE>UPI00049FA259 FMN-dependent L-lactate dehydrogenase LldD OS=Citrobacter amalonaticus OX=35703 GN=lldD SS=EMBLWGS:MBE0397937 PC=UP000642697:Unassembled WGS sequenceMIISAASDYRAAAQRILPPFLFHYIDGGAYAEHTLRRNVEDLSDVALRQRVLKNMSDLSLETTLFNEKLSMPVALAPVGLCGMYARRGEVQAAAAADAKGIPFTLSTVSVCPIEEVAPTINRPMWFQLYVLRDRGFMRNALERAKAAGCSTLVFTVDMPTPGARYRDAHSGMSGPNAALRRYWQAVTHPQWAWDVGLNGRPHDLGNISAYLGKPTGLEDYIGWLANNFDPSISWKDLEWIREFWDGPMVIKGILDPEDARDAVRFGADGIVVSNHGGRQLDGVLSSARALPAIADAVKGDIAILADSGIRNGLDVVRMIALGADTVLLGRAYLYALATAGQAGVANLLNLIEKEMKVAMTLTGAKSITDISKDSLVQEINKLPAALAPLSQGDAA>UPI00049FA280 Heavy metal-binding domain-containing protein OS=Citrobacter amalonaticus OX=35703 GN=ILP74_13475 SS=EMBLWGS:MBE0396429 PC=UP000642697:Unassembled WGS sequenceMQFSTTPTLEGQSIVEYCGVVTGEAILGANIFRDFFAGIRDIVGGRSGAYEKELRKAREIAFAELGEQAKALGADAVVGIDIDYETVGKDSSMLMVSVSGTAVKTRR>UPI00049FA29A Uncharacterized protein OS=Citrobacter amalonaticus OX=35703 GN=ILP74_18525 SS=EMBLWGS:MBE0397363 PC=UP000642697:Unassembled WGS sequenceMIRPVFLLTSFFLLSGCSTTDWAAINKQVSDTAANLTKTLGGNSSGESGGMPLMSPARQQAMQSVDKTFSVPVDVDTAAARLKRHYKFISTQELEALRQAANDGDWKAAAEDDAHPVWDAMPGSYYKMGSDWNERDHLDIEIEKNGSGSRLYVVYRSSSSQRLAGSGVTKLMNDVRAVAAGEKR>UPI00049FA2BB Proofreading thioesterase EntH OS=Citrobacter amalonaticus OX=35703 GN=entH SS=EMBLWGS:MBE0396726 PC=UP000642697:Unassembled WGS sequenceMIWKRHFTLDELNATSQNTMVAHLGIIYTRLGDDVLEAEMPVDTRTHQPFGLLHGGASAALAETLGSMAGYLMTRDGQCVVGTELNATHHRAVSQGKVRGVCQPLHLGRQSQSWEIVVFDEQGRRCCTCRLGTAVLG>UPI00049FA2CC Uncharacterized protein OS=Citrobacter amalonaticus OX=35703 GN=ILP74_11945 SS=EMBLWGS:MBE0396145 PC=UP000642697:Unassembled WGS sequenceMNDQMFIETLIISSSFFAIAAVIVISVLILERGS>UPI00049FA405 Transcriptional activator NhaR OS=Citrobacter amalonaticus OX=35703 GN=nhaR SS=EMBLWGS:MBE0397202 PC=UP000642697:Unassembled WGS sequenceMSHINYNHLYYFWHVYKEGSVVGAAEALYLTPQTITGQIKALEERLQGKLFKRKGRGLEPSELGELVFRYADKMFTLSQEMLDIVNYRKESNLLFDVGVADALSKRLVSNVLDAAVVEGEQIHLRCFESTHEMLLEQLSQHKLDMIISDCPIDSTQQEGLFSVKIGECSVSFWCTQPLPEKSFPACLEERRLLIPGRRSMLGRKLLNWINSQGLKVEILGEFDDAALMKAFGATHNAIFVAPTLYAHDFYADDSVVEIGRVENVMEEYHAIFAERMIQHPAVQRICNTNYSALFTPQNK>UPI00049FA421 DUF1738 domain-containing protein OS=Citrobacter amalonaticus OX=35703 GN=ILP74_18110 SS=EMBLWGS:MBE0397285 PC=UP000642697:Unassembled WGS sequenceMKKTTSHKAARRPAKQTDLYRQITDRIVVALENGVAPWRKPWRAASGSGLAGLPLNATTGRHYSGVNVLLLWMSAEEQGFRNNRWMTYRQAQQAGGQVCKGEKATLAVVYKDWTKQAEDREGNRLYDSDGKPLMETVPMLKPLQLFNAEQCEGLPAEVAASPEQPPTVDEDGILCPDVMNRVIRMFNATGVKHRMLPQNRAYYRPLTDEIVMPVAEQFFTEADCWSTLLHELVHSTGHAKRLNREGITSSSRKFGDPVYAFEELIAEMGSAFLCAQLGVFGEVQHDSYVDHWLKVLKSDKKALFRACRHAREASEYLLALPESQAVAA>UPI00049FA43E Small-conductance mechanosensitive channel MscS OS=Citrobacter amalonaticus OX=35703 GN=ILP74_17830 SS=EMBLWGS:MBE0397234 PC=UP000642697:Unassembled WGS sequenceMEDLNVVDSINGAGSWLVRNQELLLSYAVNIVAAVAIVIVGMIVARLVSNTVNRLMVARHIDATVADFLSALVRYGIIAFTLIAALGRVGVQTASVIAVLGAAGLAVGLALQGSLSNLAAGVLLVMFRPFRAGEYVDLGGVAGTVLNVQIFSTTMRTVDGKIVVIPNGKIIAGNIINFSREPVRRNEFIISVAYDSDIDKVKQILTGIIESDDRILKDREMTVRLNELGASSINFVVRVWSNSGDLQNVYWDVLERIKREFDAAGISFPYPQMDVNFKRVKEVTAE>UPI00049FA455 GGDEF domain-containing protein OS=Citrobacter amalonaticus OX=35703 GN=ILP74_10000 SS=EMBLWGS:MBE0395780 PC=UP000642697:Unassembled WGS sequenceMIAHNLNALDLLTQPVWIVSPCTEELVFANRIAREVMQGYTFSQLRKGVYSTHAQNTLPNYITDLRRQRDIVEILTVPRENHQAALTCRLSINDLPGTGEVILFEGIDIPAVQGLKASRSATYQRKKQGFYARFFLTNSAPMLLIDPSRDGLIVDANLAALNFYGYSLEAMCQKHTWEINMLGRQVLPVMHEIARLPGGHKPLNFVHKIADGTTRHVQTYAGPIEIYGDRLMLCIIHDITEQKRLEQELERAALHDALTGLLNRRQFYQLTEQGQMQHLTLAQDYSLLLIDTDRFKSINDLYGHLKGDEVLCELARNLESCARKGDLVFRWGGEEFVLLLPRTPLETALQLAESIRASVAKIGLPGLPRFTVSIGVAHREPNESIDELFKRMDAALYRAKNDGRNRVLAA>UPI00049FA4F4 Bifunctional riboflavin kinase/FAD synthetase OS=Citrobacter amalonaticus OX=35703 GN=ribF SS=EMBLWGS:MBE0397200 PC=UP000642697:Unassembled WGS sequenceMKLIRGIHNLSQAPHGCVLTIGNFDGVHRGHRALLQGLQEEGRRRGLPVMVMIFEPQPLELFATDKAPARLTRLREKLRYLAQCGVDYVLCVRFDRRFAALTAQTFISELLVNRLGVQFLAVGDDFRFGAGREGDFLLLQKAGVEYGFDITSTQTFCEGGVRISSTAVRQALAQDNLALAESLLGHPFTISGRVVHGDELGRTIGFPTANLPLRRQVSPVKGVYAVEVMGLGEKPLPGVANIGTRPTVSGVRQQLEVHLLDVAMDLYGRHIDVVLRKKIRNEQRFASLDELKAQIARDELTARDFFGLSKPA>UPI00049FA551 Cytochrome b N-terminal domain-containing protein OS=Citrobacter amalonaticus OX=35703 GN=ILP74_09300 SS=EMBLWGS:MBE0395647 PC=UP000642697:Unassembled WGS sequenceMIRWRIPFPRSIAGVWLFAVGAILLVMLGIQILTGIVLAMFYVPTTSLAFDSIIHIMRAVSHGELLRNMHAIGASLFFFACYLHIFRGMYYSVYRRPYVTMWMVSVTLYVLLMITAFLGYSLIWGQKSYWAATVITSFARAIPVVGDGLYTLLVGGYAPGTPTLGRFYVLHFILPVVVVVMTIFHVRTVQSAFAHAMKRAFSEKASRRLLFDYRITDADAIKITLFLMLFTWFLFFAPHYLSSADNFIPADPTVTPAIVAPEWYFLPFFSILRCFPNELVGLVAMCGAVLIFYFLPWLDTSRARFRHYSKWVKWGFWLWVLNVCFLGWLGSKALVGPDLTEGIRNEEGWIRAVSQLSTIGYFAWFLLVLPCRRFLEKQD>UPI00049FA6AF Colanic acid polymerase WcaD OS=Citrobacter amalonaticus OX=35703 GN=wcaD SS=EMBLWGS:MBE0395219 PC=UP000642697:Unassembled WGS sequenceMSRSIRICSYLLLPLIYLLVNVKIAQLGESFPITIVTFLPVLLLLYVERISIKKLMIALGIGAGLTAFNYLFGQSLDASKYVTSTMLFVYIVIIIGMVWSIRFKTISPHNYRKILRFFYLVVALVVVLAAAEMAQIILTGGSSLMESISKYLIYSNSYVLNFIKFGGKRTTALYFEPAFFALALISIWLSIKQFGIKTPKTDAMILAGIILSGSFSGVMTFILFYLLEWAFQYLNKDAIKKKLPLAIISLAVFLVGVIFAFPYISTRLGDLGTEGSSSYYRIVGPLVMVGYSLTHIDGVVRFGSLYEYVASFGIFNGADVGKTIDNGLYLLIIYFSWFAVLMTLWYMWKILKMTLNAFGDNRNFRVQLYLFTPVSLFFTGSIFSPEYAFLIVCPFILRKALNMTKV>UPI00049FA75D Rod shape-determining protein MreC OS=Citrobacter amalonaticus OX=35703 GN=mreC SS=EMBLWGS:MBE0397610 PC=UP000642697:Unassembled WGS sequenceMKPIFSRGPSLQIRLILAVLVALGVIIADSRLGTFSQIRTYMDTAVSPFYFISNGPRELLDGVSQTLASRDQLELENRALRQELLLKNSELLMLGQYKQENARLRELLGSPLRQDEQKMVTQVISTVNDPYSDQVVIDKGSVNGVYEGQPVISDKGVVGQVVAVAKLTSRVLLICDATHALPIQVLRNDIRVIAAGNGCTDDLQLEHLPANTDIRVGDVLVTSGLGGRFPEGYPVGVVSSVKLDTQRAYTVIQARPTAGLQRLRYLLLLWGADRNGANPMTPEDVHRVANERLMQMMPQVLPSPDMMGPPSPVPAPATGITSSPAAPPAGATPPPARAPGGQ>UPI00049FA84F Universal stress protein UspC OS=Citrobacter amalonaticus OX=35703 GN=uspC SS=EMBLWGS:MBE0395503 PC=UP000642697:Unassembled WGS sequenceMSYTHILVAVAVTPESQQLLAKAVSIARPVNARISLITLASDPELYNQFAAPMLEDLRAVMHEETTDFLEKLGKEAGYPIAQTFITYGELSEHILDVCRKHDVDLVICGNHNHSFFSRASCSAKSVVSSSLVDVLLVPLEGD>UPI00049FA852 MarC family NAAT transporter OS=Citrobacter amalonaticus OX=35703 GN=ILP74_10770 SS=EMBLWGS:MBE0395924 PC=UP000642697:Unassembled WGS sequenceMMDLFKAIGLGLVVILPLANPLTTVALFLGLAGNMNSAERNHQSLMASVYVFAIMMVAYYAGQLVMNTFGISIPGLRIAGGLIVAFIGFRMLFPQQKAHESPEARSKSEELEDEPTANIAFVPLAMPSTAGPGTIAMIISSASTVRHGTDFPDWVITVAPPLIFALVGVILWGCLRSSGAIMRLVGKGGIEAISRLMGFLLVCMGVQFIINGVLEIIKTY>UPI00049FA95F Arginine--tRNA ligase OS=Citrobacter amalonaticus OX=35703 GN=argS SS=EMBLWGS:MBE0395526 PC=UP000642697:Unassembled WGS sequenceMNIQALLSEKVSQAMIAAGAPADCEPQVRQSAKVQFGDYQANGMMAVAKKLGMAPRQLAEQVLTHLDLNGIASKVEIAGPGFINIFLDAAFLAEHVEQALTSDRLGVTKPAKQTVVVDYSAPNVAKEMHVGHLRSTIIGDAAVRTLEFLGHHVIRANHVGDWGTQFGMLIAYLEKQQQENAGEMALADLEGFYREAKKHYDEDEAFAERARSYVVKLQGGDEYFLQMWRKLVDITMSQNQITYDRLNVTLTRDDVMGESLYNPMLPGIVADLKAKGLAVESEGATVVFLDEYKNKEGEPMGVIIQKKDGGYLYTTTDIACAKYRYETLHADRVLYYIDSRQHQHLMQAWTIVRKAGYVPDAVPLEHHMFGMMLGKDGKPFKTRAGGTVKLADLLDEALERARRLVSEKNPDMPADELEKLANAVGIGAVKYADLSKNRTTDYIFDWDNMLAFEGNTAPYMQYAYTRVLSVFRKSGLDENDLANAKVQLSEDREAQLAARLLQFEETLTVVAREGTPHVMCAYLYDVAGLFSGFYEHCPILSAENEEVRNSRLKLALLTAKTLKLGLDTLGIETVERM>UPI00049FA98D ATP-dependent protease ATP-binding subunit ClpX OS=Citrobacter amalonaticus OX=35703 GN=clpX SS=EMBLWGS:MBE0396863 PC=UP000642697:Unassembled WGS sequenceMTDKRKDGSGKLLYCSFCGKSQHEVRKLIAGPSVYICDECVDLCNDIIREEIKEVAPHRERSALPTPHEIRNHLDDYVIGQEQAKKVLAVAVYNHYKRLRNGDTSNGVELGKSNILLIGPTGSGKTLLAETLARLLDVPFTMADATTLTEAGYVGEDVENIIQKLLQKCDYDVQKAQRGIVYIDEIDKISRKSDNPSITRDVSGEGVQQALLKLIEGTVAAVPPQGGRKHPQQEFLQVDTSKILFICGGAFAGLDKVIANRVETGSGIGFGATVKAKSDKASEGELLSQVEPEDLIKFGLIPEFIGRLPVVATLSELSEEALIQILKEPKNALTKQYQALFNLEGVDLEFRDEALDAIARKAMARKTGARGLRSIVEAALLDTMYDLPSMEDVEKVVIDESVIGGQSKPLLIYGKPEAQQASGE>UPI00049FA9DD Anaerobic ribonucleoside-triphosphate reductase-activating protein OS=Citrobacter amalonaticus OX=35703 GN=nrdG SS=EMBLWGS:MBE0394142 PC=UP000642697:Unassembled WGS sequenceMNYHQYYPVDIVNGPGTRCTLFVSGCVHECPGCYNKSTWRLNSGQPFTKAMEDQIISDLNDTRIHRQGISLSGGDPLHPQNVPDILKLVQRIRAECAGKDIWVWTGYKLDELNAAQMQVVDLINVLVDGKFVQDLKDPALIWRGSSNQVVHHLR>UPI00049FA9F5 Threonine/serine transporter TdcC OS=Citrobacter amalonaticus OX=35703 GN=tdcC SS=EMBLWGS:MBE0397488 PC=UP000642697:Unassembled WGS sequenceMSTSDSIVSSQTKQSSWRKSDTTWTLGLFGTAIGAGVLFFPIRAGFGGLIPILLMLVLAYPIAFYCHRALARLCLSGSNPSGNITETVEEHFGKTGGVVITFLYFFAICPLLWIYGVTITNTFMTFWENQLQLPALNRGFVALFLLLLMAFVIWFGKDLMVKVMSYLVWPFIASLVLISLSLIPYWNSAVIDQVDISNIALTGHDGILVTVWLGISIMVFSFNFSPIVSSFVVSKREEYEKDFGREYTEQKCSQIISRASMLMVAVVMFFAFSCLFTLSPANMADAKAQNIPVLSYLANHFASLSGTKSTFAIVLEYGASIIALVAIFKSFFGHYLGTLEGLNGLVLKFGYKGDKTKVSSGKLNTISMIFIMGSTWIVAYANPNILDLIEAMGAPIIASLLCLLPMYAIRKAPSLAKYRGRLDNVFVTVIGLLTILNIVYKLF>UPI00049FAA5E Uncharacterized protein OS=Citrobacter amalonaticus OX=35703 GN=ILP74_15690 SS=EMBLWGS:MBE0396845 PC=UP000642697:Unassembled WGS sequenceMKQQTLSIASNYGEACELLRSGYVKHVCLSWNVGSDEFFRIASDWCDAGAKIKKEGDNFVISLKGFPVPRQH>UPI00049FAB11 Signal peptidase I OS=Citrobacter amalonaticus OX=35703 GN=lepB SS=EMBLWGS:MBE0394607 PC=UP000642697:Unassembled WGS sequenceMANMFALILVIATLVTGILWCVDKFIFAPKRRERQAAVQAAAGDSLDKATLKKVSPKPGWLETGASVFPVLAIVLVVRSFIYEPFQIPSGSMMPTLLIGDFILVEKFAYGIKDPIYQKTLIETGHPKRGDIVVFKYPEDPRLDYIKRAVGLPGDKVTYDPVAKEVTIQPGCRSGQACENALPVTYSDVQPSDFVQTFARRNGGEATSGFFEIPLSETKENGIRLSERKETLGDVTHRILTVPIAQDQVGMYYQQSGQPLATWIVPPGQYFMMGDNRDNSADSRYWGFVPEANLVGKATAIWMSFDKQEGEWPTGVRLSRIGGIH>UPI00049FAB41 Glycosyltransferase family 4 protein OS=Citrobacter amalonaticus OX=35703 GN=ILP74_21665 SS=EMBLWGS:MBE0397962 PC=UP000642697:Unassembled WGS sequenceMIVAFCLYKYFPFGGLQRDFMRIAQTVAARGHQVRIYTQSWDGECPDAFELIRVPVKSRTNHGRNAEYYAWVQAHLREHPVDRVVGFNKMPGLDVYFAADVCYAEKVEQEKGFFYRLTSRYRHYAAFERATFEQGKPTQLLMLTDTQIADFQKHYQTEAERFHILPPGIYPDRKYSAQIPNAREIVRQKNGISEQQNLLLQVGSDFTRKGVDRSIEALASLPAALRHNTLLYIVGQDKPRKFEALAEKRGVRSNVHFFSGRNDVSELMAAADLLLHPAYQEAAGIVLLEAITAGLPVLTTAVCGYAHYITQANCGDVIEEPWRQDALNDILRKALTQPSLRAAWAENARHYADTQDLYSLPEKAADIITGGLDG>UPI00049FAC3F ABC transporter permease OS=Citrobacter amalonaticus OX=35703 GN=ILP74_12675 SS=EMBLWGS:MBE0396279 PC=UP000642697:Unassembled WGS sequenceMAAKRTMLWLLVWRALRLRFQRVSVVFAALMVGATIVTALSAVWFDINTKMSEELRTFGANFYIGPGHGASMPQRELQTLLDDAPAGLVHGASPWLYGMARTELEKVVIVGVWFESLQKLVPYWQVQGSWIGVSFDDRNAMIGVKLAERLNVQPGDNVTLVDHNQRKNLQIKGIVEAGDATDNMLIVSLDVAQAWLHQPGKISHGLLSVSNDVGQVDRYASRLQSQYPDLEIRPVRKVSASEGQVLDKIKGLMGLVSLVILALSSLCVNTTLMAIVGERAREFALQKALGASNGDIVRQILLETCIIALAAVVCGWLLGYLLAQLLGLTVFNAAISLRLPVLPITLVLSLLVAILAAIVPVRRAVSVEPANVLKGE>UPI00049FAC98 Transcriptional regulator ExuR OS=Citrobacter amalonaticus OX=35703 GN=exuR SS=EMBLWGS:MBE0397469 PC=UP000642697:Unassembled WGS sequenceMEITEPRRLYQQLAADLKERIEQGVYLVGDKLPAERFIADEKNVSRTVVREAIIMLEVEGYVEVRKGSGIHVISNQPRHQQVTDESLEFANYGPFELLQARQLIESNIAEFAATQVTKQDIMKLMTIQEQARNEKCFRDSEWDLQFHIQVALATQNSALAAIVEKMWTQRSHNPYWKKLHEHIDARTVDNWCDDHDQILKALIRKDPHAAKLAMWQHLENTKIMLFNETSDDFEFNADRYLFAENPVVHLDTAANATK>UPI00049FAE25 Inosine/xanthosine triphosphatase OS=Citrobacter amalonaticus OX=35703 GN=yjjX SS=EMBLWGS:MBE0397228 PC=UP000642697:Unassembled WGS sequenceMHHVVSATTNPAKIQAILQAFNEIFGEGSCHIESVDVDSGVPEQPFGSPETRAGARNRVENARRARPDADFWVAIEAGIDEDATFSWVVVENATQRGEARSATLPLPAVILEKVREGEALGPVMSHYTGIDKIGRKEGAIGVFTAGKLTRASVYHQAVILALSPFHNAIYR>UPI00049FAE38 Flagellar protein FlhE OS=Citrobacter amalonaticus OX=35703 GN=flhe SS=EMBLWGS:MBE0395523 PC=UP000642697:Unassembled WGS sequenceMRKLLWLLLFPLVVQAAGEGAWQASSIGITLNHRGVAASSSPLSSSQPVSGLMTLVAWRYELNGPTPAGLRVRLCSQSRCVEIEGQSGTTQAFNNVPAVEPLRFIWEVPGGGRLIPALKVQSNQVIVNYR>UPI00049FAEAB Glycerol dehydratase reactivase beta/small subunit family protein OS=Citrobacter amalonaticus OX=35703 GN=ILP74_07310 SS=EMBLWGS:MBE0395281 PC=UP000642697:Unassembled WGS sequenceMDSSVTTPAIVISTIGDCLSVWKEVLLGIEEEGIPFVIQNQAAGEVVQSAWQAARQSPLLVGIACNEETLVVHYKNLPTSAPLFTLTYRQNSLDRRSTGNNAARLVKGIPFRDLNA>UPI00049FAFAC N-acetyltransferase OS=Citrobacter amalonaticus OX=35703 GN=ILP74_06535 SS=EMBLWGS:MBE0395132 PC=UP000642697:Unassembled WGS sequenceMSVADTLSDNDIAVRDALPDDVEAISSLYAWHVLNGRASFEETPPTIDEMRRRMRTVAQYGLPWLVALYRGVVVGYCYATQYRPRPAYRYTLEESIYVDASMTGRGIGSLLMQTLITRCEEGPWRQMVAVIGDGHNNPGSLRLHKKHGFEIAGQLRSVGYKKGDWRDTLIMQRPLNEGDWTLPE>UPI00049FB058 Transcription termination/antitermination protein NusA OS=Citrobacter amalonaticus OX=35703 GN=nusA SS=EMBLWGS:MBE0397539 PC=UP000642697:Unassembled WGS sequenceMNKEILAVVEAVSNEKALPREKIFEALESALATATKKKYEQEIDVRVEIDRKSGDFDTFRRWVIVEEVTQPTKEITLEAARFEDESLNLGDYVEDQIESVTFDRITTQTAKQVIVQKVREAERAMVVDQFREHEGEIITGVVKKVNRDNISLDLGSNAEAVILREDMLPRENFRPGDRIRGVLYSVRPEARGAQLFVTRSKPEMLIELFRIEVPEIGEEVIEIKAAARDPGSRAKIAVKTNDKRIDPVGACVGMRGARVQAVSTELGGERIDIVLWDDNPAQFVINAMAPADVASIVVDEDKHTMDIAVEAGNLAQAIGRNGQNVRLASQLSGWELNVMTVDDLQAKHQAEAHAAIDTFTKYLDIDEEFATVLVEEGFATLEELAYVPMKELLEIDGLDEPTVEALRERAKNALTTLALAQEESLGDNKPADDLLNLEGLDRDMAFKLAARGVCTLEDLAEQGIDDLADIEGLTDEKAGELIMAARNICWFGDEA>UPI00049FB088 YfcZ/YiiS family protein OS=Citrobacter amalonaticus OX=35703 GN=ILP74_05125 SS=EMBLWGS:MBE0394859 PC=UP000642697:Unassembled WGS sequenceMSKCSADETPVCCCMDVGTIMDNSDCTASYSRVFANRAEAEETLAALTEKARSVESEPCKITSTFTEESDGVRLDIDFVFACEAETLIFQLGLR>UPI00049FB1DA Carboxylating nicotinate-nucleotide diphosphorylase OS=Citrobacter amalonaticus OX=35703 GN=nadC SS=EMBLWGS:MBE0397114 PC=UP000642697:Unassembled WGS sequenceMPPRRYNPDYRRDALLERINLDIPNVVAQALREDLGGQVDASNDITAQLLPENSRSHATVITREDGVFCGKRWVEEVFIQLAGDDVSITWHVEDGDSIKANQPLFELDGPSRVLLTGERTALNFVQTLSGVASEVRKYVDILEGTKTQLLDTRKTLPGLRTALKYAVLCGGGANHRLGLSDAFLIKENHIIASGSVRQAVEKAFWLHPDVPVEVEVENLDELDDALKAGADIIMLDNFETEQMREAVKRTNGQARLEVSGNVTNETLREFAETGVDFISVGALTKHLRALDLSMRFANA>UPI00049FB1F7 DNA-binding transcriptional regulator OxyR OS=Citrobacter amalonaticus OX=35703 GN=oxyR SS=EMBLWGS:MBE0398369 PC=UP000642697:Unassembled WGS sequenceMNIRDLEYLVALAEHRHFRRAADSCHVSQPTLSGQIRKLEDELGVMLLERTSRKVLFTQAGLLLVDQARTVLREVKVLKEMASQQGETMSGPLHIGLIPTVGPYLLPHIIPMLHQTFPKLEMYLHEAQTHQLLAQLDSGKLDCVILALVKESEAFIEVPLFDEPMLLAIYEDHPWANRECVPMSDLAGEKLLMLEDGHCLRDQAMGFCFEAGADEDTHFRATSLETLRNMVAAGSGITLLPALAVPQERKRDGVVYLPCIKPEPRRTVGLVYRPGSPLRSRYEQLAEAIRGAMDGHFDKALKKAV>UPI00049FB306 Two-component system response regulator KdpE OS=Citrobacter amalonaticus OX=35703 GN=kdpE SS=EMBLWGS:MBE0396650 PC=UP000642697:Unassembled WGS sequenceMTNVLIVEDEQAIRRFLRTALEADGLRVYDAETLQRGLLEAATRKPDLIILDLGLPDGDGIDFIRDLRQWSAVPVIVLSARSEESDKIAALDAGADDYLSKPFGIGELQARLRVALRRHSATPSPEPVVRFSDVVVDLAAHLIHRGETEIHLTPIEFRLLAVLLNNAGKVLTQRQLLNQVWGPNAVEHSHYLRIYMGHLRQKLERDPARPRHFITETGIGYRFMP>UPI00049FB327 Uncharacterized protein OS=Citrobacter amalonaticus OX=35703 GN=ILP74_21520 SS=EMBLWGS:MBE0397933 PC=UP000642697:Unassembled WGS sequenceMAKIGENVPLLIDKAVDFMASSQAFKEYLNKMPARDDVPSEVPAENAQMYLQRLNYYRRLYQPVQEKE>UPI00049FB44D PTS N-acetylgalactosamine transporter subunit IIC OS=Citrobacter amalonaticus OX=35703 GN=agaC SS=EMBLWGS:MBE0397513 PC=UP000642697:Unassembled WGS sequenceMNEITLLQGISLAALVFFLGIDFWLEALFLFRPIIVCTLTGAILGDIHIGLITGGLTELAFAGLTPAGGVQPPNPIMAGLMTTVIAWSTGVDAKTAIGLGLPFSLLMQYVILFFYSAFSLFMSRADNCAKEANTRAFSRLNWLTTLIVACSYAIIAFLCTYLAQGAMQALVKAMPAWLTHGFEVAGGILPAVGFGLLLRVMFKAQYIPYLIAGFLFVCYIQVNNLLPVAVLGAGFAVYEFFNAKAKQQAQPQPAAHKNDDEEDYSNGI>UPI00049FB55A GDP-mannose 4,6-dehydratase OS=Citrobacter amalonaticus OX=35703 GN=gmd SS=EMBLWGS:MBE0395222 PC=UP000642697:Unassembled WGS sequenceMSKVALITGVTGQDGSYLAELLLEKGYEVHGIKRRASSFNTERVDHIYQDPHASNPKFHLHYGDLTDSSNLTRILQEVQPDEVYNLGAMSHVAVSFESPEYTADVDAMGTLRLLEAIRFLGLEKKTRFYQASTSELYGLVQEIPQKETTPFYPRSPYAVAKLYAYWITVNYRESYGIYACNGILFNHESPRRGETFVTRKITRAIANIAQGLESCLYLGNMDSLRDWGHAKDYVKMQWMMLQQEQPEDFVIATGVQYSVRQFVEMAAAQLGIKLRFEGEGVEEKGIVVSVTGHDAPGVKPGDVIVAVDPRYFRPAEVETLLGDPSKAHEKLGWKPEITLQEMVSEMVAKDLEAAKKHSLLKSHGYEVAIALES>UPI00049FB56F UDP-N-acetylmuramate--L-alanine ligase OS=Citrobacter amalonaticus OX=35703 GN=murC SS=EMBLWGS:MBE0397130 PC=UP000642697:Unassembled WGS sequenceMNTQQLAKLRSIVPEMRRVRHIHFVGIGGAGMGGIAEVLANEGYQISGSDLAPNPVTQQLTNLGATIYFNHRPENVRDASVVVVSSAISADNPEIVAAHEARIPVIRRAEMLAELMRFRHGIAIAGTHGKTTTTAMVSSIYAEAGLDPTFVNGGLVKAAGVHARLGHSRYLIAEADESDASFLHLQPMVAIVTNIEADHMDTYHGDFENLKQTFINFLHNLPFYGRAVMCVDDPVIRELLPRVGRQTTTYGFSDDADVRVEDYQQVGPQGHFTLLRQGMQDLRVTLNAPGRHNALNAAAAVAVATEEGIADDAILRALESFQGTGRRFDFLGEYPLEPVNGKAGTAMLVDDYGHHPTEVDATIKAARAGWPDKNLVMLFQPHRFTRTRDLYDDFANVLTQVDALLMLDVYAAGEAPIPGADSRSLCRTIRGRGKIDPILVSDPAQVAEMLAPVLTGNDLILVQGAGNIGKIARSLAEIKLKPQIQEDEQHG>UPI00049FB579 PTS cellobiose/arbutin/salicin transporter subunit IIBC OS=Citrobacter amalonaticus OX=35703 GN=ascF SS=EMBLWGS:MBE0394495 PC=UP000642697:Unassembled WGS sequenceMSKNYAALARSVVTALGGVDNITAVTHCMTRLRFVVKDDTLVDSATLKTLSGVLGVVRSDNQCQVIIGNTVSQAYREVVNLLPGDMQPAEPIGKPKLTLRRIGAGILDALIGTMSPLIPAIIGGSMLKLLAMILEMTGVLEKGSSTLTILTVIGDGAFFFLPLMVAASAAIKFKTNMSLAIAIAGVLVHPSFIDLMAKAAQGEHVEFALIPVTAVKYTYTVIPALVMTWCLSYIERWVDRITPAVTKNFLKPMLIVLIAAPLAIVLIGPIGIWIGSAISALVYTIHGYLGWLSVAIMGALWPLLVMTGMHRVFTPTIIQTIAETGKEGMVMPSEIGANLSLGGSSLAVAWKTKNPELRQTALAAAASAIMAGISEPALYGVAVRLKRPLIASLISGFICGAVAGMAGLASHSMAAPGLFTSVQFFDPANPMTIVWVFGVMALAVVLSFVLTLLLGFEDIPVEDAAAQARKNQAAQPSTVKGASV>UPI00049FB58F Molybdate ABC transporter substrate-binding protein OS=Citrobacter amalonaticus OX=35703 GN=modA SS=EMBLWGS:MBE0396571 PC=UP000642697:Unassembled WGS sequenceMARTWLRLFAGATLSLSVAGQTLADEGKITVFAAASLTNAMQDIAAEYKKEKNVDVVSSFASSSTLARQIEAGAPADLFISADQKWMDYAVDKKAIDTATRQTLLGNSLVVVAPKASEQKAFTIDNNTRWTTLLNGGRLAVGDPEHVPAGIYAKEALQKLGAWETLSPKLAPAEDVRGALALVERNEAPLGIVYGSDAVASKGVNVVATFPEDSHKKVEYPIAIVDGHKNATVSAFYDYLKGPQAAEIFKRYGFTTK>UPI00049FB596 MarR family transcriptional regulator OS=Citrobacter amalonaticus OX=35703 GN=ILP74_12660 SS=EMBLWGS:MBE0396276 PC=UP000642697:Unassembled WGS sequenceMAFIWNDESLALLRENAGVLSTQHIAQMLCTNVTVVRNMAYRLKLSLRVSAYSQKRIQQVQALYESDEPLTMKEIAVRTGLTFSTVQYIVYVKLKHKPYATREFIAFETQDAVHYRVQKEFVDTERTRLQQPLDNSRFQELYLKDGTAYCARNIRHEVIISE>UPI00049FB5C7 DNA gyrase inhibitor SbmC OS=Citrobacter amalonaticus OX=35703 GN=sbmC SS=EMBLWGS:MBE0395264 PC=UP000642697:Unassembled WGS sequenceMNYEIRNADKRTVAGFHLVGPWEQTVKQGFDQLMMWVDGKQVVAQEWIAVYFDNPDEVPAEKLRCSTVVSVPADFVIPENSEGVTLSEIDGGQYATAVARVDNNDFATPWYQFFNSLMQDKAYEMACKPCFEVYLNNGCEDGYWDIEMYIAVQPATK>UPI00049FB62D DUF1481 domain-containing protein OS=Citrobacter amalonaticus OX=35703 GN=ILP74_24060 SS=EMBLWGS:MBE0398402 PC=UP000642697:Unassembled WGS sequenceMNSFNEGAASPLSSVWRRTLTLAGVLLLTACSHNASLPPFTASGFADNQGAVRIWRKDASDGVHMLSVFSPWRNGSTTTSEYRWQGDSLSLIELNIYGEPPEHIRVRFDDRGELSFMQREVDGHKQQLSNDQIELYRYRAEQIRQTSDALRQGRVVLRQGRWHAAERTVTTCEGETIKPDLDAWAMSHIERRQSHSSVDVSVAWLEAPEGSQLLLVANSDFCHWQPKEKTF>UPI00049FB664 Aerobic respiration two-component sensor histidine kinase ArcB OS=Citrobacter amalonaticus OX=35703 GN=arcB SS=EMBLWGS:MBE0397577 PC=UP000642697:Unassembled WGS sequenceMKQIRMLAQYYVDLMMKLGLVRFSLLLALALVVLAIVVQMAVTMVLHGRVEQIDVIRSIFFGLLITPWAVYFLSVVVEQLEESRQRLSRLVQKLEEMRERDLKLNVQLKDNISQLNQEIADREKAEAERQSTFEQLKVEIKEREEAQIQLEQQSSFLRSFLDASPDLVFYRNEDKEFSGCNRAMELLTGKSEKQLVHLKPADVYSPEAAEKVIETDEKVFRHNVSLTYEQWLDYPDGRKACFEIRKVPYYDRVGKRHGLMGFGRDITERKRYQDALERASRDKTTFISTISHELRTPLNGIVGLSRILLDTELTAEQEKYLKTIHVSAVTLGNIFNDIIDMDKMERRKVQLDNQPVDFTSFMADLENLSGLQAQQKGLRFVLDPTLPLPHKVVTDGTRLRQILWNLISNAVKFTQQGQVTVRVRYDAGSTLHFEVEDSGIGIPQDEQDKIFAMYYQVKDSHGGKPATGTGIGLAVSRRLAKNMGGDITVASQPGKGSIFTLTVHAPAVAEEVEDAFEDDDLPLPALNVLLVEDIELNVIVARSVLEKLGNSVDVAMTGKAALEMFTPGEYDLVLLDIQLPDMTGLDISRELTQRYAREDLPPLVALTANVLKDKKEYLDAGMDDVLSKPLSVPALTAMIKKYWDTQDEEECTVKSEESSKSQALLDIPMLEQYIELVGPKLITDGLAVFEKMMPGYLSVLESNLTARDKKGVVEEGHKIKGAAGSVGLRHLQQLGQQIQSPDLPAWEDNVGEWIEEMKQEWQHDVAVLKAWVANAGKK>UPI00049FB684 Multidrug efflux MFS transporter permease subunit EmrB OS=Citrobacter amalonaticus OX=35703 GN=emrB SS=EMBLWGS:MBE0394521 PC=UP000642697:Unassembled WGS sequenceMQQQKPLEGAQLVIMTIALSLATFMQVLDSTIANVAIPTIAGNLGSSLSQGTWVITSFGVANAISIPITGWLAKRVGEVKLFMWSTVAFAIASWACGVSSSLNMLIFFRVVQGVVAGPLIPLSQSLLLNNYPPAKRSIALALWSMTVIVAPICGPILGGYISDNYHWGWIFFINVPIGIAVVLMTLQTLRGRETRTEQRRIDAIGLALLVIGIGSLQIMLDRGKELDWFASQEIIILTVVAVVAISFLIVWELTDDNPIVDLSLFKSRNFTIGCLCISLAYMLYFGAIVLLPQLLQEVYGYTATWAGLASAPVGIIPVILSPIIGRFAHKLDMRRLVTFSFIMYAVCFYWRAWTFEPGMDFGASAWPQFIQGFAVACFFMPLTTITLSGLPPERLAAASSLSNFTRTLAGSIGTSITTTMWTNRESMHHAQLTESVNPYNPNAQAMYDQLQGLGMTQQQASGWIAQQITNQGLIISANEIFWMSAGIFLVLLGLVWFAKPPFGAGGGGGGAH>UPI00049FB72B RNA helicase OS=Citrobacter amalonaticus OX=35703 GN=ILP74_18670 SS=EMBLWGS:MBE0397387 PC=UP000642697:Unassembled WGS sequenceMNSIFYSVITLLLLTGGVLLLMREFNKPRNAEELPSETQSIPLTKEEGEDHFSALMNAITPVWYWRVNHEYIDFLHATIKRMTMAQLNDTPGLFDAQRRCSDLNSAVYKYYDTIKKRCLNGEKVPHSDLDVLNLRQCFREFSVEAYPSLVALVWPEYQRPWINPDEV>UPI00049FB749 Protein YaiA OS=Citrobacter amalonaticus OX=35703 GN=yaiA SS=EMBLWGS:MBE0396921 PC=UP000642697:Unassembled WGS sequenceMPTKPPYPREAYIVTIEKGTPGQTVTWYQLRADHPKPDSLISEHPSAQEAMDAKARYEDPDKT>UPI00049FB7BE Arginine ABC transporter ATP-binding protein ArtP OS=Citrobacter amalonaticus OX=35703 GN=artP SS=EMBLWGS:MBE0396431 PC=UP000642697:Unassembled WGS sequenceMSIQLNGINCFYGAHQALFDITLDCPQGETLVLLGPSGAGKSSLLRVLNLLEMPRSGTLNIAGNHFDFRKTPSDKAIRELRQNVGMVFQQYNLWPHLTVQQNLIEAPCRVLGLSKDQALARAEKLLERLRLKPYSDRYPLHLSGGQQQRVAIARALMMEPQVLLFDEPTAALDPEITAQIVSIIRELAETKITQVIVTHEVEVARKTASRVVYMENGHIVEQGNASCFADPQTEAFKNYLSH>UPI00049FB812 YtfJ family protein OS=Citrobacter amalonaticus OX=35703 GN=ILP74_00925 SS=EMBLWGS:MBE0394086 PC=UP000642697:Unassembled WGS sequenceMTLRKILALACLLAPMMASAHSFETGQRVPPVGIADRGELILENDKFSYKSWNSAQLPGKVRILQHIAGRTSAKEKNATLIEAIKAAKLPHDVYQTTTIVNTDDAIPGSGMFVRSSLESNKKLYPWSQFIVDSNGVARKAWQLDEESSAIAVLDKDGRVQWAKDGALTQEEVQQVITLLHKLLNK>UPI00049FB854 Thioredoxin-dependent thiol peroxidase OS=Citrobacter amalonaticus OX=35703 GN=bcp SS=EMBLWGS:MBE0394689 PC=UP000642697:Unassembled WGS sequenceMNPLKAGDIAPKFSLPDQDGEQVNLTDFQGQRVLVYFYPKAMTPGCTVQACGLRDNMDELKKAGVDVLGISTDKPEKLSRFAEKELLNFTLLSDEDHQVCEQFGVWGEKSFMGKTYDGIHRISFLIDADGKIEHVFDDFKTSNHHDVVLNWLKENA>UPI00049FB88D Redox-sensitive transcriptional activator SoxR OS=Citrobacter amalonaticus OX=35703 GN=soxR SS=EMBLWGS:MBE0393931 PC=UP000642697:Unassembled WGS sequenceMEKKLPRIKAMLTPGEVAKRSGVAVSALHFYESKGLISSIRNSGNQRRYRRDVLRYVAIIKIAQRIGIPLATISEALGVLPEGHTLSAKEWKQLSSQWREELDRRIHTLVALRDELDGCIGCGCLSRKDCPLRNPGDRLGEEGTGARLLEDEQN>UPI00049FB9B1 TRNA (Cytosine(32)/uridine(32)-2'-O)-methyltransferase TrmJ OS=Citrobacter amalonaticus OX=35703 GN=trmJ SS=EMBLWGS:MBE0394644 PC=UP000642697:Unassembled WGS sequenceMLQNIRIVLVETSHTGNMGSVARAMKTMGLTNLWLVNPLVKPDSQAIALAAGASDVIGNAQIVDTLDEALAGCSLVVGTSARSRTLPWPMLDPRECGLKSVAEAANTPVALVFGRERVGLTNDELQKCHYHVAIAANPEYSSLNLAMAVQVIAYEVRMAWLATQQNPDAVVHEEAPYPLVDDLERFYGHLEQTLLSTGFIRENHPGQVMNKLRRLFTRARPESQELNILRGMLASIEQQNKDK>UPI00049FB9EE 3-keto-5-aminohexanoate cleavage protein OS=Citrobacter amalonaticus OX=35703 GN=ILP74_14490 SS=EMBLWGS:MBE0396620 PC=UP000642697:Unassembled WGS sequenceMSDTVIISLAPVAADCPRVIPEELAQEILASVEQGAAIVHLHVRDKQGRLTSDTTDFQATIDHVMRHSDLIIQASTGGVSTMSIAERCAPLDCPGVEMASLNVGSVNLGDAVYVNPGPDVEYCSQQITQRSIIPEFEVFEIGMINNILALEEKISFKKPMLFNIVLGHRGSSPATVDALIALRSMIPRDALWGITHFGRKNFDIIAAAVGMGASEVRIGFEDSYYLSAEEKAQHNYQQVAKLATLIRSMDKKVATPDIARQMLAIPPRQ>UPI00049FBA2A Acetolactate synthase 2 small subunit OS=Citrobacter amalonaticus OX=35703 GN=ilvM SS=EMBLWGS:MBE0398121 PC=UP000642697:Unassembled WGS sequenceMMQHQVAVEARFNPETLERVLRVVRHRGFQVCAMNMEAARDAQNINIELTVASPRSVDLLFSQLSKLVDVAHVAICQSTTTSQQIRA>UPI00049FBABB Murein hydrolase activator NlpD OS=Citrobacter amalonaticus OX=35703 GN=nlpD SS=EMBLWGS:MBE0394466 PC=UP000642697:Unassembled WGS sequenceMSAGSPKFTVSRIAALSLVSLWLAGCSNSSNPPAPVSSVNGNAPTNTSSGMLITPPPKMGTTTSAPQTPQIQPVQRPTTQPTHVQPVAEQPVQMENGRIVYNRQYGNIPKGSYSGGSTYTVKKGDTLFYIAWITGNDFRDLAQRNNVQAPYALNVGQTLQVGNASGAPITGGNAITQADAAEQGVVTRPAQNSAIAVASKPTITYSEDSGEQSANKMLPNNKPAGTVVTAPVTAPAVSATESTASSSSTSSPISTWRWPTDGKVIENFGASEGGNKGIDIAGSKGQAIIATADGRVVYAGNALRGYGNLIIIKHNDDYLSAYAHNDTMLVREQQEVKAGQKIATMGSTGTSSTRLHFEIRYKGKSVNPLRYLPQR>UPI00049FBBB0 PTS sugar transporter subunit IIC OS=Citrobacter amalonaticus OX=35703 GN=ILP74_23190 SS=EMBLWGS:MBE0398244 PC=UP000642697:Unassembled WGS sequenceMNSFVEFIVKDLLGQASILIAFIAMLGLILQKKSPGKTAEGTFKTLLGFLIMMAGINIIVATLTFLNDIFTQGFGMKGYITDVAAIAGLANRELGSEVALTLMVIFAVNIIIARLTPLKYIFLTGQALLWMATIGAVIGYKSGLTGLPLILTGGVFGGVMAVLMPALAQPVVRRITGSDDVALGHFCTIGYLVQAAVAKVVGKGSRSTEDLELPDNFKFLQDTYLAMAVVMVPMYLIPALAAGPEYIAQFSGGVNYLMYAFMQSIQFVAGVFVLYSGVRLLLNELVPAFRGIAMRIVPDAKPALDCPVLFPYAPNAVIVGFLATTVGSIIGMLVFPMFGLAMILPGLLTNFFAGGTAGVFGNALGGRRGAMIGGVVHGLFITFLPAILVPMLETYGFTGVTFSDSDVISSGLVLGHAFQNNWLFVALFIVFVAALAWFVNGKSAKPKGENVHESV>UPI00049FBBBC YbjN domain-containing protein OS=Citrobacter amalonaticus OX=35703 GN=ILP74_13540 SS=EMBLWGS:MBE0396442 PC=UP000642697:Unassembled WGS sequenceMTELVVPTLDTLRQWLDDLGMSFFECDTCQALHLPHMQNFDGIYDAKLDLVDNTLLFSAMAEVRPSALLPLAADLSAINASSLTVKAFLDMQDDNLPKLVVCQSLSVMQGVTYAQFEWFVRQSEEQISMVILEAGAHQMLFTAEEDAQKSSVENHFLH>UPI00049FBBE6 TIGR03747 family integrating conjugative element membrane protein OS=Citrobacter amalonaticus OX=35703 GN=ILP74_18455 SS=EMBLWGS:MBE0397349 PC=UP000642697:Unassembled WGS sequenceMAEVKRPPQQQTLPERKHGILYNLLWGWPWALVGVVLSSLLLSLLIEYIGIAFFWPEAGAAHSEAVMNTELGWLSTEFTRSLLLSEPSVTVVRWVSTAYQWAFMDSGFLDWVRQQYAHQMHSDNAVTREINSWSGWLAGYLREYLLATVWISIITLVRVTILVLSVPLFVLVVVVALVEGLGRRDLRRYGAGYESSFVYHHAKKLVKPAAVVPAMLYLSWPTAVYPNLLLLPAAVLLGIAVTVTTASFKKYL>UPI00049FBC60 Nucleoid occlusion factor SlmA OS=Citrobacter amalonaticus OX=35703 GN=slmA SS=EMBLWGS:MBE0397972 PC=UP000642697:Unassembled WGS sequenceMAEKQTAKRNRREEILQSLALMLESSDGSQRITTAKLAASVGVSEAALYRHFPSKTRMFDSLIEFIEDSLITRINLILKDEKDTHARVRLIVLLILGFGERNPGLTRILTGHALMFEQDRLQGRINQLFERIEAQLRQVLREKRMREGEGYVTDETLLASQLLAFCEGMLSRFVRSEFKYRPTDDFDARWPLLAAQLQ>UPI00049FBC88 Flagellar basal body-associated protein FliL OS=Citrobacter amalonaticus OX=35703 GN=fliL SS=EMBLWGS:MBE0395387 PC=UP000642697:Unassembled WGS sequenceMTDSAISKKSKRSIWIPLLVIITLAACATAGYSYWRMQQHPTPAANEPPPPPAPVFFALDTFTVNLGDADRVLYIGVTLRLKDEATRTRLNEYLPEVRSRLLLLFSRQDANALSTEEGKQKLIAAIKETLSPPLVAGQPKQDVTDVLYTAFILR>UPI00049FBC8F Type 1 fimbrial protein OS=Citrobacter amalonaticus OX=35703 GN=ILP74_00955 SS=EMBLWGS:MBE0394092 PC=UP000642697:Unassembled WGS sequenceMNRKLKLISAMVLSSVAIGNAHAALTDDQGTVHFQGKIINPPCEVSTDSKTVNVTFTPMVATTFAAIGSENPQTQDVVINLKSCPADTTINLTFSGATATTSDQLKAMTGSDATGVGIVMYGTGSAASTKVVFDNKPDAAFAQTTPAGEASDLAFNYKAKVVATVTPADIKGGDFTADTTYTISYP>UPI00049FBD5C Citrate lyase acyl carrier protein OS=Citrobacter amalonaticus OX=35703 GN=citD SS=EMBLWGS:MBE0396708 PC=UP000642697:Unassembled WGS sequenceMKINQAAVAGTLESGDVMIRIAPLDTQEIDLQINSSVEKQFGEAIRATILDVLTQYDVRGVQLNVDDKGALDCILRARLEALLARASGIPALPWEDRQ>UPI00049FBD6C MFS transporter OS=Citrobacter amalonaticus OX=35703 GN=ILP74_11500 SS=EMBLWGS:MBE0396059 PC=UP000642697:Unassembled WGS sequenceMKNHYFPTAMGLYLNYLVHGMGVILMSLNMASLEQQWQTNAAGVSVVISSLGIGRLSVLFCAGLLSDRFGRRPFIILGMLCYVTFFFGILTTHNIIIAYAFGVLAGMANSFLDAGTYPSLMEAFPSSPSTANILIKAFVSGGQFLLPIIISLLVWAELWFGWSFIVAAAIMLVNGLFLLRCAFPPHPGRRLAPVTPTHEPHQRPAERHRCAFIDLASYTLYGYISMATFYLISQWLAQYGQFVAGMSYTLSIKLLSIYTCGSLLCVLITAPLVRKTVRSTTLLMLYTFVSFVALLTVCLHPTMYVVIVFAFVIGFSSAGGVVQIGLTLMAARFPHAKGKATGIYYSAGSIATFTIPLVTAHLSQRSIADIMWFDTGIAAVGFLLALFIGYRSRLETKRQLMMSPVAE>UPI00049FBD79 DUF2623 domain-containing protein OS=Citrobacter amalonaticus OX=35703 GN=ILP74_18645 SS=EMBLWGS:MBE0397382 PC=UP000642697:Unassembled WGS sequenceMNNHFGKGLMAGLNATQADSARNVAKFCSDYKRGFVLGFSHRMFEKTGDRQLSAWEAGILTRRYGLDKEMVMDFFRENQSSITIRFFMAGYRLEG>UPI00049FBDF1 Beta-glucosidase BglX OS=Citrobacter amalonaticus OX=35703 GN=bglX SS=EMBLWGS:MBE0395134 PC=UP000642697:Unassembled WGS sequenceMKWLCSVGVAVSLALQPALAEELFGNHPLTPEARDAFVTQLLTKMTVDEKIGQLRLISVGPDNPKEAIREMIKDGQVGAIFNTVTRQDIRKMQDQVMELSRLKIPLFFAYDVLHGQRTVFPISLGLASSFNLDAVKTVGRISAYEAADDGLNMTWAPMVDVSRDPRWGRASEGFGEDTYLTSIMGKTMVEAMQGKSPADRYSVMTSVKHFAAYGAVEGGKEYNTVDMSPQRLFNDYMPPYKAGLDAGSGAVMVALNSLNGTPATSDAWLLKDILRDKWGFKGITVSDHGAIKELIKHGTASDPEDAVRVALKSGINMSMSDEYYSKYLPGLIKSGKVTMAELDDATRHVLNVKYDMGLFNDPYSHLGAKESDPVDTNAESRLHRKEAREVARESLVLLKNRLDTLPLKKSGTIAVVGPLADSKRDVMGSWSAAGVADQSITVLTGIKNALGTKGKVVYAHGANVTNDKDIVTFLNQYEEAVKVDPRSPQEMIDEAVKAAKQSDVVVAVVGEAQGMAHEASSRTDITLPQSQRDLIAALKATGKPLVLVLMNGRPLALVKEDQQADAILETWFAGTEGGNAIADVLFGDYNPSGKLPMSFPRSVGQIPTYYSHLNTGRPYNADKPNKYTSRYFDEANGPLYPFGYGLSYTTFDVSDVTLSAPTLKRDGSVTASVKVTNTGKREGETVIQLYVQDVTASLSRPVKELKGFKKVNLKPGETQTVSFPIDINALKFWNQQMKFDAEPGKFNVFIGVDSARVKQGSFELL>UPI00049FBE57 Dihydrolipoyl dehydrogenase OS=Citrobacter amalonaticus OX=35703 GN=lpdA SS=EMBLWGS:MBE0397105 PC=UP000642697:Unassembled WGS sequenceMSTEIKTQVVVLGAGPAGYSAAFRCADLGLETVIVERYSTLGGVCLNVGCIPSKALLHVAKVIEEAKALAEHGIVFGEPKTDIDKIRTWKEKVITQLTGGLAGMAKGRKVKVVTGLGKFTGANTLEVEGENGKTVINFDNAIIAAGSRPIQLPFIPHEDPRVWDSTDALELKSVPKRLLVMGGGIIGLEMGTVYHALGSEIDVVEMFDQVIPAADKDIVKVFTKRISKKFNLMLETKVTAVEAKEDGIYVSMEGKKAPAEAQRYDAVLVAIGRVPNGKNLDAGKAGVEVDDRGFIRVDKQLRTNVPHIFAIGDIVGQPMLAHKGVHEGHVAAEVIAGMKHYFDPKVIPSIAYTEPEVAWVGLTEKEAKEKGISYETATFPWAASGRAIASDCADGMTKLIFDKETHRVIGGAIVGTNGGELLGEIGLAIEMGCDAEDIALTIHAHPTLHESVGLAAEVFEGSITDLPNAKAKKKK>UPI00049FBE82 3-methyl-2-oxobutanoate hydroxymethyltransferase OS=Citrobacter amalonaticus OX=35703 GN=panB SS=EMBLWGS:MBE0397082 PC=UP000642697:Unassembled WGS sequenceMKPTTLSLLQKCKQEKKRFATITAYDYSFARLFADEGINVMLVGDSLGMTVQGHDSTLPVTVEDIAYHTQAVRRGAPNCLLLADLPFMAYATPEQAFENSATVMRAGANMVKIEGGAWLVETVKMLTERAVPVCGHLGLTPQSVNIFGGYKIQGRGDAGQVLLDDALALEAAGAQLIVLECVPVELAKRVTEALSIPVIGIGAGNVTDGQILVMHDAFGITGGHIPKFAKNFLTQAGDMRAAVRQYIAEVESGVYPGEEHSFH>UPI00049FBE96 Two-domain cob(I)yrinic acid a,c-diamide adenosyltransferase PduO OS=Citrobacter amalonaticus OX=35703 GN=pduO SS=EMBLWGS:MBE0395275 PC=UP000642697:Unassembled WGS sequenceMAIYTRTGDAGTTALFTGQRVSKTHPRVEAYGTLDELNAALSLCVCATHSAQHRALLEAIQLQIFWFSAELASESEQPSADQRYISSEEIAALEAAIDTAMGRVAPLRSFILPGRCEAASRLHFARTLARRAERRLVELAAEVTVRHVLMRYINRLSDCLYALARAEDHDAHQDTIIREVTKRYLAAGHAPVKKESTMSLSFGDLHQLTRAAVERAQVLNVPVVISIVDANGTEAVTWRMPDALLVSSELAPKKAWTAVAMKTATHELASVVQPGAPLYGLESHMQGKVVTFGGGYALWRDGLLIGGLGISGGSVEQDMDIAQAAIAAINVRTHQ>UPI00049FBEA2 YmjA family protein OS=Citrobacter amalonaticus OX=35703 GN=ILP74_17105 SS=EMBLWGS:MBE0397097 PC=UP000642697:Unassembled WGS sequenceMGNEIPLKYYDIVDEYSTESAEPVNASERDPLARYFQLLIARLTNNEEISEEAQQEMAVDAGIDAQRIDDIAEFLNRWGNE>UPI00049FBEA5 Phosphoenolpyruvate synthase OS=Citrobacter amalonaticus OX=35703 GN=ppsA SS=EMBLWGS:MBE0396070 PC=UP000642697:Unassembled WGS sequenceMSNNGSSPLVLWYNQLGMNDVDRVGGKNASLGEMITNLSGMGVSVPNGFATTADAFNQFLDQSGVNQRIYELLDQTDIDDVNALAKAGAQIRQWIIDTPFQPELENAIRDAYAQLSADDQHASFAVRSSATAEDMPDASFAGQQETFLNVQGFDAVLVAVKHVFASLFNDRAISYRVHQGYDHRGVALSAGVQRMVRSDLASSGVMFSIDTESGFDQVVFITSAWGLGEMVVQGAVNPDEFYVHKPTLAANRPSIVRRTMGSKKIRMVYAPTQEHGKQVTIEDVPQESRDIFSLTNDEVQELAKQAVQIEKHYGRPMDIEWAKDGHTGKLFIVQARPETVRSRGQVMERYTLHAQGKIIAEGRAIGHRIGAGPVKVIHDISEMNRIEPGDVLVTDMTDPDWEPIMKKAAAIVTNRGGRTCHAAIIARELGIPAVVGCGDATERMKDDEKVTVSCAEGDTGYVYADMLDFSVKSSSVDTMPDLPLKVMMNVGNPDRAFDFACLPNEGVGLARLEFIINRMIGVHPRALLEFDDQDAKLQNEIREMMKGFDSPREFYVGRLTEGIATLGAAFWPKRVIVRLSDFKSNEYANLVGGERYEPEEENPMLGFRGAGRYVSDSFRDCFALECDAVKRVRNEMGLTNVEIMIPFVRTVEQAKAVVEELARQGLKRGENGLKIIMMCEIPSNALLAEQFLQYFDGFSIGSNDMTQLALGLDRDSGVVSELFDERNDAVKALLSMAIRAAKKQGKYVGICGQGPSDHEDFAAWLMEEGIDSLSLNPDTVVQTWLSLAELKK>UPI00049FBED1 Uncharacterized protein OS=Citrobacter amalonaticus OX=35703 GN=ILP74_18465 SS=EMBLWGS:MBE0397351 PC=UP000642697:Unassembled WGS sequenceMHIILNSLRYALWLVFCLFRHAVFVPAAFGGVYLLAWLVFGHPVSDLNDQLQKEATAWRTAPPGHYMWEECPVPDNAAPPEAKPAACTVTAVSTETAANNYLLSLRAVWIIFFILSNVLYVLWRLLADALRYHSLSCREAGAGRGTYIRMADGKIIKEADDE>UPI00049FBF2C Fumarate reductase subunit FrdC OS=Citrobacter amalonaticus OX=35703 GN=frdC SS=EMBLWGS:MBE0394040 PC=UP000642697:Unassembled WGS sequenceMTTKRKPYVRPMTSTWWKKLPFYRFYMLREGTAVPAVWFSIELIFGLFALKHGVESWAGFVGFLQNPVVVILNVIALAAALLHTKTWFELAPKAANIIVKDEKMGPEPIIKGLWAVTVVATVVILFVALFW>UPI00049FBF4A Murein transglycosylase A OS=Citrobacter amalonaticus OX=35703 GN=mltA SS=EMBLWGS:MBE0394408 PC=UP000642697:Unassembled WGS sequenceMKGRWVSYLLMGAVVAMLAACSSKPTDRGQQYKDGKFTQPFSLVNQPDAIGAPINAGDFAEQVNQIRSASPRLYGNQSNVYNAVQDWLRSGGDTRTMRQFGLDAWQMEGADNYGNVQFTGYYTPVIQARHTRQGEFQYPIYRMPPKRGRLPSRAEIYAGALSENYILAYSNSLMDNFIMDVQGSGYIDFGDGSPLNFFSYAGKNGHAYRSIGKVLIDRGEVKKEDMSMQAIRHWGETHSEAEVRELLEQNPSFVFFKPQSFAPVKGASAVPLIGRASVASDRSIIPAGTTLLAEVPLLDNNGKFNGQYELRLMVALDVGGAIKGQHFDIYQGIGPDAGHRAGWYNHYGRVWVLKSAPGAGNVFSG>UPI00049FBF95 Sulfurtransferase TusE OS=Citrobacter amalonaticus OX=35703 GN=tusE SS=EMBLWGS:MBE0396336 PC=UP000642697:Unassembled WGS sequenceMLIFEGKEIATDADGYLKDSAQWSEPMAVVIAENEGIILSPEHWEVVRFVRDFYLEFNTSPAIRMLVKAMANKFGEEKGNSRYLYRLFPKGPAKQATKIAGLPKPVKCI>UPI00049FBFB8 Glutamate mutase L OS=Citrobacter amalonaticus OX=35703 GN=ILP74_14410 SS=EMBLWGS:MBE0396604 PC=UP000642697:Unassembled WGS sequenceMQTVSVDIGSTWTKAALFAKEGDALTLVNHVLTPTTTHHLAEGFFASLNQVLNVADARPLLNRGEVTLKYSSSAKGGLAVAAMGLVPSITLESAKVTAHSAGAKIAQYYSYKLNRHDIQALEASPPDILLFTGGTDGGEESYGLANARALAESSLDCAIIYAGNRDIQDDVQAILGHKDLTTVDNILPDLDHPNPFAARKAICDVFLSRIVKGKGLDVIVGETGEEPMPTPWTVYELVKAISEVDSAWREFMLIDMGGATTDVYSASANTLSPDTVLHGVPEPFVKRTVEGDLGMRVSAVVVGESTQELVKVVFAQQPARQEAFYGYLRHLVAHPDYLPQSEEEKYFDSLLAGLCVGYAAERHAGTKKQVCTCVGNVDLQMGRDLTTVRKVVGSGGWLSRASQFDIHHWLKYRELDDDGRRILLPTQFEYYRDAKGLLPLLANVARLDPQAAARTSIHCLTL>UPI00049FC008 ABC transporter substrate-binding protein OS=Citrobacter amalonaticus OX=35703 GN=ILP74_09325 SS=EMBLWGS:MBE0395652 PC=UP000642697:Unassembled WGS sequenceMHANVTSLPYKKMLLVVAGAALFLTSGLSHAAKTEYPLTIKNCGRDMTFHAAPKRVATVGQNSTEILYALGLADRVVGTSLWFGPVPDAYKAANDKIAVIAQNIPSFEGIIAKKPDLVASQFEWQIGPAGTVASYEQFSELKVPVYTAPADCAKDNEDGGDGVRKGMFDIAMVYQEVADLARIFDVQDKGEELIASLKARETAAKNKIAGMDNSVSAVFWFSSADLQLDPYVAGKLGPAAWIAQTLGIKNIIDSAEEWPTVGWETIAKAQPTVIVLGEMSRRRFPADDWQVKMDYLKSDPVTQLIPAVKADHLPVIDVQTMNAGIRTIDGVEKLADALVEYGLAHPQAAH>UPI00049FC071 Type IV conjugative transfer system coupling protein TraD OS=Citrobacter amalonaticus OX=35703 GN=traD SS=EMBLWGS:MBE0397350 PC=UP000642697:Unassembled WGS sequenceMSNRYVIEALLRPAVELNTAVVSGIAAYVCVQAPWAVALAPSVSYVTAAGFAALAVTRTHQGMKIIRYRRNLRRLPRYVMSTKQIPVSHRRLFLGRGFRWTQKHTQRLQDTLRPEVARYLQPNRFYLGARQLEMMTEHRLPWLGKLLSADTPLNPVRPLPPVGGNPALHGIEPDEKDVTLALGERVGHTVVYGTTRVGKTRLAELLVTQDIRRDEVTIVFDPKGDADLMKRVWAEAHRAGRGDELYIFHLGWPEISARYNAVGRFGRVSEVASRVAGQLSGEGNSAAFREFAWRFVNIIARALVALGERPDYTLIMRYVNNIADLYIRYAEKIIQAQLPALQTQIENNQQVLGEDDVPRNMQGQPDALRIWAIEVALSSEEGKKLYDPILDGLRSAVRYDRTYFDKIVASLLPLLEKLTTGKTAELLSPDYQDIDDTRPIFDWEQIIRKKAVVYVGLDALSDSEVASAVGNSMFADLVSVAGHIYKHGINAGLPGGKEGKSLINLHCDEFNELMGDEFIPLINKGGGAGMQVTAYTQTSSDIEARIGNAAKTAQVQGNFNNLIMLRVRENRTAELLTTQLPQVEIYTKTLVSGHQDTADVNADQDFTSSTQDRVGTVKVPLLEPADIVTLPKGQAFALLEGGQLWKIRMPLPAGDADDVLMPESIEKIAEEMRRSYHSGESWWRDGPALNVPVTGGANG>UPI00049FC10F Xylose ABC transporter ATP-binding protein OS=Citrobacter amalonaticus OX=35703 GN=ILP74_21405 SS=EMBLWGS:MBE0397911 PC=UP000642697:Unassembled WGS sequenceMSCLLEMKNITKTFGVVKAIDNVSLRLNAGEIISLCGENGSGKSTLMKVLCGIYPHGSYEGEIVFAGETLQANHIRDTERKGIAIIHQELALVKHLTVLENIFLGSEISRHGVLDYDMMTLRCQKLLRQVSLAISPDTRVGELGLGQQQLVEIAKALNKQVRLLILDEPTASLTEQETTVLLEIIRDLQQHDIACIYISHKLNEVKAISDTICVIRDGKHIGTRDAAGMSEDDIITMMVGRELTALYPSEPHTTGEEILRVDHLTAWHPVNRHIRRVNDVSFSLKRGEILGIAGLVGAGRTELVQCLFGVWPGRWEGEVVIDGQSVNIRNCQQAIAHGIAMVPEDRKRDGIVPVMAVGKNITLAALDQFTGKVSQLDDAAEYKCILESLQRLKVKTSSPELAIGRLSGGNQQKAILARCLLLNPQILILDEPTRGIDIGAKYEIYKLINQLVQQGIAVIVISSELPEVLGLSDRILVMHEGKLKANLVNHNLTQEQVMEAALRSEHHVEKQSV>UPI00049FC18B Citrate/succinate antiporter CitT OS=Citrobacter amalonaticus OX=35703 GN=citT SS=EMBLWGS:MBE0396713 PC=UP000642697:Unassembled WGS sequenceMSLSKDSIWKLLAPLVVMGVMFLIPVPDGMPPQAWHYFAVFVAMIVGMILEPIPATAISFIAVTICVIGSNYLLFDAKELADPAFDAGKQALKWGLAGFSSTTVWLVFGAFIFALGYEVTGLGRRIALFLVKFMGKRTLTLGYAIVIIDILLAPFTPSNTARTGGTVFPVIKNLPPLFKSFPNDPSARRIGGYLMWMMVISTSLSSSMFVTGAAPNVLGLEFVSKIAGVQISWLQWFLGFLPVGIILLIVAPWLSYVLYKPEITHSAEVAAWAGDELKTMGTLTRKEWTLIGLVLLSLGLWVFGGEMIDATAVGLLAVSLMLALHVVPWKDITKYNSAWNTLVNLATLVVMANGLTRSGFIDWFASTMSTHLEGFSPNATVIVLVLVFYFAHYLFASLSAHTATMLPVILAVGKGIPGVPMEHLCILLVLSIGIMGCLTPYATGPGVIIYGCGYVKSKDYWRLGAIFGVIYIAMLLLVGWPILAMWN>UPI00049FC20E Cell envelope integrity protein TolA OS=Citrobacter amalonaticus OX=35703 GN=tolA SS=EMBLWGS:MBE0396592 PC=UP000642697:Unassembled WGS sequenceMSKATEQNDKLKRAIIISAVLHVILFAVLIWSSFDEHIEASAGGGGGSSIDAVMVDPGAVVQQYERQQQQQSSAQRAKEQREKLEQQQAEELREKQAAEQERLKQIEKERLAAQEQQKQAEADAKKAQEQQKQAEEAAKKAAADAKAKADAQVKEAAEAAKKAAADAQKKAEAEAAKAAADAKKKAEAEAAKAAADAKKKAEAEAAKQAAAEKAAAAKAAAAAEKAAAEKKAAEKAAAEKKAAAEKAAADKKAAAEKAAADKKAAAEKAAAAKKAAAEKAAAASGVDDLLGDLSSGKNAPKTGGGAKGNNASPAGSGNTKNNGASGADISNYAGQIKSAIESKFYDASSYAGKTCTLRIKLASDGMLLDIQSEGGDPALCQAALAAARQAKIPKPPSQAVYEVFKNAPLDFKP>UPI00049FC20F LuxR family transcriptional regulator OS=Citrobacter amalonaticus OX=35703 GN=ILP74_02270 SS=EMBLWGS:MBE0394332 PC=UP000642697:Unassembled WGS sequenceMKLSDSVFSDDYFFIVGISALLTPELIDENYTIVDVDESLLQRSTEYLFPGRKIIAFITNDLDYYALCHLRDITFIDKRRRINEILSCLFVNDSRYAYRVKYTLSFRESEVLDCIQKGMEANEIGELLGMSMKTFYAHRRSLIFKLQLGNRVSLYRNIARIRMCKPCVHEPDLT>UPI00049FC23D Fatty acid biosynthesis protein FabY OS=Citrobacter amalonaticus OX=35703 GN=fabY SS=EMBLWGS:MBE0398225 PC=UP000642697:Unassembled WGS sequenceMYHLRVPQTEEELERYYQFRWEMLRKPLHQPKGSERDAWDAMAHHQMVVDEEGNLVAVGRLYINADNEASIRFMAVDPTVQEKGLGTLMAMTLESVARQEGVKRVTCSAREDAVEFFAKLGFVNQGEITTPQTTPVRHFLMIKPIASLDDILHRGDWCGQLQQAWYEHIPLSEKMGVRIQQYTGQKFITTMPETGNQNPHHTLFAGSLFSLATLTGWGLIWLMLRERHLGGTIILADAHIRYSKPISGRPAAVADLGSLSGDLDRLARGKKARVQLQVELLGNDTPGAIFEGIYIVLPAKPFGPYEEGGNEEE>UPI00049FC289 Family 43 glycosylhydrolase OS=Citrobacter amalonaticus OX=35703 GN=ILP74_17185 SS=EMBLWGS:MBE0397111 PC=UP000642697:Unassembled WGS sequenceMQNWPNPFIEQRADPFILRDGSDYYFIASVPEYDRLEIRRADSLEGLRTASPVVVWRKPEHGPMSELIWAPEIHHLDGRWVIYFAAAHTQALDKLGMFQHRMYALECTDPDPLTGNWVEKGQVKTPFDTFALDATTFHHQGKQWYLWAQKSPDIAGNSNIYLAELENPWTIKGQPVMLSKPEYDWECRGFWVNEGPAVLTHGDRLFISYSASATDENYCMGLLWIDIHADPLNPANWHKSPRPVFTTSNENRQYGPGHNSFTQTPEGDDVLVYHARNYTEIEGDPLYDPNRHTRLKLIHWDDHGMPDFGIPPADTL>UPI00049FC30A FMNH2-dependent alkanesulfonate monooxygenase OS=Citrobacter amalonaticus OX=35703 GN=ssuD SS=EMBLWGS:MBE0396362 PC=UP000642697:Unassembled WGS sequenceMSLNMFWFLPTHGDGHYLGTEEGSRPVDHGYLQQIAQAADRLGFTGVLIPTGRSCEDAWLVAASMIPVTQRLKFLVALRPSVTSPTVAARQAATLDRLSNGRALFNLVTGSDPQELAGDGVFLDHSERYEASSEFTQVWRRLLQGETVDFNGKHIHVRGAKLFFPPIQQPYPPLYFGGSSDVAQDLAAEQVDLYLTWGEPPELVKEKIAQVRAKAAARGRNIRFGIRLHVIVRETNEEAWRAADRLIAHLDDDTIAKAQAAFARTDSVGQHRMAALHNGKRDQLEISPNLWAGVGLVRGGAGTALVGDGPTVAARINEYAALGIDSFVLSGYPHLEEAYRVGELLFPHLDVAIPEVPQPQRQHQQGEAVANEFIPRKVAQS>UPI00049FC3CC Sensor histidine kinase OS=Citrobacter amalonaticus OX=35703 GN=ILP74_06580 SS=EMBLWGS:MBE0395141 PC=UP000642697:Unassembled WGS sequenceMYEFNLVLLLLQQMCVFLVIAWLMSKTRLFIPLMQVTVRLPHKLLCYVTFSIFCILGTYFGLHIDDSIANTRAIGAVMGGLLGGPVVGGLVGLTGGLHRYSMGGMTALSCMISTIVEGLLGGLVHSVLTRRGRTDKVFNPLTAGAITFVAEMVQMLIILLIARPFDDAYRLVSNIAAPMMVTNTVGAALFMRILLDKRAMFEKYTSAFSATALKVAASTEGILRQGFNEVNSMKVAQVLYKELDIGAVAITDRERLLAFTGIGDDHHLPGRPISSGYTLRAIETGEVVYADGNEVPYRCSLHPQCKLGSTLVIPLRGENQRVMGTIKLYEAKNRLFSSINRTLGEGIAQLLSAQILAGQYERQKAMLTQSEIKLLHAQVNPHFLFNALNTIKAVVRRDSEQASQLVQDLSTFFRKNLKRPSEIVTLADEIEHVNAYLQIEKARFQSRLQVSLNVPDALAHQQLPAFTLQPIVENAIKHGTSQLLGTGEVSITARQEGQYLMLDIEDNAGLYQPSANVSGLGMNLVDKRLRERFGDDYGISVVCEPDCFTRITLRLPLEETHD>UPI00049FC3F9 RNA polymerase sigma factor RpoH OS=Citrobacter amalonaticus OX=35703 GN=rpoH SS=EMBLWGS:MBE0397805 PC=UP000642697:Unassembled WGS sequenceMTKEMQNLALAPVGNLESYIRAANAWPMLSADEERALAEKLHYQGDLEAAKTLILSHLRFVVHVARNYAGYGLPQADLIQEGNIGLMKAVRRFNPEVGVRLVSFAVHWIKAEIHEYVLRNWRIVKVATTKAQRKLFFNLRKTKQRLGWFNQDEVEMVARELGVSSKDVREMESRMAAQDMTFDMSPDDESDSQPMAPVLYLQDKTSNFADGIEEDNWEDQAANKLTHAMEGLDERSQDIIRARWLDEDNKSTLQELADRYGVSAERVRQLEKNAMKKLRAAIEA>UPI00049FC438 Lipopolysaccharide biosynthesis protein OS=Citrobacter amalonaticus OX=35703 GN=ILP74_12770 SS=EMBLWGS:MBE0396297 PC=UP000642697:Unassembled WGS sequenceMKKWFADGAFRTIIRNSAYLGSSNVVSALLGLLALSCAGKGMTPAMFGVLVIVQSYTKAISDFAKFQTWQLVVQYGTPALENNNQQQFRDVISFSFSLDIASGAAAILCGMGLLPFLSHSLGLDADSFWLAMLYCTLIPSMASSTPTGILRAVNRFDLIAVQQATKPFLRALGSVIAYFGDFGFAGFVITWYASSLVGGTMYWWFAARELRSRNIHGALRPRLFESARRLEGAWNFVWATNFAHTIWSARNSCSTVLVGVVLGPAAAGLFKIAMTFFDATGTPAQLLAKSFYPEVMRLDPRSKKPWQLGMKSALLAGGIGIVVALAVVIVGKPLISLVFGVKYLQAYDLIQIMLGAIIVSMLGFPQESLLLMSGKQRAFLTAQTLASIAYIVLLPGLSHLFGVIGAAFAYFAGQCLDVLLSLIPTLHAYRHRQRLPFTADKETHS>UPI00049FC582 4-hydroxy-tetrahydrodipicolinate synthase OS=Citrobacter amalonaticus OX=35703 GN=dapA SS=EMBLWGS:MBE0394691 PC=UP000642697:Unassembled WGS sequenceMFTGSIVALVTPMDEKGNVCRSSLKKLIDYHVASGTSAIVSVGTTGESATLSHDEHGDVVMMTLELADGRIPVIAGTGANATAEAISLTQRFNDSGIVGCLTVTPYYNRPTQEGLFQHFKAIAEHTDLPQILYNVPSRTGCDMLPETVGRLAELKNIVAIKEATGNLSRVHQIKELVSDDFLLLSGDDATAMDFMQLGGHGVISVTSNVAARDMAEMCKLAAEGRFAEARTINQRLMPLHNKLFVEPNPIPVKWACKALGLVATDTLRLPMTPITDNGREIVKAALKHAGLL>UPI00049FC754 Glycerol dehydrogenase OS=Citrobacter amalonaticus OX=35703 GN=ILP74_23730 SS=EMBLWGS:MBE0398347 PC=UP000642697:Unassembled WGS sequenceMDRIIQSPGKYIQGADVITRLGEYLKPLAERWLVVGDKFVLGFAQGELEKSFKNAGLALEVAPFGGECSQNEIDRLRSVAENAQCSAVLGIGGGKTLDTAKALAHFMNLPVAIAPTIASTDAPCSALSVIYTDAGEFDRYLLLPHNPHMVIVDTKIVAGAPARLLAAGIGDALATWFEARACSRSGAITMAGGKCTQAALALAELCYNTLLEEGEKAMLAAEQHVVTPALERVIEANTYLSGVGFESGGLAAAHAVHNGLTAIPDAHHYYHGEKVAFGTLTQLILENAPVEEIETVAALCHSVGLPITLAQLDIKQDIPAKMRIVAEASCAEGETIHNMPGGATPDQVYAALLVADQYGQRYLQEWE>UPI00049FC7BC Flagellar motor protein MotB OS=Citrobacter amalonaticus OX=35703 GN=motB SS=EMBLWGS:MBE0395507 PC=UP000642697:Unassembled WGS sequenceMKNRSHPIVVVKRRKHKGHGGGAHGSWKIAYADFMTAMMAFFLVMWLISISSPKELIQIAEYFRTPLATAVTGGNRISNSQSAIPGGGDDFTQQQGEVNKQPNLDELKKRMEQNRLSKLRGDLDQLIESDPKLRALRPHLKIDLVQEGLRIQIIDSQNRPMFKTGSAEVEPYMRDILRAIAPVLNGIPNRISLSGHTDDFPYANGEKGYSNWELSADRANASRRELVQGGLDDGKVLRVVGMAATMRMSDRGPDDAINRRISLLVLNKQSEQAILHENAESQNEPVSVLQQPEASPQVSVPTSPPANPR>UPI00049FC7D1 Polyphosphate kinase 1 OS=Citrobacter amalonaticus OX=35703 GN=ppk1 SS=EMBLWGS:MBE0394675 PC=UP000642697:Unassembled WGS sequenceMGQEKLYIEKELSWLAFNERVLQEAADKSNPLIERMRFLGIYSNNLDEFYKVRFAELKRRIIISEEQGSNSHSRHLLGKIQSRVLKADQEFDGLYNELLLEMARNQIFLINERQLSVNQQSWLRHYFKQYLRQHITPILINRETDLVQFLKDDYTYLAVEIIRGDTIRYALLEIPSDKVPRFVNLPPEAPRRRKPMILLDNILRYCLDDIFKGFFDYDALNAYSMKMTRDAEYDLVHEMESSLMELMSSSLKQRLTAEPVRFVYQRDMPNALVEVLREKLTISRYDSIVPGGRYHNFKDFINFPNVGKANLVNKPLPRLRHIWFDKEKFRNGFDAIRERDVLLYYPYHTFEHVLELLRQASFDPSVLAIKINIYRVAKDSRIIDSMIHAAHNGKKVTVVVELQARFDEEANIHWAKRLTEAGVHVIFSAPGLKIHAKLFLISRKEGEEVVRYAHIGTGNFNEKTARLYTDYSLLTADARITNEVRRVFNFIENPYRPVTFDYLLVSPQNSRRLLYEMIDREIANAQQGLPSGITLKLNNLVDKGLVDRLYAASSSGVPVNLLIRGMCSLIPNLEGISDNIRVISIVDRYLEHDRVYIFENGGDKQVYLSSADWMTRNIDYRIEVATPLLDPRLKQRVLDIIDILFSDTVKARYIDKELSNRYVPRGNRRKVQSQLAIYDYIKSLEQSD>UPI00049FC7F7 1-deoxy-D-xylulose-5-phosphate synthase OS=Citrobacter amalonaticus OX=35703 GN=dxs SS=EMBLWGS:MBE0396885 PC=UP000642697:Unassembled WGS sequenceMSFDIAKYPTLALVDSTQELRLLPKESLPKLCDELRRYLLDSVSRSSGHFASGLGTVELTVALHYVYNTPFDQLIWDVGHQAYPHKILTGRRDKIGTIRQKGGLHPFPWRGESEYDVLSVGHSSTSISAGIGVAVAAEKEGKDRRTVCVIGDGAITAGMAFEAMNHAGDIKPDMLVILNDNEMSISENVGALNNHLAQLLSGKLYSSLREGGKKVFSGVPPIKELLKRTEEHIKGMVVPGTLFEELGFNYIGPVDGHDVLGLITTLKNMRDLKGPQFLHIMTKKGRGYEPAEKDPITFHAVPKFDPSSGCLPKSSGGLPSYSKIFGDWLCETAAKDSKLMAITPAMREGSGMVEFSRKFPDRYFDVAIAEQHAVTFAAGLAIGGYKPVVAIYSTFLQRAYDQVIHDVAIQKLPVMFAIDRAGIVGADGQTHQGAFDLSYLRCIPDMVIMTPSDENECRQMLFTGYHYSDGPTAVRYPRGNAVGVELTPLEKLPIGKGVVKRRGEKLAILNFGTLMPDAAKVAESLNATLVDMRFVKPLDEALILEMAACHEVLVTLEENAIMGGAGSGVNEVLMAHRKPVPVLNIGLPDFFIPQGTQDEARAGLGLDAAGIEAKIKAWLA>UPI00049FC810 Transcriptional regulator OS=Citrobacter amalonaticus OX=35703 GN=ILP74_20370 SS=EMBLWGS:MBE0397709 PC=UP000642697:Unassembled WGS sequenceMSRSLLTNETSELDLLDQRPFDQTDFDILKSYEAVVDGLAMLIGSHCEIVLHSLQDLKCSAIRIANGEHTGRKIGSPITDLALRMLHDMTGADSSVSKCYFTRAKSGVLMKSVTIAIRNREHRVIGLLCINMNLDVPFSQIMNTFIPPETPEVGSSVNFASSVEDLVTQTLEFTIEEVNADRNVSNNAKNRQIVLNLYEKGIFDIKDAINQVADRLNISKHTVYLYIRQFKSGDFQGQDK>UPI00049FC882 YgeY family selenium metabolism-linked hydrolase OS=Citrobacter amalonaticus OX=35703 GN=ILP74_01830 SS=EMBLWGS:MBE0394257 PC=UP000642697:Unassembled WGS sequenceMAKHIPFKLILEKANDYKEDMTRFLRDMVAIPSESCDEKRVIHRIKQEMEKVGFDKVEIDPMGNILGYIGHGPRLVAMDAHIDTVGIGNINNWNFDPYEGMETDELIGGRGTSDQEGGMASMVYAGKIIKDLGLEDEYTLLVTGTVQEEDCDGLCWQYIIEQSGIRPEFVVSTEPTDCQVYRGQRGRMEIRIDVQGVSCHGSAPERGDNAIFKMGPILNELQELSQHLAYDEFLGKGTLTVSEIFFTSPSRCAVADSCAVSIDRRLTWGETWEGALEEIRALPAVKKANAVVSMYNYDRPSWTGLVYPTECYFPTWKVEEDHFTVKALVNAYEGLFGKAPVVDKWTFSTNGVSIMGRHGIPVIGFGPGKEPEAHAPNEKTWKSHLVTCAAMYAAIPLSWLATK>UPI00049FC8AB Lactoylglutathione lyase OS=Citrobacter amalonaticus OX=35703 GN=gloA SS=EMBLWGS:MBE0396007 PC=UP000642697:Unassembled WGS sequenceMRLLHTMLRVGDLQRSIDFYTKVLGMKLLRTSENTEYKYSLAFVGYGEESDEAVIELTYNWGVDKYELGTAYGHIALSVDNAAEACERIRQNGGNVTREAGPVKGGTTVIAFVEDPDGYKIELIEEKDAGKGLGN>UPI00049FC969 YhjR family protein OS=Citrobacter amalonaticus OX=35703 GN=ILP74_21215 SS=EMBLWGS:MBE0397876 PC=UP000642697:Unassembled WGS sequenceMSDNEPGTQTDSTLGYTFQNDFLALSQAFSLPEFDYTDISQREQLAAAIKRWPLLAEFAQQQ>UPI00049FC987 Aspartate ammonia-lyase OS=Citrobacter amalonaticus OX=35703 GN=aspA SS=EMBLWGS:MBE0394025 PC=UP000642697:Unassembled WGS sequenceMLNNIRIEEDLLGTREVPAEAYYGVHTLRAIENFYISNNKISDIPEFVRGMVMVKKAAALANKELQTIPKSVANAIIAACDEVLNNGKCMDQFPVDVYQGGAGTSVNMNTNEVLANIGLELMGHQKGEYQYLNPNDHVNKCQSTNDAYPTGFRIAVYASIVKLVDAINQLREGFERKAVEFQDILKMGRTQLQDAVPMTLGQEFRAFSVLLKEEVKNIERTAELLLEVNLGATAIGTGLNTPKEYSPLAVQKLAEVTGFACVPAEDLIEATSDCGAYVMVHGALKRLAVKMSKICNDLRLLSSGPRAGLNEINLPELQAGSSIMPAKVNPVVPEVVNQVCFKVIGNDITVTMASEAGQLQLNVMEPVIGQAMFESIHILSNACYNLLEKCVNGITANKEVCEGYVYNSIGIVTYLNPFIGHHNGDIVGKICAETGKSVREVVLERGLLTEAELDDIFSAQNLMHPAYKAKRYTDENEQ>UPI00049FCAC7 Excinuclease ABC subunit UvrA OS=Citrobacter amalonaticus OX=35703 GN=uvrA SS=EMBLWGS:MBE0398467 PC=UP000642697:Unassembled WGS sequenceMDKIEVRGARTHNLKNINLVIPRDKLIVVTGLSGSGKSSLAFDTLYAEGQRRYVESLSAYARQFLSLMEKPDVDHIEGLSPAISIEQKSTSHNPRSTVGTITEIHDYLRLLFARVGEPRCPDHDVPLAAQTVSQMVDNVLSQPEGKRLMLLAPVIKERKGEHTKTLENLASQGYIRARIDGEVCDLSDPPKLELQKKHTIEVVIDRFKVRDDLSTRLAESFETALELSGGTAVVADMDDEKAEELLFSANFACPICGYSMRELEPRLFSFNNPAGACPTCDGLGVQQYFDPDRVIQNPELSLAGGAIRGWDRRNFYYFQMLKSLAEHYKFDVEAPWASLSANVHKVVLYGSGKESIEFKYMNDRGDTSVRRHPFEGVLHNMERRYKETESSAVREELAKFISNRPCASCEGTRLKREARHVFVENTPLPTISDMSIGHAMDFFNNLKLAGQRAKIAEKILKEIGDRLKFLVNVGLNYLTLSRSAETLSGGEAQRIRLASQIGAGLVGVMYVLDEPSIGLHQRDNERLLGTLIHLRNLGNTVIVVEHDEDAIRAADHVIDIGPGAGVHGGEVVAEGPLEAIMAVPESLTGQYMSGKRKIEVPKQRVPANPEKVLKLTGARGNNLKDVTLTLPVGLFTCITGVSGSGKSTLINDTLFPIAQRQLNGATIAEPAPYRDVQGLEHFDKVIDIDQSPIGRTPRSNPATYTGVFTPVRELFAGVPESRSRGYTPGRFSFNVRGGRCEACQGDGVIKVEMHFLPDIYVPCDQCKGKRYNRETLEIKYKGKTIHEVLDMTIEEAREFFDAVPALARKLQTLMDVGLTYIRLGQSATTLSGGEAQRVKLARELSKRGTGQTLYILDEPTTGLHFADIQQLLDVLHQLRDQGNTIVVIEHNLDVIKTADWIVDLGPEGGSGGGEILVSGTPETVAECEASHTARFLKPML>UPI00049FCB5B Multidrug/spermidine efflux SMR transporter subunit MdtJ OS=Citrobacter amalonaticus OX=35703 GN=mdtJ SS=EMBLWGS:MBE0395962 PC=UP000642697:Unassembled WGS sequenceMFYWILLGLAIAAEITGTLSMKWASVSDDNSGFILMLVMIAASYIFLSFAVKKIALGVAYALWEGIGILFITLFSVLLFDEPLSVMKIAGLATLVVGIVLIKSGTRKARQSTKEVTHATV>UPI00049FCCC0 YnfA family protein OS=Citrobacter amalonaticus OX=35703 GN=ILP74_10890 SS=EMBLWGS:MBE0395947 PC=UP000642697:Unassembled WGS sequenceMLKTTLLFFATALCEIIGCFLPWLWLKRGATVWLLLPAGVALALFVWLLTLHPAASGRVYAAYGGVYVCTALIWLRVVDGVKLSLYDWSGALIALCGMLIIVAGWGRG>UPI00049FCCCD Transcription antitermination factor NusB OS=Citrobacter amalonaticus OX=35703 GN=nusB SS=EMBLWGS:MBE0396889 PC=UP000642697:Unassembled WGS sequenceMKPAARRRARECAVQALYSWQLSQNDIADVEYQFLAEQDVKDVDVMYFRELLSGVATNSAYLDGLMKPYLSRLLEELGQVEKAVLRIALFELSKRSDVPYKVAINEAIELAKTFGAEDSHKFVNGVLDKAAPAIRPNKK>UPI00049FCCD6 Ribonuclease E inhibitor RraB OS=Citrobacter amalonaticus OX=35703 GN=rraB SS=EMBLWGS:MBE0394118 PC=UP000642697:Unassembled WGS sequenceMANPELLEEQREETRLIIEELLDDGSDPDALYTIEHHLSADDFETLEKAAVEAFKLGYEVTEPEELEVEEGDTVICCDILSECALKADLIDAQVEQLMNLAEKYDVEYDGWGTYFEDPNGEDGEEGDDEDYVDEDDDGVRH>UPI00049FCCF5 DNA-protecting protein DprA OS=Citrobacter amalonaticus OX=35703 GN=dprA SS=EMBLWGS:MBE0397662 PC=UP000642697:Unassembled WGS sequenceMTRTEIWLRLMAVNELFGDEMVRVAHGLISQPAIDIAALRRAGLSPSQATRFLTFPEKELERTLRWLELPHHHLLLADSEDYPAQLRAIEDYPGAVFVVGSPRCLHDFQLAVVGSRTPSWYGERWGQMFCEKLASWDITITSGLARGIDGIAHRAAIQAKGKSIAVLGNGLQSVYPRRHAGMADALLETGGALVSEFPLMMAPLPRNFPRRNRIISGLSKGVLVVEAALRSGSLVTARCALEQGREVFAIPGPLGSPGSEGPHWLVKQGATLVTAPEEIMENLQYGLHWLPDEPEKSLYSSDQEGAALPFPELLANVGDEVTPVDVVAERAGQPVPEVVAQLLELELAGWIAVVPGGYVRLRRACHVRRTNVFV>UPI00049FCE60 HTH-type transcriptional regulator OS=Citrobacter amalonaticus OX=35703 GN=ILP74_21350 SS=EMBLWGS:MBE0397900 PC=UP000642697:Unassembled WGS sequenceMESKDPMFELLSSLEQIVFKEETSKITLTHKPSPFSEFEQLRRGSGLETDDFARALGVTVALVQEWESKRVKPSSTELKLMRLIQANPALSKQLME>UPI00049FCEF9 Two-component system response regulator RstA OS=Citrobacter amalonaticus OX=35703 GN=rstA SS=EMBLWGS:MBE0395970 PC=UP000642697:Unassembled WGS sequenceMNTIVFVEDDPEVGALIAAYLAKHDFDVIVEPRGDLAEARILQVQPDLVLLDIMLPGKDGMTLCRDLQGQWQGPIVLLTSLDSDMNHILALEMGACDYILKTTPPAVLLARLRLHLRQSDQSGHSKGIQPAAVTPHKTLRFGSLTIDPINRVVLLSGEQVILSTADFELLWELATHAGQIMDRDALLKNLRGVSYDGMDRSVDVAISRLRKKLLDNAAEPYRIKTVRNKGYLFAPHAWDDVPSRHSGM>UPI00049FCFC7 Urease accessory protein UreE OS=Citrobacter amalonaticus OX=35703 GN=ureE SS=EMBLWGS:MBE0397437 PC=UP000642697:Unassembled WGS sequenceMLLLTQRVDTPAHITATLTLPIDIRVKSRAKVQLNDGREAGLMLPRGLLLRGGDCLSTEDGTEVVEIIAASEAVSVVRCTDPFLLAKACYHLGNRHVPLQILPDELRYHHDHVLDTMLRQFRLEVTFAHLPFEPEAGAYAGESHGHHHSHAH>UPI00049FD007 Bifunctional biotin--[acetyl-CoA-carboxylase] synthetase/biotin operon repressor OS=Citrobacter amalonaticus OX=35703 GN=ILP74_23910 SS=EMBLWGS:MBE0398376 PC=UP000642697:Unassembled WGS sequenceMKDNTVPLMLISLLADGEFHSGEQLGEQLGMSRAAINKHIQTLRDWGVDVFTVPGKGYSLPEPIQLLDSDRIYSQLDRGTVAVLPVIDSTNQYLLDRIDLLQSGDACVAEYQQAGRGRRGRKWFSPFGANLYLSMFWRLEQGPAAAIGLSLVIGIVMAEVLRDLGADKVRVKWPNDLYLLDRKLAGILVELTGKTGDAAQIVIGAGINMAMRRVEEGVVNQGWITLQEAGITLDRNTLAARLIRELRTALELFEQEGLAPYLSRWEKLDNFINRQVKLIIGDKEIYGISRGIDAQGALLLEQDGIIKPWVGGEISLRSAE>UPI00049FD11C Hydroxyethylthiazole kinase OS=Citrobacter amalonaticus OX=35703 GN=thiM SS=EMBLWGS:MBE0395149 PC=UP000642697:Unassembled WGS sequenceMQPDLHSREHAVRTLQDFRTLSPLTHCMTNDVVQSFTANTLLALGASPAMVIEPEEARQFAALASALLINVGTLTQPRAHAMRAAVEQANSAKTPWTLDPVAVGALEYRRRFCLELLALTPAAIRGNASEILALAGESAGGRGVDATDPVATALPAAQILARHTGAVVVVTGEVDYITDGERTLSVTGGDPLMTKVVGTGCALSAVVAASCALPGNRIENIASACGWMKLAGQHASKICHGPGSFLPAFLDALYTLDAEVAA>UPI00049FD1BF Cell-envelope stress modulator CpxP OS=Citrobacter amalonaticus OX=35703 GN=cpxP SS=EMBLWGS:MBE0398269 PC=UP000642697:Unassembled WGS sequenceMRKVTAAVMASTLALSTVSHAAEVVTGDNWHPGESSAPRTVQSHMFDGISLTEHQRQQMRDLMQQARHEQPPVNVSEMETMHRLITAENFDETAVRAQAEKMAQAQVARQVEMARVRNQMYRLLTPEQQAVLNEKHQQRMEQLRDVTHWQKSSSLKLLSSSNSRSQ>UPI00049FD256 Catalase/peroxidase HPI OS=Citrobacter amalonaticus OX=35703 GN=katG SS=EMBLWGS:MBE0398345 PC=UP000642697:Unassembled WGS sequenceMSTPDDIHNPTSAGKCPFHQGGHDQSAGAGTTSRDWWPNQLRVDLLNQHSNRSNPLGEDFDYRKEFSKLDYSALKGDLRALLTESQPWWPADWGTYAGLFIRMAWHGAGTYRSVDGRGGAGRGQQRFAPLNSWPDNVSLDKARRLLWPIKQKYGQKISWADLFILAGNVALENSGFRTFGFGAGREDVWEPDLDVNWGDEKTWLAHRDPEELAKRPLAATEMGLIYVNPEGPNASGEPLSAASAIRATFGNMGMNDEETVALIAGGHTLGKTHGAGPATHVGPDPEVAPIENQGLGWKSDFGSGVGADAITSGLEVVWTQTPTQWSNYFFENLFKYEWVQTRSPAGAIQFEAVDAPEIIPDPFDPSKKRKPTMLVTDLTLRFDPEFEKISRRFLNDPQAFNEAFARAWFKLTHRDMGPKARYIGPEVPKEDLIWQDPLPQPTFNPTEEDILSLKSAIADSGLSVGELVSVAWASASTFRGGDKRGGANGARLALAPQRGWDVNAIATRALPVLEKIQRDSAKASLADIIVLAGVVGVEKAASAAGVSIHVPFTPGRVDARQDQTDIEMFELLKPIADGFRNYRGEPGVATTESLLIDKAQQLTLTAPEMTVLVGGLRVLGANYDGSKHGVFTDRPGVLSNDFFVNLLDMRHEWKPVDESNEQFEGRDRQTGEVKYTASRADLVFGSHAVLRALAEVYAGSDAHVKFVKDFVAAWVKVMNLDRFDLQ>UPI00049FD288 Chaperone NapD OS=Citrobacter amalonaticus OX=35703 GN=napD SS=EMBLWGS:MBE0395006 PC=UP000642697:Unassembled WGS sequenceMHINWQVCSLIVQAKGEQVQDISTQLNALPGCEVAVSDEQSGQMIAVVEAEHSETLMQTIESVRNVAGVLAVSLVYHQQEEQGEETP>UPI00049FD2F9 YoaH family protein OS=Citrobacter amalonaticus OX=35703 GN=ILP74_08995 SS=EMBLWGS:MBE0395588 PC=UP000642697:Unassembled WGS sequenceMFAGLPSLSHEQQQKAVERIQELMSQGMSSGQAIALVAEELRATHTGERIVARFEDEDE>UPI00049FD319 50S ribosomal protein L11 methyltransferase OS=Citrobacter amalonaticus OX=35703 GN=prmA SS=EMBLWGS:MBE0397622 PC=UP000642697:Unassembled WGS sequenceMPWIQLKLNTTGANAEALSDALMEAGSVSITFQDTHDTPVFEPLPGETRLWGDTDVIGLFDAETDMKEVVAILEHHPLLGVGFAHKIEQLEDKDWEREWMDNFHPMRFGERLWICPSWRDVPDENAVNVMLDPGLAFGTGTHPTTSLCLQWLDSLDLTGKTVIDFGCGSGILAIAALKLGAAKAIGIDIDPQAIQASRDNAQRNGVSERLELYLPQDQPDAMKADVVVANILAGPLRELAPLISVLPVTGGLLGLSGILASQAESVCEAYADLFALDPVVEKEEWCRITGRKK>UPI00049FD335 DUF3561 family protein OS=Citrobacter amalonaticus OX=35703 GN=ILP74_02935 SS=EMBLWGS:MBE0394459 PC=UP000642697:Unassembled WGS sequenceMRNSQNITLTTTDAFATDDETTWSLPGAVVGFASWLLALGIPFLVYGPNTLFFFIYTWPFFLALMPVAVVVGIALHSLLNGKLRYSIVATLLTVCAMFGALFMWLLG>UPI00049FD342 F0F1 ATP synthase subunit A OS=Citrobacter amalonaticus OX=35703 GN=atpB SS=EMBLWGS:MBE0398099 PC=UP000642697:Unassembled WGS sequenceMASENMTPQDYIGHHLNNLQLDLRTFSLVDPHNPPATFWTLNIDSMFFSVVLGLLFLALFRSVAKKATSGVPGKFQTAIELIIGFVHGSVKDMYHGKSKLIAPLALTIFVWVFLMNLMDLLPIDLLPYIGEHIFGLPALRVVPSADVNVTLSMALGVFILILFYSIKMKGIGGFAKELTLQPFNHWAFIPVNLILEGVSLLSKPVSLGLRLFGNMYAGELIFILIAGLLPWWSQWILNVPWAIFHILIITLQAFIFMVLTIVYLSMASEEH>UPI00049FD350 Rod shape-determining protein MreD OS=Citrobacter amalonaticus OX=35703 GN=mreD SS=EMBLWGS:MBE0397609 PC=UP000642697:Unassembled WGS sequenceMASYRSQGRWVIWLSFFIALLLQVMPWPDEIIVFRPNWVLLILLYWILALPHRVNVGTGFVMGAILDLISGSTLGVRALSMSIIAYLVALKFQLFRNLALWQQALVVMLLSLVVDIIVFWSEFLVINVSFRPEVFWSSVVNGVLWPWLFLLMRKVRQQFAVQ>UPI00049FD4B5 DUF4311 domain-containing protein OS=Citrobacter amalonaticus OX=35703 GN=ILP74_01250 SS=EMBLWGS:MBE0394148 PC=UP000642697:Unassembled WGS sequenceMFLIILIKSLIIGALVGVGVGAGAARMFHAPTTQGMGAFRTLGELNSCEGDPASHFSFGLGFFFNAWASSVAAGSFTQDVDHRIIPNWGAAALMIKNRNVGETLHDPKKMAIACGVIGMIVVTFLNLTASSVPEALQVTAVKVLVPAANLLVNTVMPVIFWLAAIDAGKKSGFWATVFGGAAQLIMGNAVPGLVLGILIGKGVEESGWNHVTKVMMAAIVLLFVLSGFFRGFDMKMIESFHMTVPNWLELIHNSLSGK>UPI00049FD4CC Chorismate lyase OS=Citrobacter amalonaticus OX=35703 GN=ubiC SS=EMBLWGS:MBE0398454 PC=UP000642697:Unassembled WGS sequenceMSHPALTQLRALRYFEEIPALDPEQLDWLLLEDSMTKRFEQQGKRVTVTLIREGFVGQNEVVEELTRLPKESRYWLREILLCADDEPWLAGRTVVPESTLSGPELALQHLGKTPLGRYLFTSSTLTRDFIEIGCDAGLWGRRSRLRLSGKPLMLTELFLPASPLY>UPI00049FD502 Phosphatidylglycerophosphatase C OS=Citrobacter amalonaticus OX=35703 GN=yfhb SS=EMBLWGS:MBE0394617 PC=UP000642697:Unassembled WGS sequenceMTNHARRVVFFDLDGTLHQQDMFGSFLRYLLRRQPLNALLVLPLLPVIVGALLIKGRAARWPMSLLLWGCTFGHSEASLQARQADFVRWFRQNVTAFPQVQERLTTYLLSSDADIWLITGSPQSLVEQVYVDTPWLPRVNLIASQMKRGYGGWVLTLRCLGHEKVAQLERKIGTPLRLYSGYSDSNQDNPLLYFCQHRWRVTPRGELQQLE>UPI00049FD541 Phosphatase OS=Citrobacter amalonaticus OX=35703 GN=ILP74_12560 SS=EMBLWGS:MBE0396261 PC=UP000642697:Unassembled WGS sequenceMYPVDLHMHTVASTHAYSTLSDYIAQAKRQGLKLFAITDHGPDMADAPHHWHFINMRIWPRVVDGVGILRGIEANIKNIEGEIDCTGPMLTSLDLIIAGFHEPVFAPHDEATNTQAMIATMASGVVHIISHPGNPKYPVDITAIAEAAAKYNVALEINNSSFLHSRKGSEANCRAVAAAVRDAGGWVALGSDSHTAFTMGDFGECLKIIEEVNFPEDRILNVTPKRLLNFLESRGMAPIPEFAEL>UPI00049FD576 Formate dehydrogenase subunit beta OS=Citrobacter amalonaticus OX=35703 GN=fdxH SS=EMBLWGS:MBE0395892 PC=UP000642697:Unassembled WGS sequenceMSLETQDIIKRSATNTITPPPQARDYKAEVAKLIDVSSCVGCKACQVACSEWNDIRDEVGHCVGVYDNPADLSAKSWTVMRFSETEQNGKLEWLIRKDGCMHCEDPGCLKACPSAGAIIQYANGIVDFQSEHCIGCGYCIAGCPFNIPRLNKEDNRVYKCTLCVDRVSVGQEPACVKTCPTGAIHFGTKQEMLEMGEQRVAKLKARGYEHAGVYNPQGVGGTHVMYVLHHADQPELYHGLPNEPKVDTSINLWKGALKPLAAAGFIATFAGLIYHYIGIGPNKEVDDDEEDHHE>UPI00049FD58D L-valine transporter subunit YgaH OS=Citrobacter amalonaticus OX=35703 GN=ygaH SS=EMBLWGS:MBE0394524 PC=UP000642697:Unassembled WGS sequenceMRYEVLLLGLLVGCVNYGFRYLPLRLKMGNTRPGKRGATGVLLDTIGIASICALLVVSTAPEVMHDASRFVPTLVGFAVLGASFYKTRSIIIPTLLSALAYGLAWKMLAVL>UPI00049FD5A2 DeoR/GlpR transcriptional regulator OS=Citrobacter amalonaticus OX=35703 GN=ILP74_19265 SS=EMBLWGS:MBE0397502 PC=UP000642697:Unassembled WGS sequenceMLDNLRRREQIIDLLCDQGSVRVEPLSVHFGVSSVTIRNDLRYLEQKGCVMRSYGGAVLNQHFALDRPLQDKDRLNRDVKSRIAEKAASFVKDGDTLILDSGSTTTLIPPLLKSRRDLVVMTNALNIAWELANFERVDVMILGGNVRQSVYSLYGPSAEHQLRQYRFDKLFLGVDGFCLEAGITTPHPGEAHLNQVMCQVAQEVTVVADSSKFGRKSFCMISEISGIDRVITDSGIPEHYHQALSQMGVDVVIVDE>UPI00049FD5E5 Glutathione-dependent disulfide-bond oxidoreductase OS=Citrobacter amalonaticus OX=35703 GN=yghU SS=EMBLWGS:MBE0397373 PC=UP000642697:Unassembled WGS sequenceMSDNTYQPAKVWTWEKSNGGAFANINRPVSGPTHDKTLPVGKHPLQLYSLGTPNGQKVTIMLEELLAQGVSGAEYDAWLIRIGEGDQFSSGFVEVNPNSKIPALRDHSQNPPVRVFESGSILLYLAEKYGYFLPQDLAKRTETLNWLFWLQGAAPFLGGGFGHFYHYAPVKIEYAINRFTMEAKRLLDVLDKQLANNAYVAGDEYTIADMAIWPWFGNVVLGNVYDAAEFLDAGNYKNVQRWAKEIAERPAVKRGRIVNRTNGPLNEQLHERHDASDFETNTEDKRQN>UPI00049FD63A RidA family protein OS=Citrobacter amalonaticus OX=35703 GN=ILP74_09075 SS=EMBLWGS:MBE0395604 PC=UP000642697:Unassembled WGS sequenceMHPDITLNTTSHGLPPAGHYSASVTAGGFVFISGQLPITPHGEKKAGASFEEQTRQVLENVDACLKGAGVSRQHLVSVRVYVTDINQWPTFNDIYAEWIGDFRPSRVVAGVAELHYGSALEVEALALAQ>UPI00049FD71D TRNA pseudouridine(65) synthase TruC OS=Citrobacter amalonaticus OX=35703 GN=truC SS=EMBLWGS:MBE0394432 PC=UP000642697:Unassembled WGS sequenceMLEILYQDEWLVAVNKPSGWLVHRSWLDRDEKVVVMQTVRDQIGQHVFTAHRLDRPTSGVLLMGLSSEAGRRLAQQFEQHQIHKRYHAIVRGWLMEEAVLDYPLVEELDKIADKFARENKDPQPAVTHYRGLATVEMPVATGRYPTTRYGLVELDPQTGRKHQLRRHLAHLRHPIIGDSKHGDLRQNRSAAEHFGCNRLMLHASQLSLTHPFTGEPLTIHAGLDTVWMQALSQFGWQGLLPDNERVELSEPAGQDERIRSSFRE>UPI00049FD729 PadR family transcriptional regulator OS=Citrobacter amalonaticus OX=35703 GN=ILP74_19025 SS=EMBLWGS:MBE0397456 PC=UP000642697:Unassembled WGS sequenceMRHHHEGCCKGEGHHHGGCEHRHGRGGGGGRRQRFFGHGELRLVILDILSREASHGYELIKAIENLTQGNYTPSPGVIYPTLDFLQDQAFIAVSEEDGGRKKITLTDTGAQWLEENREHLGHIQERVNARNVGFELRKNPQMKRALENFKAVLDLRVNQGDISDAQLKKIIGVIDRAALEITQLD>UPI00049FD783 Alanine transaminase AlaA OS=Citrobacter amalonaticus OX=35703 GN=alaA SS=EMBLWGS:MBE0394930 PC=UP000642697:Unassembled WGS sequenceMSPIEKSSKLDNVCYDIRGPVLKEAKRLEEEGNKVLKLNIGNPAPFGFDAPDEILVDVIRNLPTAQGYSDSKGLYSARKAIMQHYQARGMRDVTVEDIYIGNGVSELIVQAMQALLNSGDEMLVPAPDYPLWTAAVALSGGNAVHYLCDESSDWFPDLDDIRAKITPRTRGIVIINPNNPTGAVYSKELLMEIVEIARQNDLIIFADEIYDKILYDDAEHHSIAAMAPDLLTVTFNGLSKTYRVAGFRQGWMVLNGPKKHAKGYIEGLEMLASMRLCANVPAQHAIQTALGGYQSISEFIMPGGRLYEQRNRAWELINDIPGVSCVKPRGALYMFPKIDAKRFNIHDDQKMVLDFLLQEKVLLVQGTAFNWPWPDHVRIVTLPRIDDIEMSLSKFARFLSGYHQL>UPI00049FD7E3 NADPH-dependent FMN reductase OS=Citrobacter amalonaticus OX=35703 GN=ssuE SS=EMBLWGS:MBE0396360 PC=UP000642697:Unassembled WGS sequenceMRVITLAGSPRFPSRSSALLEYAREKLNGQDVEVYHWNLHNFEPEDLLYARFDSPALKTLVAQLKEADGLIVATPVYKAAYSGALKTLLDLLPERALEGKVVLPLATGGTVAHLLAVDYALKPVLSALKAQEILHGVFADDSQVIDYQHKPHFTPNLQLRLDKALETFWHALHRRDVKVPAFDTVRGAAHA>UPI00049FD850 C4-dicarboxylate TRAP transporter substrate-binding protein OS=Citrobacter amalonaticus OX=35703 GN=ILP74_01415 SS=EMBLWGS:MBE0394180 PC=UP000642697:Unassembled WGS sequenceMNKIIAVLITVCTFFLPFTIQAKPLSIKVAYENNPGEPLDVVMRYWADILNKKSNGEITLVLYPSSQLGSKQDVTEQAMMGMNVITLTDVAFLADYEPDLGILFGPYLTDDPQKLFKIYESDWFRQKNEDLKKKGIHVVMNNYLYGTRQIISKKPIRKVEDLAGMKIRVPNNVMQIKAIQAMGATPTPMPLGEVYPALTQGVIDGVENPISVLQGQKLFEQAKYLSMVNYLTNTSVWIGGEAFFSTLSPEQLELIHSTGYEAGLYSQKLTIERDAEMLKAMEAEGVEVIYPDTEAFRQKARDVYTQFPEWTPGLYETIQQQLQ>UPI00049FD8AC Uncharacterized protein OS=Citrobacter amalonaticus OX=35703 GN=ILP74_18390 SS=EMBLWGS:MBE0397336 PC=UP000642697:Unassembled WGS sequenceMHDVLNEKRSIQTIRDHTSIIATHAFKQHLLQVRTLDNVGMAAPEDFHFIVTFKDDVDNADATVKPLAEAVLLGDRVRFIVKPAKQYPELTKVMAGFVEVIEHSIRRFFDTNGRIISTRKVNGSAEKWHLH>UPI00049FD908 Carbonic anhydrase OS=Citrobacter amalonaticus OX=35703 GN=ILP74_04730 SS=EMBLWGS:MBE0394787 PC=UP000642697:Unassembled WGS sequenceMKEIIDGFLKFQRKVFPERAELFRRLATQQNPRALFISCSDSRLVPELVTQREPGDLFVIRNAGNIVPSYGPEPGGVTASVEYAVSALRVSDIVICGHSDCGAMTAIAGCHCLDHMPAVNHWLRYADSARVVNESRMHTSLAEKTASMVRENVIAQLANIQTHPSVRLALEEGRIALHGWVYDIESAEIAAYDGASRQFVSLAKNPDTCAMPYRQPSAA>UPI00049FD9C5 Elongation factor 4 OS=Citrobacter amalonaticus OX=35703 GN=lepA SS=EMBLWGS:MBE0394606 PC=UP000642697:Unassembled WGS sequenceMKNIRNFSIIAHIDHGKSTLSDRIIQICGGLSDREMEAQVLDSMDLERERGITIKAQSVTLDFKSADGETYQLNFIDTPGHVDFSYEVSRSLAACEGALLVVDAGQGVEAQTLANCYTAMEMDLEVVPVLNKIDLPAADPERVAEEIEDIVGIDATDAVRCSAKTGVGVTDVLERLVRDIPPPQGDPDGPLQALIIDSWFDNYLGVVSLVRIKNGTLRKGDKIKVMSTGQTYNADRLGIFTPKQVDRTELKCGEVGWLVCAIKDILGAPVGDTLTQARNPAEKALPGFKKVKPQVYAGLFPVSSDDYENFRDALGKLSLNDASLFYEPESSTALGFGFRCGFLGLLHMEIIQERLEREYDLDLITTAPTVVYEVETTAKETIYVDSPSKLPPLNNIYELREPIAECHMLLPQAYLGNVITLCIEKRGVQTNMVYHGNQVALTYEIPMAEVVLDFFDRLKSTSRGYASLDYNFKRFQASDMVRVDVLINNERVDALALITHRDNSQSRGRELVEKMKDLIPRQQFDIAIQAAIGTHIIARSTVKQLRKNVLAKCYGGDISRKKKLLQKQKEGKKRMKQIGNVELPQEAFLAILHVGKDSK>UPI00049FDABD Tetratricopeptide repeat-containing protein OS=Citrobacter amalonaticus OX=35703 GN=ILP74_09375 SS=EMBLWGS:MBE0395662 PC=UP000642697:Unassembled WGS sequenceMRSLADFEFNKAPLCDGMILASESIRLDFPSQTVYDELERLVSLAQEEISQLLSQDEQLEKLLALFYGEWGFTDTRGVYRLSDALWLDQVLKNRQGSAVSLGAILLWIANRLSLPLVPVIFPTQLILRIESLEGEMWLINPFNGETLNEHTLEVWLKGNISPVAELFNEDLDEADNAEVIRKLLDTLKSSLMEEQQMELALRASEALLQFNPEDPYEIRDRGLIYAQLECEHVALTDLSYFVEQCPEDPISEMIRAQINNISHKQIVLH>UPI00049FDB73 MFS transporter OS=Citrobacter amalonaticus OX=35703 GN=ILP74_11505 SS=EMBLWGS:MBE0396060 PC=UP000642697:Unassembled WGS sequenceMSQNKAFSTPFFLAVICIYLSYFLHGISVITLAQNMTSLAAKFSTDSAGIAYLISGIGLGRLVSILFFGVLSDKFGRRAIILLGAALYILFFFGIPASPNLMVAFVLAVCVGVANSALDTGGYPALMECFPKASGSAVILVKAMVSFGQMLYPMLVGYMLLNNIWYGYGVIIPGILFVLITLMLLRSQFPGQLVDASVAKELPQMNSKPLVWLEGVASVMFGVAAFSTFYVIVVWMPKYAMAFAGMEEADALKTITYYSLGSLVCVFIFAALLKKMVRPIWANVFNAGLATVTAAVIYLWPSPLVCNAGAFVIGFSAAGGILQLGVSVMSEFFPKSKAKVTSVYMMMGGLANFVIPLITGYLSTIGLQYIILLDFAFALLAFITGIIVFVRYYRVFNIPQNDIRLGERYFSTKS>UPI00049FDB87 TRAP transporter substrate-binding protein OS=Citrobacter amalonaticus OX=35703 GN=ILP74_01185 SS=EMBLWGS:MBE0394135 PC=UP000642697:Unassembled WGS sequenceMKLTKTLLNLCVGTALVLAAQAASAQTLRAADVHPADYPNVVAVKHMGEKLSAATDGRLDIKTFPGGVLGDEKQMIEQAQLGAIDIIRVSMSPVAAILPEINVFTLPYIFRDEDHLHKVLDGAIGQEIGDRLTANSQSRLVFLGWMDAGTRNLITKAPVVKPEDLKGMKIRVQTSPVSLDTLKAMGANAIAMGTSEVFSGMQTGVIDGTENNPPTFVAHNYLPVAKNYTWSKHFIIPELFLFSKAKWDKLKKEDQELIIKLAKEAQIEQRQLWEAYNAKSLETMKANGVNFHDIDTDYFYKATQPVRDQYGKDHQDLIKRIQDVK>UPI00049FDBA6 DNA-binding transcriptional regulator KdgR OS=Citrobacter amalonaticus OX=35703 GN=kdgR SS=EMBLWGS:MBE0395570 PC=UP000642697:Unassembled WGS sequenceMANADLDKQPDSVSSVLKVFGILQALGEEREIGITELSQRVMMSKSTVYRFLQTMKTLGYVSQEGESEKYSLTLKLFELGARALQNVDLIRSADIQMRELSRLTKETVHLGALDEDSIVYIHKIDSMYNLRMYSRVGRRNPLYSTAIGKVLLAWRDRDEVKQILDGVEYKRSTGRTITTTEALLPVLDKVREQGYGEDNEEQEEGLRCIGVPVFDRFGVVIAGLSISFPTLRFSEEKLHDYVSMLHTAARKISEQMGYNDYPF>UPI00049FDC07 Aspartate carbamoyltransferase OS=Citrobacter amalonaticus OX=35703 GN=pyrB SS=EMBLWGS:MBE0394130 PC=UP000642697:Unassembled WGS sequenceMANPLYQKHIISINDLSRDDLNLVLATAAKLKANPQPELLKHKVIASCFFEASTRTRLSFETSMHRLGASVVGFSDSANTSLGKKGETLADTISVISTYVDAIVMRHPQEGAARLATEFSGKVPVLNAGDGSNQHPTQTLLDLFTIQETQGRLNNLHVAMVGDLKYGRTVHSLTQALAKFDGNRFYFIAPDALAMPQYILDMLDEKGIAWSLHASIEEVMAEVDILYMTRVQKERLDPSEYANVKAQFVLRASDLVGARENMKVLHPLPRIDEIATDVDKTPHAWYFQQAGNGIFARQALLALVLTSDLAL>UPI00049FDCC1 Adenylate kinase OS=Citrobacter amalonaticus OX=35703 GN=adk SS=EMBLWGS:MBE0396823 PC=UP000642697:Unassembled WGS sequenceMRIILLGAPGAGKGTQAQFIMEKYGIPQISTGDMLRAAVKSGSELGKQAKDIMDAGKLVTDELVIALVKERIAQEDCRNGFLLDGFPRTIPQADAMKEAGIVVDYVLEFDVPDELIVDRIVGRRVHAASGRVYHIKFNPPKVEGKDDVTGEELTTRKDDQEETVRKRLVEYHQMTAPLIGYYQKEAAAGNTQYAKVDGTQAVADVRAALEKILG>UPI00049FDCFB Exopolyphosphatase OS=Citrobacter amalonaticus OX=35703 GN=ppx SS=EMBLWGS:MBE0394674 PC=UP000642697:Unassembled WGS sequenceMPIHDKTPRPQEFAAVDLGSNSFHMVIARVVDGAMQIIGRLKQRVHLADGLGEDNMLSEEAMERGLSCLSLFAERLQGFAPSSVCIVGTHTLRQAQNATDFLKRAEKVIPYPIEIISGNEEARLIFMGVEHTQPEKGRKLVIDIGGGSTELVIGENFEPKLVESRRMGCVSFAQIYFPGGVISKENFQRARMAAAQKLETLTWQFRIQGWNVALGASGTIKAAHEVLLEMGEKDGFITPERLDRLVTELLQHRSFESLSLPGLSEERKAVFVPGLAILCGVFDALAIRELRLSDGALREGVLYEMEGRFRHQDVRSRTASSLANQYNIDSEQARRVLETTMQMYDQWQTQQPKLAHPQLEALLKWAAMLHEVGLNINHSGLHRHSAYILQNSDLPGFNQEQQTMMATLVRYHRKAVKLDDLPRFTLFKKKQFLPLIQLLRLGVLLNNQRQATTTPPTLTLITDDNHWTLRFPHDWFSQNALVLLDLEKEQQYWEAVTGWRLKIEEERSPEIAA>UPI00049FDD60 Universal stress protein UspE OS=Citrobacter amalonaticus OX=35703 GN=uspE SS=EMBLWGS:MBE0395769 PC=UP000642697:Unassembled WGS sequenceMAMYQNMLVVIDPNQDDQPALRRAVYLHQRIGGKIKAFLPIYDFSYEMTTLLSPDERTAMRQGVISQRTAWIREQAKYYIEAGVPVEIKVVWHNRPFEAIIQEVISGGHDLVLKMAHQHDRLEAVIFTPTDWHLLRKCPSPVWMVKDQPWPEGGKALVAVNLASEEPYHNALNEKLVKETLQLAEQVNHTEVHLVGAYPVTPINIAIELPEFDPSVYNDAIRGQHLLAMKALRQKFSIDEKVTHVEKGLPEEVIPDLAEHLQAGIVVLGTVGRTGISAAFLGNTAEQVIDHLRCDLLVIKPDQYQTPVELDDEEDD>UPI00049FDD93 MgtC/SapB family protein OS=Citrobacter amalonaticus OX=35703 GN=ILP74_15530 SS=EMBLWGS:MBE0396813 PC=UP000642697:Unassembled WGS sequenceMNTLSFLLFGEGESLLITLGKICVAFILGGIIGLERESKGKPVGFKTCVIISVASCVLTIVSIQSAEYYAEISMNIRSDPMRLAAQIISGVGFLGAGVILHRHDDAISGLTTAAIVWASAGVGITSGAGFYMHALLATGLFLLAIKLSYFVIFLQTKNQLPGKVKIRIILDEKSGLQTLVESINQQKNIIEAITIRDVKKGKIEVNLKVVIRKKMTLPELYNNLSTLEHVCAIALEH>UPI00049FDE2A 3-phosphoserine/phosphohydroxythreonine transaminase OS=Citrobacter amalonaticus OX=35703 GN=serC SS=EMBLWGS:MBE0396389 PC=UP000642697:Unassembled WGS sequenceMAQVFNFSSGPAMLPADVLKLAQKDLCDWNGLGTSVMEVSHRGKEFIQVAQEAEQDFRDLLNIPSNYKVLFCHGGGRGQFSAIPLNILGDKTTADYVDAGYWAASAVKEAKKYCTPNVIDAKVSVDGLRGVKPMSEWQPSADAAYLHYCPNETIDGIAINETPNFGSDVVVTADFSSTILSGPLDVSRYGIIYAGAQKNIGPAGLTIVIVREDLLGKANVACPSILDYSVLNDNDSMFNTPPTFAWYLSGLVFKWLKANGGVAAMDKINQQKAELLYGVIDNSDFYRNDVAKANRSRMNVPFQLADSALDKLFLEESFAAGLHALKGHRVVGGMRASIYNAMPLEGVKALADFMTDFERRRG>UPI00049FDEBF PTS IIA-like nitrogen regulatory protein PtsN OS=Citrobacter amalonaticus OX=35703 GN=ptsN SS=EMBLWGS:MBE0397571 PC=UP000642697:Unassembled WGS sequenceMINNDTTLQLSSVLNQECTRSAVHCQSKKRALEIISELAAKQLSLPPQVVFEAILTREKMGSTGIGNGIAIPHGKLEEDTLRAVGVFVQLETPIAFDAIDNQPVDLLFALLVPADQTKTHLHTLSLVAKRLADKTICRRLRAAQSDEELYQIITDTEGGQDEA>UPI00049FDFC8 DUF1883 domain-containing protein OS=Citrobacter amalonaticus OX=35703 GN=ILP74_09400 SS=EMBLWGS:MBE03
[truncated: 1,200,000 more chars]
