# Supplementary material for: Proteomic and metabolomic analysis reveals new insights into quaternary amine metabolism in Citrobacter amalonaticus CJ25
Source: mSphere. 2025 Aug 25;10(9):e00421-25. doi: 10.1128/msphere.00421-25 (PMC12482172; doi:10.1128/msphere.00421-25)
Supplement: Supplemental figures — Figures S1 through S6. [file msphere.00421-25-s0005.docx]

Supplementary information


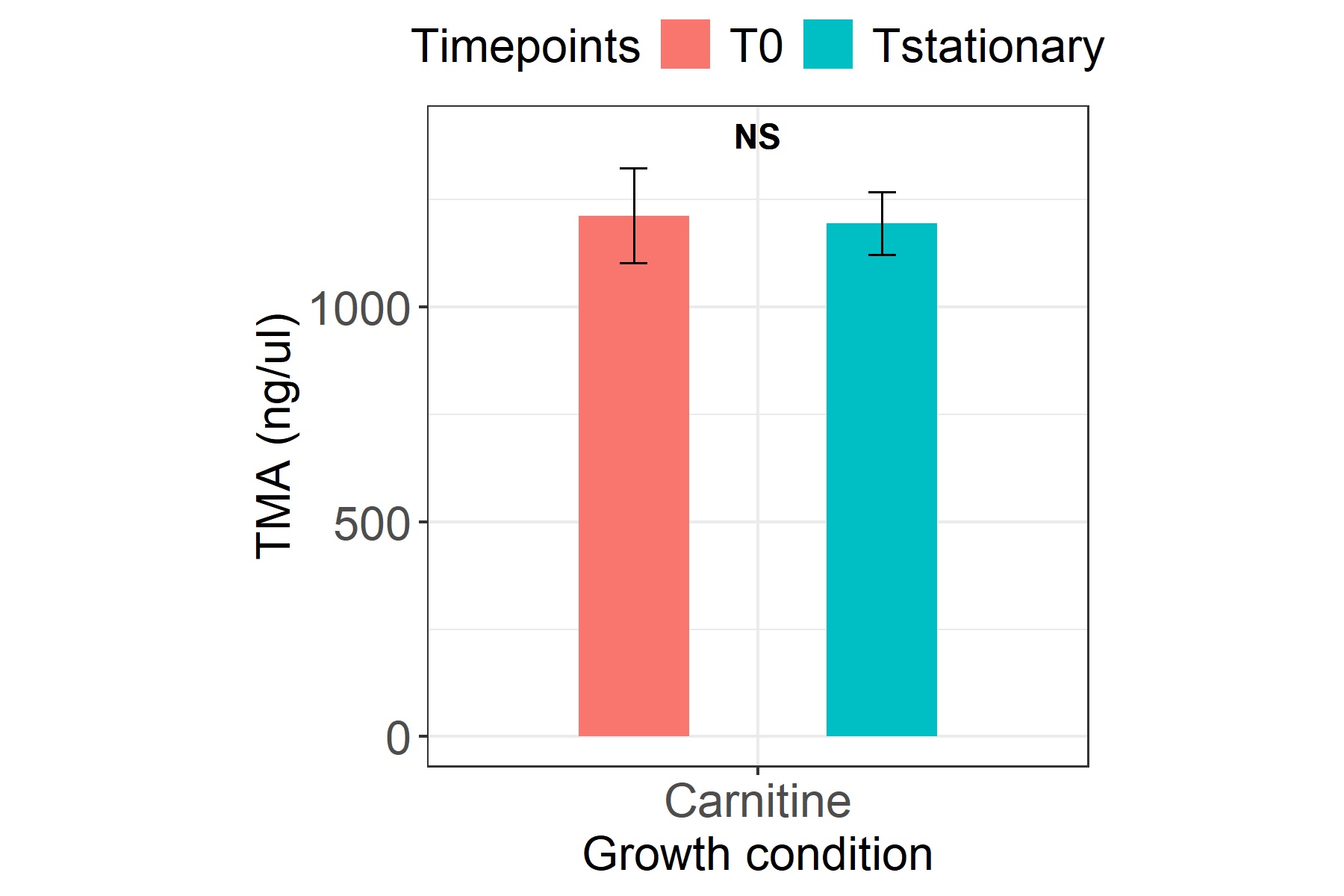


Figure S1: Absolute concentration of TMA (ng/ul) compared between carnitine and glucose growth conditions.


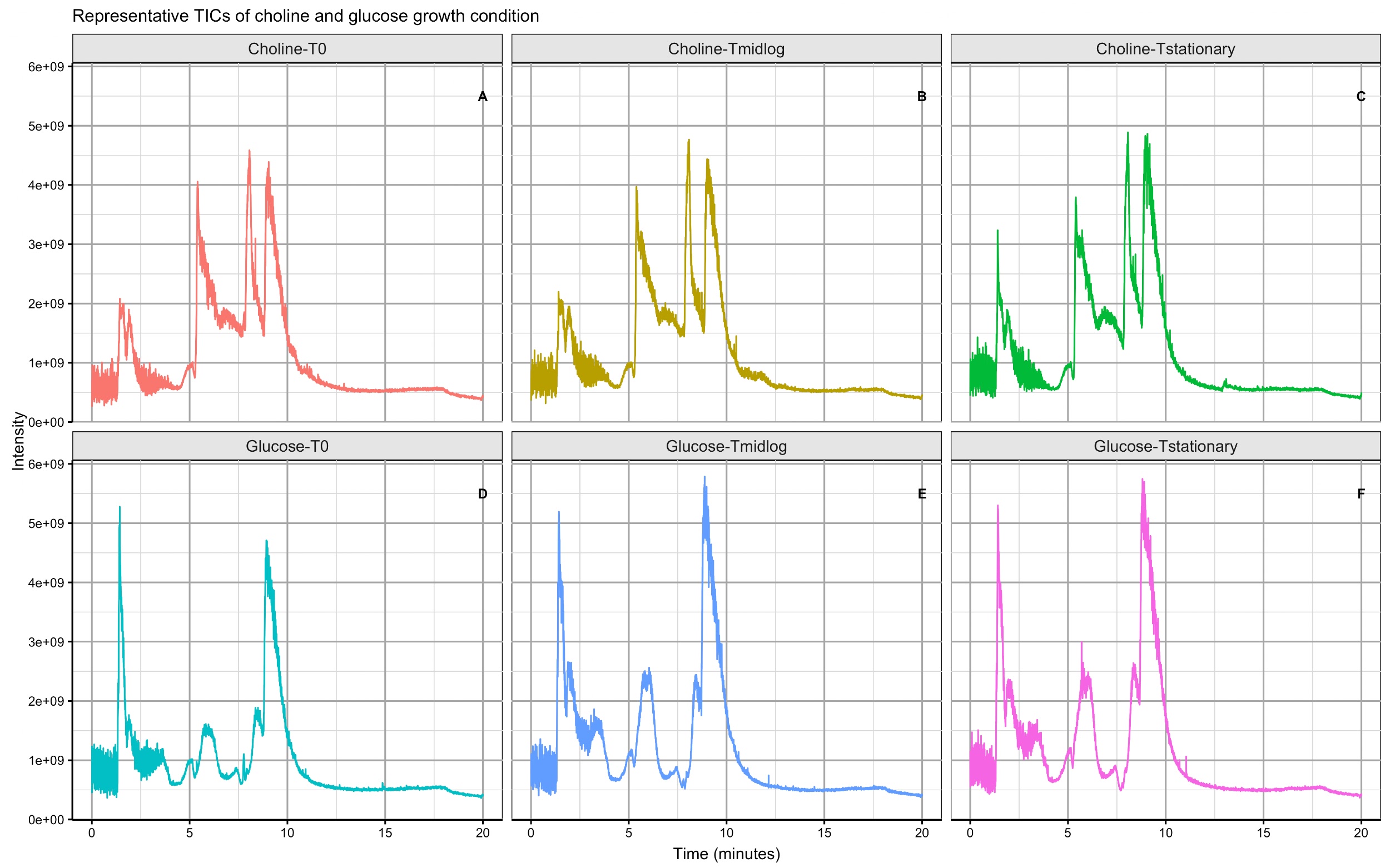


Figure S2 Representative Total Ion chromatogram (TICs) of choline and glucose growth conditions at three different time points.


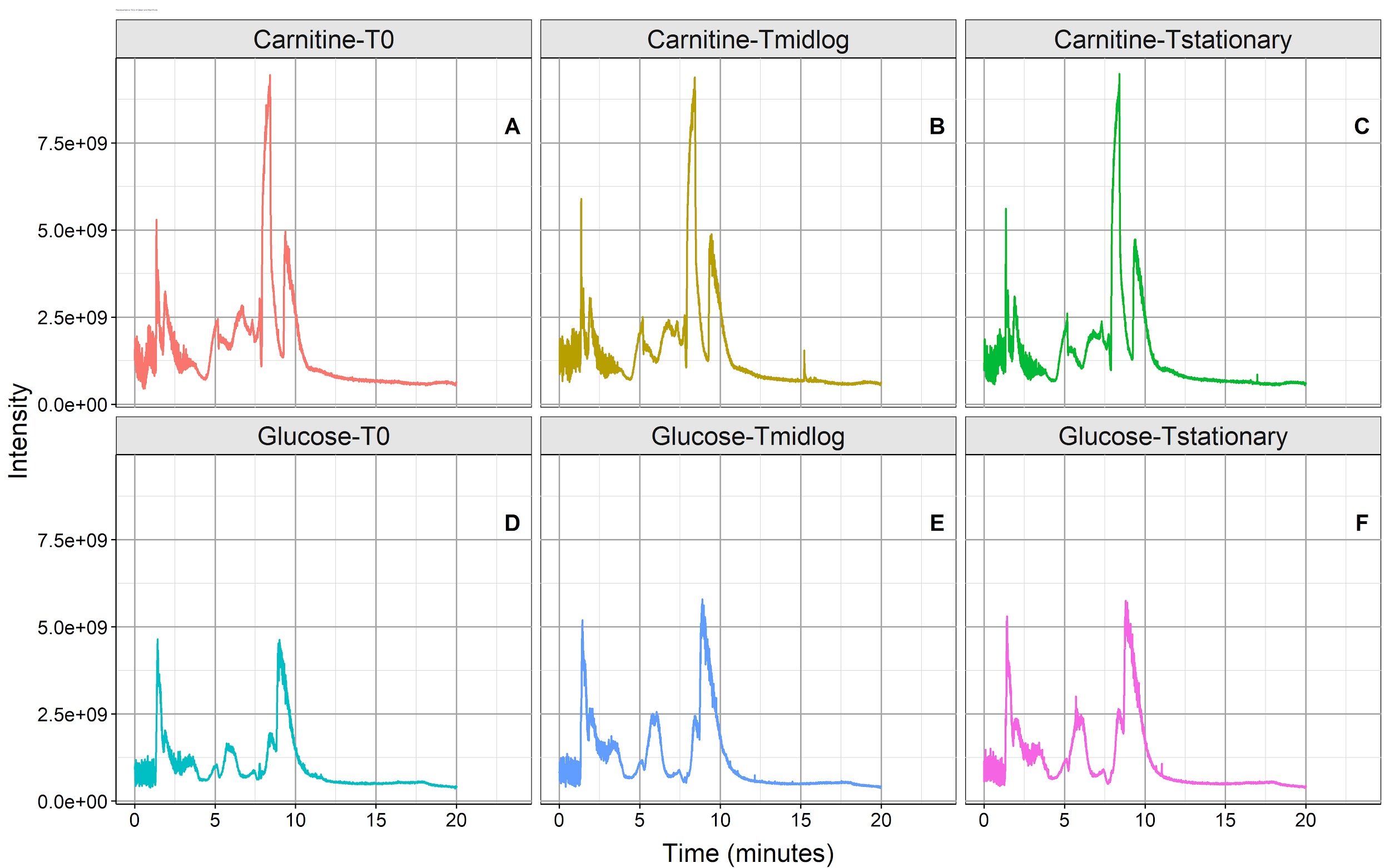


Figure S3 Representative Total Ion chromatogram (TICs) of carntine and glucose growth conditions at three different time points.


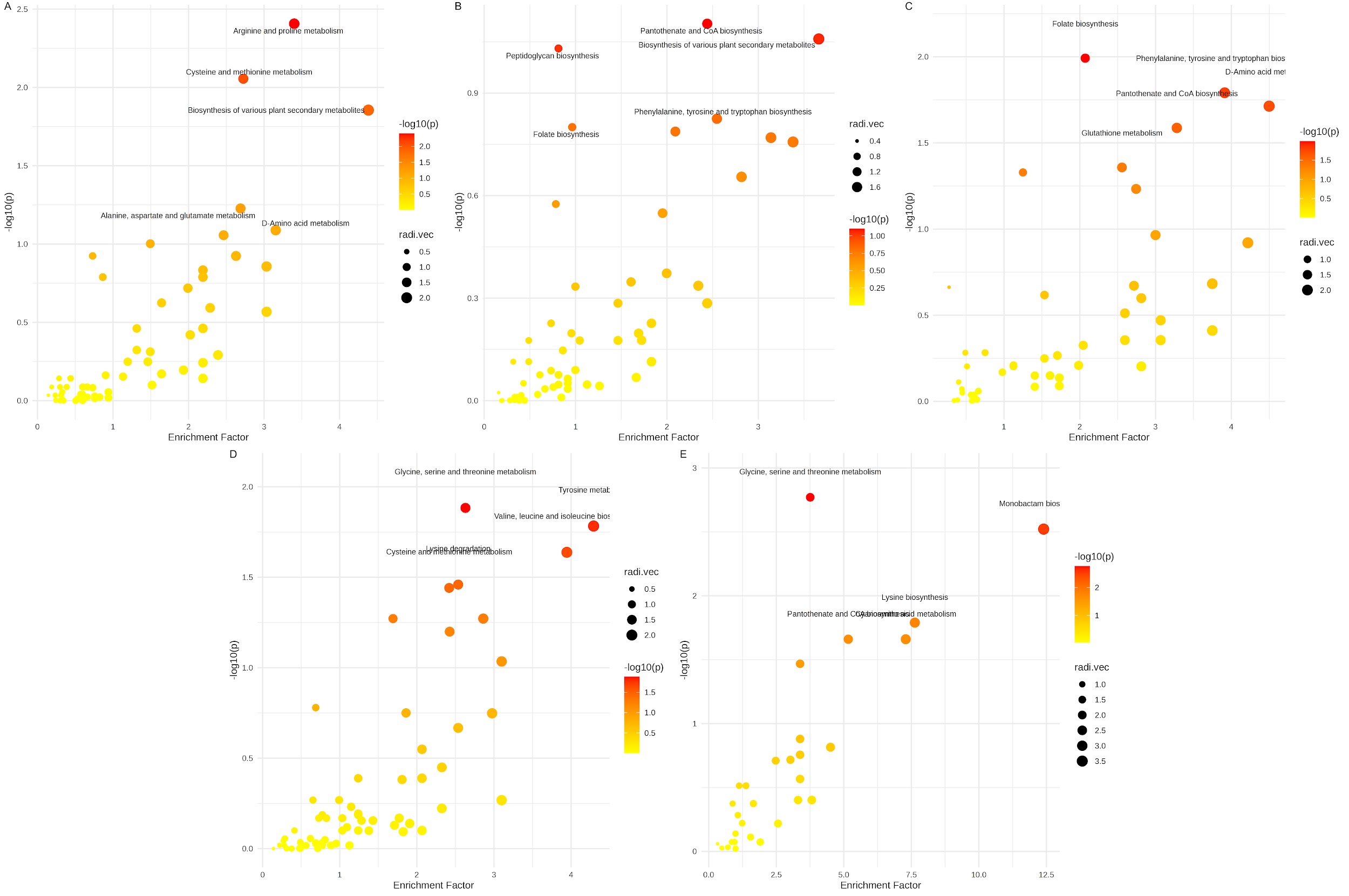


Figure S4 Functional analysis of metabolites in mid-log of growth conditions (A) choline and glucose, (B) carntine and glucose, different time points (T0 vs Tmidlog) for each growth condition.(C) choline, (D) carnitine, and (E) glucose. The color, varying from yellow to red, indicates the metabolites are present in the data with different levels of significance. The radi. vec (dot size) represents the pathway impact. The enrichment factor of a pathway is calculated as the ratio between the number of significant pathway hits and the expected number of compound hits within the pathway.


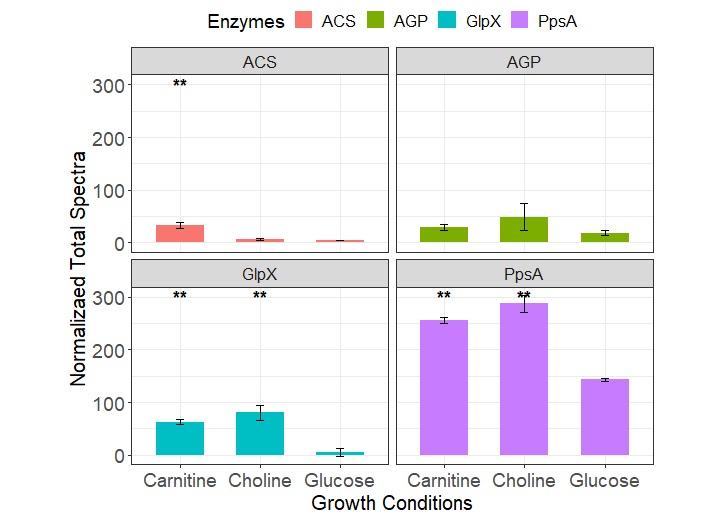


Figure S5 Relative abundance of various proteins involved in gluconeogenesis, such as phosphoenolpyruvate synthetase (PpsA), fructose 1,6-bisphosphatase (GlpX), glucose-1-phosphatase (Agp), and acetyl-CoA synthetase(ACS) at different growth conditions: choline, carnitine vs glucose. “**” represents p-value<0.05 compared to the glucose growth condition.


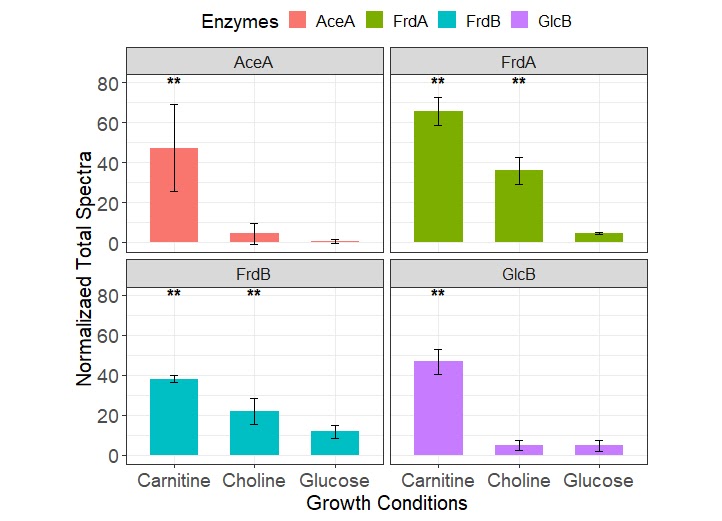


Figure S6 Relative abundance of various proteins involved in glyoxylate cycle, such as isocitrate lyase (AceA), malate synthase (GlcB), fumarate reductase (FrdB), and succinate dehydrogenase (FrdA) at different growth conditions: choline, carnitine vs glucose. “**” represents p-value<0.05 compared to the glucose growth condition.
