# Supplementary material for: Proteomic and metabolomic analysis reveals new insights into quaternary amine metabolism in Citrobacter amalonaticus CJ25
Source: mSphere. 2025 Aug 25;10(9):e00421-25. doi: 10.1128/msphere.00421-25 (PMC12482172; doi:10.1128/msphere.00421-25)
Supplement: Legends — for Files S1 through S4. [file msphere.00421-25-s0006.docx]

Supplementary file legends

**Supplementary File 1**: Excel spreadsheet listing the 5664 metabolic features identified by Compound Discoverer exported from the positive mode, in which 1403 (24.7%) features have tentative IDs.

**Supplementary File 2**: Excel spreadsheet listing the differentially produced proteins from *Citrobacter amalonaticus* CJ25 harvested at mid-log for choline vs glucose presented as normalized total spectra with their putative annotations. The replicates are listed as A, B, and C for each substrate.

**Supplementary File 3**: Excel spreadsheet listing the differentially produced proteins from *Citrobacter amalonaticus* CJ25 harvested at mid-log for carnitine vs glucose presented as normalized total spectra with their putative annotations. The replicates are listed as A, B, and C for each substrate.

**Supplementary File 4**: Text file of the *Citrobacter amalonaticus* CJ25 proteome.
